# Substrate-Controlled Rh(III)-Catalyzed Regioselective Synthesis of Benzimidazole-5-carboxylic Acid with Iodonium Ylide toward Benzimidazole-Fused Isochromenes and Isoquinolines

**DOI:** 10.1021/acs.joc.5c02744

**Published:** 2026-06-23

**Authors:** Yu-Tung Cheng, Chen-Ting Liao, Ganesh P. Pawar, Chai-Lin Kao, Chung-Ming Sun

**Affiliations:** † Department of Applied Chemistry, 34914 National Yang-Ming Chiao-Tung University,1001 Ta-Hsueh Road, Hsinchu 300-10, Taiwan; ‡ Department of Medicinal and Applied Chemistry, 38023 Kaohsiung Medical University, 100, Shih-Chuan First Road, Kaohsiung 807-08, Taiwan; § Institute of Microbial Technology (CSIR), Chandigarh 160036, India

## Abstract

A rhodium­(III)-catalyzed substrate-controlled regiodivergent cascade [4 + 2] annulation of benzimidazole-5-carboxylic acids with iodonium ylides has been developed for the efficient synthesis of tetracyclic benzimidazo-isocoumarin and pentacyclic benzimidazo-isoquinoline frameworks. The regioselectivity of C­(sp^2^)–H activation is modulated by the electronic nature of 2-substituents groups on the benzimidazole core, wherein both the 5-carboxylic acid and the imidazole moiety act as directing groups. Iodonium ylides serve as carbene precursors and C2 synthon in this transformation. The detailed substrate scope study and control experiments support the proposed mechanism.

## Graphical abstract

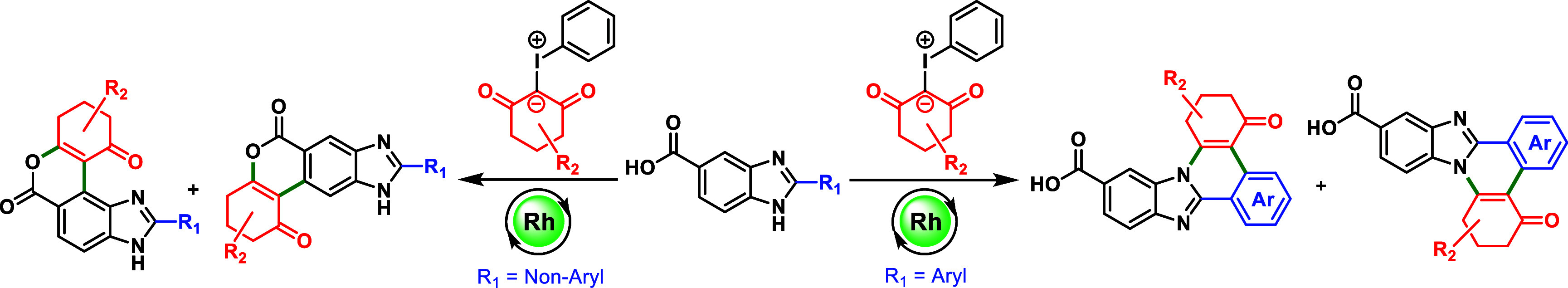

## Introduction

Molecular hybridization is an effective strategy for obtaining novel chemical entities, enabling the exploration of uncharted biological spaces that play a significant role in drug discovery.^1^ In this approach, two chemically distinct pharmacophore motifs are combined into a single chemical entity through linking, fusing, or merging methods.^2^ The resulting hybrid molecule may retain the pharmacological effects of the individual motifs and can function as a “one drug–multiple targets” agent.^3^

Benzimidazole, a privileged structure, has emerged as one of the most therapeutically important moieties in drug discovery. Benzimidazole derivatives exhibit a broad spectrum of bioactivities, including antibacterial, antiviral, anticancer, antidiabetic, antimalarial, anti-HIV, and antifungal properties.^4,5^ Beyond their therapeutic significance, these derivatives have also found applications in material science, catalysis, and the agricultural industry.^6–9^ The versatility of benzimidazole derivatives across diverse fields has spurred the search for novel strategies for their functionalization.

Numerous reports have documented the *ortho* C–H functionalization of the aryl ring in 2-arylbenzimidazoles (Scheme 1). For instance, Kamal and colleagues reported the *ortho*-hydroxylation of 2-arylbenzimidazoles via Pd­(OAc)_2_-catalyzed C–H activation.^10^ Similarly, the research groups of Vinayak and Qiao achieved copper- and palladium-catalyzed, benzimidazole-directed *ortho*-nitration of the aryl ring.^11,12^ Recently, Tang disclosed a rhodium-catalyzed *ortho*-C­(sp^2^)–H activation/acylalkylation reaction using benzimidazole as a directing group,^13^ while Huang demonstrated the substrate-controlled divergent synthesis of benzimidazole-fused quinolines and spirocyclic benzimidazole-fused isoindoles using benzimidazole and diazo compounds via Rh catalysis.^14^

Recently, directing group-assisted, transition-metal-catalyzed C–H activation/cyclization has emerged as a powerful strategy for constructing fused heterocycles (Scheme 1).^15–18^ In this context, 2-arylbenzimidazoles have been fused with diverse coupling partners through *ortho*-C­(sp^2^)–H activation and N–H annulation. For example, Ru-, Ni-, and Rh-catalyzed reactions of 2-arylbenzimidazoles with alkynes via dual N–H and *ortho*-C–H activation afford benzimidazoisoquinolines.^19–23^ similar products have also been prepared from reactions with alkenes.^24–27^ Guo reported a Pd-catalyzed coupling between 2-arylbenzimidazoles and benzimidazoles to form benzimidazoquinazolines, .^28^ while other groups employed maleimides and styrene compounds as coupling partner to synthesize benzimidazoisoquinolines.^29–32^

In all reported methods to date, the reactive *ortho*-C–H bond of the aryl ring has been employed for benzimidazole functionalization. Notably, only one example exists in the literature where alkenylation of *N*-protected 2-arylbenzimidazoles was achieved through the activation of the nonreactive C4–H bond.^33^

The therapeutic importance of functionalized benzimidazole derivatives, combined with the demand for novel strategies to diversify this privileged scaffold, prompted us to develop a method for activating and functionalizing the inert C4–H bond in benzimidazole-carboxylic acid derivatives. We hypothesized that a transition-metal-catalyzed reaction between benzimidazole-carboxylic acid and iodonium ylide guided by COOH-directed C4–H activation and annulation would furnish fused benzimidazole-based heterocycles.

Herein, we report a rhodium-catalyzed regioselective C–H activation/cyclization cascade between benzimidazole-carboxylic acids and iodonium ylides, enabling efficient access to fused benzimidazo-fused isocoumarin and benzimidazo-isoquinoline derivatives.

## Results and Discussion

To evaluate the feasibility of our proposed strategy, 2-benzyl-1*H*-benzo­[*d*]­imidazole-5-carboxylic acid **1a** and iodonium ylide **2a** were selected as model substrates. Initial screening with 2.5 mol % Ru or Pd catalysts in the presence of 25 mol % of AgOAc as an additive in HFIP solvent at 80 °C for 6 h failed to deliver the desired product, and the starting material **1a** was recovered (Table 1, entries 1–3). However, when [Cp*IrCl_2_]_2_ (2.5 mol %) was used in the same reaction condition, TLC analysis revealed the formation of two new spots (Table 1, entry 4). Isolation and spectroscopic characterization confirmed the formation of two distinct products: 2-phenyl-3,8,9,10-tetrahydrobenzo­[3,4]­isochromeno­[5,6-*d*]­imidazole-6,11-dione (**3a**, 14% yield) and 9-phenyl-2,3,4,8-tetrahydrobenzo­[3,4]­isochromeno­[6,7-*d*]­imidazole-1,6-dione (**4a**, 15% yield). The structure of **3a** was supported by 1H NMR spectrum, which displayed two characteristic doublets at 8.21 ppm (*J* = 8.6 Hz) and 7.89 ppm (*J* = 8.6 Hz), corresponding to the C6–H and C7–H protons, whereas 13C NMR spectrum exhibited the lactone carbonyl carbon signal at 171.7 ppm. In addition, the 1H NMR spectrum of compound **4a** showed two singlets at 9.17 and 8.38 ppm for C4–H and C7–H protons and 13C NMR spectrum of the same compound showed a lactone carbonyl carbon signal at 169.8 ppm. The structures of **3g** and **4a** were unambiguously confirmed by X-ray crystallographic analysis (Figure 1).

**Table 1:**
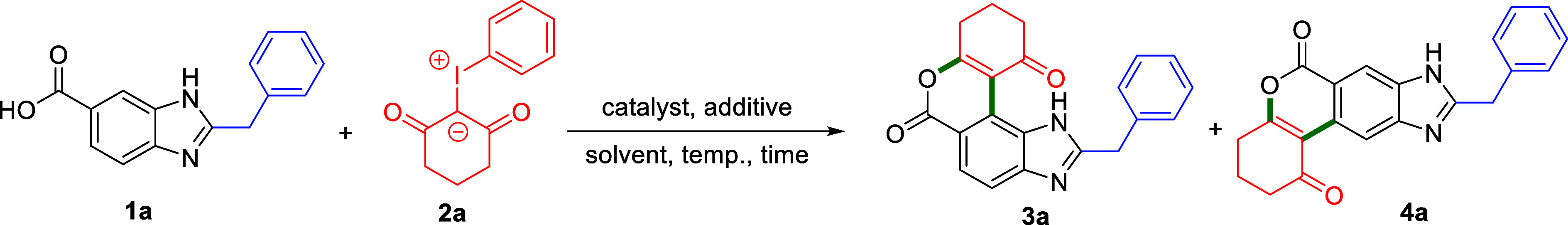
Optimization of Reaction Conditions^a^

						yield^b^	
entry	catalyst	additive	solvent	temp (°C)	Time	**3a**	**4a**
1	[Cp*RuCl_2_]_2_	AgOAc	HFIP	80	6	0	0
2	[(*p*-cymene)RuCl_2_]_2_	AgOAc	HFIP	80	6	0	0
3	Pd(OAc)_2_	AgOAc	HFIP	80	6	0	0
4	[Cp*IrCl_2_]_2_	AgOAc	HFIP	80	6	14	15
5	[Cp*RhCl_2_]_2_	AgOAc	HFIP	80	6	28	52
6	[Cp*RhCl_2_]_2_	AgOTf	HFIP	80	6	10	26
7	[Cp*RhCl_2_]_2_	AgSbF_6_	HFIP	80	6	13	30
8	[Cp*RhCl_2_]_2_	NaOAc	HFIP	80	6	22	37
9	[Cp*RhCl_2_]_2_	Zn(OAc)_2_	HFIP	80	6	15	26
10	[Cp*RhCl_2_]_2_	Cu(OAc)_2_	HFIP	80	6	0	0
11	[Cp*RhCl_2_]_2_	AgOAc	TFE	80	6	54	33
12	[Cp*RhCl_2_]_2_	AgOAc	MeOH	80	6	44	12
13	[Cp*RhCl_2_]_2_	AgOAc	EtOH	80	6	45	11
14	[Cp*RhCl_2_]_2_	AgOAc	DMF	80	6	3	22
15	[Cp*RhCl_2_]_2_	AgOAc	Toluene	80	6	0	0
16	[Cp*RhCl_2_]_2_	AgOAc	DCE	80	6	0	0
17	[Cp*RhCl_2_]_2_	AgOAc	TFE	90	6	**51**	**34**

a Reaction conditions: **1a** (0.1 mmol), **2a** (0.12 mmol), catalyst (2.5 mol %), additive (25 mol %), solvent (0.6 mL), sealed tube·.

b Isolated Yield.

**Figure 1:**
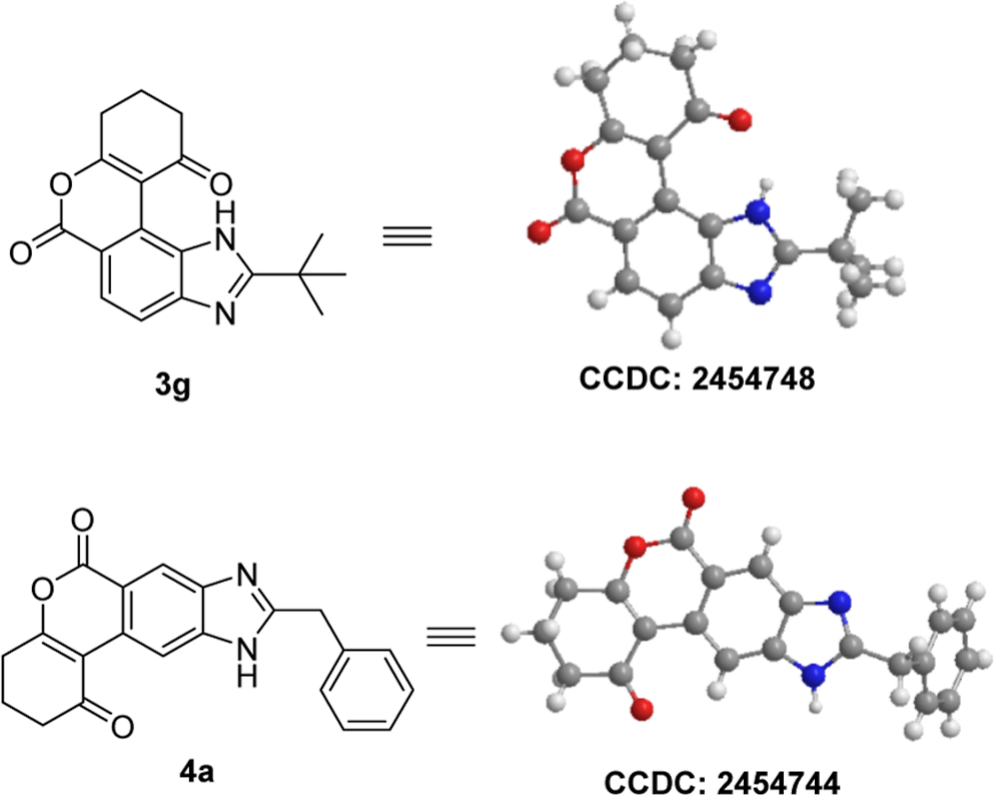
ORTEP diagram of **3g** and **4a**.

When [Cp*RhCl_2_]_2_ was employed under the same reaction condition, the desired products **3a** and **4a** were obtained in 28 and 52% yields (Table 1, entry 5), respectively. Screening of various additives, including AgOTf, AgSbF_6_, NaOAc, Zn­(OAc)_2_, and Cu­(OAc)_2_, failed to improve the yields of **3a** and **4a** (Table 1, entries 6–10). Next, an evaluation of polar protic solvents, such as TFE, MeOH and EtOH, revealed that TFE was the most suitable solvent, affording desired products **3a** and **4a** in 54 and 33% yields, respectively (Table 1, entries 11–13). Screening of aprotic solvents such as DMF, toluene, and DCE did not improve the product yields (Table 1, entry 14–16). Next, evaluation of the reaction temperature revealed an effect on the **3a** and **4a** formation. Increasing the reaction temperature to 90 °C resulted in 51 and 34% yields, respectively (Table 1, entry 17).

With the established reaction condition in hand, the substrate scope of benzimidazole-5-carboxylic acid (**1**) bearing alkyl and nonaryl (R^1^) substituents at the C2-position was investigated (Table 2). Accordingly, 2-benzyl-1H-benzo­[*d*]­imidazole-5-carboxylic acid **1a** reacted smoothly with iodonium ylide **2a**, delivering the desired products **3a** and **4a** in 51 and 34% yields, respectively. Switching the benzyl group with a phenethyl moiety **1b** afforded the annulated products **3b** and **4b** in good yields. The reaction was then extended to a cycloalkyl-containing substrate **1c**, delivering the desired product **3c** and **4c** in 40 and 46% yields, respectively. Benzimidazole-5-carboxylic acid **1** bearing alkyl C2 substituents such as propyl **1d** and methyl **1e** afforded the desired products (**3d**, **3e**, **4d**, and **4e**) in good yield. Benzimidazoles **1** bearing electron-withdrawing substituents such as trifluoromethyl **1f** and 2,6-dichlorophenyl **1i** reacted smoothly with **2a** to furnish the desired products (**3f**, **4f**, **3i**, and **4i**) in good yields. In addition, bulky substituted *tert*-butyl **1g** and mesityl **1h** groups reacted well with **2a** to furnished cyclic products (**3g**, **4g**, **3h**, and **4h**) in good to excellent yields. Notably, substrates **1f, 1g**, and **1i** exhibited unusual reactivity, leading to the formation of dual C–H activated adducts **7** (Table S1). This behavior may be attributed to competitive Rh-chelation between the carboxylic acid and imidazole nitrogen. The presence of either bulky substituents (**1g** and **1h**) or electron-withdrawing groups (**1f** and **1i**) likely suppresses coordination of the imidazole nitrogen to the Rh center, thereby enhancing the directing ability and reactivity of the carboxylic acid group. Benzimidazole C2–substituted halogen such as chloro **1j** and bromo **1k** were well tolerated and delivers compound **3j**, **4j**, **3k**, and **4k** in appreciable yield. Furthermore, C2-substituted phenyl thioether substrate **1l** and methyl thioether substrate **1m** smoothly underwent cyclization to afford the corresponding products **3l**, **4l**, **3m**, and **4m** in moderate to good yields. Notably, substrates bearing benzoyl (**1n**) and 1-hydroxyethyl (**1o**) substituents delivered the desired products **3n**, **4n**, **3o**, and **4o** in low yields. This outcome can be attributed to disruption of the catalytic cycle through the coordination of the imidazole nitrogen together with the alcohol or ketone oxygen, leading to the formation of a stable five-membered metallacyclic complex. Gratifyingly, the C2-ethoxy-substituted substrate **1p** furnished products **3p** and **4p** in excellent yields. C2-unsubstituted substrate **1q** and C2-amino-substituted substrate **1r** afforded the corresponding products in moderate to low yields, likely due to the poor solubility of the starting materials under the reaction conditions. Furthermore, C2-furan-2-yl benzimidazole **1s** also delivered the annulated products **3s** and **4s** in low yield, consistent with diminished reactivity of this heteroaryl system. In addition, C2-trans-alkenyl substrates exhibited sluggish reactivity, as (*E*)-styryl **1t** and (*E*)-prop-1-en-1-yl **1u** afforded the corresponding products **3t**, **4t**, **3u**, and **4u** in poor yields. In contrast, the corresponding cis isomer, (*Z*)-prop-1-en-1-yl substrate **1v**, showed significantly enhanced reactivity, delivering products **3v** and **4v** in moderate yield. To further explore the scope of the C–H annulation reaction, the benzimidazole moiety **1** was replaced with a benzo­[*d*]­oxazole framework. The C2-benzyl-substituted substrate **1x** and C2-phenyl-substituted benzoxazole acid **1y** were subsequently evaluated, and both substrates showed diminished reactivity. The corresponding products **3x**, **4x**, **3y**, and **4y** were obtained in low yields, along with significant formation of dual C–H-activated side product **7** (Table S1).

**Table 2:**
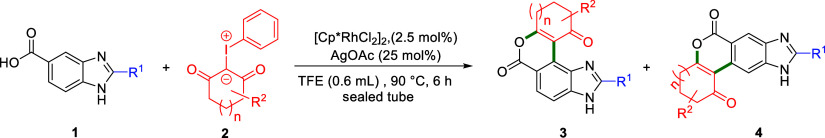

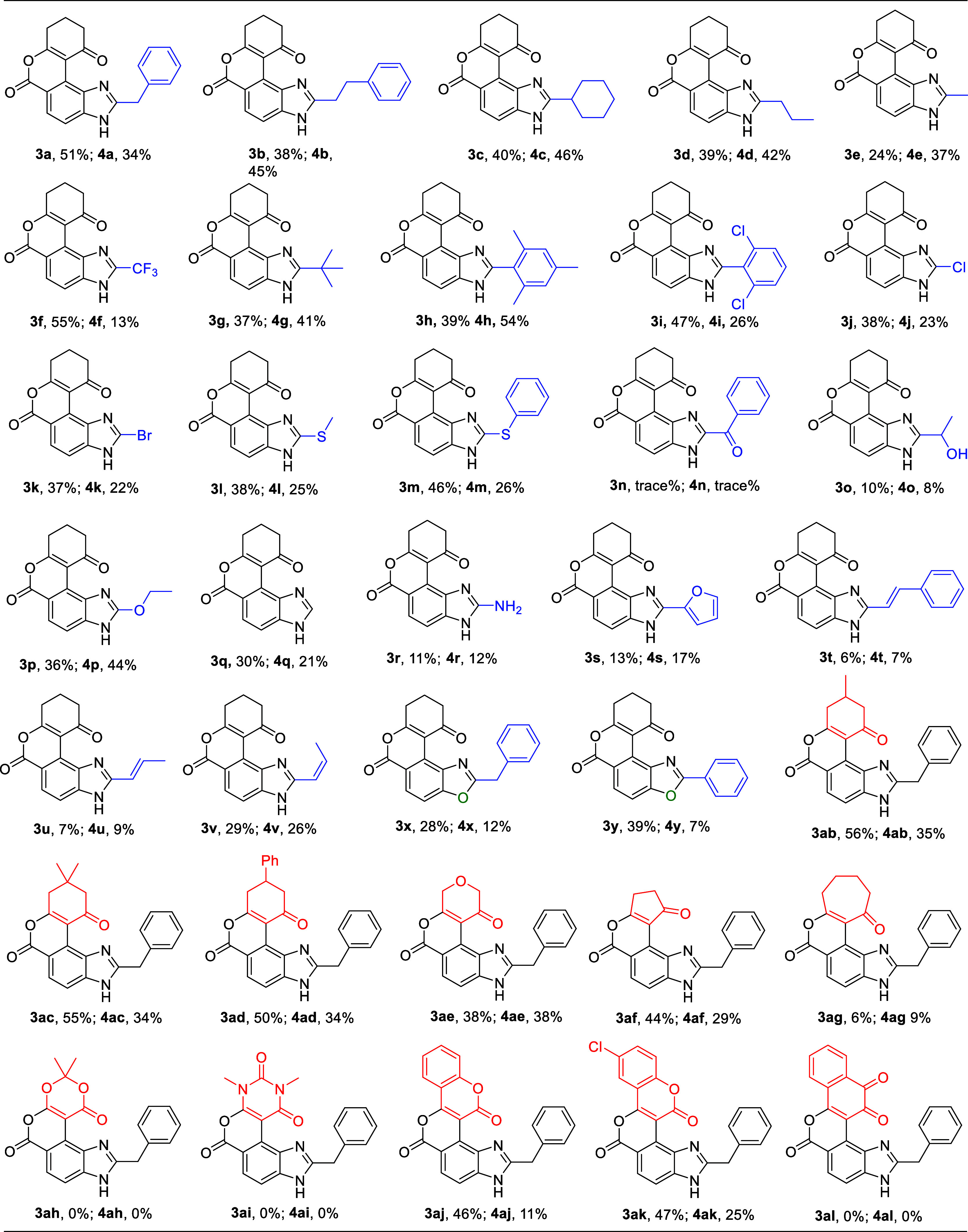
Substrate Scope for the Synthesis of Benzimidazo-Isocoumarin Derivatives

a Reaction conditions: **1a** (0.1 mmol), **2a** (0.12 mmol), [Cp*RhCl_2_]_2_ (2.5 mol %), AgOAc (25 mol %), in a sealed tube, TFE (0.6 mL), 6 h, 90 °C.

**Table 3:**
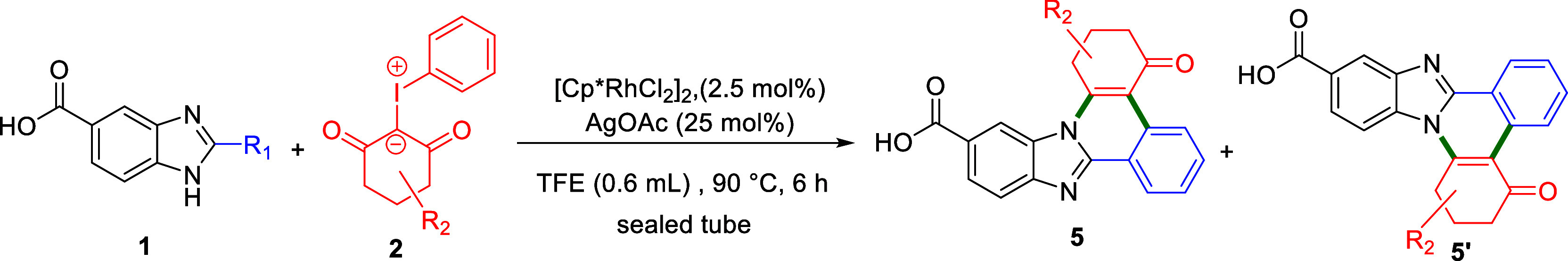

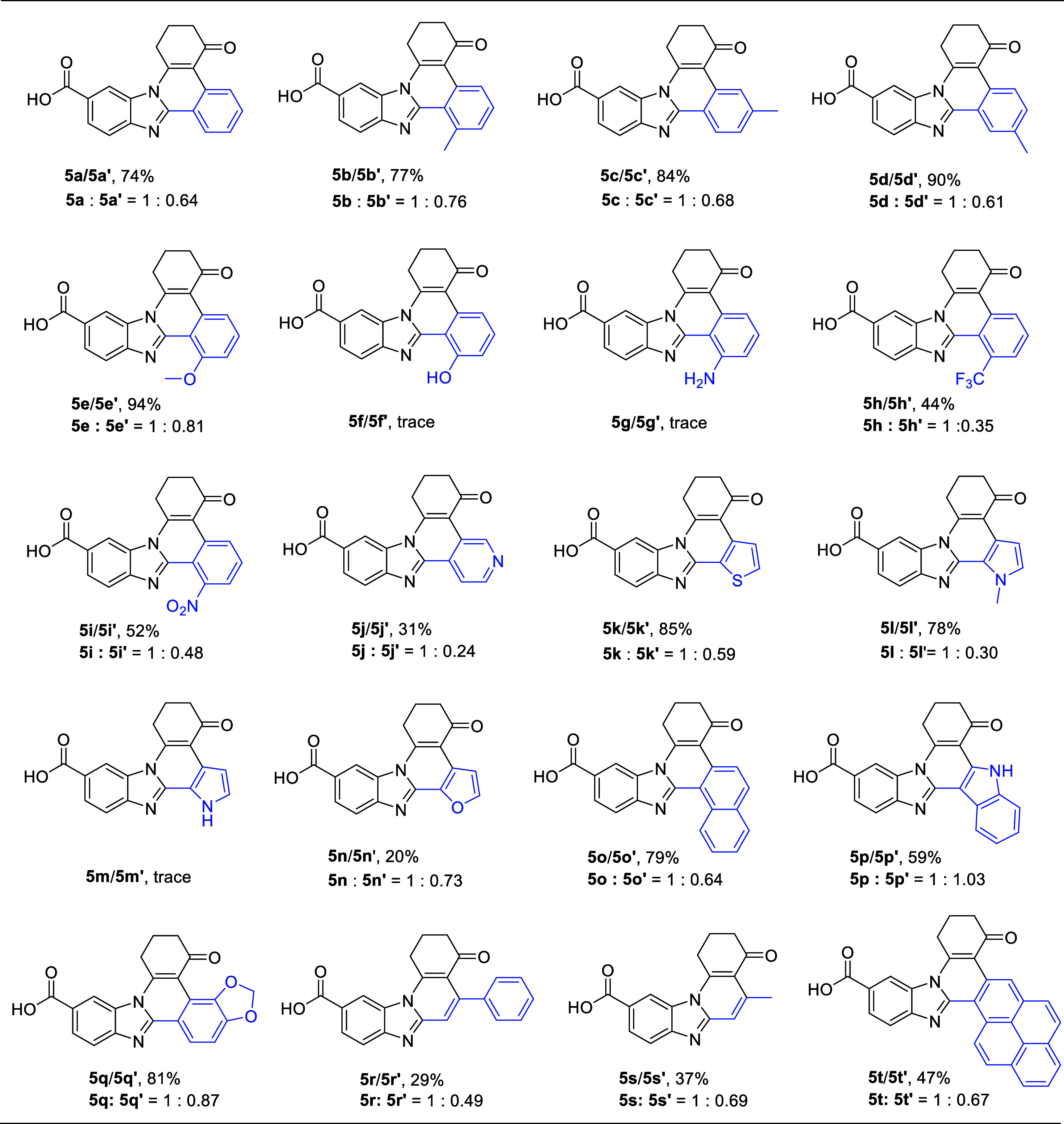
Substrate Scope for the Synthesis of the Benzimidazo-Isoquinoline Derivative

a Reaction conditions: **1a** (0.1 mmol), **2a** (0.12 mmol), [Cp*RhCl_2_]_2_ (2.5 mol %), AgOAc (25 mol %), in a sealed tube, TFE (0.6 mL), 6 h, 90 °C.

Next, the scope of the iodonium ylides was investigated by varying the *R* ^2^ in Table 2. Phenyl iodonium ylides of 1,3-cyclohexanedione bearing methyl **2b**, dimethyl **2c**, and phenyl **2d** substituents at the 5-position smoothly react with **1a**, affording the desired products **3ab–3ad** and **4ab–4ad** in good yields. The O-heterocyclic iodonium ylide **2e** underwent a smooth transformation to furnish **3ae** and **4ae** each in 38% yields. The five-member cyclic iodonium ylide **2f** smoothly participated in the reaction affording **3af** and **4af** in 44 and 29% yields. Whereas the seven-membered cyclic iodonium ylide **2ag** did not react efficiently with **1a** to afford **3ag** and **4ag** in poor yields, due to the seven-membered ring being unstable and prone to decomposition. Notably, phenyl iodonium ylides derived from Meldrum’s acid **2h** and 1,3-dimethylbarbituric acid **2i** failed to react with **1a** to deliver products (**3ah**, **4ah**, **3ai**, and **4ai**). This is probably due to the nature of the acetal and urea moieties, which are susceptible to decomposition under the reaction conditions. The bulky and lactone containing iodonium ylide of chromane-2,4-dione **2j** and its chloro-substituted analog **2k** were well tolerated to deliver the desired products (**3aj**, **4aj**, **3ak**, and **4ak**) in moderate to good yield. However, the similarly structured iodonium ylide of naphthalene-1,2,4­(3H)-trione **2l** failed to react with **1a**, and no desired products (**3l** and **4l**) were obtained, which is likely due to the instability of the 1,2-dione group.

Next, the reactivity of 2-phenylbenzimidazole-5-carboxylic acid **1z** with **2a** was examined to probe the regio-selectivity of C–H activation. Surprisingly, the expected products were not obtained; instead, two new polar compounds exhibiting fluorescence were observed. Isolation and their spectroscopic analysis confirmed the formation of inseparable regioisomers 4-oxo-1,2,3,4-tetrahydrobenzo­[4,5]­imidazo­[1,2-*f*]­phenanthridine-12-carboxylic acid **5a** and 4-oxo-1,2,3,4-tetrahydrobenzo­[4,5]­imidazo­[1,2-*f*]­phenanthridine-11-carboxylic acid **5a′** in 74% yield along with dual *ortho* C–H activation product **6a** in 21% yield (Table S1). The proton NMR spectrum of **5a**/**5a′** showed singlets at 8.82 and 8.45 ppm corresponding to C4–H proton and doublets at 8.14 (*J* = 8.5 Hz) and 8.00 ppm (*J* = 8.8 Hz) corresponding to C6–H protons. Moreover, the 13C NMR spectrum exhibited the presence of carbonyl carbon of carboxylic acid at 167.5 and 167.3, respectively. These results showed the C–H activation at the *ortho*-position of the C2-substituted phenyl ring, rather than at the C4–H and C6–H positions of the benzimidazole core. This outcome suggests that the imine moiety acts as an internal directing group for Rh-catalyzed ortho C–H activation. The regioselectivity of the reaction is likely governed by the significant difference in metal-binding affinity between the carboxylic acid and imidazole functionalities. In substrates bearing a suitable C–H activation site, particularly C­(sp^2^)-hybridized aryl substituents, the imidazole unit preferentially acts as the dominant directing group and promotes exclusive ortho C–H activation on the aryl moiety. Consequently, this pathway selectively affords products **5**/**5′** rather than **3/4**.

Next, the scope of 2-arylbenzimidazoles was investigated (Table 3). Methyl substitution on the phenyl ring at *ortho*, *para*, and *meta* positions (**1aa**-**1ac**) successfully reacted with **2a** to afford mixtures of regio-isomers **5b**/**5b′** (1:0.76), **5c**/**5c′** (1:0.68), and **5d**/**5d′** (1:0.61) in high yield (77–90%). When employing an electron-donating methoxy **1ad** group on the phenyl ring, it enhanced the reactivity, affording the desired products **5e**/**5e′** (1:0.81) with a yield of 94%. This is due to increased electron density of the aromatic ring and enhanced reactivity of the nitrogen atom toward Rh chelation. However, the phenyl ring with hydroxy **1ae** and amino **1af** groups at the *ortho*-position produced only traces of compounds **5f/5f′** and **5g**/**5g′**, due to the trapping of the Rh catalyst. In contrast, substrates bearing electron-withdrawing substituents, such as ortho-CF_3_, ortho-NO_2_, and 4-pyridyl groups **(1ag–1ai)**, afforded the annulated products **5h**/**5h′**, **5i**/**5i′**, and **5j**/**5j′** in relatively low yields (31–52%). Notably, secondary C–H activation ortho to the carboxylic acid group was also observed in these cases. For the CF_3_-substituted substrate **1ah**, uncyclized enol intermediate **8h** (Table S1) was generated after the initial C–H activation step (40%). This outcome is likely attributable to the reduced nucleophilicity of imidazole nitrogen and increased steric hindrance by the *ortho*-CF_3_ substituent.

Apart from the substituted 2-phenyl benzimidazoles, other heterocyclic scaffolds were also explored. Substrates with C2-bearing thiophen-2-yl **1aj** and *N*-methyl-pyrrol-2-yl **1ak** are well tolerated, and **5k**/**5k′** (1:0.59) and **5l**/**5l′** (1:0.3) are obtained in 85 and 78% yields, respectively. Notably, pyrrol-2-yl substrate **1al** without *N*-methyl substitution afforded the desired product **5m**/**5m′** in only trace yield. While furan-2-yl substrate **1s** reacts with **2a**, affording **5** *n*/**5n′** (1:0.73) in poor yield. We further explored substrates with larger steric profiles such as naphthalene **1am** and indole **1an** rings. Pleasingly, the targeted C–H annulation reactions proceeded smoothly under standard conditions, furnishing **5o**/**5o′** and **5p**/**5p′** in good to excellent yields (59–79%). Interesting results are observed on the substrate with phenyl fused with dioxol ring **1ao**, which is asymmetric with cyclized acetal at the *para* and *meta* positions. The substrate is of excellent reactivity due to electron-donating property of the alkoxy group, furnishing target compound **5q**/**5q′** (1:0.87) in 81% yield; the dual C–H activation product **6q**/**6q′** (Table S1) was also observed (17%). Moreover, substrates with C2-alkenyl group, such as (E)-styryl **1t** and (E)-prop-1-en-1-yl **1u** can also perform C–H activation and afford the desired product (**5r**/**5r′**, **5s**/**5s′**), albeit in lower yield (29–37%), along with the dual C–H activation side product **9** (Table S1), formed via the direction of the carboxylic acid group. This suggests that olefinic C–H has lower reactivity in our catalytic system. Notably, even substrates with extremely sterically encumbered pyren-1-yl **1ap** could lead to the expected product **5t**/**5t′** in fairly good yield.

**Scheme 1:**
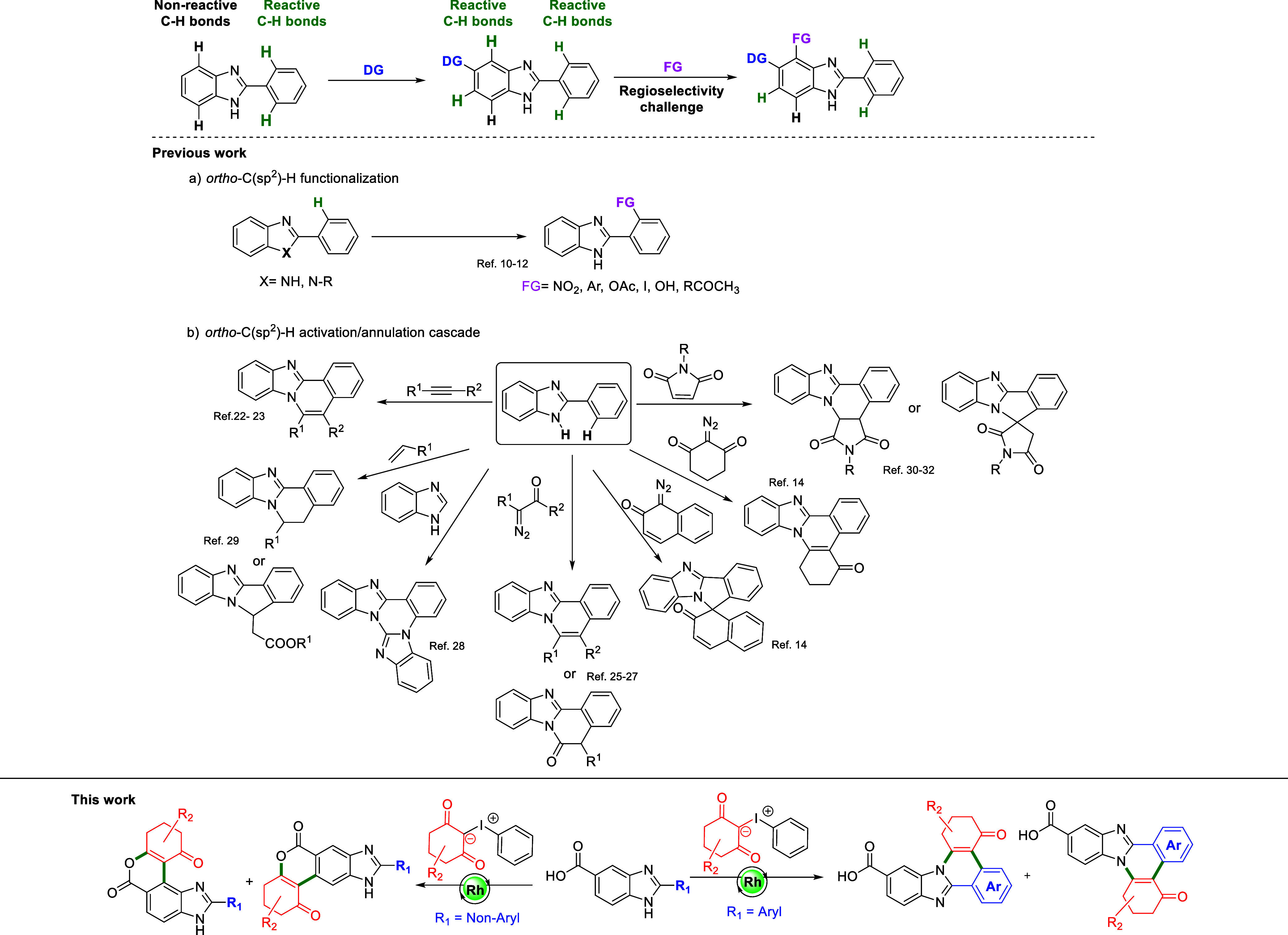
C–H Functionalization of 2-Aryl Benzimidazoles

Next, a gram–scale reaction was performed employing compound **1a** (1.98 mmol) and **2a** (2.38 mmol) under the standard reaction condition affording **3a** (295 mg, 43%) and **4a** (225 mg, 33%) (Scheme 2a). Moreover, the post–synthetic modification of the isochromeno-imidazole derivative **3a** was demonstrated (Scheme 2b). Oxidation of compound **3a** with IBX in DMSO at 80 °C for 2 h afford **3o** in 18% yield, in which the methylene of the benzyl moiety has been transformed into a carbonyl, while oxidation of **4a** results in a much higher yield (48%) of **4o**. This provided us with a roundabout method toward a target compound that cannot be obtained through the Rh-catalyzed reaction. Amidation of compound **3a** with tryptamine in a sealed tube at 60 °C for 24 h delivered the lactam 7-(2-(1*H*-indol-3-yl)­ethyl)-2-benzyl-3,8,9,10-tetrahydro-6*H*-imidazo­[4,5-k]­phenanthridine-6,11­(7*H*)-dione **10** in 84% yield. In addition, treatment of **3a** with sodium borohydride at room temperature for 24 h reduced the carbonyl of ketone into the alcohol derivative 2-benzyl-11-hydroxy-8,9,10,11-tetrahydrobenzo [3,4] isochromeno­[5,6-*d*] imidazole-6­(3*H*)-one **13** in 50% yield. While the reaction of **3a** with sodium cyanoborohydride and zinc iodide at 80 °C for 12 h results in deoxygenation of the ketone to afford alkane derivative **14** (20%), and alkene derivative **15** (23%) was presumably formed by the dehydration of alcohol **13**, a small amount of aromatized derivative **16** (6%) was also observed (See SI). Furthermore, the potential of one-pot transformation toward lactam derivatives **11** and **12** without isolation of lactones **3** and **4** had been investigated (Scheme 2c). Substrate **1a** and iodonium ylide **2a** were first reacted under standard conditions, after which 3 equiv of benzylamine was added, and the reaction tube was heated under seal for another 12 h. To our delight, all of **3a** and **4a** had been completely consumed and afforded lactam **11** and **12** in good yield (44 and 27%).

**Scheme 2:**
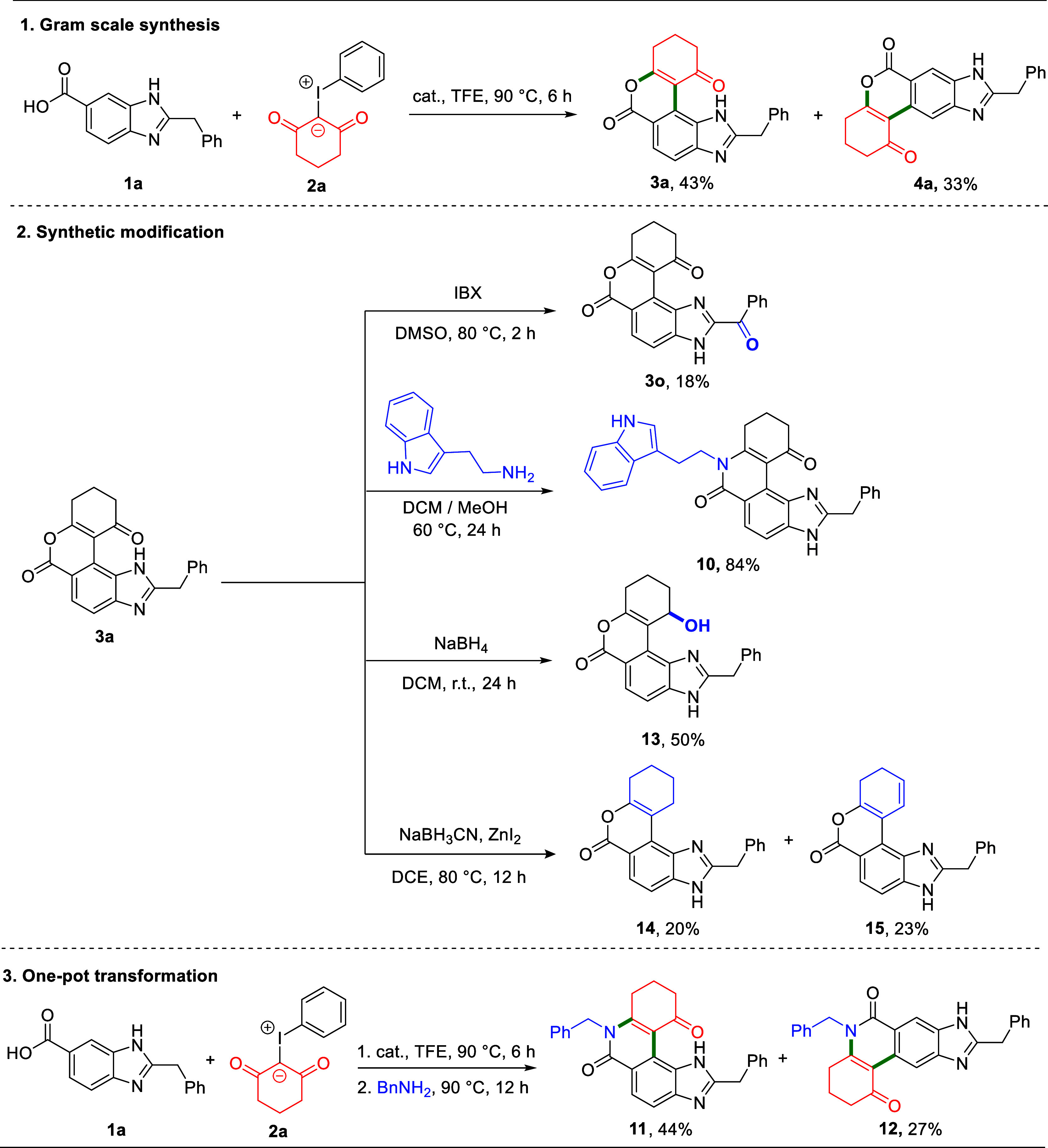
Synthetic Utility of Isochromeno-Imidazole Derivatives **3**

In order to gain mechanistic insight, a series of control experiments were conducted. First, the reaction of benzimidazole-5-carboxylic methyl ester 1a* with iodonium ylide **2a** under standard conditions showed no conversion, indicating that the ester group is ineffective as a directing group (Scheme 3a). Similarly, amide-functionalized compound **17** was treated with **2a** under optimized conditions and also failed to produce C–H annulated products, confirming that the amide group did not facilitate the transformation (Scheme 3b). Next, deuterium-labeling experiments were conducted with benzimidazole carboxylic acid **1z** under the optimized conditions in the presence of excess CD_3_OD, substantial *ortho* H/D exchange (95% D incorporation) was observed, indicating that the C–H bond activation step is reversible (Scheme 3c). Similarly, when **1a** was treated with CD_3_OD as the solvent under the optimized protocol, significant H/D exchange was observed at the C4–H (91%) and C6–H (88%) positions, further supporting the reversible nature of the C–H bond activation step (Scheme 3d). Furthermore, treatment of **1a** with the radical scavenger TEMPO under the standard conditions afforded the desired products with no significant decrease of yield, confirming that the transformation did not proceed via a radical pathway (Scheme 3e).

**Scheme 3:**
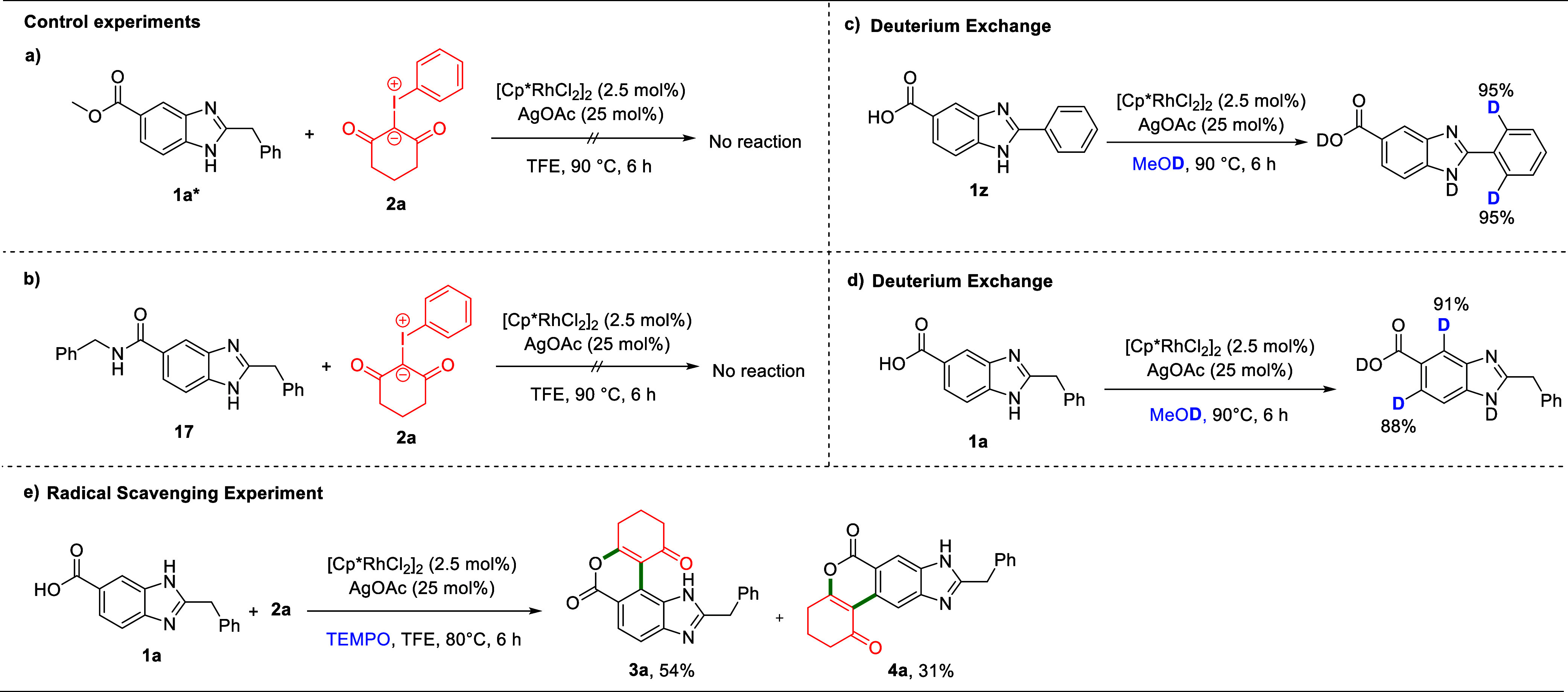
Control Experiments

Based on control experiments and literature study, a plausible reaction mechanism for the formation of **3a** and **5a** is proposed, as shown in Scheme 4. Initially, 2-benzyl-1H-benzo­[*d*]­imidazole-5-carboxylic acid **1a** undergoes C4–H and C6–H bond activation in the presence of the active Rh catalyst to generate the five-membered rhodacycle intermediate **A**. Similarly, 2-arylbenzimidazole-5-carboxylic acid **1z** undergoes *ortho*-C–H activation on the aryl ring to furnish intermediate **E**. Subsequently, iodonium ylide **2a** coordinates with rhodacycles **A** and **E**, followed by elimination of iodobenzene to generate the corresponding Rh–carbenoid intermediates **B** and **F**. These intermediates undergo migratory insertion to generate six-membered rhodacycles **C** and **G**, respectively, followed by protonolysis gives intermediates **D** and **H** with concurrent regeneration of the Rh catalyst. Finally, intermediates **D** and **H** undergo intramolecular nucleophilic addition, wherein the carboxylic acid (=O) and imidazole (=N) groups react with the conjugated enol via Michael addition. Subsequent dehydration leads to the formation [4 + 2]-annulation products.

**Scheme 4:**
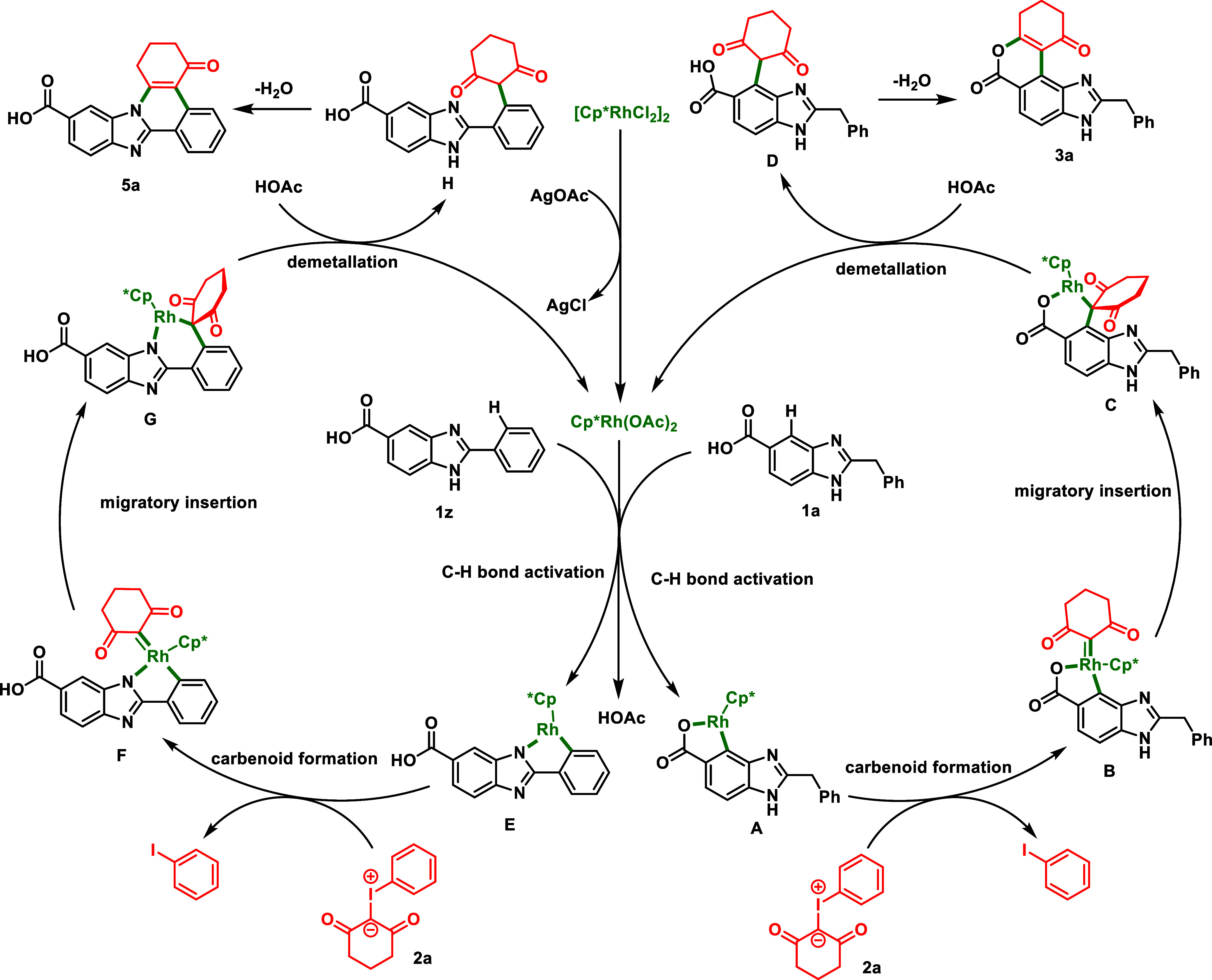
Proposed Mechanism for the Formation of **3a** and **5a**

## Conclusion

This work describes a Rh (III)-catalyzed, substrate-controlled regiodivergent [4 + 2] annulation that leverages the inherent competition between directing groups in benzimidazole-5-carboxylic acids. By reacting with iodonium ylides, the reaction selectively affords either benzimidazo-fused isochromenones or isoquinolines. Mechanistic studies highlight how the carboxylic acid and imidazole groups exert contrasting influences on regioselective C–H bond activation. Readily available materials, broad substrate scope, scalability, and postsynthetic derivatization demonstrate the method’s practicality. Overall, this approach provides a versatile route to complex benzimidazole compounds with potential utility in medicinal chemistry and functional materials development.

## Supplementary Material



## Data Availability

The data underlying this study are available in the published article and its Supporting Information

## Notes

Experimental Section with detailed experimental procedures, characterization data of all of the new compounds, copies of NMR spectra of the products, and X-ray data for compounds **3g** and **4a** (PDF)
