# Supplementary material for: Substrate-Controlled Rh(III)-Catalyzed Regioselective Synthesis of Benzimidazole-5-carboxylic Acid with Iodonium Ylide toward Benzimidazole-Fused Isochromenes and Isoquinolines
Source: J Org Chem. 2026 Jun 23;91(26):8727–36. doi: 10.1021/acs.joc.5c02744 (PMC13339643; doi:10.1021/acs.joc.5c02744)
Supplement: Supplementary file 1 [file jo5c02744_si_001.pdf]

# Supporting Information

## Substrate Controlled Rh(III)-Catalyzed Regioselective Synthesis of Benzimidazole-5-Carboxylic Acid with Iodonium Ylide toward Benzimidazole-Fused Isochromenes and Isoquinolines

Yu-Tung Cheng[1], Chen-Ting Liao[1] ,Ganesh Pawar[1,3], Chai-Lin Kao[2] and Chung-Ming Sun[1,2]*

[1] *Department of Applied Chemistry, National Yang-Ming Chiao-Tung University,1001 Ta-Hsueh Road, Hsinchu 300-10, Taiwan*

[2] *Department of Medicinal and Applied Chemistry, Kaohsiung Medical University, 100, Shih-Chuan 1st Road, Kaohsiung 807-08, Taiwan*

[3] *Institute of Microbial Technology (CSIR), Chandigarh 160036, India.*

E-mail: cmsun@nycu.edu.tw

**Table of Contents**

General Information ……………………………………………………………………S2
Side-Products for the C-H Annulation Reaction of Benzimidazole-5-Carboxylic Acids with Iodonium Ylides …………………………………………………………………......S2
Procedures for the C-H Annulation Reaction of Benzimidazole-5-Carboxylic Acids with Iodonium Ylides ……………………………………………………………………..S2
Procedures for the Preparation of the Substrates ……………………………………….S3
Procedures for Synthetic Transformations ..……..…………………………………......S14
Control Experiments ……………………………………………………………………..S17
Characterization Data ……………………………………………………………………S18
NMR Spectra ……………………………………………………………………………S64
X-Ray Crystallography Data ……………………………………………………….....S218
References ………………………………………………………………………………...S220

## General Information

All the reactions were carried out under air unless specified. All the reactions that require heating were heated by silicone oil bath. The $^{1}H$ NMR spectra were recorded on a 400 MHz or 600 MHz NMR spectrometer. The $^{13}C$ NMR spectra was recorded at 101 MHz or 151 MHz. The $^{19}F$ NMR spectra were recorded at 376 MHz or 564 MHz. The residual solvent signals were used as references and the chemical shifts were converted to the TMS scale. The coupling constants were given in Hz. HRMS data were obtained using an ESI+ mode. The conversion of starting materials was monitored by thin layer chromatography (TLC) using silica gel plates (silica gel 60 F254 0.25 mm), and components were visualized under UV light (254 and 365 nm). Column chromatography was performed on silica gel 200-300 mesh. Unless otherwise noted, all other compounds have been reported in the literature or are commercially available. Commercial reagents were used without further purification.

## Side-Products for the C-H Annulation Reaction of Benzimidazole-5-Carboxylic Acids with Iodonium Ylides

**Table S1. List of side-products**

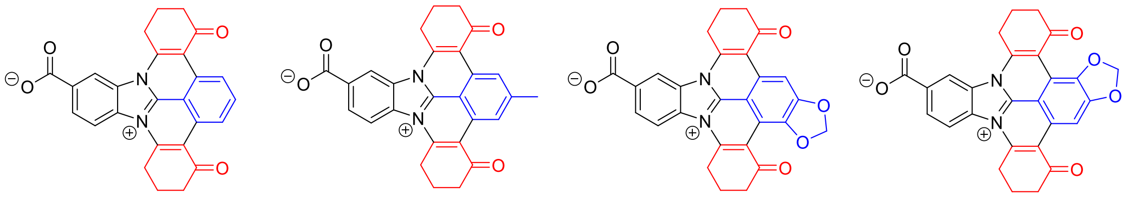

**6a**, 21%  **6c**, 14%  **6q/6q'**, 17%
**6q** : **6q'** = 1 : 0.82

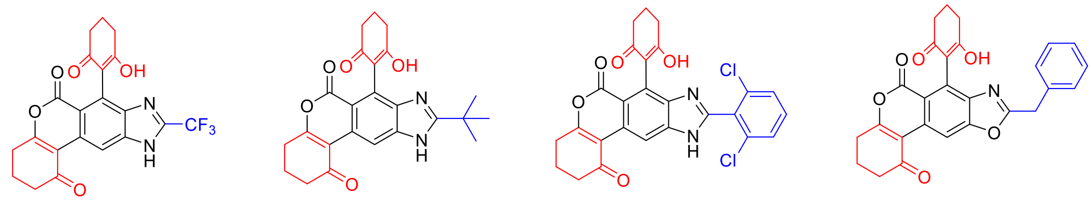

**7f**, 16%  **7g**, 12%  **7i**, 14%  **7x**, 25%

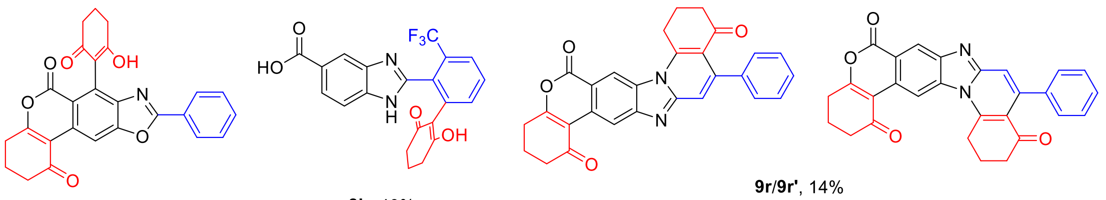

**7y**, 17%  **8h**, 40%  **9r/9r'**, 14%
**9r** : **9r'** = 1 : 0.31

## Procedures for the C-H Annulation Reaction of Benzimidazole-5-Carboxylic acids with Iodonium Ylides

### General procedures for the synthesis of 3 and 4

Benzimidazole-5-carboxylic acid **1** (0.1 mmol), [Cp*RhCl$_2$]$_2$ (1.5 mg, 0.005 mmol), AgOAc (4.2 mg, 0.025 mmol) and TFE (0.6 mL) were charged into a reaction tube. Then iodonium ylide **2** (0.12 mmol) was added. The tube was sealed and the reaction mixture was stirred at 90 °C for 6 h. The crude reaction mixture was diluted by DCM and MeOH to dissolve the precipitated products and vacuum filtration was performed to remove silver salts. The filtrate was concentrated under reduced pressure, then the crude mixture was purified by silica gel chromatography using EA/DCM to afford **3** and **4**.

**General procedures for the synthesis of 5/5'**

Benzimidazole-5-carboxylic acid **1** (0.1 mmol), [Cp*RhCl$_2$]$_2$ (1.5 mg, 0.005 mmol), AgOAc (4.2 mg, 0.025 mmol) and TFE (0.6 mL) were charged into a reaction tube. Then iodonium ylide **2** (0.12 mmol) was added. The tube was sealed and the reaction mixture was stirred at 90 °C for 6 h. The crude reaction mixture was concentrated under reduced pressure, then purified by silica gel chromatography using EA/MeOH to afford **5/5'** as an inseparable mixture.

## Procedures for the Preparation of the Substrates

**Procedures for methyl esterification of 3,4-diaminobenzoic acid**

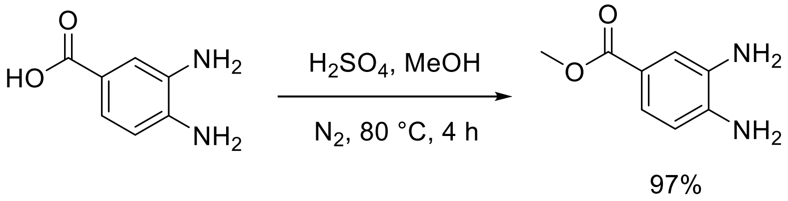


To a suspension of 3,4-diaminobenzoic acid (5 g, 13.2 mmol) in MeOH (150 mL) was slowly added concentrated $H_2SO_4$ (10 mL) under vigorous stirring. The reaction mixture was refluxed under nitrogen atmosphere at 80 °C for 4 h. After removing excess MeOH under reduced pressure, the reaction mixture was carefully basified by aqueous $Na_2CO_3$ and then extracted with EtOAc. The organic phase was dried with $MgSO_4$ and the solvent was removed under reduced pressure to afford methyl 3,4-diaminobenzoate (5.3 g, 97% yield) as a tan solid.

**General procedures for the synthesis of methyl 2-alkylbenzimidazole-5-carboxylate (1a*-1g*, 1p*-1q*, 1ar*)**

A mixture of methyl 3,4-diaminobenzoate (166 mg, 1 mmol) and alkyl carboxylic acid (10 mmol) was stirred under nitrogen atmosphere at reaction temperature. After completion of reaction, the mixture was diluted by EtOAc, basified by aqueous $Na_2CO_3$ and then extracted with EtOAc. The organic phase was dried with $MgSO_4$ and the solvent was removed under reduced pressure, then purified by silica gel

chromatography using EA/Hexane to afford 2-alkylbenzimidazole-5-carboxylate **1a*-1g***, **1p*-1q***, **1ar***.

**Table S2. Substrate list of 2-alkylbenzimidazole-5-carboxylates.**

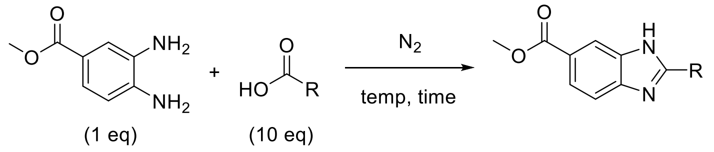


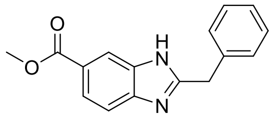

**1a*** (120 °C, 4 h, 91%)[a]
EA / Hex = 2 : 3

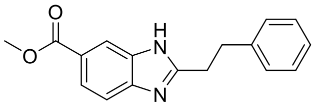

**1b*** (120 °C, 5 h, 92%)[a]
EA / Hex = 2 : 3

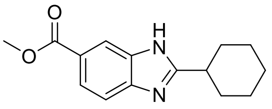

**1c*** (140 °C, 10 h, 84%)
EA / Hex = 1 : 2

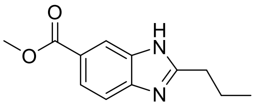

**1d*** (120 °C, 7 h, 92%)
EA / Hex = 3 : 2

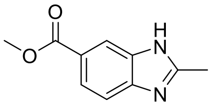

**1e*** (120 °C, 4 h, 98%)[b]
EA / Hex = 3 : 1

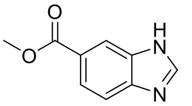

**1q*** (100 °C, 2 h, 95%)[b]
EA

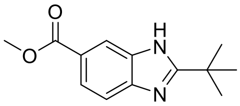

**1g*** (170 °C, 48 h, 71%)
EA / Hex = 1 : 2

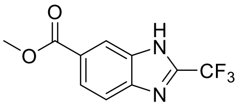

**1f*** (100 °C, 2 h, 94%)[b,c]
EA / Hex = 1 : 3

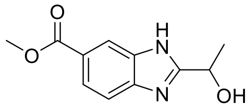

**1p*** (100 °C, 2 h, 81%)
EA / Hex = 3 : 1

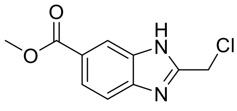

**1ar*** (100 °C, 3 h, 64%)
EA / Hex = 2 : 3

[a] 5 eq. of carboxylic acid was used. [b] Column chromatography was not required. [c] The reaction was performed in a sealed tube.

**General procedures for the synthesis of methyl 2-arylbenzimidazole-5-carboxylate (1h*-1i*, 1s*-1t*, 1z*-1ae*, 1ag*-1ap*)[1]**

To a mixture of methyl 3,4-diaminobenzoate (166 mg, 1 mmol), aryl aldehyde (1.1 mmol), and NaF (13 mg, 0.3 mmol) was added DMF (3 mL). The reaction mixture was stirred vigorously under air at 130 ºC. After completion of reaction, as indicated by the disappearing of yellow imine intermediate, the mixture was diluted by water and then extracted with EtOAc. The organic phase was dried with $MgSO_4$ and the solvent was removed under reduced pressure, then purified by silica gel chromatography using EA/Hexane to afford 2-arylbenzimidazole-5-carboxylate **1h*-1i***, **1s*-1t***, **1z*-1ae***, **1ag*-1ap***.

**Table S3. Substrate list of 2-arylbenzimidazole-5-carboxylates.**

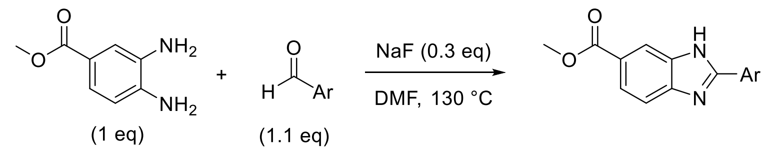

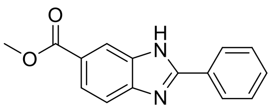

**1z*** (9 h, 88%)
EA / Hex = 1 : 2

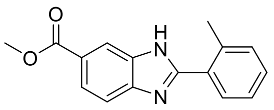

**1aa*** (11 h, 89%)
EA / Hex = 1 : 2

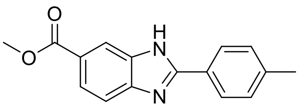

**1ab*** (16 h, 86%)
EA / Hex = 1 : 2

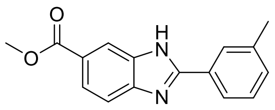

**1ac*** (22 h, 88%)
EA / Hex = 1 : 2

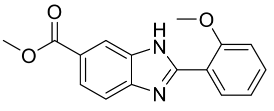

**1ad*** (60 h, 85%)
EA / Hex = 2 : 3

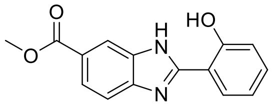

**1ae*** (19 h, 76%)
EA / Hex = 2 : 5

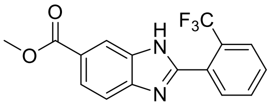

**1ag*** (36 h, 87%)
EA / Hex = 1 : 2

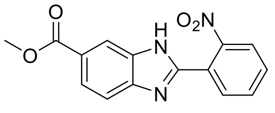

**1ah*** (24 h, 81%)
EA / Hex = 1 : 1

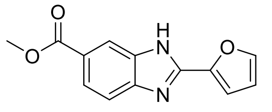

**1s*** (7 h, 79%)
EA / Hex = 1 : 1

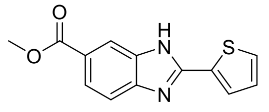

**1aj*** (22 h, 86%)
EA / Hex = 2 : 3

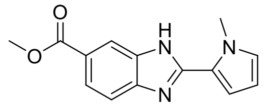

**1ak*** (45 h, 72%)
EA / Hex = 2 : 5

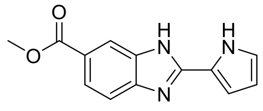

**1al*** (20 h, 78%)
EA / Hex = 1 : 2

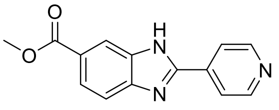

**1ai*** (10 h, 81%)
EA

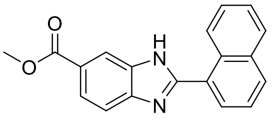

**1am*** (25 h, 88%)
EA / Hex = 1 : 2

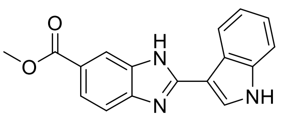

**1an*** (22 h, 85%)
EA / Hex = 3 : 1

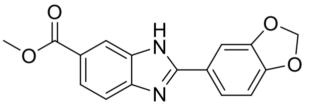

**1ao*** (18 h, 88%)
EA / Hex = 2 : 5

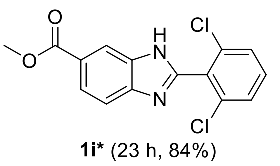

**1i*** (23 h, 84%)
EA / Hex = 1 : 2

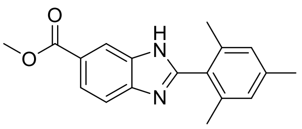

**1h*** (29 h, 80%)
EA / Hex = 2 : 5

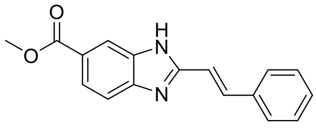

**1t*** (35 h, 62%)
EA / Hex = 1 : 2

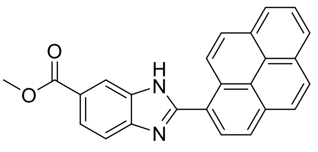

**1ap*** (30 h, 84%)
EA / Hex = 1 : 2

**Procedures for the synthesis of methyl 2-(2-aminophenyl)benzimidazole-5-carboxylate (1af*)**

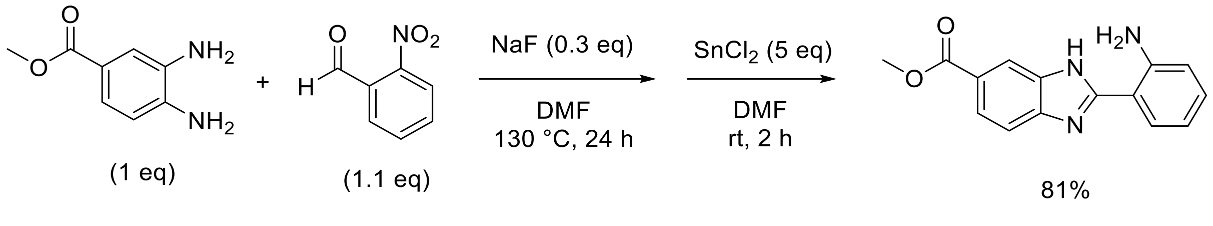

To a mixture of methyl 3,4-diaminobenzoate (166 mg, 1 mmol), 2-nitrobenzaldehyde (166 mg, 1.1 mmol), and NaF (13 mg, 0.3 mmol) was added DMF (3 mL). The reaction mixture was stirred vigorously under air at 130 ºC for 24 h. The reaction was cooled to room temperature, then $SnCl_2$ (950 mg, 5 mmol) was added into the reaction mixture, and kept stirring for another 2 h. After completion of reaction, the mixture was basified by aqueous $Na_2CO_3$ and then extracted with EtOAc. The organic phase was dried with $MgSO_4$ and the solvent was removed under reduced pressure, then purified by

silica gel chromatography using EA/Hexane (2:7) to afford methyl 2-(2-aminophenyl)benzimidazole-5-carboxylate **1af*** (216 mg, 81% yield).

**Procedures for the synthesis of methyl 2-benzoylbenzimidazole-5-carboxylate (1o*)[2]**

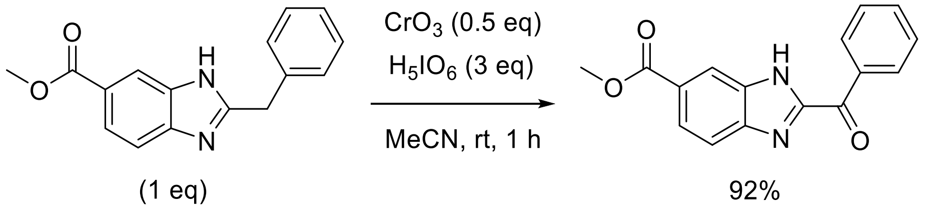


$H_5IO_6$ (684 mg, 3 mmol) was dissolved in MeCN (8 mL), then $CrO_3$ (50 mg, 5 mmol) was dissolved into the solution. Methyl 2-benzylbenzimidazole-5-carboxylate **1a*** was added and stirred at room temperature for 1 h, the mixture was diluted by water and basified by aqueous $Na_2CO_3$, then extracted with EtOAc. The organic phase was dried with $MgSO_4$ and the solvent was removed under reduced pressure to afford methyl 2-benzoylbenzimidazole-5-carboxylate **1o*** (258 mg, 92% yield) as white solid.

**Procedures for the synthesis of methyl 2-aminobenzimidazole-5-carboxylate (1r*)[3]**

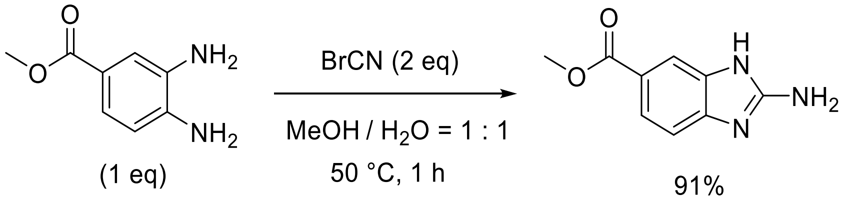


To a solution of methyl 3,4-diaminobenzoate (166 mg, 1 mmol) in 50% aqueous MeOH (3 mL), BrCN (212 mg, 2 mmol) was added and stirred at 50 °C for 1 h. The reaction mixture was basified by aqueous $Na_2CO_3$ and then extracted with EtOAc. The organic phase was dried with $MgSO_4$ and the solvent was removed under reduced pressure to afford methyl 2-aminobenzimidazole-5-carboxylate **1r*** (174 mg, 91% yield) as pale orange solid.

**Procedures for the synthesis of methyl 2-ethoxybenzimidazole-5-carboxylate (1n*)[4]**

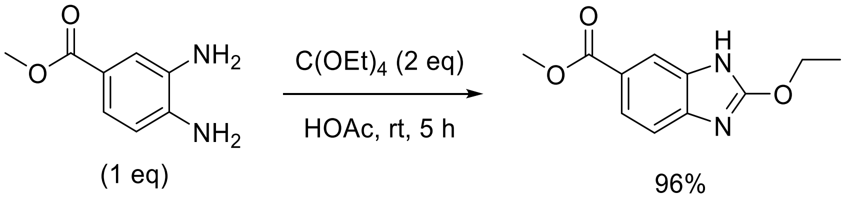


To a solution of methyl 3,4-diaminobenzoate (166 mg, 1 mmol) in acetic acid (3 mL), tetraethyl

orthocarbonate (384 mg, 2 mmol) was added and stirred at room temperature for 5 h. The reaction mixture was basified by aqueous $Na_2CO_3$ and then extracted with EtOAc. The organic phase was dried with $MgSO_4$ and the solvent was removed under reduced pressure to afford methyl 2-ethoxybenzimidazole-5-carboxylate **1n*** (212 mg, 96% yield) as pale orange solid.

**Procedures for the synthesis of methyl thiobenzimidazolinone-5-carboxylate (1at*)[5]**

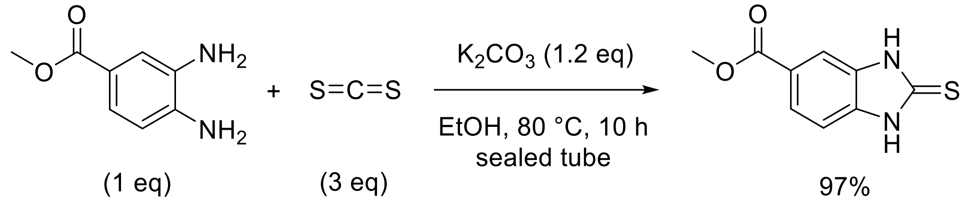


A solution of methyl 3,4-diaminobenzoate (166 mg, 1 mmol) in EtOH (2 mL) was charged into reaction tube, followed by addition of $K_2CO_3$ (166 mg, 1.2 mmol) and $CS_2$ (228 mg, 3 mmol). The tube was sealed and stirred at 80 ºC for 10 h. The reaction mixture was diluted by water and then extracted with EtOAc. The organic phase was dried with $MgSO_4$ and the solvent was removed under reduced pressure to afford methyl thiobenzimidazolinone-5-carboxylate **1at*** (203 mg, 97% yield) as pale orange powder.

**Procedures for the synthesis of methyl 2-(methylthio)benzimidazole-5-carboxylate (1l*)**

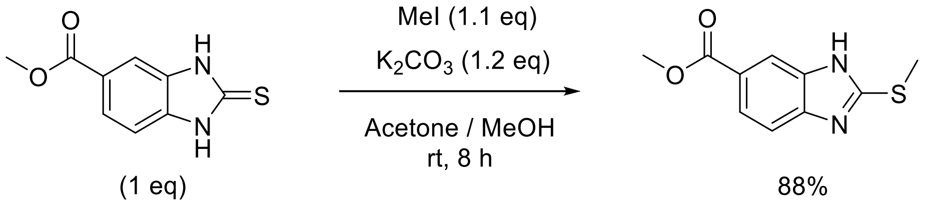


Methyl thiobenzimidazolinone-5-carboxylate **1at*** (208 mg, 1 mmol) was dissolved in acetone/MeOH (5:1), then $K_2CO_3$ (166 mg, 1.2 mmol) was added, followed by slow addition of MeI (156 mg, 1.1 mmol) under strong stirring. After 8 h the reaction mixture was diluted by water and then extracted with EtOAc. The organic phase was dried with $MgSO_4$ and the solvent was removed under reduced pressure, then purified by silica gel chromatography using EA/Hexane (1:3) to afford methyl 2-(methylthio)benzimidazole-5-carboxylate **1l*** (188 mg, 88% yield) as colorless oil.

**Procedures for the synthesis of methyl 2-(phenylthio)benzimidazole-5-carboxylate (1m*)[6]**

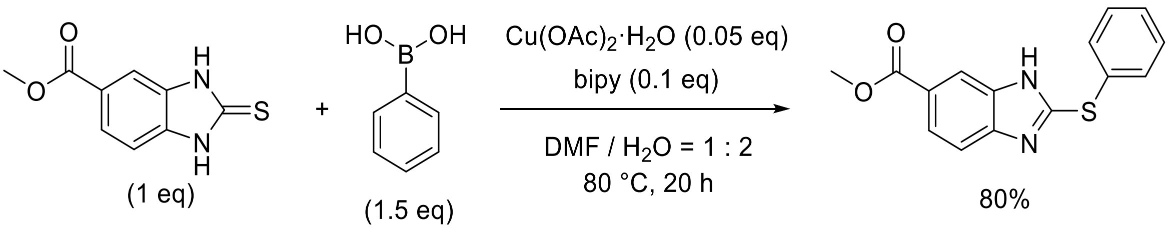

To a mixture of methyl thiobenzimidazolinone-5-carboxylate **1at*** (208 mg, 1 mmol), phenylboronic acid (183 mg, 1.5 mmol), $Cu(OAc)_2{\cdot}H_2O$ (10 mg, 0.05 mmol), and 2,2'-bipyridine (16 mg, 0.1 mmol) was added DMF (2 mL) and water (4 mL). The reaction mixture was stirred under air at 80 ºC for 20 h, then basified by aqueous $Na_2CO_3$ and extracted with EtOAc. The organic phase was dried with $MgSO_4$ and the solvent was removed under reduced pressure, then purified by silica gel chromatography using EA/Hexane (1:4) to afford methyl 2-(phenylthio)benzimidazole-5-carboxylate **1m*** (226 mg, 80% yield) as colorless oil.

**Procedures for the synthesis of methyl 2-bromobenzimidazole-5-carboxylate (1k*)**[7]

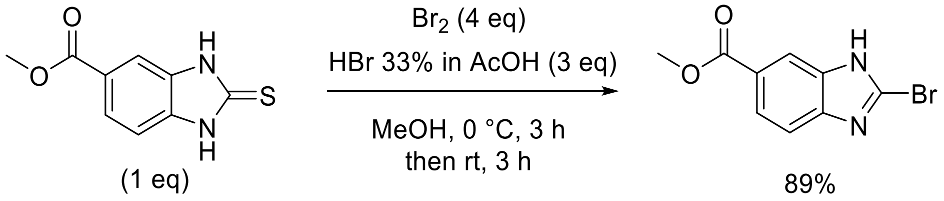

To an ice cooled suspension of methyl thiobenzimidazolinone-5-carboxylate **1at*** (208 mg, 1 mmol) in MeOH (8 mL) was added 33% HBr solution in AcOH (730 mg, 3 mmol). Under strong stirring, $Br_2$ (640 mg, 4 mmol) was slowly added into the mixture, the reaction was kept in ice bath for 3 h, followed by warming to room temperature for another 3 h. After precipitate had developed, the reaction mixture was basified by aqueous $Na_2CO_3$ and extracted with EtOAc. The organic phase was dried with $MgSO_4$ and the solvent was removed under reduced pressure, then purified by silica gel chromatography using EA/Hexane (1:2) to afford methyl 2-bromobenzimidazole-5-carboxylate **1k*** (228 mg, 89% yield) as off-white solid.

**Procedures for the synthesis of methyl 2-chlorobenzimidazole-5-carboxylate (1j*)**

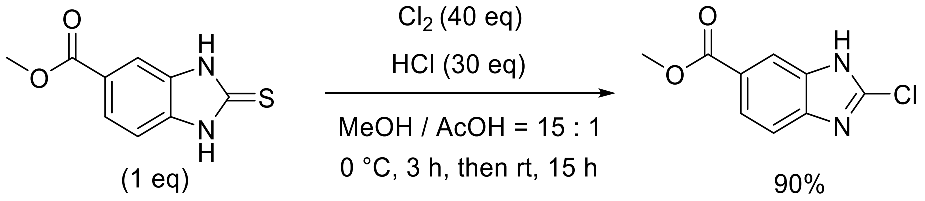

To an ice cooled suspension of methyl thiobenzimidazolinone-5-carboxylate **1at*** (208 mg, 1 mmol) in

MeOH (8 mL) was added AcOH (0.5 mL). Cooling under ice bath, gaseous HCl (40 mmol) and then $Cl_2$ (30 mmol) was sparged into the reaction mixture, which was kept stirring in ice bath for 3 h, then then bring to room temperature for another 15 h. After completion, the reaction mixture was basified by aqueous $Na_2CO_3$ and extracted with EtOAc. The organic phase was dried with $MgSO_4$ and the solvent was removed under reduced pressure, then purified by silica gel chromatography using EA/Hexane (1:2) to afford methyl 2-chlorobenzimidazole-5-carboxylate **1j*** (189 mg, 90% yield) as white solid.

**Procedures for the synthesis of ((5-(methoxycarbonyl)benzimidazol-2-yl)methyl)triphenylphosphonium chloride (1at*)[8]**

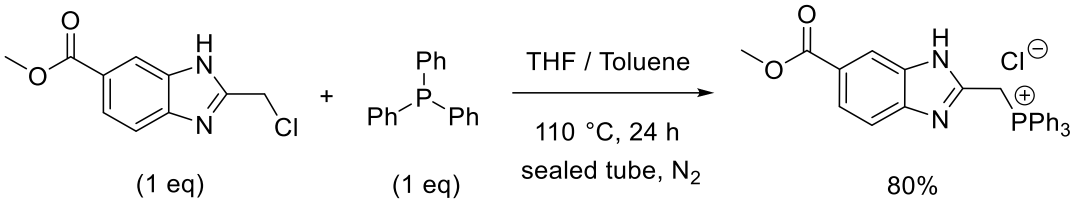


A solution of methyl 2-(chloromethyl)benzimidazole-5-carboxylate **1ar*** (225 mg, 1 mmol) in THF (1.5 mL) and a solution of $PPh_3$ (262 mg, 1 mmol) in toluene (1.5 mL) was charged into a reaction tube, it was then sealed and stirred under nitrogen atmosphere at 110 °C for 24 h. After the reaction had cooled to room temperature, the precipitate was collected by vacuum filtration, washed by toluene and EtOAc, then dried to afford phosphonium salt **1as*** (390 mg, 80% yield) as white powder.

**Procedures for the synthesis of methyl 2-vinylbenzimidazole-5-carboxylate (1w*)**

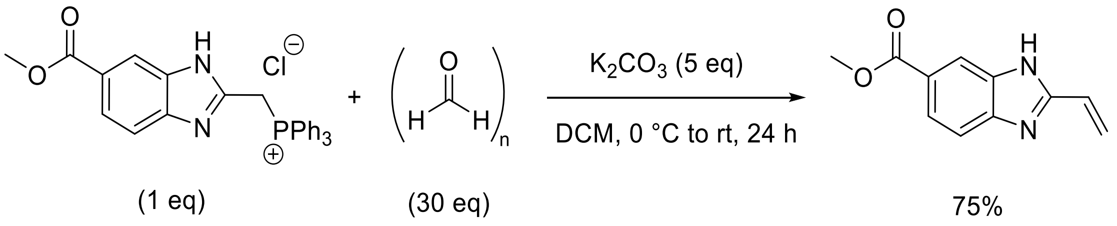


To an ice cooled solution of phosphonium salt **1as*** (487 mg, 1 mmol) in DCM (6 mL) was added $K_2CO_3$ (690 mg, 5 mmol). After stirring for 5 min, paraformaldehyde (900 mg, 30 mmol) was added, and kept stirring at room temperature for 24 h. The reaction mixture was filtered and the filtrate was concentrated under reduced pressure, then purified by silica gel chromatography using EA/Hexane (1:1) to afford methyl 2-vinylbenzimidazole-5-carboxylate **1w*** (152 mg, 75% yield) as white solid.

**Procedures for the synthesis of methyl 2-(prop-1-en-1-yl)benzimidazole-5-carboxylates (1u*/1v*)**

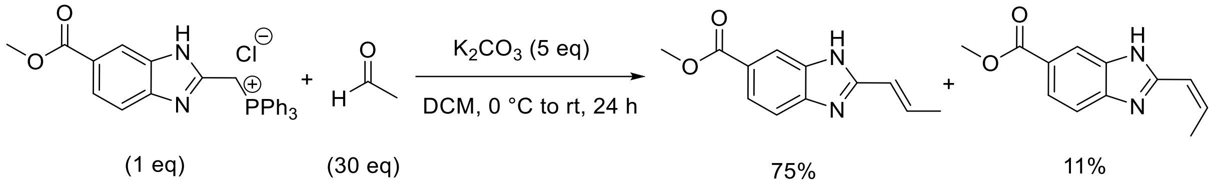


To a reaction tube, an ice cooled solution of phosphonium salt **1as*** (487 mg, 1 mmol) in DCM (6 mL) was added $K_2CO_3$ (690 mg, 5 mmol). After stirring for 5 min, acetaldehyde (1320 mg, 30 mmol) was added, the tube was sealed and kept stirring at room temperature for 24 h. The reaction mixture was quenched by water and then extracted with EtOAc. The organic phase was dried with $MgSO_4$ and the solvent was removed under reduced pressure, then purified by silica gel chromatography using EA/Hexane (2:3) to afford methyl (*E*)-2-(prop-1-en-1-yl)benzimidazole-5-carboxylate **1u*** (153 mg, 75% yield) and methyl (*Z*)-2-(prop-1-en-1-yl)benzimidazole-5-carboxylate **1v*** (22 mg, 11% yield) as white solids.

**General hydrolysis procedures to benzimidazole-5-carboxylic acid (1a-1w, 1z-1ap)**

To a solution of ester **1*** (1 mmol) in THF was added 5% aqueous NaOH (3 mmol). The reaction mixture was stirred under nitrogen at 60 ºC. After completion of reaction, THF was removed under reduced pressure. Then stoichiometric amount of dilute HCl was added dropwise to the ice cooled solution of carboxylate salt under vigorous stirring. The precipitated carboxylic acid was collected by vacuum filtration, washed with ice cold water then dried to afford benzimidazole-5-carboxylic acid **1a-1w**, **1z-1ap** as powder.

**Table S4. Substrate list of benzimidazole-5-carboxylic acids.**

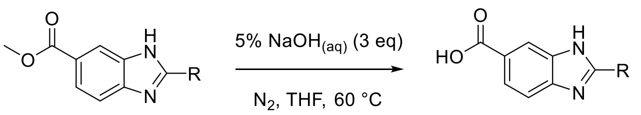


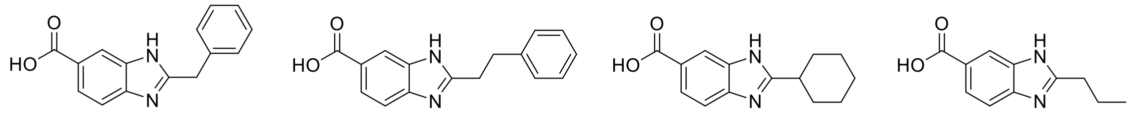

**1a** (16 h, 87%) **1b** (12 h, 95%) **1c** (15 h, 89%) **1d** (12 h, 96%)*

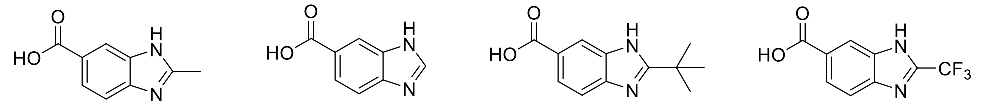

**1e** (12 h, 65%) **1q** (18 h, 90%) **1g** (12 h, 74%) **1f** (12 h, 93%)

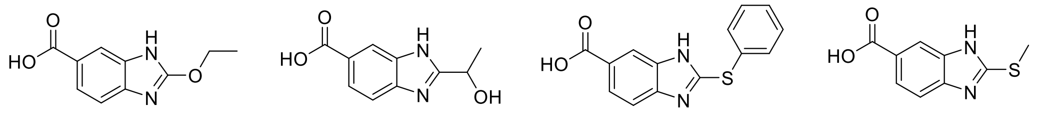

**1n** (16 h, 94%) **1p** (9 h, 96%)* **1m** (11 h, 87%) **1l** (12 h, 88%)

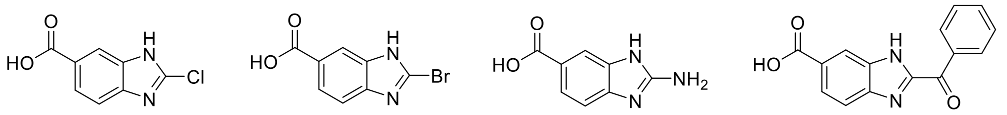

**1j** (12 h, 94%) **1k** (12 h, 91%) **1r** (15 h, 89%) **1o** (12 h, 91%)

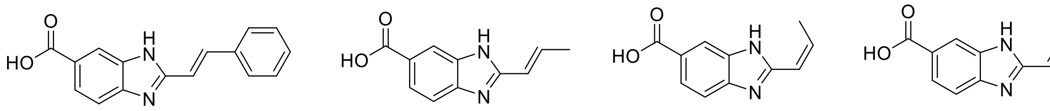

**1t** (12 h, 82%) **1u** (17 h, 86%) **1v** (20 h, 85%) **1w** (12 h, 78%)

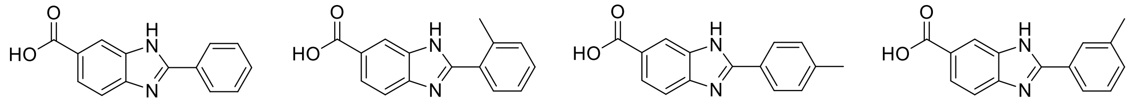

**1z** (16 h, 91%) **1aa** (14 h, 81%) **1ab** (15 h, 92%) **1ac** (18 h, 93%)

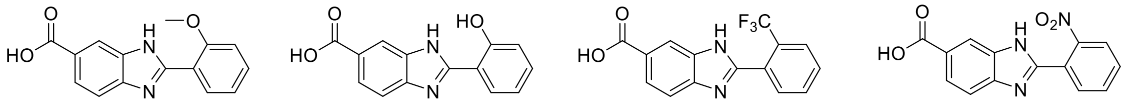

**1ad** (27 h, 94%)* **1ae** (15 h, 96%) **1ag** (24 h, 94%) **1ah** (51 h, 84%)

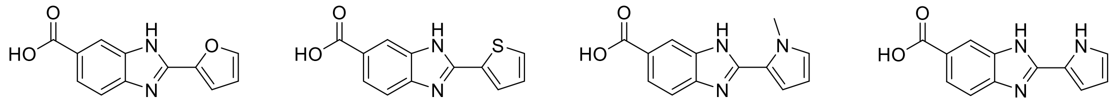

**1s** (12 h, 90%) **1aj** (14 h, 96%) **1ak** (15 h, 98%) **1al** (14 h, 65%)

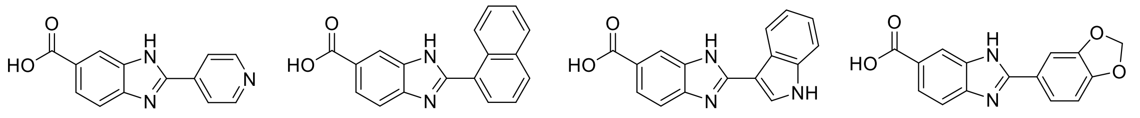

**1ai** (24 h, 95%) **1am** (24 h, 90%) **1an** (135 h, 87%) **1ao** (20 h, 91%)

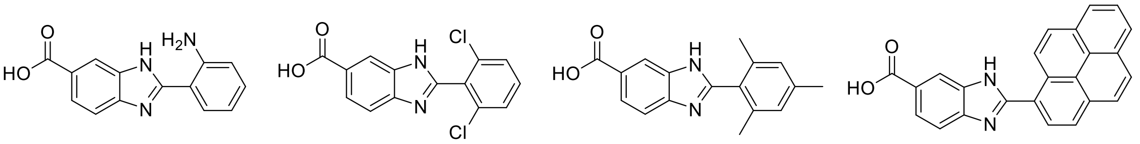

**1af** (18 h, 91%) **1i** (15 h, 93%) **1h** (14 h, 91%) **1ap** (70 h, 98%)

*The solution was extracted by EA and the combined organic phase was concentrated to afford **1** as powder.

**Procedures for methyl esterification of 3-amino-4-hydroxybenzoic acid**

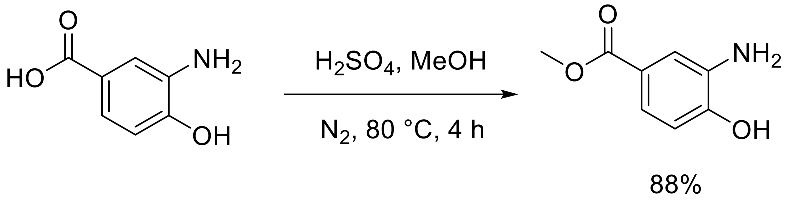


To a solution of 3-amino-4-hydroxybenzoic acid (1 g, 6.5 mmol) in MeOH (30 mL) was slowly added concentrated $H_2SO_4$ (2 mL) under vigorous stirring. The reaction mixture was refluxed under nitrogen atmosphere at 80 ºC for 4 h. After removing excess MeOH under reduced pressure, the reaction mixture was carefully basified by aqueous $NaHCO_3$ and then extracted with EtOAc. The organic phase was dried with $MgSO_4$ and the solvent was removed under reduced pressure to afford methyl 3-amino-4-hydroxybenzoate (0.96 g, 88% yield) as a tan solid.

**Procedures for the synthesis of methyl 2-benzylbenzoxazole-5-carboxylate (1x*)**

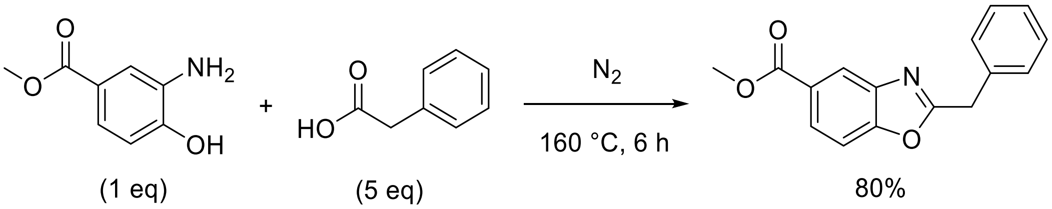


A mixture of methyl 3-amino-4-hydroxybenzoate (167 mg, 1 mmol) and phenylacetic acid (690 mg, 5 mmol) was stirred under nitrogen atmosphere at 160 ºC for 6 h. After completion of reaction, the mixture was diluted by EtOAc, basified by aqueous $Na_2CO_3$ and then extracted with EtOAc. The organic phase was dried with $MgSO_4$ and the solvent was removed under reduced pressure, then purified by silica gel chromatography using EA/Hexane (1:10) to afford methyl 2-benzylbenzoxazole-5-carboxylate **1x*** (213 mg, 80% yield) as a white solid.

**Procedures for the synthesis of methyl 2-phenylbenzoxazole-5-carboxylate (1y*)** [9]

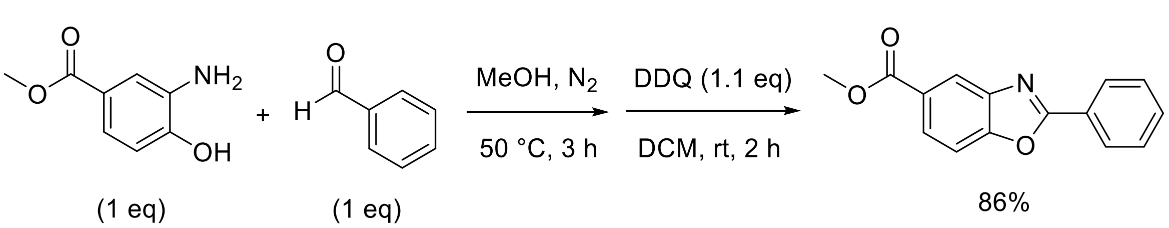


To a solution of methyl 3-amino-4-hydroxybenzoate (167 mg, 1 mmol) in MeOH (5 mL) was added benzaldehyde (106 mg, 1 mmol). The reaction mixture was stirred under nitrogen atmosphere at 50 ºC

for 3 h. After removing MeOH under reduced pressure, the crude imine was dissolved by DCM (5 mL), then DDQ (250 mg, 1.1 mmol) was added. After stirring at room temperature for 2 h, the reaction mixture was basified by saturated aqueous $NaHCO_3$ and then extracted with EtOAc. The organic phase was dried with $MgSO_4$ and the solvent was removed under reduced pressure, then purified by silica gel chromatography using EA/Hexane (1:10) to afford methyl 2-phenylbenzoxazole-5-carboxylate **1y*** (211 mg, 86% yield) as an off-white solid.

**General hydrolysis procedures to benzoxazole-5-carboxylic acid (1x-1y)**

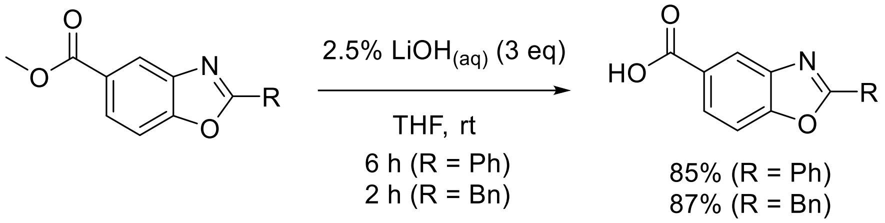


To a solution of ester **1x*** or **1y*** (1 mmol) in THF was added 2.5% aqueous LiOH (3 mmol). The reaction mixture was stirred at room temperature. After completion of reaction, THF was removed under reduced pressure. Then stoichiometric amount of dilute HCl was added dropwise to the ice cooled solution of carboxylate salt under vigorous stirring. The precipitated carboxylic acid was collected by vacuum filtration, washed with ice cold water then dried to afford benzoxazole-5-carboxylic acid **1x** or **1y** as white powder.

**General procedures for the synthesis of iodonium ylide (2)[10]**

To an ice cooled solution of 1,3-diketone (1 mmol) in MeOH (2 mL) was added a solution of KOH (140 mg, 2.5 mmol) in MeOH (1.5 mL), followed by a solution of (diacetoxyiodo)benzene (345 mg, 1.07 mmol) in MeOH (3 mL). The reaction was kept stirred in ice bath for 2 h, then excess MeOH was removed under reduced pressure, the crude mixture was diluted by water and extracted with DCM (30 mL x 5). The combined organic phase was dried with $MgSO_4$ and the solvent was removed under reduced pressure to afford iodonium ylide **2** as white to pale yellow solid.

**Table S5. Substrate list of iodonium ylides.**

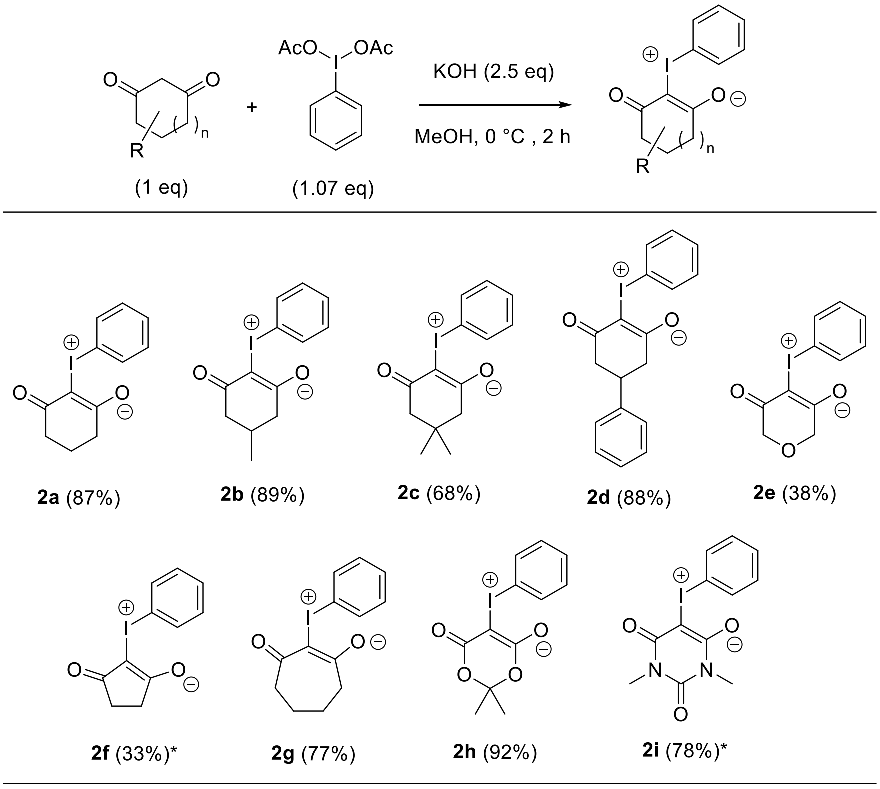


*Purified by washing the crude precipitate with water and MeOH.

### Procedures for the amidation of 2-benzylbenzimidazole-5-carboxylic acid

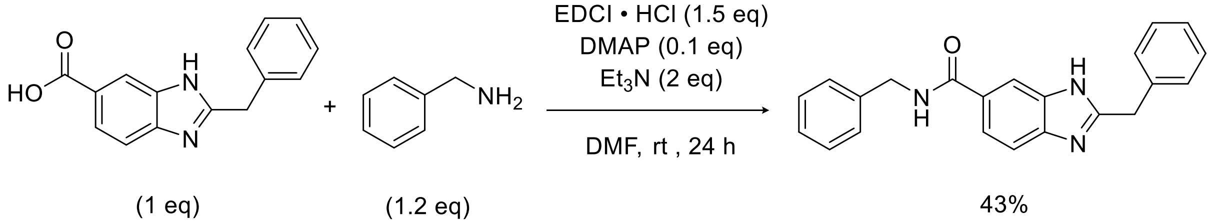


2-benzylbenzimidazole-5-carboxylic acid **1a** (252 mg, 1 mmol), EDCI • HCl (288 mg, 1.5 mmol), and DMAP (12 mg, 0.1 mmol) was dissolved in DMF (4 mL), then $Et_3N$ (202 mg, 2 mmol) and benzylamine (128 mg, 1.2 mmol) was added to the solution. After stirring at room temperature for 24 h, the reaction mixture was diluted by water and extracted with EtOAc. The organic phase was dried with $MgSO_4$ and the solvent was removed under reduced pressure, then purified by silica gel chromatography using EA/DCM (2:1) to afford *N*,2-dibenzylbenzimidazole-5-carboxamide **17** (147 mg, 43% yield) as white solid.

## Procedures for Synthetic Transformations

### Procedures for the benzylic oxidation of 3a and 4a to ketone derivatives 3o and 4o[11]

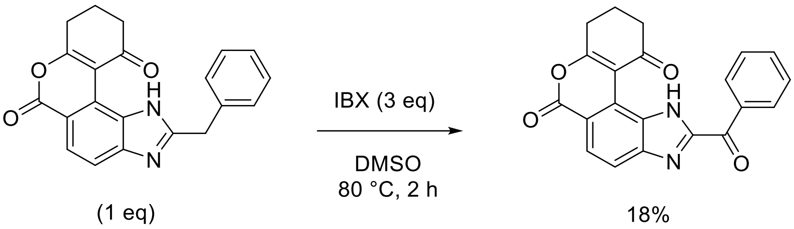


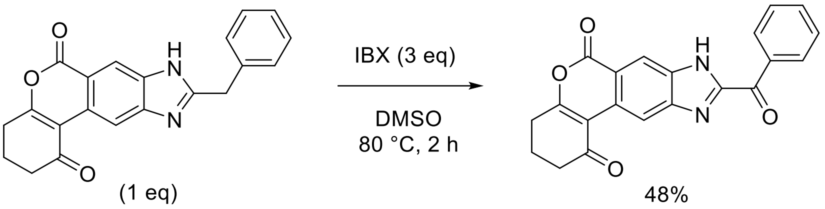


To a mixture of compound **3a** or **4a** (34.4 mg, 0.1 mmol) and DMSO (0.6 mL) was added IBX (84.0 mg, 0.3 mmol). After stirred at 80 ºC for 2 h, the crude mixture was basified by aqueous $Na_2CO_3$ and extracted with EtOAc. The organic phase was dried with $MgSO_4$ and the solvent was removed under reduced pressure, then purified by silica gel chromatography using EA/DCM (1:12 for **3o**; 1:6 for **4o**) to afford the ketone derivative **3o** (6.3 mg, 18% yield) or **4o** (17.2 mg, 48% yield) as off-white solid.

**Procedures for the amidation of 3a to tryptamine lactam derivative 10**

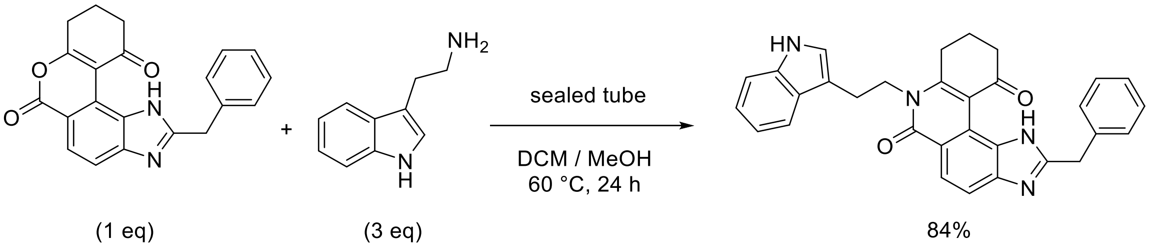


Compound **3a** (34.4 mg, 0.1 mmol) was charged into reaction tube and dissolved by DCM (1.6 mL), then tryptamine (48.0 mg, 0.3 mmol) and MeOH (0.4 mL) was added. The reaction tube was sealed and stirred at 60 ºC for 24 h, the crude mixture was concentrated under reduced pressure, then purified by silica gel chromatography using EA/DCM (1:2) to afford tryptamine lactam **10** (40.7 mg, 84% yield) as colorless gummy solid.

**Procedures for the reduction of 3a to alcohol derivative 13**

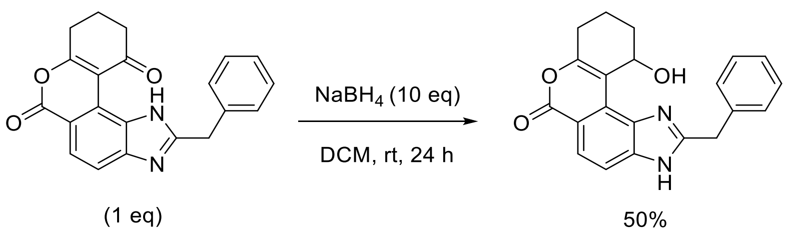

Compound **3a** (34.4 mg, 0.1 mmol) was dissolved in DCM (2 mL), then $NaBH_4$ (38.0 mg, 1 mmol) was added. After stirred at room temperature for 24 h, the crude mixture was diluted by water and extracted with EtOAc. The organic phase was dried with $MgSO_4$ and the solvent was removed under reduced pressure, then purified by silica gel chromatography using EA/DCM (1:3) to afford the alcohol derivative **13** (17.4 mg, 50% yield) as colorless gummy solid.

**Procedures for the deoxygenation of 3a to derivative 14, 15 and 16[12]**

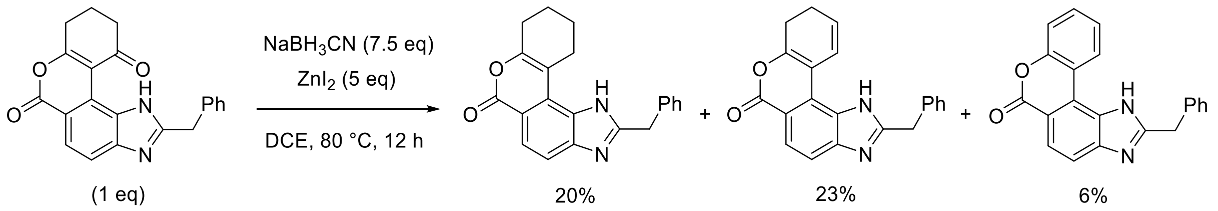


Compound **3a** (34.4 mg, 0.1 mmol) was dissolved in DCE (2 mL), then $NaBH_3CN$ (47.2 mg, 0.75 mmol) and $ZnI_2$ (160 mg, 0.5 mmol) was added. After stirred at 80 ºC for 12 h, the crude mixture was basified by aqueous $Na_2CO_3$ and extracted with EtOAc. The organic phase was dried with $MgSO_4$ and the solvent was removed under reduced pressure, then purified by silica gel chromatography using EA/DCM (1:6) to afford the deoxygenated derivative **14** (6.8 mg, 20% yield), **15** (7.5 mg, 23% yield), and **16** (2.0 mg, 6% yield) as colorless gummy solid.

**Procedures for the one-pot synthesis of benzylamine lactam derivative 11 and 12**

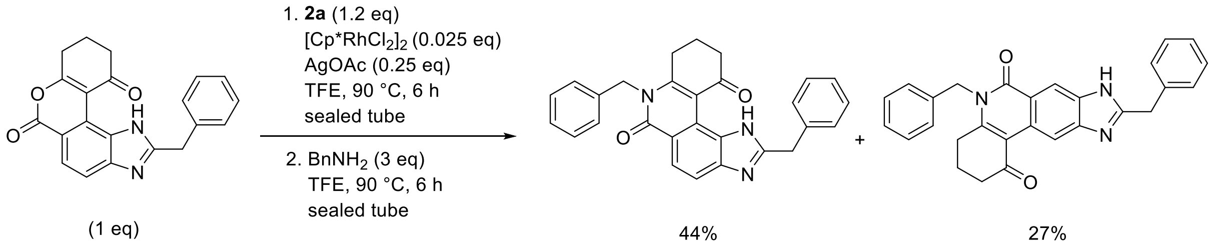


2-benzylbenzimidazole-5-carboxylic acid **1a** (0.1 mmol), $[Cp^*RhCl_2]_2$ (1.5 mg, 0.005 mmol), AgOAc (4.2 mg, 0.025 mmol) and TFE (0.6 mL) were charged into a reaction tube. Then iodonium ylide **2a** (0.12 mmol) was added. The tube was sealed and the reaction mixture was stirred at 90 ºC for 6 h. Benzylamine (32.1 mg, 0.3 mmol) was added and react under seal at 90 ºC for another 12 h. The crude reaction mixture was diluted by DCM and MeOH and filtered under vacuum. The filtrate was concentrated under reduced pressure, then the crude mixture was purified by silica gel chromatography using EA/DCM (1:3 ~ 1:1) to afford **11** (19.1 mg, 44% yield) and **12** (11.8 mg, 27% yield) as colorless gummy solid.

## Control Experiments

### General procedures for deuterium exchange experiments

Benzimidazole-5-carboxylic acid (0.1 mmol), $[Cp^{*}RhCl_2]_2$ (1.5 mg, 0.005 mmol), AgOAc (4.2 mg, 0.025 mmol) and $CD_3OD$ (0.6 mL) were charged into a reaction tube. The tube was sealed and the reaction mixture was stirred at 90 ºC for 6 h. The crude reaction mixture was then checked with NMR to calculate the percentage of deuterium being exchanged.

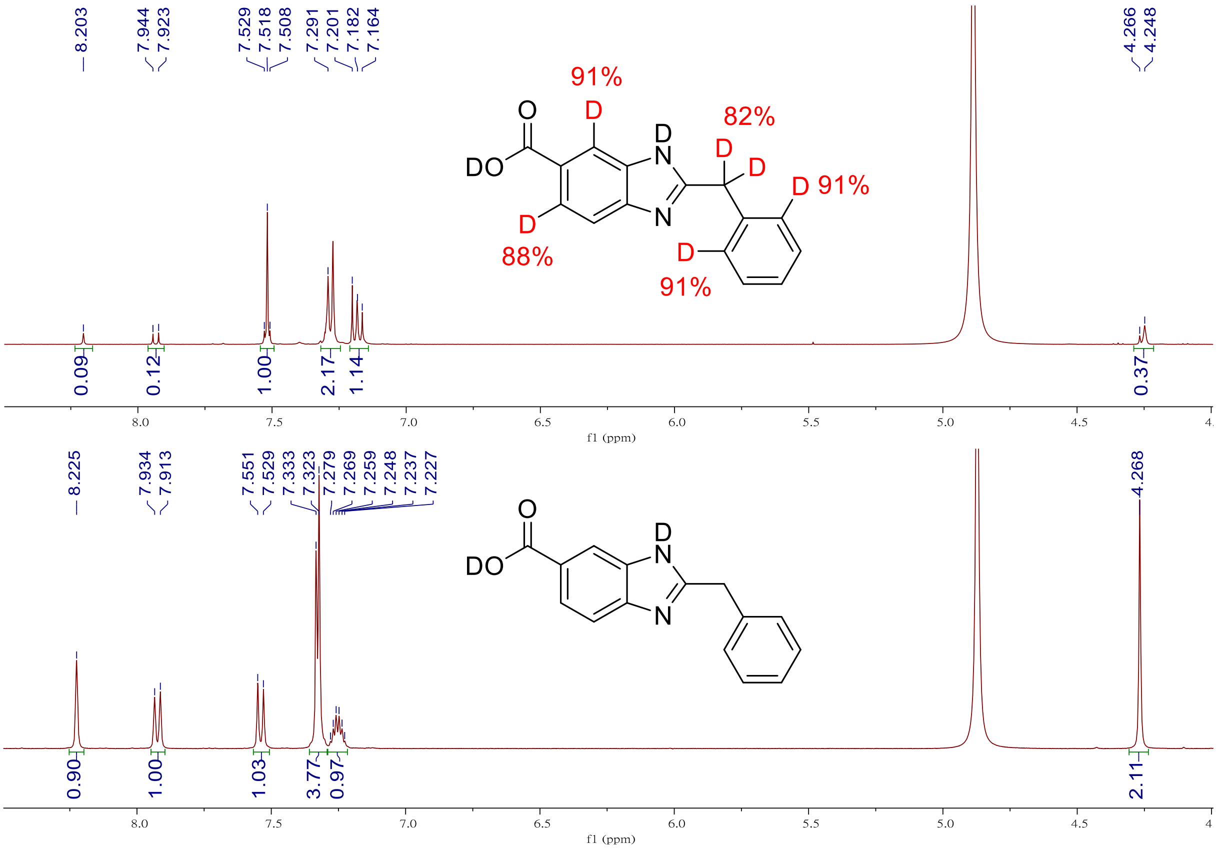


**Figure S1.** ¹H NMR spectrum for deuterium exchange experiment of **1a** (101 MHz, $CD_3OD$).

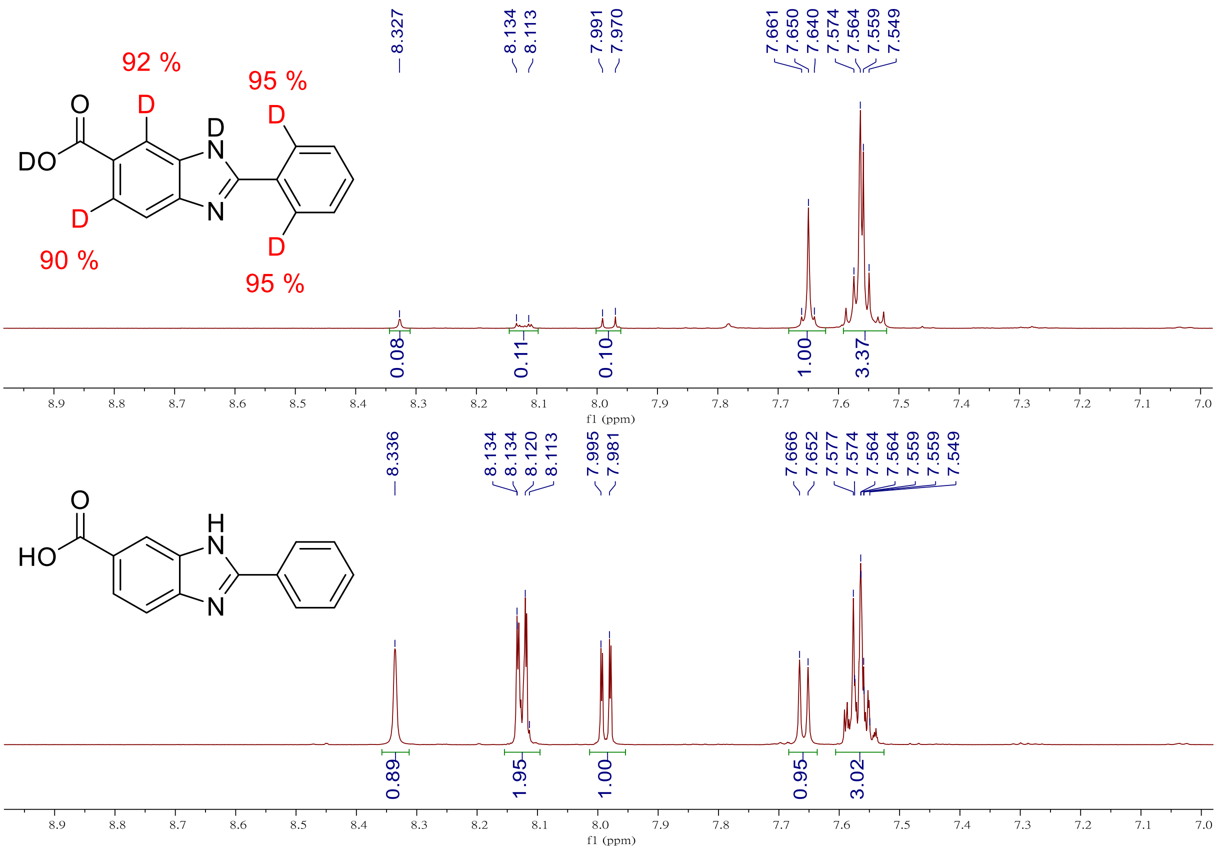


**Figure S2.** $^{1}$H NMR spectrum for deuterium exchange experiment of **1z** (101 MHz, $CD_3OD$).

## Characterization Data

### 2-benzylbenzimidazole-5-carboxylic acid (1a)

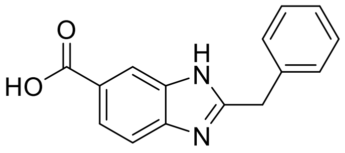

White solid, yield: 1159 mg, 87%; $^{1}$H NMR (400 MHz, $CD_3OD$): δ 8.23 (d, $J$ = 1.0 Hz, 1H), 7.92 (dd, $J$ = 8.5, 1.6 Hz, 1H), 7.54 (d, $J$ = 8.5 Hz, 1H), 7.33 – 7.32 (m, 4H), 7.28 – 7.23 (m, 1H), 4.27 (s, 2H); $^{13}$C{$^{1}$H} NMR (101 MHz, $CD_3OD$): δ 170.5, 157.9, 142.7, 139.5, 137.7, 129.9, 129.8, 128.1, 126.2, 125.2, 118.2, 114.9, 36.1; HRMS (ESI, m/z) calculated for $C_{15}H_{13}N_2O_2$ $(M + H)^+$ 253.0972, found 253.0977.

### 2-phenethylbenzimidazole-5-carboxylic acid (1b)

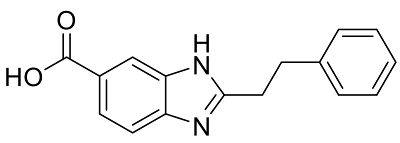

Beige solid, yield: 229 mg, 95%; $^{1}$H NMR (600 MHz, $CD_3OD$): δ 8.21 (d, $J$ = 0.8 Hz, 1H), 7.93 (dd, $J$ = 8.5, 1.5 Hz, 1H), 7.54 (d, $J$ = 8.5 Hz, 1H), 7.24 (t, $J$ = 7.4 Hz, 2H), 7.20 (d, $J$ = 7.0 Hz, 2H), 7.17 (t, $J$ = 7.2 Hz, 1H), 3.24 – 3.21 (m, 2H), 3.18 – 3.15 (m, 2H); $^{13}$C{$^{1}$H} NMR (101 MHz, $CD_3OD$): δ 170.5, 158.6, 142.5, 141.6, 139.3, 129.6, 129.3, 127.4, 126.1, 125.1, 118.0, 114.8, 35.2, 31.9; HRMS (ESI, m/z) calculated for $C_{16}H_{15}N_2O_2$ $(M + H)^+$ 267.1128, found 267.1130.

**2-cyclohexylbenzimidazole-5-carboxylic acid (1c)**

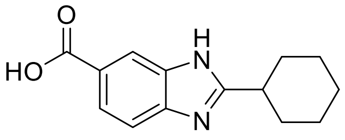

Off-white solid, yield: 178 mg, 89%; $^{1}$H NMR (600 MHz, $CD_3OD$): δ 8.23 (d, *J* = 0.9 Hz, 1H), 7.92 (dd, *J* = 8.4, 1.5 Hz, 1H), 7.54 (d, *J* = 8.5 Hz, 1H), 2.95 (tt, *J* = 11.9, 3.5 Hz, 1H), 2.12 – 2.09 (m, 2H), 1.92 – 1.89 (m, 2H), 1.81 – 1.78 (m, 1H), 1.69 (qd, *J* = 12.5, 3.3 Hz, 2H), 1.49 (qt, *J* = 12.9, 3.3 Hz, 2H), 1.37 (qt, *J* = 12.7, 3.5 Hz, 1H); $^{13}C\{^{1}H\}$ NMR (101 MHz, $CD_3OD$): δ 170.6, 163.3, 142.4, 139.1, 126.1, 125.1, 118.0, 114.8, 39.8, 32.6, 27.1, 26.9; HRMS (ESI, m/z) calculated for $C_{14}H_{17}N_2O_2$ $(M + H)^+$ 245.1285, found 245.1293.

**2-propylbenzimidazole-5-carboxylic acid (1d)**

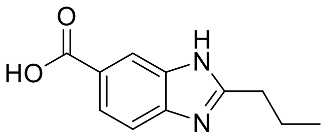

Beige solid, yield: 176 mg, 96%; $^{1}$H NMR (600 MHz, $CD_3OD$): δ 8.22 (dd, *J* = 1.4, 0.5 Hz, 1H), 7.92 (dd, *J* = 8.5, 1.5 Hz, 1H), 7.54 (dd, *J* = 8.5, 0.5 Hz, 1H), 2.90 (t, *J* = 7.5 Hz, 2H), 1.88 (sex, *J* = 7.5 Hz, 2H), 1.01 (t, *J* = 7.4 Hz, 3H); $^{13}C\{^{1}H\}$ NMR (101 MHz, $CD_3OD$): δ 170.6, 159.4, 142.5, 139.2, 126.2, 125.1, 117.9, 114.7, 31.7, 22.5, 14.0; HRMS (ESI, m/z) calculated for $C_{11}H_{13}N_2O_2$ $(M + H)^+$ 205.0972, found 205.0979.

**2-methylbenzimidazole-5-carboxylic acid (1e)**

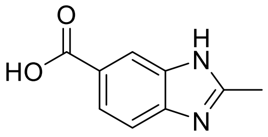

Off-white solid, yield: 186 mg, 65%; $^{1}$H NMR (400 MHz, $CD_3OD$): δ 8.20 (dd, *J* = 1.5, 0.6 Hz, 1H), 7.92 (dd, *J* = 8.5, 1.6 Hz, 1H), 7.53 (dd, *J* = 8.5, 0.6 Hz, 1H), 2.61 (s, 3H); $^{13}C\{^{1}H\}$ NMR (101 MHz, $CD_3OD$): δ 170.5, 155.7, 142.5, 139.3, 126.1, 125.1, 117.8, 114.6, 14.4; HRMS (ESI, m/z) calculated for $C_9H_9N_2O_2$ $(M + H)^+$ 177.0659, found 177.0661.

**2-(trifluoromethyl)benzimidazole-5-carboxylic acid (1f)**

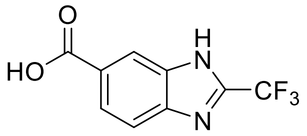

Beige solid, yield: 171 mg, 93%; $^{1}$H NMR (400 MHz, $CD_3OD$): δ 8.42 (s, 1H), 8.10 (dd, *J* = 8.5, 1.2 Hz, 1H), 7.75 (d, *J* = 7.7 Hz, 1H); $^{13}C\{^{1}H\}$ NMR (101 MHz, $CD_3OD$): δ 169.6, 144.3 (q, *J* = 41 Hz), 128.4, 126.9, 120.1 (q, *J* = 271 Hz); $^{19}$F NMR (376 MHz, $CD_3OD$) δ -66.1; HRMS (ESI, m/z) calculated for $C_9H_6F_3N_2O_2$ $(M + H)^+$ 231.0376, found 231.0375.

**2-(*tert*-butyl)benzimidazole-5-carboxylic acid (1g)**

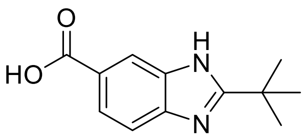


White solid, yield: 340 mg, 74%; $^1$H NMR (400 MHz, $CD_3OD$): δ 8.25 (dd, $J$ = 1.5, 0.6 Hz, 1H), 7.93 (dd, $J$ = 8.5, 1.6 Hz, 1H), 7.56 (dd, $J$ = 8.5, 0.6 Hz, 1H), 1.49 (s, 9H); $^{13}C\{^1H\}$ NMR (151 MHz, $CD_3OD$): δ 170.6, 166.6, 142.6 (br), 139.3 (br), 126.0, 125.0, 118.1, 114.8, 34.7, 29.5; HRMS (ESI, m/z) calculated for $C_{12}H_{15}N_2O_2$ $(M + H)^+$ 219.1129, found 219.1135.

**2-mesitylbenzimidazole-5-carboxylic acid (1h)**

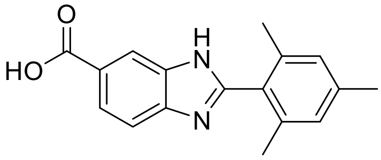


Off-white solid, yield: 201 mg, 91%; $^1$H NMR (600 MHz, $CD_3OD$): δ 8.34 (d, $J$ = 0.4 Hz, 1H), 8.01 (dd, $J$ = 8.5, 1.4 Hz, 1H), 7.66 (d, $J$ = 8.5 Hz, 1H), 7.02 (s, 2H), 2.35 (s, 3H), 2.12 (s, 6H); $^{13}C\{^1H\}$ NMR (101 MHz, $CD_3OD$): δ 170.4, 156.0, 142.5, 141.2, 139.5, 138.8, 129.3, 128.7, 126.4, 125.4, 118.6, 115.2, 21.3, 19.9; HRMS (ESI, m/z) calculated for $C_{17}H_{17}N_2O_2$ $(M + H)^+$ 281.1285, found 281.1286.

**2-(2,6-dichlorophenyl)benzimidazole-5-carboxylic acid (1i)**

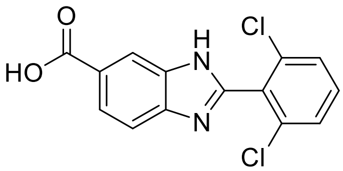


Off-white solid, yield: 168 mg, 93%; $^1$H NMR (600 MHz, $CD_3OD$): δ 8.38 (s, 1H), 8.04 (dd, $J$ = 8.5, 1.5 Hz, 1H), 7.70 (d, $J$ = 8.4 Hz, 1H), 7.60 – 7.59 (m, 2H), 7.57 – 7.54 (m, 1H); $^{13}C\{^1H\}$ NMR (101 MHz, $CD_3OD$): δ 170.2, 151.0, 137.1, 133.6, 130.9, 129.5, 126.9, 125.7; HRMS (ESI, m/z) calculated for $C_{14}H_9Cl_2N_2O_2$ $(M + H)^+$ 307.0036, found 307.0037.

**2-chlorobenzimidazole-5-carboxylic acid (1j)**

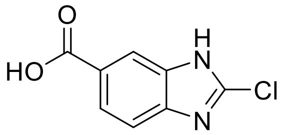


Beige solid, yield: 200 mg, 94%; $^1$H NMR (600 MHz, $CD_3OD$): δ 8.20 (dd, $J$ = 1.4, 0.5 Hz, 1H), 7.97 (dd, $J$ = 8.6, 1.6 Hz, 1H), 7.55 (dd, $J$ = 8.5, 0.4 Hz, 1H); $^{13}C\{^1H\}$ NMR (151 MHz, $CD_3OD$): δ 169.9, 143.0, 142.4, 139.4, 126.9, 125.8, 118.1, 115.0; HRMS (ESI, m/z) calculated for $C_8H_6ClN_2O_2$ $(M + H)^+$ 197.0112, found 197.0119.

**2-bromobenzimidazole-5-carboxylic acid (1k)**

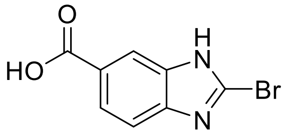


Beige solid, yield: 202 mg, 91%; $^1$H NMR (600 MHz, $CD_3OD$): δ 8.22 (dd, $J$ = 1.5, 0.6 Hz, 1H), 7.97 (dd, $J$ = 8.5, 1.5 Hz, 1H), 7.57 (dd, $J$ = 8.6, 0.6 Hz, 1H); $^{13}C\{^1H\}$ NMR (151 MHz, $CD_3OD$): δ 169.8, 143.1, 140.2, 130.6, 126.9, 125.8, 118.0, 114.8; HRMS (ESI, m/z) calculated for $C_8H_6BrN_2O_2$ $(M + H)^+$ 240.9608, found 240.9610.

**2-(methylthio)benzimidazole-5-carboxylic acid (1l)**

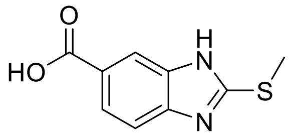

Off-white solid, yield: 199 mg, 88%; $^1$H NMR (400 MHz, $CD_3OD$): δ 8.16 (d, $J$ = 1.0 Hz, 1H), 7.90 (dd, $J$ = 8.5, 1.6 Hz, 1H), 7.49 (d, $J$ = 8.4 Hz, 1H), 2.76 (s, 3H); $^{13}C\{^1H\}$ NMR (151 MHz, $CD_3OD$): δ 170.4, 156.8, 143.8 (br), 140.4 (br), 125.6, 125.0, 116.9, 114.0, 14.3; HRMS (ESI, m/z) calculated for $C_9H_9N_2O_2S$ $(M + H)^+$ 209.0379, found 209.0384.

**2-(phenylthio)benzimidazole-5-carboxylic acid (1m)**

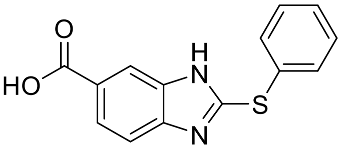

Off-white solid, yield: 90 mg, 87%; $^1$H NMR (400 MHz, $CD_3OD$): δ 8.18 (d, $J$ = 0.8 Hz, 1H), 7.93 (dd, $J$ = 8.5, 1.5 Hz, 1H), 7.60 – 7.57 (m, 2H), 7.51 (d, $J$ = 8.5 Hz, 1H), 7.47 – 7.44 (m, 3H); $^{13}C\{^1H\}$ NMR (151 MHz, $CD_3OD$): δ 170.2, 153.3, 143.5 (br), 140.4 (br), 134.1, 131.0, 130.8, 130.4, 126.3, 125.5, 117.9, 114.8; HRMS (ESI, m/z) calculated for $C_{14}H_{11}N_2O_2S$ $(M + H)^+$ 271.0536, found 271.0542.

**2-ethoxybenzimidazole-5-carboxylic acid (1n)**

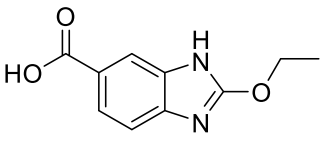

Beige solid, yield: 363 mg, 94%; $^1$H NMR (600 MHz, $CD_3OD$): δ 8.02 (d, $J$ = 1.2 Hz, 1H), 7.86 (dd, $J$ = 8.4, 1.6 Hz, 1H), 7.37 (d, $J$ = 8.3 Hz, 1H), 4.54 (q, $J$ = 7.1 Hz, 2H), 1.47 (t, $J$ = 7.1 Hz, 3H); $^{13}C\{^1H\}$ NMR (151 MHz, $CD_3OD$): δ 170.5, 161.3, 141.5, 137.2, 125.0, 124.7, 116.2, 113.7, 67.8, 14.8; HRMS (ESI, m/z) calculated for $C_{10}H_{11}N_2O_3$ $(M + H)^+$ 207.0764, found 207.0769.

**2-benzoylbenzimidazole-5-carboxylic acid (1o)**

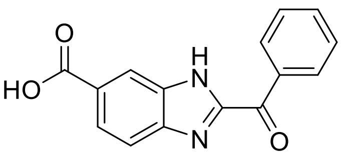

Pale yellow-orange solid, yield: 180 mg, 91%; $^1$H NMR (600 MHz, $CD_3OD$): δ 8.50 (dd, $J$ = 8.4, 1.2 Hz, 2H), 8.49 (s, 1H), 8.08 (dd, $J$ = 8.6, 1.4 Hz, 1H), 7.77 (d, $J$ = 8.6 Hz, 1H), 7.72 (tt, $J$ = 7.4, 1.2 Hz, 1H), 7.60 (t, $J$ = 7.8 Hz, 2H); $^{13}C\{^1H\}$ NMR (101 MHz, $CD_3OD$): δ 185.3, 169.8, 151.3, 137.0, 135.0, 132.0, 129.6, 128.1, 127.1, 121.3 (br), 116.6 (br); HRMS (ESI, m/z) calculated for $C_{15}H_{11}N_2O_3$ $(M + H)^+$ 267.0765, found 267.0772.

**2-(1-hydroxyethyl)benzimidazole-5-carboxylic acid (1p)**

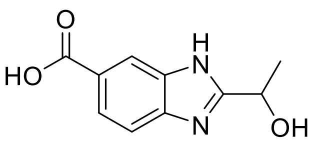

Beige solid, yield: 316 mg, 96%; $^1$H NMR (600 MHz, $CD_3OD$): δ 8.27 (d, $J$ = 1.4 Hz, 1H), 7.94 (dd, $J$ = 8.5, 1.4 Hz, 1H), 7.58 (d, $J$ = 8.5 Hz, 1H), 5.09 (q, $J$ = 6.7 Hz, 1H), 1.63 (d, $J$ = 6.7 Hz, 3H); $^{13}C\{^1H\}$ NMR (151 MHz, $CD_3OD$): δ 170.5, 162.5, 142.4, 139.2, 126.1, 125.1, 118.5, 115.2, 65.5, 23.1; HRMS (ESI, m/z) calculated for $C_{10}H_{11}N_2O_3$ $(M + H)^+$ 207.0764, found 207.0772.

**benzimidazole-5-carboxylic acid (1q)**

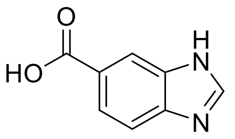

Off-white solid, yield: 443 mg, 90%; $^1$H NMR (400 MHz, $CD_3OD$): δ 8.35 (dd, $J$ = 1.4, 0.5 Hz, 1H), 8.33 (s, 1H), 7.99 (dd, $J$ = 8.5, 1.6 Hz, 1H), 7.67 (dd, $J$ = 8.5, 0.5 Hz, 1H); $^{13}C\{^1H\}$ NMR (151 MHz, $CD_3OD$): δ 170.4, 144.9, 141.6 (br), 130.9 (br), 126.7, 125.4, 119.1, 115.5; HRMS (ESI, m/z) calculated for $C_8H_7N_2O_2$ $(M + H)^+$ 163.0502, found 163.0500.

**2-aminobenzimidazole-5-carboxylic acid (1r)**

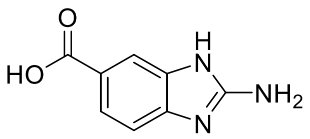

Red-orange solid, yield: 277 mg, 89%; $^1$H NMR (600 MHz, $(CD_3)_2SO$): δ 7.68 (d, $J$ = 8.6 1.4 Hz, 1H), 7.56 (dd, $J$ = 8.2, 1.6 Hz, 1H), 7.12 (d, $J$ = 8.2 Hz, 1H), 6.51 (s, 2H) ; $^{13}C\{^1H\}$ NMR (151 MHz, $(CD_3)_2SO$): δ 168.3, 157.0, 143.9 (br), 136.8 (br), 121.9, 121.2, 112.0, 111.5; HRMS (ESI, m/z) calculated for $C_8H_8N_3O_2$ $(M + H)^+$ 178.0612, found 178.0611.

**2-(furan-2-yl)benzimidazole-5-carboxylic acid (1s)**

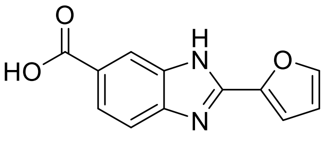

Pale orange solid, yield: 160 mg, 90%; $^1$H NMR (400 MHz, $CD_3OD$): δ 8.29 (d, $J$ = 0.9 Hz, 1H), 8.00 (dd, $J$ = 8.5, 1.5 Hz, 1H), 7.82 (dd, $J$ = 1.7, 0.6 Hz, 1H), 7.64 (dd, $J$ = 8.5, 0.4 Hz, 1H), 7.30 (dd, $J$ = 3.6, 0.6 Hz, 1H), 6.72 (dd, $J$ = 3.5, 1.8 Hz, 1H); $^{13}C\{^1H\}$ NMR (101 MHz, $CD_3OD$): δ 170.1, 147.3, 146.7, 145.5, 142.4, 139.1, 126.9, 126.0, 118.3, 115.3, 113.5, 113.5; HRMS (ESI, m/z) calculated for $C_{12}H_9N_2O_3$ $(M + H)^+$ 229.0608, found 229.0614.

**(*E*)-2-styrylbenzimidazole-5-carboxylic acid (1t)**

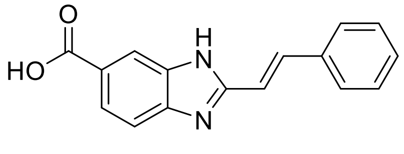

Pale yellow solid, yield: 164 mg, 82%; $^1$H NMR (600 MHz, $CD_3OD$): δ 8.27 (d, $J$ = 0.7 Hz, 1H), 8.00 (dd, $J$ = 8.5, 1.4 Hz, 1H), 7.73 (d, $J$ = 16.5 Hz, 1H), 7.66 (d, $J$ = 7.4 Hz, 2H), 7.62 (d, $J$ = 8.5 Hz, 1H), 7.44 (t, $J$ = 7.4 Hz, 2H), 7.39 (t, $J$ = 7.3 Hz, 1H), 7.19 (d, $J$ = 16.6 Hz, 1H); $^{13}C\{^1H\}$ NMR

(101 MHz, $CD_3OD$): δ 170.4, 154.9, 143.0, 139.8, 138.3, 136.8, 130.5, 130.0, 128.3, 126.6, 125.7, 118.2, 116.7, 115.0; HRMS (ESI, m/z) calculated for $C_{16}H_{13}N_2O_2$ $(M + H)^+$ 265.0972, found 265.0977.

**(*E*)-2-(prop-1-en-1-yl)benzimidazole-5-carboxylic acid (1u)**

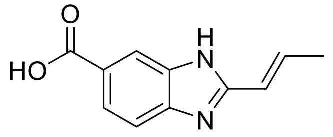

Pale yellow solid, yield: 85 mg, 86%; $^1H$ NMR (600 MHz, $CD_3OD$): δ 8.21 (dd, $J$ = 1.5, 0.5 Hz, 1H), 7.93 (dd, $J$ = 8.5, 1.5 Hz, 1H), 7.54 (dd, $J$ = 8.5, 0.4 Hz, 1H), 6.91 (dq, $J$ = 16.0, 6.8 Hz, 1H), 6.52 (dq, $J$ = 16.0, 1.7 Hz, 1H), 2.02 (dd, $J$ = 6.8, 1.8 Hz, 3H); $^{13}C\{^1H\}$ NMR (151 MHz, $CD_3OD$): δ 170.3, 154.8, 142.7, 139.4, 137.7, 126.4, 125.5, 120.8, 118.0, 114.9, 18.8; HRMS (ESI, m/z) calculated for $C_{11}H_{11}N_2O_2$ $(M + H)^+$ 203.0815, found 203.0820.

**(*Z*)-2-(prop-1-en-1-yl)benzimidazole-5-carboxylic acid (1v)**

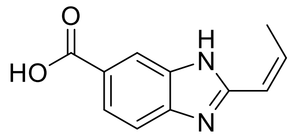

Beige solid, yield: 28 mg, 85%; $^1H$ NMR (600 MHz, $CD_3OD$): δ 8.28 (d, $J$ = 0.9 Hz, 1H), 7.94 (dd, $J$ = 8.5, 1.6 Hz, 1H), 7.59 (d, $J$ = 8.5 Hz, 1H), 6.45 (dq, $J$ = 11.7, 1.8 Hz, 1H), 6.30 (dq, $J$ = 11.7, 7.3 Hz, 1H), 2.22 (dd, $J$ = 7.3, 1.7 Hz, 3H); $^{13}C\{^1H\}$ NMR (151 MHz, $CD_3OD$): δ 170.5, 154.0, 142.8 (br), 139.4 (br), 137.8, 126.3, 125.4, 118.8, 118.4, 115.1, 16.0; HRMS (ESI, m/z) calculated for $C_{11}H_{11}N_2O_2$ $(M + H)^+$ 203.0815, found 203.0821.

**2-vinylbenzimidazole-5-carboxylic acid (1w)**

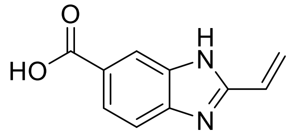

Off-white solid, yield: 146 mg, 78%; $^1H$ NMR (400 MHz, $CD_3OD$): δ 8.26 (s, 1H), 7.96 (dd, $J$ = 8.5, 1.3 Hz, 1H), 7.58 (d, $J$ = 8.5 Hz, 1H), 6.83 (dd, $J$ = 17.8, 11.3 Hz, 1H), 6.34 (d, $J$ = 17.8 Hz, 1H), 5.80 (d, $J$ = 11.4 Hz, 1H); $^{13}C\{^1H\}$ NMR (151 MHz, $CD_3OD$): δ 170.3, 154.5, 142.7 (br), 139.6 (br), 126.8, 126.6, 125.7, 123.2, 118.6, 115.2; HRMS (ESI, m/z) calculated for $C_{10}H_9N_2O_2$ $(M + H)^+$ 189.0659, found 189.0665.

**2-benzylbenzoxazole-5-carboxylic acid (1x)**

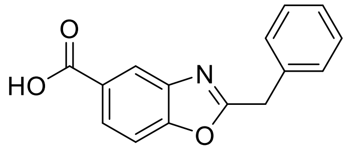

White solid, yield: 96 mg, 87%; $^1H$ NMR (400 MHz, $CD_3OD$): δ 8.30 (d, $J$ = 1.1 Hz, 1H), 8.08 (dd, $J$ = 8.6, 1.6 Hz, 1H), 7.62 (d, $J$ = 8.6 Hz, 1H), 7.38 (d, $J$ = 7.1 Hz, 2H), 7.35 (t, $J$ = 7.6 Hz, 2H); 7.28 (t, $J$ = 7.2 Hz, 1H), 4.33 (s, 2H); $^{13}C\{^1H\}$ NMR (151 MHz, $CD_3OD$): δ 169.2, 169.0, 155.2, 142.2, 135.9, 130.1, 129.9, 128.9, 128.5, 128.2, 122.3, 111.5, 35.7; HRMS (ESI, m/z) calculated for $C_{15}H_{12}NO_3$ $(M + H)^+$ 254.0812, found 254.0812.

**2-phenylbenzoxazole-5-carboxylic acid (1y)**

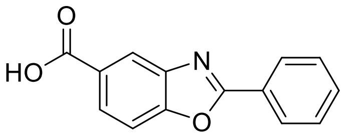

Off-white solid, yield: 40 mg, 85%; $^{1}H$ NMR (400 MHz, $CD_3OD$): δ 8.40 (dd, *J* = 1.6, 0.5 Hz, 1H), 8.29 – 8.26 (m, 2H), 8.15 (dd, *J* = 8.6, 1.7 Hz, 1H), 7.77 (dd, *J* = 8.6, 0.5 Hz, 1H), 7.67– 7.58 (m, 3H); $^{13}C\{^{1}H\}$ NMR (151 MHz, $CD_3OD$): δ 169.2, 166.0, 155.0, 143.1, 133.5, 130.3, 129.3, 128.8, 128.6, 127.6, 122.6, 111.7; HRMS (ESI, m/z) calculated for $C_{14}H_{10}NO_3$ $(M + H)^+$ 240.0655, found 240.0661.

**2-phenylbenzimidazole-5-carboxylic acid (1z)**

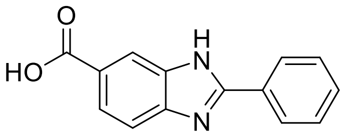

Off-white solid, yield: 177 mg, 91%; $^{1}H$ NMR (400 MHz, $CD_3OD$): δ 8.34 (d, *J* = 0.9 Hz, 1H), 8.14 – 8.11 (m, 2H), 7.99 (dd, *J* = 8.5, 1.5 Hz, 1H), 7.66 (d, *J* = 8.5 Hz, 1H), 7.60 – 7.53 (m, 3H); $^{13}C\{^{1}H\}$ NMR (101 MHz, $CD_3OD$): δ 170.4, 155.7, 143.4 (br), 140.1 (br), 131.9, 130.4, 130.2, 128.0, 126.6, 125.6, 118.5, 115.3; HRMS (ESI, m/z) calculated for $C_{14}H_{11}N_2O_2$ $(M + H)^+$ 239.0815, found 239.0820.

**2-(*o*-tolyl)benzimidazole-5-carboxylic acid (1aa)**

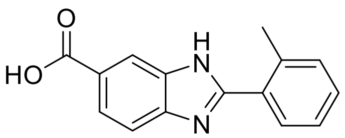

Off-white solid, yield: 180 mg, 81%; $^{1}H$ NMR (400 MHz, $CD_3OD$): δ 8.36 (dd, *J* = 1.6, 0.6 Hz, 1H), 8.00 (dd, *J* = 8.5, 1.6 Hz, 1H), 7.68 (dd, *J* = 8.5, 0.6 Hz, 1H), 7.64 (dd, *J* = 7.7, 1.2 Hz, 1H), 7.44 (td, *J* = 7.4, 1.4 Hz, 1H), 7.40 (d, *J* = 7.7 Hz, 1H), 7.36 (t, *J* = 7.5 Hz, 1H), 2.52 (s, 3H); $^{13}C\{^{1}H\}$ NMR (151 MHz, $CD_3OD$): δ 170.5, 156.4, 142.8, 139.7, 138.6, 132.2, 131.4, 130.9, 130.7, 127.2, 126.3, 125.5, 118.6, 115.3, 20.5; HRMS (ESI, m/z) calculated for $C_{15}H_{13}N_2O_2$ $(M + H)^+$ 253.0972, found 253.0972.

**2-(*p*-tolyl)benzimidazole-5-carboxylic acid (1ab)**

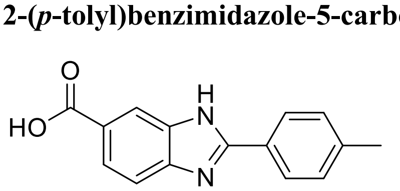

Off-white solid, yield: 197 mg, 92%; $^{1}H$ NMR (600 MHz, $CD_3OD$): δ 8.32 (s, 1H), 8.01 (d, *J* = 8.2 Hz, 2H), 7.99 (dd, *J* = 8.5, 1.4 Hz, 1H), 7.65 (d, *J* = 8.5 Hz, 1H), 7.40 (d, *J* = 8.0 Hz, 2H), 2.44 (s, 3H); $^{13}C\{^{1}H\}$ NMR (151 MHz, $CD_3OD$): δ 170.3, 155.7, 143.0, 142.8, 139.4, 130.9, 128.0, 127.0, 126.6, 125.7, 118.2, 115.2, 21.5; HRMS (ESI, m/z) calculated for $C_{15}H_{13}N_2O_2$ $(M + H)^+$ 253.0972, found 253.0974.

**2-(*m*-tolyl)benzimidazole-5-carboxylic acid (1ac)**

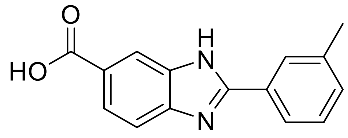

Off-white solid, yield: 195 mg, 93%; $^{1}$H NMR (400 MHz, $CD_3OD$): δ 8.32 (d, *J* = 1.0 Hz, 1H), 8.00 (dd, *J* = 8.5, 1.5 Hz, 1H), 7.94 (s, 1H), 7.90 (d, *J* = 7.8 Hz, 1H), 7.66 (d, *J* = 8.5 Hz, 1H), 7.46 (t, *J* = 7.6 Hz, 1H), 7.39 (d, *J* = 7.6 Hz, 1H), 2.47 (s, 3H); $^{13}$C{$^{1}$H} NMR (151 MHz, $CD_3OD$): δ 170.1, 156.6, 142.6, 140.4, 139.3, 133.0, 130.2, 129.6, 128.6, 126.9, 125.9, 125.3, 118.3, 115.2, 21.4; HRMS (ESI, m/z) calculated for $C_{15}H_{13}N_2O_2$ $(M + H)^+$ 253.0972, found 253.0977.

**2-(2-methoxyphenyl)benzimidazole-5-carboxylic acid (1ad)**

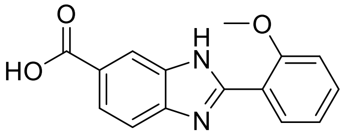

Beige solid, yield: 210 mg, 94%; $^{1}$H NMR (600 MHz, $CD_3OD$): δ 8.37 (d, *J* = 0.9 Hz, 1H), 8.26 (dd, *J* = 7.8, 1.6 Hz, 1H), 7.97 (dd, *J* = 8.5, 1.5 Hz, 1H), 7.68 (dd, *J* = 8.5, 0.5 Hz, 1H), 7.52 (td, *J* = 7.9, 1.7 Hz, 1H), 7.24 (d, *J* = 8.3 Hz, 1H), 7.14 (td, *J* = 7.6, 0.9 Hz, 1H), 4.08 (s, 3H); $^{13}$C{$^{1}$H} NMR (151 MHz, $CD_3OD$): δ 170.7, 158.9, 153.5, 142.7 (br), 139.2 (br), 133.5, 131.2, 126.3, 125.3, 122.3, 118.5, 118.3, 115.4, 113.0, 56.4; HRMS (ESI, m/z) calculated for $C_{15}H_{13}N_2O_3$ $(M + H)^+$ 269.0921, found 269.0926.

**2-(2-hydroxyphenyl)benzimidazole-5-carboxylic acid (1ae)**

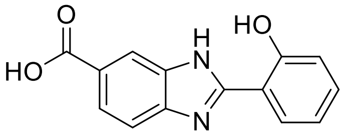

Beige solid, yield: 89 mg, 96%; $^{1}$H NMR (600 MHz, $CD_3OD$): δ 8.31 (d, *J* = 1.0 Hz, 1H), 7.97 (dd, *J* = 8.5, 1.5 Hz, 1H), 7.94 (dd, *J* = 7.9, 1.4 Hz, 1H), 7.65 (d, *J* = 8.5 Hz, 1H), 7.37 (td, *J* = 7.8, 1.6 Hz, 1H), 7.03 (dd, *J* = 8.3, 0.7 Hz, 1H), 6.99 (td, *J* = 7.6, 0.9 Hz, 1H); $^{13}$C{$^{1}$H} NMR (151 MHz, $CD_3OD$): δ 170.4, 159.5, 155.3, 142.1 (br), 138.6 (br), 133.3, 127.6, 126.5, 125.6, 120.5, 118.3, 118.1 (br), 115.1 (br), 114.0; HRMS (ESI, m/z) calculated for $C_{14}H_{11}N_2O_3$ $(M + H)^+$ 255.0764, found 255.0765.

**2-(2-aminophenyl)benzimidazole-5-carboxylic acid (1af)**

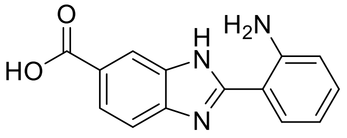

Pale yellow solid, yield: 159 mg, 91%; $^{1}$H NMR (600 MHz, $CD_3OD$): δ 8.29 (s, 1H), 7.94 (dd, *J* = 8.4, 1.5 Hz, 1H), 7.73 (dd, *J* = 7.9, 1.3 Hz, 1H), 7.61 (d, *J* = 8.4, 1H), 7.21 (td, *J* = 7.7, 1.4 Hz, 1H), 6.88 (d, *J* = 8.2 Hz, 1H), 6.75 (td, *J* = 7.5, 1.0 Hz, 1H); $^{13}$C{$^{1}$H} NMR (151 MHz, $CD_3OD$): δ 170.6, 156.5, 149.4, 143.1 (br), 139.8 (br), 132.2, 128.7, 125.7, 125.2, 118.1 (br), 118.0, 117.7, 114.9 (br), 112.4; HRMS (ESI, m/z) calculated for $C_{14}H_{12}N_3O_2$ $(M + H)^+$ 254.0924, found 254.0924.

**2-(2-(trifluoromethyl)phenyl)benzimidazole-5-carboxylic acid (1ag)**

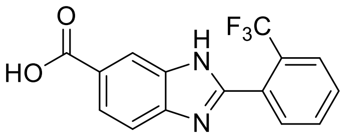

Off-white solid, yield: 248 mg, 94%; $^{1}H$ NMR (600 MHz, $CD_3OD$): δ 8.37 (s, 1H), 8.03 (dd, *J* = 8.5, 1.5 Hz, 1H), 7.94 (d, *J* = 7.4 Hz, 1H), 7.83 – 7.76 (m, 3H), 7.83 – 7.80 (m, 1H), 7.69 (d, *J* = 8.3 Hz, 1H); $^{13}C\{^{1}H\}$ NMR (151 MHz, $CD_3OD$): δ 170.2, 153.6, 133.3, 133.3, 131.9, 130.5, 130.5 (q, *J* = 31 Hz), 127.7 (q, *J* = 5 Hz), 126.8, 125.7, 125.1 (q, *J* = 273 Hz), 118.9 (br), 115.4 (br); $^{19}F$ NMR (564 MHz, $CD_3OD$): δ -60.0; HRMS (ESI, m/z) calculated for $C_{15}H_{10}F_3N_2O_2$ $(M + H)^+$ 307.0689, found 307.0693.

**2-(2-nitrophenyl)benzimidazole-5-carboxylic acid (1ah)**

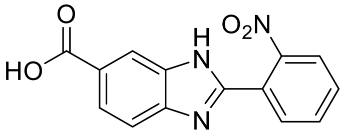

Yellow-orange solid, yield: 191 mg, 84%; $^{1}H$ NMR (600 MHz, $CD_3OD$): δ 8.35 (s, 1H), 8.17 (d, *J* = 8.5 Hz, 1H), 8.02 (dd, *J* = 8.5, 1.4 Hz, 1H), 7.90 – 7.86 (m, 2H), 7.83 – 7.80 (m, 1H), 7.67 (d, *J* = 8.6 Hz, 1H); $^{13}C\{^{1}H\}$ NMR (151 MHz, $CD_3OD$): δ 170.2, 152.4, 150.1, 134.5, 133.1, 132.7, 126.8, 126.4, 126.0, 125.7, 119.1 (br), 115.5 (br); HRMS (ESI, m/z) calculated for $C_{14}H_{10}N_3O_4$ $(M + H)^+$ 284.0666, found 284.0675.

**2-(pyridin-4-yl)benzimidazole-5-carboxylic acid (1ai)**

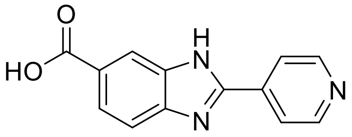

Pale yellow solid, yield: 180 mg, 95%; $^{1}H$ NMR (400 MHz, $CD_3OD$): δ 8.79 – 8.77 (m, 2H), 8.40 (s, 1H), 8.16 – 8.14 (m, 2H), 8.05 (dd, *J* = 8.6, 1.5 Hz, 1H), 7.74 (d, *J* = 8.6 Hz, 1H); $^{13}C\{^{1}H\}$ NMR (151 MHz, $(CD_3)_2SO$): δ 167.6, 151.1, 150.5, 136.7, 125.4, 124.2, 120.6; HRMS (ESI, m/z) calculated for $C_{13}H_{10}N_3O_2$ $(M + H)^+$ 240.0768, found 240.0768.

**2-(thiophen-2-yl)benzimidazole-5-carboxylic acid (1aj)**

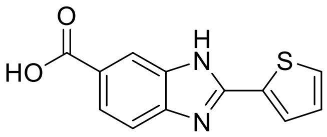

Pale orange solid, yield: 198 mg, 96%; $^{1}H$ NMR (400 MHz, $CD_3OD$): δ 8.28 (dd, *J* = 1.5, 0.6 Hz, 1H), 7.98 (dd, *J* = 8.5, 1.5 Hz, 1H), 7.85 (dd, *J* = 3.7, 1.1 Hz, 1H), 7.72 (dd, *J* = 5.0, 1.1 Hz, 1H), 7.61 (dd, *J* = 8.5, 0.6 Hz, 1H), 7.25 (dd, *J* = 5.0, 3.8 Hz, 1H); $^{13}C\{^{1}H\}$ NMR (101 MHz, $CD_3OD$): δ 170.2, 150.9, 142.9, 139.5, 132.8, 130.8, 129.4, 129.4, 126.6, 125.8, 118.1, 115.1; HRMS (ESI, m/z) calculated for $C_{12}H_9N_2O_2S$ $(M + H)^+$ 245.0379, found 245.0379.

**2-(1-methylpyrrol-2-yl)benzimidazole-5-carboxylic acid (1ak)**

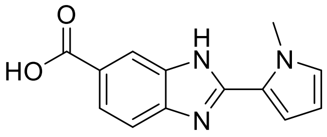

Pale orange solid, yield: 167 mg, 98%; $^{1}$H NMR (400 MHz, $CD_3OD$): δ 8.27 (dd, $J$ = 1.4, 0.5 Hz, 1H), 7.96 (dd, $J$ = 8.5, 1.6 Hz, 1H), 7.60 (dd, $J$ = 8.5, 0.5 Hz, 1H), 6.98 (t, $J$ = 2.1 Hz, 1H), 6.88 (dd, $J$ = 3.9, 1.7 Hz, 1H), 6.24 (dd, $J$ = 3.9, 2.6 Hz, 1H), 4.08 (s, 3H); $^{13}$C{$^{1}$H} NMR (151 MHz, $CD_3OD$): δ 170.4, 150.0, 142.6, 139.2, 129.5, 126.1, 125.5, 123.0, 117.7, 114.7, 114.5, 109.7, 36.8; HRMS (ESI, m/z) calculated for $C_{13}H_{12}N_3O_2$ $(M + H)^+$ 242.0924, found 242.0930.

**2-(pyrrol-2-yl)benzimidazole-5-carboxylic acid (1al)**

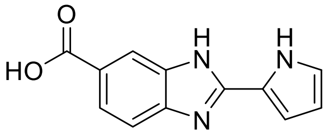

Pale brown solid, yield: 114 mg, 65%; $^{1}$H NMR (600 MHz, $CD_3OD$): δ 8.21 (d, $J$ = 1.0 Hz, 1H), 7.93 (dd, $J$ = 8.4, 1.6 Hz, 1H), 7.55 (d, $J$ = 8.5 Hz, 1H), 7.05 (dd, $J$ = 2.6, 1.4 Hz, 1H), 6.94 (dd, $J$ = 3.7, 1.4 Hz, 1H), 6.30 (dd, $J$ = 3.7, 2.6 Hz, 1H); $^{13}$C{$^{1}$H} NMR (151 MHz, $CD_3OD$): δ 170.6, 150.6, 125.9, 125.3, 123.5, 122.6, 117.4 (br), 114.6 (br), 112.0, 111.0; HRMS (ESI, m/z) calculated for $C_{12}H_{10}N_3O_2$ $(M + H)^+$ 228.0768, found 228.0774.

**2-(naphthalen-1-yl)benzimidazole-5-carboxylic acid (1am)**

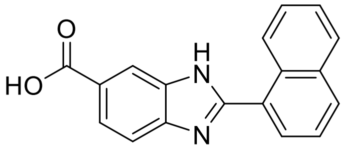

Off-white solid, yield: 226 mg, 90%; $^{1}$H NMR (600 MHz, $CD_3OD$): δ 8.48 – 8.45 (m, 1H), 8.43 (d, $J$ = 0.9 Hz, 1H), 8.11 (d, $J$ = 8.3 Hz, 1H), 8.07 (dd, $J$ = 8.5, 1.5 Hz, 1H), 8.03 – 8.00 (m, 1H), 7.92 (dd, $J$ = 7.1, 1.1 Hz, 1H), 7.75 (d, $J$ = 8.5 Hz, 1H), 7.66 (dd, $J$ = 8.2, 7.2 Hz, 1H), 7.64 – 7.59 (m, 2H); $^{13}$C{$^{1}$H} NMR (151 MHz, $CD_3OD$): δ 170.2, 155.5, 142.5, 139.5, 135.4, 132.3, 132.3, 129.7, 129.6, 128.5, 128.1, 127.7, 126.8, 126.3, 126.2, 125.8, 118.7, 115.4; HRMS (ESI, m/z) calculated for $C_{18}H_{13}N_2O_2$ $(M + H)^+$ 289.0972, found 289.0977.

**2-(indol-3-yl)benzimidazole-5-carboxylic acid (1an)**

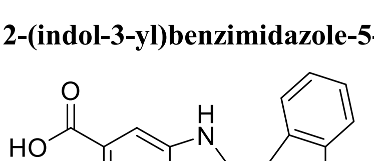

Pale yellow solid, yield: 167 mg, 87%; $^{1}$H NMR (600 MHz, $CD_3OD$): δ 8.29 (d, $J$ = 1.1 Hz, 1H), 8.29 – 8.26 (m, 1H), 8.08 (s, 1H), 7.97 (dd, $J$ = 8.4, 1.4 Hz, 1H), 7.63 (d, $J$ = 8.4 Hz, 1H), 7.52 – 7.49 (m, 1H), 7.29 – 7.25 (m, 2H); $^{13}$C{$^{1}$H} NMR (151 MHz, $CD_3OD$): δ 170.5, 153.1, 142.4, 138.6, 138.5, 128.8, 126.3, 126.0, 125.5, 124.1, 122.3, 121.1, 117.1, 114.4, 113.1, 106.0; HRMS (ESI, m/z) calculated for $C_{16}H_{12}N_3O_2$ $(M + H)^+$ 278.0924, found 278.0930.

**2-(benzo[*d*][1,3]dioxol-5-yl)benzimidazole-5-carboxylic acid (1ao)**

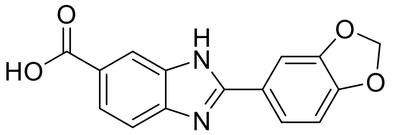

Beige solid, yield: 219 mg, 91%; $^{1}$H NMR (600 MHz, $CD_3OD$): δ 8.28 (d, *J* = 0.8 Hz, 1H), 7.99 (dd, *J* = 8.5, 1.5 Hz, 1H), 7.65 (dd, *J* = 8.2, 1.7 Hz, 1H), 7.63 (d, *J* = 8.5 Hz, 1H), 7.57 (d, *J* = 1.7 Hz, 1H), 7.02 (d, *J* = 8.2 Hz, 1H), 6.09 (s, *2*H); $^{13}C\{^1H\}$ NMR (151 MHz, $CD_3OD$): δ 170.0, 155.1, 151.9, 150.0, 142.2, 138.8, 126.8, 125.9, 123.1, 123.1, 117.9, 115.0, 109.9, 107.9, 103.4; HRMS (ESI, m/z) calculated for $C_{15}H_{11}N_2O_4$ $(M + H)^+$ 283.0713, found 283.0722.

**2-(pyren-1-yl)benzimidazole-5-carboxylic acid (1ap)**

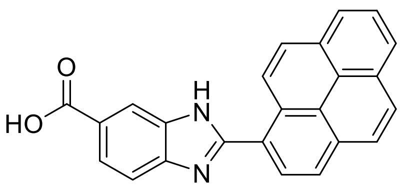

Dark yellow solid, yield: 298 mg, 98%; $^{1}$H NMR (600 MHz, $CD_3OD$): δ 8.77 (d, *J* = 9.2 Hz, 1H), 8.48 (d, *J* = 0.8 Hz, 1H), 8.40 (d, *J* = 7.9 Hz, 1H), 8.39 (d, *J* = 8.0 Hz, 1H), 8.33 (d, *J* = 7.6 Hz, 1H), 8.32 (d, *J* = 7.6 Hz, 1H), 8.28 (d, *J* = 9.4 Hz, 1H), 8.26 (d, *J* = 8.9 Hz, 1H), 8.20 (d, *J* = 8.9 Hz, 1H), 8.12 (t, *J* = 7.6 Hz, 1H), 8.11 (dd, *J* = 8.6, 1.6 Hz, 1H), 7.80 (d, *J* = 8.5 Hz, 1H); $^{13}C\{^1H\}$ NMR (151 MHz, $(CD_3)_2SO$): δ 167.8, 153.7, 141.7 (br), 138.4 (br), 132.2, 130.8, 130.2, 129.0, 128.9, 128.9, 127.6, 127.3, 126.8, 126.2, 125.9, 125.2, 125.2, 124.9, 124.2, 124.0, 123.6, 123.0, 117.1 (br), 114.9 (br); HRMS (ESI, m/z) calculated for $C_{24}H_{15}N_2O_2$ $(M + H)^+$ 363.1129, found 363.1143.

**Methyl 2-(chloromethyl)benzimidazole-5-carboxylate (1ar*)**

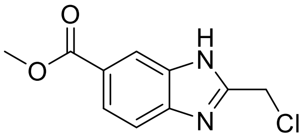

Off-white solid, yield: 432 mg, 64%; Eluent: EA/Hexane = 2:3; $^{1}$H NMR (600 MHz, $CDCl_3$): δ 8.35 (br, 1H), 8.02 (dd, *J* = 8.5, 1.5 Hz, 1H), 7.62 (br, 1H), 4.87 (s, 2H), 3.95 (s, 3H); HRMS (ESI, m/z) calculated for $C_{10}H_{10}ClN_2O_2$ $(M + H)^+$ 225.0425, found 225.0434.

**((5-(methoxycarbonyl)benzimidazol-2-yl)methyl)triphenylphosphonium chloride (1as*)**

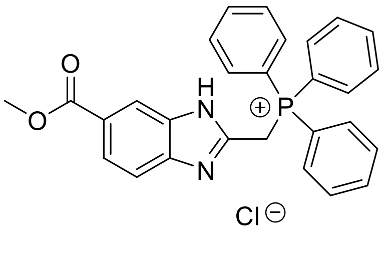

Off-white solid, yield: 716 mg, 77%; $^{1}$H NMR (600 MHz, $CDCl_3$): δ 8.24 (s, 1H), 7.87 (dd, *J* = 8.5, 1.4 Hz, 1H), 7.82 (ddd, *J* = 13.1, 8.4, 1.1 Hz, 6H), 7.75 (t, *J* = 7.6 Hz, 3H), 7.61 (td, *J* = 7.9, 3.6 Hz, 6H), 7.49 (d, *J* = 8.4 Hz, 1H), 5.59 (d, *J* = 14.5 Hz, 2H), 3.89 (s, 3H); $^{31}P$ NMR (243 MHz, $CDCl_3$): δ 22.0.

**Methyl thiobenzimidazolinone-5-carboxylate (1at*)**

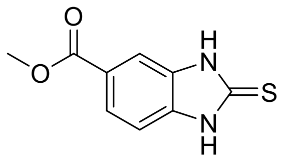

Pale orange solid, yield: 1232 mg, 99%; $^1H$ NMR (400 MHz, $(CD_3)_2SO$): δ 12.83 (br, 2H), 7.77 (dd, $J$ = 8.4, 1.5 Hz, 1H), 7.66 (d, $J$ = 1.3 Hz, 1H), 7.23 (br, $J$ = 8.4 Hz, 1H), 3.84 (s, 3H); $^{13}C\{^1H\}$ NMR (101 MHz, $CD_3OD$): δ 171.9, 168.3, 137.4, 133.7, 125.8, 125.8, 111.9, 110.3, 52.7; HRMS (ESI, m/z) calculated for $C_9H_9N_2O_2S$ $(M + H)^+$ 209.0379, found 209.0383.

**2-(phenyl-λ$^3$-iodaneylidene)cyclohexane-1,3-dione (2a)**

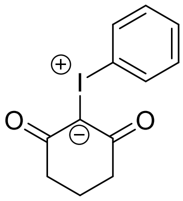

White solid, yield: 1419 mg, 90%; $^1H$ NMR (400 MHz, $(CD_3)_2SO$): δ 7.69 (d, $J$ = 7.2 Hz, 2H), 7.49 (t, $J$ = 7.4 Hz, 1H), 7.39 (t, $J$ = 7.5 Hz, 2H), 2.42 (t, $J$ = 6.3 Hz, 4H), 1.81 (quin, $J$ = 6.3 Hz, 2H); $^{13}C\{^1H\}$ NMR (101 MHz, $(CD_3)_2SO$): δ 187.7, 132.2, 130.9, 130.1, 113.4, 96.6, 36.4, 21.0; HRMS (ESI, m/z) calculated for $C_{12}H_{12}IO_2$ $(M + H)^+$ 314.9877, found 314.9878.

**2-(phenyl-λ$^3$-iodaneylidene)-5-methylcyclohexane-1,3-dione (2b)**

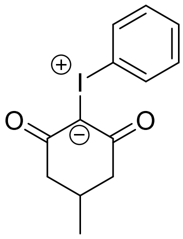

Beige solid, yield: 291 mg, 89%; $^1H$ NMR (400 MHz, $(CD_3)_2SO$): δ 7.68 (d, $J$ = 8.3 Hz, 2H), 7.48 (t, $J$ = 7.3 Hz, 1H), 7.38 (t, $J$ = 7.6 Hz, 2H), 2.48 – 2.44 (m, 2H), 2.22 – 2.16 (m, 2H), 2.14 – 2.06 (m, 1H), 0.99 (d, $J$ = 6.2 Hz, 3H); $^{13}C\{^1H\}$ NMR (101 MHz, $(CD_3)_2SO$): δ 187.2, 132.2, 130.9, 130.1, 113.5, 96.1, 44.5, 28.0, 20.5; HRMS (ESI, m/z) calculated for $C_{13}H_{14}IO_2$ $(M + H)^+$ 329.0033, found 329.0041.

**2-(phenyl-λ$^3$-iodaneylidene)-5,5-dimethylcyclohexane-1,3-dione (2c)**

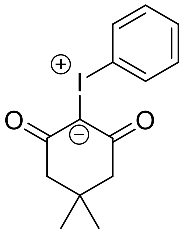

Beige solid, yield: 232 mg, 68%; $^1H$ NMR (400 MHz, $(CD_3)_2SO$): δ 7.69 (d, $J$ = 7.2 Hz, 2H), 7.48 (t, $J$ = 7.4 Hz, 1H), 7.38 (t, $J$ = 7.6 Hz, 2H), 2.31 (s, 4H), 0.98 (s, 6H); $^{13}C\{^1H\}$ NMR (101 MHz, $(CD_3)_2SO$): δ 186.8, 132.4, 130.9, 130.2, 114.0, 95.0, 50.1, 31.7, 28.0; HRMS (ESI, m/z) calculated for $C_{14}H_{16}IO_2$ $(M + H)^+$ 343.0190, found 343.0197.

**2-(phenyl-λ$^3$-iodaneylidene)-5-phenylcyclohexane-1,3-dione (2d)**

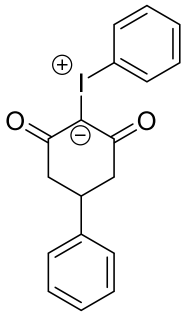

Beige solid, yield: 343 mg, 88%; $^1H$ NMR (400 MHz, $(CD_3)_2SO$): δ 7.70 (d, *J* = 7.8 Hz, 2H), 7.50 (t, *J* = 7.4 Hz, 1H), 7.38 (t, *J* = 7.8 Hz, 2H), 7.33 – 7.27 (m, 4H), 7.21 (t, *J* = 6.8 Hz, 1H), 3.33 – 3.27 (m, 1H), 2.76 (dd, *J* = 15.9, 10.9 Hz, 2H), 2.63 (dd, *J* = 16.0, 4.4 Hz, 2H); $^{13}C\{^1H\}$ NMR (101 MHz, $(CD_3)_2SO$): δ 186.6, 143.8, 132.4, 131.0, 130.2, 128.4, 126.9, 126.4, 113.5, 95.9, 43.4, 38.4; HRMS (ESI, m/z) calculated for $C_{18}H_{16}IO_2$ $(M + H)^+$ 391.0190, found 391.0195.

**4-(phenyl-λ$^3$-iodaneylidene)-(2*H*,4*H*,6*H*)-pyran-3,5-dione (2e)**

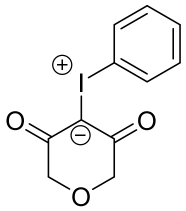

Beige solid, yield: 119 mg, 38%; $^1H$ NMR (400 MHz, $(CD_3)_2SO$): δ 7.75 (d, *J* = 8.1 Hz, 2H), 7.51 (t, *J* = 7.4 Hz, 1H), 7.41 (t, *J* = 7.6 Hz, 2H), 4.15 (s, 4H); $^{13}C\{^1H\}$ NMR (101 MHz, $(CD_3)_2SO$): δ 185.2, 132.9, 131.1, 130.4, 114.0, 90.0, 71.3; HRMS (ESI, m/z) calculated for $C_{11}H_{10}IO_3$ $(M + H)^+$ 316.9669, found 316.9674.

**2-(phenyl-λ$^3$-iodaneylidene)cyclopentane-1,3-dione (2f)**

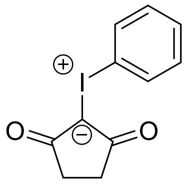

Beige solid, yield: 245 mg, 33%; $^1H$ NMR (400 MHz, $(CD_3)_2SO$): δ 7.75 (d, *J* = 7.3 Hz, 2H), 7.52 (t, *J* = 7.4 Hz, 1H), 7.41 (t, *J* = 7.6 Hz, 2H), 2.47 (s, 4H); $^{13}C\{^1H\}$ NMR (101 MHz, $(CD_3)_2SO$): δ 195.7, 132.4, 131.1, 130.5, 115.6, 85.4, 33.5; HRMS (ESI, m/z) calculated for $C_{11}H_{10}IO_2$ $(M + H)^+$ 300.9720, found 300.9726.

**2-(phenyl-λ$^3$-iodaneylidene)cycloheptane-1,3-dione (2g)**

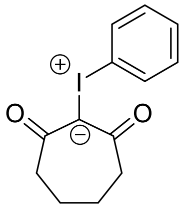

Beige solid, yield: 251 mg, 77%; $^1H$ NMR (400 MHz, $(CD_3)_2SO$): δ 7.69 (d, *J* = 7.2 Hz, 2H), 7.48 (t, *J* = 7.3 Hz, 1H), 7.39 (t, *J* = 7.5 Hz, 2H), 2.70 (t, *J* = 6.1 Hz, 4H), 1.76 (quin, *J* = 3.1 Hz, 4H); $^{13}C\{^1H\}$ NMR (101 MHz, $(CD_3)_2SO$): δ 188.9, 131.9, 130.8, 130.0, 113.1, 104.3, 36.7, 21.5; HRMS (ESI, m/z) calculated for $C_{13}H_{14}IO_2$ $(M + H)^+$ 329.0033, found 329.0038.

**2,2-dimethyl-5-(phenyl-λ$^3$-iodaneylidene)-1,3-dioxane-4,6-dione (2h)**

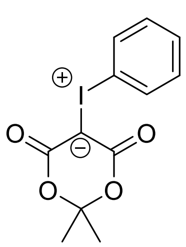

White solid, yield: 318 mg, 92%; $^1H$ NMR (400 MHz, $(CD_3)_2SO$): δ 7.79 (d, *J* = 7.3 Hz, 2H), 7.55 (t, *J* = 7.4 Hz, 1H), 7.46 (t, *J* = 7.6 Hz, 2H), 1.56 (s, 6H); $^{13}C\{^1H\}$ NMR (101 MHz, $(CD_3)_2SO$): δ 162.9, 132.5, 131.0, 130.7, 116.4, 102.8, 57.0, 25.6; HRMS (ESI, m/z) calculated for $C_{12}H_{12}IO_4$ $(M + H)^+$ 346.9775, found 346.9775.

**1,3-dimethyl-5-(phenyl-λ$^3$-iodaneylidene)-(1*H*,3*H*,5*H*)-pyrimidine-2,4,6-trione (2i)**

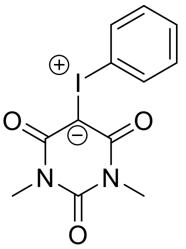

White solid, yield: 279 mg, 78%; $^1$H NMR (400 MHz, $(CD_3)_2SO$): δ 7.80 (d, *J* = 7.3 Hz, 2H), 7.53 (t, *J* = 7.4 Hz, 1H), 7.43 (t, *J* = 7.6 Hz, 2H), 3.16 (s, 6H); $^{13}C\{^1H\}$ NMR (101 MHz, $(CD_3)_2SO$): δ 160.9, 152.6, 132.4, 131.1, 130.6, 116.1, 70.0, 28.6.

**3-(phenyl-λ$^3$-iodaneylidene)chromane-2,4-dione (2j)**

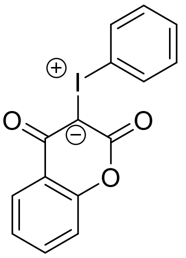

White solid; $^1$H NMR (400 MHz, $(CD_3)_2SO$): δ 7.90 (dd, *J* = 7.8, 1.6 Hz, 1H), 7.85 (d, *J* = 8.2 Hz, 2H), 7.57 (td, *J* = 7.7, 1.5 Hz, 1H), 7.53 (t, *J* = 7.6 Hz, 1H), 7.42 (t, *J* = 7.7 Hz, 2H), 7.28 (d, *J* = 8.0 Hz, 1H), 7.26 (t, *J* = 7.5 Hz, 1H); $^{13}C\{^1H\}$ NMR (101 MHz, $(CD_3)_2SO$): δ 172.6, 161.0, 154.0, 133.1, 132.9, 131.2, 130.8, 125.7, 123.5, 119.9, 116.3, 115.1, 82.1; HRMS (ESI, m/z) calculated for $C_{15}H_{10}IO_3$ $(M + H)^+$ 364.9669, found 364.9667.

**6-chloro-3-(phenyl-λ$^3$-iodaneylidene)chromane-2,4-dione (2k)**

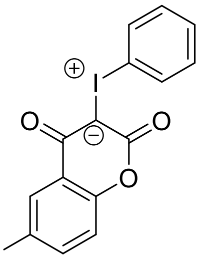

White solid; $^1$H NMR (400 MHz, $(CD_3)_2SO$): δ 7.83 (d, *J* = 7.2 Hz, 2H), 7.82 (d, *J* = 2.7 Hz, 1H), 7.61 (dd, *J* = 8.7, 2.7 Hz, 1H), 7.54 (t, *J* = 7.4 Hz, 1H), 7.43 (t, *J* = 7.8 Hz, 2H), 7.34 (d, *J* = 8.8 Hz, 1H); $^{13}C\{^1H\}$ NMR (101 MHz, $(CD_3)_2SO$): δ 171.3, 160.6, 152.6, 133.3, 132.6, 131.2, 130.9, 127.7, 124.7, 121.2 118.6, 115.2, 82.2; found 314.9878; HRMS (ESI, m/z) calculated for $C_{15}H_9ClIO_3$ $(M + H)^+$ 398.9279, found 398.9285.

**3-(phenyl-λ$^3$-iodaneylidene)-(3*H*)-naphthalene-1,2,4-trione (2l)**

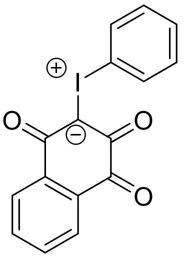

Yellow-orange solid; $^1$H NMR (400 MHz, $(CD_3)_2SO$): δ 8.05 (dd, *J* = 7.7, 1.1 Hz, 1H), 7.97 (dd, *J* = 7.6, 1.0 Hz, 1H), 7.85 (d, *J* = 7.4 Hz, 2H), 7.81 (td, *J* = 7.5, 1.3 Hz, 1H), 7.72 (td, *J* = 7.5, 1.3 Hz, 1H), 7.51 (t, *J* = 7.4 Hz, 1H), 7.40 (t, *J* = 7.6 Hz, 2H); $^{13}C\{^1H\}$ NMR (101 MHz, $(CD_3)_2SO$): δ 181.0, 174.9, 170.0, 134.7, 133.3, 133.1, 132.2, 131.2, 130.9, 130.6, 126.9, 126.9, 114.0, 103.0; HRMS (ESI, m/z) calculated for $C_{16}H_{10}IO_3$ $(M + H)^+$ 376.9669, found 376.9674.

**2-benzyl-8,9,10-trihydrobenzo[3,4]isochromeno[5,6-*d*]imidazole-6,11-dione (3a)**

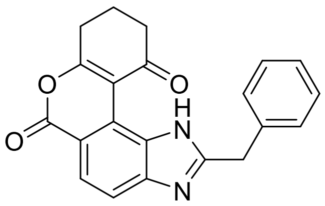

White solid, yield: 17.5 mg, 51%; Eluent: EA/DCM = 1:4; $^{1}$H NMR (400 MHz, $CDCl_3$): δ 12.54 (br, 1H), 8.21 (d, *J* = 8.6 Hz, 1H), 7.89 (d, *J* = 8.6 Hz, 1H), 7.41 – 7.37 (m, 4H), 7.35 – 7.29 (m, 1H), 4.38 (s, 2H), 3.01 (t, *J* = 6.4 Hz, 2H), 2.72 (t, *J* = 6.7 Hz, 2H), 2.17 (quin, *J* = 6.5 Hz, 2H); $^{13}$C{$^{1}$H} NMR (101 MHz, $CDCl_3$): δ 199.0, 171.7, 160.5, 156.3, 149.5, 136.0, 129.2, 129.1, 128.9, 127.5, 124.1, 120.8, 119.5, 113.8, 112.8, 39.5, 36.3, 30.1, 19.7; HRMS (ESI, m/z) calculated for $C_{21}H_{17}N_2O_3$ $(M + H)^+$ 345.1234, found 345.1233.

**2-phenethyl-8,9,10-trihydrobenzo[3,4]isochromeno[5,6-*d*]imidazole-6,11-dione (3b)**

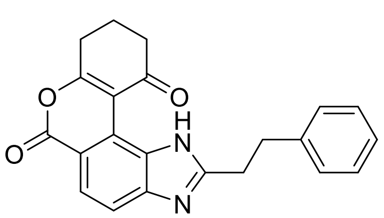

White solid, yield: 13.6 mg, 38%; Eluent: EA/DCM = 1:4; $^{1}$H NMR (400 MHz, $CDCl_3$): δ 12.48 (br, 1H), 8.20 (d, *J* = 8.6 Hz, 1H), 7.86 (d, *J* = 8.6 Hz, 1H), 7.32 – 7.19 (m, 5H), 3.34 – 3.22 (m, 4H), 3.03 (t, *J* = 6.4 Hz, 2H), 2.77 (t, *J* = 6.7 Hz, 2H), 2.20 (quin, *J* = 6.5 Hz, 2H); $^{13}$C{$^{1}$H} NMR (101 MHz, $CDCl_3$): δ 199.1, 171.8, 160.5, 156.9, 149.4, 140.4, 128.8, 128.6, 128.5, 126.6, 124.1, 120.7, 119.4, 113.8, 112.8, 39.5, 34.1, 31.5, 30.1, 19.8; HRMS (ESI, m/z) calculated for $C_{22}H_{19}N_2O_3$ $(M + H)^+$ 359.1390, found 359.1392.

**2-cyclohexyl-8,9,10-trihydrobenzo[3,4]isochromeno[5,6-*d*]imidazole-6,11-dione (3c)**

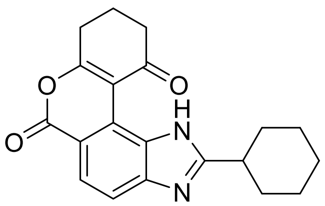

White solid, yield: 13.5 mg, 40%; Eluent: EA/DCM = 1:4; $^{1}$H NMR (400 MHz, $CDCl_3$): δ 12.58 (br, 1H), 8.18 (d, *J* = 8.6 Hz, 1H), 7.85 (d, *J* = 8.6 Hz, 1H), 3.03 (t, *J* = 6.4 Hz, 2H), 3.00 (tt, *J* = 11.7, 3.6 Hz, 1H), 2.79 (t, *J* = 6.7 Hz, 2H), 2.25 – 2.22 (m, 2H), 2.21 (quin, *J* = 6.5 Hz, 2H), 1.94 – 1.89 (m, 2H), 1.81 – 1.75 (m, 1H), 1.71 (qd, *J* = 12.4, 3.1 Hz, 2H), 1.48 (qt, *J* = 12.6, 3.1 Hz, 2H), 1.35 (qt, *J* = 12.4, 3.3 Hz, 1H); $^{13}$C{$^{1}$H} NMR (101 MHz, $CDCl_3$): δ 199.3, 171.8, 161.8, 160.5, 149.4, 128.4, 124.0, 120.7, 119.3, 113.5, 112.9, 39.6, 38.6, 31.7, 30.1, 26.1, 25.9, 19.8; HRMS (ESI, m/z) calculated for $C_{20}H_{21}N_2O_3$ $(M + H)^+$ 337.1547, found 337.1542.

**2-propyl-8,9,10-trihydrobenzo[3,4]isochromeno[5,6-*d*]imidazole-6,11-dione (3d)**

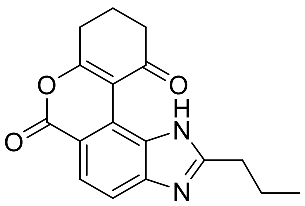

White solid, yield: 11.6 mg, 39%; Eluent: EA/DCM = 1:3; $^{1}$H NMR (400 MHz, $CDCl_3$): δ 12.51 (br, 1H), 8.18 (d, *J* = 8.6 Hz, 1H), 7.84 (d, *J* = 8.6 Hz, 1H), 3.03 (t, *J* = 6.3 Hz, 2H), 2.98 (t, *J* = 7.6 Hz, 2H), 2.78 (t, *J* = 6.7 Hz, 2H), 2.21 (quin, *J* = 6.5 Hz, 2H), 1.95 (sex, *J* = 7.4 Hz, 2H), 1.07 (t, *J* = 7.4 Hz, 3H); $^{13}$C{$^{1}$H} NMR (101 MHz, $CDCl_3$): δ 199.2, 171.8, 160.5, 157.9, 149.5, 128.6, 124.1,

120.7, 119.3, 113.6, 112.9, 39.6, 31.6, 30.1, 21.5, 19.8, 14.0, HRMS (ESI, m/z) calculated for $C_{17}H_{17}N_2O_3$ (M + H)$^+$ 297.1234, found 297.1236.

**2-methyl-8,9,10-trihydrobenzo[3,4]isochromeno[5,6-*d*]imidazole-6,11-dione (3e)**

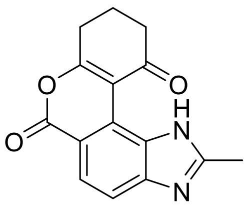


White solid, yield: 6.4 mg, 24%; Eluent: EA/DCM = 1:1; $^1$H NMR (400 MHz, $CDCl_3$): δ 12.62 (br, 1H), 8.22 (d, *J* = 8.6 Hz, 1H), 7.87 (d, *J* = 8.6 Hz, 1H), 3.05 (t, *J* = 6.4 Hz, 2H), 2.80 (t, *J* = 6.7 Hz, 2H), 2.75 (s, 3H), 2.22 (quin, *J* = 6.5 Hz, 2H); $^{13}C\{^1H\}$ NMR (101 MHz, $CDCl_3$): δ 199.3, 172.1, 160.4, 153.7, 148.6, 128.6, 124.5, 120.3, 119.4, 114.1, 112.8, 39.6, 30.1, 19.8, 15.4; HRMS (ESI, m/z) calculated for $C_{15}H_{13}N_2O_3$ (M + H)$^+$ 269.0921, found 269.0927.

**2-(trifluoromethyl)-8,9,10-trihydrobenzo[3,4]isochromeno[5,6-*d*]imidazole-6,11-dione (3f)**

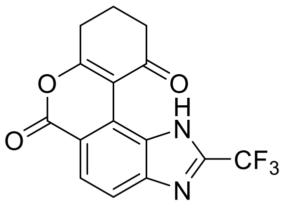


White solid, yield: 17.8 mg, 55%; Eluent: EA/DCM = 1:20; $^1$H NMR (400 MHz, $CDCl_3$): δ 13.67 (br, 1H), 8.30 (d, *J* = 8.7 Hz, 1H), 8.02 (d, *J* = 8.7 Hz, 1H), 3.08 (t, *J* = 6.3 Hz, 2H), 2.84 (t, *J* = 6.7 Hz, 2H), 2.25 (quin, *J* = 6.5 Hz, 2H); $^{13}C\{^1H\}$ NMR (101 MHz, $CDCl_3$): δ 199.4, 173.1, 159.7, 147.7, 142.9 (q, *J* = 41 Hz), 128.2, 125.2, 122.7, 121.5, 118.5 (q, *J* = 273 Hz), 116.3, 112.5, 39.4, 30.1, 19.7; $^{19}F$ NMR (376 MHz, $CD_3OD$) δ -64.7; HRMS (ESI, m/z) calculated for $C_{15}H_{10}F_3N_2O_3$ (M + H)$^+$ 323.0638, found 323.0639.

**2-(*tert*-butyl)-8,9,10-trihydrobenzo[3,4]isochromeno[5,6-*d*]imidazole-6,11-dione (3g)**

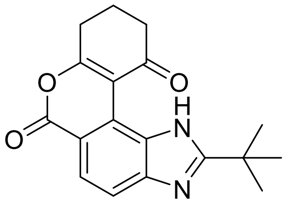


White solid, yield: 11.4 mg, 37%; Eluent: EA/DCM = 1:4; $^1$H NMR (400 MHz, $CDCl_3$): δ 12.76 (br, 1H), 8.20 (d, *J* = 8.6 Hz, 1H), 7.89 (d, *J* = 8.6 Hz, 1H), 3.04 (t, *J* = 6.4 Hz, 2H), 2.80 (t, *J* = 6.7 Hz, 2H), 2.21 (quin, *J* = 6.5 Hz, 2H), 1.56 (s, 9H); $^{13}C\{^1H\}$ NMR (151 MHz, $CDCl_3$): δ 199.4, 171.9, 165.3, 160.5, 149.3, 128.4, 124.1, 120.8, 119.5, 113.6, 112.9, 39.6, 33.8, 30.2, 29.4, 19.8; HRMS (ESI, m/z) calculated for $C_{18}H_{19}N_2O_3$ (M + H)$^+$ 311.1390, found 311.1398.

**2-mesityl-8,9,10-trihydrobenzo[3,4]isochromeno[5,6-*d*]imidazole-6,11-dione (3h)**

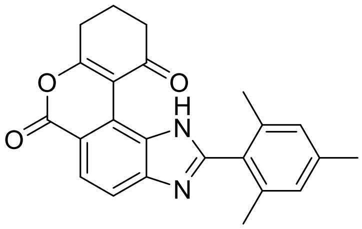


White solid, yield: 14.4 mg, 39%; Eluent: EA/DCM = 1:8; $^1$H NMR (400 MHz, $CDCl_3$): δ 12.59 (br, 1H), 8.28 (d, *J* = 8.6 Hz, 1H), 8.00 (d, *J* = 8.6 Hz, 1H), 6.99 (s, 2H), 3.06 (t, *J* = 6.3 Hz, 2H), 2.77 (t, *J* = 6.7 Hz, 2H), 2.36 (s, 3H), 2.22 (quin, *J* = 6.6 Hz, 2H), 2.21 (s, 6H); $^{13}C\{^1H\}$

NMR (101 MHz, $CDCl_3$): δ 199.1, 171.9, 160.5, 154.3, 149.1, 139.9, 137.9, 128.7, 128.6, 127.3, 124.2, 121.3, 119.9, 114.2, 113.0, 39.6, 30.1, 21.4, 20.3, 19.8; HRMS (ESI, m/z) calculated for $C_{23}H_{21}N_2O_3$ $(M + H)^+$ 373.1547, found 373.1541.

**2-(2,6-dichlorophenyl)-8,9,10-trihydrobenzo[3,4]isochromeno[5,6-*d*]imidazole-6,11-dione (3i)**

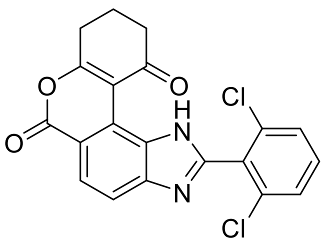


White solid, yield: 18.8 mg, 47%; Eluent: EA/DCM = 1:6; $^1H$ NMR (400 MHz, $CDCl_3$): δ 12.99 (br, 1H), 8.31 (d, $J$ = 8.6 Hz, 1H), 8.05 (d, $J$ = 8.6 Hz, 1H), 7.50 – 7.47 (m, 2H), 7.44 – 7.40 (m, 1H), 3.07 (t, $J$ = 6.4 Hz, 2H), 2.80 (t, $J$ = 6.7 Hz, 2H), 2.23 (quin, $J$ = 6.5 Hz, 2H); $^{13}C\{^1H\}$ NMR (101 MHz, $CDCl_3$): δ 199.3, 172.2, 160.4, 149.1, 148.9, 136.2, 132.0, 129.6, 128.8, 128.5, 124.4, 121.9, 120.4, 114.9, 113.0, 39.6, 30.2, 19.8; HRMS (ESI, m/z) calculated for $C_{20}H_{13}Cl_2N_2O_3$ $(M + H)^+$ 399.0298, found 399.0298.

**2-chloro-8,9,10-trihydrobenzo[3,4]isochromeno[5,6-*d*]imidazole-6,11-dione (3j)**

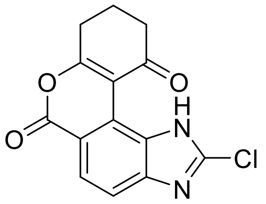


White solid, yield: 11.0 mg, 38%; Eluent: EA/DCM = 1:8; $^1H$ NMR (400 MHz, $CDCl_3$): δ 12.85 (br, 1H), 8.24 (d, $J$ = 8.6 Hz, 1H), 7.84 (d, $J$ = 8.6 Hz, 1H), 3.06 (t, $J$ = 6.4 Hz, 2H), 2.81 (t, $J$ = 6.7 Hz, 2H), 2.23 (quin, $J$ = 6.6 Hz, 2H); $^{13}C\{^1H\}$ NMR (151 MHz, $CDCl_3$): δ 199.2, 172.4, 160.0, 148.5, 141.7, 129.0, 124.7, 120.8, 119.4, 114.7, 112.5, 39.5, 30.1, 19.8; HRMS (ESI, m/z) calculated for $C_{14}H_{10}ClN_2O_3$ $(M + H)^+$ 289.0375, found 289.0378.

**2-bromo-8,9,10-trihydrobenzo[3,4]isochromeno[5,6-*d*]imidazole-6,11-dione (3k)**

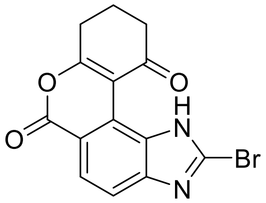


White solid, yield: 12.2 mg, 37%; Eluent: EA/DCM = 1:9; $^1H$ NMR (400 MHz, $CDCl_3$): δ 12.93 (br, 1H), 8.22 (d, $J$ = 8.6 Hz, 1H), 7.85 (d, $J$ = 8.6 Hz, 1H), 3.05 (t, $J$ = 6.3 Hz, 2H), 2.81 (t, $J$ = 6.7 Hz, 2H), 2.23 (quin, $J$ = 6.5 Hz, 2H); $^{13}C\{^1H\}$ NMR (101 MHz, $CDCl_3$): δ 199.2, 172.4, 160.0, 149.4, 130.0, 129.4, 124.6, 120.7, 119.2, 114.6, 112.5, 39.5, 30.1, 19.8; HRMS (ESI, m/z) calculated for $C_{14}H_{10}BrN_2O_3$ $(M + H)^+$ 332.9870, found 332.9870.

**2-(methylthio)-8,9,10-trihydrobenzo[3,4]isochromeno[5,6-*d*]imidazole-6,11-dione (3l)**

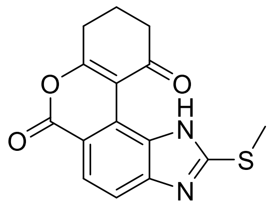

White solid, yield: 11.3 mg, 38%; Eluent: EA/DCM = 1:7; $^{1}H$ NMR (400 MHz, $CDCl_3$): δ 12.57 (br, 1H), 8.18 (d, *J* = 8.6 Hz, 1H), 7.79 (d, *J* = 8.6 Hz, 1H), 3.03 (t, *J* = 6.3 Hz, 2H), 2.82 (s, 3H), 2.78 (t, *J* = 6.7 Hz, 2H), 2.21 (quin, *J* = 6.5 Hz, 2H); $^{13}C\{^{1}H\}$ NMR (151 MHz, $CDCl_3$): δ 199.2, 171.8, 160.4, 155.3, 150.0, 130.0, 124.4, 119.5, 118.3, 113.3, 112.8, 39.5, 30.1, 19.8, 14.4; HRMS (ESI, m/z) calculated for $C_{15}H_{13}N_2O_3S$ $(M + H)^+$ 301.0642, found 301.0645.

**2-(phenylthio)-8,9,10-trihydrobenzo[3,4]isochromeno[5,6-*d*]imidazole-6,11-dione (3m)**

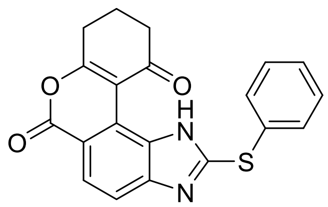

White solid, yield: 16.8 mg, 46%; Eluent: EA/DCM = 1:9; $^{1}H$ NMR (400 MHz, $CDCl_3$): δ 12.37 (br, 1H), 8.15 (d, *J* = 8.6 Hz, 1H), 7.77 (d, *J* = 8.6 Hz, 1H), 7.75 – 7.72 (m, 2H), 7.54 – 7.50 (m, 3H), 2.97 (t, *J* = 6.4 Hz, 2H), 2.64 (t, *J* = 6.7 Hz, 2H), 2.13 (quin, *J* = 6.5 Hz, 2H); $^{13}C\{^{1}H\}$ NMR (151 MHz, $CDCl_3$): δ 198.7, 171.7, 160.3, 153.9, 150.3, 134.7, 130.3, 130.2, 129.4, 128.3, 124.4, 120.1, 118.7, 113.6, 112.6, 39.4, 30.0, 19.7; HRMS (ESI, m/z) calculated for $C_{20}H_{15}N_2O_3S$ $(M + H)^+$ 363.0798, found 363.0799.

**2-ethoxy-8,9,10-trihydrobenzo[3,4]isochromeno[5,6-*d*]imidazole-6,11-dione (3n)**

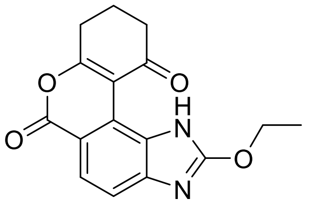

White solid, yield: 13.0 mg, 44%; Eluent: EA/DCM = 1:7; $^{1}H$ NMR (400 MHz, $CDCl_3$): δ 11.64 (br, 1H), 8.14 (d, *J* = 8.5 Hz, 1H), 7.66 (d, *J* = 8.5 Hz, 1H), 4.62 (q, *J* = 7.1 Hz, 2H), 3.00 (t, *J* = 6.4 Hz, 2H), 2.75 (t, *J* = 6.7 Hz, 2H), 2.19 (quin, *J* = 6.5 Hz, 2H), 1.50 (t, *J* = 7.1 Hz, 3H); $^{13}C\{^{1}H\}$ NMR (151 MHz, $CDCl_3$): δ 198.9, 171.2, 160.5, 159.5, 148.1, 126.7, 124.3, 119.1, 117.7, 112.7, 112.7, 66.5, 39.5, 30.0, 19.8, 14.8; HRMS (ESI, m/z) calculated for $C_{16}H_{15}N_2O_4$ $(M + H)^+$ 299.1026, found 299.1026.

**2-benzoyl-8,9,10-trihydrobenzo[3,4]isochromeno[5,6-*d*]imidazole-6,11-dione (3o)**

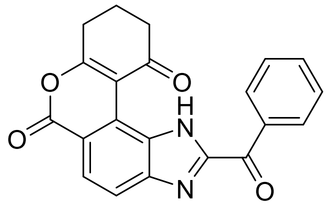

White solid, yield: 6.3 mg, 18%; Eluent: EA/DCM = 1:12; $^{1}H$ NMR (600 MHz, $CDCl_3$): δ 13.53 (br, 1H), 8.70 (d, *J* = 7.8 Hz, 1H), 8.28 (d, *J* = 8.6 Hz, 1H), 8.07 (d, *J* = 8.7 Hz, 1H), 7.68 (t, *J* = 7.4 Hz, 1H), 7.58 (m, *J* = 7.8 Hz, 2H), 3.07 (t, *J* = 6.3 Hz, 2H), 2.84 (t, *J* = 6.7 Hz, 2H), 2.25 (quin, *J* = 6.5 Hz, 2H); $^{13}C\{^{1}H\}$ NMR (151 MHz, $CDCl_3$): δ 198.5, 183.3, 172.1, 160.1, 149.6, 149.0, 135.5, 134.2, 131.4, 128.7, 128.6, 124.8, 123.4, 121.7, 116.7, 112.9, 39.4, 30.1, 19.8; HRMS (ESI, m/z) calculated for $C_{21}H_{15}N_2O_4$ $(M + H)^+$ 359.1026, found 359.1029.

**2-(1-hydroxyethyl)-8,9,10-trihydrobenzo[3,4]isochromeno[5,6-*d*]imidazole-6,11-dione (3p)**

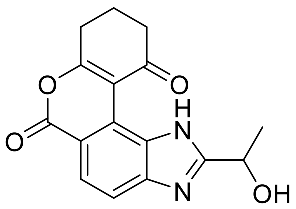

White solid, yield: 3.0 mg, 10%; Eluent: EA/DCM = 3:1; $^1$H NMR (600 MHz, $(CD_3)_2SO$): δ 12.53 (s, 1H), 8.03 (d, *J* = 8.5 Hz, 1H), 7.82 (d, *J* = 8.5 Hz, 1H), 6.13 (d, *J* = 5.2 Hz, 1H), 5.05 (quin, *J* = 6.3 Hz, 1H), 3.01 (t, *J* = 6.3 Hz, 2H), 2.75 (t, *J* = 6.7 Hz, 2H), 2.11 (quin, *J* = 6.4 Hz, 2H), 1.55 (d, *J* = 6.7 Hz, 3H); $^{13}C\{^1H\}$ NMR (151 MHz, $(CD_3)_2SO$): δ 198.9, 172.2, 161.3, 159.9, 148.9, 127.3, 122.9, 120.1, 119.8, 113.0, 111.8, 63.4, 38.6, 29.2, 22.8, 19.1; HRMS (ESI, m/z) calculated for $C_{16}H_{15}N_2O_4$ $(M + H)^+$ 299.1027, found 299.1027.

**8,9,10-trihydrobenzo[3,4]isochromeno[5,6-*d*]imidazole-6,11-dione (3q)**

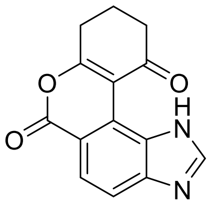

White solid, yield: 7.7 mg, 30%; Eluent: EA/DCM = 2:1; $^1$H NMR (600 MHz, $CDCl_3$): δ 12.91 (br, 1H), 8.26 (s, 1H), 8.25 (d, *J* = 8.6 Hz, 1H), 7.98 (d, *J* = 8.6 Hz, 1H), 3.06 (t, *J* = 6.4 Hz, 2H), 2.81 (t, *J* = 6.7 Hz, 2H), 2.23 (quin, *J* = 6.5 Hz, 2H); $^{13}C\{^1H\}$ NMR (151 MHz, $CDCl_3$): δ 199.2, 172.0, 160.4, 148.8, 143.1, 128.0, 124.2, 121.8, 120.4, 114.7, 112.9, 39.6, 30.1, 19.8; HRMS (ESI, m/z) calculated for $C_{14}H_{11}N_2O_3$ $(M + H)^+$ 255.0764, found 255.0766.

**2-amino-8,9,10-trihydrobenzo[3,4]isochromeno[5,6-*d*]imidazole-6,11-dione (3r)**

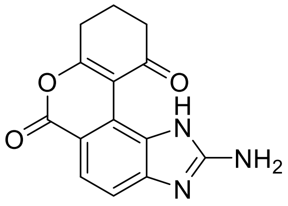

White solid, yield: 2.9 mg, 11%; Eluent: EA; $^1$H NMR (600 MHz, $(CD_3)_2SO$): δ 11.10 (s, 1H), 7.87 (d, *J* = 8.5 Hz, 1H), 7.35 (d, *J* = 8.4 Hz, 1H), 6.93 (s, 2H), 2.94 (t, *J* = 6.2 Hz, 2H), 2.70 (t, *J* = 6.7 Hz, 2H), 2.08 (quin, *J* = 6.4 Hz, 2H); $^{13}C\{^1H\}$ NMR (151 MHz, $(CD_3)_2SO$): δ 198.5, 170.8, 160.2, 157.3, 151.1, 126.7, 123.2, 116.0, 115.7, 111.8, 109.3, 38.6, 29.0, 19.2; HRMS (ESI, m/z) calculated for $C_{14}H_{12}N_3O_3$ $(M + H)^+$ 270.0874, found 270.0877.

**2-(furan-2-yl)-8,9,10-trihydrobenzo[3,4]isochromeno[5,6-*d*]imidazole-6,11-dione (3s)**

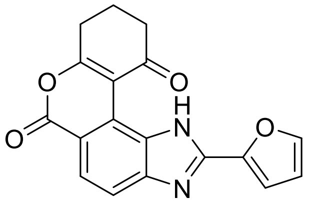

White solid, yield: 4.2 mg, 13%; Eluent: EA/DCM = 1:3; $^1$H NMR (400 MHz, $CDCl_3$): δ 13.10 (br, 1H), 8.24 (d, *J* = 8.6 Hz, 1H), 7.91 (d, *J* = 8.6 Hz, 1H), 7.66 (dd, *J* = 1.8, 0.7 Hz, 1H), 7.31 (dd, *J* = 3.5, 0.7 Hz, 1H), 6.64 (dd, *J* = 3.5, 1.8 Hz, 1H), 3.06 (t, *J* = 6.4 Hz, 2H), 2.83 (t, *J* = 6.7 Hz, 2H), 2.24 (quin, *J* = 6.6 Hz, 2H); $^{13}C\{^1H\}$ NMR (101 MHz, $CDCl_3$): δ 199.2, 172.1, 160.3, 149.9, 146.1, 145.1, 114.9, 128.5, 124.8, 121.0, 119.8, 114.2, 112.9, 112.7, 112.2, 39.5, 30.1, 19.8; HRMS (ESI, m/z)

calculated for $C_{18}H_{13}N_2O_4$ $(M + H)^+$ 321.0870, found 321.0894.

**(*E*)-2-styryl-8,9,10-trihydrobenzo[3,4]isochromeno[5,6-*d*]imidazole-6,11-dione (3t)**

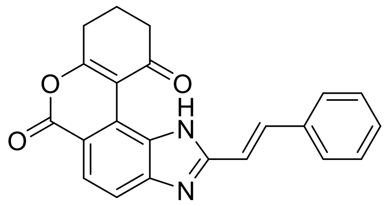


White solid, yield: 2.3 mg, 6%; Eluent: EA/DCM = 1:4; $^1H$ NMR (600 MHz, $CDCl_3$): δ 12.93 (br, 1H), 8.24 (d, *J* = 8.6 Hz, 1H), 7.90 (d, *J* = 8.6 Hz, 1H), 7.78 (d, *J* = 16.4 Hz, 1H), 7.63 (d, *J* = 7.4 Hz, 2H), 7.43 (t, *J* = 7.4 Hz, 2H), 7.38 (t, *J* = 7.3 Hz, 1H), 7.20 (d, *J* = 16.4 Hz, 1H), 3.06 (t, *J* = 6.4 Hz, 2H), 2.83 (t, *J* = 6.7 Hz, 2H), 2.24 (quin, *J* = 6.5 Hz, 2H); $^{13}C\{^1H\}$ NMR (151 MHz, $CDCl_3$): δ 199.4, 172.1, 160.4, 153.2, 149.9, 137.6, 135.6, 129.7, 129.1, 129.0, 127.6, 124.7, 120.8, 119.5, 116.1, 114.1, 113.0, 39.6, 30.2, 19.8; HRMS (ESI, m/z) calculated for $C_{22}H_{17}N_2O_3$ $(M + H)^+$ 357.1234, found 357.1235.

**(*E*)-2-(prop-1-en-1-yl)-8,9,10-trihydrobenzo[3,4]isochromeno[5,6-*d*]imidazole-6,11-dione (3u)**

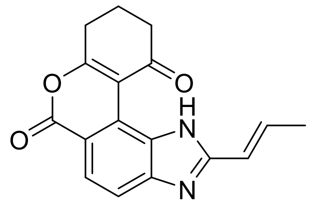


White solid, yield: 2.1 mg, 7%; Eluent: EA/DCM = 1:5; $^1H$ NMR (600 MHz, $CDCl_3$): δ 12.67 (br, 1H), 8.20 (d, *J* = 8.6 Hz, 1H), 7.83 (d, *J* = 8.5 Hz, 1H), 6.94 (dq, *J* = 15.9, 6.8 Hz, 1H), 6.56 (dq, *J* = 15.9, 1.8 Hz, 1H), 3.03 (t, *J* = 6.4 Hz, 2H), 2.79 (t, *J* = 6.7 Hz, 2H), 2.21 (quin, *J* = 6.5 Hz, 2H), 2.04 (dd, *J* = 6.8, 1.6 Hz, 3H); $^{13}C\{^1H\}$ NMR (151 MHz, $CDCl_3$): δ 199.5, 172.7, 160.6, 153.5, 150.1, 136.7, 128.9, 124.6, 121.0, 120.7, 119.5, 114.1, 113.1, 39.8, 30.3, 20.0, 19.1; HRMS (ESI, m/z) calculated for $C_{17}H_{15}N_2O_3$ $(M + H)^+$ 295.1077, found 295.1083.

**(*Z*)-2-(prop-1-en-1-yl)-8,9,10-trihydrobenzo[3,4]isochromeno[5,6-*d*]imidazole-6,11-dione (3v)**

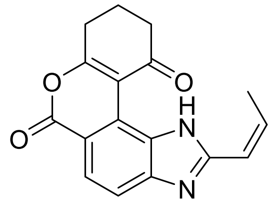


White solid, yield: 8.6 mg, 29%; Eluent: EA/DCM = 1:6; $^1H$ NMR (400 MHz, $CDCl_3$): δ 12.80 (br, 1H), 8.20 (d, *J* = 8.6 Hz, 1H), 7.89 (d, *J* = 8.6 Hz, 1H), 6.55 (dq, *J* = 11.8, 1.8 Hz, 1H), 6.32 (dq, *J* = 11.7, 7.3 Hz, 1H), 3.04 (t, *J* = 6.3 Hz, 2H), 2.79 (t, *J* = 6.7 Hz, 2H), 2.36 (dd, *J* = 7.3, 1.7 Hz, 3H), 2.21 (quin, *J* = 6.6 Hz, 2H); $^{13}C\{^1H\}$ NMR (151 MHz, $CDCl_3$): δ 199.2, 171.9, 160.4, 152.8, 149.6, 136.4, 128.6, 124.3, 121.0, 119.5, 119.1, 114.0, 113.0, 39.6, 30.1, 19.8, 15.8; HRMS (ESI, m/z) calculated for $C_{17}H_{15}N_2O_3$ $(M + H)^+$ 295.1077, found 295.1085.

**2-benzyl-8,9,10-trihydrobenzo[3,4]isochromeno[5,6-*d*]oxazole-6,11-dione (3x)**

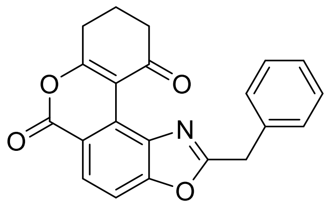

White solid, yield: 9.8 mg, 28%; Eluent: EA/DCM = 1:15; $^1$H NMR (600 MHz, $CDCl_3$): δ 8.28 (d, *J* = 8.7 Hz, 1H), 7.61 (d, *J* = 8.6 Hz, 1H), 7.39 (d, *J* = 7.1 Hz, 2H), 7.34 (t, *J* = 7.6 Hz, 2H), 7.28 (t, *J* = 7.4 Hz, 1H), 4.38 (s, 2H), 2.93 (t, *J* = 6.3 Hz, 2H), 2.80 (t, *J* = 6.8 Hz, 2H), 2.23 (quin, *J* = 6.5 Hz, 2H); $^{13}C\{^1H\}$ NMR (151 MHz, $CDCl_3$): δ 193.1, 168.7, 165.0, 160.5, 156.0, 136.9, 134.5, 129.2, 129.0, 127.5, 127.4, 127.1, 117.4, 113.7, 112.0, 38.1, 35.5, 28.9, 20.0; HRMS (ESI, m/z) calculated for $C_{21}H_{16}NO_4$ $(M + H)^+$ 346.1074, found 346.1074.

**2-phenyl-8,9,10-trihydrobenzo[3,4]isochromeno[5,6-*d*]oxazole-6,11-dione (3y)**

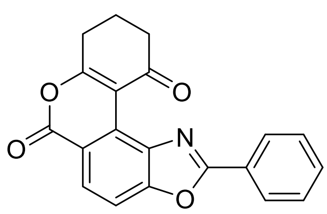

White solid, yield: 12.9 mg, 39%; Eluent: EA/DCM = 1:20; $^1$H NMR (400 MHz, $CDCl_3$): δ 8.33 (d, *J* = 8.6 Hz, 1H), 8.30 – 8.28 (m, 2H), 7.74 (d, *J* = 8.6 Hz, 1H), 7.57 – 7.51 (m, 3H), 2.94 (t, *J* = 6.3 Hz, 2H), 2.83 (t, *J* = 6.7 Hz, 2H), 2.25 (quin, *J* = 6.5 Hz, 2H); $^{13}C\{^1H\}$ NMR (151 MHz, $CDCl_3$): δ 192.7, 168.5, 163.1, 160.5, 155.7, 137.7, 132.2, 129.1, 128.2, 127.6, 127.2, 126.7, 117.6, 113.8, 112.0, 38.1, 28.9, 20.0; HRMS (ESI, m/z) calculated for $C_{20}H_{14}NO_4$ $(M + H)^+$ 332.0918, found 332.0929.

**2-benzyl-9-methyl-8,9,10-trihydrobenzo[3,4]isochromeno[5,6-*d*]imidazole-6,11-dione (3ab)**

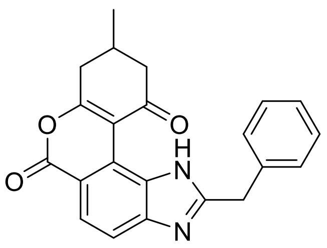

White solid, yield: 20.1 mg, 56%; Eluent: EA/DCM = 1:4; $^1$H NMR (400 MHz, $CDCl_3$): δ 12.51 (br, 1H), 8.18 (d, *J* = 8.6 Hz, 1H), 7.86 (d, *J* = 8.6 Hz, 1H), 7.40 – 7.36 (m, 4H), 7.34 – 7.29 (m, 1H), 4.36 (s, 2H), 3.03 – 2.98 (m, 1H), 2.81 – 2.67 (m, 2H), 2.45 – 2.37 (m, 2H), 1.18 (d, *J* = 5.6 Hz, 3H); $^{13}C\{^1H\}$ NMR (101 MHz, $CDCl_3$): δ 198.9, 171.1, 160.5, 156.2, 149.4, 136.0, 129.2, 129.1, 128.9, 127.5, 124.1, 120.8, 119.4, 113.8, 112.4, 47.6, 37.9, 36.3, 27.4, 20.7; HRMS (ESI, m/z) calculated for $C_{22}H_{19}N_2O_3$ $(M + H)^+$ 359.1390, found 359.1390.

**2-benzyl-9,9-dimethyl-8,9,10-trihydrobenzo[3,4]isochromeno[5,6-*d*]imidazole-6,11-dione (3ac)**

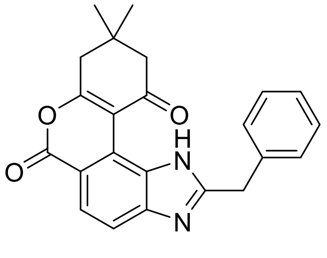

White solid, yield: 20.3 mg, 55%; Eluent: EA/DCM = 1:4; $^1$H NMR (400 MHz, $CDCl_3$): δ 12.54 (br, 1H), 8.20 (d, *J* = 8.6 Hz, 1H), 7.87 (d, *J* = 8.6 Hz, 1H), 7.41 – 7.36 (m, 4H), 7.33 – 7.29 (m, 1H), 4.36 (s, 2H), 2.87 (s, 2H), 2.58 (s, 2H), 1.18 (s, 6H); $^{13}C\{^1H\}$ NMR (101 MHz, $CDCl_3$): δ 198.9, 170.2, 160.7, 156.2, 149.5, 136.0, 129.2, 129.1, 128.9, 127.5, 124.1, 120.9, 119.3, 113.7, 111.8, 53.3, 43.6, 36.3, 31.7, 28.1; HRMS (ESI, m/z) calculated for $C_{23}H_{21}N_2O_3$

$(M + H)^+$ 373.1547, found 373.1548.

**2-benzyl-9-phenyl-8,9,10-trihydrobenzo[3,4]isochromeno[5,6-*d*]imidazole-6,11-dione (3ad)**

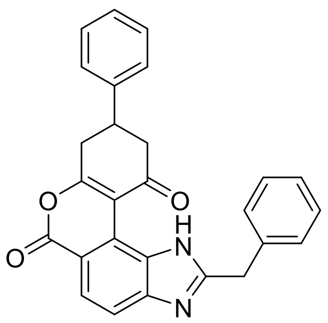


White solid, yield: 21.1 mg, 50%; Eluent: EA/DCM = 1:5; $^1$H NMR (400 MHz, $CDCl_3$): δ 12.48 (br, 1H), 8.20 (d, *J* = 8.6 Hz, 1H), 7.88 (d, *J* = 8.6 Hz, 1H), 7.42 – 7.37 (m, 6H), 7.34 – 7.27 (m, 4H), 4.37 (s, 2H), 3.59 – 3.51 (m, 1H), 3.22 (d, *J* = 8.3 Hz, 2H), 3.02 (dd, *J* = 16.4, 4.3 Hz, 1H), 2.93 (dd, *J* = 16.4, 13.4 Hz, 1H); $^{13}$C{$^1$H} NMR (101 MHz, $CDCl_3$): δ 198.1, 170.8, 160.4, 156.4, 149.5, 140.9, 135.9, 129.2, 129.2, 129.1, 128.9, 127.8, 127.5, 126.6, 124.2, 121.0, 119.2, 113.8, 112.6, 46.3, 37.6, 37.3, 36.3; HRMS (ESI, m/z) calculated for $C_{27}H_{21}N_2O_3$ $(M + H)^+$ 421.1547, found 421.1548.

**2-benzyl-8,10-dihydropyrano[3',4':3,4]isochromeno[5,6-*d*]imidazole-6,11-dione (3ae)**

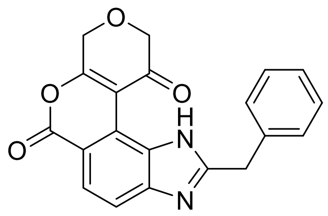


White solid, yield: 13.1 mg, 38%; Eluent: EA/DCM = 1:4; $^1$H NMR (400 MHz, $CDCl_3$): δ 12.22 (br, 1H), 8.19 (d, *J* = 8.6 Hz, 1H), 7.91 (d, *J* = 8.6 Hz, 1H), 7.43 – 7.40 (m, 4H), 7.36 – 7.31 (m, 1H), 4.77 (s, 2H), 4.38 (s, 4H); $^{13}$C{$^1$H} NMR (101 MHz, $CDCl_3$): δ 194.0, 168.5, 159.2, 157.0, 149.5, 135.7, 129.3, 129.1, 128.7, 127.7, 124.4, 121.4, 117.8, 113.6, 110.5, 72.9, 65.6, 36.3; HRMS (ESI, m/z) calculated for $C_{20}H_{15}N_2O_4$ $(M + H)^+$ 347.1026, found 347.1028.

**2-benzyl-8,9-dihydrocyclopenta[3,4]isochromeno[5,6-*d*]imidazole-6,10-dione (3af)**

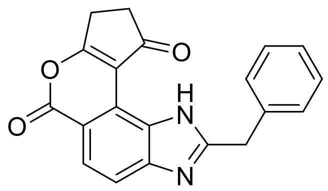


White solid, yield: 14.6 mg, 44%; Eluent: EA/DCM = 1:4; $^1$H NMR (400 MHz, $CDCl_3$): δ 11.85 (br, 1H), 8.10 (d, *J* = 8.7 Hz, 1H), 7.84 (d, *J* = 8.7 Hz, 1H), 7.44 – 7.39 (m, 4H), 7.37 – 7.31 (m, 1H), 4.39 (s, 2H), 3.13 – 3.10 (m, 2H), 2.80 – 2.78 (m, 2H); $^{13}$C{$^1$H} NMR (101 MHz, $CDCl_3$): δ 202.4, 182.1, 160.9, 157.4, 148.9, 135.6, 129.3, 129.2, 128.8, 127.7, 124.3, 120.7, 117.1, 114.8, 111.4, 36.3, 34.2, 26.9; HRMS (ESI, m/z) calculated for $C_{20}H_{15}N_2O_3$ $(M + H)^+$ 331.1077, found 331.1077.

**2-benzyl-8,9,10,11-tetrahydrocyclohepta[3,4]isochromeno[5,6-*d*]imidazole-6,12-dione (3ag)**

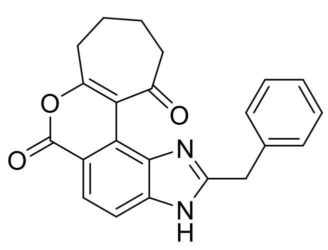

White solid, yield: 2.0 mg, 6%; Eluent: EA/DCM = 1:4; $^{1}H$ NMR (400 MHz, $CDCl_3$): δ 10.98 (br, 1H), 8.22 (d, $J$ = 8.6 Hz, 1H), 7.87 (d, $J$ = 8.6 Hz, 1H), 7.42 – 7.37 (m, 4H), 7.35 – 7.30 (m, 1H), 4.35 (s, 2H), 3.00 (t, $J$ = 6.1 Hz, 2H), 2.82 (t, $J$ = 6.2 Hz, 2H), 1.98 – 1.94 (m, 4H); $^{13}C\{^{1}H\}$ NMR (151 MHz, $CDCl_3$): δ 206.9, 167.3, 161.3, 156.4, 149.5, 135.9, 129.3, 129.1, 128.2, 127.6, 124.1, 120.7, 120.4, 115.4, 113.9, 43.1, 36.2, 32.8, 22.8, 22.2; HRMS (ESI, m/z) calculated for $C_{22}H_{19}N_2O_3$ $(M + H)^+$ 359.1390, found 359.1392.

**4-benzyl-6*H*-chromeno[4',3':3,4]isochromeno[5,6-*d*]imidazole-6,13-dione (3aj)**

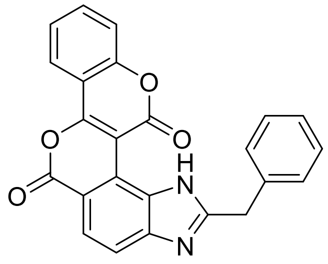

White solid, yield: 18.1 mg, 46%; Eluent: EA/DCM = 1:7; $^{1}H$ NMR (400 MHz, $CDCl_3$): δ 12.61 (br, 1H), 8.28 (d, $J$ = 8.6 Hz, 1H), 8.19 (dd, $J$ = 8.0, 1.4 Hz, 1H), 7.92 (d, $J$ = 8.6 Hz, 1H), 7.68 (td, $J$ = 7.8, 1.5 Hz, 1H), 7.44 (td, $J$ = 7.7, 0.9 Hz, 1H), 7.42 – 7.39 (m, 5H), 7.36 – 7.31 (m, 1H), 4.38 (s, 2H); $^{13}C\{^{1}H\}$ NMR (101 MHz, $CDCl_3$): δ 161.5, 159.1, 158.7, 157.0, 152.4, 149.7, 135.6, 134.2, 129.4, 129.2, 129.0, 127.7, 125.7, 124.8, 124.2, 121.8, 118.6, 116.7, 114.2, 113.6, 101.5, 36.3; HRMS (ESI, m/z) calculated for $C_{24}H_{15}N_2O_4$ $(M + H)^+$ 395.1026, found 395.1026.

**4-benzyl-10-chloro-6*H*-chromeno[4',3':3,4]isochromeno[5,6-*d*]imidazole-6,13-dione (3ak)**

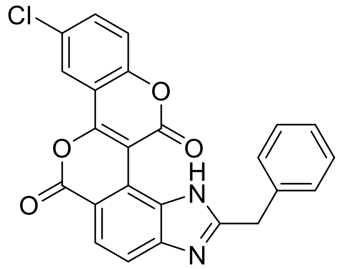

White solid, yield: 20.1 mg, 47%; Eluent: EA/DCM = 1:6; $^{1}H$ NMR (400 MHz, $CDCl_3$): δ 12.60 (br, 1H), 8.34 (d, $J$ = 8.6 Hz, 1H), 8.22 (d, $J$ = 2.4 Hz, 1H), 8.01 (d, $J$ = 8.6 Hz, 1H), 7.64 (dd, $J$ = 8.9, 2.5 Hz, 1H), 7.42 – 7.41 (m, 4H), 7.39 (d, $J$ = 8.9 Hz, 1H), 7.37 – 7.32 (m, 1H), 4.43 (s, 2H); $^{13}C\{^{1}H\}$ NMR (151 MHz, $CDCl_3$): δ 161.2, 158.7, 157.7, 157.2, 150.7, 149.7 (br), 135.4, 134.3, 131.7, 129.4, 129.2, 129.1, 127.8, 125.1, 123.7, 122.2, 118.3, 118.2, 114.8, 114.6, 102.3, 36.3; HRMS (ESI, m/z) calculated for $C_{24}H_{14}ClN_2O_4$ $(M + H)^+$ 429.0637, found 429.0637.

**9-benzyl-2,3,4-trihydrobenzo[3,4]isochromeno[6,7-*d*]imidazole-1,6-dione (4a)**

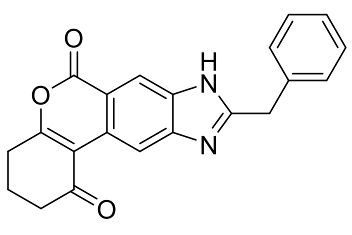

White solid, yield: 11.8 mg, 34%; Eluent: EA/DCM = 1:2; $^{1}H$ NMR (400 MHz, $CD_3OD$): δ 9.17 (s, 1H), 8.38 (s, 1H), 7.37 – 7.32 (m, 4H), 7.28 – 7.24 (m, 1H), 4.30 (s, 2H), 2.91 (t, $J$ = 6.3 Hz, 2H), 2.63 (t, $J$ = 6.7 Hz, 2H), 2.14 (quin, $J$ = 6.5 Hz, 2H); $^{13}C\{^{1}H\}$ NMR (101 MHz,

$CD_3OD$): δ 199.4, 169.8, 162.6, 160.5, 137.4, 129.9, 129.9, 129.7, 128.3, 118.0 (br), 116.1, 112.5, 111.4 (br), 39.9, 36.3, 29.7, 21.1; HRMS (ESI, m/z) calculated for $C_{21}H_{17}N_2O_3$ $(M + H)^+$ 345.1234, found 345.1234.

**9-phenethyl-2,3,4-trihydrobenzo[3,4]isochromeno[6,7-*d*]imidazole-1,6-dione (4b)**

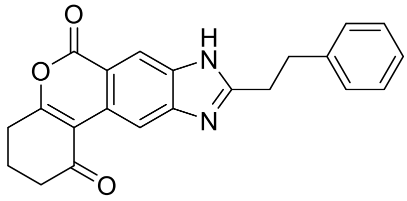


White solid, yield: 16.2 mg, 45%; Eluent: EA/DCM = 1:3; $^1H$ NMR (400 MHz, $CD_3OD$): δ 8.99 (s, 1H), 8.19 (s, 1H), 7.26 – 7.20 (m, 4H), 7.18 – 7.14 (m, 1H), 3.24 – 3.14 (m, 4H), 2.86 (t, $J$ = 6.3 Hz, 2H), 2.60 (t, $J$ = 6.7 Hz, 2H), 2.13 (quin, $J$ = 6.5 Hz, 2H); $^{13}C\{^1H\}$ NMR (101 MHz, $CDCl_3$): δ 198.1, 168.2, 161.5, 159.0, 140.2, 128.9, 128.4, 128.4, 126.8, 115.0, 112.0, 39.3, 34.0, 31.5, 29.1, 20.2; HRMS (ESI, m/z) calculated for $C_{22}H_{19}N_2O_3$ $(M + H)^+$ 359.1390, found 359.1391.

**9-cyclohexyl-2,3,4-trihydrobenzo[3,4]isochromeno[6,7-*d*]imidazole-1,6-dione (4c)**

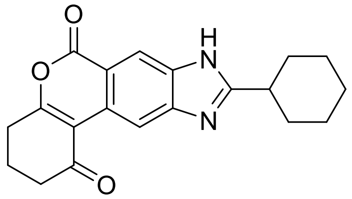


White solid, yield: 15.6 mg, 46%; Eluent: EA/DCM = 1:3; $^1H$ NMR (400 MHz, $CD_3OD$): δ 9.15 (s, 1H), 8.35 (s, 1H), 2.97 (tt, $J$ = 11.6, 3.5 Hz, 1H), 2.94 (t, $J$ = 6.3 Hz, 2H), 2.65 (t, $J$ = 6.7 Hz, 2H), 2.17 (quin, $J$ = 6.6 Hz, 2H), 2.14 – 2.11 (m, 2H), 1.95 – 1.89 (m, 2H), 1.83 – 1.79 (m, 1H), 1.70 (qd, $J$ = 12.4, 3.0 Hz, 2H), 1.50 (qt, $J$ = 12.6, 3.1 Hz, 2H), 1.38 (qt, $J$ = 12.4, 3.2 Hz, 1H); $^{13}C\{^1H\}$ NMR (151 MHz, $(CD_3)_2SO$): δ 197.4, 168.1, 163.6 (br), 160.6, 142.7 (br), 140.0 (br), 127.4, 119.2 (br), 113.5, 110.8, 106.9 (br), 38.5, 37.7, 30.9, 28.2, 25.4, 25.3, 19.5; HRMS (ESI, m/z) calculated for $C_{20}H_{21}N_2O_3$ $(M + H)^+$ 337.1547, found 337.1542.

**9-propyl-2,3,4-trihydrobenzo[3,4]isochromeno[6,7-*d*]imidazole-1,6-dione (4d)**

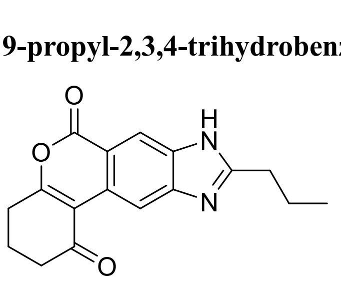


White solid, yield: 12.5 mg, 42%; Eluent: EA/DCM = 1:1; $^1H$ NMR (400 MHz, $CD_3OD$): δ 9.03 (s, 1H), 8.22 (s, 1H), 2.92 (t, $J$ = 7.6 Hz, 2H), 2.89 (t, $J$ = 6.3 Hz, 2H), 2.62 (t, $J$ = 6.7 Hz, 2H), 2.15 (quin, $J$ = 6.5 Hz, 2H), 1.91 (sex, $J$ = 7.5 Hz, 2H), 1.05 (t, $J$ = 7.4 Hz, 3H); $^{13}C\{^1H\}$ NMR (101 MHz, $CD_3OD$): δ 199.4, 169.6, 162.5, 162.1, 129.4, 115.7, 112.5, 39.9, 31.9, 29.7, 22.3, 21.1, 14.1; HRMS (ESI, m/z) calculated for $C_{17}H_{17}N_2O_3$ $(M + H)^+$ 297.1234, found 297.1234.

**9-methyl-2,3,4-trihydrobenzo[3,4]isochromeno[6,7-*d*]imidazole-1,6-dione (4e)**

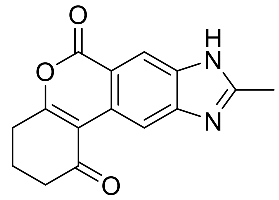

White solid, yield: 10.0 mg, 37%; Eluent: EA/DCM = 3:1; $^1$H NMR (400 MHz, $(CD_3)_2SO$): δ 12.77 (br, 1H), 9.07 (s, 1H), 8.27 (s, 1H), 2.91 (t, $J$ = 6.2 Hz, 2H), 2.60 (t, $J$ = 6.7 Hz, 2H), 2.57 (s, 3H), 2.07 (quin, $J$ = 6.5 Hz, 2H); $^{13}C\{^1H\}$ NMR (101 MHz, $(CD_3)_2SO$): δ 197.5, 168.2, 160.6, 127.4, 113.5, 110.8, 38.6, 28.3, 19.6, 14.9; HRMS (ESI, m/z) calculated for $C_{15}H_{13}N_2O_3$ $(M + H)^+$ 269.0921, found 269.0921.

**9-(trifluoromethyl)-2,3,4-trihydrobenzo[3,4]isochromeno[6,7-*d*]imidazole-1,6-dione (4f)**

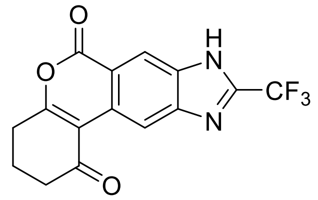

White solid, yield: 4.1 mg, 13%; Eluent: EA/DCM = 1:10; $^1$H NMR (400 MHz, $CD_3OD$): δ 9.40 (s, 1H), 8.64 (s, 1H), 2.98 (t, $J$ = 6.3 Hz, 2H), 2.69 (t, $J$ = 6.7 Hz, 2H), 2.19 (quin, $J$ = 6.5 Hz, 2H); $^{13}C\{^1H\}$ NMR (151 MHz, $(CD_3)_2SO$): δ 197.4, 169.2, 160.1, 144.5 (q, $J$ = 40 Hz), 129.2, 120.5 (br), 118.6 (q, $J$ = 272 Hz), 116.0, 110.9 (br), 110.4, 38.5, 28.3, 19.5; $^{19}$F NMR (376 MHz, $CD_3OD$) δ -66.4; HRMS (ESI, m/z) calculated for $C_{15}H_{10}F_3N_2O_3$ $(M + H)^+$ 323.0638, found 323.0640.

**9-(*tert*-butyl)-2,3,4-trihydrobenzo[3,4]isochromeno[6,7-*d*]imidazole-1,6-dione (4g)**

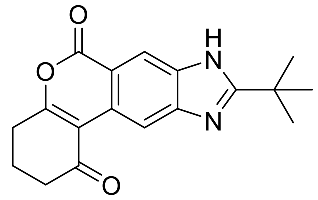

White solid, yield: 12.8 mg, 41%; Eluent: EA/DCM = 1:3; $^1$H NMR (400 MHz, $CD_3OD$): δ 9.05 (s, 1H), 8.24 (s, 1H), 2.87 (t, $J$ = 6.3 Hz, 2H), 2.60 (t, $J$ = 6.7 Hz, 2H), 2.14 (quin, $J$ = 6.5 Hz, 2H), 1.51 (s, 9H); $^{13}C\{^1H\}$ NMR (151 MHz, $CD_3OD$): δ 199.7, 169.7, 169.3, 162.6, 144.3 (br), 140.2 (br), 129.3, 117.7 (br), 115.6, 112.5, 111.4 (br), 39.8, 34.9, 29.6, 29.5, 21.0; HRMS (ESI, m/z) calculated for $C_{18}H_{19}N_2O_3$ $(M + H)^+$ 311.1390, found 311.1395.

**9-mesityl-2,3,4-trihydrobenzo[3,4]isochromeno[6,7-*d*]imidazole-1,6-dione (4h)**

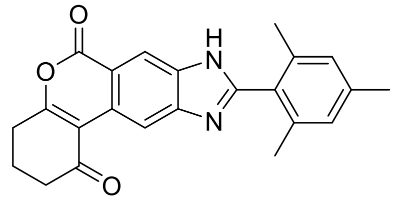

White solid, yield: 20.0 mg, 54%; Eluent: EA/DCM = 1:6; $^1$H NMR (400 MHz, $CD_3OD$): δ 9.30 (s, 1H), 8.51 (s, 1H), 7.04 (s, 2H), 2.96 (t, $J$ = 6.3 Hz, 2H), 2.67 (t, $J$ = 6.7 Hz, 2H), 2.36 (s, 3H), 2.19 (quin, $J$ = 6.5 Hz, 2H), 2.15 (s, 6H); $^{13}C\{^1H\}$ NMR (101 MHz, $CD_3OD$): δ 199.5, 170.0, 162.6, 158.6 (br), 141.4, 138.8, 129.9 (br), 129.4, 128.4, 116.4, 112.6, 39.9, 29.8, 21.3, 21.1, 20.0; HRMS (ESI, m/z) calculated for $C_{23}H_{21}N_2O_3$ $(M + H)^+$ 373.1547, found 373.1542.

**9-(2,6-dichlorophenyl)-2,3,4-trihydrobenzo[3,4]isochromeno[6,7-*d*]imidazole-1,6-dione (4i)**

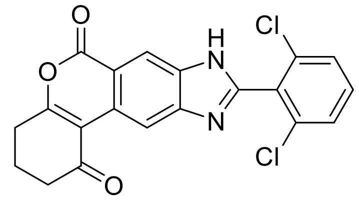

White solid, yield: 10.3 mg, 26%; Eluent: EA/DCM = 1:5; $^1$H NMR (600 MHz, $(CD_3)_2SO$): δ 9.25 (s, 1H), 8.52 (s, 1H), 7.73 – 7.71 (m, 2H), 7.68 – 7.65 (m, 1H), 2.95 (t, $J$ = 6.2 Hz, 2H), 2.63 (t, $J$ = 6.7 Hz, 2H), 2.10 (quin, $J$ = 6.5 Hz, 2H); $^{13}$C{$^1$H} NMR (151 MHz, $(CD_3)_2SO$): δ 197.5, 168.7, 160.5, 151.3, 134.7, 133.0, 129.6, 128.5, 128.2, 114.7, 110.7, 38.5, 28.3, 19.6; HRMS (ESI, m/z) calculated for $C_{20}H_{13}Cl_2N_2O_3$ $(M + H)^+$ 399.0298, found 399.0297.

**9-chloro-2,3,4-trihydrobenzo[3,4]isochromeno[6,7-*d*]imidazole-1,6-dione (4j)**

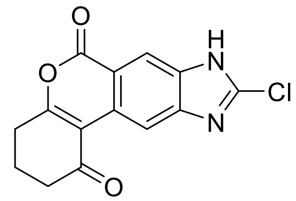

White solid, yield: 6.7 mg, 23%; Eluent: EA/DCM = 1:3; $^1$H NMR (400 MHz, $(CD_3)_2SO$): δ 9.09 (s, 1H), 8.30 (s, 1H), 2.92 (t, $J$ = 6.2 Hz, 2H), 2.61 (t, $J$ = 6.6 Hz, 2H), 2.08 (quin, $J$ = 6.4 Hz, 2H); $^{13}$C{$^1$H} NMR (151 MHz, $(CD_3)_2SO$): δ 197.4, 168.8, 160.2, 143.8, 128.3, 114.6, 110.4, 38.5, 28.3, 19.5; HRMS (ESI, m/z) calculated for $C_{14}H_{10}ClN_2O_3$ $(M + H)^+$ 289.0375, found 289.0378.

**9-bromo-2,3,4-trihydrobenzo[3,4]isochromeno[6,7-*d*]imidazole-1,6-dione (4k)**

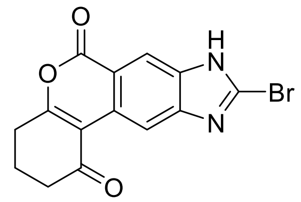

White solid, yield: 7.4 mg, 22%; Eluent: EA/DCM = 1:3; $^1$H NMR (400 MHz, $(CD_3)_2SO$): δ 9.11 (s, 1H), 8.32 (s, 1H), 2.92 (t, $J$ = 6.2 Hz, 2H), 2.61 (t, $J$ = 6.6 Hz, 2H), 2.08 (quin, $J$ = 6.4 Hz, 2H); $^{13}$C{$^1$H} NMR (151 MHz, $(CD_3)_2SO$): δ 197.4, 168.8, 160.3, 132.7 (br), 128.2, 114.5, 110.5, 38.5, 28.3, 19.5; HRMS (ESI, m/z) calculated for $C_{14}H_{10}BrN_2O_3$ $(M + H)^+$ 332.9870, found 332.9871.

**9-(methylthio)-2,3,4-trihydrobenzo[3,4]isochromeno[6,7-*d*]imidazole-1,6-dione (4l)**

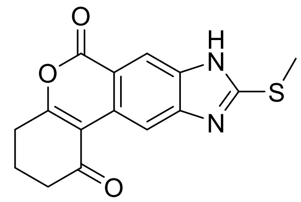

White solid, yield: 7.4 mg, 25%; Eluent: EA/DCM = 1:3; $^1$H NMR (400 MHz, $(CD_3)_2SO$): δ 9.03 (s, 1H), 8.21 (s, 1H), 2.91 (t, $J$ = 6.2 Hz, 2H), 2.75 (s, 3H), 2.60 (t, $J$ = 6.6 Hz, 2H), 2.07 (quin, $J$ = 6.4 Hz, 2H); $^{13}$C{$^1$H} NMR (151 MHz, $(CD_3)_2SO$): δ 197.5, 168.4, 160.5, 157.6 (br), 127.7, 113.6, 110.7, 38.6, 28.3, 19.6, 13.7; HRMS (ESI, m/z) calculated for $C_{15}H_{13}N_2O_3S$ $(M + H)^+$ 301.0642, found 301.0647.

**9-(phenylthio)-2,3,4-trihydrobenzo[3,4]isochromeno[6,7-*d*]imidazole-1,6-dione (4m)**

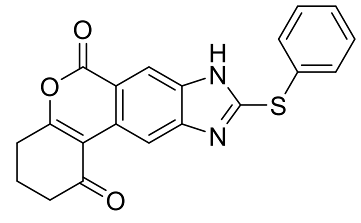

White solid, yield: 9.3 mg, 26%; Eluent: EA/DCM = 1:5; $^{1}$H NMR (400 MHz, $(CD_3)_2SO$): δ 9.03 (s, 1H), 8.23 (s, 1H), 7.70 – 7.65 (m, 2H), 7.53 – 7.49 (m, 3H), 2.90 (t, *J* = 6.2 Hz, 2H), 2.59 (t, *J* = 6.6 Hz, 2H), 2.06 (quin, *J* = 6.4 Hz, 2H); $^{13}C\{^{1}H\}$ NMR (151 MHz, $(CD_3)_2SO$): δ 197.4, 168.5, 160.4, 154.5 (br), 133.5, 129.9, 129.5, 128.3, 127.9, 114.0, 110.6, 38.5, 28.3, 19.5; HRMS (ESI, m/z) calculated for $C_{20}H_{15}N_2O_3S$ $(M + H)^+$ 363.0798, found 363.0800.

**9-ethoxy-2,3,4-trihydrobenzo[3,4]isochromeno[6,7-*d*]imidazole-1,6-dione (4n)**

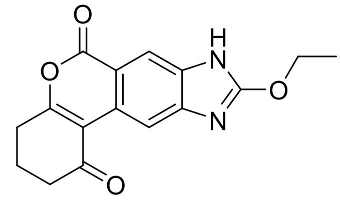

White solid, yield: 10.8 mg, 36%; Eluent: EA/DCM = 1:3; $^{1}$H NMR (400 MHz, $(CD_3)_2SO$): δ 12.46 (br, 1H), 9.03 (s, 1H), 8.05 (s, 1H), 4.56 (q, *J* = 7.1 Hz, 2H), 2.90 (t, *J* = 6.2 Hz, 2H), 2.59 (t, *J* = 6.6 Hz, 2H), 2.07 (quin, *J* = 6.4 Hz, 2H), 1.41 (t, *J* = 7.1 Hz, 3H); $^{13}C\{^{1}H\}$ NMR (151 MHz, $(CD_3)_2SO$): δ 197.4, 168.0, 161.3 (br), 160.4, 127.5, 112.9, 110.8, 66.2, 38.5, 28.2, 19.6, 14.4; HRMS (ESI, m/z) calculated for $C_{16}H_{15}N_2O_4$ $(M + H)^+$ 299.1026, found 299.1033.

**9-benzoyl-2,3,4-trihydrobenzo[3,4]isochromeno[6,7-*d*]imidazole-1,6-dione (4o)**

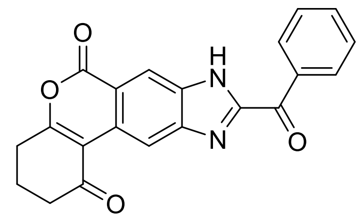

White solid, yield: 8.6 mg, 48%; Eluent: EA/DCM = 1:6; $^{1}$H NMR (600 MHz, $(CD_3)_2SO$): δ 13.89 (br, 1H), 9.29 (s, 1H), 8.61 (s, 1H), 8.56 (d, *J* = 7.4 Hz, 2H), 7.77 (t, *J* = 7.4 Hz, 1H), 7.64 (t, *J* = 7.7 Hz, 2H), 2.93 (t, *J* = 6.2 Hz, 3H), 2.62 (t, *J* = 6.6 Hz, 3H), 2.09 (quin, *J* = 6.4 Hz, 3H); $^{13}C\{^{1}H\}$ NMR (151 MHz, $(CD_3)_2SO$): δ 197.4, 183.4, 169.0, 160.2, 151.5, 135.1, 134.1, 130.9, 128.6, 128.4, 116.0 (br), 110.5, 38.5, 28.4, 19.5; HRMS (ESI, m/z) calculated for $C_{21}H_{15}N_2O_4$ $(M + H)^+$ 359.1026, found 359.1031.

**9-(1-hydroxyethyl)-2,3,4-trihydrobenzo[3,4]isochromeno[6,7-*d*]imidazole-1,6-dione (4p)**

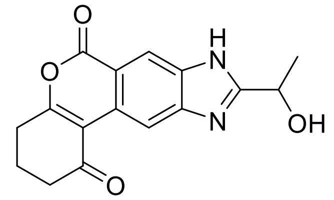

White solid, yield: 2.3 mg, 8%; Eluent: EA; $^{1}$H NMR (400 MHz, $CD_3OD$): δ 9.12 (s, 1H), 8.31 (s, 1H), 5.11 (q, *J* = 6.7 Hz, 1H), 2.92 (t, *J* = 6.3 Hz, 2H), 2.64 (t, *J* = 6.7 Hz, 2H), 2.16 (quin, *J* = 6.5 Hz, 2H), 1.65 (d, *J* = 6.7 Hz, 3H); $^{13}C\{^{1}H\}$ NMR (101 MHz, $CD_3OD$): δ 199.5, 169.8, 165.2 (br), 162.6, 129.5, 116.0, 112.5, 65.5, 39.9, 29.7, 23.0, 21.1; HRMS (ESI, m/z) calculated for $C_{16}H_{15}N_2O_4$ $(M + H)^+$ 299.1026, found 299.1036.

**2,3,4-trihydrobenzo[3,4]isochromeno[6,7-*d*]imidazole-1,6-dione (4q)**

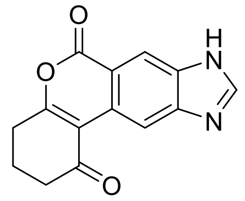

White solid, yield: 5.4 mg, 21%; Eluent: EA; $^1$H NMR (600 MHz, $(CD_3)_2SO$): δ 9.22 (s, 1H), 8.55 (s, 1H), 8.44 (s, 1H), 2.93 (t, *J* = 6.2 Hz, 2H), 2.61 (t, *J* = 6.6 Hz, 2H), 2.08 (quin, *J* = 6.4 Hz, 2H); $^{13}C\{^1H\}$ NMR (151 MHz, $(CD_3)_2SO$): δ 197.4, 168.3, 160.6, 146.8, 127.6, 114.1, 110.7, 38.5, 28.3, 19.5; HRMS (ESI, m/z) calculated for $C_{14}H_{11}N_2O_3$ $(M + H)^+$ 255.0764, found 255.0769.

**9-amino-2,3,4-trihydrobenzo[3,4]isochromeno[6,7-*d*]imidazole-1,6-dione (4r)**

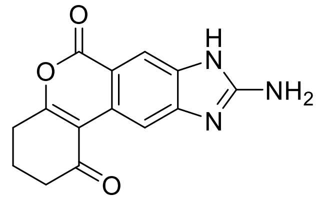

White solid, yield: 3.2 mg, 12%; Eluent: EA/MeOH = 10:1; $^1$H NMR (400 MHz, $(CD_3)_2SO$): δ 8.75 (s, 1H), 7.86 (s, 1H), 7.22 (br, 2H), 2.89 (t, *J* = 6.1 Hz, 2H), 2.58 (t, *J* = 6.5 Hz, 2H), 2.05 (quin, *J* = 6.3 Hz, 2H); $^{13}C\{^1H\}$ NMR (151 MHz, $(CD_3)_2SO$): δ 197.5, 167.4, 160.7, 157.7 (br), 111.1, 38.7, 28.3, 19.7; HRMS (ESI, m/z) calculated for $C_{14}H_{12}N_3O_3$ $(M + H)^+$ 270.0874, found 270.0880.

**9-(furan-2-yl)-2,3,4-trihydrobenzo[3,4]isochromeno[6,7-*d*]imidazole-1,6-dione (4s)**

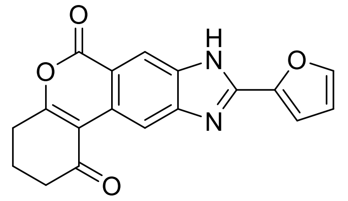

White solid, yield: 5.0 mg, 17%; Eluent: EA/DCM = 1:2; $^1$H NMR (400 MHz, $(CD_3)_2SO$): δ 9.19 (s, 1H), 8.37 (s, 1H), 8.04 (d, *J* = 1.4 Hz, 1H), 7.36 (d, *J* = 3.4 Hz, 1H), 6.80 (dd, *J* = 3.5, 1.7 Hz, 1H), 2.93 (t, *J* = 6.2 Hz, 2H), 2.62 (t, *J* = 6.6 Hz, 2H), 2.09 (quin, *J* = 6.4 Hz, 2H); $^{13}C\{^1H\}$ NMR (151 MHz, $(CD_3)_2SO$): δ 197.4, 168.4 (br), 160.4, 147.6 (br), 145.9, 144.5, 128.1 (br), 114.4, 112.8, 112.7, 110.7, 38.5, 28.3, 19.5; HRMS (ESI, m/z) calculated for $C_{18}H_{13}N_2O_4$ $(M + H)^+$ 321.0870, found 321.0872.

**(*E*)-9-styryl-2,3,4-trihydrobenzo[3,4]isochromeno[6,7-*d*]imidazole-1,6-dione (4t)**

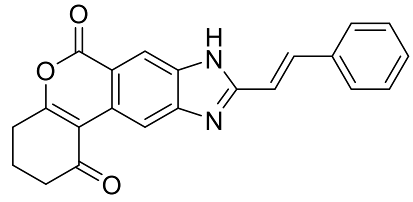

White solid, yield: 2.6 mg, 7%; Eluent: EA/DCM = 1:3; $^1$H NMR (600 MHz, $(CD_3)_2SO$): δ 9.17 (s, 1H), 8.36 (s, 1H), 7.79 (d, *J* = 16.5 Hz, 1H), 7.73 (d, *J* = 7.4 Hz, 2H), 7.47 (t, *J* = 7.5 Hz, 2H), 7.41 (t, *J* = 7.3 Hz, 1H), 7.29 (d, *J* = 16.5 Hz, 1H), 2.93 (t, *J* = 6.2 Hz, 2H), 2.62 (t, *J* = 6.7 Hz, 2H), 2.09 (quin, *J* = 6.4 Hz, 2H); $^{13}C\{^1H\}$ NMR (151 MHz, $(CD_3)_2SO$): δ 197.5, 168.3, 160.5, 155.6 (br), 137.1, 135.3, 129.4, 129.0, 128.0, 127.4, 116.9, 114.1, 110.8, 38.6, 28.3, 19.6; HRMS (ESI, m/z) calculated for $C_{22}H_{17}N_2O_3$ $(M + H)^+$ 357.1234, found 357.1235.

**(*E*)-9-(prop-1-en-1-yl)-2,3,4-trihydrobenzo[3,4]isochromeno[6,7-*d*]imidazole-1,6-dione (4u)**

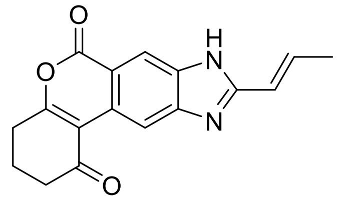

White solid, yield: 2.7 mg, 9%; Eluent: EA/DCM = 1:3; $^{1}$H NMR (400 MHz, $CD_3OD$): δ 9.15 (s, 1H), 8.33 (s, 1H), 6.98 (dq, *J* = 16.0, 6.8 Hz, 1H), 6.53 (dq, *J* = 16.0, 1.8 Hz, 1H), 2.95 (t, *J* = 6.3 Hz, 2H), 2.66 (t, *J* = 6.7 Hz, 2H), 2.17 (quin, *J* = 6.5 Hz, 2H), 2.05 (dd, *J* = 6.8, 1.6 Hz, 3H); $^{13}C\{^{1}H\}$ NMR (151 MHz, $CD_3OD$): δ 199.4, 169.8, 162.6, 157.1, 139.1, 129.9, 120.8, 116.2, 112.5, 39.9, 29.7, 21.1, 18.9; HRMS (ESI, m/z) calculated for $C_{17}H_{15}N_2O_3$ $(M + H)^+$ 295.1077, found 295.1080.

**(*Z*)-9-(prop-1-en-1-yl)-2,3,4-trihydrobenzo[3,4]isochromeno[6,7-*d*]imidazole-1,6-dione (4v)**

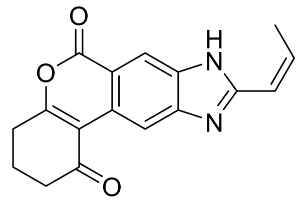

White solid, yield: 7.5 mg, 26%; Eluent: EA/DCM = 1:3; $^{1}$H NMR (400 MHz, $(CD_3)_2SO$): δ 9.15 (s, 1H), 8.35 (s, 1H), 6.45 (dq, *J* = 11.5, 1.5 Hz, 1H), 6.34 (dq, *J* = 11.6, 7.1 Hz, 1H), 2.92 (t, *J* = 6.2 Hz, 2H), 2.61 (t, *J* = 6.6 Hz, 2H), 2.32 (dd, *J* = 7.1, 1.6 Hz, 3H), 2.08 (quin, *J* = 6.4 Hz, 2H); $^{13}C\{^{1}H\}$ NMR (151 MHz, $(CD_3)_2SO$): δ 197.5, 168.3, 160.5, 155.0, 138.0, 127.7, 117.6, 114.0, 110.8, 38.6, 28.3, 19.6, 15.9; HRMS (ESI, m/z) calculated for $C_{17}H_{15}N_2O_3$ $(M + H)^+$ 295.1077, found 295.1084.

**9-benzyl-2,3,4-trihydrobenzo[3,4]isochromeno[6,7-*d*]oxazole-1,6-dione (4x)**

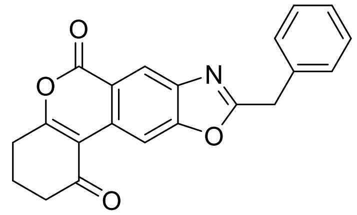

White solid, yield: 4.3 mg, 12%; Eluent: EA/DCM = 1:30; $^{1}$H NMR (400 MHz, $CDCl_3$): δ 9.27 (s, 1H), 8.62 (s, 1H), 7.41 (d, *J* = 7.1 Hz, 2H), 7.37 (t, *J* = 7.6 Hz, 2H), 7.31 (t, *J* = 7.3 Hz, 1H), 2.99 (t, *J* = 6.3 Hz, 2H), 4.33 (s, 2H), 2.96 (t, *J* = 6.3 Hz, 2H), 2.68 (t, *J* = 6.7 Hz, 2H), 2.19 (quin, *J* = 6.5 Hz, 2H); $^{13}C\{^{1}H\}$ NMR (151 MHz, $(CD_3)_2SO$): δ 197.0, 169.1, 168.4, 160.5, 156.1, 141.7, 134.0, 131.5, 129.3, 129.1, 127.8, 121.8, 117.3, 111.6, 108.1, 39.1, 35.5, 29.1, 20.1; HRMS (ESI, m/z) calculated for $C_{21}H_{16}NO_4$ $(M + H)^+$ 346.1074, found 346.1074.

**9-phenyl-2,3,4-trihydrobenzo[3,4]isochromeno[6,7-*d*]oxazole-1,6-dione (4y)**

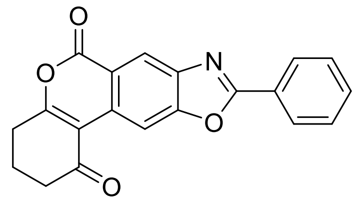

White solid, yield: 2.3 mg, 7%; Eluent: EA/DCM = 1:50; $^{1}$H NMR (600 MHz, $CDCl_3$): δ 9.40 (s, 1H), 8.70 (s, 1H), 8.32 – 8.30 (m, 2H), 7.62 – 7.56 (m, 3H), 2.99 (t, *J* = 6.3 Hz, 2H), 2.71 (t, *J* = 6.7 Hz, 2H), 2.21 (quin, *J* = 6.5 Hz, 2H); $^{13}C\{^{1}H\}$ NMR (151 MHz, $(CD_3)_2SO$): δ 197.1, 169.1, 165.9, 160.5, 155.8, 142.5, 132.7, 131.7, 129.3, 128.3, 126.4, 121.9, 117.7, 111.7, 108.2, 39.1, 29.2, 20.1; HRMS (ESI, m/z) calculated for $C_{20}H_{14}NO_4$ $(M + H)^+$ 332.0918, found 332.0924.

**3-methyl-9-benzyl-2,3,4-trihydrobenzo[3,4]isochromeno[6,7-*d*]imidazole-1,6-dione (4ab)**

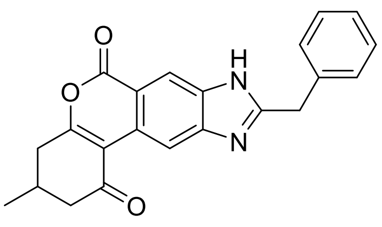

White solid, yield: 12.6 mg, 35%; Eluent: EA/DCM = 1:3; $^{1}H$ NMR (400 MHz, $CD_3OD$): δ 9.20 (s, 1H), 8.40 (s, 1H), 7.37 – 7.32 (m, 4H), 7.28 – 7.25 (m, 1H), 4.30 (s, 2H), 2.94 – 2.89 (m, 1H), 2.73 – 2.61 (m, 2H), 2.45 – 2.37 (m, 2H), 1.17 (d, *J* = 5.7 Hz, 3H); $^{13}C\{^{1}H\}$ NMR (101 MHz, $(CD_3)_2SO$): δ 197.4, 167.6, 160.6, 158.6 (br), 136.7, 128.9, 128.6, 127.5, 126.8, 119.3 (br), 113.7, 110.3, 106.8 (br), 46.5, 35.9, 35.1, 27.1, 20.3; HRMS (ESI, m/z) calculated for $C_{22}H_{19}N_2O_3$ $(M + H)^+$ 359.1390, found 359.1391.

**3,3-dimethyl-9-benzyl-2,3,4-trihydrobenzo[3,4]isochromeno[6,7-*d*]imidazole-1,6-dione (4ac)**

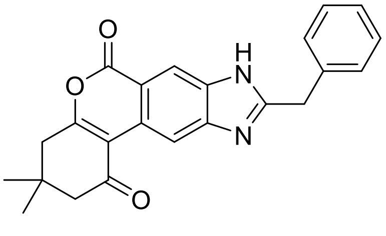

White solid, yield: 12.8 mg, 34%; Eluent: EA/DCM = 1:3; $^{1}H$ NMR (400 MHz, $CD_3OD$): δ 9.18 (s, 1H), 8.40 (s, 1H), 7.37 – 7.33 (m, 4H), 7.28 – 7.24 (m, 1H), 4.30 (s, 2H), 2.83 (s, 2H), 2.53 (s, 2H), 1.16 (s, 6H); $^{13}C\{^{1}H\}$ NMR (101 MHz, $CD_3OD$): δ 199.4, 168.2, 162.9, 160.6, 137.4, 129.9, 129.9, 129.5, 128.3, 118.0 (br), 116.1, 111.7, 111.3 (br), 53.7, 43.1, 36.3, 32.8, 28.2; HRMS (ESI, m/z) calculated for $C_{23}H_{21}N_2O_3$ $(M + H)^+$ 373.1547, found 373.1546.

**3-phenyl-9-benzyl-2,3,4-trihydrobenzo[3,4]isochromeno[6,7-*d*]imidazole-1,6-dione (4ad)**

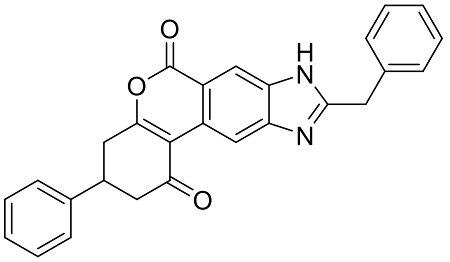

White solid, yield: 14.3 mg, 34%; Eluent: EA/DCM = 1:4; $^{1}H$ NMR (400 MHz, $CDCl_3$): δ 9.21 (s, 1H), 8.55 (s, 1H), 7.38 (t, *J* = 7.3 Hz, 2H), 7.32 – 7.23 (m, 8H), 4.33 (s, 2H), 3.59 – 3.51 (m, 1H), 3.21 – 3.11 (m, 2H), 2.91 – 2.81 (m, 2H); $^{13}C\{^{1}H\}$ NMR (101 MHz, $CDCl_3$): δ 196.9, 167.2, 161.3, 158.2, 141.6, 135.4, 129.3, 129.2, 129.2, 128.3, 127.8, 127.6, 126.7, 115.0, 111.7, 46.1, 38.2, 36.4, 36.2; HRMS (ESI, m/z) calculated for $C_{27}H_{21}N_2O_3$ $(M + H)^+$ 421.1547, found 421.1548.

**9-benzyl-2,4-dihydropyrano[3',4':3,4]isochromeno[6,7-*d*]imidazole-1,6-dione (4ae)**

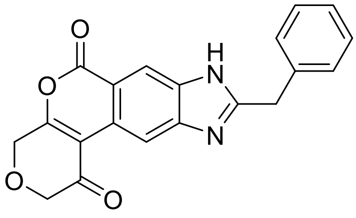

White solid, yield: 13.0 mg, 38%; Eluent: EA/DCM = 1:3; $^{1}H$ NMR (400 MHz, $CD_3OD$): δ 8.95 (s, 1H), 8.34 (s, 1H), 7.36 – 7.32 (m, 4H), 7.28 – 7.25 (m, 1H), 4.69 (s, 2H), 4.30 (s, 2H), 4.26 (s, 2H); $^{13}C\{^{1}H\}$ NMR (101 MHz, $(CD_3)_2SO$): δ 193.1, 165.6 (br), 159.8, 158.6 (br), 140.4 (br), 136.7, 129.0, 128.7, 126.9, 126.2 (br), 119.9 (br), 113.8,

108.8, 106.9 (br), 71.7, 64.3, 35.1; HRMS (ESI, m/z) calculated for $C_{20}H_{15}N_2O_4$ (M + H)$^+$ 347.1026, found 347.1028.

**8-benzyl-2,3-dihydrocyclopenta[3,4]isochromeno[6,7-*d*]imidazole-1,5-dione (4af)**

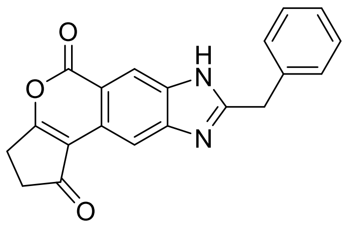


White solid, yield: 9.6 mg, 29%; Eluent: EA/DCM = 1:2; $^1$H NMR (400 MHz, $(CD_3)_2SO$): δ 8.44 (s, 1H), 8.34 (s, 1H), 7.38 – 7.32 (m, 4H), 7.28 – 7.24 (m, 1H), 4.28 (s, 2H), 3.01 – 2.98 (m, 2H), 2.68 – 2.66 (m, 2H); $^{13}$C{$^1$H} NMR (101 MHz, $(CD_3)_2SO$): δ 201.1, 179.3, 161.5, 158.7 (br), 136.7, 129.0, 128.7, 126.9, 125.7, 113.7, 112.5, 35.1, 34.5, 25.2; HRMS (ESI, m/z) calculated for $C_{20}H_{15}N_2O_3$ (M + H)$^+$ 331.1077, found 331.1078.

**10-benzyl-2,3,4,5-tetrahydrocyclohepta[3,4]isochromeno[6,7-*d*]imidazole-1,7-dione (4ag)**

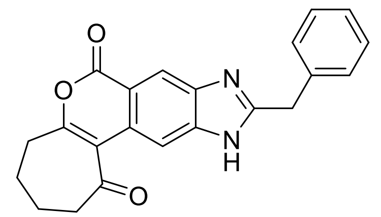


White solid, yield: 3.1 mg, 9%; Eluent: EA/DCM = 1:3; $^1$H NMR (400 MHz, $CDCl_3$): 8.56 (s, 1H), 8.23 (s, 1H), 7.37 – 7.34 (m, 2H), 7.31 – 7.29 (m, 3H), 4.34 (s, 2H), 2.95 (t, *J* = 5.9 Hz, 2H), 2.79 (t, *J* = 6.2 Hz, 2H), 1.97 – 1.94 (m, 4H); $^{13}$C{$^1$H} NMR (151 MHz, $CDCl_3$): δ 203.8, 162.3, 162.1 (br), 158.3 (br), 135.6, 129.6, 129.5, 128.1, 116.8, 115.3, 43.4, 36.4, 32.5, 23.5, 22.8; HRMS (ESI, m/z) calculated for $C_{22}H_{19}N_2O_3$ (M + H)$^+$ 359.1390, found 359.1395.

**9-benzyl-12*H*-chromeno[4',3';3,4]isochromeno[6,7-*d*]imidazole-6,12-dione (4aj)**

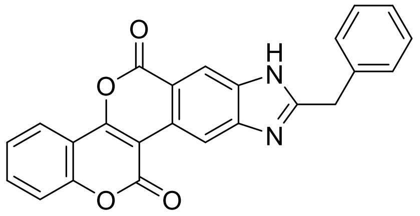


White solid, yield: 4.5 mg, 11%; Eluent: EA/DCM = 1:6; $^1$H NMR (600 MHz, $(CD_3)_2SO$): δ 13.01 (br, 1H), 9.16 (s, 1H), 8.44 (s, 1H), 8.06 (dd, *J* = 8.0, 1.2 Hz, 1H), 7.75 (td, *J* = 7.8, 1.5 Hz, 1H), 7.53 (d, *J* = 8.3 Hz, 1H), 7.50 (t, *J* = 7.7 Hz, 1H), 7.38 – 7.34 (m, 4H), 7.27 (t, *J* = 7.0 Hz, 1H), 4.30 (s, 2H); $^{13}$C{$^1$H} NMR (151 MHz, $(CD_3)_2SO$): δ 159.2, 158.6, 155.2, 151.4, 136.4, 133.0, 128.6, 128.3, 126.5, 126.2, 124.8, 122.7, 116.2, 114.1, 113.3, 101.3, 34.9; HRMS (ESI, m/z) calculated for $C_{24}H_{15}N_2O_4$ (M + H)$^+$ 395.1026, found 395.1025.

**9-benzyl-3-chloro-12*H*-chromeno[4',3';3,4]isochromeno[6,7-*d*]imidazole-6,12-dione (4ak)**

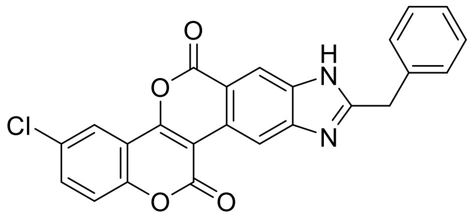

White solid, yield: 10.7 mg, 25%; Eluent: EA/DCM = 1:5; $^1$H NMR (600 MHz, $(CD_3)_2SO$): δ 12.99 (s, 1H), 9.15 (s, 1H), 8.47 (s, 1H), 7.99 (d, *J* = 2.4 Hz, 1H), 7.78 (dd, *J* = 8.8, 2.5 Hz, 1H), 7.58 (d, *J* = 8.8 Hz, 1H), 7.39 – 7.34 (m, 4H), 7.27 (t, *J* = 7.1 Hz, 1H), 4.31 (s, 2H); $^{13}C\{^1H\}$ NMR (151 MHz, $(CD_3)_2SO$): δ 158.8, 158.2, 153.9 (br), 149.9, 136.3, 132.6, 128.8, 128.6, 128.3, 126.5, 121.6, 120.2 (br), 118.3, 114.7, 114.1, 102.0, 34.8; HRMS (ESI, m/z) calculated for $C_{24}H_{14}N_2O_4Cl$ $(M + H)^+$ 429.0637, found 429.0636.

**4-oxo-1,2,3,4-tetrahydrobenzo[4,5]imidazo[1,2-*f*]phenanthridine-12-carboxylic acid (5a)**

**4-oxo-1,2,3,4-tetrahydrobenzo[4,5]imidazo[1,2-*f*]phenanthridine-11-carboxylic acid (5a')**

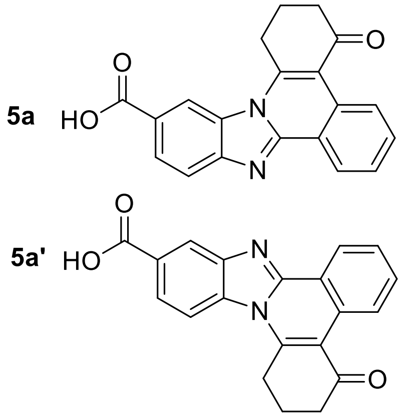


Beige solid, yield: 24.3 mg, 74% as a mixture of **5a**/**5a'** = 1 : 0.64; Eluent: EA/MeOH = 10:1; $^1$H NMR (600 MHz, $(CD_3)_2SO$): **5a**: δ 9.28 (d, *J* = 8.4 Hz, 1H), 8.82 (s, 1H), 8.75 (dd, *J* = 7.9, 1.0 Hz, 1H), 8.14 (dd, *J* = 8.5, 1.2 Hz, 1H), 7.99 (d, *J* = 8.5 Hz, 1H), 7.84 (t, *J* = 7.5 Hz, 1H), 7.73 (t, *J* = 8.0 Hz, 1H), 3.85 (t, *J* = 6.0 Hz, 2H), 2.76 (t, *J* = 6.6 Hz, 2H), 2.33 (quin, *J* = 6.5 Hz, 2H); **5a'**: δ 9.26 (d, *J* = 8.4 Hz, 1H), 8.74 (dd, *J* = 7.8, 1.0 Hz, 1H), 8.45 (d, *J* = 1.4 Hz, 1H), 8.41 (d, *J* = 8.8 Hz, 1H), 8.00 (dd, *J* = 8.8 Hz, 1H), 7.80 (t, *J* = 7.5 Hz, 1H), 7.71 (t, *J* = 8.6 Hz, 1H), 3.80 (t, *J* = 6.1 Hz, 2H), 2.75 (t, *J* = 6.0 Hz, 2H), 2.30 (quin, *J* = 6.5 Hz, 2H); $^{13}C\{^1H\}$ NMR (101 MHz, $(CD_3)_2SO$): **5a**: δ 197.7, 167.5, 150.3, 149.5, 147.7, 131.5, 131.1, 129.0, 127.8, 126.2, 126.2, 124.7, 124.7, 121.3, 119.3, 117.9, 113.8, 38.4, 28.6, 20.3; **5a'**: δ 197.7, 167.5, 150.3, 148.6, 144.2, 134.1, 131.2, 128.8, 127.8, 127.8, 126.2, 124.7, 123.2, 121.4, 120.8, 116.3, 113.8, 38.4, 28.7, 20.3; HRMS (ESI, m/z) calculated for $C_{20}H_{15}N_2O_3$ $(M + H)^+$ 331.1077, found 331.1078.

**8-methyl-4-oxo-1,2,3,4-tetrahydrobenzo[4,5]imidazo[1,2-*f*]phenanthridine-12-carboxylic acid (5b)**

**8-methyl-4-oxo-1,2,3,4-tetrahydrobenzo[4,5]imidazo[1,2-*f*]phenanthridine-11-carboxylic acid (5b')**

5b 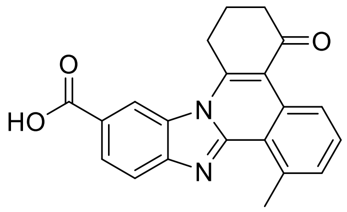

5b' 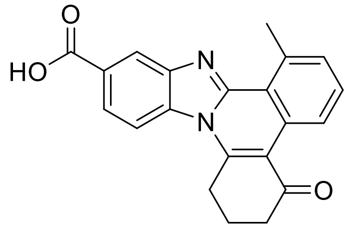

Beige solid, yield: 26.4 mg, 77% as a mixture of **5b**/**5b'** = 1 : 0.76; Eluent: EA/MeOH = 10:1; $^1$H NMR (600 MHz, $(CD_3)_2SO$): **5b**: δ 9.13 (d, *J* = 8.4 Hz, 1H), 8.79 (s, 1H), 8.10 (d, *J* = 8.5 Hz, 1H), 7.97 (d, *J* = 8.6 Hz, 1H), 7.66 (t, *J* = 7.8 Hz, 1H), 7.51 (d, *J* = 8.0 Hz, 1H), 3.78 (t, *J* = 6.0 Hz, 2H), 3.11 (s, 3H), 2.74 (t, *J* = 6.5 Hz, 2H), 2.30 (quin, *J* = 6.4 Hz, 2H); **5b'**: δ 9.10 (d, *J* = 8.3 Hz, 1H), 8.42 (d, *J* = 1.1 Hz, 1H), 8.37 (d, *J* = 8.9 Hz, 1H), 7.98 (dd, *J* = 9.2, 1.4 Hz, 1H), 7.63 (t, *J* = 7.4 Hz, 1H), 7.50 (d, *J* = 8.8 Hz, 1H), 3.73 (t, *J* = 6.0 Hz, 2H), 3.10 (s, 3H), 2.73 (t, *J* = 6.2 Hz, 2H), 2.26 (quin, *J* = 6.5 Hz, 2H); $^{13}C\{^1H\}$ NMR (151 MHz, $(CD_3)_2SO$): **5b**: δ 197.5, 167.5, 149.8, 148.8, 147.3, 137.8, 130.5, 130.3, 130.0, 129.9, 125.9, 124.8, 123.8, 120.1, 119.5, 117.9, 114.4, 38.6, 28.8, 25.1, 20.4; **5b'**: 197.5, 167.3, 150.2, 148.8, 143.9, 137.7, 133.0, 130.5, 130.3, 130.1, 127.4, 123.8, 123.3, 121.1, 120.2, 116.2, 114.4, 38.6, 28.9, 25.0, 20.4; HRMS (ESI, m/z) calculated for $C_{21}H_{17}N_2O_3$ $(M + H)^+$ 345.1234, found 345.1237.

**6-methyl-4-oxo-1,2,3,4-tetrahydrobenzo[4,5]imidazo[1,2-*f*]phenanthridine-12-carboxylic acid (5c)**

**6-methyl-4-oxo-1,2,3,4-tetrahydrobenzo[4,5]imidazo[1,2-*f*]phenanthridine-11-carboxylic acid (5c')**

5c 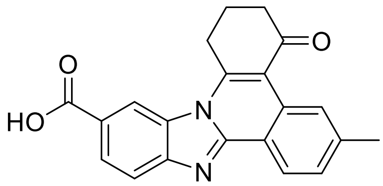

5c' 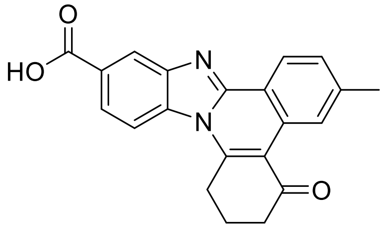

Beige solid, yield: 29.0 mg, 84% as a mixture of **5c**/**5c'** = 1 : 0.68; Eluent: EA/MeOH = 10:1; $^1$H NMR (600 MHz, $(CD_3)_2SO$): **5c**: δ 9.05 (s, 1H), 8.74 (d, *J* = 0.6 Hz, 1H), 8.60 (d, *J* = 8.1 Hz, 1H), 8.11 (dd, *J* = 8.4, 1.4 Hz, 1H), 7.94 (d, *J* = 8.5 Hz, 1H), 7.52 (dd, *J* = 7.5, 1.0 Hz, 1H), 3.78 (t, *J* = 6.1 Hz, 2H), 2.74 (t, *J* = 6.7 Hz, 2H), 2.51 (s, 3H), 2.32 (quin, *J* = 6.6 Hz, 2H); **5c'**: δ 9.04 (s, 1H), 8.59 (d, *J* = 8.1 Hz, 1H), 8.40 (d, *J* = 1.5 Hz, 1H), 8.34 (d, *J* = 8.8 Hz, 1H), 7.95 (dd, *J* = 8.8, 1.7 Hz, 1H), 7.51 (dd, *J* = 7.5, 1.0 Hz, 1H), 3.75 (t, *J* = 6.1 Hz, 2H), 2.73 (t, *J* = 6.7 Hz, 2H), 2.51 (s, 3H), 2.28 (quin, *J* = 6.6 Hz, 2H); $^{13}C\{^1H\}$ NMR (151 MHz, $(CD_3)_2SO$): **5c**: δ 197.6, 167.4, 150.2, 149.5, 147.8, 141.4, 131.0, 129.0, 128.8, 126.1, 125.8, 124.6, 124.4, 119.0, 119.0, 117.8, 113.7, 38.4, 28.5, 22.0, 20.3; **5c'**: δ 197.6, 167.3, 150.2, 148.7, 144.3, 141.1, 134.0, 129.1, 129.0, 127.6, 125.9, 124.6, 123.0, 120.6, 119.1, 116.2, 113.7, 38.4, 28.7, 22.0, 20.3; HRMS (ESI, m/z) calculated for $C_{21}H_{17}N_2O_3$ $(M + H)^+$ 345.1234, found 345.1235.

**7-methyl-4-oxo-1,2,3,4-tetrahydrobenzo[4,5]imidazo[1,2-*f*]phenanthridine-12-carboxylic acid (5d)**

**7-methyl-4-oxo-1,2,3,4-tetrahydrobenzo[4,5]imidazo[1,2-*f*]phenanthridine-11-carboxylic acid (5d')**

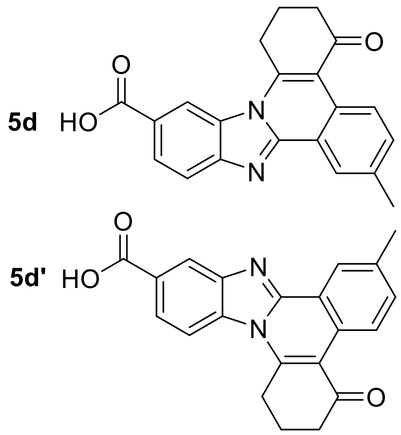

Beige solid, yield: 31.1 mg, 90% as a mixture of **5d**/**5d'** = 1 : 0.61; Eluent: EA/MeOH = 10:1; $^{1}$H NMR (600 MHz, $(CD_3)_2SO$): **5d**: δ 9.12 (d, *J* = 8.3 Hz, 2H), 8.73 (s, 1H), 8.46 (s, 1H), 8.09 (dd, *J* = 8.5, 1.3 Hz, 1H), 7.93 (d, *J* = 8.5 Hz, 1H), 7.60 (dd, *J* = 8.6, 2.0 Hz, 1H), 3.75 (t, *J* = 6.1 Hz, 2H), 2.74 (t, *J* = 6.0 Hz, 2H), 2.49 (s, 3H), 2.31 (quin, *J* = 6.5 Hz, 2H); **5d'**: δ 9.11 (d, *J* = 7.9 Hz, 1H), 8.46 (s, 1H), 8.39 (d, *J* = 1.6 Hz, 1H), 8.33 (d, *J* = 8.9 Hz, 1H), 7.96 (dd, *J* = 8.8, 1.7 Hz, 1H), 7.58 (dd, *J* = 8.6, 2.0 Hz, 1H), 3.72 (t, *J* = 6.2 Hz, 2H), 2.71 (t, *J* = 6.8 Hz, 2H), 2.48 (s, 3H), 2.28 (quin, *J* = 6.7 Hz, 2H); $^{13}C\{^{1}H\}$ NMR (151 MHz, $(CD_3)_2SO$): **5d**: δ 197.7, 167.5, 149.3, 149.3, 147.6, 137.5, 132.8, 131.0, 126.7, 126.1, 126.0, 124.6, 124.2, 121.3, 119.1, 117.8, 113.8, 38.4, 28.5, 20.9, 20.3; **5d'**: δ 197.6, 167.4, 149.3, 148.5, 144.2, 137.4, 134.0, 132.5, 127.7, 126.4, 126.1, 124.2, 123.1, 121.4, 120.7, 116.2, 113.8, 38.4, 28.6, 20.9, 20.3; HRMS (ESI, m/z) calculated for $C_{21}H_{17}N_2O_3$ $(M + H)^+$ 345.1234, found 345.1243.

**8-methoxy-4-oxo-1,2,3,4-tetrahydrobenzo[4,5]imidazo[1,2-*f*]phenanthridine-12-carboxylic acid (5e)**

**8-methoxy-4-oxo-1,2,3,4-tetrahydrobenzo[4,5]imidazo[1,2-*f*]phenanthridine-11-carboxylic acid (5e')**

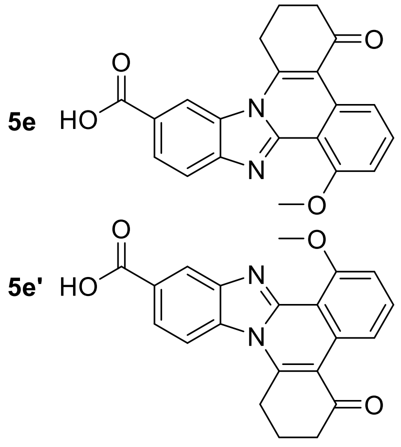

Pale yellow-orange solid, yield: 33.7 mg, 94% as a mixture of **5e**/**5e'** = 1 : 0.81; Eluent: DCM/MeOH = 10:1; $^{1}$H NMR (600 MHz, $(CD_3)_2SO$): **5e**: δ 8.84 (dd, *J* = 8.8, 0.3 Hz, 1H), 8.77 (s, 1H), 8.08 (dd, *J* = 8.4, 1.2 Hz, 1H), 7.98 (d, *J* = 8.5 Hz, 1H), 7.73 (t, *J* = 8.3 Hz, 1H), 7.31 (d, *J* = 8.0 Hz, 1H), 4.05 (s, 3H), 3.81 (t, *J* = 6.0 Hz, 2H), 2.74 (t, *J* = 6.7 Hz, 2H), 2.31 (quin, *J* = 6.4 Hz, 2H); **5e'**: δ 8.83 (dd, *J* = 8.8, 0.5 Hz, 1H), 8.45 (d, *J* = 1.6 Hz, 1H), 8.36 (d, *J* = 8.9 Hz, 1H), 7.95 (dd, *J* = 8.4, 1.7 Hz, 1H), 7.71 (t, *J* = 8.3 Hz, 1H), 7.30 (d, *J* = 8.2 Hz, 1H), 4.05 (s, 3H), 3.77 (t, *J* = 6.1 Hz, 2H), 2.73 (t, *J* = 6.7 Hz, 2H), 2.27 (quin, *J* = 6.6 Hz, 2H); $^{13}C\{^{1}H\}$ NMR (151 MHz, $(CD_3)_2SO$): **5e**: δ 197.4, 167.6, 157.9, 150.5, 148.0, 147.5, 131.9, 131.2, 129.8, 125.9, 124.7, 119.4, 117.8, 117.4, 113.9, 111.3, 109.9, 56.1, 38.6, 28.9, 20.4; **5e'**: δ 197.4, 167.4, 157.8, 150.5, 146.5, 144.6, 132.9, 131.6, 131.0, 127.5, 123.2, 121.0, 117.7, 116.1, 114.0, 111.3, 110.0, 56.1, 38.6, 29.0, 20.4; HRMS (ESI, m/z) calculated for

$C_{21}H_{17}N_2O_4$ $(M + H)^+$ 361.1183, found 361.1186.

**4-oxo-8-(trifluoromethyl)-1,2,3,4-tetrahydrobenzo[4,5]imidazo[1,2-*f*]phenanthridine-12-carboxylic acid (5h)**

**4-oxo-8-(trifluoromethyl)-1,2,3,4-tetrahydrobenzo[4,5]imidazo[1,2-*f*]phenanthridine-11-carboxylic acid (5h')**

**5h** 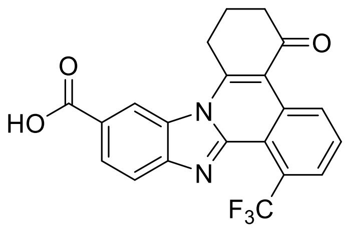


**5h'** 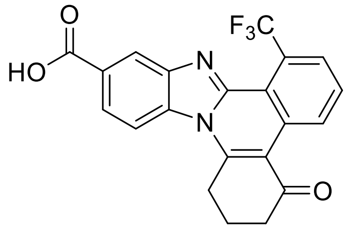


Pale brown solid, yield: 17.4 mg, 44% as a mixture of **5h**/**5h'** = 1 : 0.35; Eluent: EA/MeOH = 10:1; $^1$H NMR (400 MHz, $(CD_3)_2SO$): **5h**: δ 9.57 (d, *J* = 8.7 Hz, 1H), 8.89 (s, 1H), 8.21 (d, *J* = 7.2 Hz, 1H), 8.17 (dd, *J* = 8.6, 1.3 Hz, 1H), 8.05 (d, *J* = 8.5 Hz, 1H), 7.98 (t, *J* = 8.1 Hz, 1H), 3.90 (t, *J* = 6.0 Hz, 2H), 2.79 (t, *J* = 6.7 Hz, 2H), 2.33 (quin, *J* = 6.6 Hz, 2H); **5h'**: δ 9.55 (d, *J* = 8.5 Hz, 1H), 8.49 (d, *J* = 8.9 Hz, 1H), 8.47 (d, *J* = 1.6 Hz, 1H), 8.20 (d, *J* = 7.1 Hz, 1H), 8.06 (dd, *J* = 8.9, 1.7 Hz, 1H), 7.96 (t, *J* = 8.1 Hz, 1H), 3.84 (t, *J* = 6.2 Hz, 2H), 2.78 (t, *J* = 6.7 Hz, 2H), 2.30 (quin, *J* = 6.5 Hz, 2H); $^{13}C\{^1H\}$ NMR (151 MHz, $(CD_3)_2SO$): **5h**: δ 197.3, 167.5, 151.3, 146.8, 145.8, 131.4, 130.7, 130.7, 130.2, 127.2 (q, *J* = 8 Hz), 126.3, 126.3, 125.5 (q, *J* = 75 Hz), 124.1 (q, *J* = 273 Hz), 119.9, 118.6, 118.1, 113.7, 38.5, 28.8, 20.3; **5h'**: δ 197.3, 167.5, 151.2, 144.9, 143.6, 133.1, 131.2, 130.2, 130.0, 128.6, 127.3 (q, *J* = 8 Hz), 125.5 (q, *J* = 75 Hz), 124.1 (q, *J* = 273 Hz), 124.1, 121.4, 118.8, 116.5, 113.7, 38.5, 28.9, 20.3; $^{19}F$ NMR (564 MHz, $(CD_3)_2SO$): **5h**: δ -57.3; **5h'**: δ -57.4; HRMS (ESI, m/z) calculated for $C_{21}H_{14}F_3N_2O_3$ $(M + H)^+$ 399.0952, found 399.0948.

**8-nitro-4-oxo-1,2,3,4-tetrahydrobenzo[4,5]imidazo[1,2-*f*]phenanthridine-12-carboxylic acid (5i)**

**8-nitro-4-oxo-1,2,3,4-tetrahydrobenzo[4,5]imidazo[1,2-*f*]phenanthridine-11-carboxylic acid (5i')**

**5i** 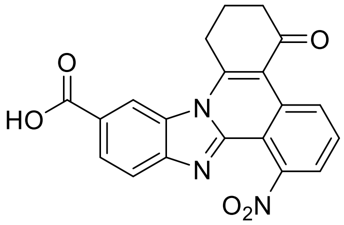


**5i'** 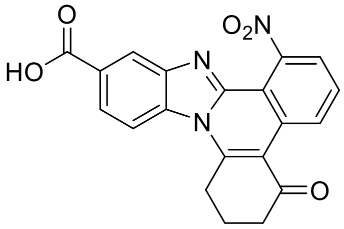


Beige solid, yield: 19.4 mg, 52% as a mixture of **5i**/**5i'** = 1 : 0.48; Eluent: EA/MeOH/$H_2O$ = 70:10:1; $^1$H NMR (600 MHz, $(CD_3)_2SO$): **5i**: δ 9.48 (dd, *J* = 8.5, 0.8 Hz, 1H), 8.84 (s, 1H), 8.14 (dd, *J* = 8.4, 1.1 Hz, 1H), 8.05 (dd, *J* = 7.3, 0.7 Hz, 1H), 7.98 (t, *J* = 8.1 Hz, 1H), 7.96 (d, *J* = 8.5 Hz, 1H), 3.88 (t, *J* = 6.0 Hz, 2H), 2.79 (t, *J* = 6.4 Hz, 2H), 2.34 (quin, *J* = 6.3 Hz, 2H); **5i'**: δ 9.46 (dd, *J* = 8.7, 0.9 Hz, 1H), 8.45 (d, *J* = 9.1 Hz, 1H), 8.36 (d, *J* = 1.3 Hz, 1H), 8.03 (dd, *J* = 7.3, 1.0 Hz, 1H), 8.03 (dd, *J* = 8.9, 1.6 Hz, 1H), 7.97 (t, *J* = 8.3 Hz, 1H), 3.82 (t, *J* = 6.2 Hz, 2H), 2.78 (t, *J* = 6.4 Hz, 2H), 2.30

(quin, $J$ = 6.8 Hz, 2H); $^{13}C\{^1H\}$ NMR (151 MHz, $(CD_3)_2SO$): **5i**: δ 197.5, 167.4, 152.1, 147.5, 146.9, 144.3, 131.5, 130.5, 130.5, 128.6, 126.6, 126.1, 121.8, 119.9, 118.1, 113.2, 111.2, 38.4, 28.8, 20.2; **5i'**: δ 197.5, 167.2, 152.1, 147.5, 143.7, 143.5, 133.3, 131.3, 130.3, 128.6, 128.6, 124.3, 121.8, 121.2, 116.6, 113.2, 111.3, 38.4, 28.9, 20.2; HRMS (ESI, m/z) calculated for $C_{20}H_{14}N_3O_5$ $(M + H)^+$ 376.0928, found 376.0928.

**5-oxo-5,6,7,8-tetrahydrobenzo[*c*]benzo[4,5]imidazo[2,1-*a*][2,6]naphthyridine-11-carboxylic acid (5j)**

**5-oxo-5,6,7,8-tetrahydrobenzo[*c*]benzo[4,5]imidazo[2,1-*a*][2,6]naphthyridine-12-carboxylic acid (5j')**

5j
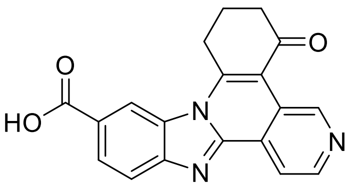


5j'
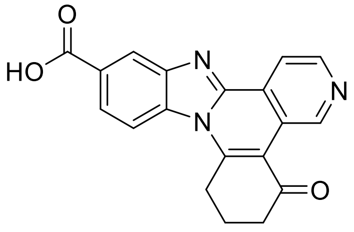


Pale orange solid, yield: 10.1 mg, 31% as a mixture of **5j**/**5j'** = 1 : 0.24; Eluent: EA/MeOH/$H_2O$ = 70:10:1; $^1H$ NMR (600 MHz, $(CD_3)_2SO$): **5j**: δ 10.49 (s, 1H), 8.89 (s, 1H), 8.85 (d, $J$ = 5.3 Hz, 1H), 8.57 (dd, $J$ = 5.1, 0.8 Hz, 1H), 8.20 (dd, $J$ = 8.5, 1.3 Hz, 1H), 8.10 (d, $J$ = 8.3 Hz, 1H), 3.90 (t, $J$ = 6.0 Hz, 2H), 2.80 (t, $J$ = 6.8 Hz, 2H), 2.36 (quin, $J$ = 6.8 Hz, 2H); **5j'**: δ 10.47 (s, 1H), 8.84 (d, $J$ = 5.2 Hz, 1H), 8.56 (dd, $J$ = 5.1, 0.8 Hz, 1H), 8.54 (d, $J$ = 1.4 Hz, 1H), 8.50 (d, $J$ = 9.1 Hz, 1H), 8.09 (dd, $J$ = 8.9, 1.7 Hz, 1H), 3.84 (t, $J$ = 6.1 Hz, 2H), 2.79 (t, $J$ = 7.0 Hz, 2H), 2.33 (quin, $J$ = 6.4 Hz, 2H); $^{13}C\{^1H\}$ NMR (151 MHz, $(CD_3)_2SO$): **5j**: δ 197.4, 167.3, 152.0, 149.0, 147.6, 147.3, 146.7, 131.2, 126.5, 126.3, 126.0, 123.8, 120.0, 118.2, 117.0, 112.8, 38.1, 28.6, 20.3; **5j'**: δ 197.4, 167.2, 151.9, 149.0, 146.8, 146.7, 144.1, 134.1, 128.2, 126.3, 124.3, 123.6, 121.4, 117.0, 116.6, 112.8, 38.1, 28.7, 20.3; HRMS (ESI, m/z) calculated for $C_{19}H_{14}N_3O_3$ $(M + H)^+$ 332.1030, found 332.1031.

**4-oxo-4,5,6,7-tetrahydrobenzo[4,5]imidazo[1,2-*a*]thieno-[2,3-*c*]quinoline-10-carboxylic acid (5k)**

**4-oxo-4,5,6,7-tetrahydrobenzo[4,5]imidazo[1,2-*a*]thieno-[2,3-*c*]quinoline-11-carboxylic acid (5k')**

5k
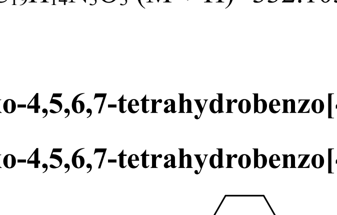


5k'
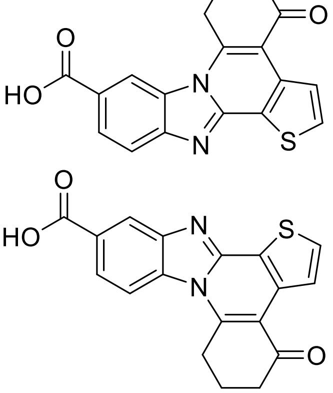


Beige solid, yield: 28.4 mg, 85% as a mixture of **5k**/**5k'** = 1 : 0.59; Eluent: EA/MeOH = 10:1; $^1H$ NMR (400 MHz, $(CD_3)_2SO$): **5k**: δ 8.83 (d, $J$ = 0.6 Hz, 1H), 8.48 (d, $J$ = 5.3 Hz, 1H), 8.19 (d, $J$ = 5.2 Hz, 1H), 8.14 (dd, $J$ = 8.5, 1.4 Hz, 1H), 7.92 (d, $J$ = 8.5 Hz, 1H), 3.84 (t, $J$ = 6.0 Hz, 2H), 2.75 (t, $J$ = 6.6 Hz, 2H), 2.36 (quin, $J$ = 6.4 Hz, 2H); **5k'**: δ 8.46 (d, $J$ = 5.2 Hz, 1H), 8.44 (d, $J$ = 8.9 Hz, 1H), 8.39 (d, $J$ = 1.6 Hz, 1H), 8.15 (d, $J$ = 5.2 Hz, 1H), 7.98 (dd, $J$ = 8.8,

1.6 Hz, 1H), 3.80 (t, $J$ = 6.0 Hz, 2H), 2.74 (t, $J$ = 5.9 Hz, 2H), 2.33 (quin, $J$ = 6.7 Hz, 2H); $^{13}C\{^{1}H\}$ NMR (151 MHz, $(CD_3)_2SO$): **5k**: δ 196.2, 167.4, 149.6, 148.3, 146.9, 136.7, 132.9, 130.5, 126.6, 125.9, 124.7, 124.0, 118.8, 117.9, 114.1, 37.2, 27.8, 20.6; **5k'**: δ 196.1, 167.3, 149.5, 146.1, 144.8, 136.2, 133.5, 132.3, 128.0, 125.9, 124.7, 122.7, 120.4, 116.3, 114.0, 37.2, 27.9, 20.6; HRMS (ESI, m/z) calculated for $C_{18}H_{13}N_2O_3S$ $(M + H)^+$ 337.0641, found 337.0640.

**1-methyl-4-oxo-4,5,6,7-tetrahydrobenzo[4,5]imidazo[1,2-*a*]pyrrolo-[2,3-*c*]quinoline-10-carboxylic acid (5l)**

**1-methyl-4-oxo-4,5,6,7-tetrahydrobenzo[4,5]imidazo[1,2-*a*]pyrrolo-[2,3-*c*]quinoline-11-carboxylic acid (5l')**

5l

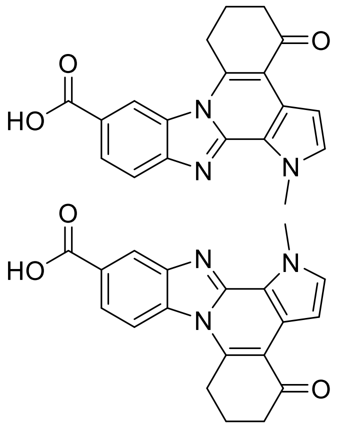

5l'

Pale yellow-orange solid, yield: 26.1 mg, 78% as a mixture of **5l**/**5l'** = 1 : 0.30; Eluent: EA/MeOH = 10:1; $^{1}H$ NMR (600 MHz, $(CD_3)_2SO$): **5l**: δ 8.73 (s, 1H), 8.05 (dd, $J$ = 8.5, 0.8 Hz, 1H), 7.82 (d, $J$ = 8.5 Hz, 1H), 7.51 (d, $J$ = 2.6 Hz, 1H), 7.20 (d, $J$ = 2.6 Hz, 1H), 4.28 (s, 3H), 3.67 (t, $J$ = 6.1 Hz, 2H), 2.66 (t, $J$ = 6.6 Hz, 2H), 2.31 (quin, $J$ = 6.5 Hz, 2H); **5l'**: δ 8.32 (d, $J$ = 8.9 Hz, 1H), 8.31 (s, 1H), 7.88 (dd, $J$ = 8.7, 1.5 Hz, 1H), 7.47 (d, $J$ = 2.6 Hz, 1H), 7.18 (d, $J$ = 2.6 Hz, 1H), 4.28 (s, 3H), 3.65 (t, $J$ = 6.7 Hz, 2H), 2.65 (t, $J$ = 6.3 Hz, 2H), 2.27 (quin, $J$ = 7.1 Hz, 2H); $^{13}C\{^{1}H\}$ NMR (151 MHz, $(CD_3)_2SO$): **5l**: δ 196.7, 167.6, 148.5, 144.6, 143.9, 132.4, 130.1, 126.0, 123.0, 122.2, 119.4, 118.2, 117.3, 113.3, 104.4, 37.0, 35.8, 27.5, 21.2; **5l'**: δ 196.7, 167.5, 144.9, 144.6, 143.3, 133.2, 131.7, 127.4, 121.8, 121.5, 119.9, 119.3, 115.6, 113.3, 104.5, 37.0, 35.8, 27.6, 21.2; HRMS (ESI, m/z) calculated for $C_{19}H_{16}N_3O_3$ $(M + H)^+$ 334.1187, found 334.1193.

**4-oxo-4,5,6,7-tetrahydrobenzo[4,5]imidazo[1,2-*a*]furo-[2,3-*c*]quinoline-10-carboxylic acid (5n)**

**4-oxo-4,5,6,7-tetrahydrobenzo[4,5]imidazo[1,2-*a*]furo-[2,3-*c*]quinoline-11-carboxylic acid (5n')**

5n

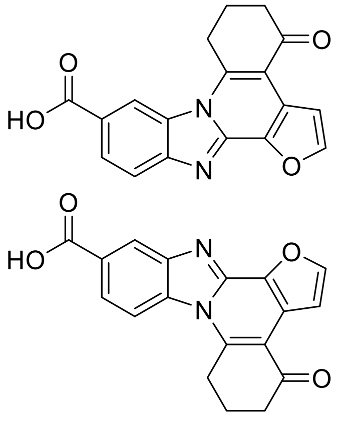

5n'

Beige solid, yield: 6.3 mg, 20% as a mixture of **5n**/**5n'** = 1 : 0.73; Eluent: EA/MeOH/$H_2O$ = 70:10:1; $^{1}H$ NMR (600 MHz, $(CD_3)_2SO$): **5n**: δ 8.85 (s, 1H), 8.39 (d, $J$ = 1.8 Hz, 1H), 8.15 (dd, $J$ = 8.5, 1.2 Hz, 1H), 7.96 (d, $J$ = 8.6 Hz, 1H), 7.64 (d, $J$ = 1.9 Hz, 1H), 3.82 (t, $J$ = 6.1 Hz, 2H), 2.73 (t, $J$ = 5.9 Hz, 2H), 2.37 (quin, $J$ = 6.5 Hz, 2H); **5n'**: δ 8.46 (d, $J$ = 8.9 Hz, 1H), 8.42 (d, $J$ = 1.5 Hz, 1H), 8.36 (d, $J$ = 1.8 Hz, 1H), 7.98 (dd, $J$ = 8.8, 1.6 Hz, 1H), 7.62 (d, $J$ = 1.8

Hz, 1H), 3.78 (t, *J* = 6.2 Hz, 2H), 2.72 (t, *J* = 6.7 Hz, 2H), 2.34 (quin, *J* = 6.6 Hz, 2H); $^{13}C\{^1H\}$ NMR (151 MHz, $(CD_3)_2SO$): **5n**: δ 196.2, 167.4, 149.5, 148.5, 148.5, 141.7, 139.5, 130.5, 126.7, 124.1, 124.0, 119.2, 117.9, 112.3, 109.0, 36.6, 27.5, 21.0; **5n'**: δ 196.2, 167.3, 149.1, 148.5, 145.0, 141.0, 139.5, 133.5, 128.2, 123.3, 122.7, 120.7, 116.3, 112.2, 109.0, 36.6, 27.6, 21.0; HRMS (ESI, m/z) calculated for $C_{18}H_{13}N_2O_4$ $(M + H)^+$ 321.0870, found 321.0870.

**7-oxo-7,8,9,10-tetrahydrobenzo[*i*]benzo[4,5]imidazo[1,2-*f*]phenanthridine-13-carboxylic acid (5o)**
**7-oxo-7,8,9,10-tetrahydrobenzo[*i*]benzo[4,5]imidazo[1,2-*f*]phenanthridine-14-carboxylic acid (5o')**

5o 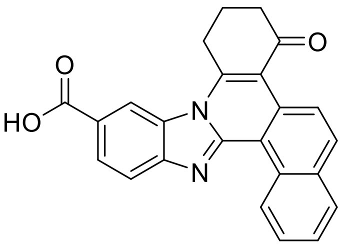

5o' 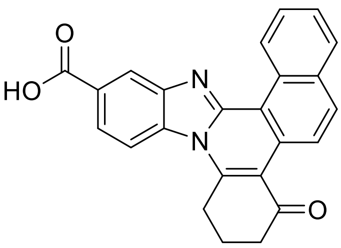

Beige solid, yield: 30.1 mg, 79% as a mixture of **5o**/**5o'** = 1 : 0.64; Eluent: EA/MeOH = 10:1; $^1H$ NMR (600 MHz, $(CD_3)_2SO$): **5o**: δ 10.72 (d, *J* = 8.7 Hz, 1H), 9.15 (d, *J* = 9.2 Hz, 1H), 8.76 (s, 1H), 8.15 (d, *J* = 9.2 Hz, 1H), 8.13 (dd, *J* = 8.5, 1.1 Hz, 1H), 8.03 (d, *J* = 8.4 Hz, 1H), 8.02 (d, *J* = 8.0 Hz, 1H), 7.82 (t, *J* = 7.8 Hz, 1H), 7.69 (t, *J* = 7.4 Hz, 1H), 3.76 (t, *J* = 5.8 Hz, 2H), 2.76 (t, *J* = 5.7 Hz, 2H), 2.31 (quin, *J* = 6.1 Hz, 2H); **5o'**: δ 10.71 (d, *J* = 8.6 Hz, 1H), 9.15 (d, *J* = 9.2 Hz, 1H), 8.49 (d, *J* = 1.2 Hz, 1H), 8.36 (d, *J* = 8.9 Hz, 1H), 8.14 (d, *J* = 9.2 Hz, 1H), 8.02 (d, *J* = 8.0 Hz, 1H), 7.96 (dd, *J* = 8.8, 1.5 Hz, 1H), 7.80 (t, *J* = 7.8 Hz, 1H), 7.68 (t, *J* = 7.4 Hz, 1H), 3.74 (t, *J* = 6.1 Hz, 2H), 2.75 (t, *J* = 5.7 Hz, 2H), 2.28 (quin, *J* = 6.4 Hz, 2H); $^{13}C\{^1H\}$ NMR (151 MHz, $(CD_3)_2SO$): **5o**: δ 197.6, 167.6, 150.1, 148.9, 147.5, 131.9, 131.8, 130.2, 129.5, 128.9, 128.2, 128.2, 128.0, 127.0, 126.4, 124.8, 123.1, 119.3, 118.2, 116.0, 114.7, 38.7, 28.8, 20.4; **5o'**: δ 197.6, 167.5, 150.1, 148.1, 144.2, 132.5, 131.8, 131.6, 129.8, 129.0, 128.2, 128.2, 128.2, 128.0, 126.9, 123.2, 123.2, 120.9, 116.5, 116.1, 114.7, 38.7, 29.0, 20.4; HRMS (ESI, m/z) calculated for $C_{24}H_{17}N_2O_3$ $(M + H)^+$ 381.1234, found 381.1237.

**6-oxo-6,7,8,9-tetrahydrobenzo[4,5]imidazo[1,2-*a*]indolo-[3,2-*c*]quinoline-12-carboxylic acid (5p)**
**6-oxo-6,7,8,9-tetrahydrobenzo[4,5]imidazo[1,2-*a*]indolo-[3,2-*c*]quinoline-13-carboxylic acid (5p')**

5p 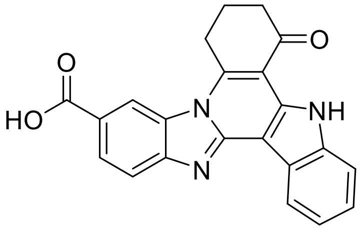

5p' 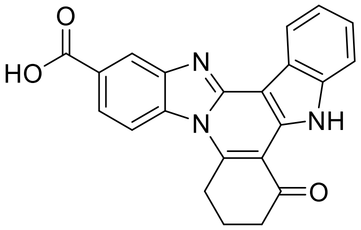

Yellow solid, yield: 21.9 mg, 59% as a mixture of **5p**/**5p’** = 1 : 1.03; Eluent: EA/MeOH = 10:1; $^{1}$H NMR (600 MHz, $(CD_3)_2SO$): **5p**: δ 12.19 (s, 1H), 8.06 (d, $J$ = 0.7 Hz, 1H), 8.40 (d, $J$ = 7.8 Hz, 1H), 8.12 (dd, $J$ = 8.4, 1.3 Hz, 1H), 7.90 (d, $J$ = 8.4 Hz, 1H), 7.87 (d, $J$ = 8.2 Hz, 1H), 7.43 (td, $J$ = 7.4, 1.1 Hz, 1H), 7.36 (td, $J$ = 7.4, 0.9 Hz, 1H), 3.84 (t, $J$ = 6.2 Hz, 2H), 2.80 (t, $J$ = 6.7 Hz, 2H), 2.41 (quin, $J$ = 6.6 Hz, 2H); **5p’**: δ 12.14 (s, 1H), 8.40 (d, $J$ = 7.8 Hz, 1H), 8.39 (d, $J$ = 1.7 Hz, 1H), 8.37 (d, $J$ = 8.9 Hz, 1H), 7.90 (d, $J$ = 8.4 Hz, 1H), 7.87 (d, $J$ = 8.0 Hz, 1H), 7.42 (td, $J$ = 7.5, 1.2 Hz, 1H), 7.36 (td, $J$ = 7.4, 1.0 Hz, 1H), 3.82 (t, $J$ = 6.1 Hz, 2H), 2.79 (t, $J$ = 6.7 Hz, 2H), 2.37 (quin, $J$ = 6.4 Hz, 2H); $^{13}C\{^{1}H\}$ NMR (151 MHz, $(CD_3)_2SO$): **5p**: δ 197.1, 167.6, 150.0, 149.7, 148.4, 138.7, 135.5, 130.0, 126.6, 124.6, 122.4, 121.2, 121.2, 120.8, 118.0, 117.5, 113.1, 108.2, 103.4, 36.7, 27.7, 20.9; **5p’**: δ 197.1, 167.6, 149.9, 147.6, 146.0, 138.5, 135.0, 133.1, 127.6, 121.5, 121.3, 121.1, 120.8, 119.6, 115.8, 113.1, 108.4, 103.1, 36.6, 27.8, 20.9; HRMS (ESI, m/z) calculated for $C_{22}H_{16}N_3O_3$ $(M + H)^+$ 370.1187, found 370.1192.

**6-oxo-6,7,8,9-tetrahydrobenzo[4,5]imidazo[1,2-*f*][1,3]dioxolo[4,5-*k*]phenanthridine-12-carboxylic acid (5q)**

**6-oxo-6,7,8,9-tetrahydrobenzo[4,5]imidazo[1,2-*f*][1,3]dioxolo[4,5-*k*]phenanthridine-13-carboxylic acid (5q’)**

5q 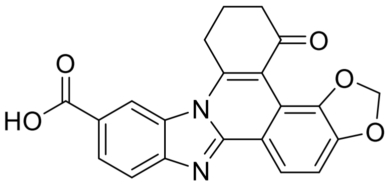

5q' 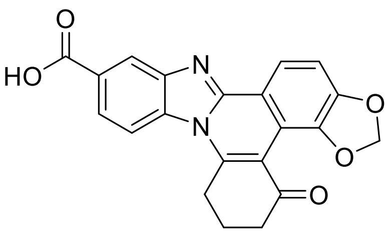

Beige solid, yield: 30.4 mg, 81% as a mixture of **5q**/**5q’** = 1 : 0.87; Eluent: EA/MeOH = 10:1; $^{1}$H NMR (600 MHz, $(CD_3)_2SO$): **5q**: δ 8.65 (d, $J$ = 0.7 Hz, 1H), 8.29 (d, $J$ = 8.3 Hz, 1H), 8.05 (dd, $J$ = 8.5, 1.3 Hz, 1H), 7.87 (d, $J$ = 8.5 Hz, 1H), 7.37 (d, $J$ = 8.4 Hz, 1H), 6.18 (s, 2H), 3.68 (t, $J$ = 6.0 Hz, 2H), 2.69 (t, $J$ = 6.5 Hz, 2H), 2.31 (quin, $J$ = 6.5 Hz, 2H); **5q’**: δ 8.33 (d, $J$ = 1.6 Hz, 1H), 8.28 (d, $J$ = 8.3 Hz, 1H), 8.25 (d, $J$ = 8.9 Hz, 1H), 7.91 (dd, $J$ = 8.8, 1.6 Hz, 1H), 7.35 (d, $J$ = 8.4 Hz, 1H), 6.17 (s, 2H), 3.64 (t, $J$ = 6.0 Hz, 2H), 2.68 (t, $J$ = 6.3 Hz, 2H), 2.28 (quin, $J$ = 6.6 Hz, 2H); $^{13}C\{^{1}H\}$ NMR (151 MHz, $(CD_3)_2SO$): **5q**: δ 192.8, 167.5, 149.8, 149.6, 149.0, 147.7, 142.2, 131.1, 126.1, 124.4, 120.2, 119.0, 117.2, 116.8, 114.8, 112.6, 110.4, 101.6, 36.9, 27.8, 20.6; **5q’**: δ 192.8, 167.4, 149.7, 149.0, 148.8, 144.2, 142.3, 134.2, 127.4, 123.2, 120.5, 120.0, 116.9, 115.6, 114.8, 112.3, 110.2, 101.5, 36.9, 27.9, 20.6; HRMS (ESI, m/z) calculated for $C_{21}H_{15}N_2O_5$

$(M + H)^+$ 375.0976, found 375.0983.

**4-oxo-5-phenyl-1,2,3,4-tetrahydrobenzo[4,5]imidazo[1,2-*a*]quinoline-10-carboxylic acid (5r)**

**4-oxo-5-phenyl-1,2,3,4-tetrahydrobenzo[4,5]imidazo[1,2-*a*]quinoline-9-carboxylic acid (5r')**

**5r** 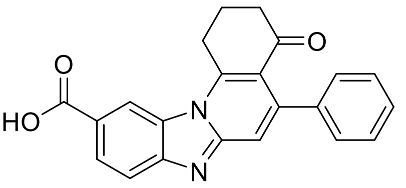

**5r'** 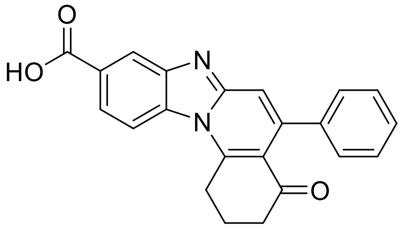

Beige solid, yield: 10.4 mg, 29% as a mixture of **5r**/**5r'** = 1 : 0.49; Eluent: EA/MeOH = 10:1; $^1$H NMR (600 MHz, $(CD_3)_2SO$): **5r**: δ 8.87 (s, 1H), 8.16 (dd, *J* = 8.6, 1.2 Hz, 1H), 7.91 (d, *J* = 8.6 Hz, 1H), 7.41 – 7.33 (m, 5H), 7.40 (s, 1H), 3.85 (t, *J* = 6.1 Hz, 2H), 2.64 (t, *J* = 6.7 Hz, 2H), 2.36 (quin, *J* = 6.4 Hz, 2H); **5r'**: δ 8.46 (d, *J* = 8.9 Hz, 1H), 8.39 (d, *J* = 1.4 Hz, 1H), 8.16 (dd, *J* = 8.6, 1.2 Hz, 1H), 7.41 – 7.33 (m, 5H), 7.36 (s, 1H), 3.82 (t, *J* = 6.0 Hz, 2H), 2.63 (t, *J* = 6.5 Hz, 2H), 2.33 (quin, *J* = 6.7 Hz, 2H); $^{13}C\{^1H\}$ NMR (151 MHz, $(CD_3)_2SO$): **5r**: δ 194.6, 167.5, 152.2, 149.9, 149.0, 144.1, 140.5, 129.7, 128.0, 127.7, 127.3, 126.8, 123.8, 118.9, 118.4, 117.5, 116.3, 37.8, 28.3, 20.4; **5r'**: δ 194.6, 167.5, 152.0, 149.0, 145.5, 143.4, 140.5, 132.6, 128.1, 127.9, 127.7, 127.2, 122.3, 120.6, 117.4, 116.6, 116.3, 37.6, 28.3, 20.4; HRMS (ESI, m/z) calculated for $C_{22}H_{17}N_2O_3$ $(M + H)^+$ 357.1234, found 357.1234.

**5-methyl-4-oxo-1,2,3,4-tetrahydrobenzo[4,5]imidazo[1,2-*a*]quinoline-10-carboxylic acid (5s)**

**5-methyl-4-oxo-1,2,3,4-tetrahydrobenzo[4,5]imidazo[1,2-*a*]quinoline-9-carboxylic acid (5s')**

**5s** 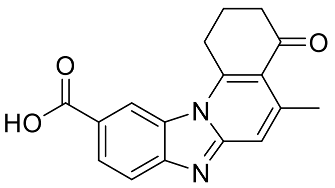

**5s'** 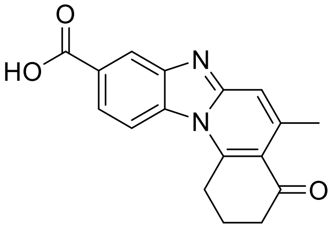

Beige solid, yield: 10.8 mg, 37% as a mixture of **5s**/**5s'** = 1 : 0.69; Eluent: EA/MeOH = 10:1; $^1$H NMR (600 MHz, $(CD_3)_2SO$): **5s**: δ 8.81 (d, *J* = 0.9 Hz, 1H), 8.12 (dd, *J* = 8.6, 1.4 Hz, 1H), 7.85 (d, *J* = 8.5 Hz, 1H), 7.45 (s, 1H), 3.79 (t, *J* = 6.1 Hz, 2H), 2.68 (s, 3H), 2.68 – 2.66 (m, 2H), 2.29 (quin, *J* = 6.6 Hz, 2H); **5s'**: δ 8.40 (d, *J* = 8.9 Hz, 1H), 8.33 (d, *J* = 1.6 Hz, 1H), 7.92 (dd, *J* = 8.8, 1.7 Hz, 1H), 7.40 (s, 1H), 3.75 (t, *J* = 6.2 Hz, 2H), 2.67 – 2.65 (m, 2H), 2.66 (s, 3H), 2.26 (quin, *J* = 6.7 Hz, 2H); $^{13}C\{^1H\}$ NMR (151 MHz, $(CD_3)_2SO$): **5s**: δ 196.8, 167.5, 151.9, 150.2, 149.0, 142.3, 129.7, 126.7, 123.0, 118.6, 118.3, 117.7, 115.4, 38.1, 28.3, 23.1, 20.2; **5s'**: δ 196.8, 167.4, 151.8, 149.3, 145.4, 141.3, 132.7, 128.1, 121.7, 120.3, 117.5, 116.6, 115.4, 38.1, 28.4, 23.1, 20.2; HRMS (ESI, m/z) calculated for $C_{17}H_{15}N_2O_3$ $(M + H)^+$ 295.1077, found 295.1079.

**7-oxo-7,8,9,10-tetrahydrobenzo[4,5]imidazo[1,2-f]phenaleno[1,9-ij]phenanthridine-13-carboxylic acid (5t)**

**7-oxo-7,8,9,10-tetrahydrobenzo[4,5]imidazo[1,2-f]phenaleno[1,9-ij]phenanthridine-14-carboxylic acid (5t')**

**5t**

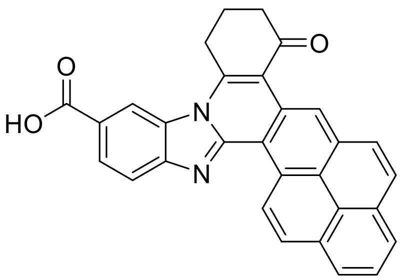

**5t'**

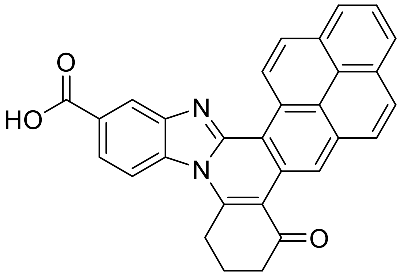

Dark yellow solid, yield: 21.3 mg, 47% as a mixture of **5t**/**5t'** = 1 : 0.67; Eluent: EA/MeOH = 10:1; $^{1}$H NMR (600 MHz, $(CD_3)_2SO$): **5t**: δ 10.80 (d, $J$ = 9.4 Hz, 1H), 9.78 (s, 1H), 8.47 (s, 1H), 8.33 (d, $J$ = 8.5 Hz, 1H), 8.25 (d, $J$ = 7.6 Hz, 1H), 8.22 (d, $J$ = 7.2 Hz, 1H), 8.10 (d, $J$ = 8.6 Hz, 1H), 8.04 (t, $J$ = 7.5 Hz, 1H), 8.02 (d, $J$ = 8.8 Hz, 1H), 8.01 (d, $J$ = 8.4 Hz, 1H), 7.92 (d, $J$ = 8.4 Hz, 1H), 3.53 (t, $J$ = 5.6 Hz, 2H), 2.75 (d, $J$ = 6.3 Hz, 2H), 2.30 (quin, $J$ = 6.1 Hz, 2H); **5t'**: δ 10.80 (d, $J$ = 9.4 Hz, 1H), 9.78 (s, 1H), 8.39 (s, 1H), 8.34 (d, $J$ = 9.1 Hz, 1H), 8.26 (d, $J$ = 7.5 Hz, 1H), 8.22 (d, $J$ = 7.2 Hz, 1H), 8.11 (d, $J$ = 8.2 Hz, 1H), 8.10 (d, $J$ = 8.6 Hz, 1H), 8.04 (t, $J$ = 7.5 Hz, 1H), 8.02 (d, $J$ = 8.8 Hz, 1H), 7.84 (d, $J$ = 8.7 Hz, 1H), 3.57 (t, $J$ = 5.4 Hz, 2H), 2.74 (d, $J$ = 6.6 Hz, 2H), 2.28 (quin, $J$ = 6.2 Hz, 2H); $^{13}C\{^{1}H\}$ NMR (151 MHz, $(CD_3)_2SO$): **5t**: δ 197.3, 167.4, 149.3, 149.0, 147.0, 132.0, 130.4, 129.7, 129.4, 129.3, 128.7, 127.9, 127.6, 127.6, 126.9, 126.5, 125.9, 125.8, 125.8, 124.6, 122.5, 122.3, 122.1, 119.0, 117.5, 114.0, 113.4, 38.6, 28.7, 20.3; **5t'**: δ 197.4, 167.3, 149.5, 148.2, 143.6, 132.5, 131.8, 130.4, 129.7, 129.1, 128.7, 127.9, 127.6, 127.4, 127.4, 126.7, 126.5, 125.9, 125.8, 123.2, 122.6, 122.3, 122.2, 120.7, 115.8, 114.0, 113.6, 38.6, 28.9, 20.3; HRMS (ESI, m/z) calculated for $C_{30}H_{19}N_2O_3$ $(M + H)^+$ 455.1391, found 455.1391.

**9,13-dioxo-6,7,8,9,13,14,15,16-octahydrotribenzo[*b*,*de*,*g*]benzo[4,5]imidazo[1,2,3-*ij*][1,8]naphthyridinium-3-carboxylate (6a)**

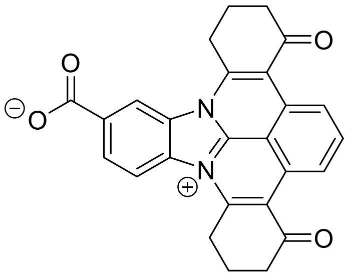

Yellow-orange solid, yield: 8.9 mg, 21%; Eluent: DCM/MeOH/$H_2O$ = 70:10:1; $^{1}$H NMR (600 MHz, $CD_3OD$): δ 9.67 (d, $J$ = 8.2 Hz, 1H), 9.66 (d, $J$ = 8.2 Hz, 1H), 9.37 (s, 1H), 8.89 (d, $J$ = 9.1 Hz, 1H), 8.63 (dd, $J$ = 9.1, 0.5 Hz, 1H), 8.50 (t, $J$ = 8.3 Hz, 1H), 4.18 (t, $J$ = 6.3 Hz, 2H), 4.12 (t, $J$ = 6.2 Hz, 2H), 3.00 (t, $J$ = 6.9 Hz, 2H), 2.99 (t, $J$ = 6.9 Hz, 2H), 2.58 (quin, $J$ = 6.4 Hz, 2H), 2.56 (quin, $J$ = 6.4 Hz, 2H); $^{13}C\{^{1}H\}$ NMR (151 MHz, $CD_3OD$): δ 198.4, 198.4, 171.5, 150.9, 150.8, 141.4, 139.5, 138.3, 132.4, 131.3, 130.7, 130.5, 130.5, 125.5, 125.5, 120.0, 120.0, 119.5, 118.3, 116.0, 39.5, 39.5, 30.3, 30.3, 21.8, 21.7; HRMS (ESI,

m/z) calculated for $C_{26}H_{19}N_2O_4$ $(M + H)^+$ 423.1339, found 423.1338.

**11-methyl-9,13-dioxo-6,7,8,9,13,14,15,16-octahydrotribenzo[*b*,*de*,*g*]benzo[4,5]imidazo[1,2,3-*ij*][1,8]naphthyridinium-3-carboxylate (6c)**

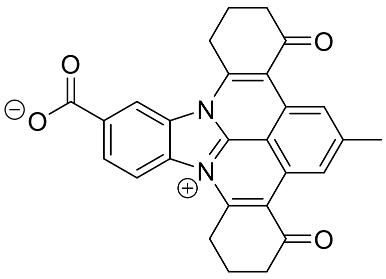

Yellow-orange solid, yield: 6.2 mg, 14%; Eluent: DCM/MeOH/$H_2O$ = 70:10:1; $^1$H NMR (600 MHz, $CD_3OD$): δ 9.49 (s, 1H), 9.49 (s, 1H), 9.31 (s, 1H), 8.84 (d, *J* = 9.0 Hz, 1H), 8.60 (dd, *J* = 9.0, 0.5 Hz, 1H), 8.49 (t, *J* = 8.3 Hz, 1H), 4.13 (t, *J* = 6.2 Hz, 2H), 4.07 (t, *J* = 6.2 Hz, 2H), 2.98 (t, *J* = 7.2 Hz, 2H), 2.97 (t, *J* = 7.0 Hz, 2H), 2.85 (s, 3H), 2.56 (quin, *J* = 6.4 Hz, 2H), 2.54 (quin, *J* = 6.3 Hz, 2H); $^{13}$C{$^1$H} NMR (151 MHz, $CD_3OD$): δ 198.5, 198.4, 171.5, 151.0, 150.9, 150.9, 141.1, 139.5, 132.3, 131.2, 130.6, 130.4, 130.4, 126.0, 126.0, 119.6, 119.6, 119.4, 118.2, 114.2, 39.6, 39.5, 30.3, 30.2, 24.1, 21.7, 21.7; HRMS (ESI, m/z) calculated for $C_{27}H_{21}N_2O_4$ $(M + H)^+$ 437.1496, found 437.1487.

**9,14-dioxo-6,7,8,9,14,15,16,17-octahydro-[1,3]dioxolo[4',5':4,5]benzo[1,2,3-*de*]benzo[b]benzo[g]benzo[4,5]imidazo[1,2,3-*ij*][1,8]naphthyridinium-3-carboxylate (6q)**
**4,17-dioxo-4,5,6,7,14,15,16,17-octahydro-[1,3]dioxolo[4',5':4,5]benzo[1,2,3-*de*]benzo[b]benzo[g]benzo[4,5]imidazo[1,2,3-*ij*][1,8]naphthyridinium-10-carboxylate (6q')**

6q

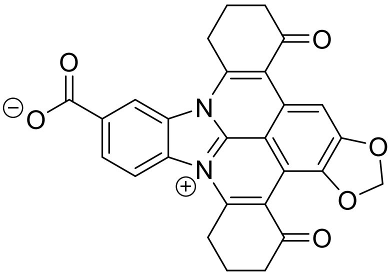

6q'

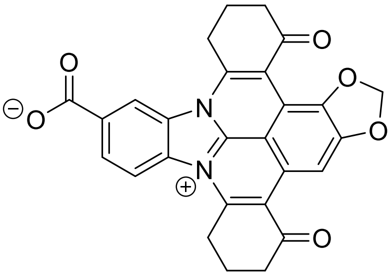

Yellow solid, yield: 8.1 mg, 17% as a mixture of **6q**/**6q'** = 1 : 0.82; Eluent: DCM/MeOH/$H_2O$ = 70:10:1; $^1$H NMR (600 MHz, $CD_3OD$): **6q**: δ 9.22 (d, *J* = 0.8 Hz, 1H), 9.12 (s, 1H), 8.71 (d, *J* = 8.9 Hz, 1H), 8.52 (dd, *J* = 8.9, 1.2 Hz, 1H), 6.36 (s, 2H), 4.06 (t, *J* = 6.2 Hz, 2H), 3.92 (t, *J* = 6.2 Hz, 2H), 2.93 (t, *J* = 7.0 Hz, 2H), 2.91 (t, *J* = 7.2 Hz, 2H), 2.51 (quin, *J* = 5.9 Hz, 2H), 2.50 (quin, *J* = 6.1 Hz, 2H); **6q'**: δ 9.16 (d, *J* = 0.7 Hz, 1H), 9.12 (s, 1H), 8.76 (d, *J* = 9.0 Hz, 1H), 8.50 (dd, *J* = 8.9, 1.1 Hz, 1H), 6.36 (s, 2H), 4.01 (t, *J* = 6.2 Hz, 2H), 3.97 (t, *J* = 6.1 Hz, 2H), 2.94 (t, *J* = 7.1 Hz, 2H), 2.93 (t, *J* = 7.0 Hz, 2H), 2.52 (quin, *J* = 5.9 Hz, 2H), 2.51 (quin, *J* = 5.9 Hz, 2H); $^{13}$C{$^1$H} NMR (151 MHz, $CD_3OD$): **6q**: δ 198.5, 195.0, 171.5, 157.9, 149.8, 149.7, 142.9, 141.0, 138.9, 132.1, 131.0, 130.5, 129.1, 120.3, 119.5, 118.7, 117.5, 113.1, 110.2, 106.6, 104.9, 39.5, 38.2, 30.2, 29.5, 22.1, 21.7; **6q'**: δ 198.5, 195.0, 171.5, 157.8, 149.8, 149.7, 142.9, 141.1, 139.5, 132.1, 131.0, 129.8, 128.9, 120.3, 119.6, 119.5, 118.3, 113.1, 110.5, 106.6, 104.9, 39.5, 38.2, 30.1, 29.5, 22.1,

21.6; HRMS (ESI, m/z) calculated for $C_{27}H_{19}N_2O_6$ (M + H)$^+$ 467.1238, found 467.1237.

**7-(2-hydroxy-6-oxocyclohex-1-en-1-yl)-9-(trifluoromethyl)-2,3,4-trihydrobenzo[3,4]isochromeno[6,7-*d*]imidazole-1,6-dione (7f)**

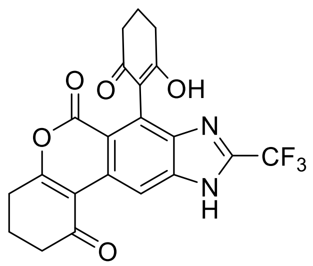


White solid, yield: 6.7 mg, 16%; Eluent: EA/DCM = 2:1; $^1$H NMR (400 MHz, $CD_3OD$): δ 9.51 (s, 1H), 2.94 (t, *J* = 6.3 Hz, 2H), 2.69 (t, *J* = 6.7 Hz, 2H), 2.67 – 2.58 (m, 4H), 2.31 – 2.22 (m, 2H), 2.17 (quin, *J* = 6.4 Hz, 2H); $^{13}$C{$^1$H} NMR (101 MHz, $CD_3OD$): δ 199.3, 170.3, 160.3, 132.4, 120.0 (q, *J* = 272 Hz), 117.0, 114.0, 112.3, 40.0, 34.3 (br), 29.7, 21.5, 21.0; $^{19}$F NMR (376 MHz, $CD_3OD$): δ -66.1; HRMS (ESI, m/z) calculated for $C_{21}H_{16}F_3N_2O_5$ (M + H)$^+$ 433.1006, found 433.1007.

**9-(*tert*-butyl)-7-(2-hydroxy-6-oxocyclohex-1-en-1-yl)-2,3,4-trihydrobenzo[3,4]isochromeno[6,7-*d*]imidazole-1,6-dione (7g)**

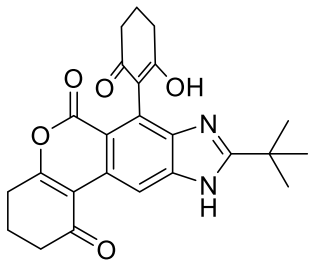


White solid, yield: 5.2 mg, 12%; Eluent: EA; $^1$H NMR (400 MHz, $CD_3OD$): δ 9.36 (s, 1H), 2.92 (t, *J* = 6.1 Hz, 2H), 2.68 – 2.57 (m, 6H), 2.29 – 2.21 (m, 2H), 2.15 (quin, *J* = 6.5 Hz, 2H), 1.50 (s, 9H); $^{13}$C{$^1$H} NMR (151 MHz, $CD_3OD$): δ 199.5, 169.4, 169.0, 161.1, 130.9, 114.7, 114.5, 112.8, 40.1, 35.0, 34.3 (br), 29.7, 29.5, 21.4, 21.2; HRMS (ESI, m/z) calculated for $C_{24}H_{25}N_2O_5$ (M + H)$^+$ 421.1758, found 421.1758.

**9-(2,6-dichlorophenyl)-7-(2-hydroxy-6-oxocyclohex-1-en-1-yl)-2,3,4-trihydrobenzo[3,4]isochromeno[6,7-*d*]imidazole-1,6-dione (7i)**

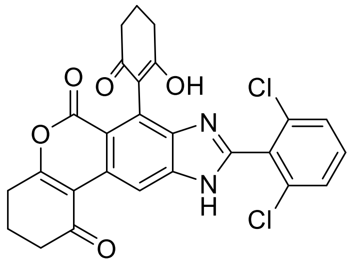


White solid, yield: 7.1 mg, 14%; Eluent: EA; $^1$H NMR (400 MHz, $CD_3OD$): δ 9.47 (s, 1H), 7.61 – 7.54 (m, 3H), 2.94 (t, *J* = 6.3 Hz, 2H), 2.69 (t, *J* = 6.7 Hz, 2H), 2.63 (t, *J* = 6.4 Hz, 4H), 2.24 – 2.14 (m, 4H); $^{13}$C{$^1$H} NMR (101 MHz, $CD_3OD$): δ 199.5, 169.7, 160.9, 153.1, 137.0, 133.7, 131.3, 130.8. 129.4, 115.7, 114.4, 113.3 (br), 112.7, 40.1, 34.1 (br), 29.7, 21.5, 21.1; HRMS (ESI, m/z) calculated for $C_{26}H_{19}Cl_2N_2O_5$ (M + H)$^+$ 509.0666, found 509.0665.

**9-benzyl-7-(2-hydroxy-6-oxocyclohex-1-en-1-yl)-2,3,4-trihydrobenzo[3,4]isochromeno[7,6-*d*]oxazole-1,6-dione (7x)**

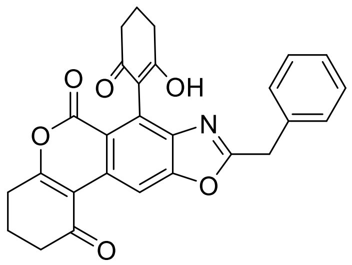


Pale orange solid, yield: 11.2 mg, 25%; Eluent: EA/DCM = 1:1; $^{1}H$ NMR (600 MHz, $CD_3OD$): δ 9.26 (s, 1H), 7.36 (d, *J* = 7.3 Hz, 2H), 7.31 (t, *J* = 7.6 Hz, 2H), 7.25 (t, *J* = 7.3 Hz, 1H), 4.31 (s, 2H), 2.87 (t, *J* = 6.3 Hz, 2H), 2.66 – 2.57 (m, 6H), 2.24 – 2.16 (m, 2H), 2.09 (quin, *J* = 6.5 Hz, 2H); $^{13}C\{^{1}H\}$ NMR (151 MHz, $CD_3OD$): δ 197.7, 169.8, 168.1, 158.5, 154.3, 140.9, 134.2, 133.0. 130.3, 128.6, 128.5, 127.1, 116.1, 112.6, 110.8, 106.3, 38.5, 34.3, 32.7 (br), 28.3, 20.1, 19.5; HRMS (ESI, m/z) calculated for $C_{27}H_{22}NO_6$ $(M + H)^+$ 456.1442, found 456.1439.

**7-(2-hydroxy-6-oxocyclohex-1-en-1-yl)-9-phenyl-2,3,4-trihydrobenzo[3,4]isochromeno[7,6-*d*]oxazole-1,6-dione (7y)**

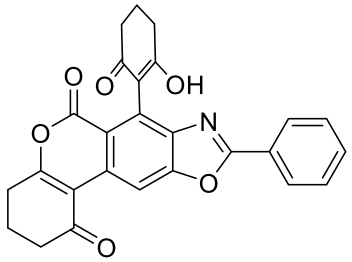


White solid, yield: 7.7 mg, 17%; Eluent: EA/DCM = 1:1; $^{1}H$ NMR (600 MHz, $(CD_3)_2SO$): δ 9.29 (s, 1H), 8.22 (d, *J* = 7.2 Hz, 2H), 7.68 (t, *J* = 7.3 Hz, 1H), 7.64 (t, *J* = 7.4 Hz, 2H), 2.91 (br, 2H), 2.65 (br, 2H), 2.52 (br, 2H), 2.11 – 2.06 (m, 4H); $^{13}C\{^{1}H\}$ NMR (151 MHz, $(CD_3)_2SO$): δ 163.7, 157.5, 153.5, 144.7, 132.6, 132.5. 132.3, 129.4, 127.7, 125.7, 116.0, 112.3, 110.3, 105.3, 38.5 (br), 28.3 (br), 20.5, 19.4; HRMS (ESI, m/z) calculated for $C_{26}H_{20}NO_6$ $(M + H)^+$ 442.1285, found 442.1287.

**2-(6'-hydroxy-2'-oxo-3-(trifluoromethyl)-2',3',4',5'-tetrahydro-[1,1'-biphenyl]2-yl)-benzimidazole-5-carboxylic acid (8h)**

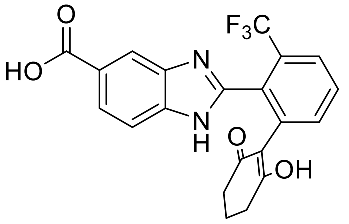


White solid, yield: 16.5 mg, 40%; Eluent: EA/MeOH/$H_2O$ = 70:10:1; $^{1}H$ NMR (600 MHz, $(CD_3)_2SO$): δ 8.12 (s, 1H), 7.80 (dd, *J* = 8.5, 1.7 Hz, 1H), 7.79 (d, *J* = 8.5 Hz, 1H), 7.69 (t, *J* = 7.9 Hz, 1H), 7.56 (d, *J* = 8.4 Hz, 1H), 7.44 (d, *J* = 7.7 Hz, 1H), 2.25 – 2.19 (m, 2H), 1.99 – 1.94 (m, 2H), 1.72 – 1.66 (m, 1H), 1.31 – 1.25 (m, 1H); $^{13}C\{^{1}H\}$ NMR (151 MHz, $(CD_3)_2SO$): δ 167.9, 150.8, 138.9, 136.2, 130.6, 129.6, 128.6 (q, *J* = 30 Hz), 124.5 (q, *J* = 5 Hz), 124.1, 124.0 (q, *J* = 275 Hz), 123.1, 117.1 (br), 114.5, 114.5 (br), 32.8 (br), 20.4; $^{19}F$ NMR (564 MHz, $CD_3OD$): δ -60.0 (br); HRMS (ESI, m/z) calculated for $C_{21}H_{16}F_3N_2O_4$ $(M + H)^+$ 417.1057, found 417.1057.

**13-phenyl-1,2,3,4,9,10,11,12-octahydrobenzo[3',4']isochromeno[6',7':4,5]imidazo[1,2-*a*]quinoline-1,6,12-trione (9r)**

**10-phenyl-1,2,3,4,11,12,13,14-octahydrobenzo[3’,4’]isochromeno[7’,6’:4,5]imidazo[1,2-*a*]quinoline-1,6,11-trione (9r’)**

9r

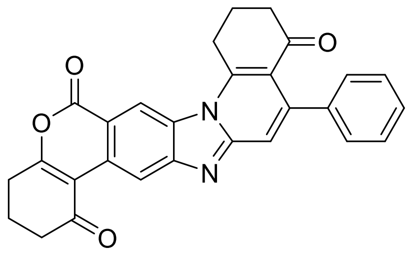

9r'

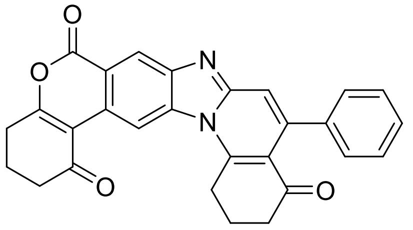

Yellow solid, yield: 6.3 mg, 14% as a mixture of **9r**/**9r’** = 1 : 0.31; Eluent: EA/DCM = 1:3; $^{1}$H NMR (600 MHz, $CDCl_3$): **9r**: δ 9.50 (s, 1H), 8.80 (s, 1H), 7.44 – 7.40 (m, 3H), 7.35 (s, 1H), 7.30 – 7.26 (m, 2H), 3.70 (t, *J* = 6.1 Hz, 2H), 2.93 (t, *J* = 6.3 Hz, 2H), 2.74 (t, *J* = 6.8 Hz, 2H), 2.69 (t, *J* = 6.4 Hz, 2H), 2.50 (quin, *J* = 6.4 Hz, 2H), 2.17 (quin, *J* = 6.5 Hz, 2H); **9r’**: δ 10.11 (s, 1H), 8.83 (s, 1H), 7.44 (s, 1H), 7.44 – 7.40 (m, 3H), 7.30 – 7.26 (m, 2H), 3.88 (t, *J* = 6.0 Hz, 2H), 2.99 (t, *J* = 6.3 Hz, 2H), 2.74 (t, *J* = 6.8 Hz, 2H), 2.73 (t, *J* = 6.4 Hz, 2H), 2.50 (quin, *J* = 6.4 Hz, 2H), 2.23 (quin, *J* = 6.5 Hz, 2H); $^{13}C\{^{1}H\}$ NMR (151 MHz, $CDCl_3$): **9r**: δ 197.1, 194.3, 168.9, 161.1, 152.0, 151.5, 150.7, 146.4, 140.0, 131.1, 129.6, 128.2, 128.1, 127.9, 118.5, 117.7, 117.4, 116.7, 113.5, 111.5, 39.0, 38.1, 29.1, 28.9, 20.9, 20.0; **9r’**: δ 198.1, 194.3, 168.6, 160.5, 151.2, 151.0, 145.7, 145.6, 140.3, 135.5, 128.2, 128.0, 127.8, 126.9, 121.6, 118.8, 118.2, 118.1, 112.9, 111.6, 39.3, 38.1, 29.2, 29.0, 21.2, 20.2; HRMS (ESI, m/z) calculated for $C_{28}H_{21}N_2O_4$ $(M + H)^+$ 449.1496, found 449.1496.

**7-(2-(indol-3-yl)ethyl)-2-benzyl-8,9,10-trihydroimidazo[4,5-*k*]phenanthridine-6,11-dione (10)**

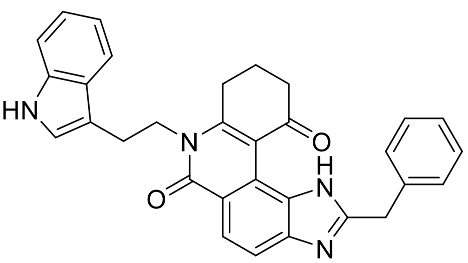

White solid, yield: 40.7 mg, 84%; Eluent: EA/DCM = 1:2; $^{1}$H NMR (600 MHz, $(CD_3)_2SO$): δ 12.07 (s, 1H), 10.88 (d, *J* = 2.4 Hz, 1H), 8.20 (d, *J* = 8.6 Hz, 1H), 7.75 (d, *J* = 8.6 Hz, 1H), 7.62 (d, *J* = 7.9 Hz, 1H), 7.40 (d, *J* = 7.3 Hz, 2H), 7.36 (d, *J* = 8.1 Hz, 1H), 7.34 (t, *J* = 7.7 Hz, 2H), 7.25 (t, *J* = 7.4 Hz, 1H), 7.19 (d, *J* = 2.1 Hz, 1H), 7.07 (t, *J* = 7.6 Hz, 1H), 6.96 (t, *J* = 7.4 Hz, 1H), 4.39 (t, *J* = 7.7 Hz, 2H), 4.33 (s, 2H), 3.11 (t, *J* = 7.8 Hz, 2H), 3.02 (t, *J* = 5.9 Hz, 2H), 2.53 (t, *J* = 6.6 Hz, 2H), 1.90 (quin, *J* = 6.2 Hz, 2H); $^{13}C\{^{1}H\}$ NMR (101 MHz, $(CD_3)_2SO$): δ 196.7, 161.6, 157.0, 154.3, 147.1, 137.5, 136.2, 128.9, 128.6, 127.9, 127.1, 126.6, 123.3, 121.2, 121.1, 120.6, 118.8, 118.6, 118.5, 118.3, 111.5, 110.8, 110.7, 45.2, 37.5, 34.6, 28.1, 23.8, 19.9; HRMS (ESI, m/z) calculated for $C_{31}H_{27}N_4O_2$ $(M + H)^+$ 487.2129, found 487.2127.

**2,7-dibenzyl-8,9,10-trihydroimidazo[4,5-*k*]phenanthridine-6,11-dione (11)**

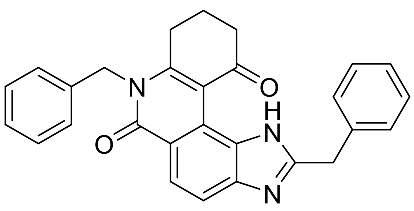

White solid, yield: 19.1 mg, 44%; Eluent: EA/DCM = 1:3; $^{1}$H NMR (400 MHz, $(CD_3)_2SO$): δ 12.10 (s, 1H), 8.17 (d, *J* = 8.7 Hz, 1H), 7.76 (d, *J* = 8.6 Hz, 1H), 7.40 (d, *J* = 7.0 Hz, 2H), 7.34 (t, *J* = 7.4 Hz, 2H), 7.34 (t, *J* = 7.4 Hz, 2H), 7.26 (t, *J* = 7.4 Hz, 1H), 7.24 (t, *J* = 7.4 Hz, 1H), 7.16 (d, *J* = 7.1 Hz, 2H), 5.53 (s, 2H), 4.35 (s, 2H), 3.00 (t, *J* = 5.5 Hz, 2H), 2.62 (t, *J* = 6.6 Hz, 2H), 1.98 (quin, *J* = 6.2 Hz, 2H); $^{13}C\{^{1}H\}$ NMR (101 MHz, $(CD_3)_2SO$): δ 196.9, 162.0, 156.9, 154.6, 147.3, 137.5, 136.6, 128.9, 128.9, 128.6, 128.1, 127.3, 126.7, 126.0, 121.5, 120.7, 119.0, 118.5, 111.3, 46.9, 37.6, 34.6, 28.4, 20.0; HRMS (ESI, m/z) calculated for $C_{28}H_{24}N_3O_2$ $(M + H)^+$ 434.1863, found 434.1864.

**5,9-dibenzyl-2,3,4-trihydroimidazo[4,5-*j*]phenanthridine-1,6-dione (12)**

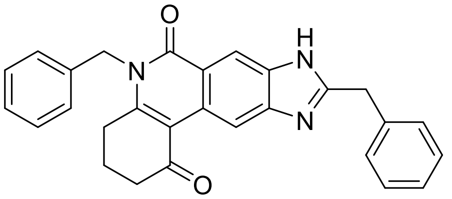

White solid, yield: 11.8 mg, 27%; Eluent: EA/DCM = 1:1; $^{1}$H NMR (400 MHz, $(CD_3)_2SO$): δ 12.70 (br, 1H), 9.41 (s, 0.3H), 9.33 (s, 0.7H), 8.50 (s, 0.7H), 8.36 (s, 0.3H), 7.39 – 7.32 (m, 2H), 7.28 – 7.23 (m, 2H), 7.15 (d, *J* = 7.5 Hz, 2H), 5.49 (s, 2H), 4.26 (s, 2H), 2.96 (t, *J* = 4.9 Hz, 2H), 2.52 (t, *J* = 6.3 Hz, 2H), 1.94 (quin, *J* = 6.2 Hz, 2H); $^{13}$C NMR (101 MHz, $(CD_3)_2SO$): δ 196.9, 162.5, 159.0, 158.0, 153.9, 153.2, 148.2, 142.7, 139.6, 137.0, 137.0, 134.1, 129.0, 128.8, 128.6, 128.3, 127.7, 127.2, 126.8, 125.9, 119.1, 118.7, 117.0, 114.6, 110.9, 110.6, 109.3, 106.8, 46.6, 38.4, 35.2, 27.9, 20.5; HRMS (ESI, m/z) calculated for $C_{28}H_{24}N_3O_2$ $(M + H)^+$ 434.1863, found 434.1867.

**2-benzyl-11-hydroxy-8,9,10,11-tetrahydrobenzo[3,4]isochromeno[5,6-*d*]imidazol-6-one (13)**

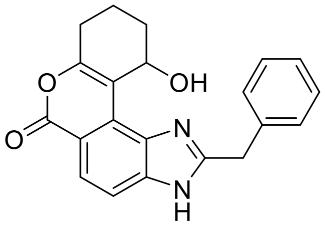

White solid, yield: 8.7 mg, 50%; Eluent: EA/DCM = 1:4; $^{1}$H NMR (600 MHz, $(CD_3)_2SO$): δ 13.19 (s, 1H), 7.99 (d, *J* = 8.6 Hz, 1H), 7.63 (d, *J* = 8.6 Hz, 1H), 7.36 – 7.32 (m, 4H), 7.28 – 7.25 (m, 1H), 6.45 (d, *J* = 4.5 Hz, 1H), 5.24 (q, *J* = 4.0 Hz, 1H), 4.32 (s, 2H), 2.62 – 2.53 (m, 2H), 1.96 – 1.83 (m, 3H), 1.80 – 1.76 (m, 1H); $^{13}C\{^{1}H\}$ NMR (151 MHz, $(CD_3)_2SO$): δ 161.6, 155.4, 154.4, 138.5, 136.8, 135.9, 129.5, 128.7, 128.7, 126.8, 123.4, 113.8, 112.4, 111.8, 62.2, 34.7, 30.2, 27.8, 17.4; HRMS (ESI, m/z) calculated for $C_{21}H_{19}N_2O_3$ $(M + H)^+$ 347.1391, found 347.1402.

**2-benzyl-8,9,10,11-tetrahydrobenzo[3,4]isochromeno[5,6-*d*]imidazol-6-one (14)**

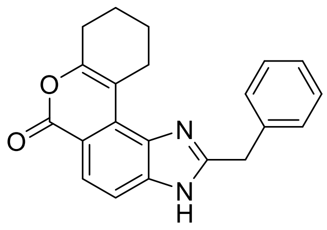

White solid, yield: 2.7 mg, 20%; Eluent: EA/DCM = 1:5; $^{1}H$ NMR (600 MHz, $(CD_3)_2SO$): δ 7.96 (d, *J* = 8.5 Hz, 1H), 7.57 (d, *J* = 8.6 Hz, 1H), 7.35 – 7.31 (m, 4H), 7.25 – 7.23 (m, 1H), 4.27 (s, 2H), 3.31 (br, 2H), 2.59 – 2.57 (m, 2H), 1.84 – 1.81 (m, 4H); $^{13}C\{^{1}H\}$ NMR (151 MHz, $(CD_3)_2SO$): δ 162.1, 152.0, 137.3, 128.6, 128.5, 126.6, 122.8, 113.2, 34.9, 27.2, 25.8, 22.0, 21.4; HRMS (ESI, m/z) calculated for $C_{21}H_{19}N_2O_2$ $(M + H)^+$ 331.1442, found 331.1448.

**2-benzyl-8,9-dihydrobenzo[3,4]isochromeno[5,6-*d*]imidazol-6-one (15)**

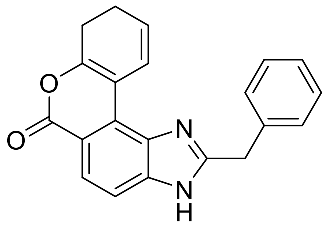

White solid, yield: 3.0 mg, 23%; Eluent: EA/DCM = 1:6; $^{1}H$ NMR (400 MHz, $(CD_3)_2SO$): δ 12.95 (br, 1H), 8.20 (dt, *J* = 9.8, 1.8 Hz, 1H), 7.97 (d, *J* = 8.6 Hz, 1H), 7.59 (d, *J* = 8.6 Hz, 1H), 7.34 – 7.33 (m, 4H), 7.28 – 7.23 (m, 1H), 5.90 (dt, *J* = 9.7, 4.4 Hz, 1H), 4.29 (s, 2H), 2.77 (d, *J* = 9.6 Hz, 1H), 2.46 (tdd, *J* = 9.6, 4.4, 1.7 Hz, 1H); $^{13}C\{^{1}H\}$ NMR (151 MHz, $(CD_3)_2SO$): δ 161.7, 154.6, 153.0, 139.2, 137.4, 137.1, 128.6, 128.6, 127.2, 126.7, 123.1, 123.1, 122.6, 113.1, 111.6, 108.7, 34.9, 25.2, 22.0; HRMS (ESI, m/z) calculated for $C_{21}H_{17}N_2O_2$ $(M + H)^+$ 329.1285, found 329.1293.

***N*,2-dibenzylbenzimidazole-5-carboxamide (17)**

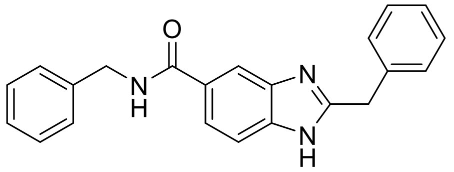

White solid, yield: 74 mg, 43%; Eluent: EA/DCM = 2:1; $^{1}H$ NMR (400 MHz, $(CD_3)_2SO$): δ 12.56 (s, 0.5H), 12.49 (s, 0.5H), 8.99 (t, *J* = 6.2 Hz, 0.5H), 8.97 (t, *J* = 6.3 Hz, 0.5H), 8.15 (s, 0.5H), 7.99 (s, 0.5H), 7.75 (dd, *J* = 8.6, 1.3 Hz, 0.5H), 7.72 (dd, *J* = 8.6, 1.3 Hz, 0.5H), 7.57 (d, *J* = 8.4 Hz, 0.5H), 7.46 (d, *J* = 8.4 Hz, 0.5H), 7.33 – 7.30 (m, 8H), 7.27 – 7.21 (m, 2H), 4.50 (d, *J* = 6.0 Hz, 2H), 4.21 (s, 1H), 4.20 (s, 1H); $^{13}C\{^{1}H\}$ NMR (101 MHz, $(CD_3)_2SO$): δ 166.8, 166.6, 155.9, 155.2, 145.6, 142.9, 140.0, 140.0, 137.4, 137.4, 136.6, 134.1, 128.8, 128.7, 128.2, 128.0, 127.6, 127.1, 126.6, 126.6, 121.5, 120.5, 117.6, 110.6, 110.5, 42.6, 35.0, 35.0; HRMS (ESI, m/z) calculated for $C_{22}H_{20}N_3O$ $(M + H)^+$ 342.1601, found 342.1606.

## NMR Spectra

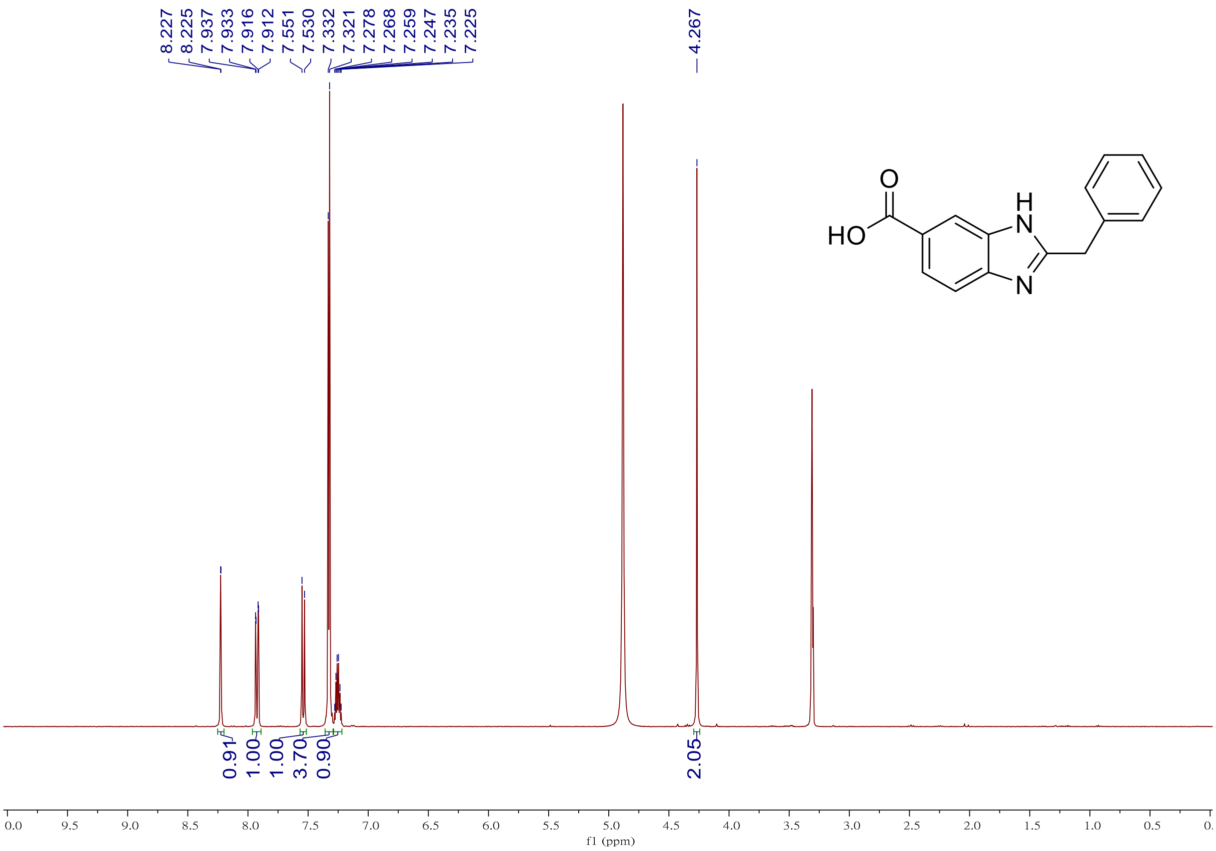


**Figure S3.** $^{1}$H NMR spectrum of compound **1a** (400 MHz, $CD_3OD$).

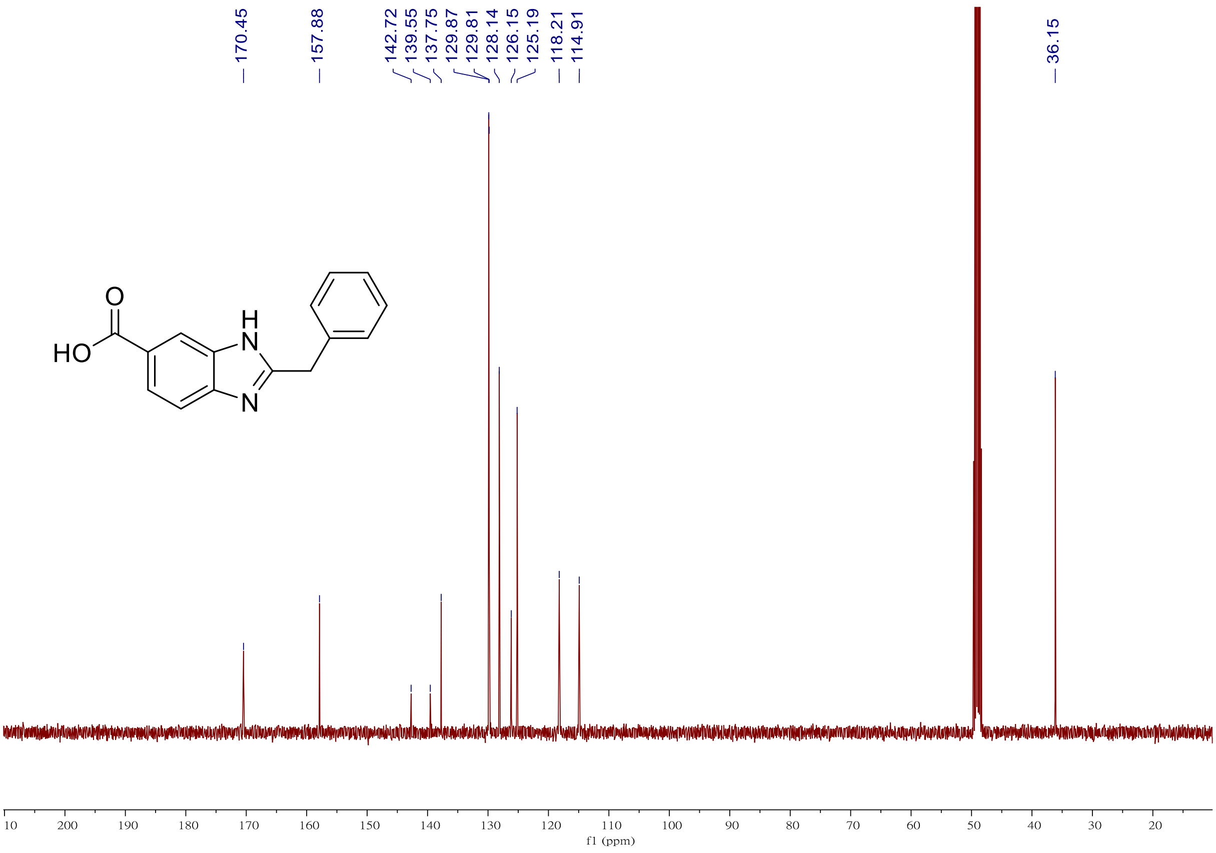


**Figure S4.** $^{13}C\{^{1}H\}$ NMR spectrum of compound **1a** (101 MHz, $CD_3OD$).

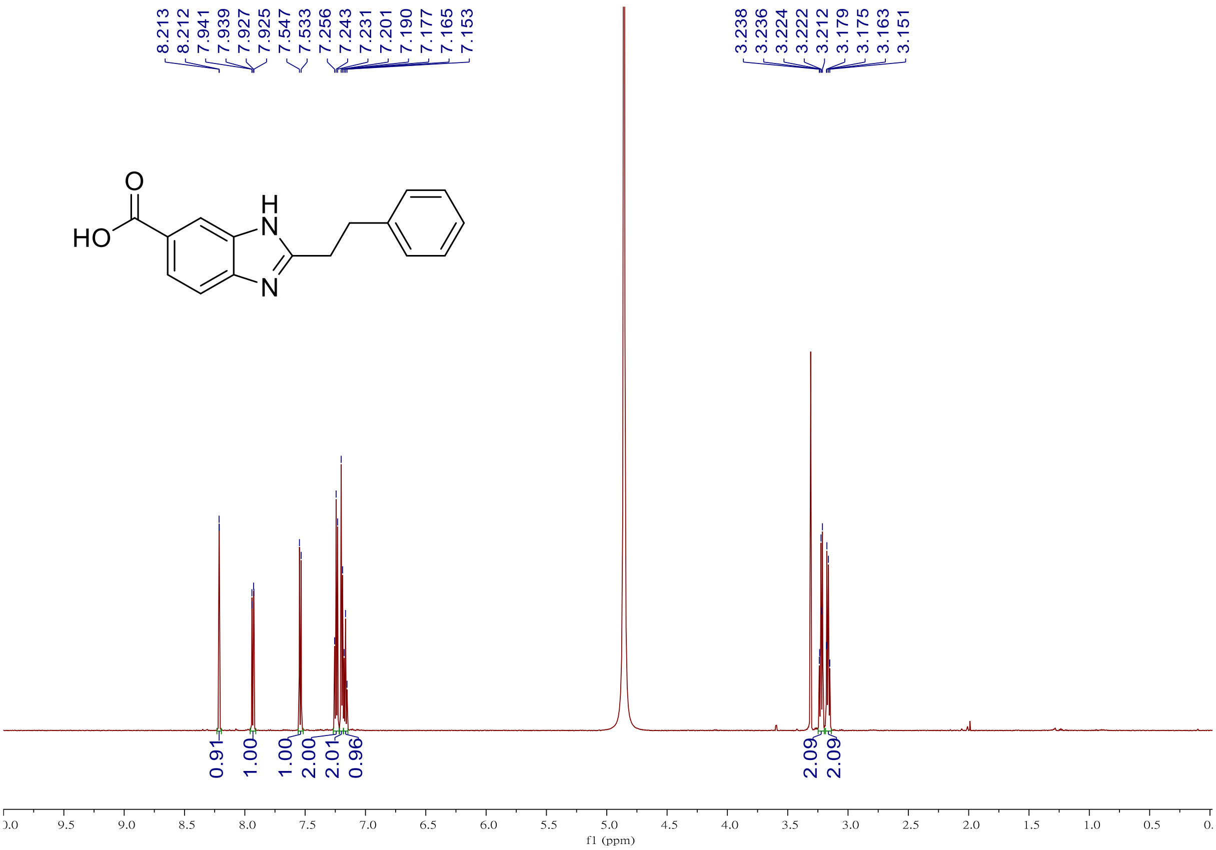


**Figure S5.** $^{1}$H NMR spectrum of compound **1b** (600 MHz, $CD_3OD$).

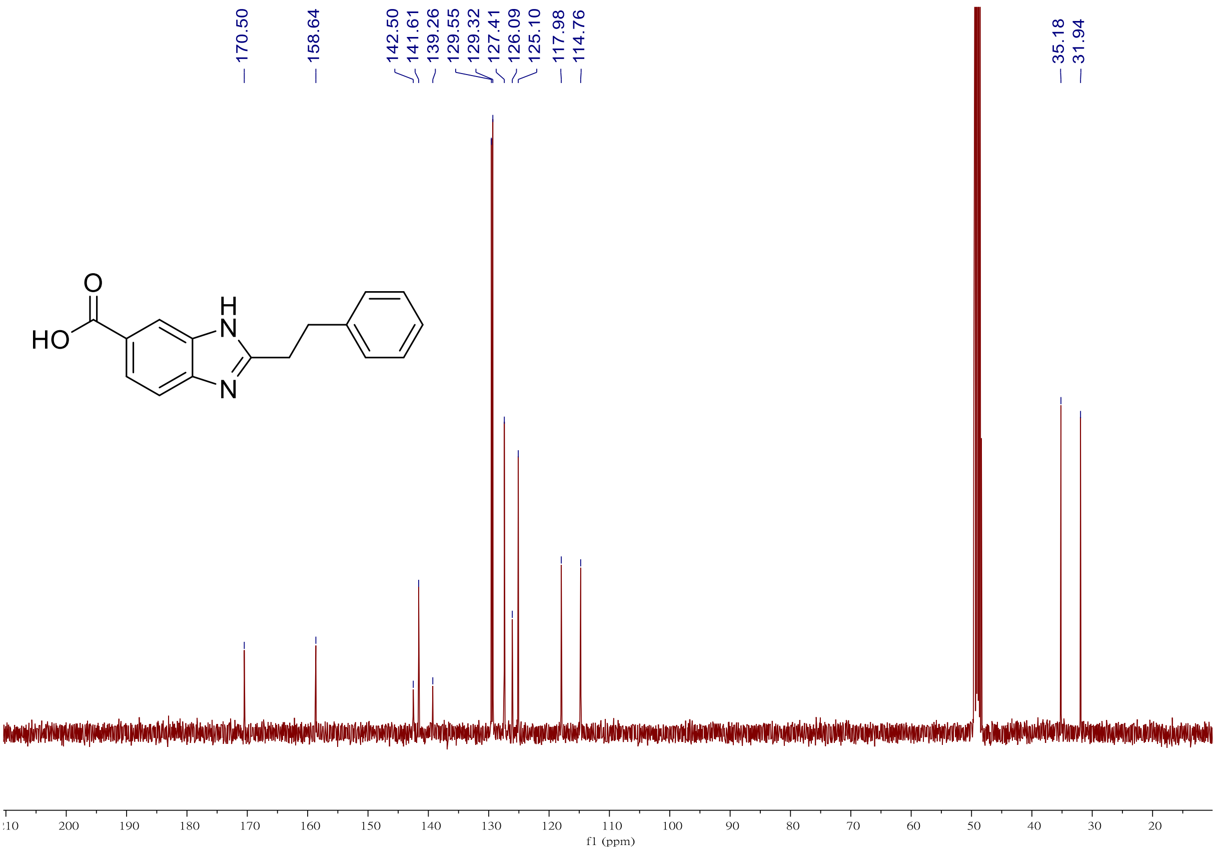


**Figure S6.** $^{13}C\{^{1}H\}$ NMR spectrum of compound **1b** (101 MHz, $CD_3OD$).

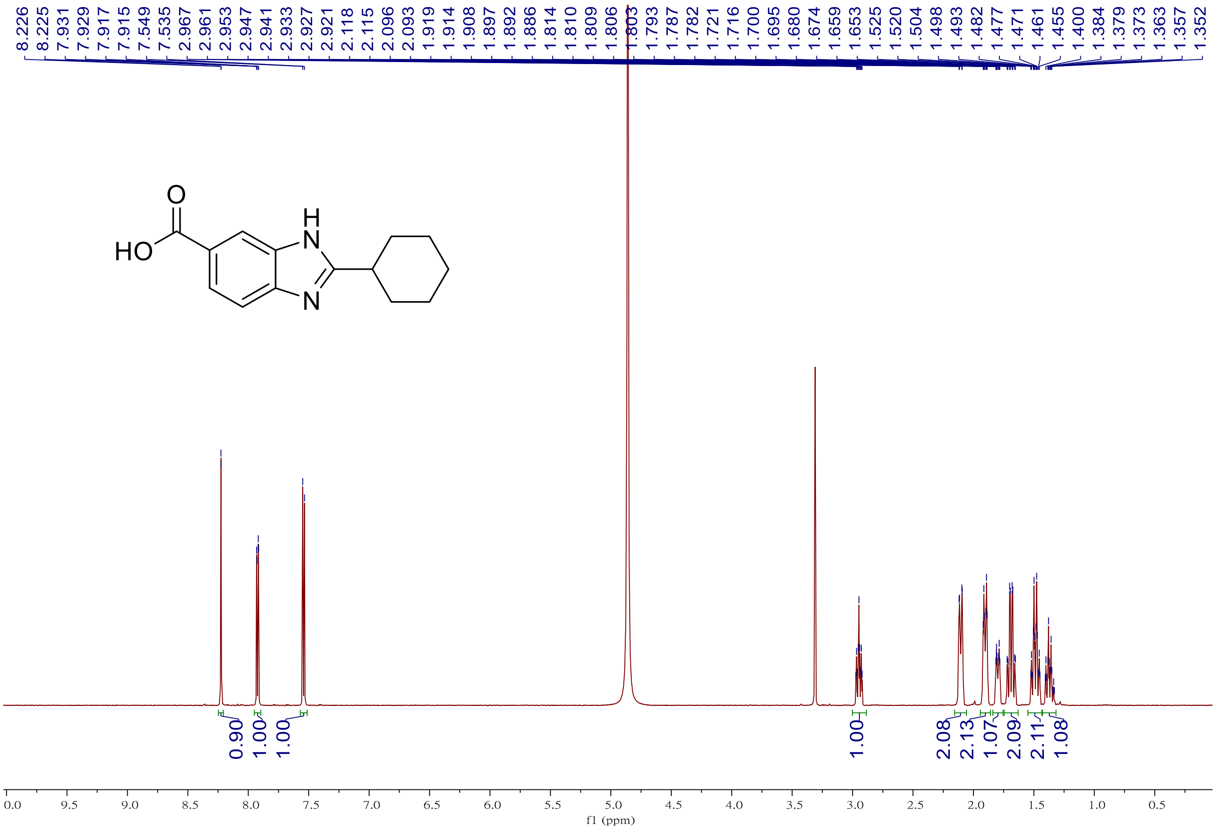


**Figure S7.** $^{1}H$ NMR spectrum of compound **1c** (600 MHz, $CD_3OD$).

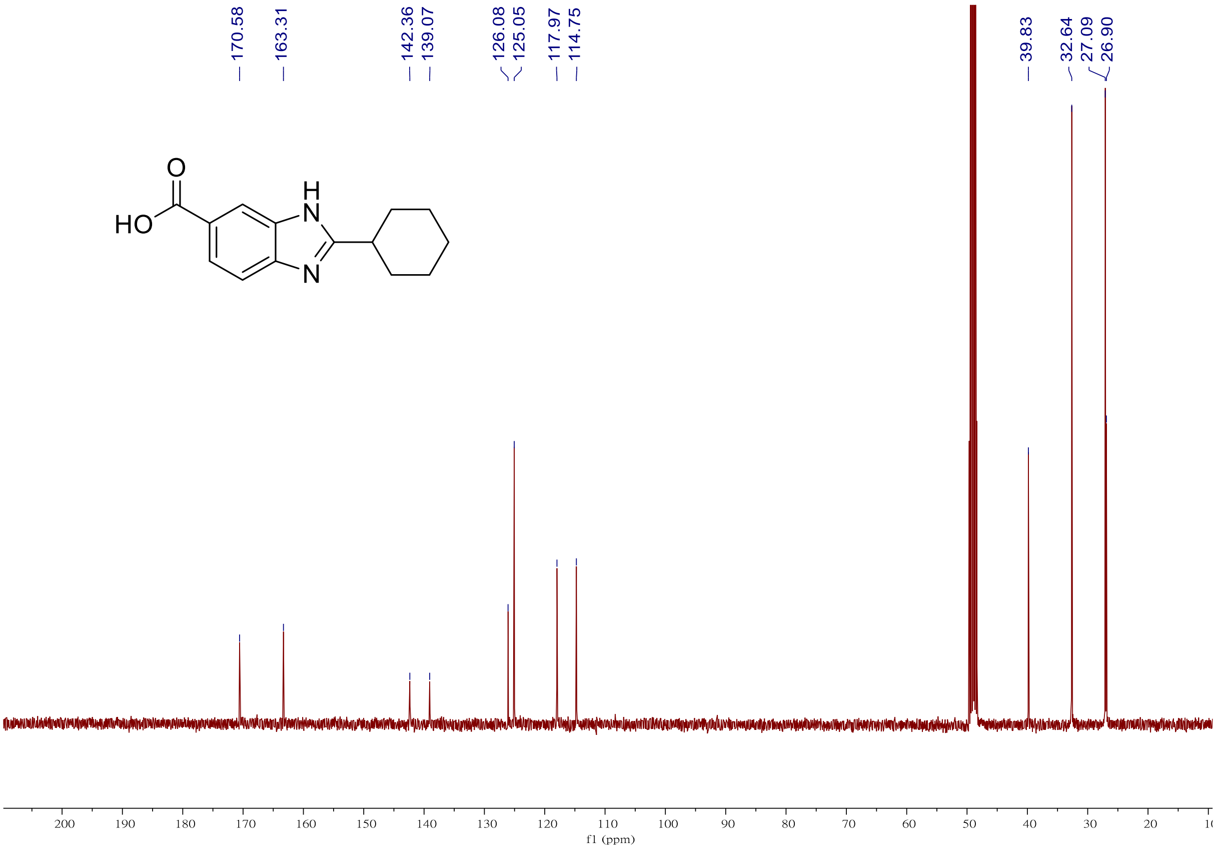


**Figure S8.** $^{13}C\{^{1}H\}$ NMR spectrum of compound **1c** (101 MHz, $CD_3OD$).

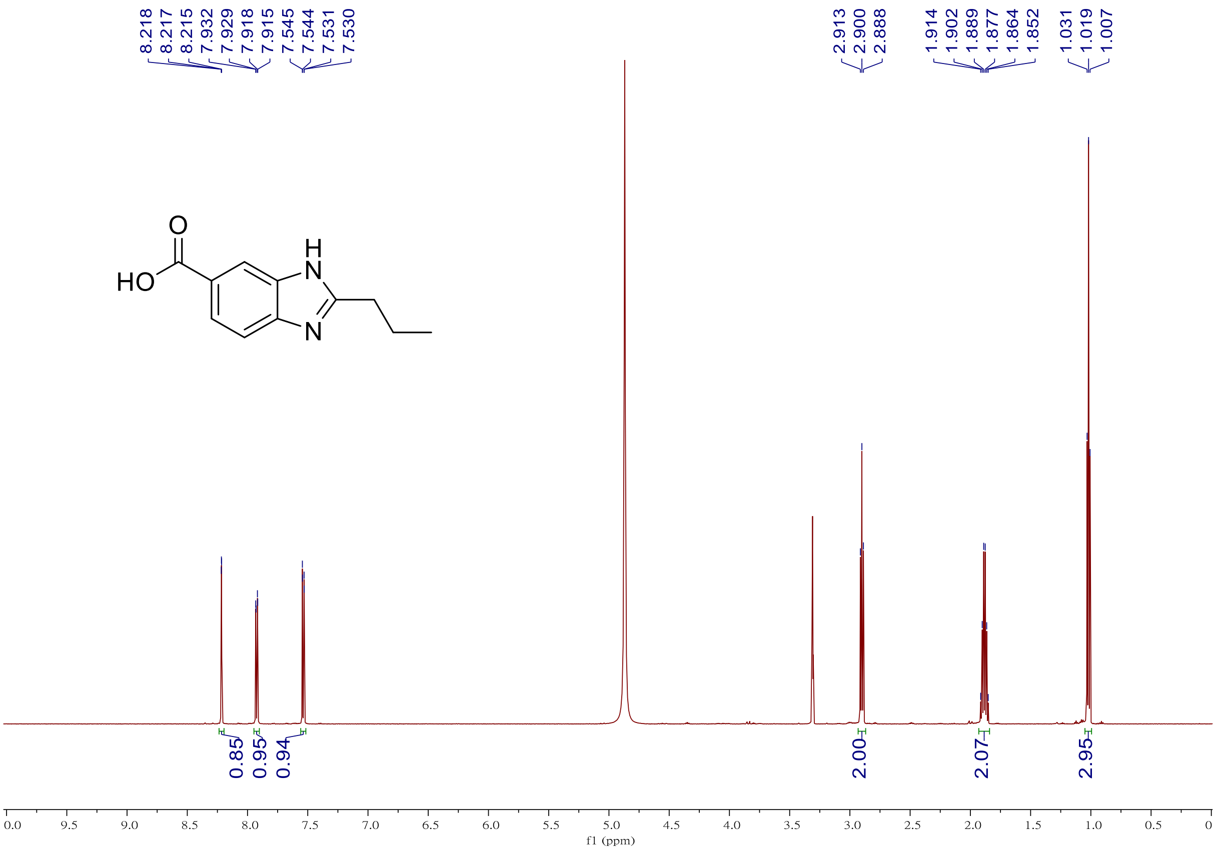


**Figure S9.** $^{1}$H NMR spectrum of compound **1d** (600 MHz, $CD_3OD$).

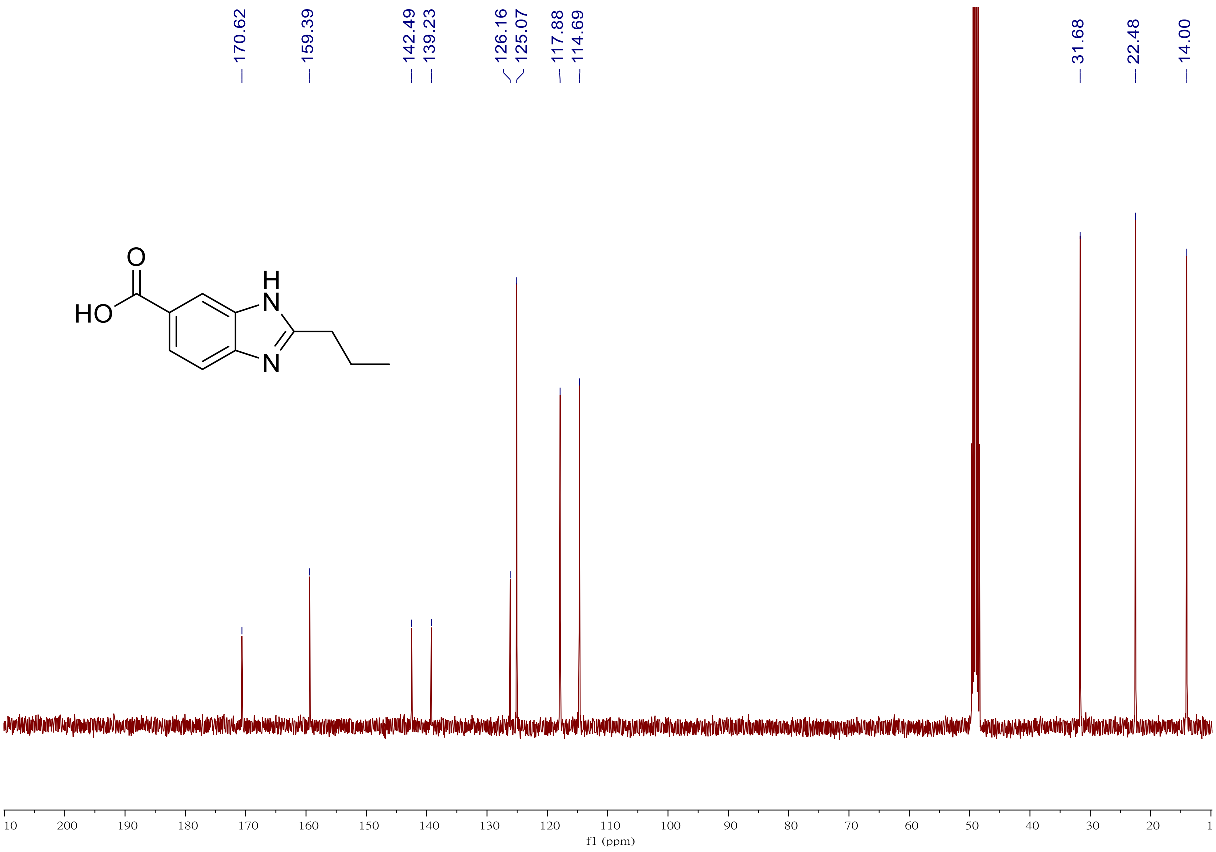


**Figure S10.** $^{13}C\{^1H\}$ NMR spectrum of compound **1d** (101 MHz, $CD_3OD$).

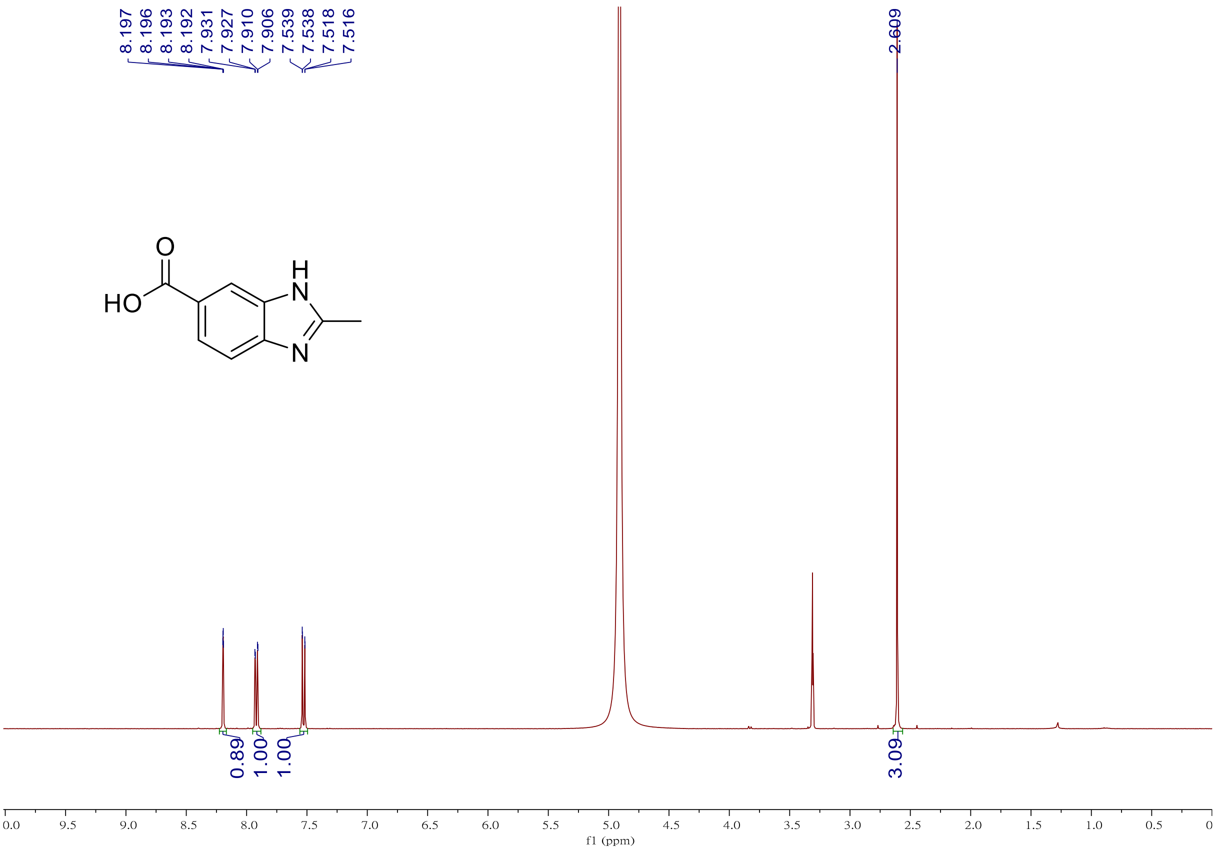


**Figure S11.** $^{1}$H NMR spectrum of compound **1e** (400 MHz, $CD_3OD$).

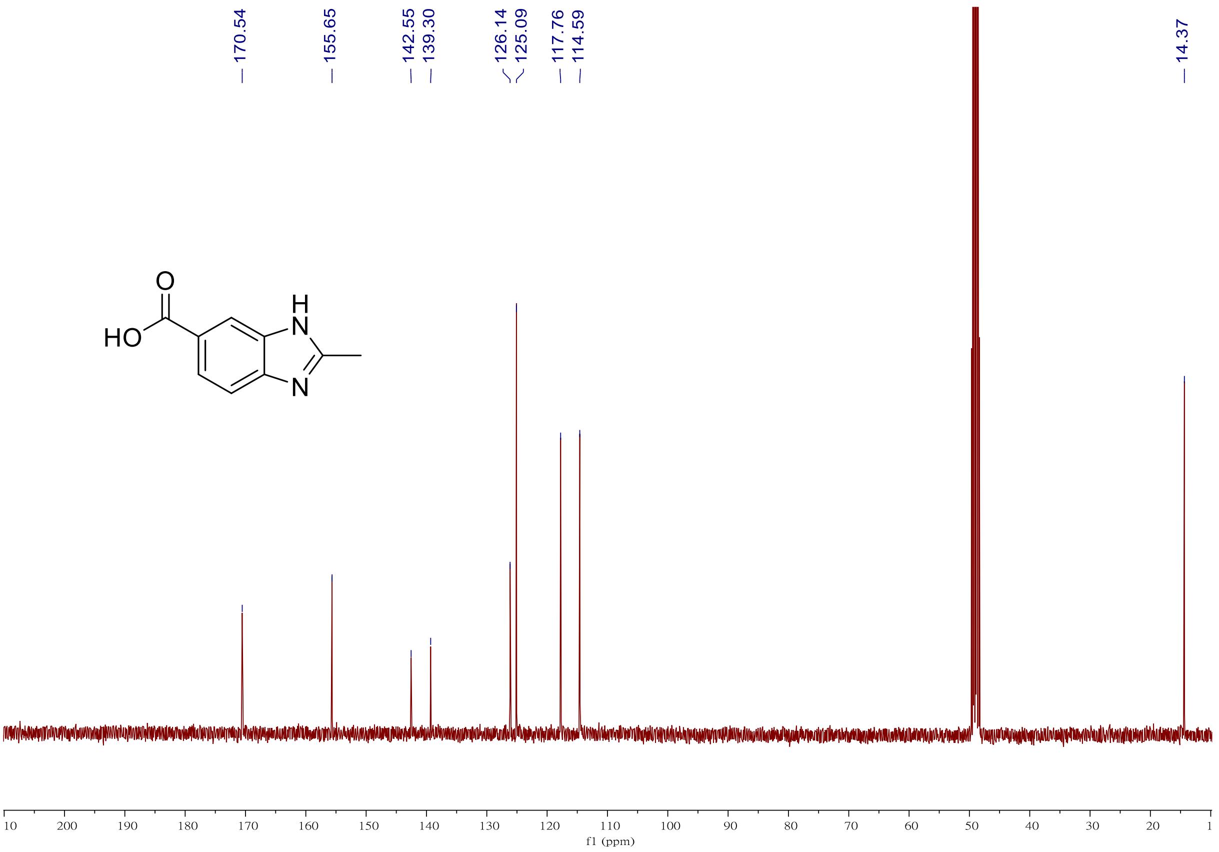


**Figure S12.** $^{13}C\{^{1}H\}$ NMR spectrum of compound **1e** (101 MHz, $CD_3OD$).

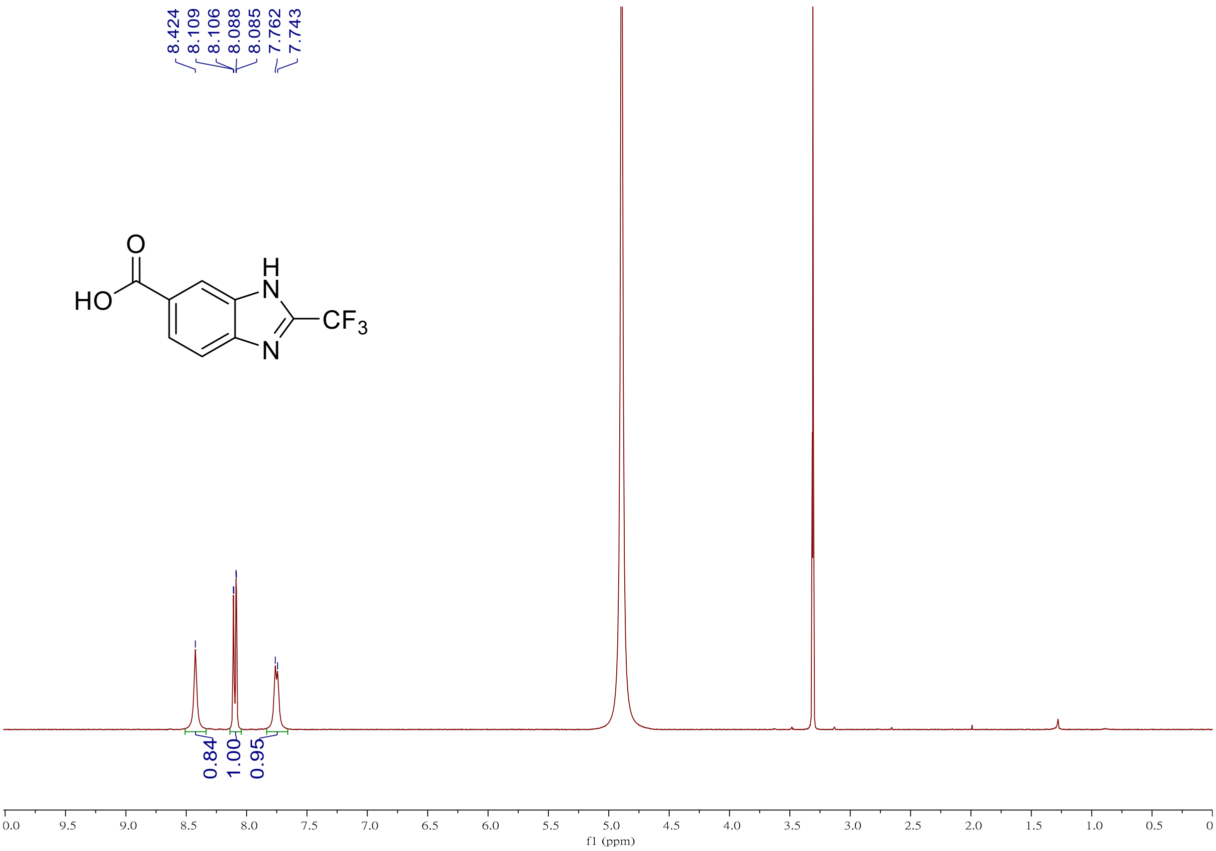


**Figure S13.** $^{1}H$ NMR spectrum of compound **1f** (400 MHz, $CD_3OD$).

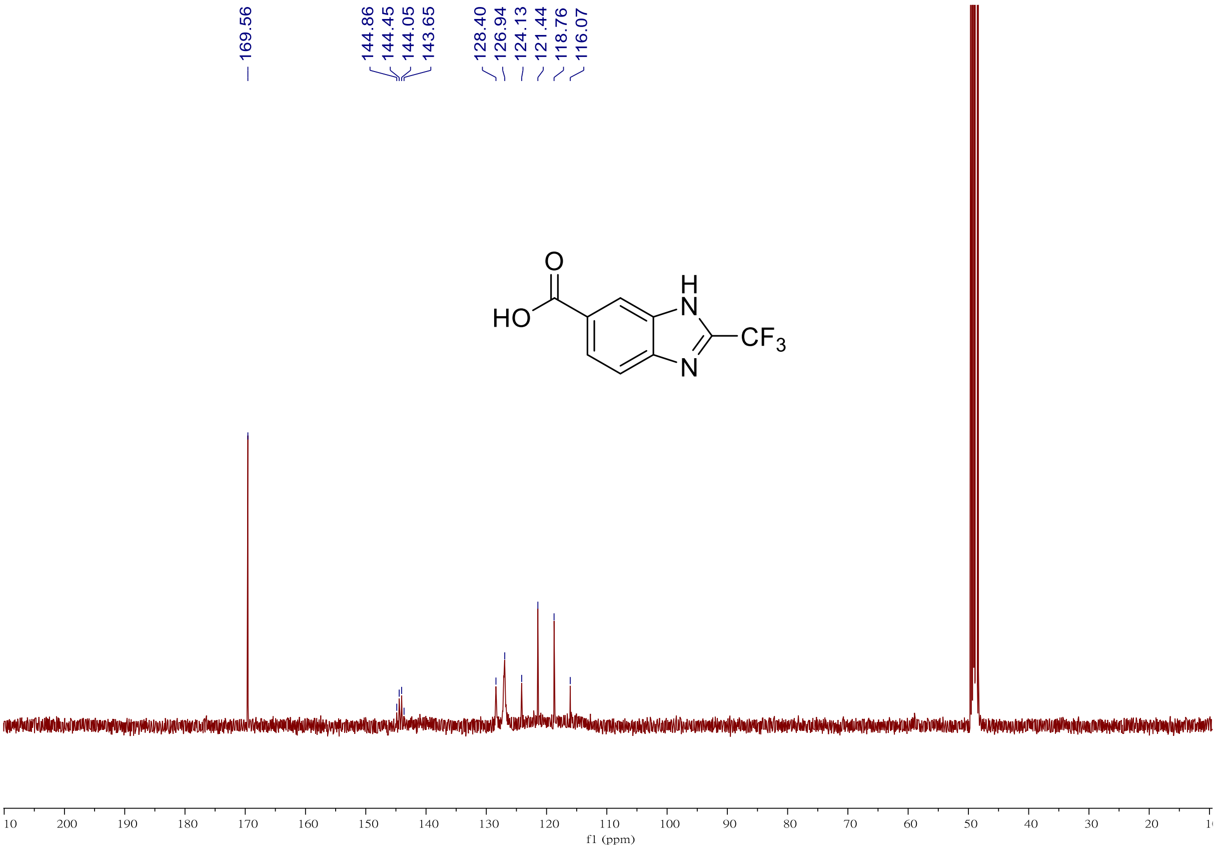


**Figure S14.** $^{13}C\{^{1}H\}$ NMR spectrum of compound **1f** (101 MHz, $CD_3OD$).

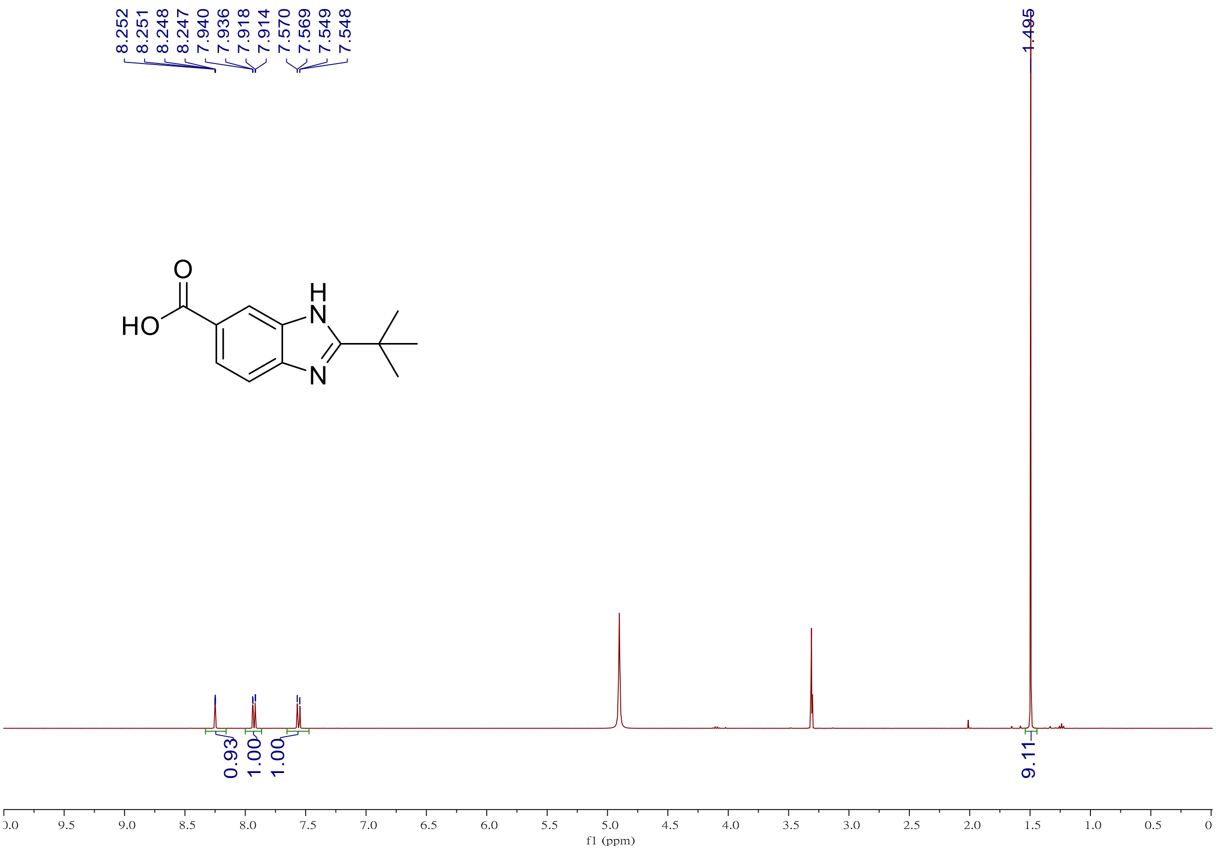


**Figure S15.** $^{1}$H NMR spectrum of compound **1g** (400 MHz, $CD_3OD$).

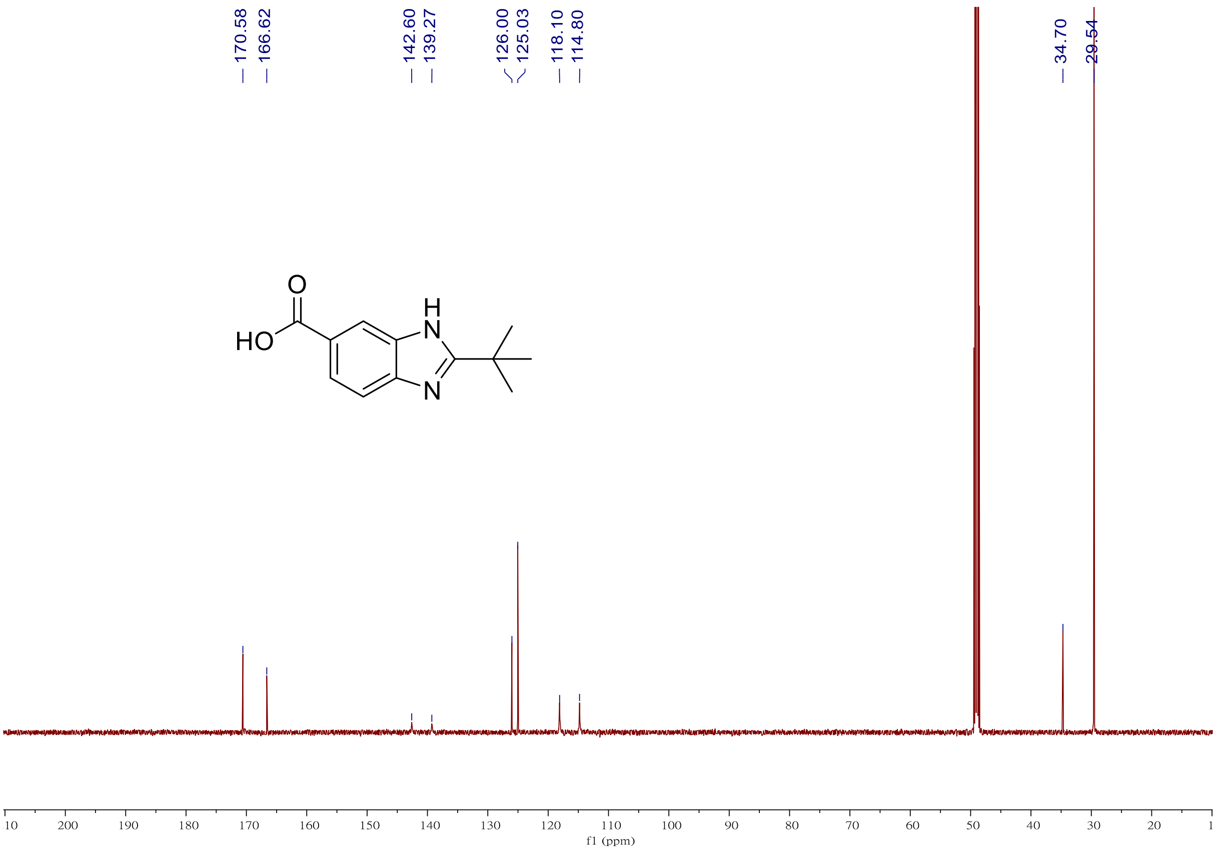


**Figure S16.** $^{13}C\{^{1}H\}$ NMR spectrum of compound **1g** (151 MHz, $CD_3OD$).

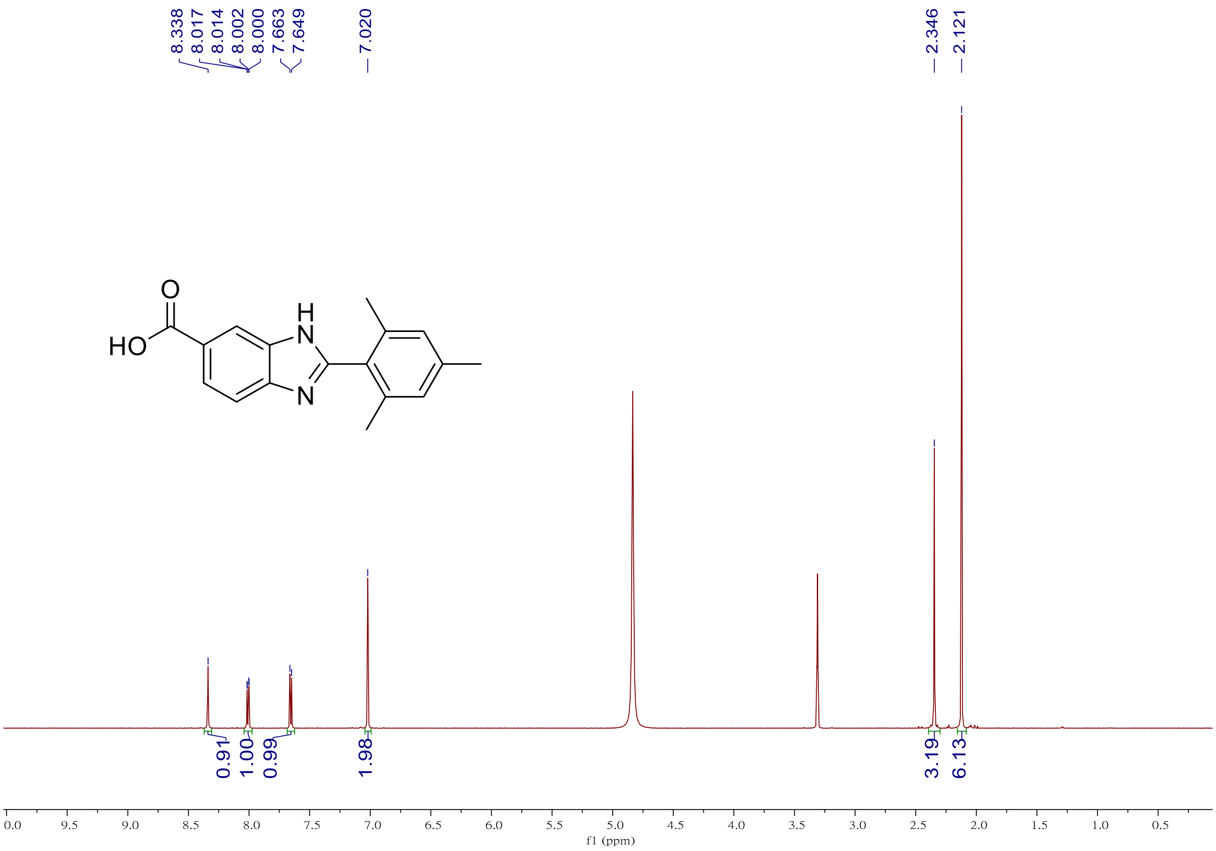


**Figure S17.** $^{1}$H NMR spectrum of compound **1h** (600 MHz, $CD_3OD$).

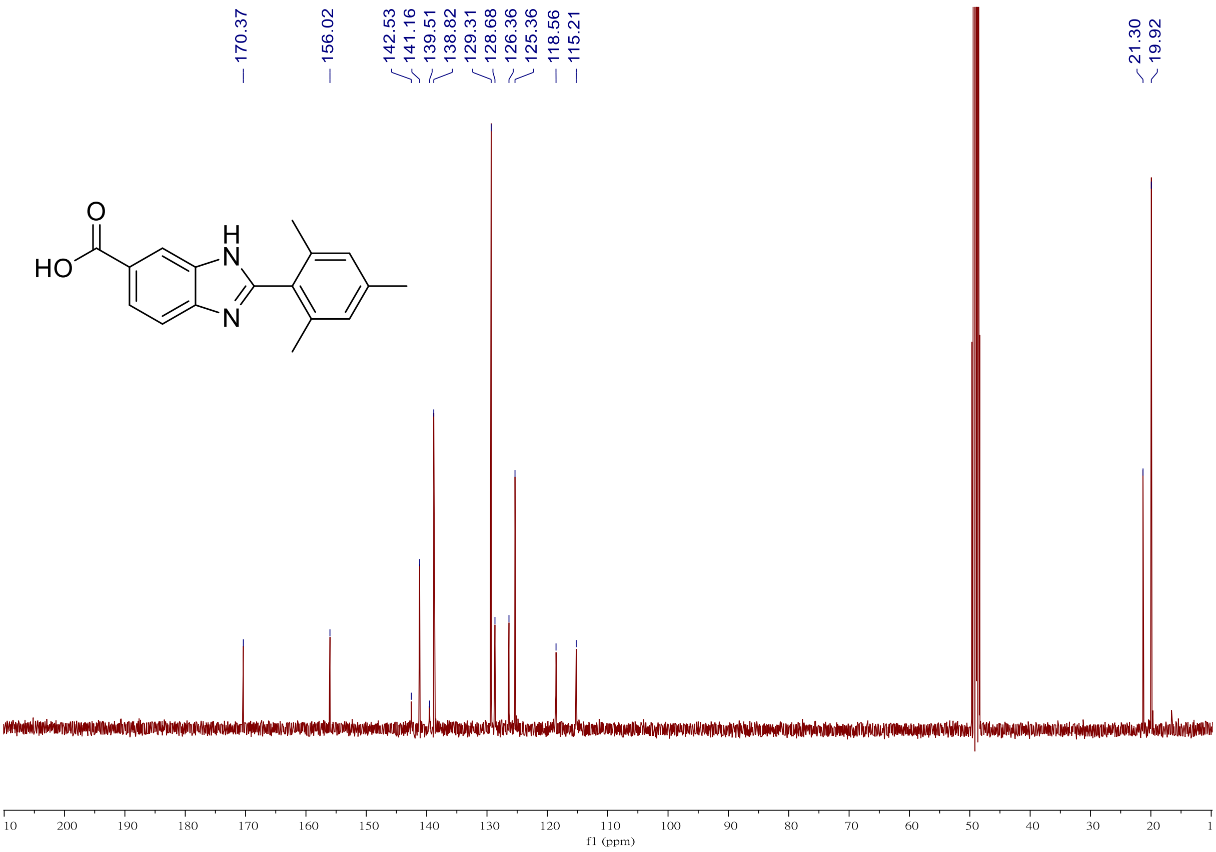


**Figure S18.** $^{13}C\{^1H\}$ NMR spectrum of compound **1h** (101 MHz, $CD_3OD$).

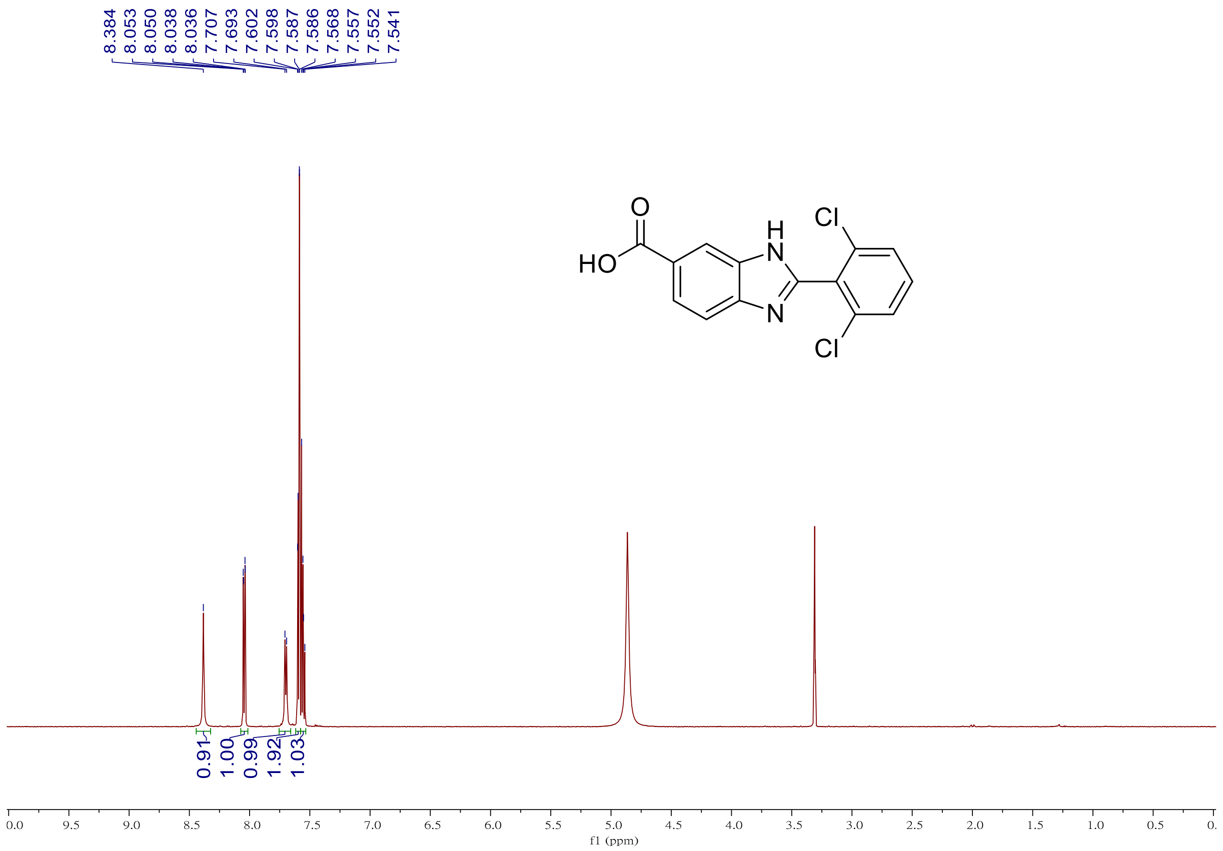


**Figure S19.** $^{1}$H NMR spectrum of compound **1i** (600 MHz, $CD_3OD$).

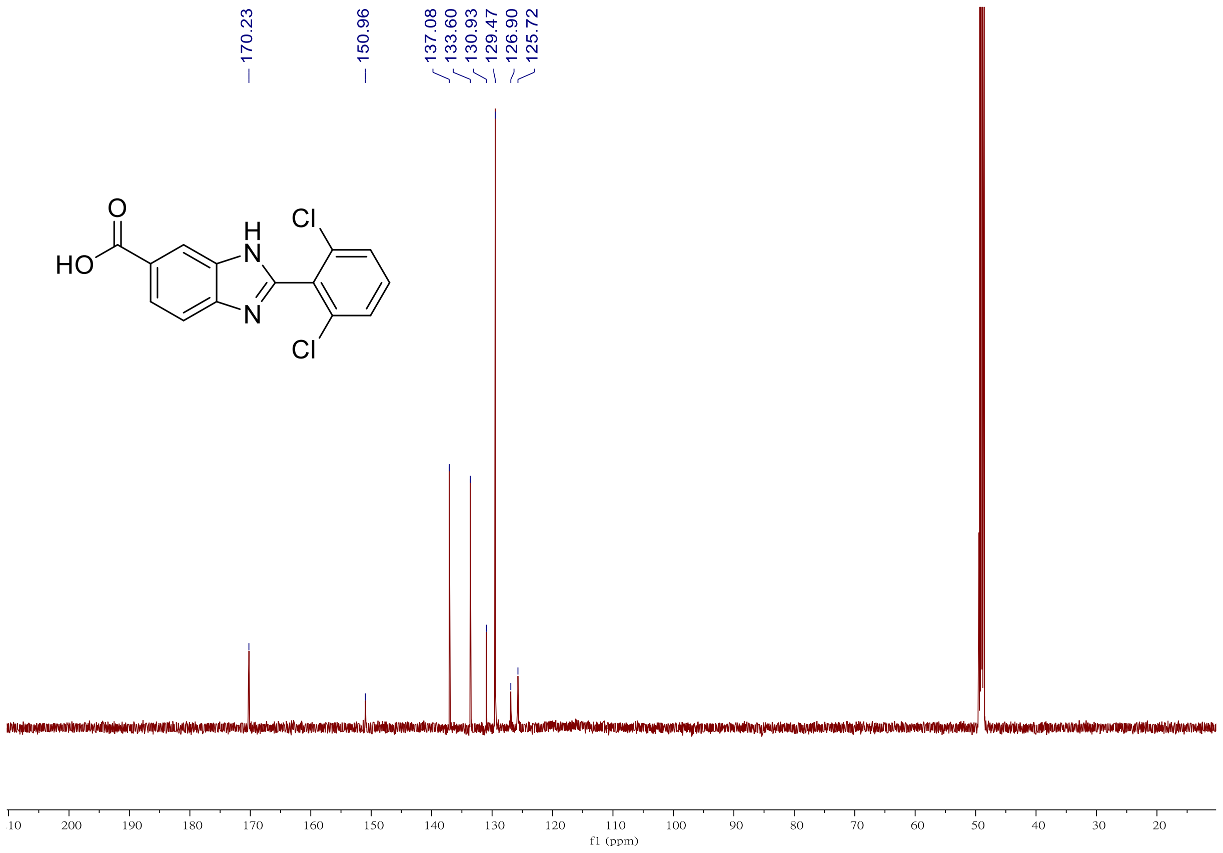


**Figure S20.** $^{13}C\{^{1}H\}$ NMR spectrum of compound **1i** (151 MHz, $CD_3OD$).

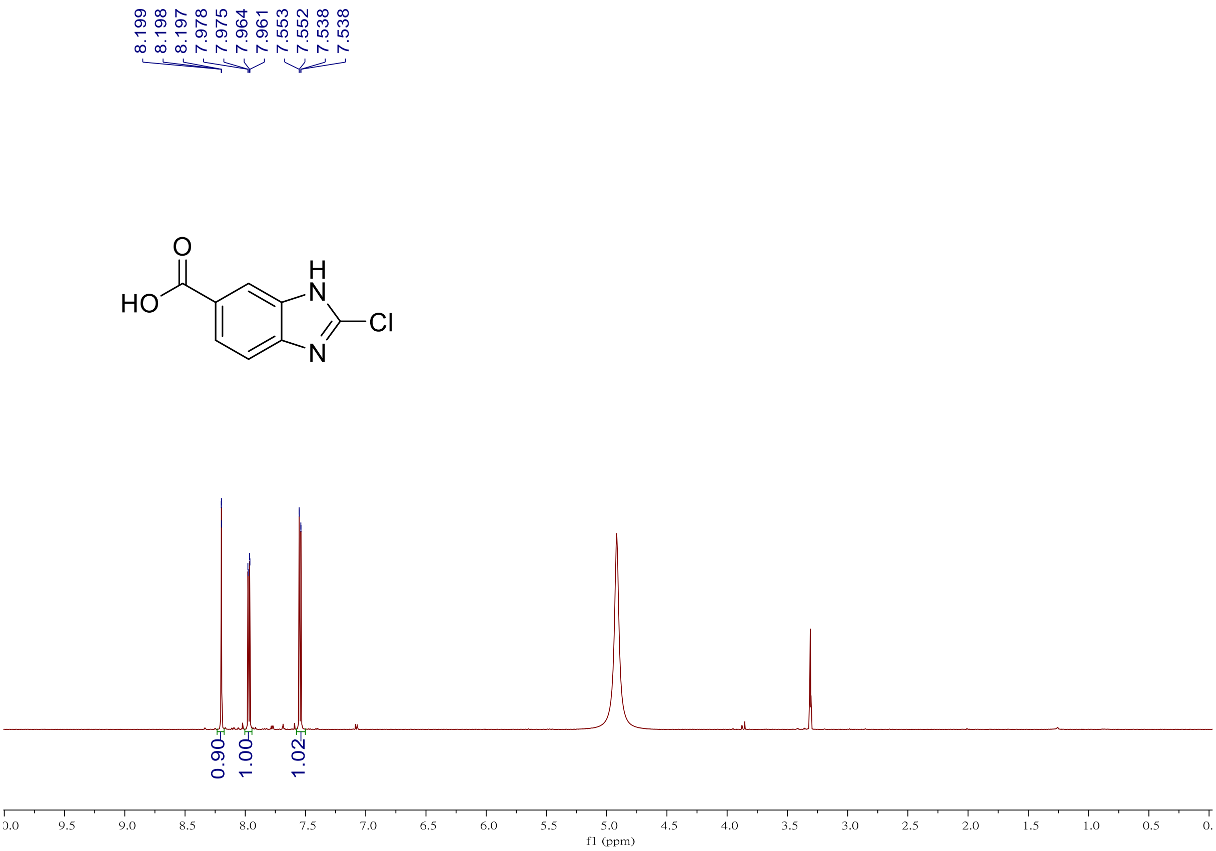


**Figure S21.** ¹H NMR spectrum of compound **1j** (600 MHz, $CD_3OD$).

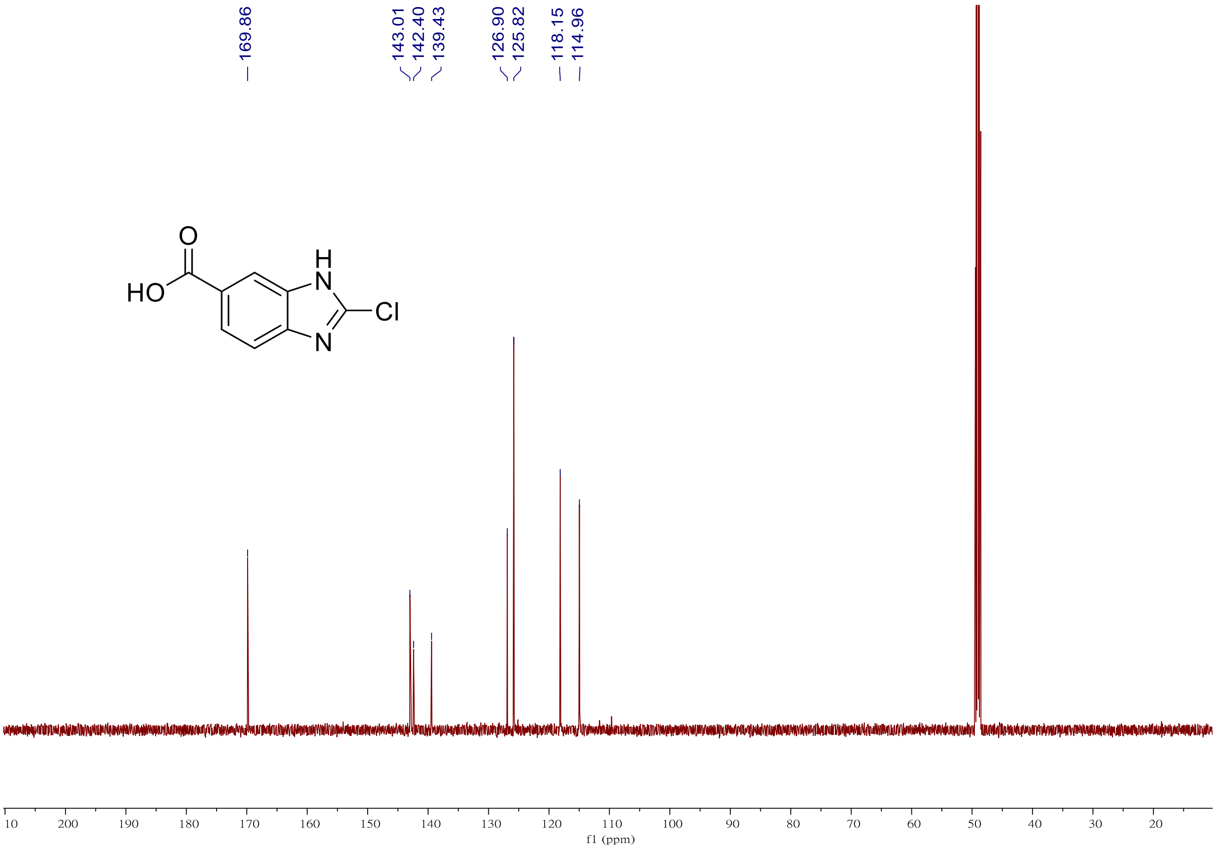


**Figure S22.** $^{13}C\{^{1}H\}$ NMR spectrum of compound **1j** (151 MHz, $CD_3OD$).

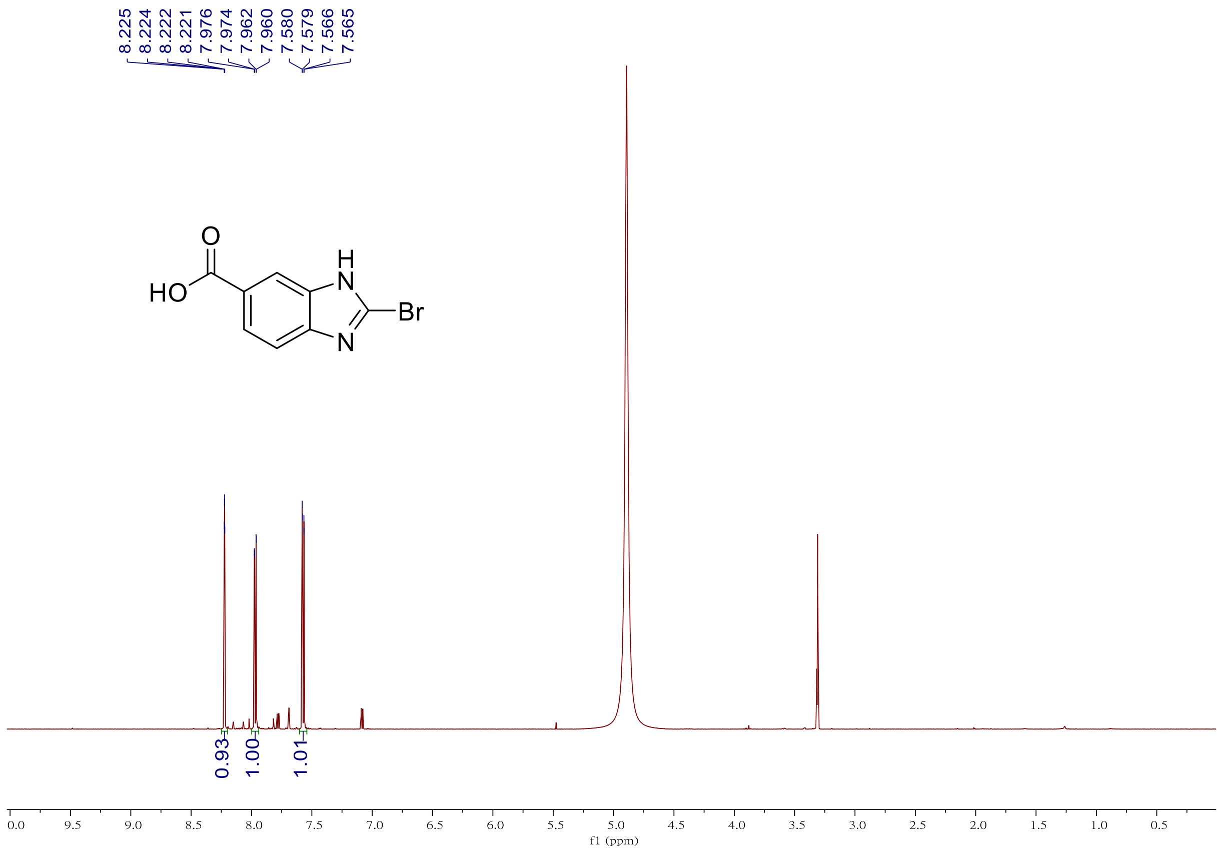


**Figure S23.** $^{1}$H NMR spectrum of compound **1k** (600 MHz, $CD_3OD$).

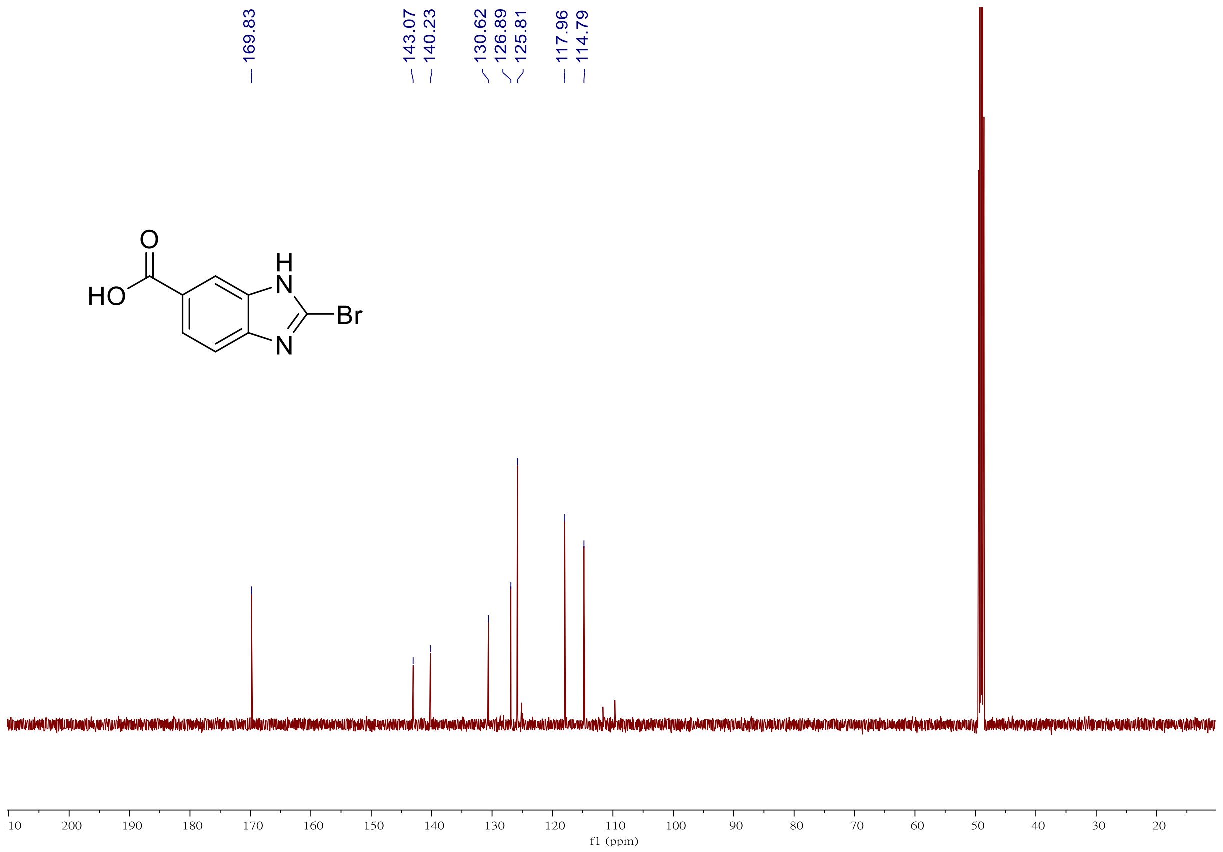


**Figure S24.** $^{13}C\{^{1}H\}$ NMR spectrum of compound **1k** (151 MHz, $CD_3OD$).

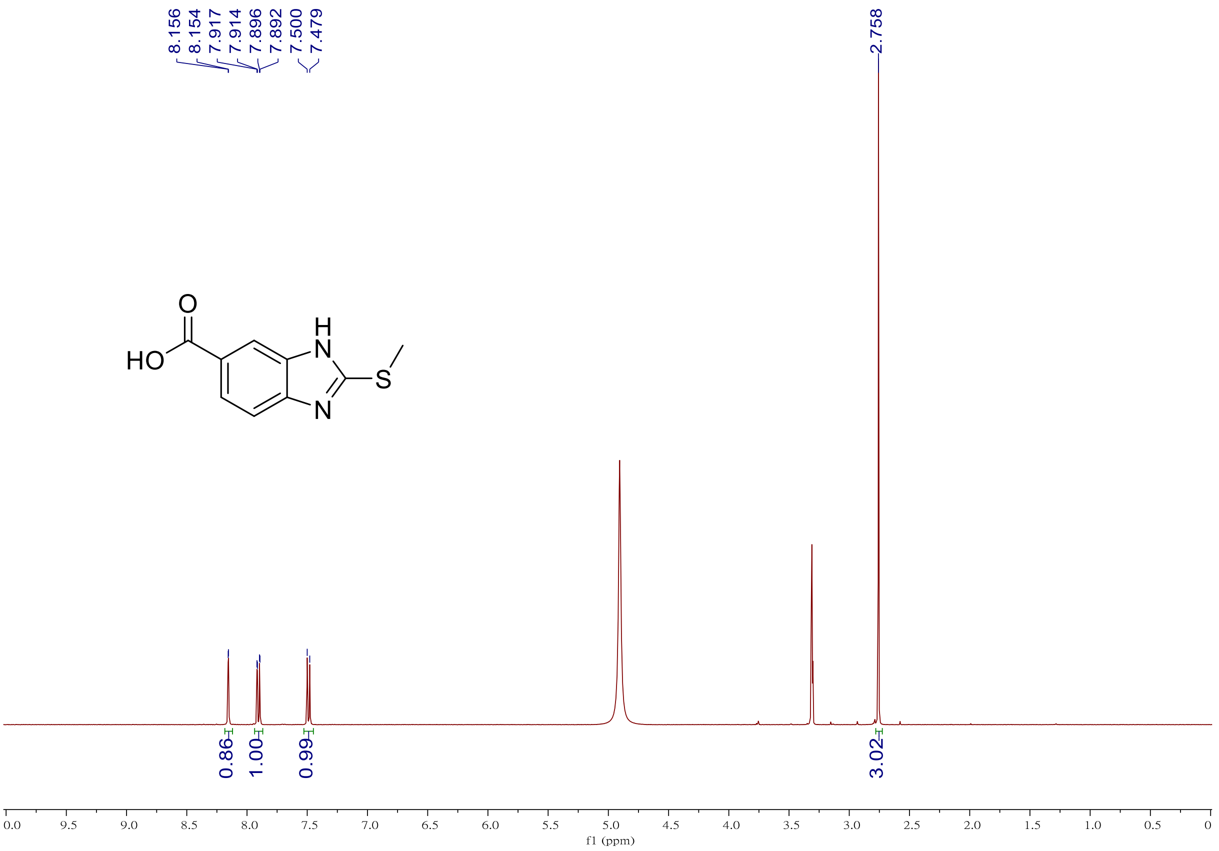


**Figure S25.** $^{1}$H NMR spectrum of compound **1l** (400 MHz, $CD_3OD$).

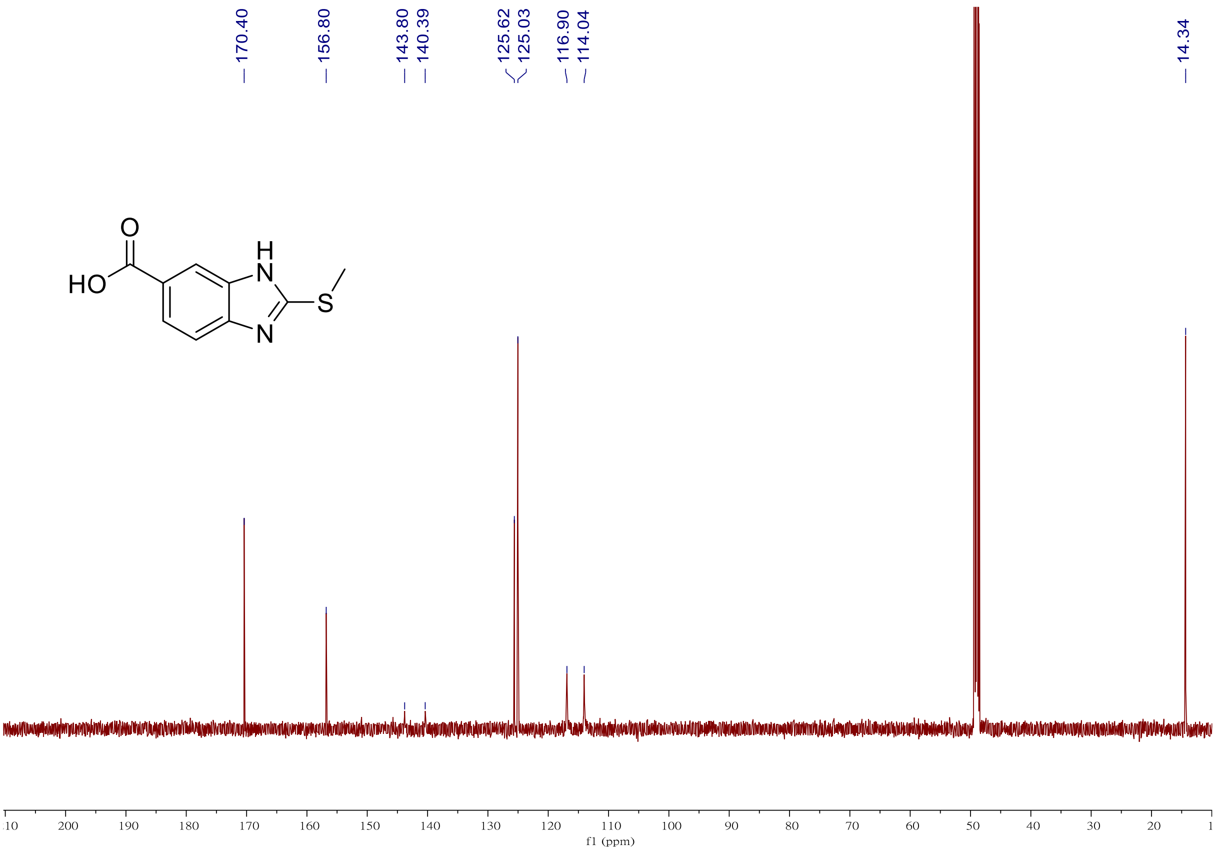


**Figure S26.** $^{13}C\{^{1}H\}$ NMR spectrum of compound **1l** (151 MHz, $CD_3OD$).

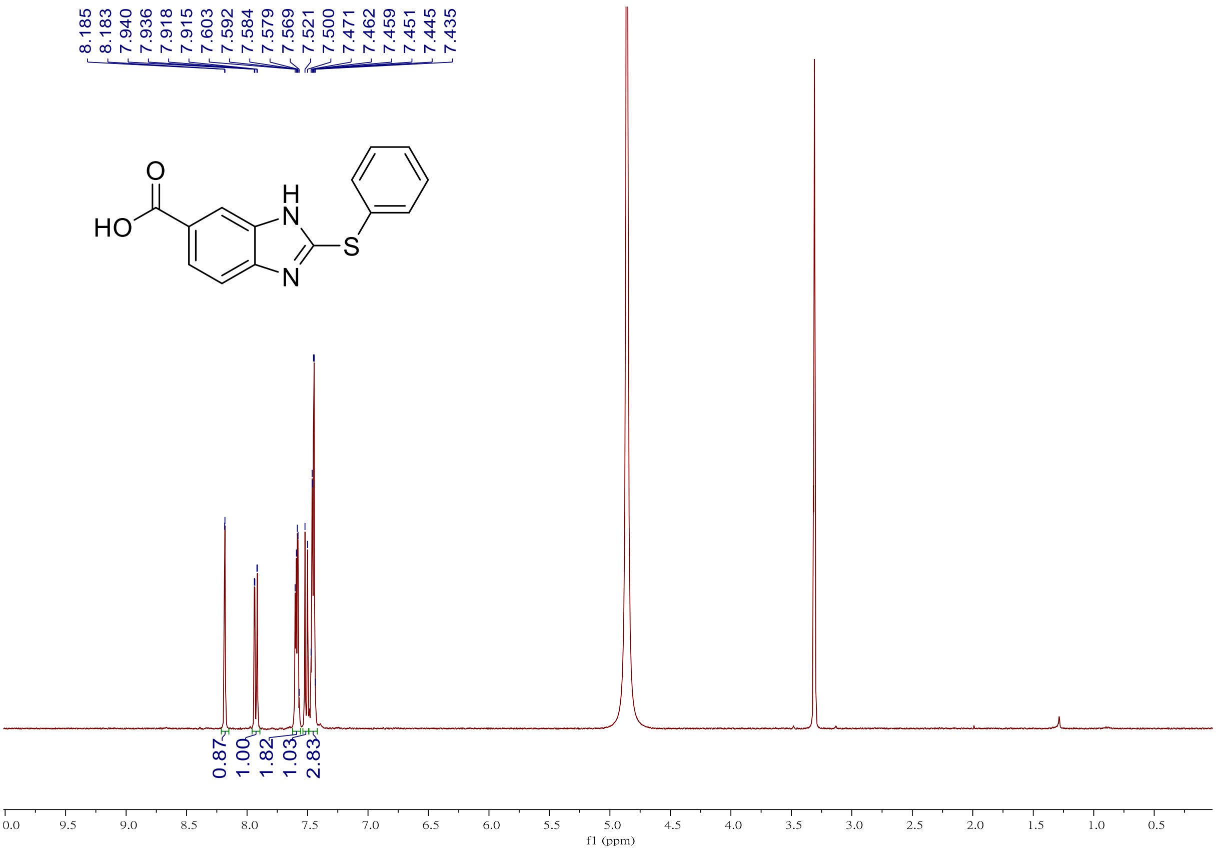


**Figure S27.** $^{1}H$ NMR spectrum of compound **1m** (400 MHz, $CD_3OD$).

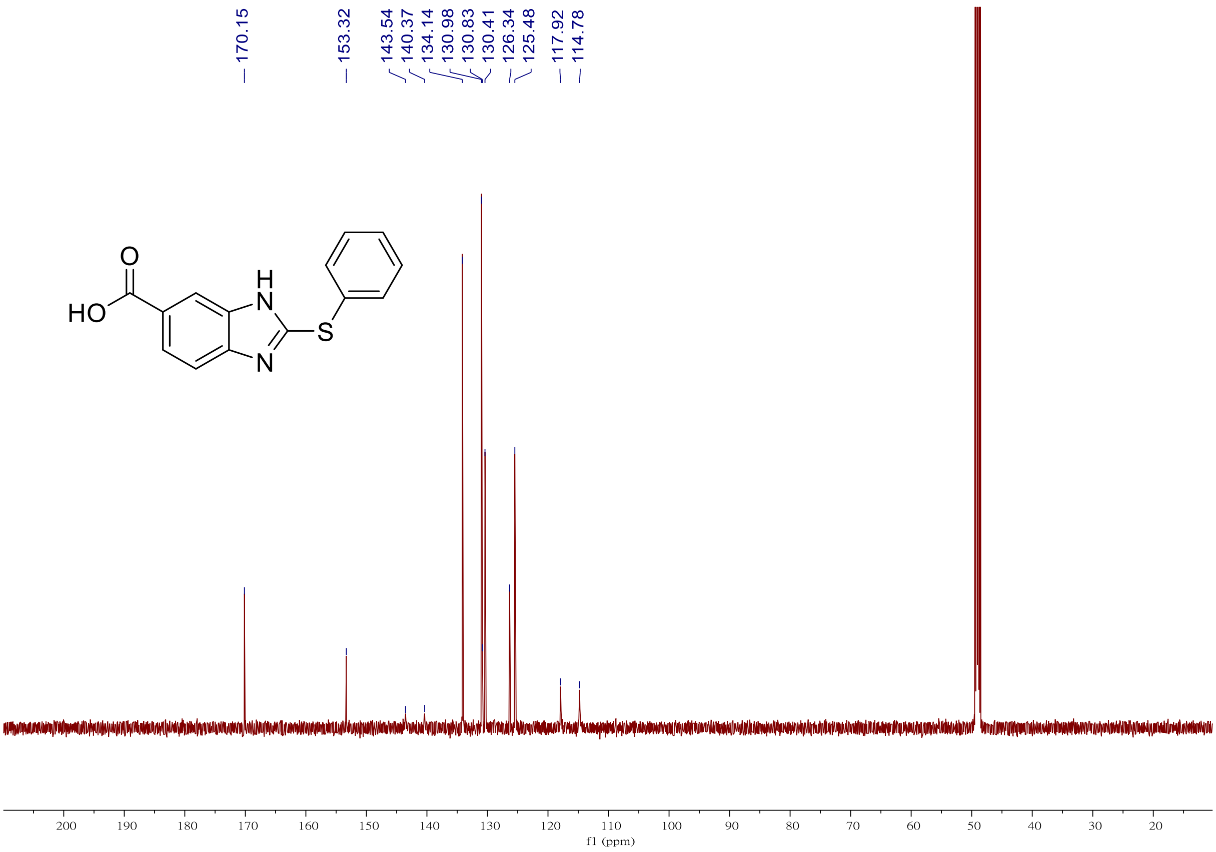


**Figure S28.** $^{13}C\{^{1}H\}$ NMR spectrum of compound **1m** (151 MHz, $CD_3OD$).

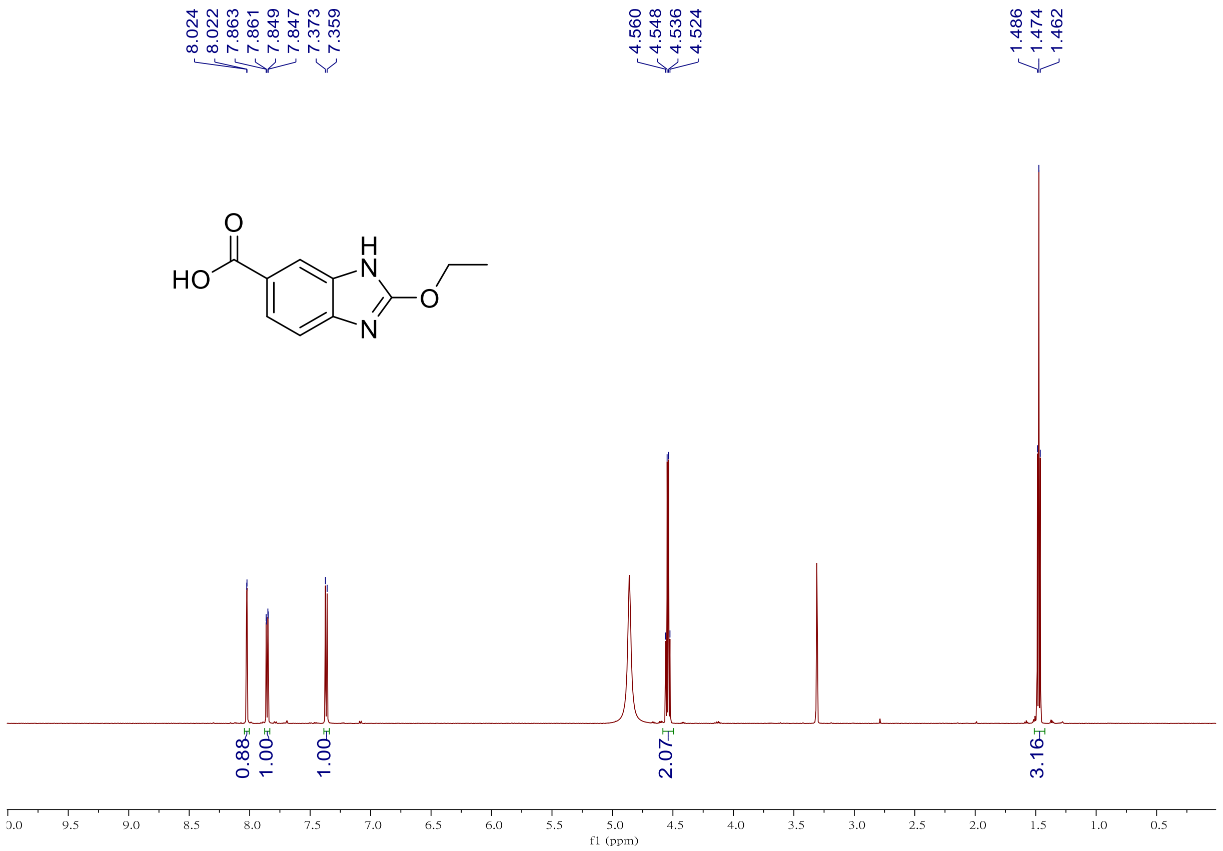


**Figure S29.** ¹H NMR spectrum of compound **1n** (600 MHz, $CD_3OD$).

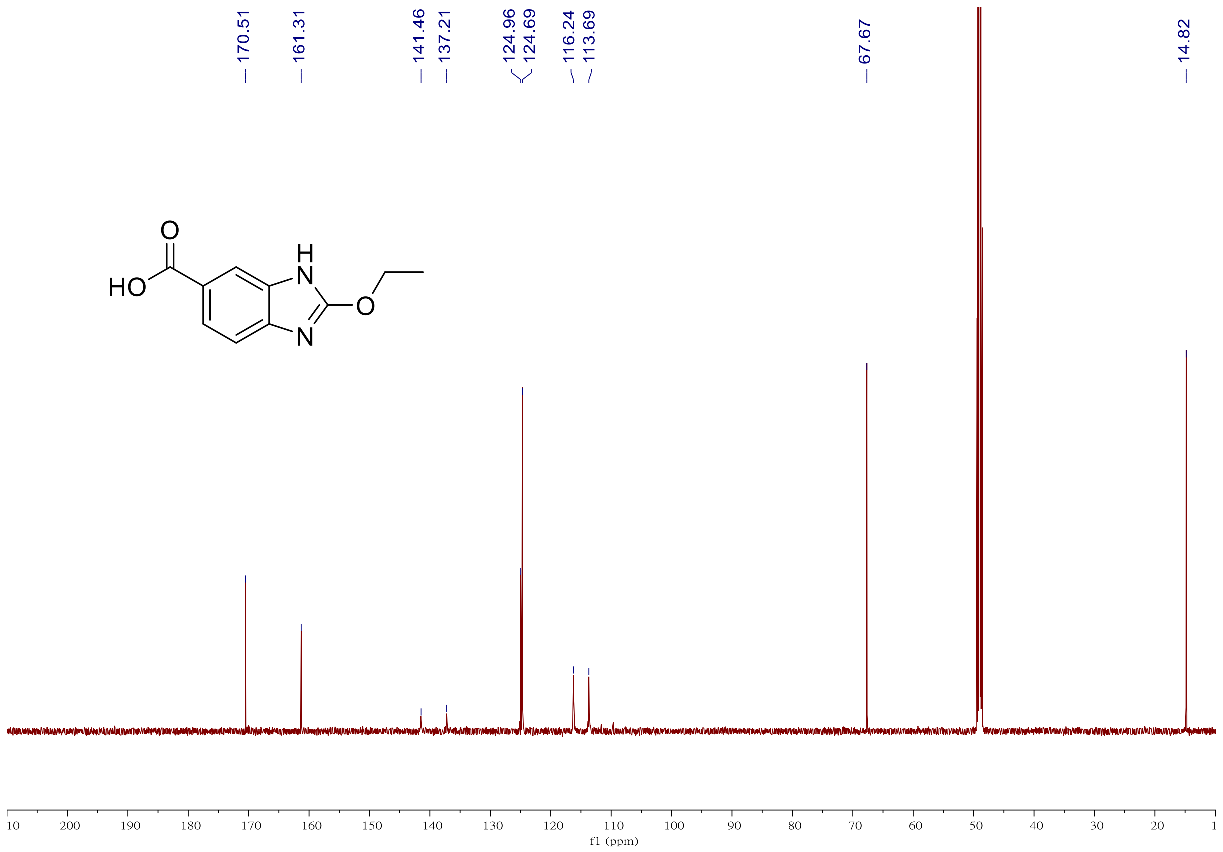


**Figure S30.** $^{13}C\{^1H\}$ NMR spectrum of compound **1n** (151 MHz, $CD_3OD$).

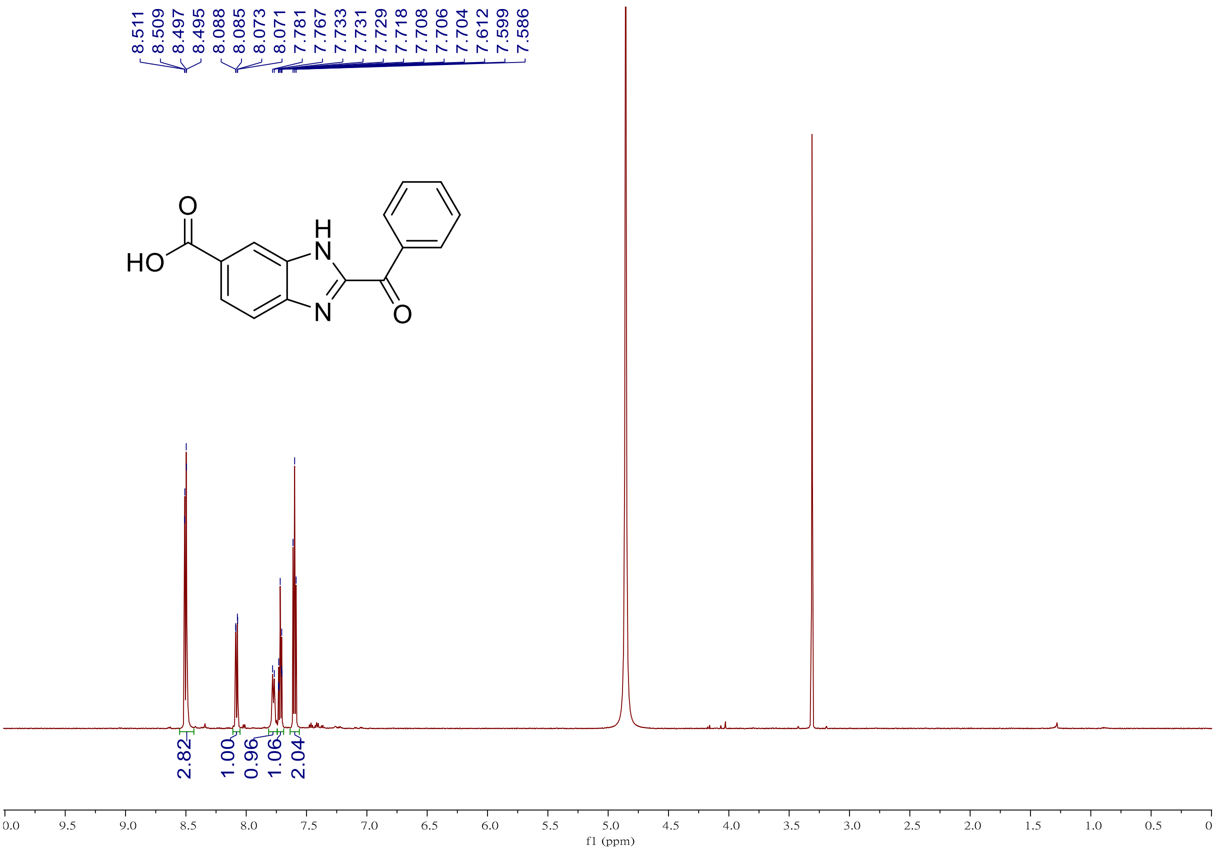


**Figure S31.** $^{1}$H NMR spectrum of compound **1o** (600 MHz, $CD_3OD$).

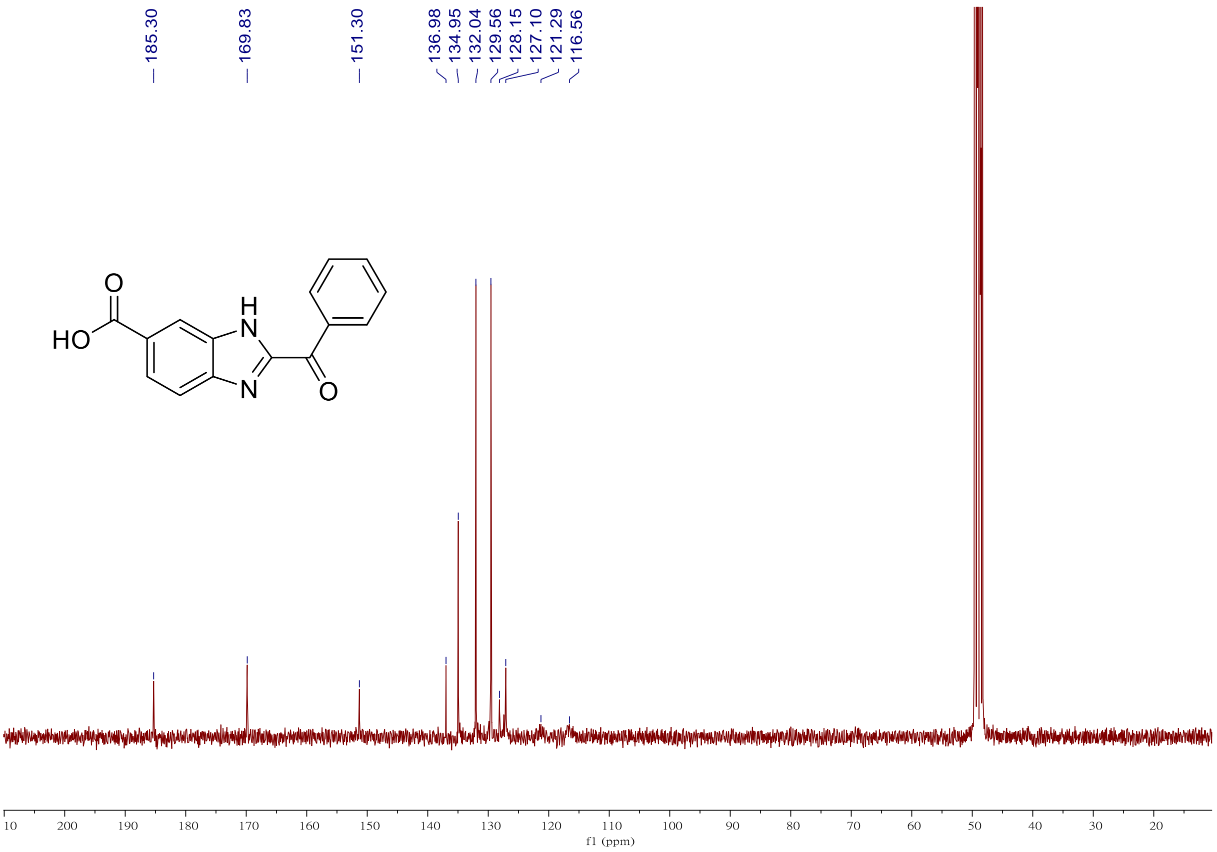


**Figure S32.** $^{13}C\{^{1}H\}$ NMR spectrum of compound **1o** (101 MHz, $CD_3OD$).

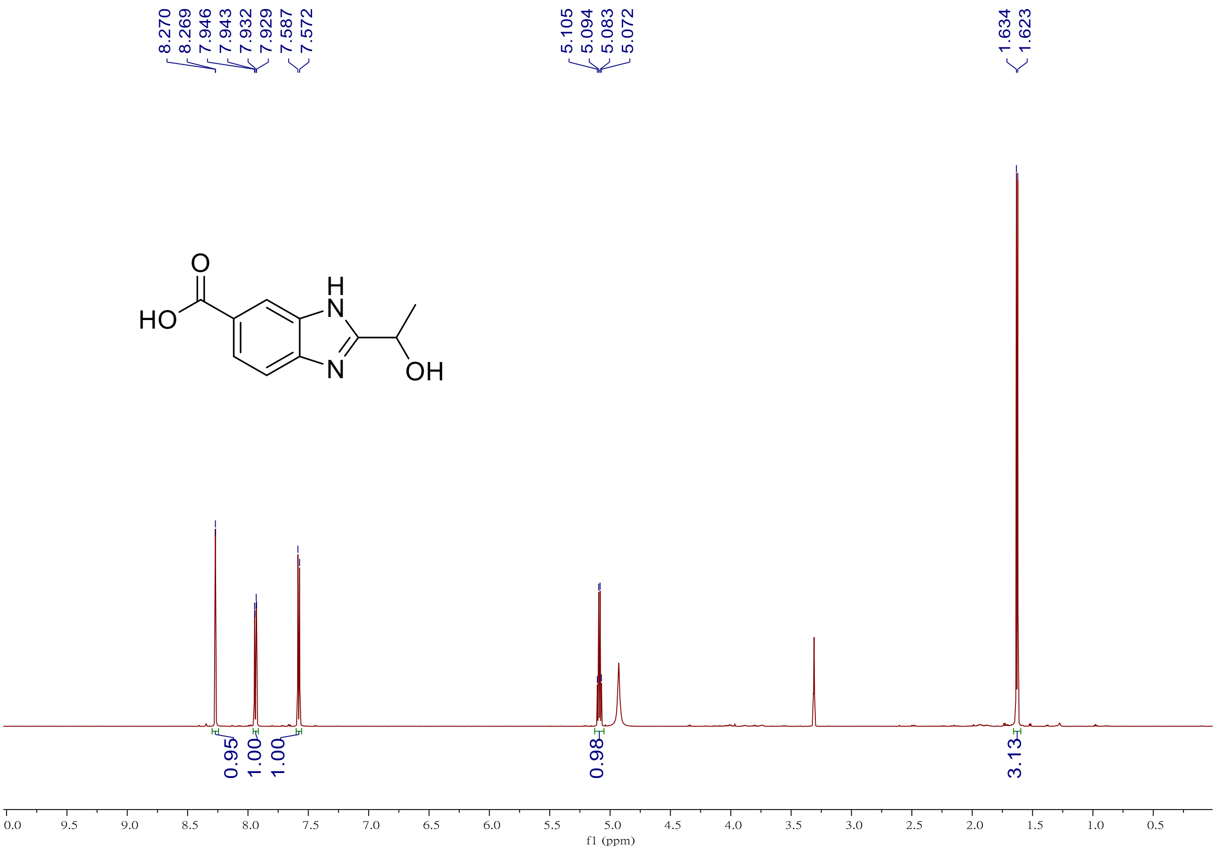


**Figure S33.** $^{1}H$ NMR spectrum of compound **1p** (600 MHz, $CD_3OD$).

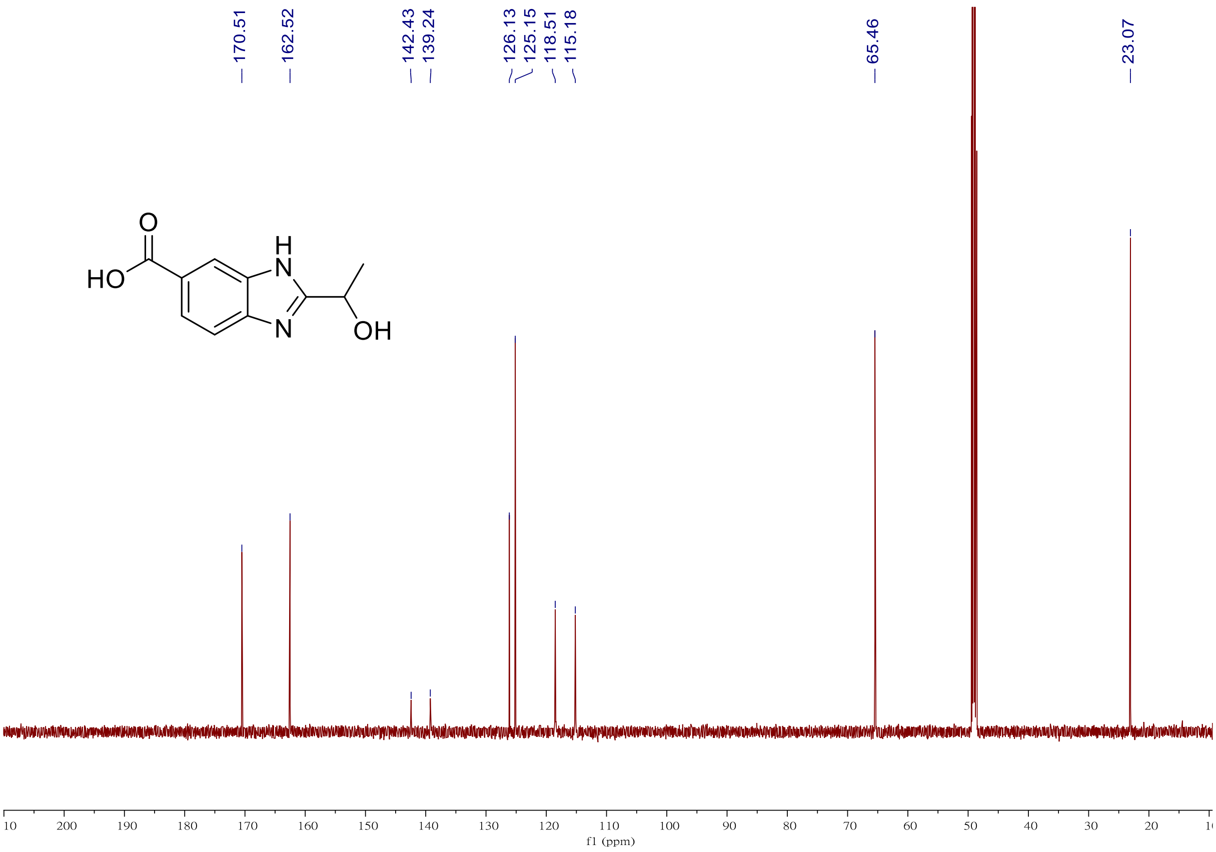


**Figure S34.** $^{13}C\{^{1}H\}$ NMR spectrum of compound **1p** (151 MHz, $CD_3OD$).

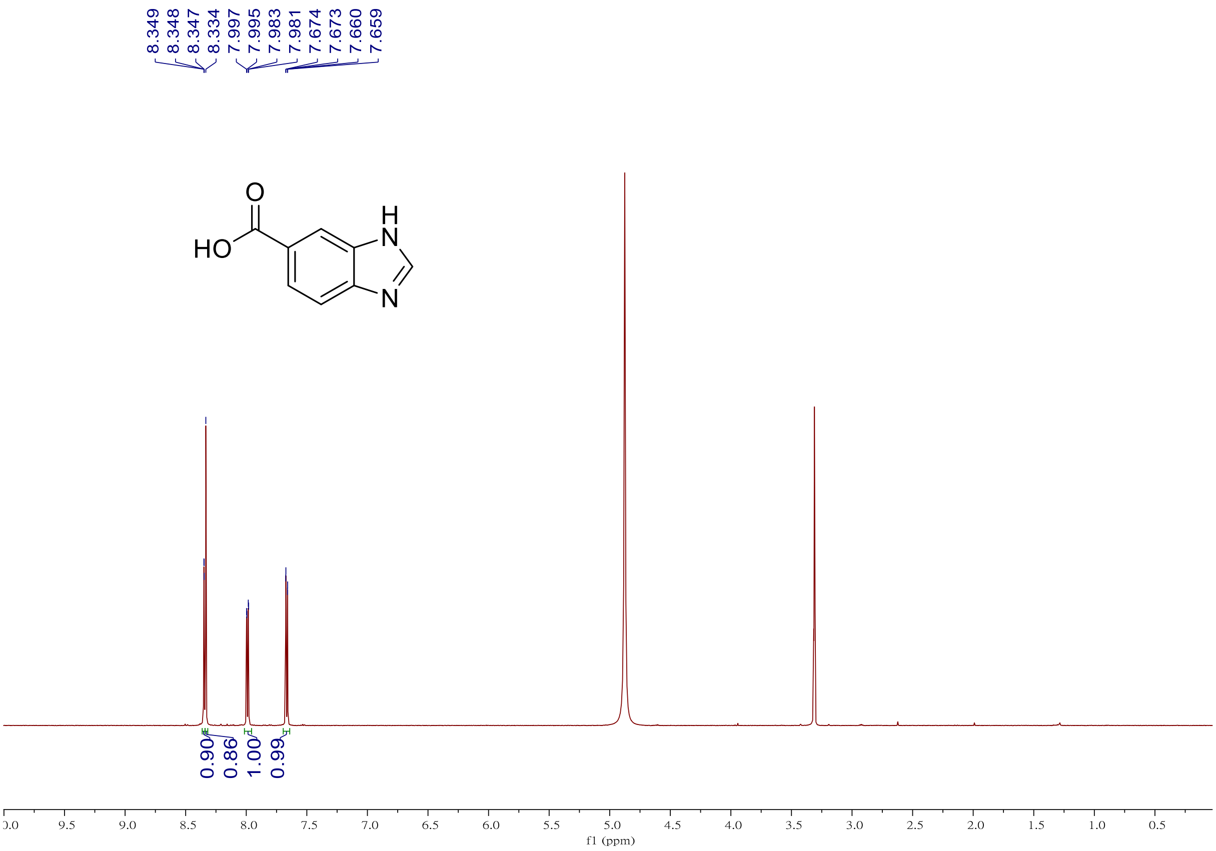


**Figure S35.** $^{1}$H NMR spectrum of compound **1q** (600 MHz, $CD_3OD$).

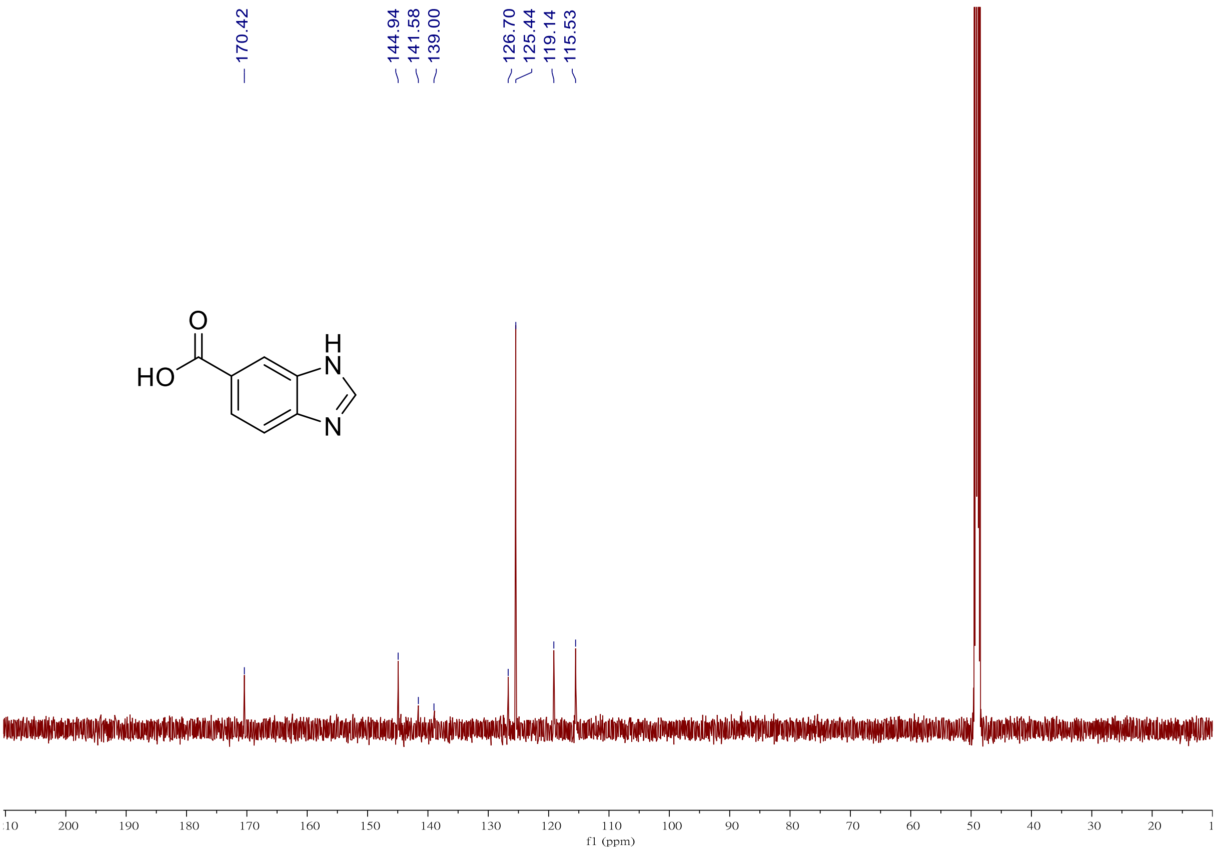


**Figure S36.** $^{13}$C{$^{1}$H} NMR spectrum of compound **1q** (151 MHz, $CD_3OD$).

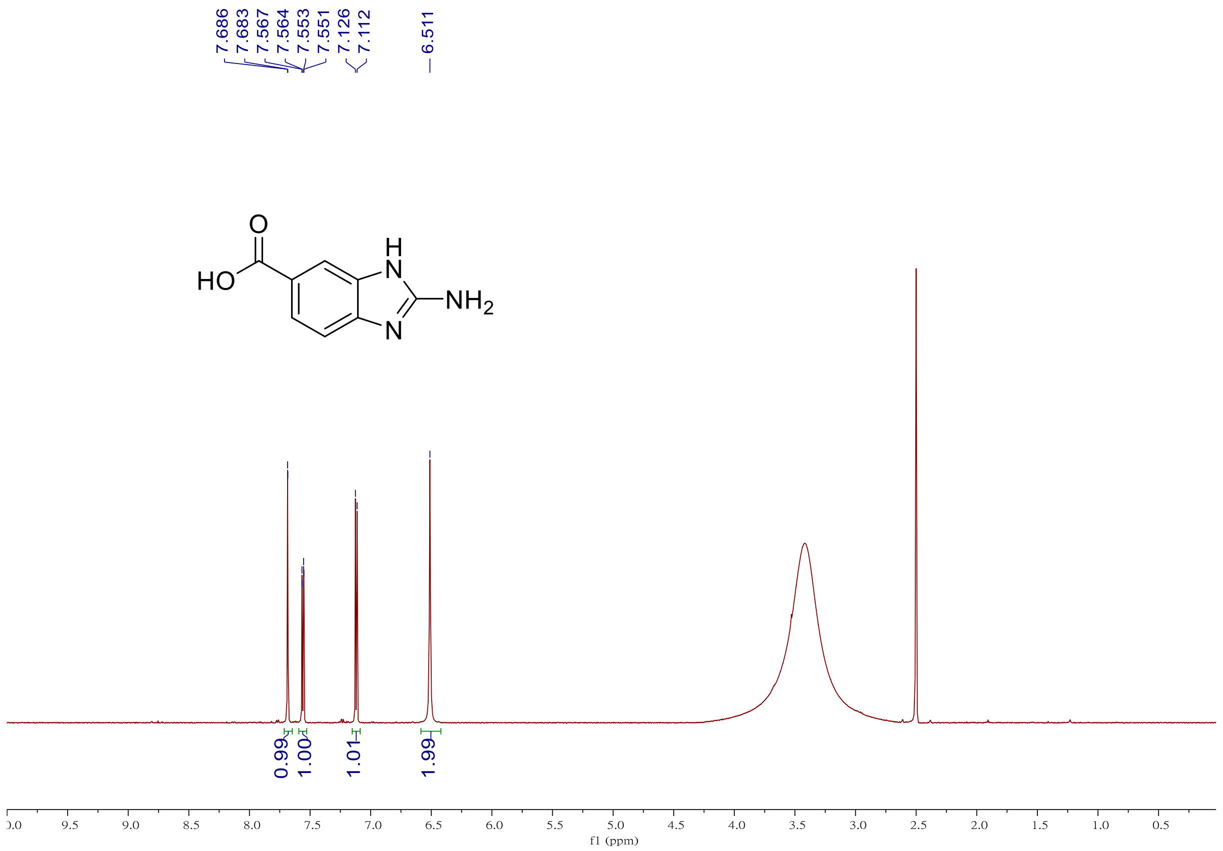


**Figure S37.** $^{1}H$ NMR spectrum of compound **1r** (600 MHz, $(CD_3)_2SO$).

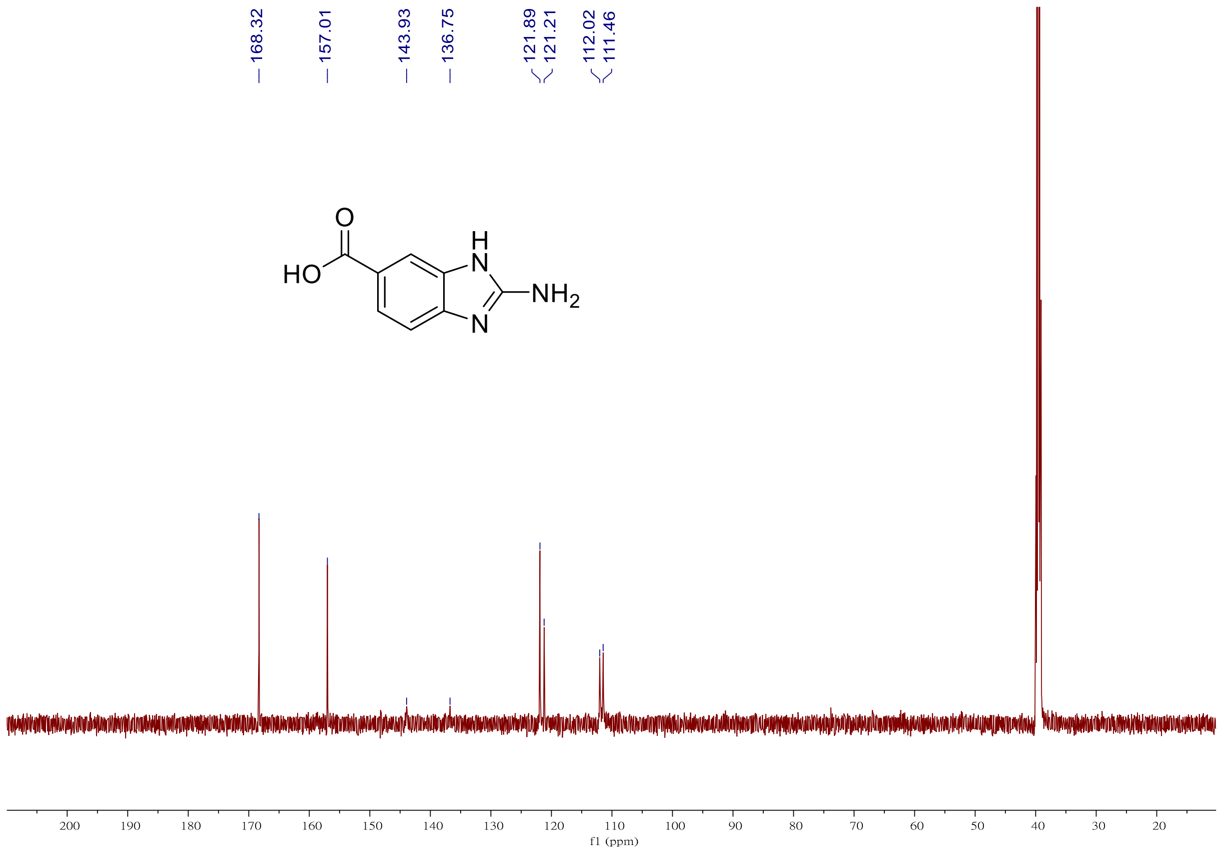


**Figure S38.** $^{13}C\{^{1}H\}$ NMR spectrum of compound **1r** (151 MHz, $(CD_3)_2SO$).

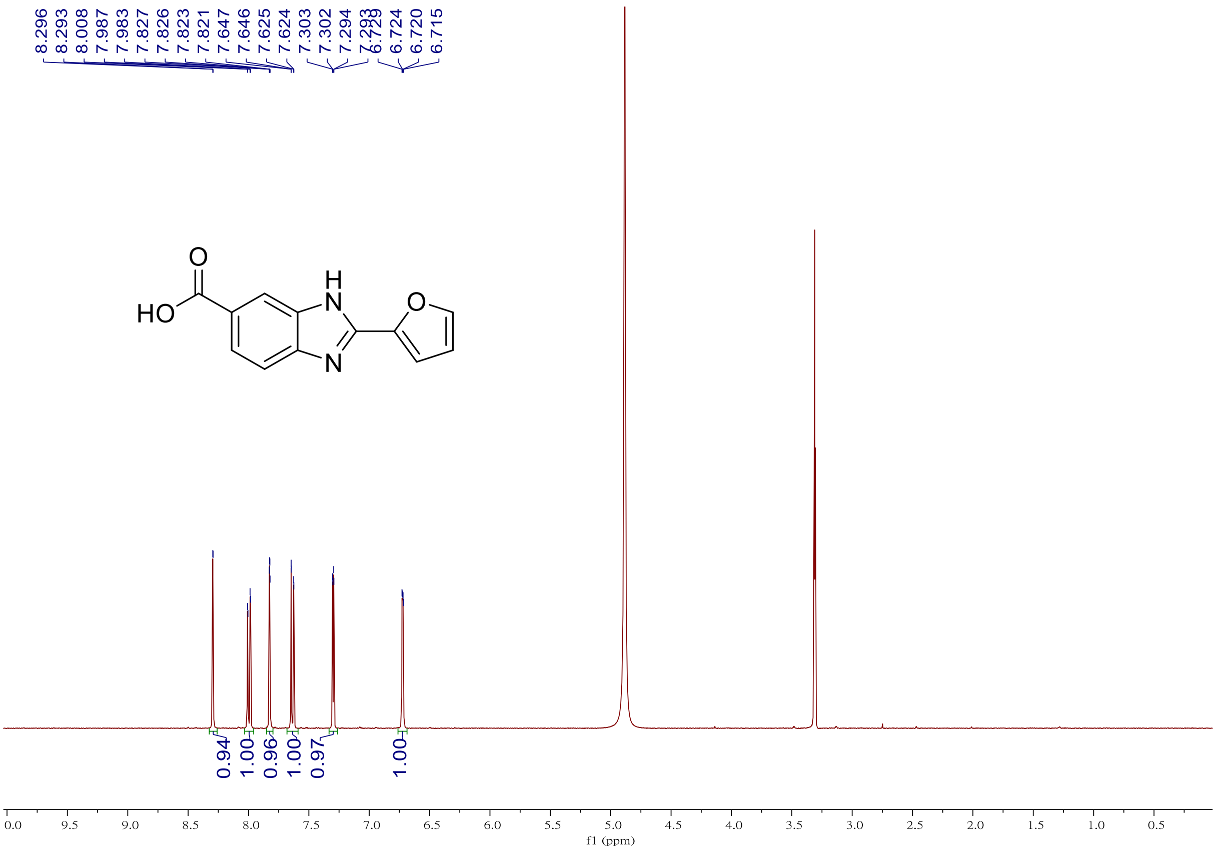


**Figure S39.** $^{1}H$ NMR spectrum of compound **1s** (400 MHz, $CD_3OD$).

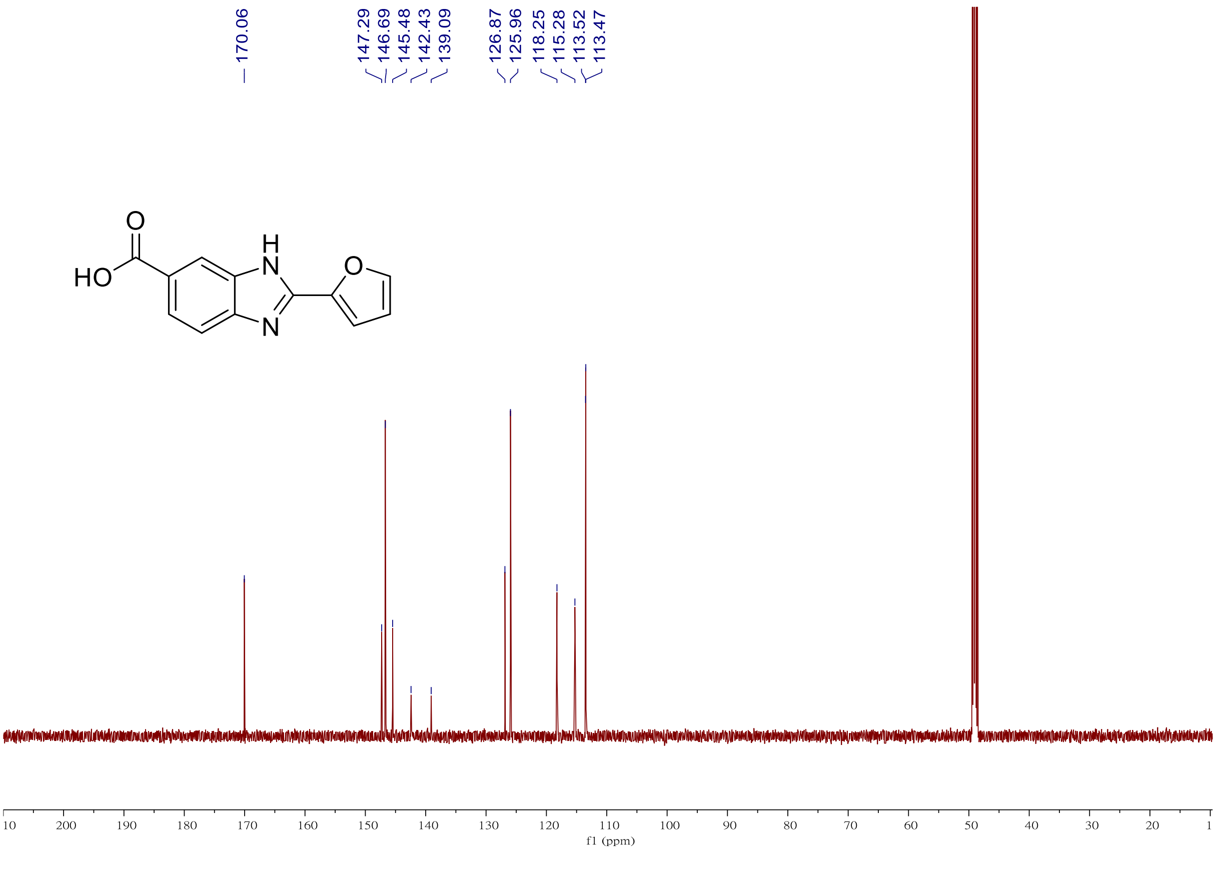


**Figure S40.** $^{13}C\{^{1}H\}$ NMR spectrum of compound **1s** (151 MHz, $CD_3OD$).

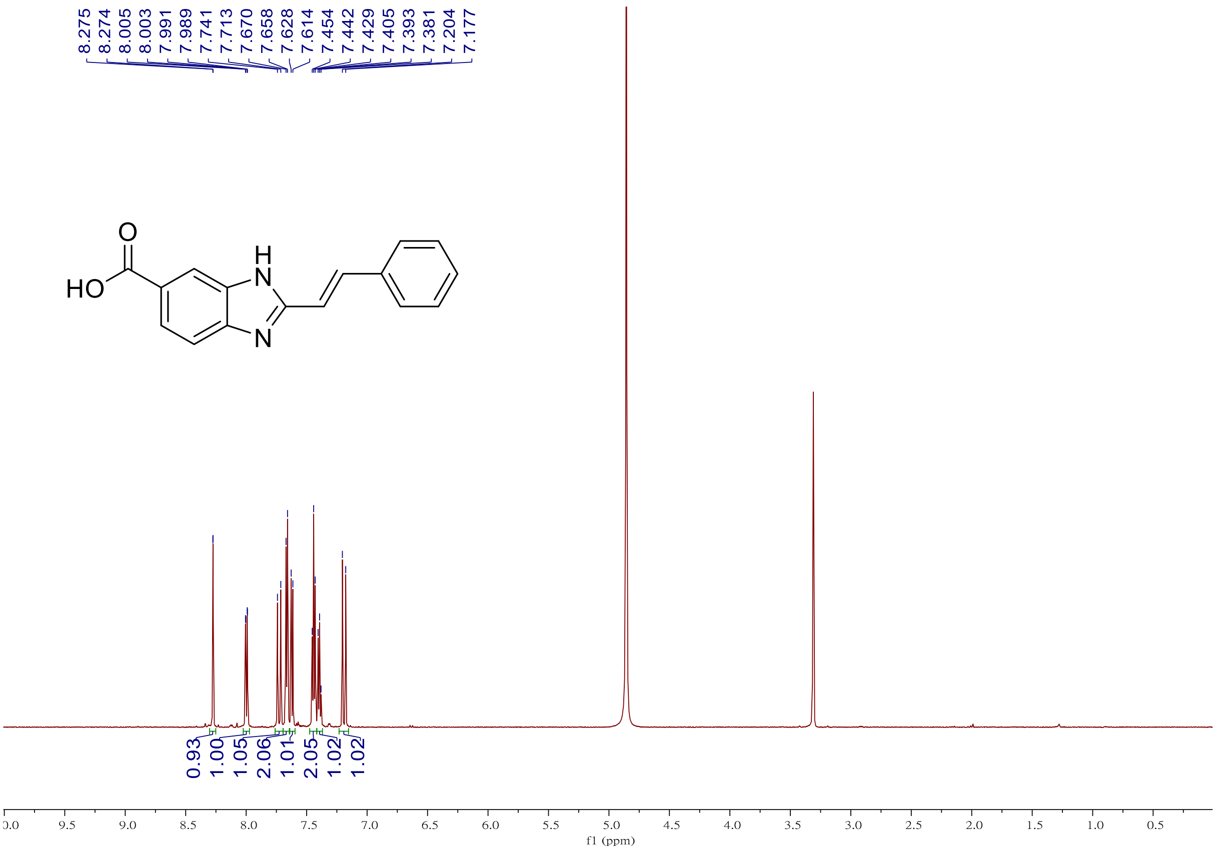


**Figure S41.** $^{1}H$ NMR spectrum of compound **1t** (600 MHz, $CD_3OD$).

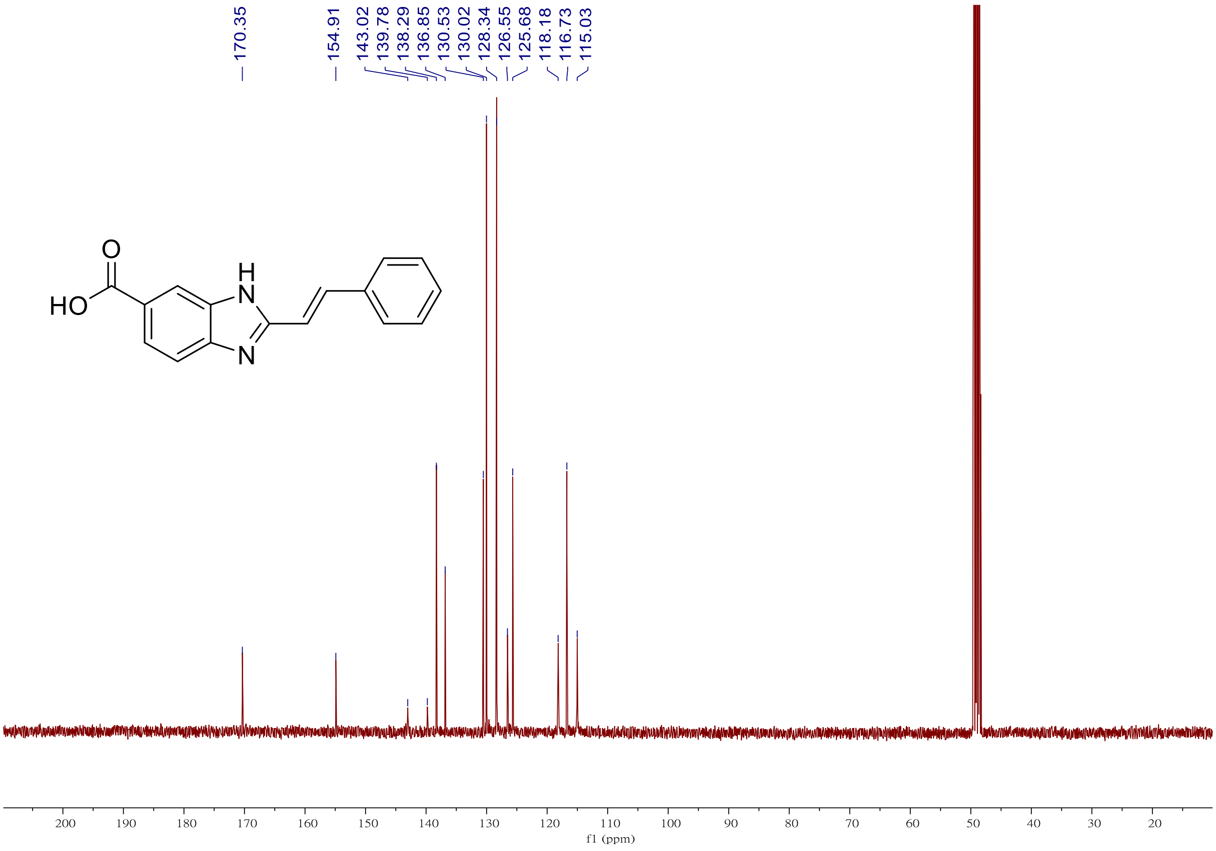


**Figure S42.** $^{13}C\{^{1}H\}$ NMR spectrum of compound **1t** (101 MHz, $CD_3OD$).

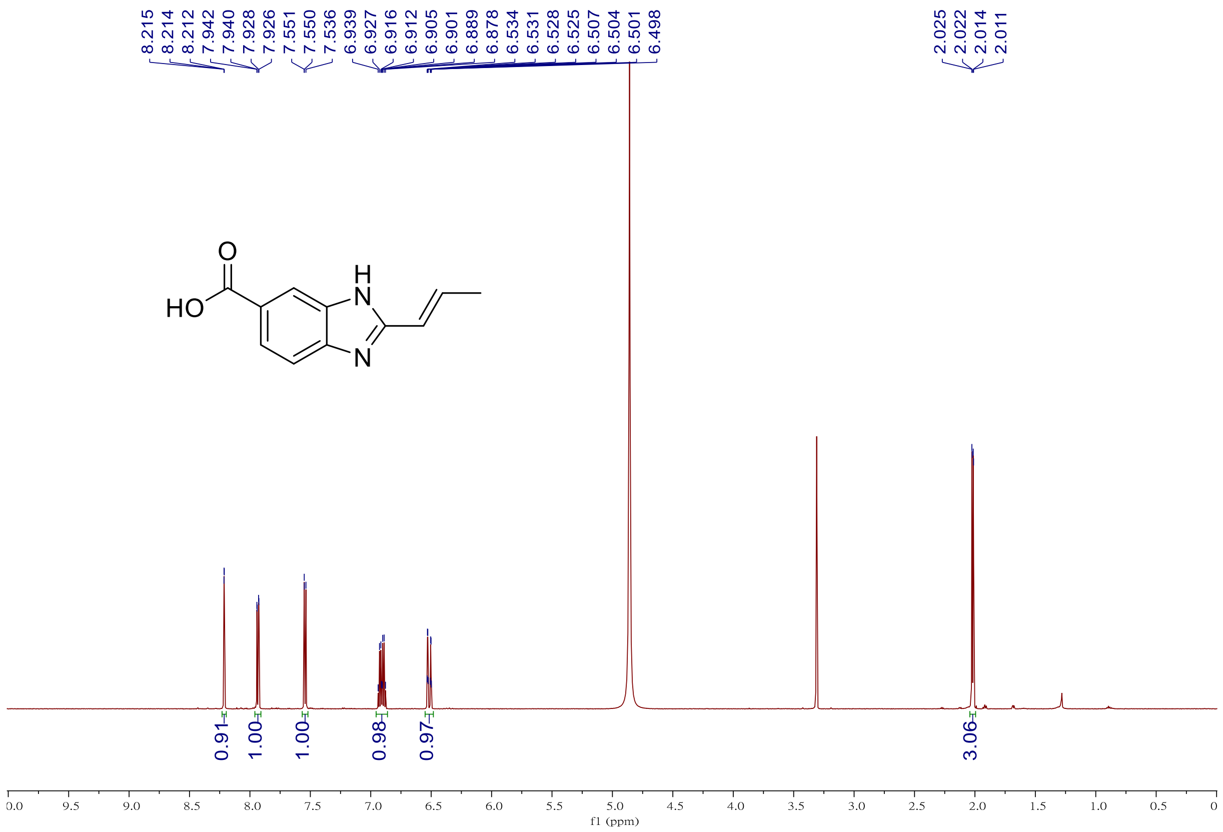


**Figure S43.** $^{1}$H NMR spectrum of compound **1u** (600 MHz, $CD_3OD$).

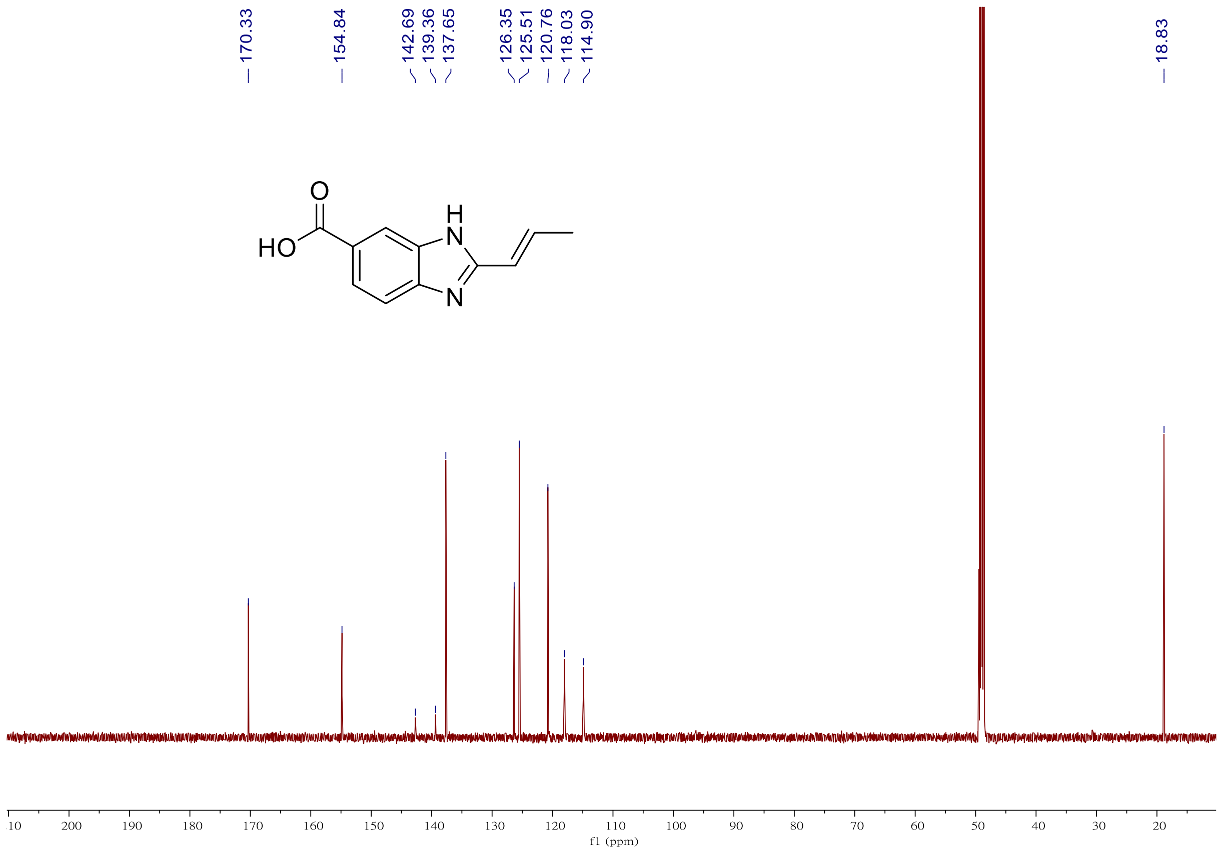


**Figure S44.** $^{13}C\{^{1}H\}$ NMR spectrum of compound **1u** (151 MHz, $CD_3OD$).

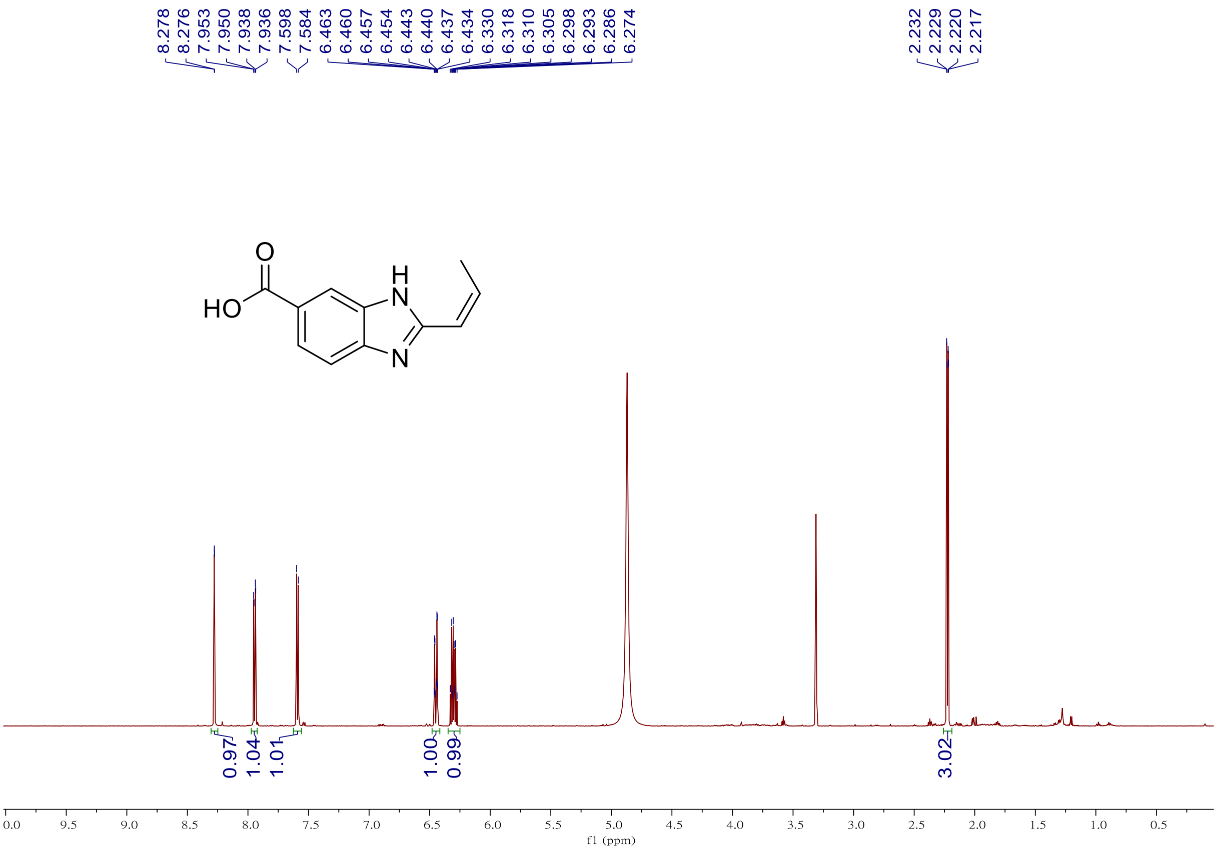


**Figure S45.** $^{1}H$ NMR spectrum of compound **1v** (600 MHz, $CD_3OD$).

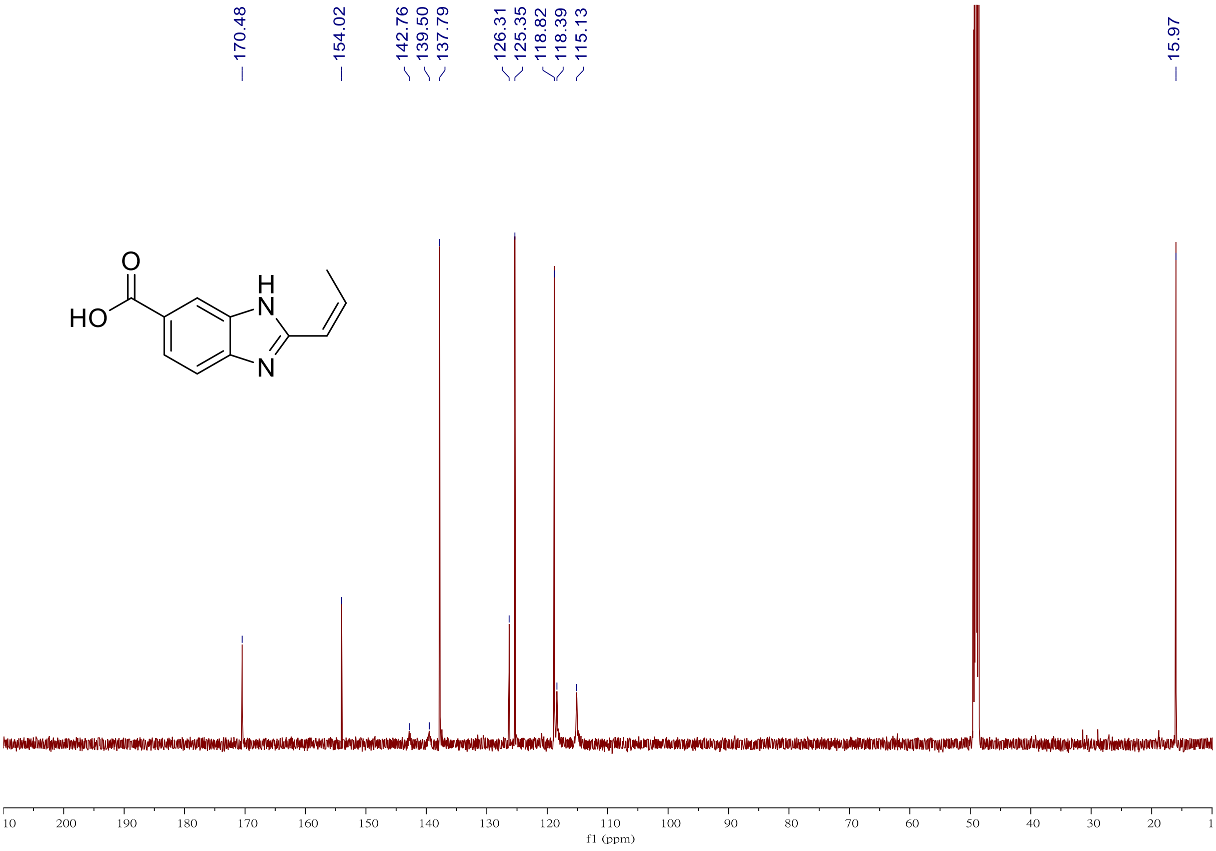


**Figure S46.** $^{13}C\{^{1}H\}$ NMR spectrum of compound **1v** (151 MHz, $CD_3OD$).

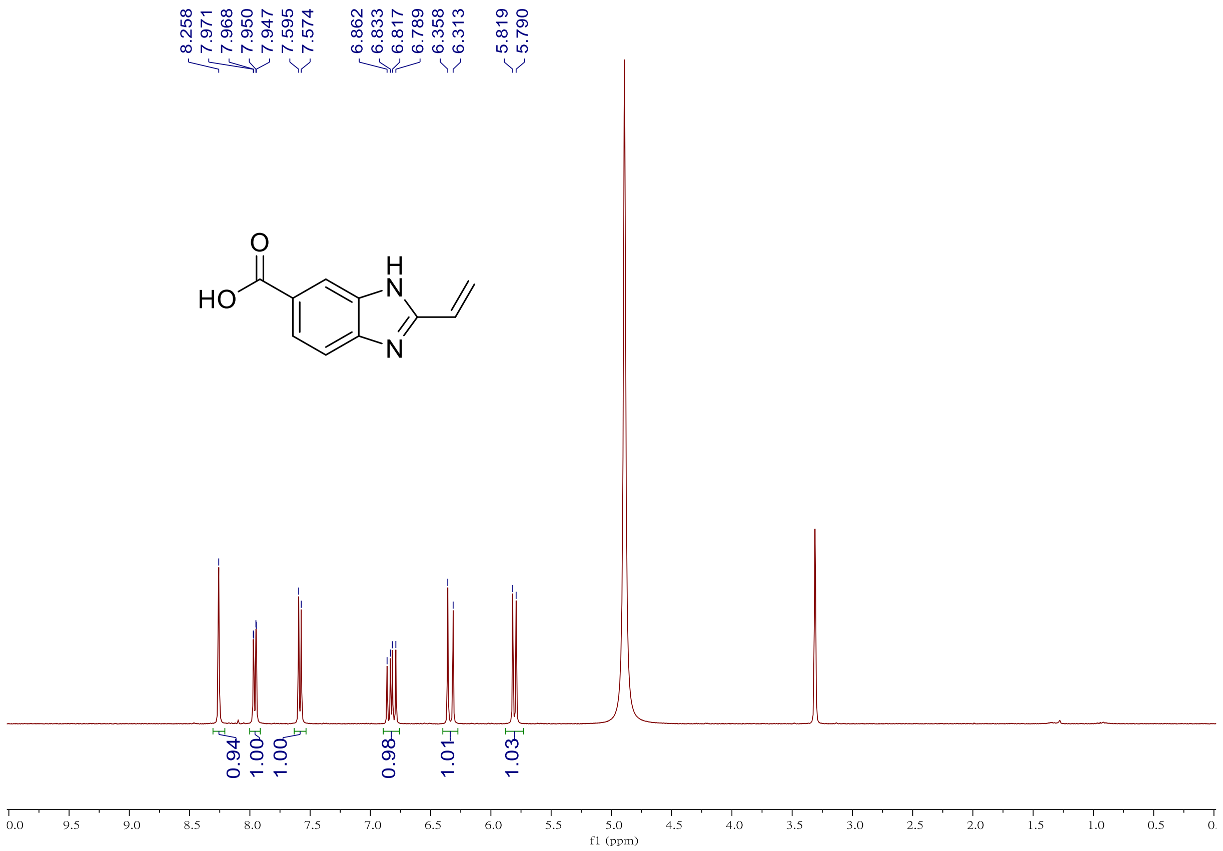


**Figure S47.** $^1$H NMR spectrum of compound **1w** (400 MHz, $CD_3OD$).

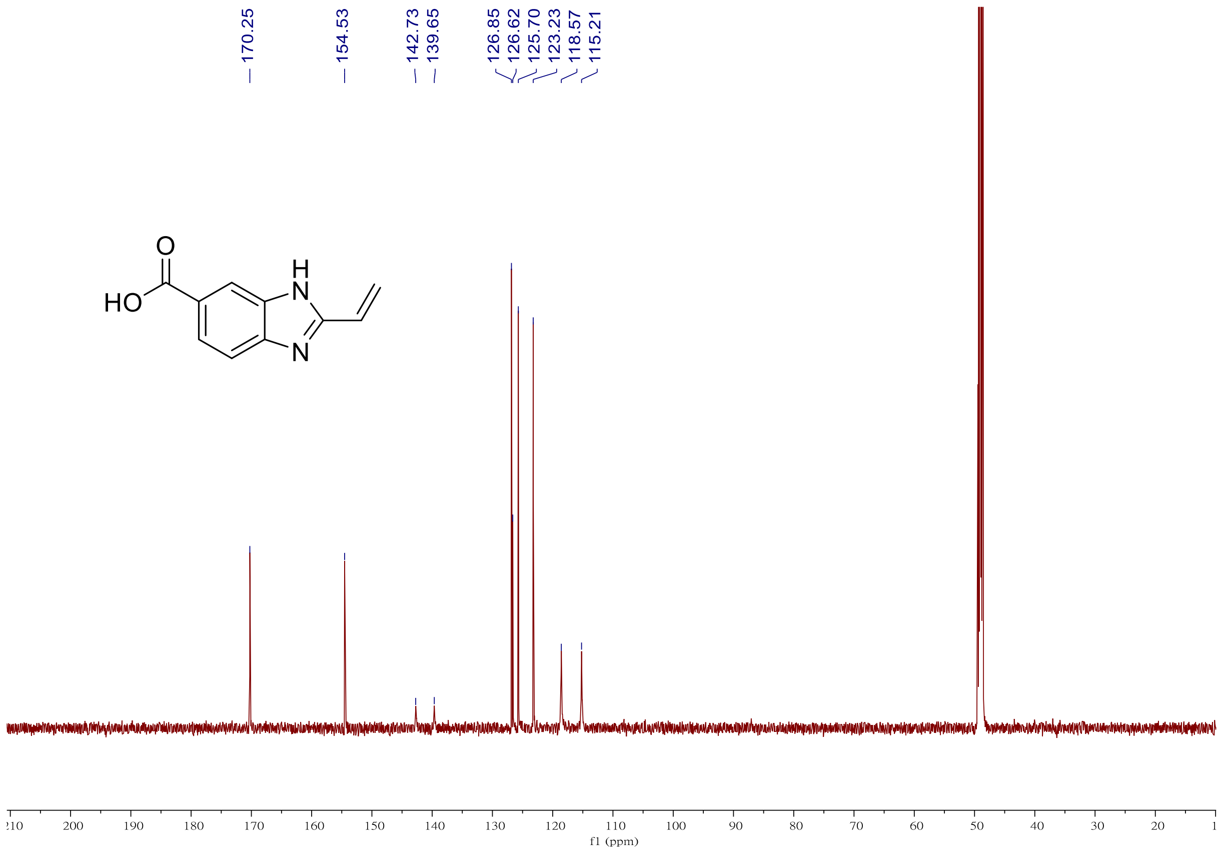


**Figure S48.** $^{13}C\{^1H\}$ NMR spectrum of compound **1w** (151 MHz, $CD_3OD$).

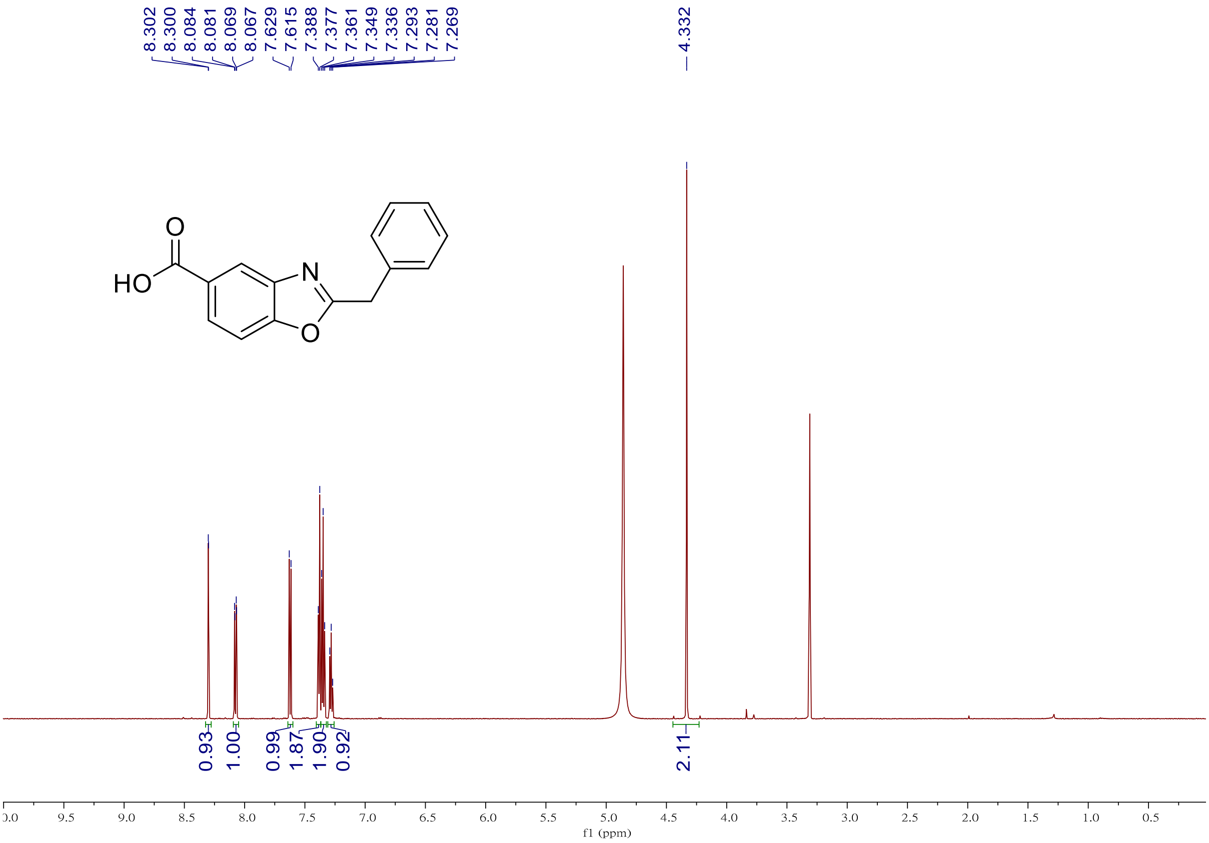


**Figure S49.** $^{1}H$ NMR spectrum of compound **1x** (600 MHz, $CD_3OD$).

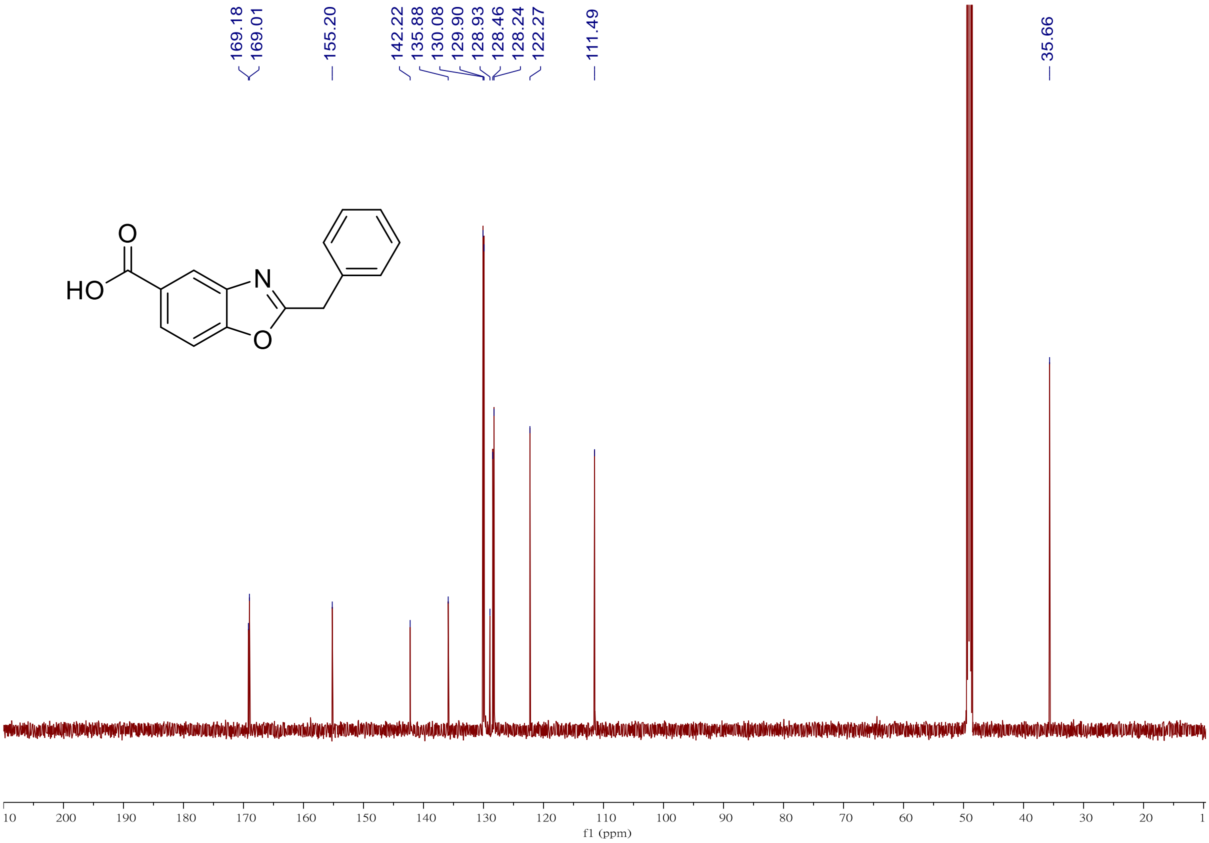


**Figure S50.** $^{13}C\{^{1}H\}$ NMR spectrum of compound **1x** (151 MHz, $CD_3OD$).

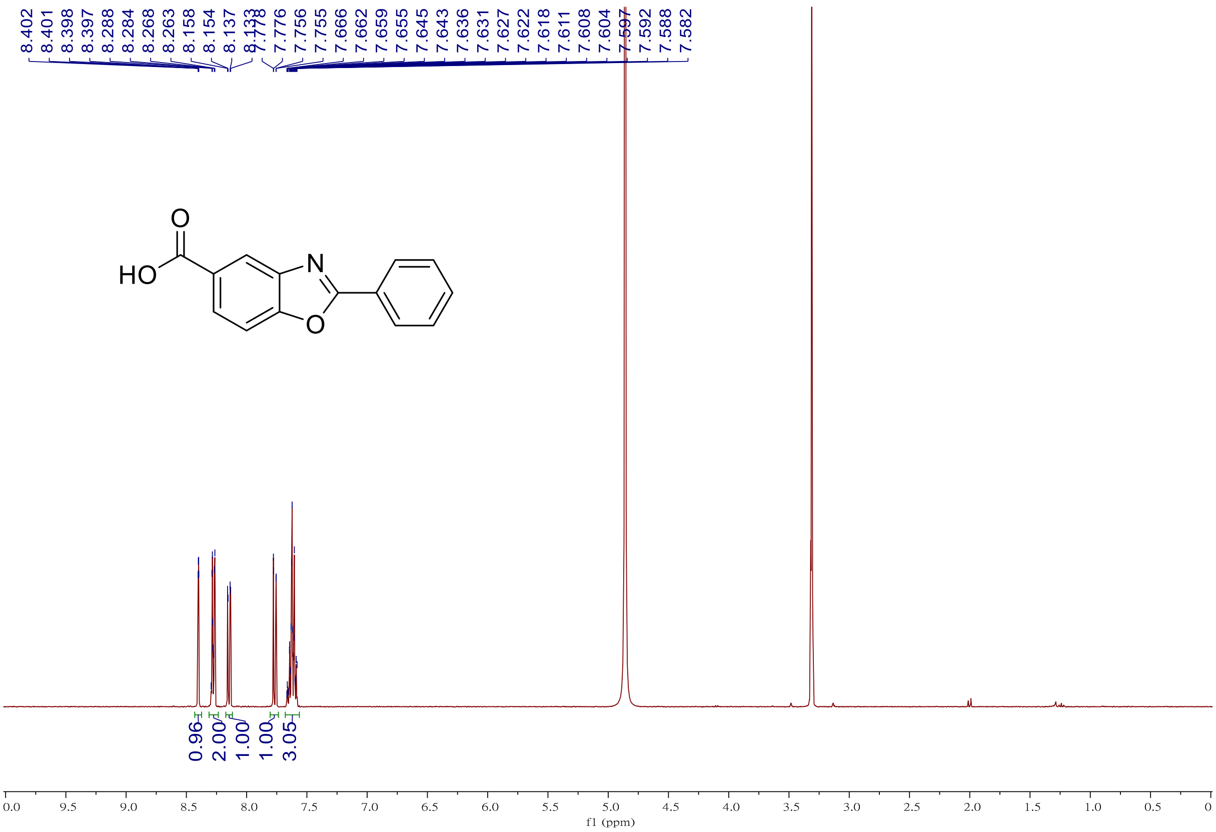


**Figure S51.** $^{1}H$ NMR spectrum of compound **1y** (400 MHz, $CD_3OD$).

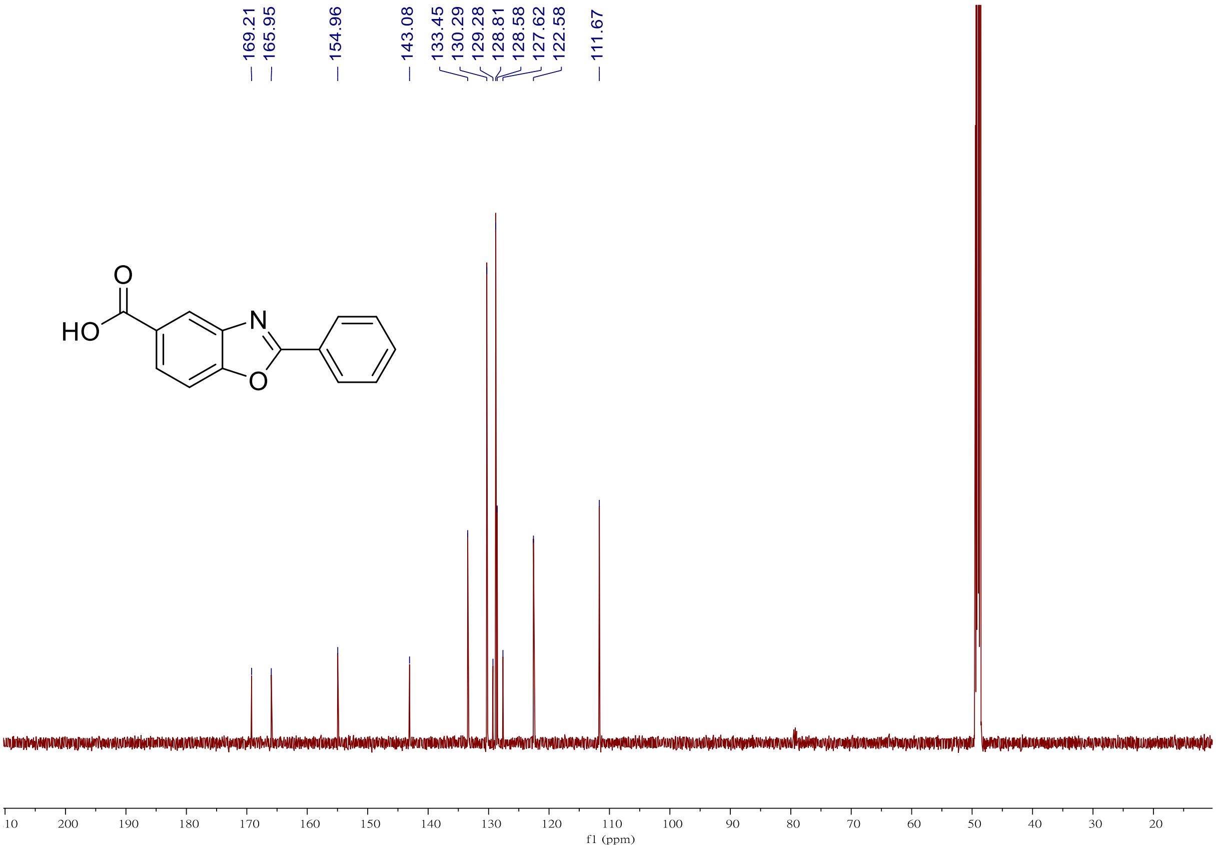


**Figure S52.** $^{13}C\{^{1}H\}$ NMR spectrum of compound **1y** (151 MHz, $CD_3OD$).

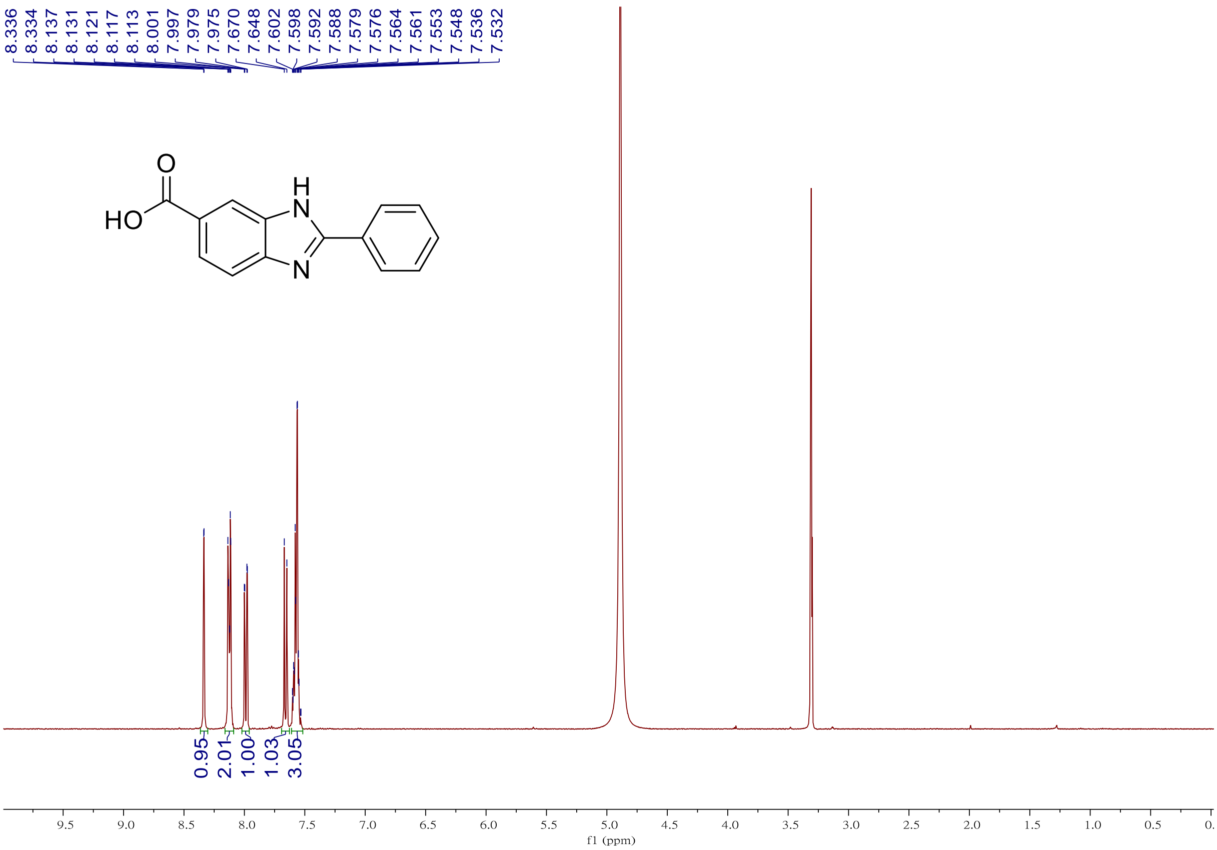

**Figure S53.** $^{1}$H NMR spectrum of compound **1z** (400 MHz, $CD_3OD$).

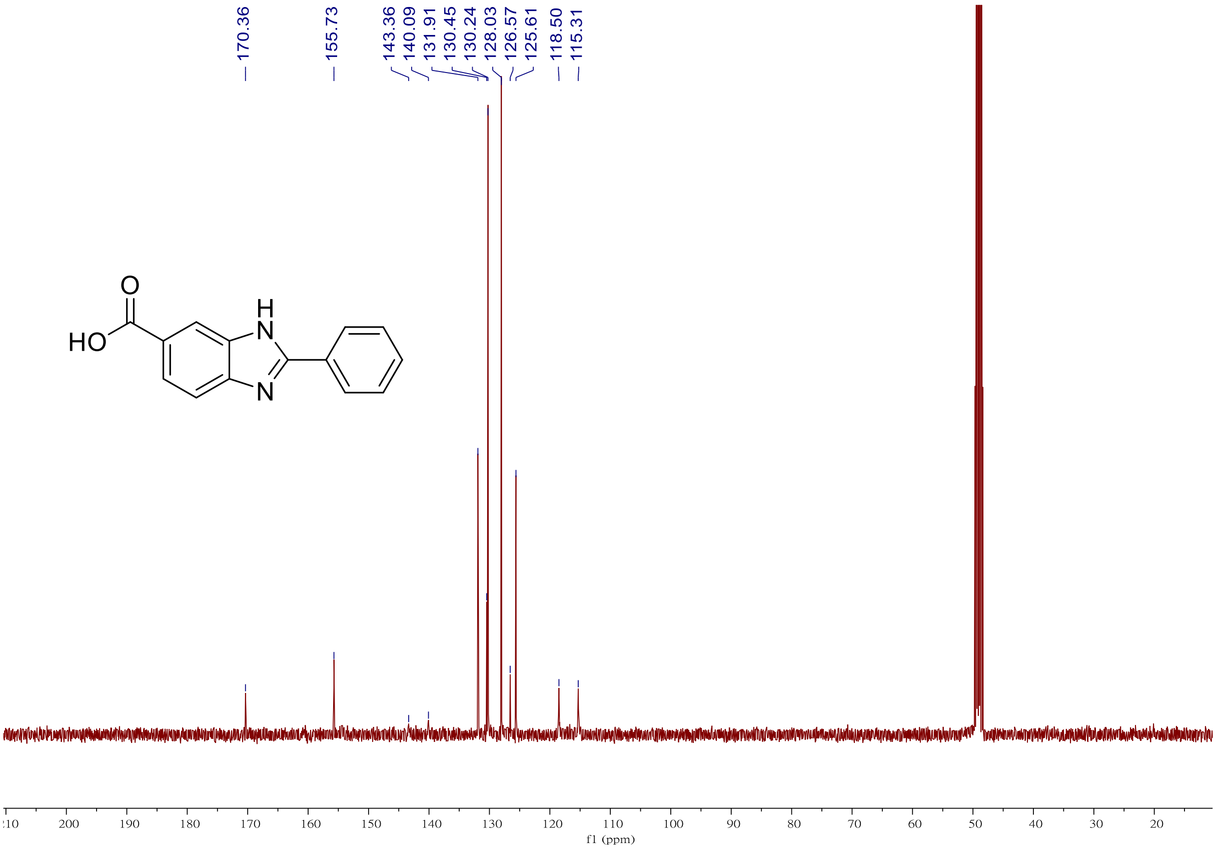

**Figure S54.** $^{13}C\{^{1}H\}$ NMR spectrum of compound **1z** (101 MHz, $CD_3OD$).

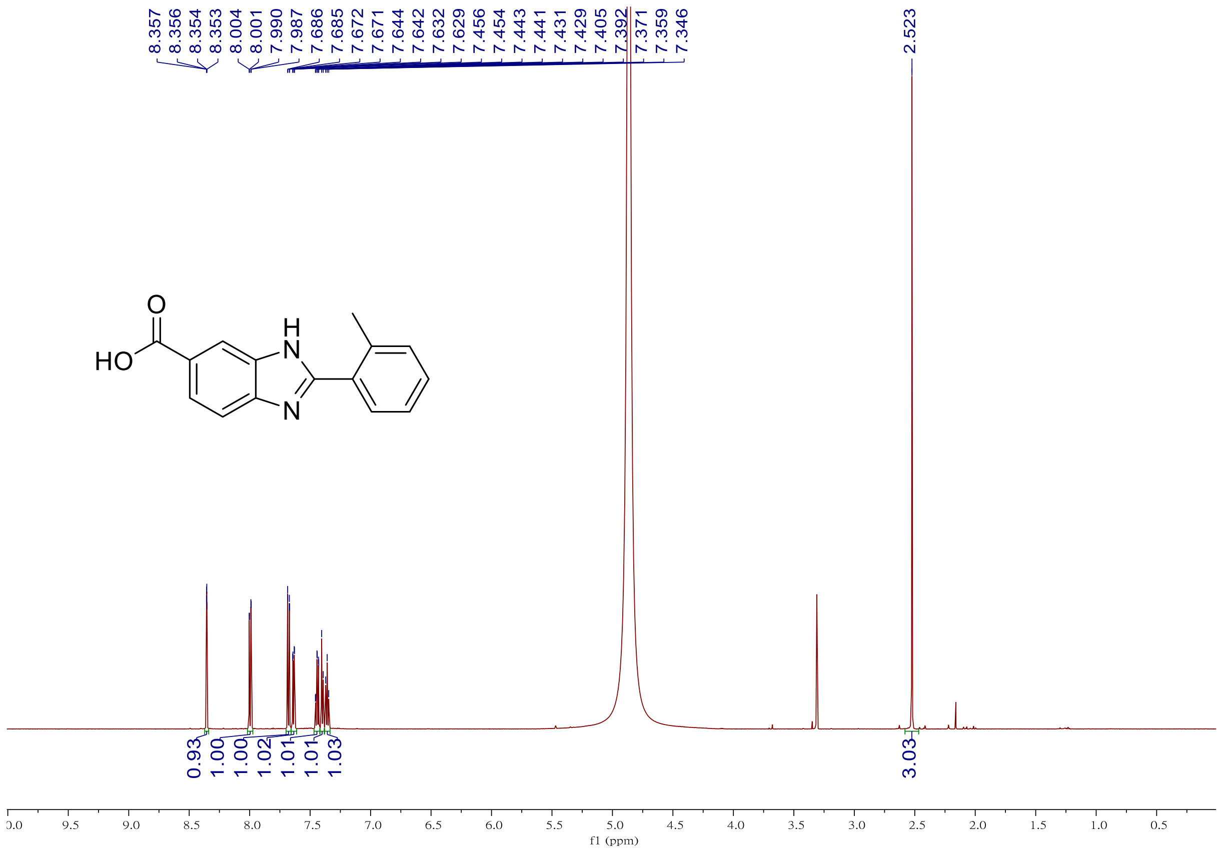

**Figure S55.** $^{1}H$ NMR spectrum of compound **1aa** (400 MHz, $CD_3OD$).

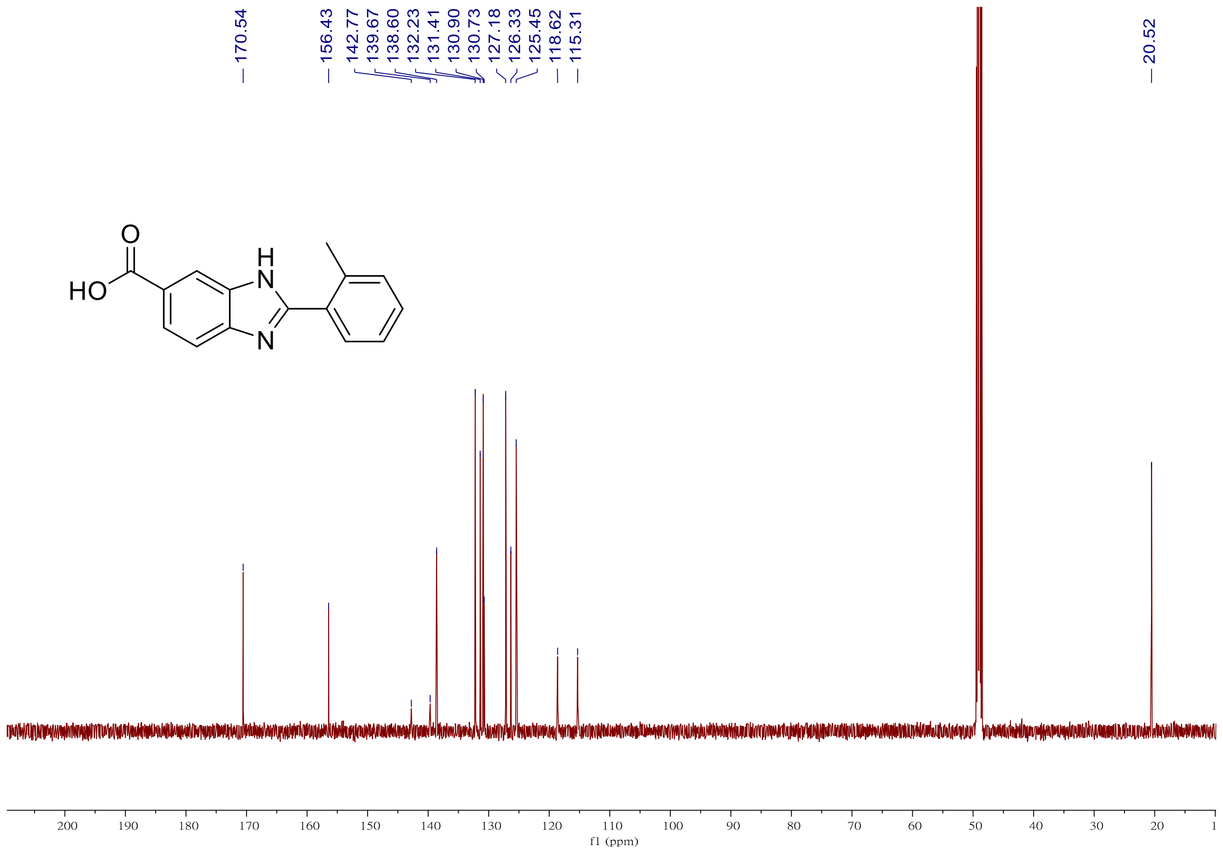

**Figure S56.** $^{13}C\{^{1}H\}$ NMR spectrum of compound **1aa** (151 MHz, $CD_3OD$).

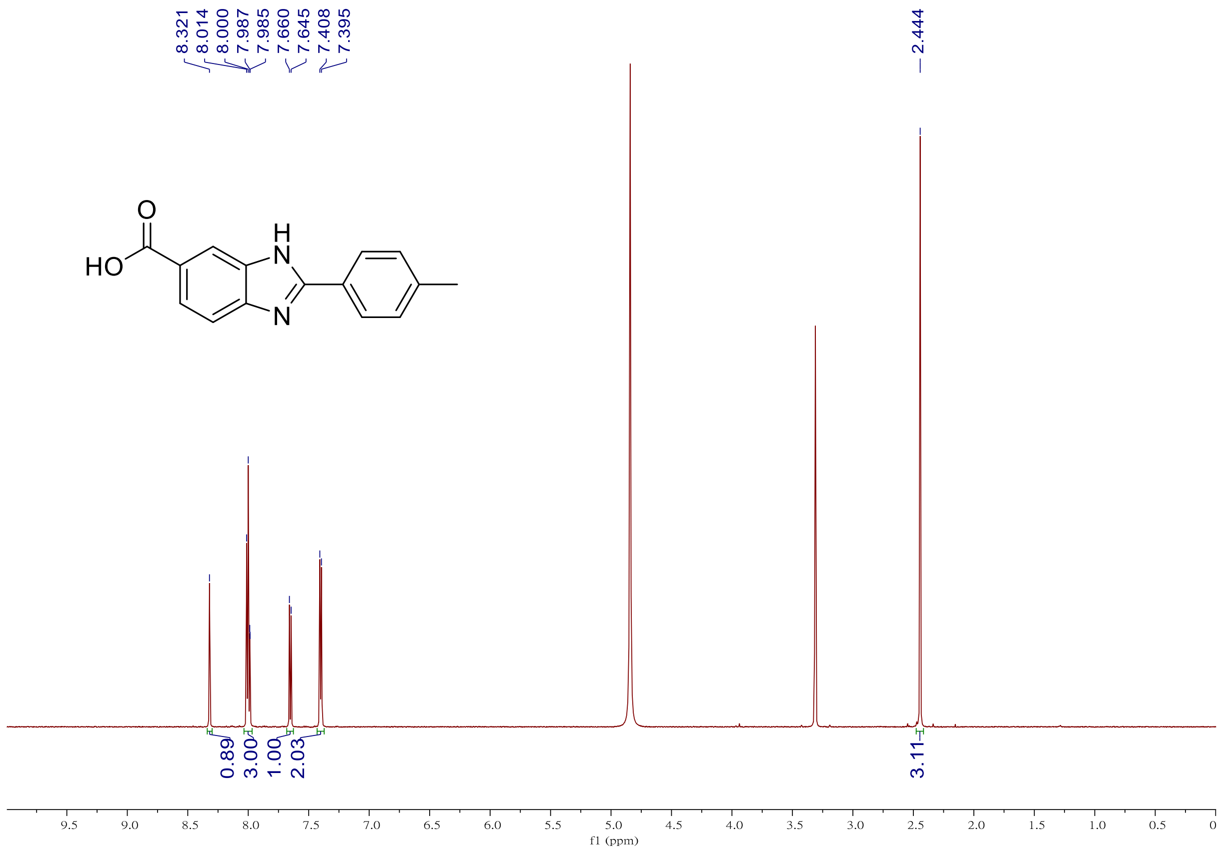


**Figure S57.** $^{1}$H NMR spectrum of compound **1ab** (600 MHz, $CD_3OD$).

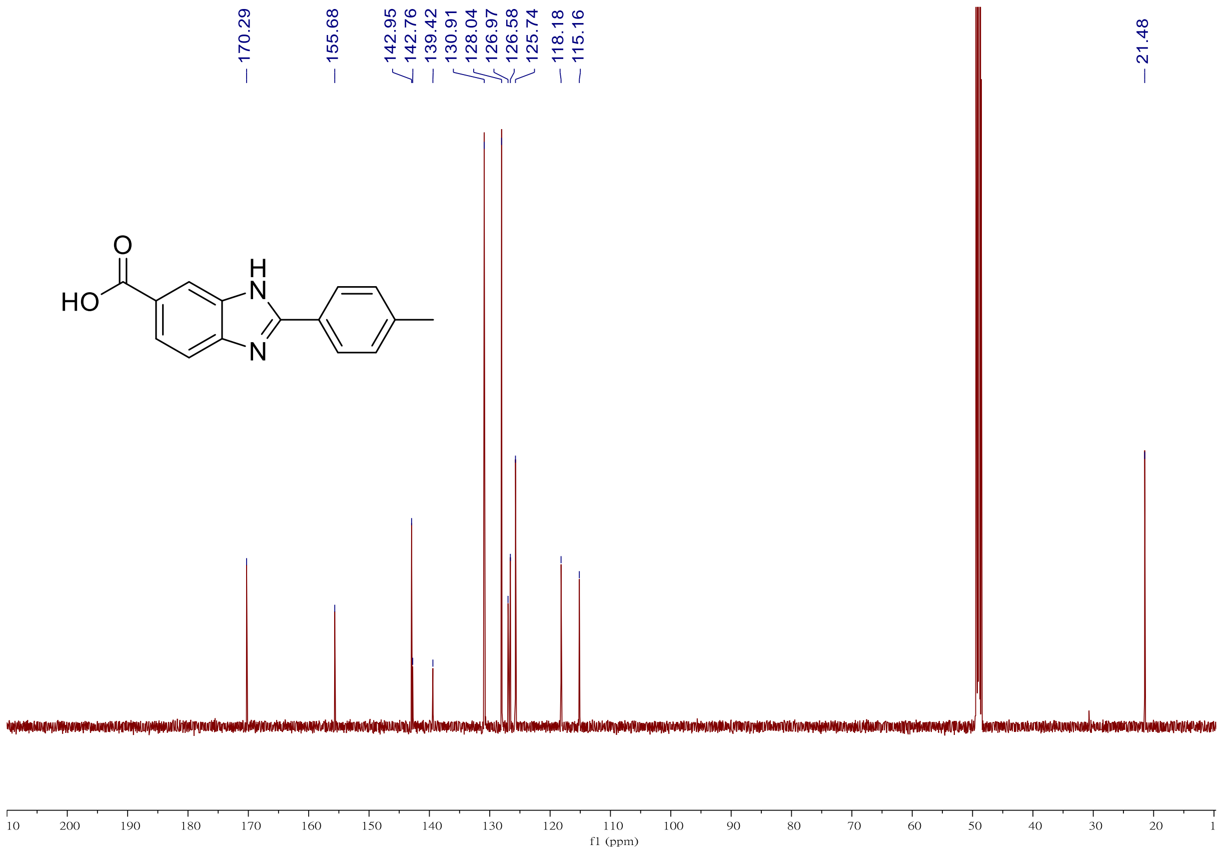


**Figure S58.** $^{13}C\{^{1}H\}$ NMR spectrum of compound **1ab** (151 MHz, $CD_3OD$).

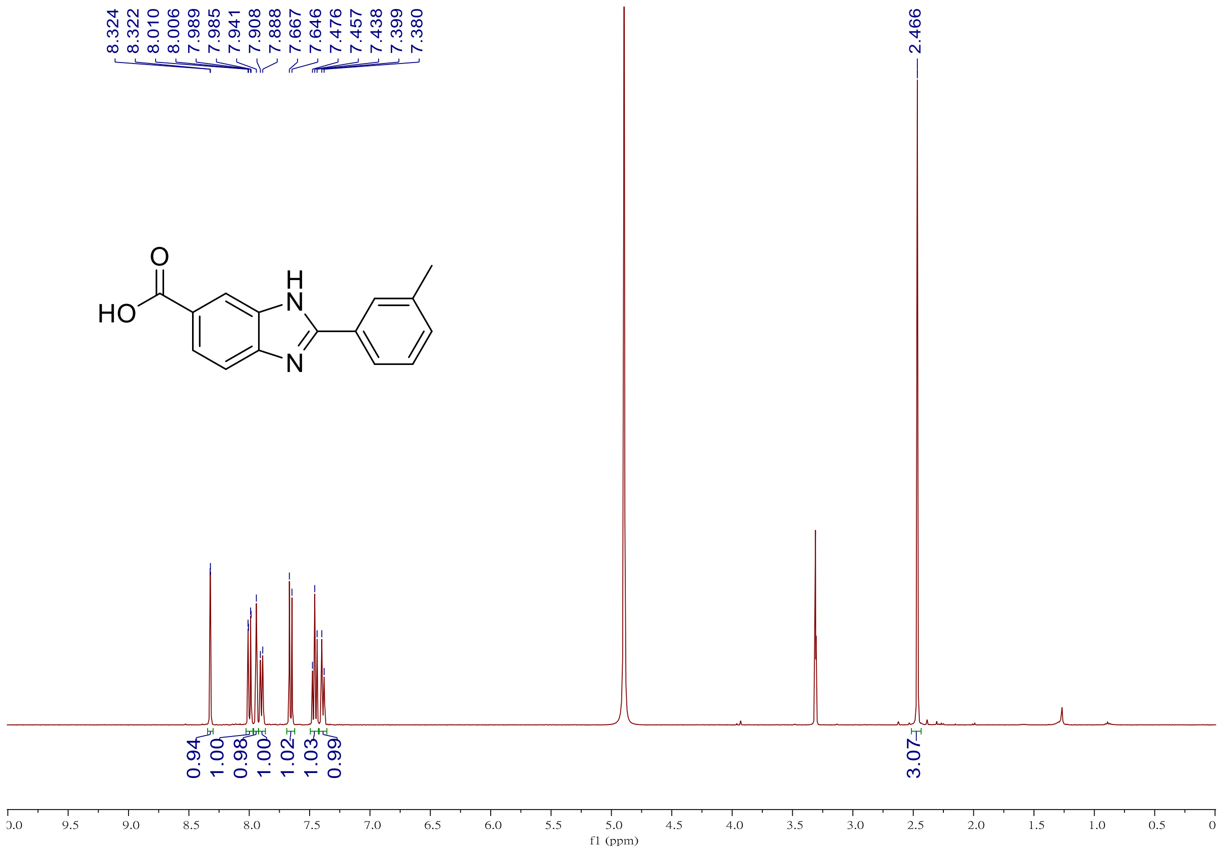


**Figure S59.** $^{1}$H NMR spectrum of compound **1ac** (400 MHz, $CD_3OD$).

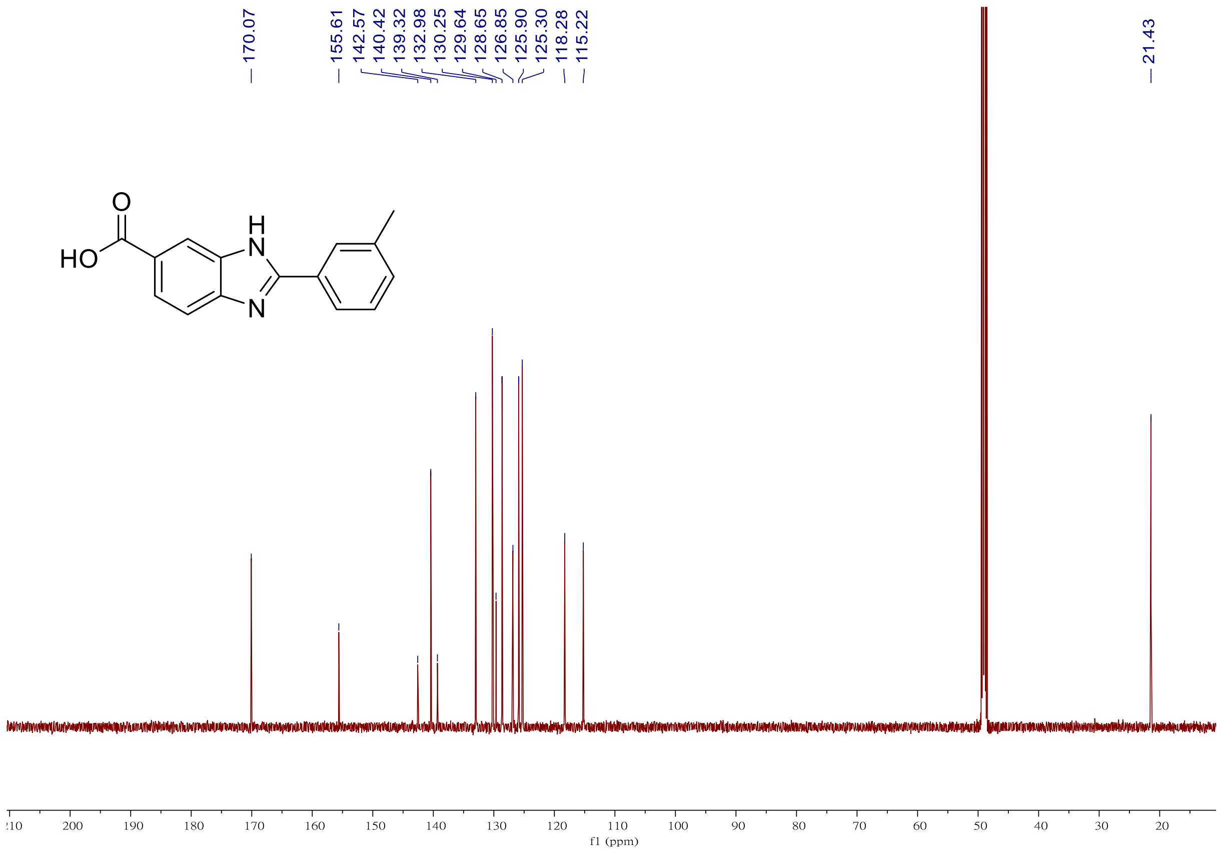


**Figure S60.** $^{13}C\{^{1}H\}$ NMR spectrum of compound **1ac** (151 MHz, $CD_3OD$).

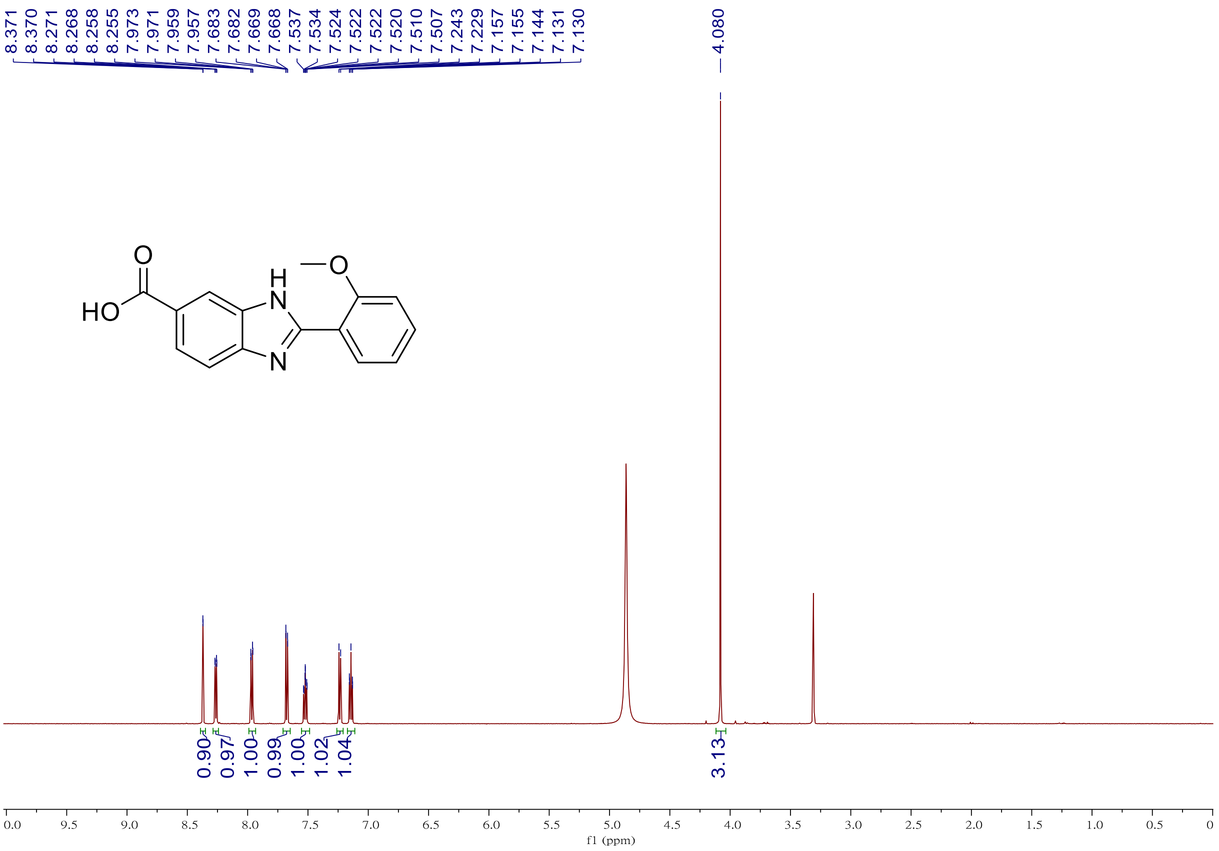

**Figure S61.** $^{1}$H NMR spectrum of compound **1ad** (600 MHz, $CD_3OD$).

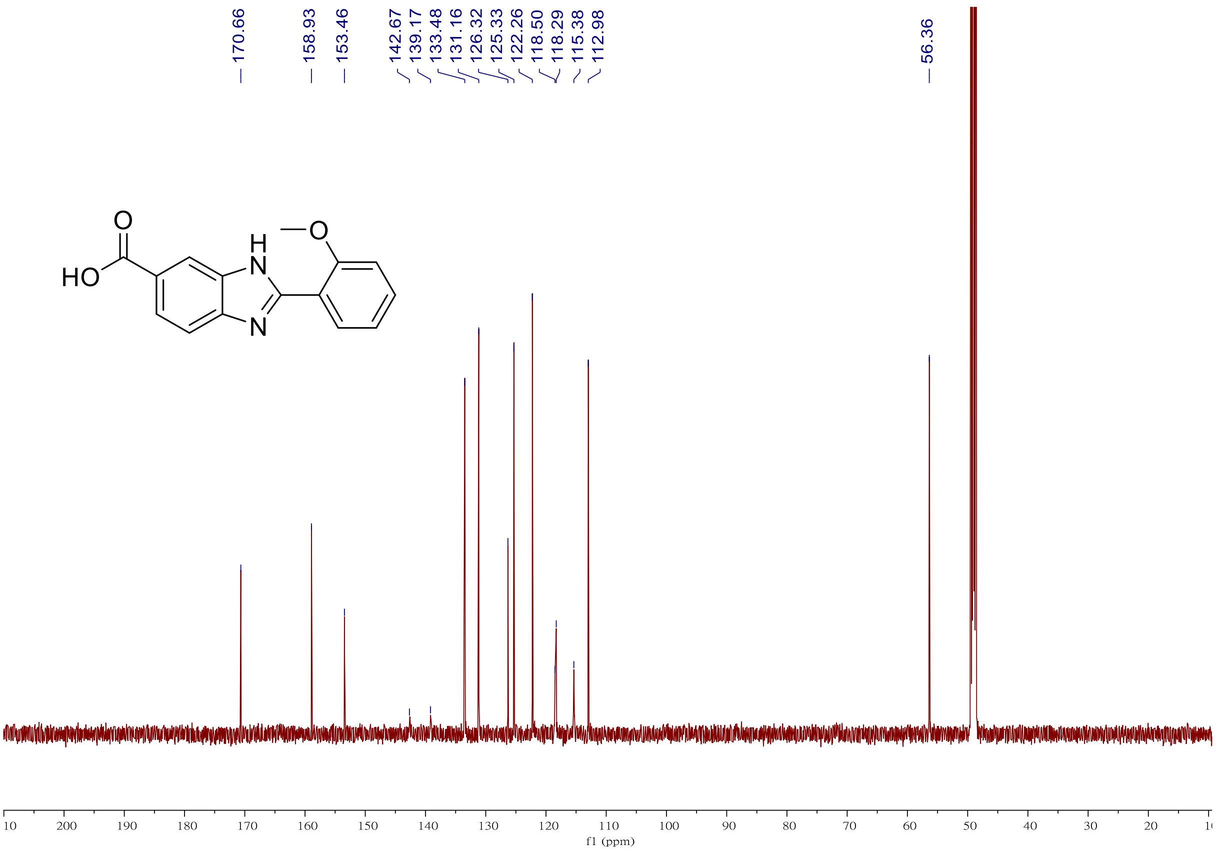

**Figure S62.** $^{13}C\{^{1}H\}$ NMR spectrum of compound **1ad** (151 MHz, $CD_3OD$).

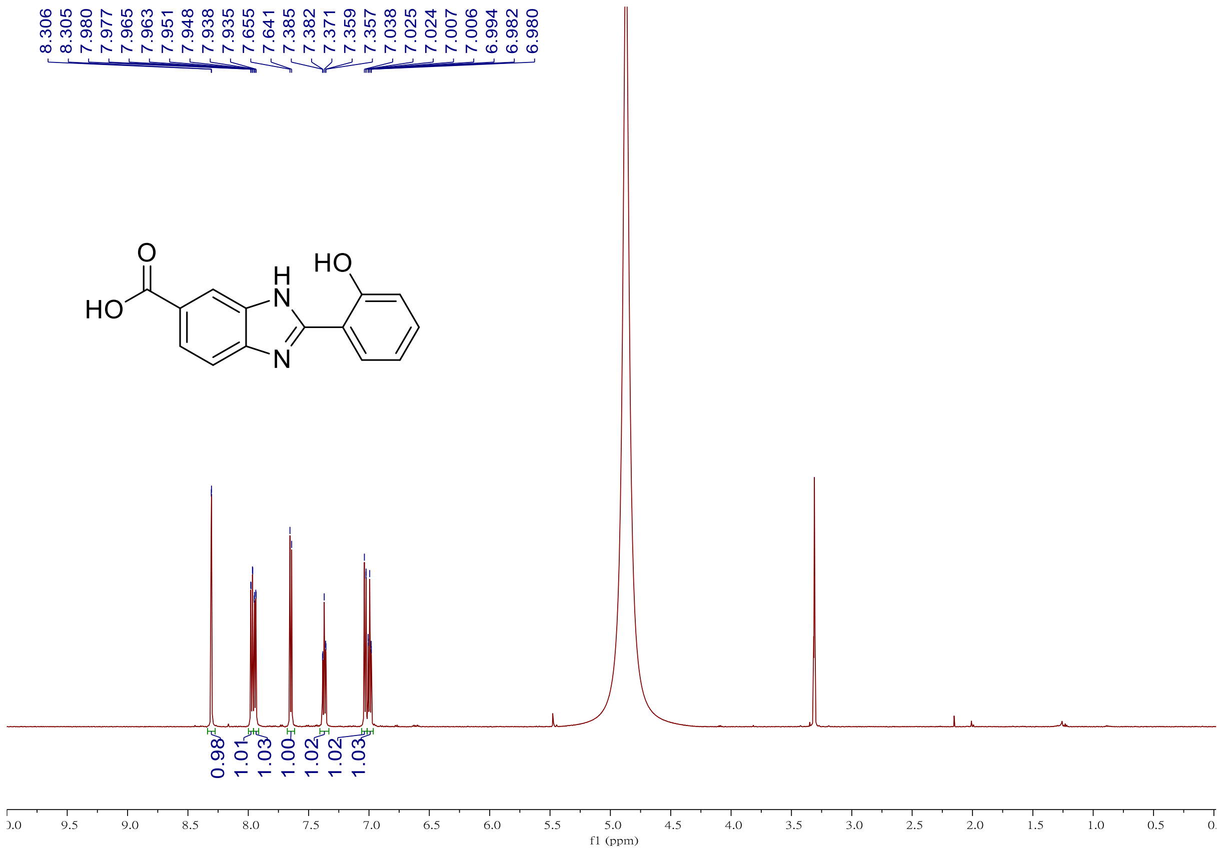


**Figure S63.** $^{1}$H NMR spectrum of compound **1ae** (600 MHz, $CD_3OD$).

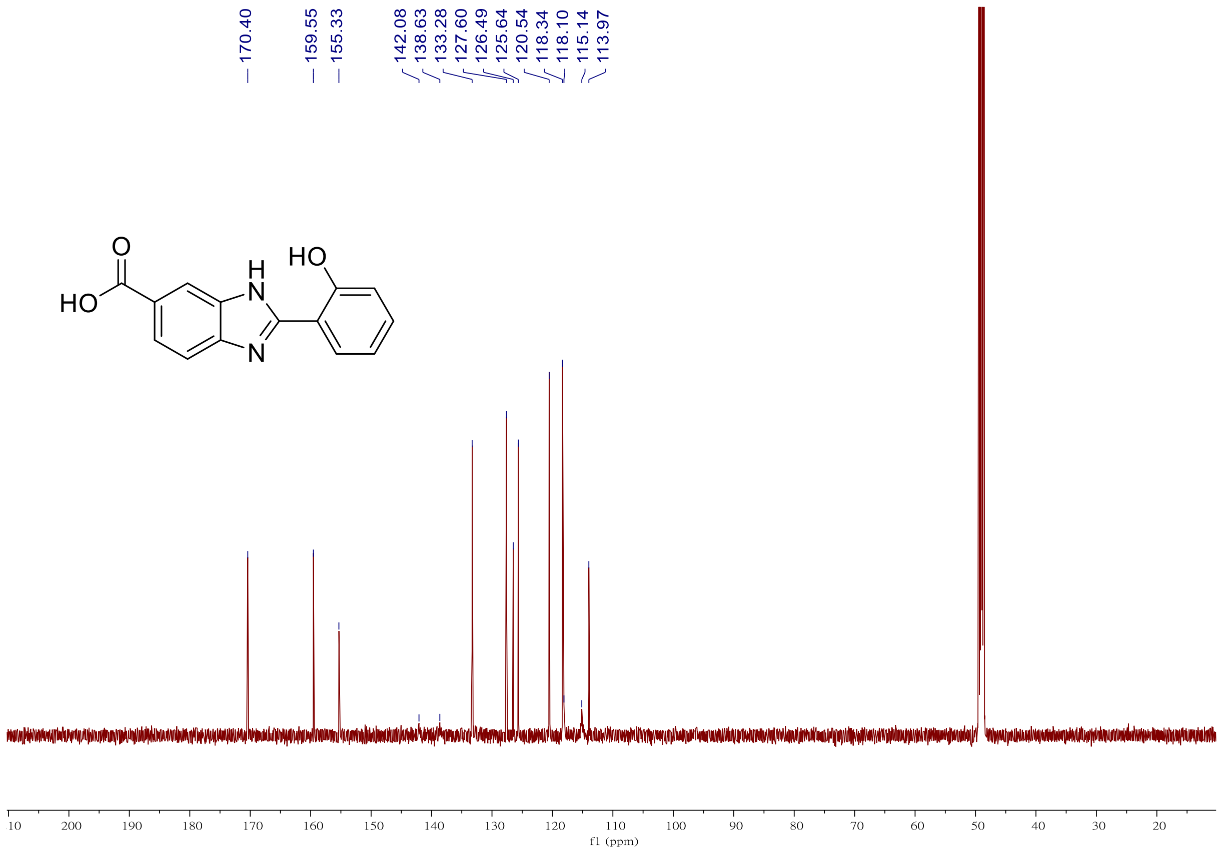


**Figure S64.** $^{13}$C{$^{1}$H} NMR spectrum of compound **1ae** (151 MHz, $CD_3OD$).

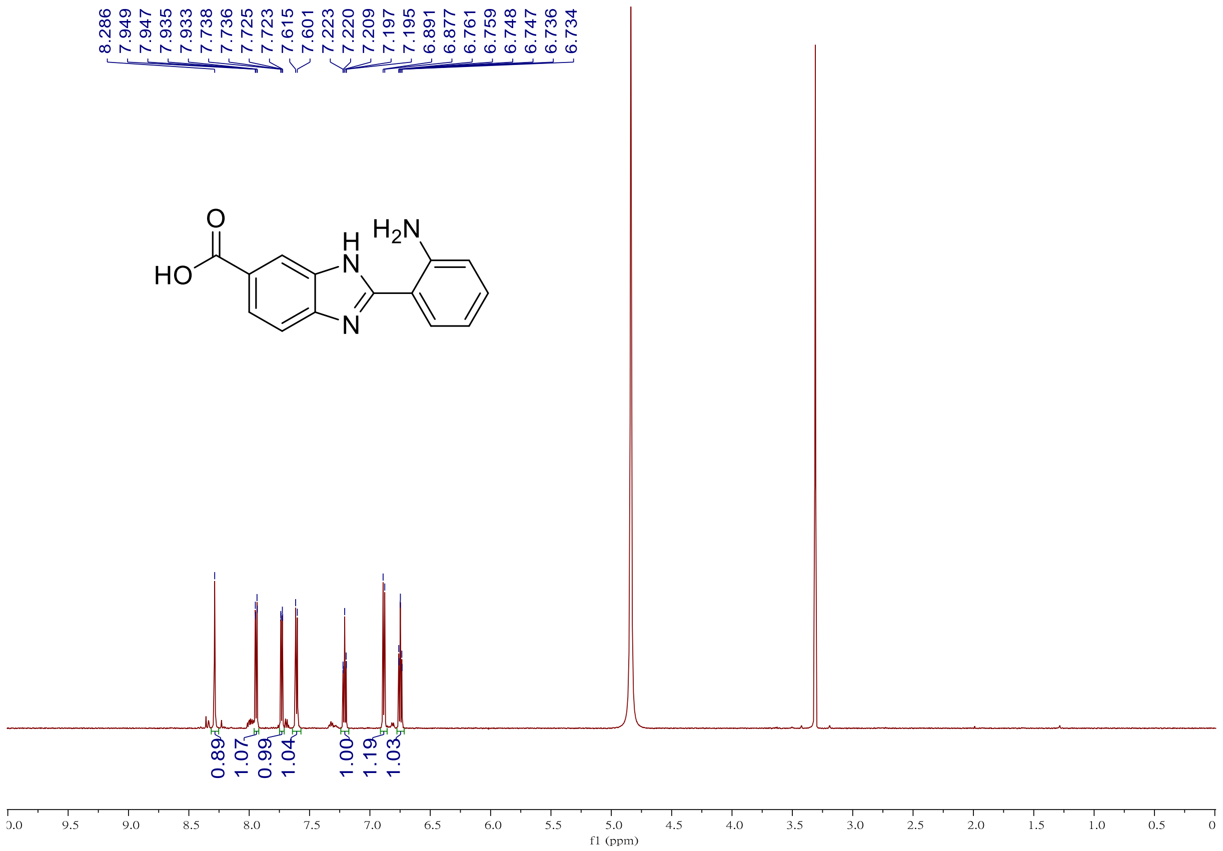


**Figure S65.** $^{1}$H NMR spectrum of compound **1af** (600 MHz, $CD_3OD$).

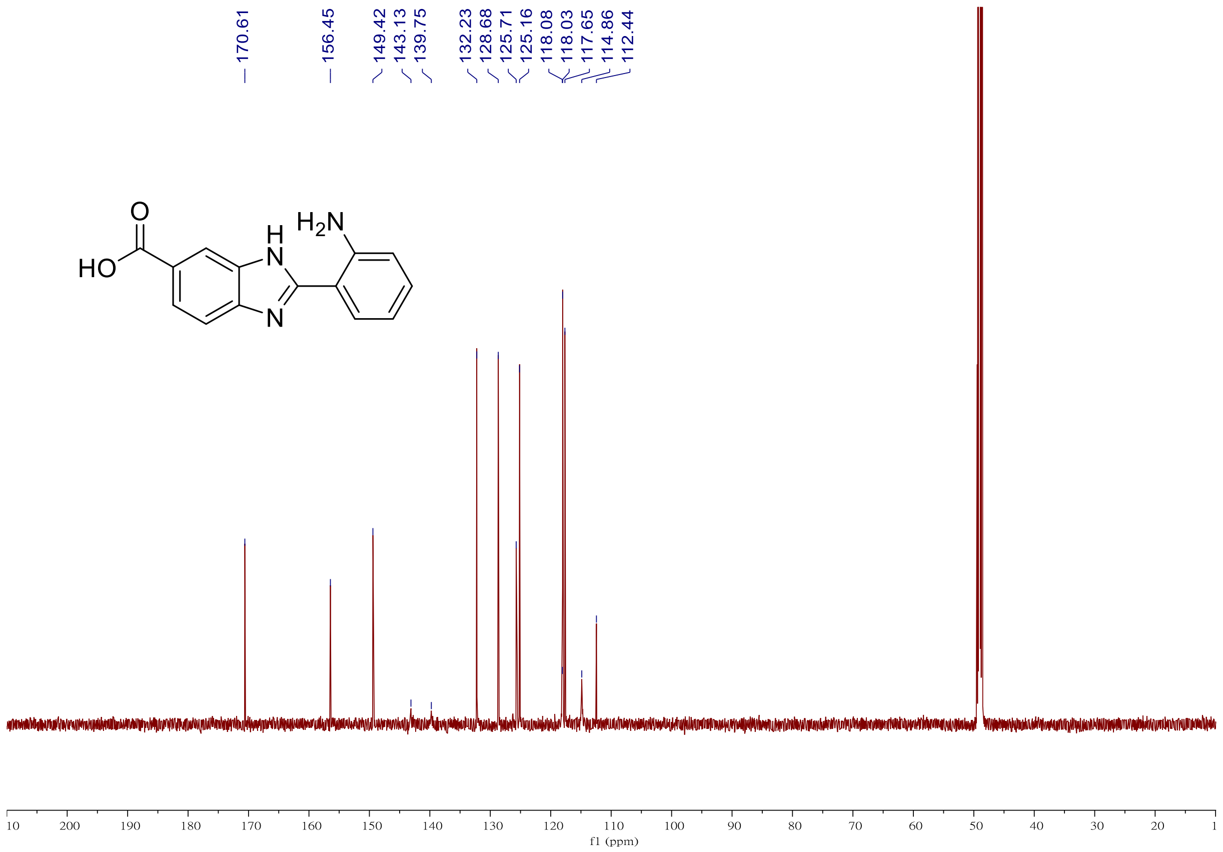


**Figure S66.** $^{13}$C{$^{1}$H} NMR spectrum of compound **1af** (151 MHz, $CD_3OD$).

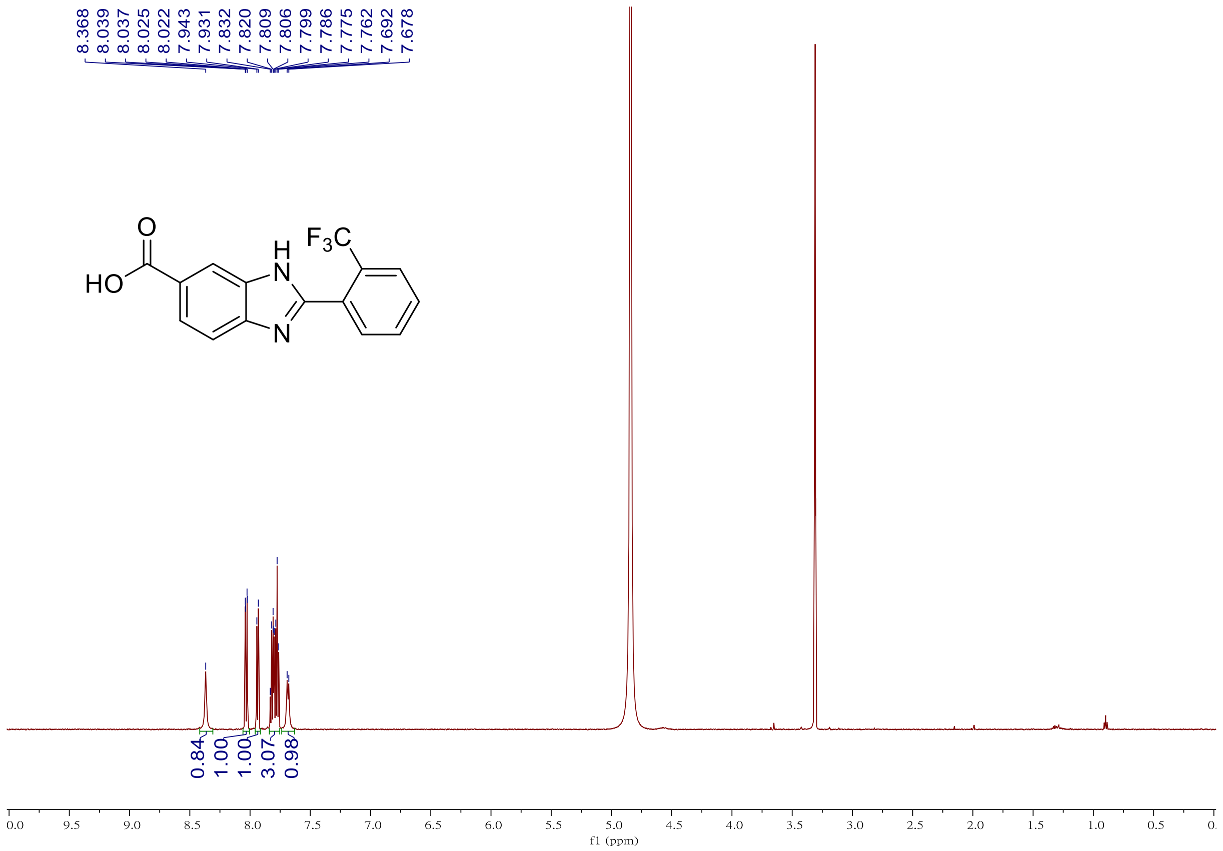


**Figure S67.** $^{1}$H NMR spectrum of compound **1ag** (600 MHz, $CD_3OD$).

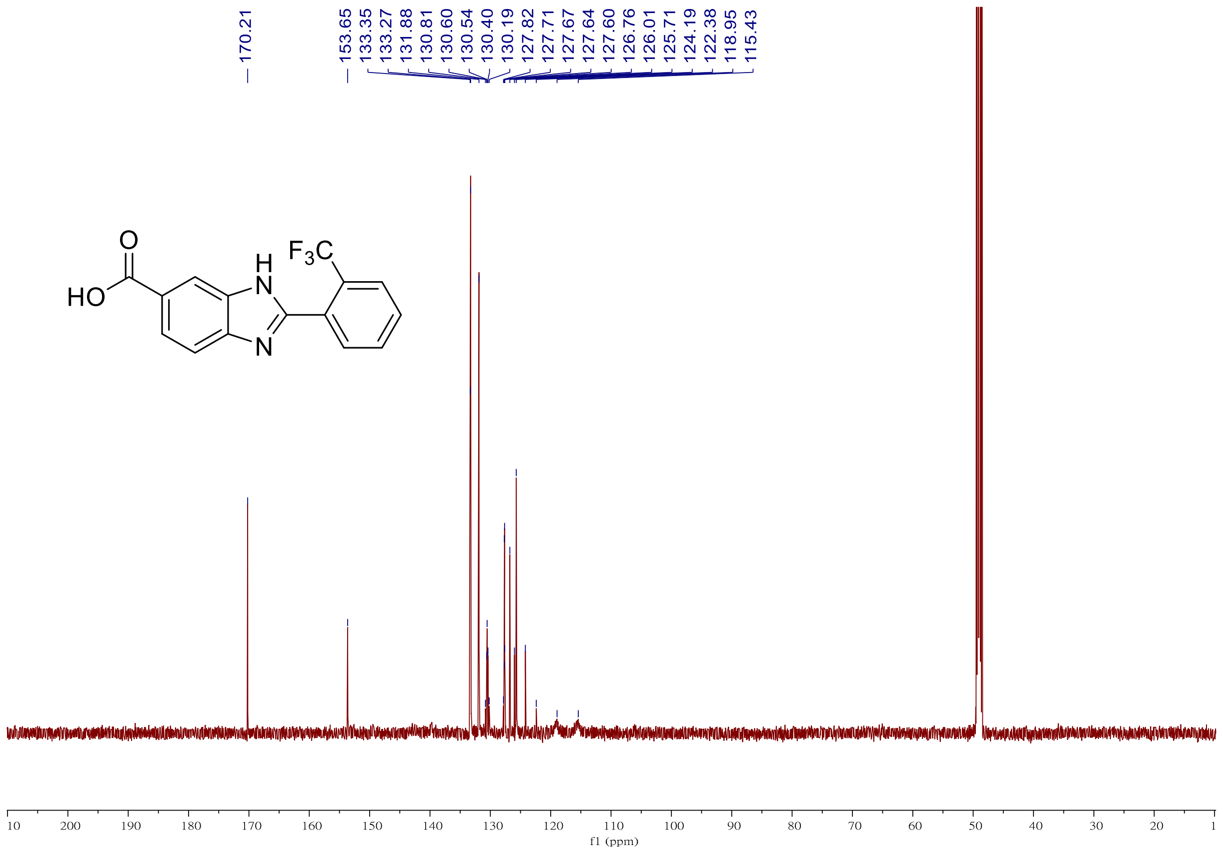


**Figure S68.** $^{13}C\{^{1}H\}$ NMR spectrum of compound **1ag** (151 MHz, $CD_3OD$).

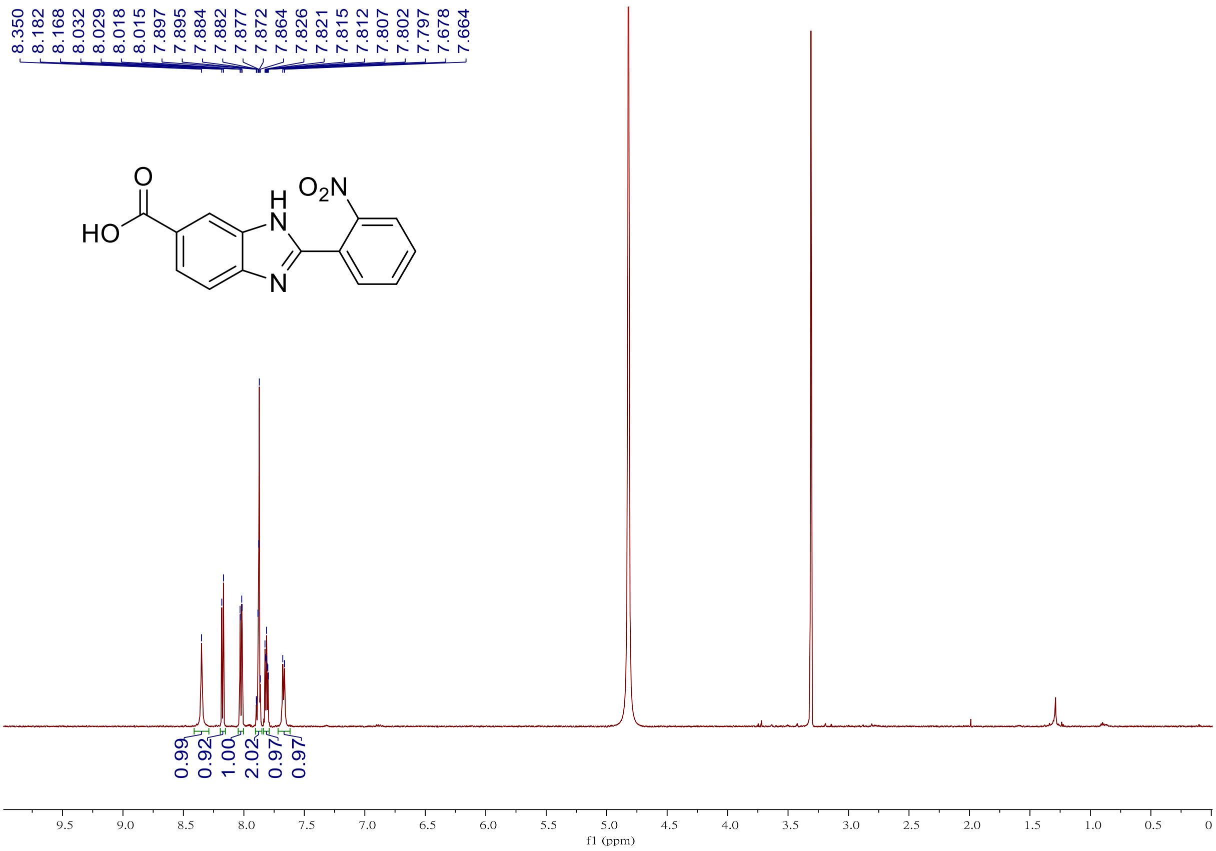


**Figure S69.** $^{1}$H NMR spectrum of compound **1ah** (600 MHz, $CD_3OD$).

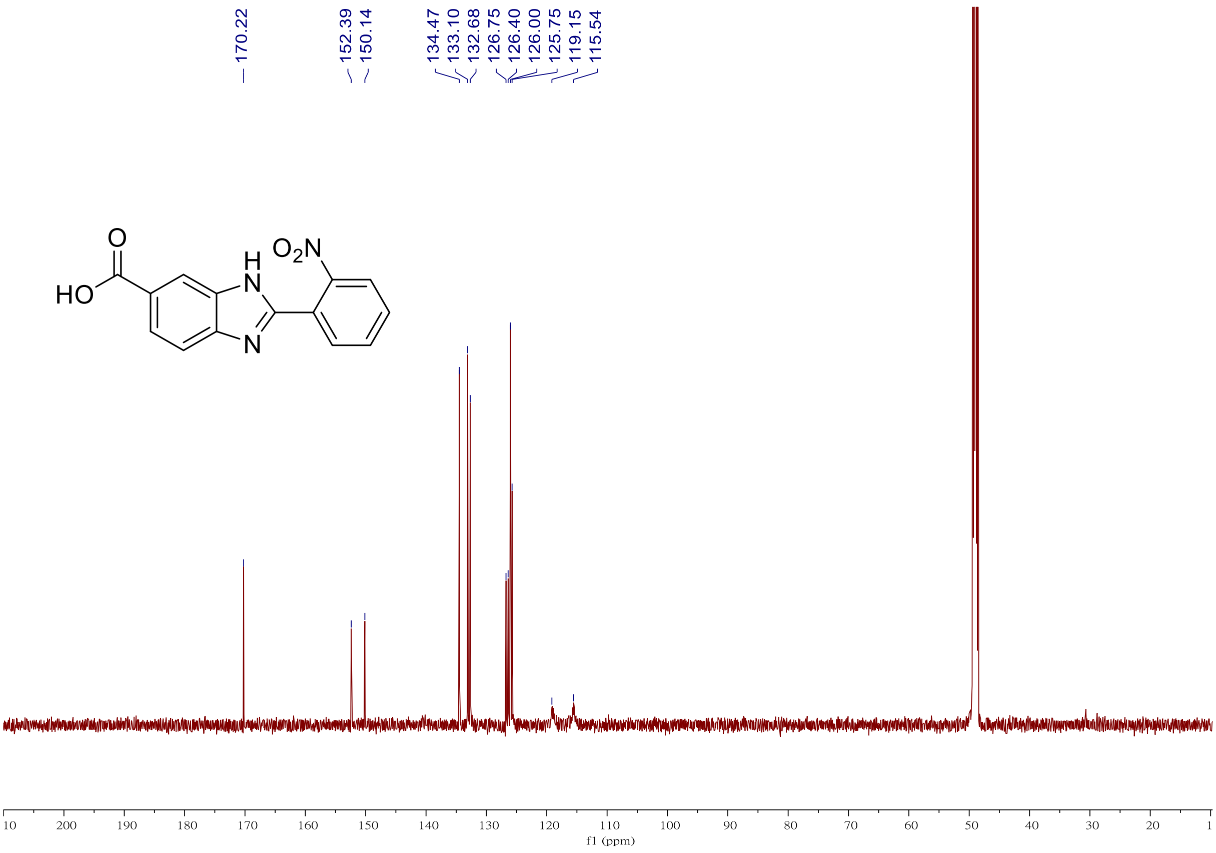


**Figure S70.** $^{13}$C{$^{1}$H} NMR spectrum of compound **1ah** (151 MHz, $CD_3OD$).

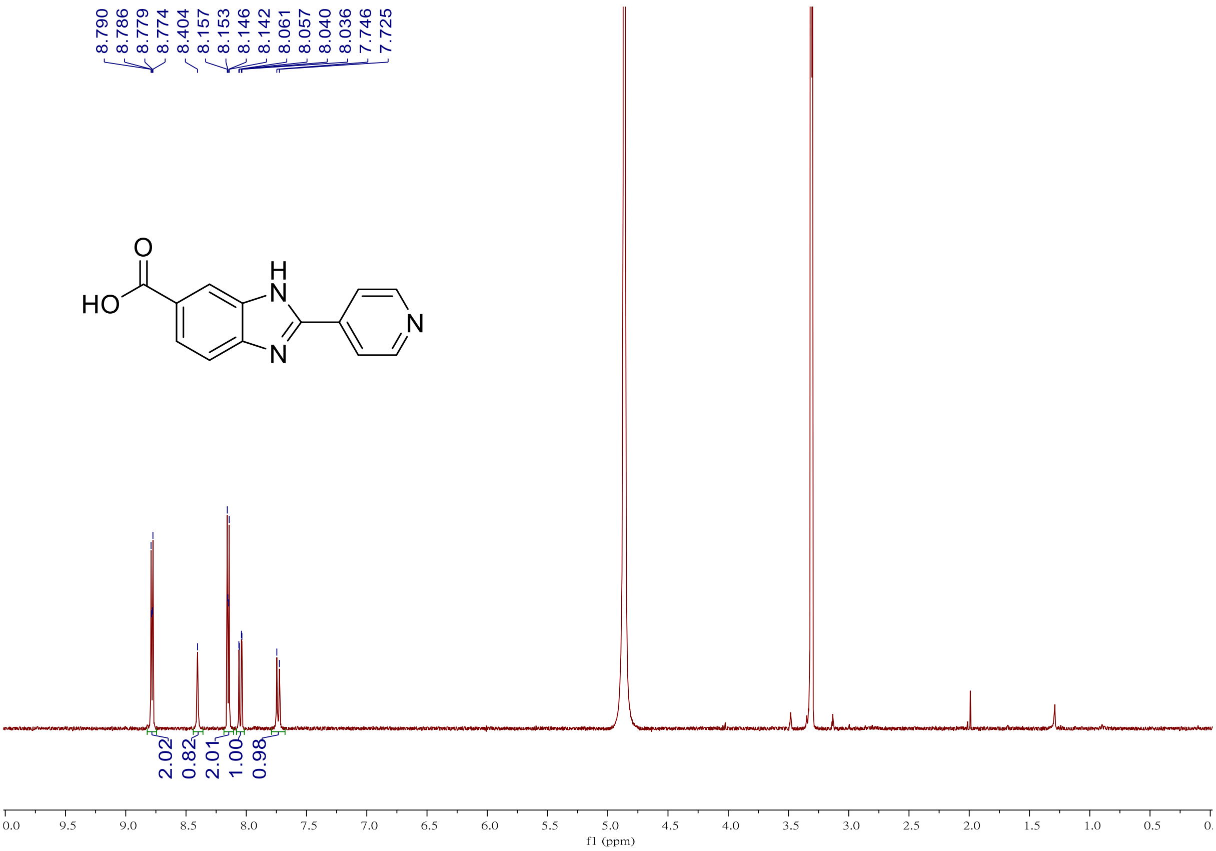


**Figure S71.** $^{1}$H NMR spectrum of compound **1ai** (400 MHz, $CD_3OD$).

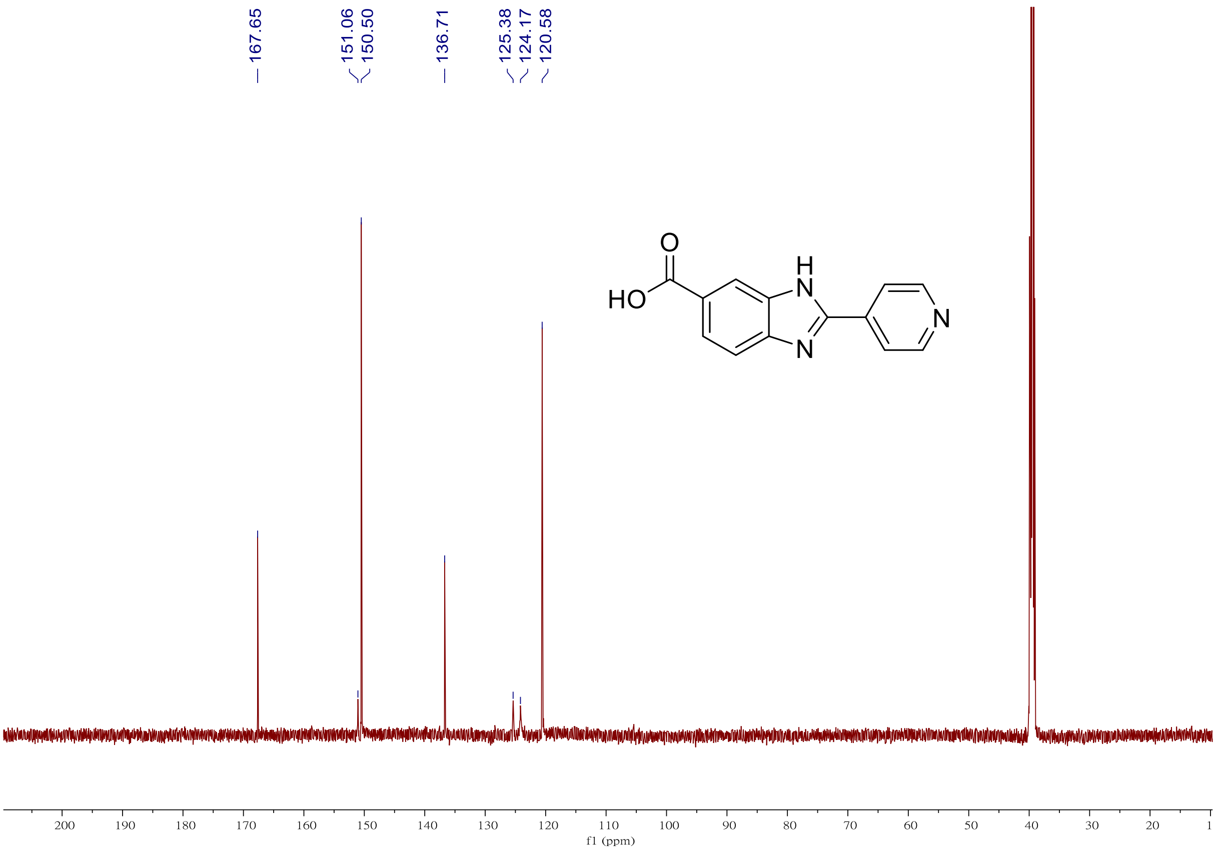


**Figure S72.** $^{13}C\{^{1}H\}$ NMR spectrum of compound **1ai** (151 MHz, $(CD_3)_2SO$).

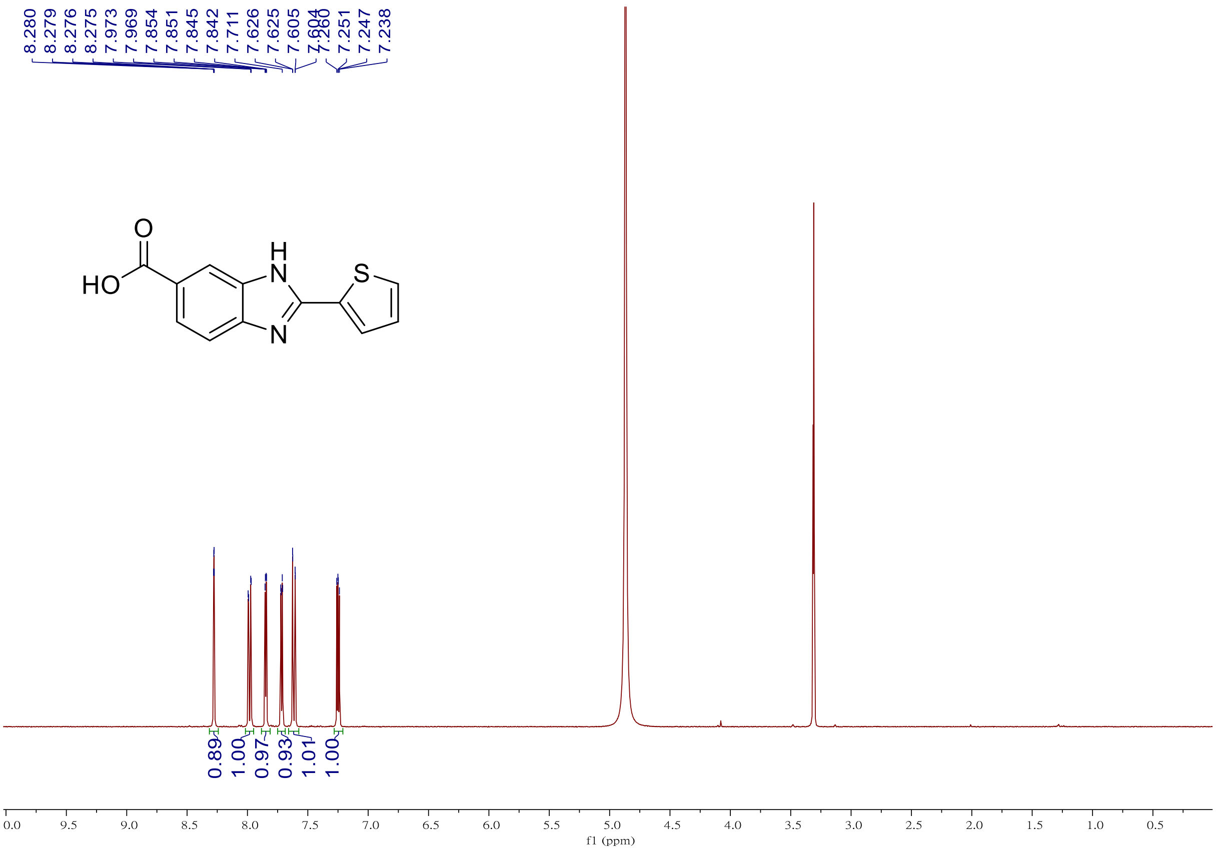


**Figure S73.** $^{1}$H NMR spectrum of compound **1aj** (400 MHz, $CD_3OD$).

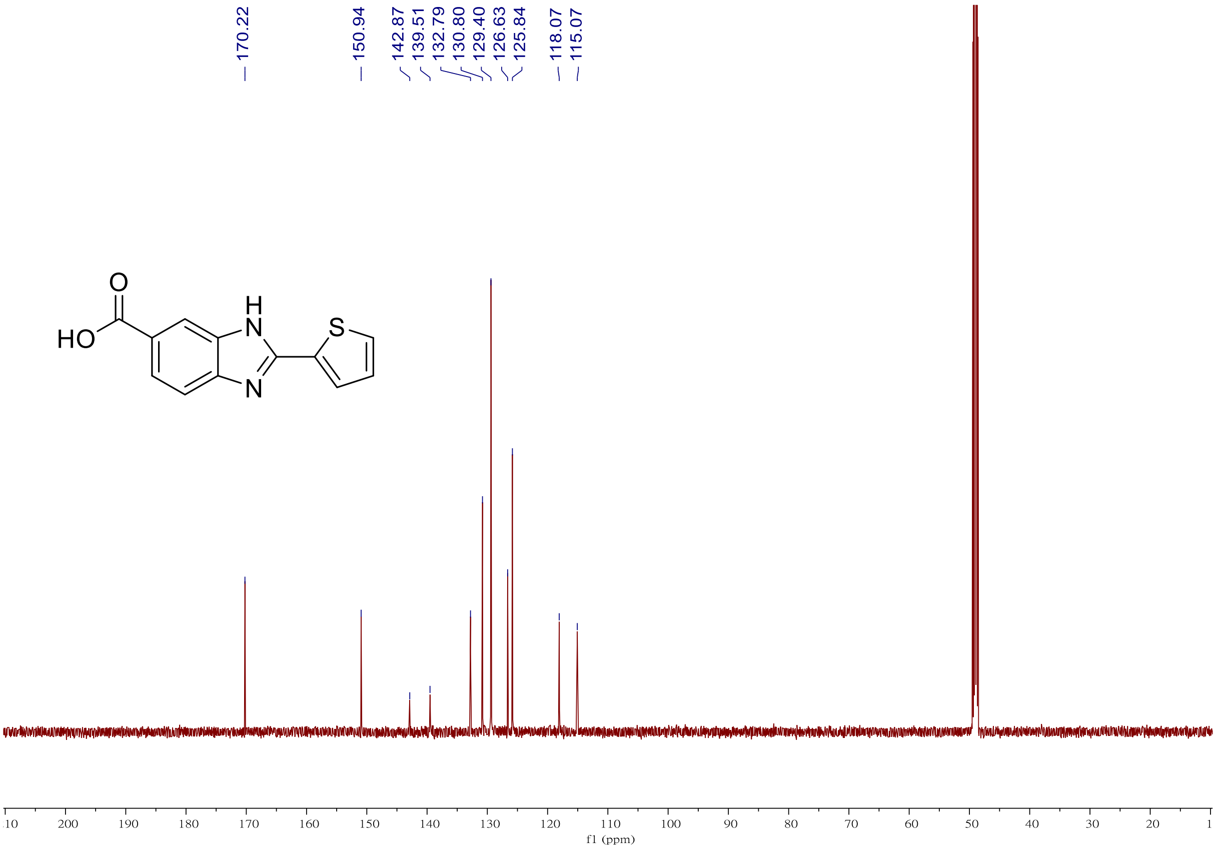


**Figure S74.** $^{13}C\{^{1}H\}$ NMR spectrum of compound **1aj** (151 MHz, $CD_3OD$).

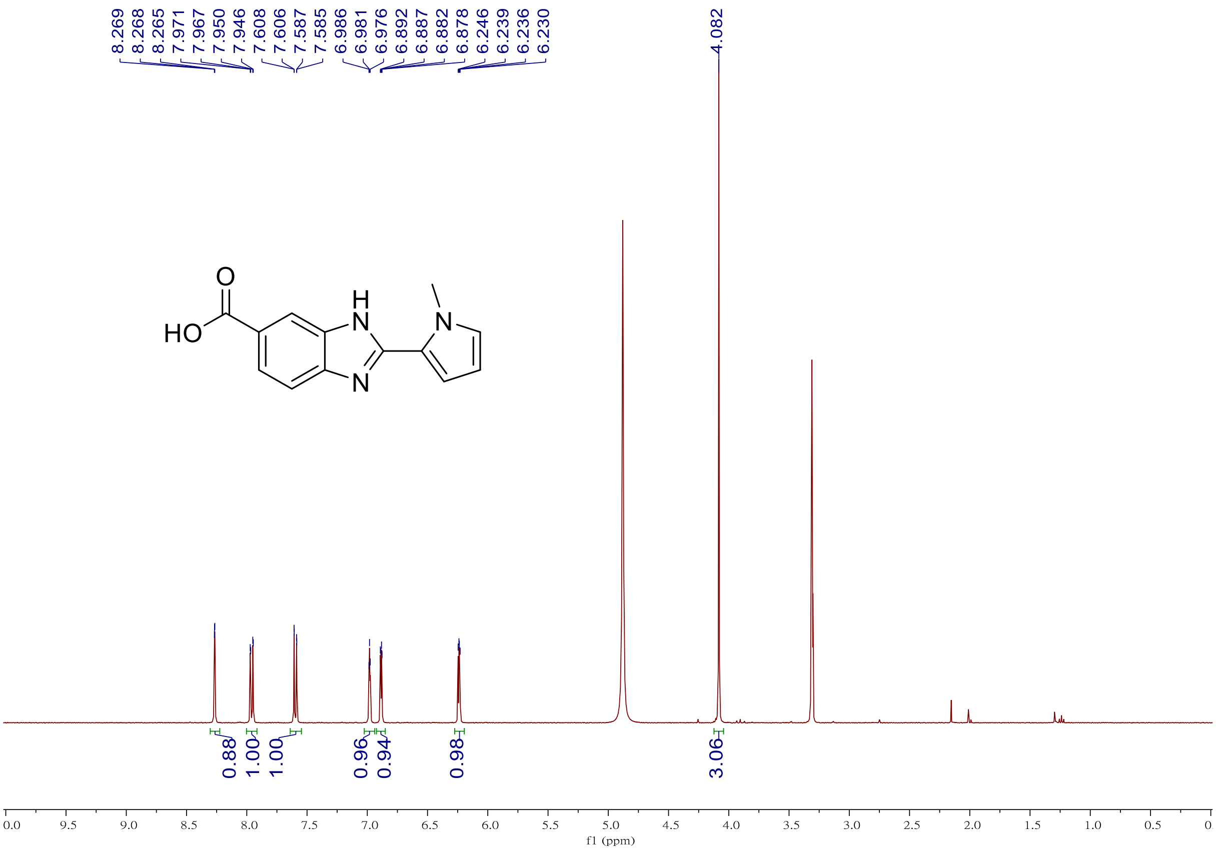


**Figure S75.** $^{1}H$ NMR spectrum of compound **1ak** (400 MHz, $CD_3OD$).

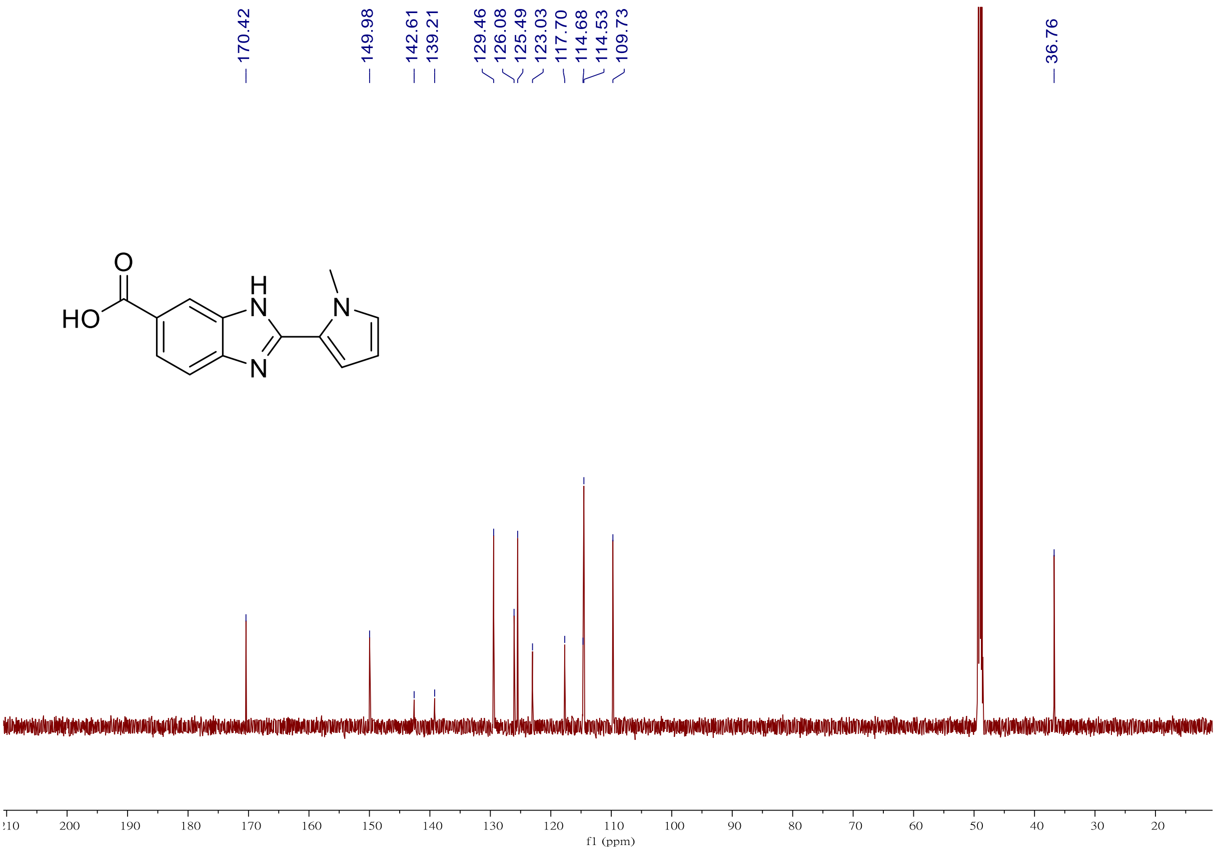


**Figure S76.** $^{13}C\{^{1}H\}$ NMR spectrum of compound **1ak** (151 MHz, $CD_3OD$).

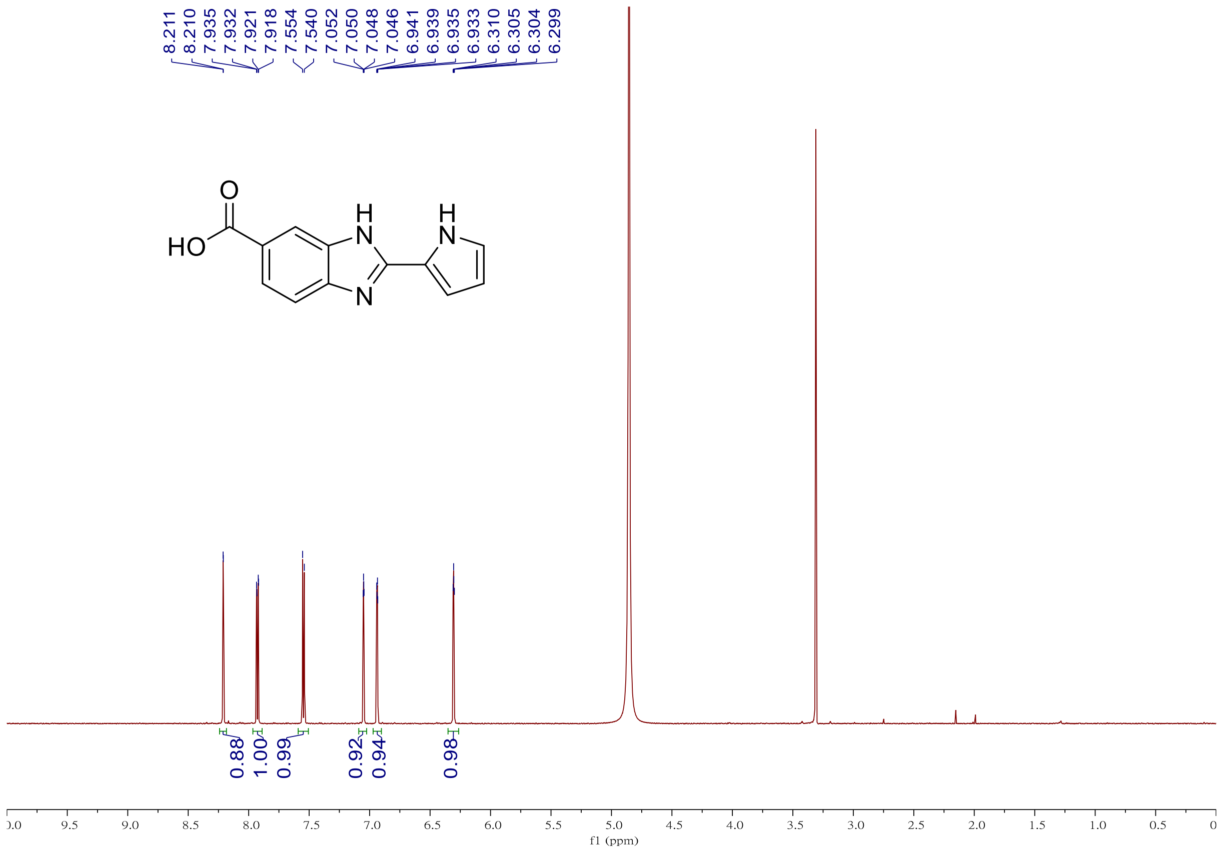


**Figure S77.** $^{1}$H NMR spectrum of compound **1al** (600 MHz, $CD_3OD$).

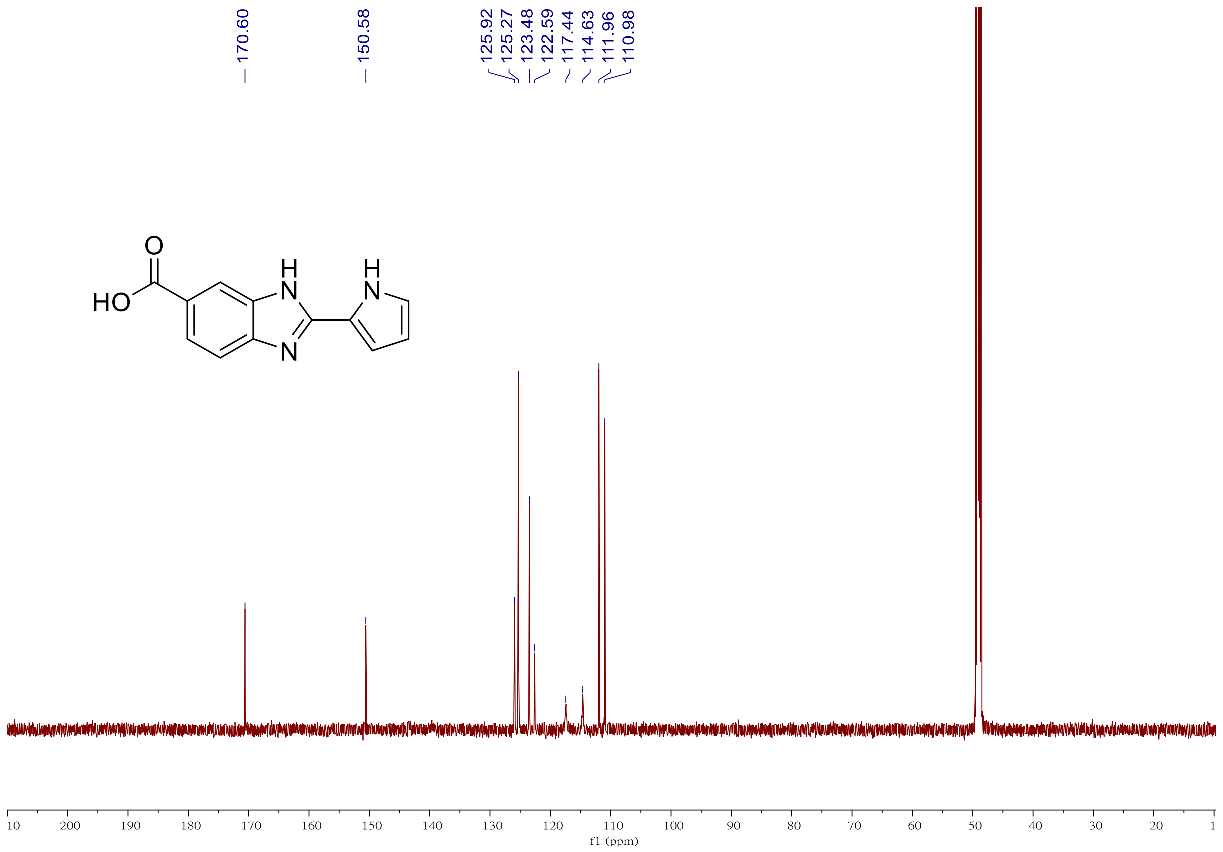


**Figure S78.** $^{13}C\{^{1}H\}$ NMR spectrum of compound **1al** (151 MHz, $CD_3OD$).

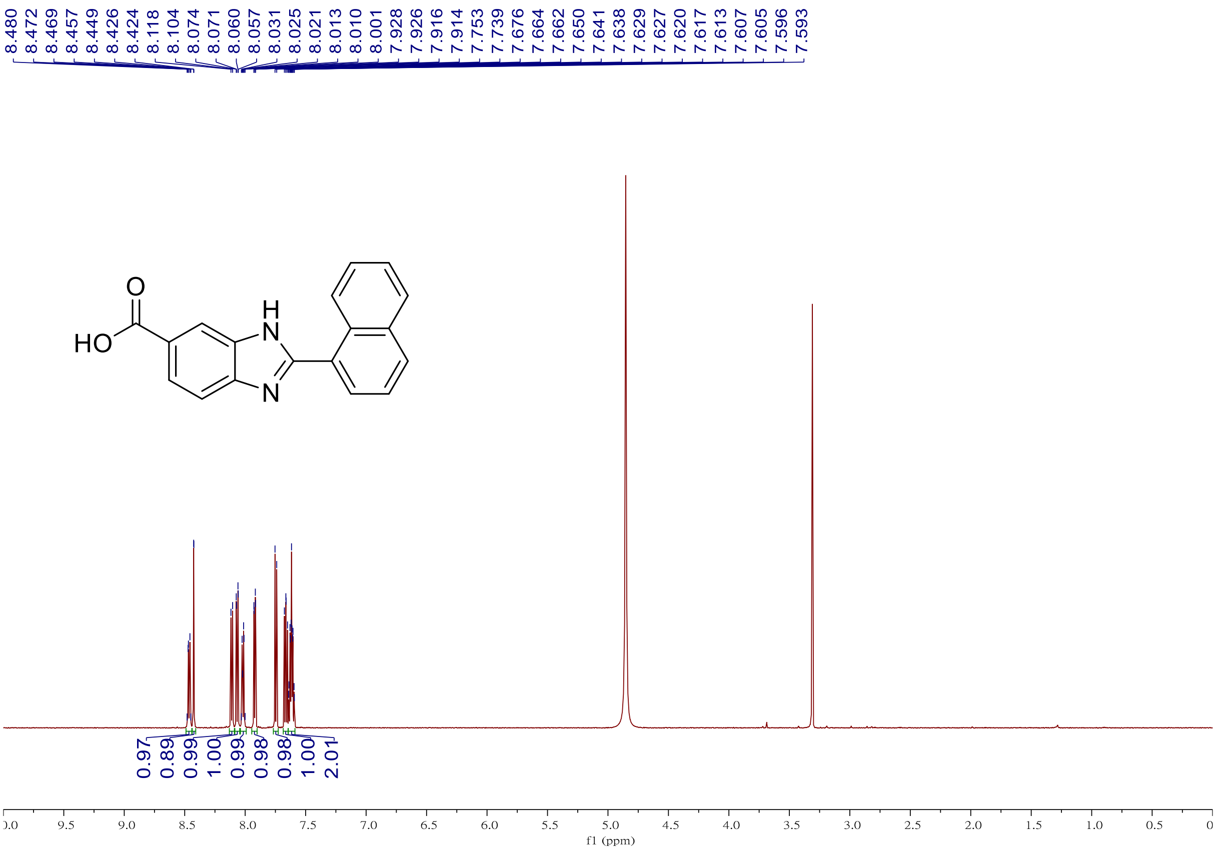


**Figure S79.** $^{1}H$ NMR spectrum of compound **1am** (600 MHz, $CD_3OD$).

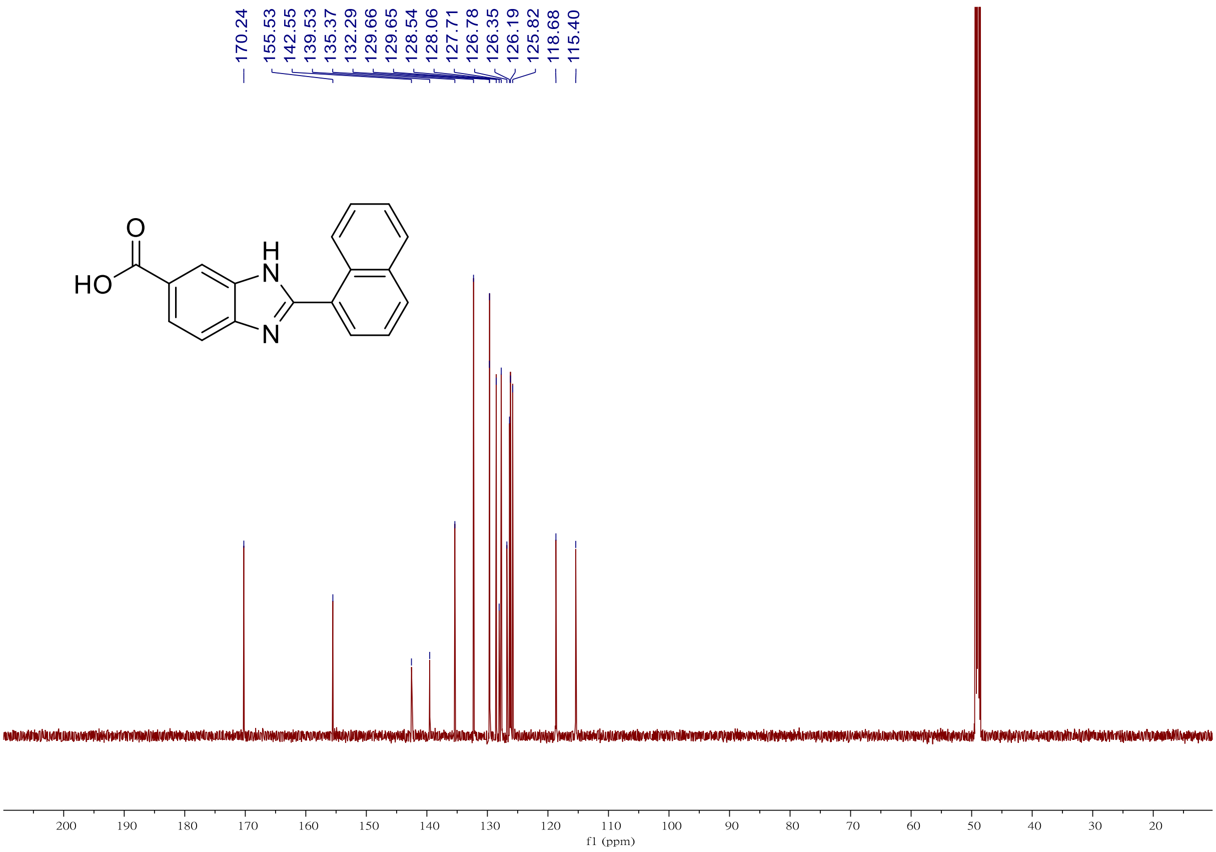


**Figure S80.** $^{13}C\{^{1}H\}$ NMR spectrum of compound **1am** (151 MHz, $CD_3OD$).

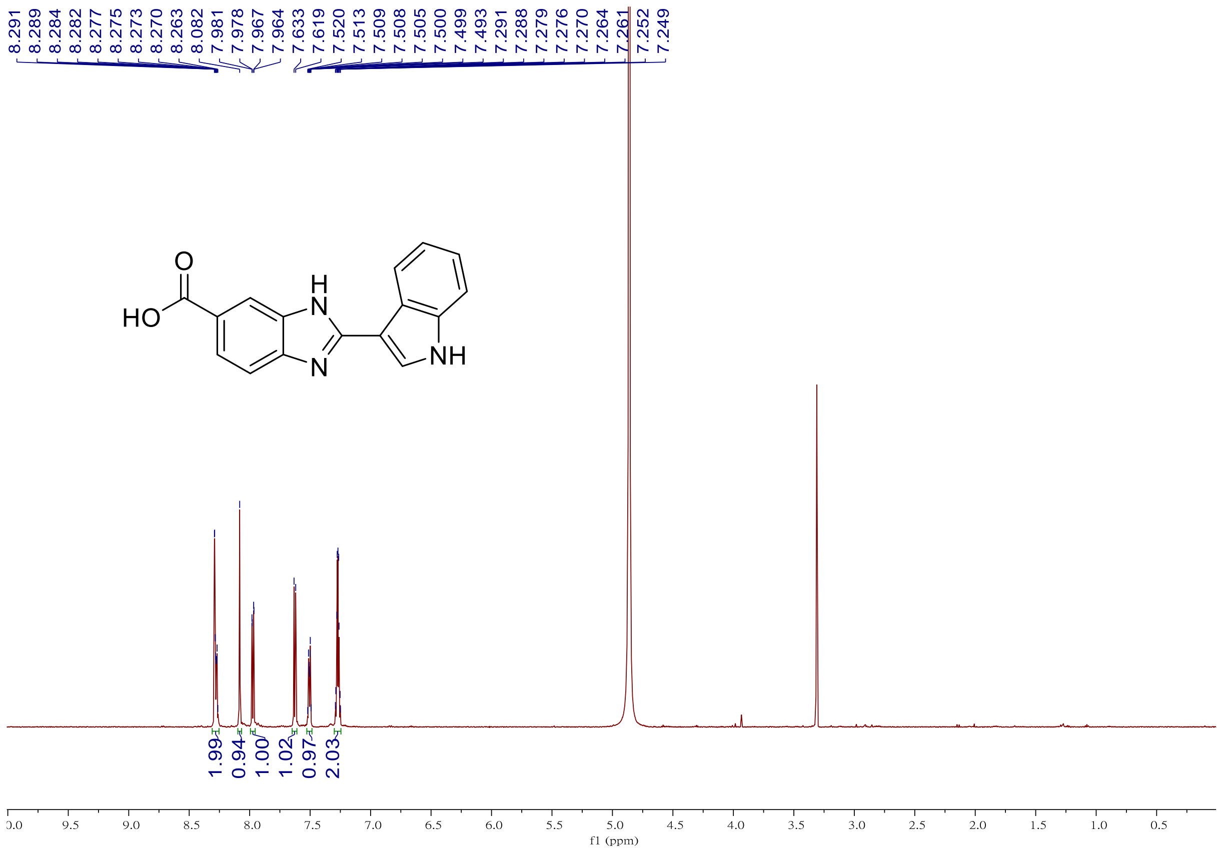


**Figure S81.** $^{1}H$ NMR spectrum of compound **1an** (600 MHz, $CD_3OD$).

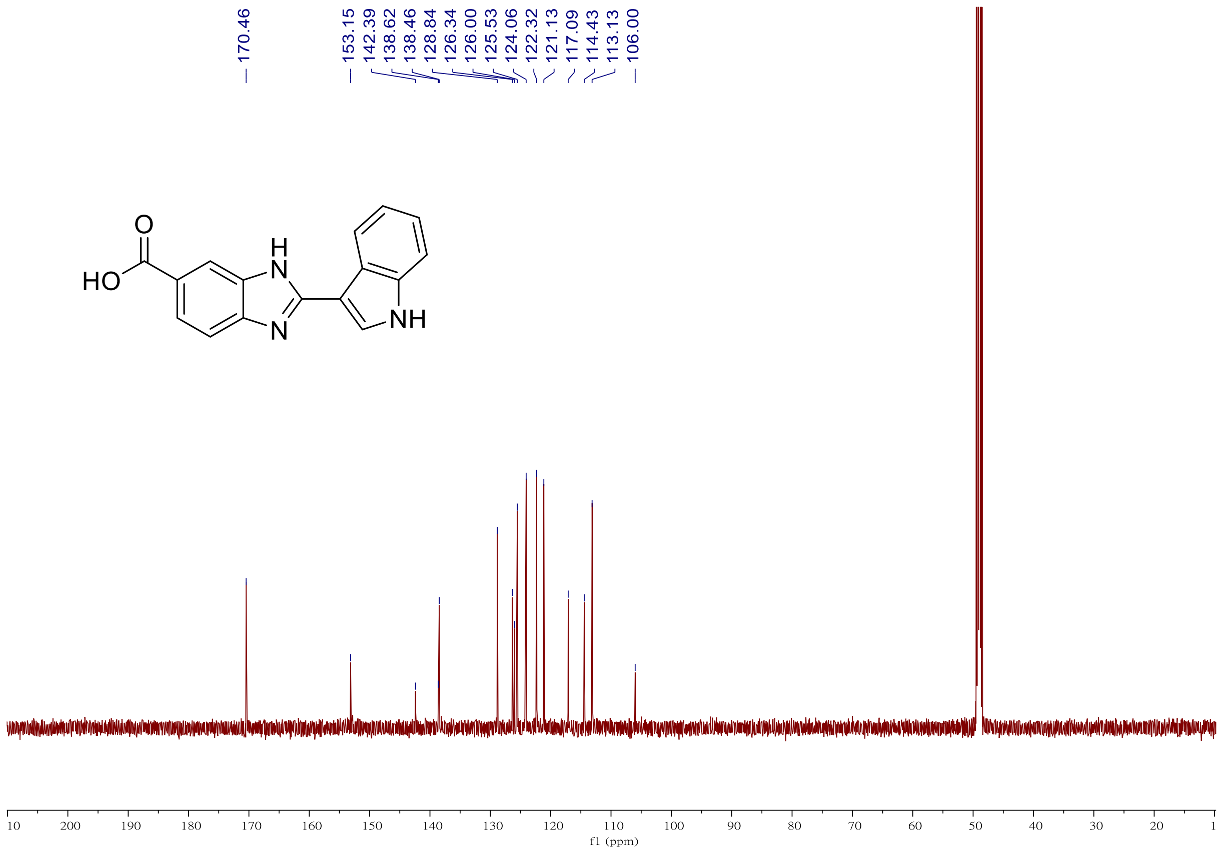


**Figure S82.** $^{13}C\{^{1}H\}$ NMR spectrum of compound **1an** (151 MHz, $CD_3OD$).

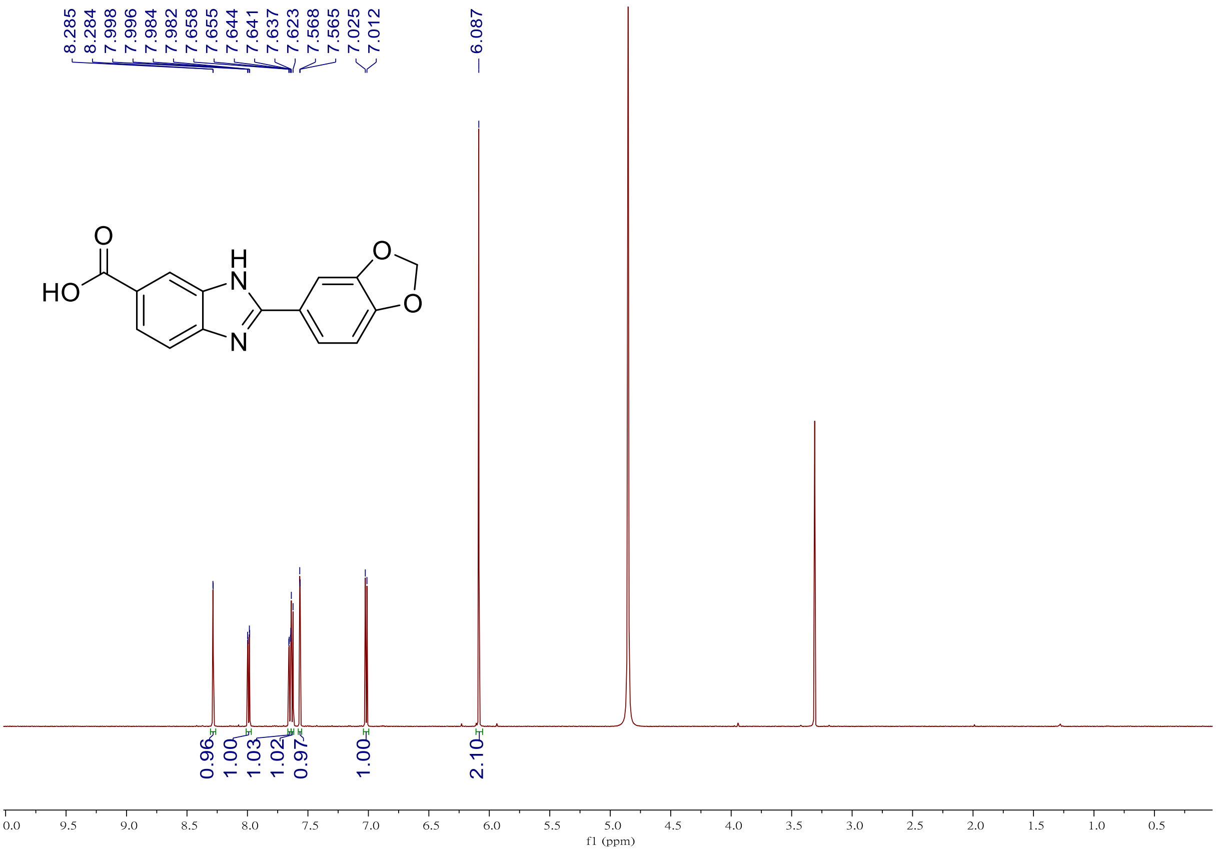


**Figure S83.** $^{1}$H NMR spectrum of compound **1ao** (600 MHz, $CD_3OD$).

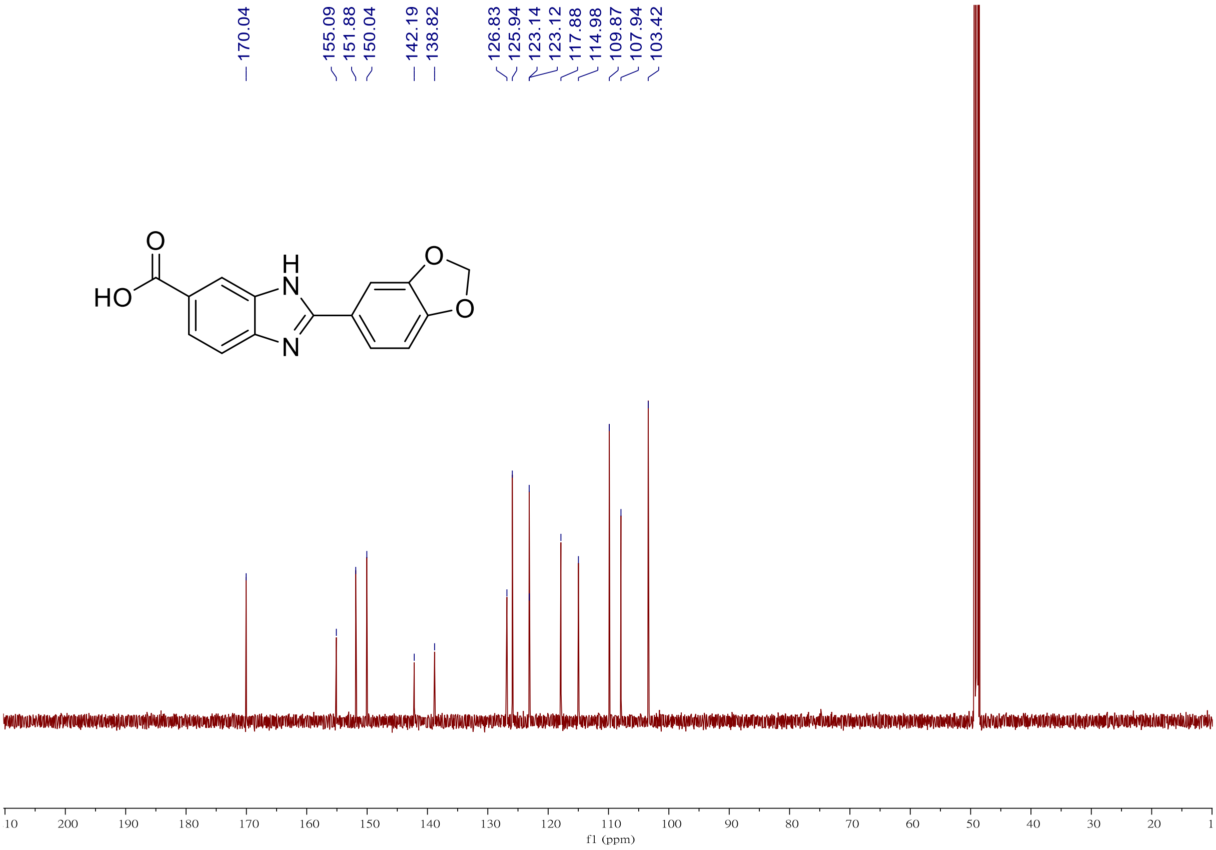


**Figure S84.** $^{13}C\{^{1}H\}$ NMR spectrum of compound **1ao** (151 MHz, $CD_3OD$).

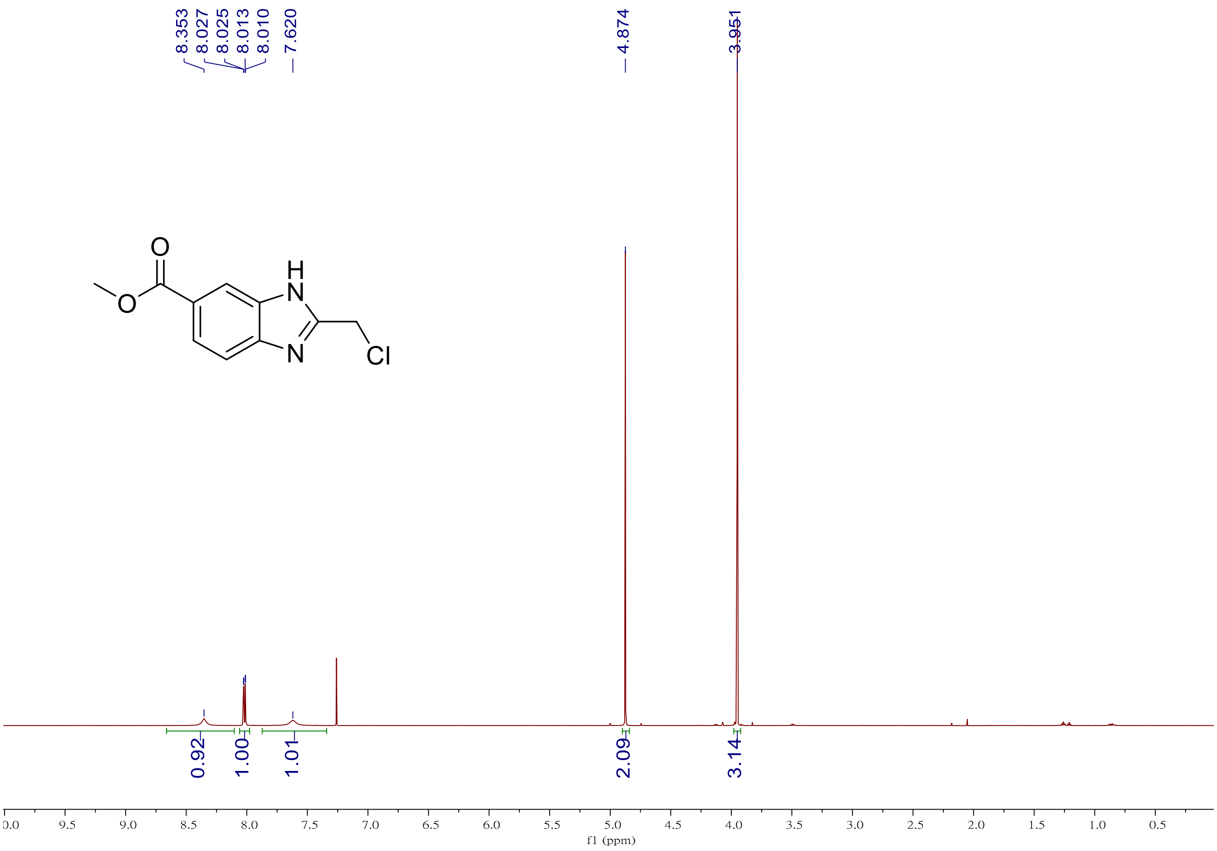


**Figure S85.** $^{1}$H NMR spectrum of compound **1ar*** (600 MHz, $CDCl_3$).

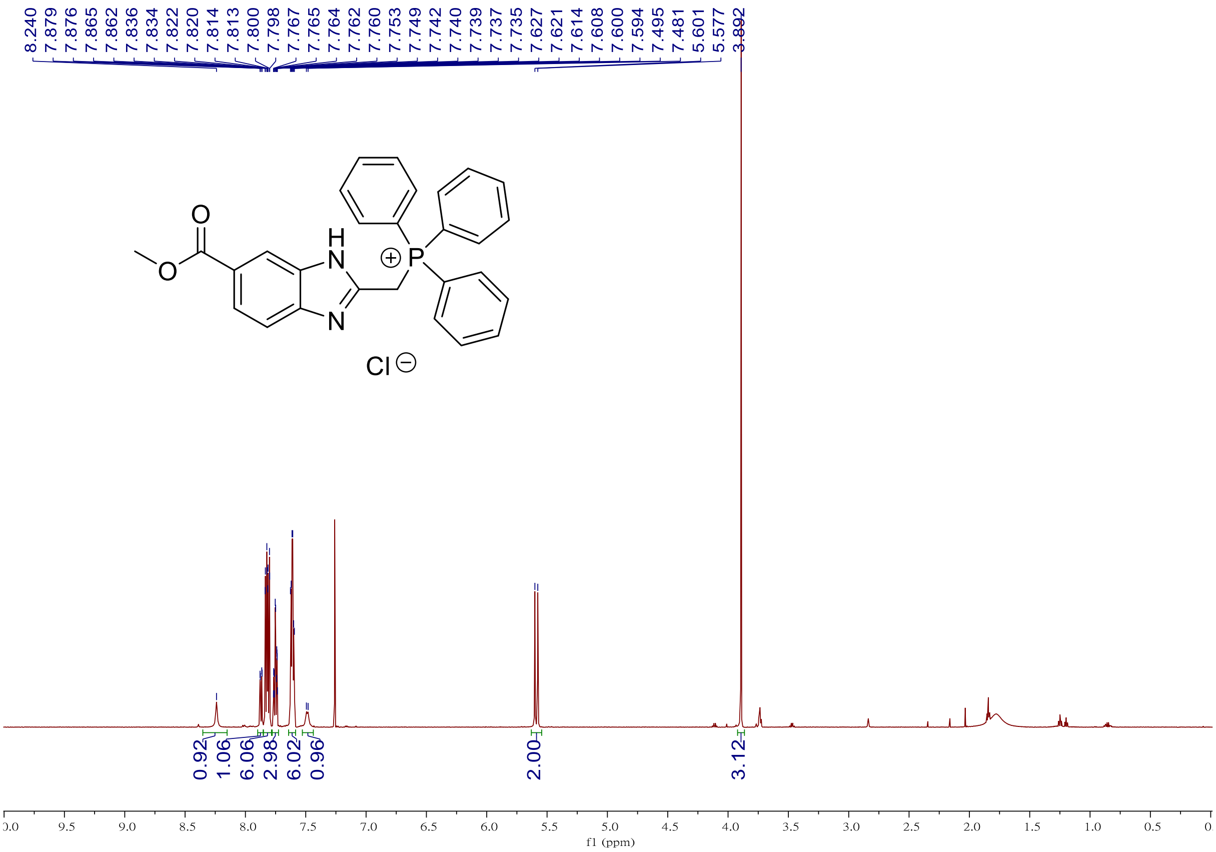


**Figure S86.** $^{1}$H NMR spectrum of compound **1as*** (600 MHz, $CDCl_3$).

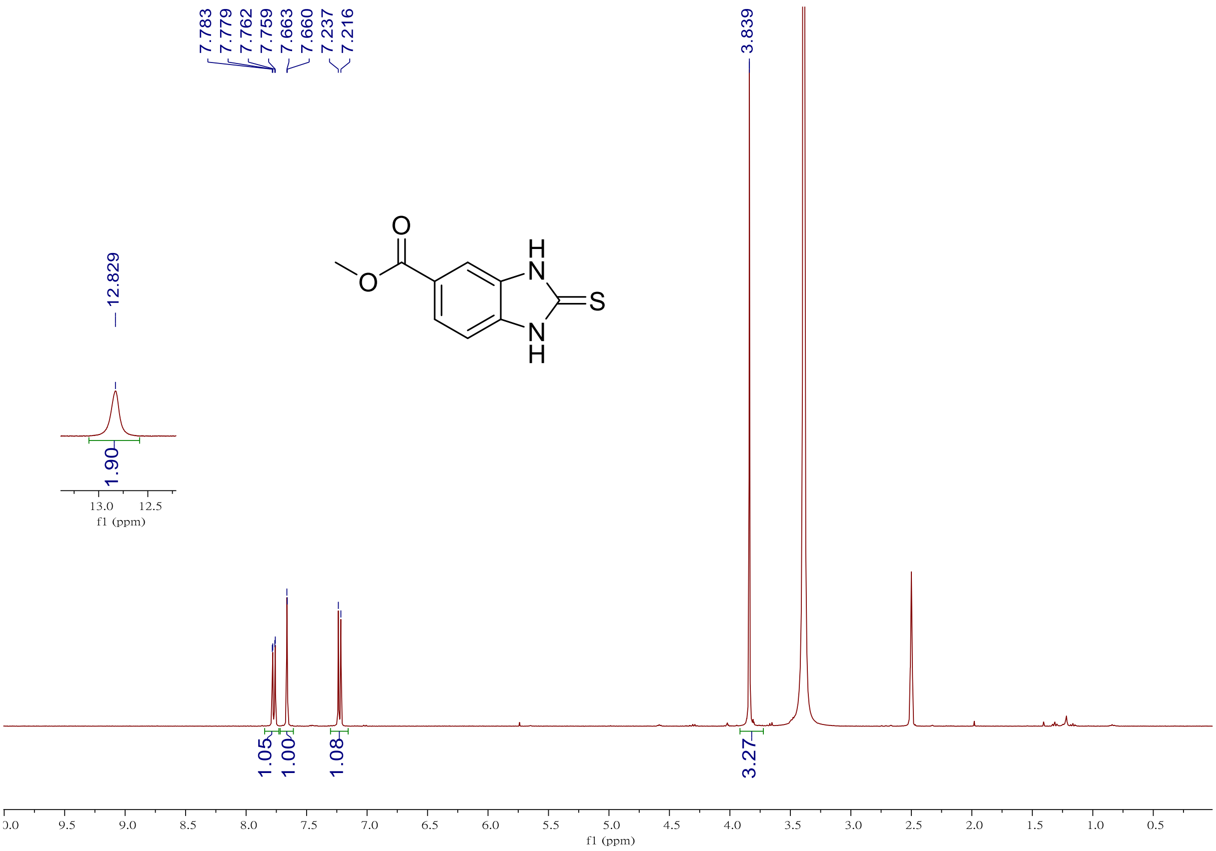


**Figure S87.** $^{1}$H NMR spectrum of compound **1at*** (400 MHz, $(CD_3)_2SO$).

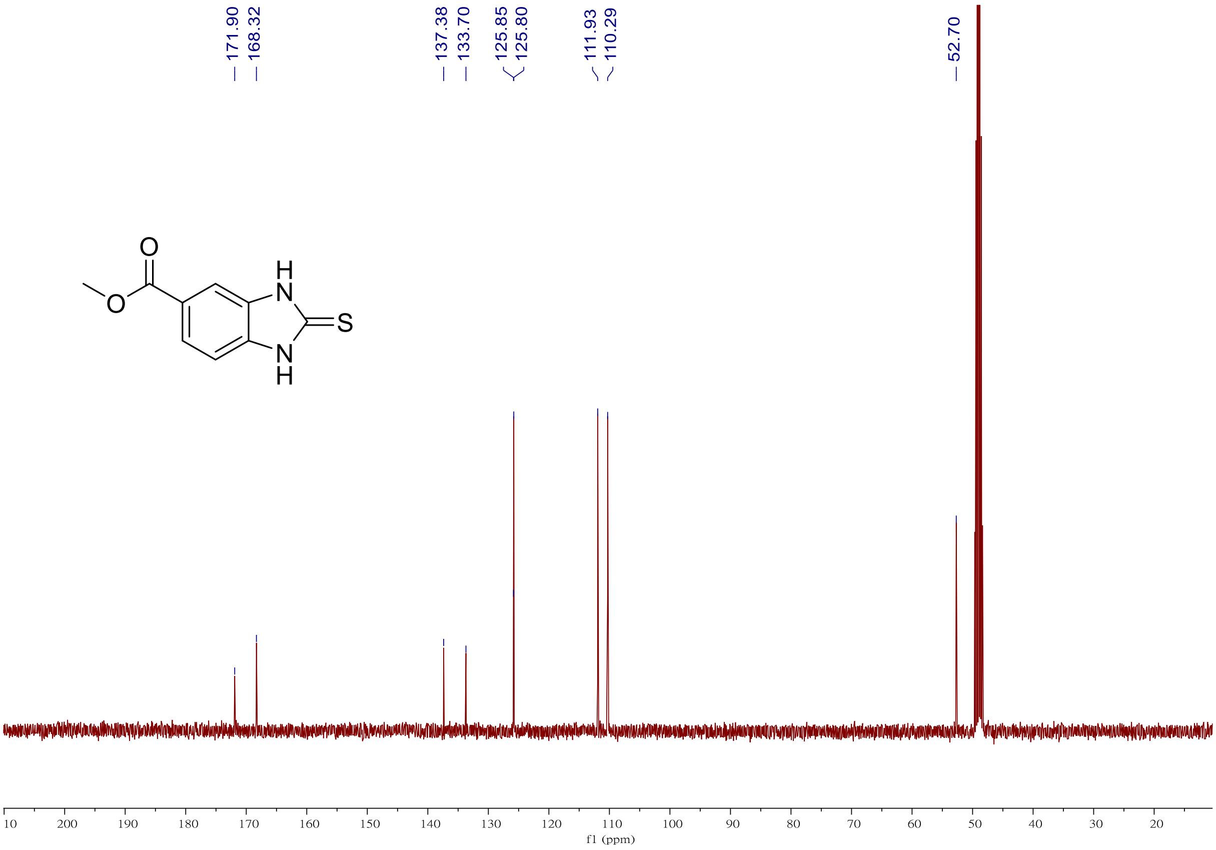


**Figure S88.** $^{13}C\{^{1}H\}$ NMR spectrum of compound **1at*** (101 MHz, $CD_3OD$).

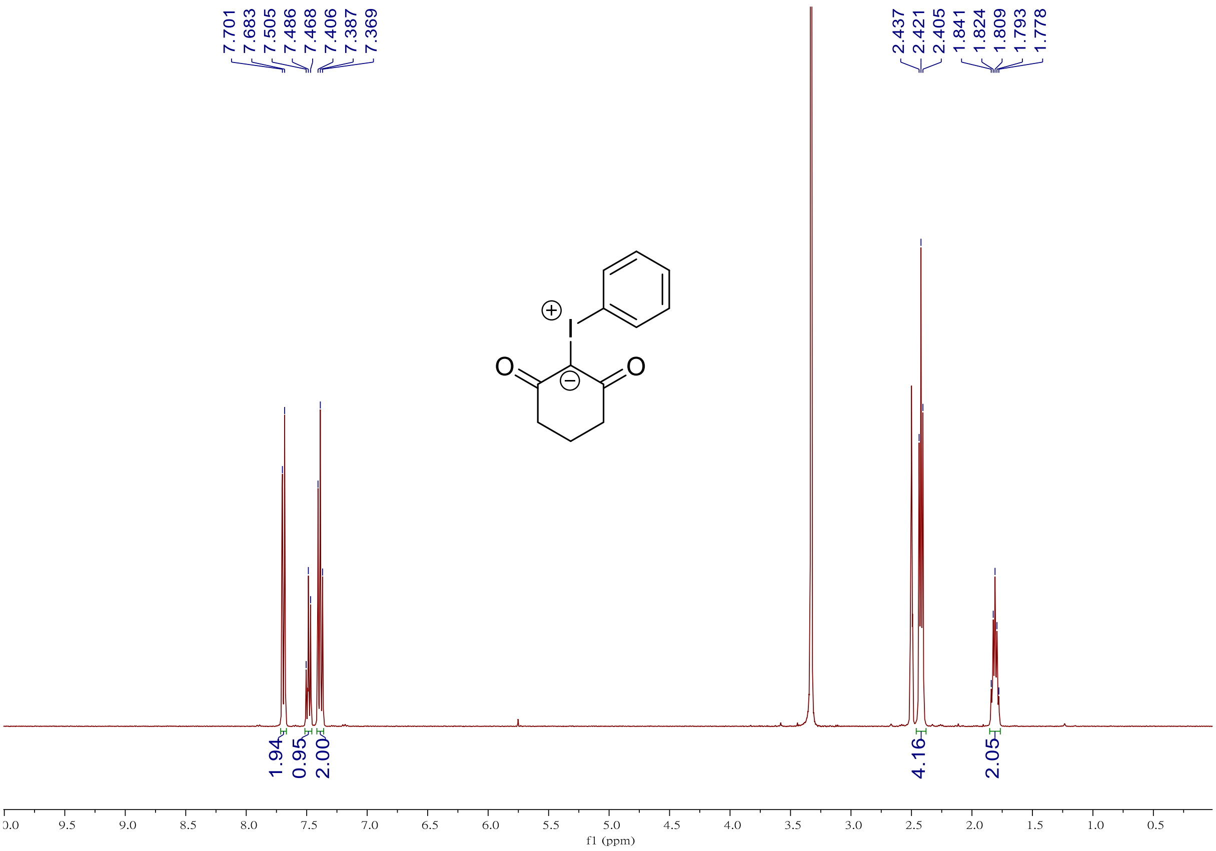


**Figure S89.** $^{1}$H NMR spectrum of compound **2a** (400 MHz, $(CD_3)_2SO$).

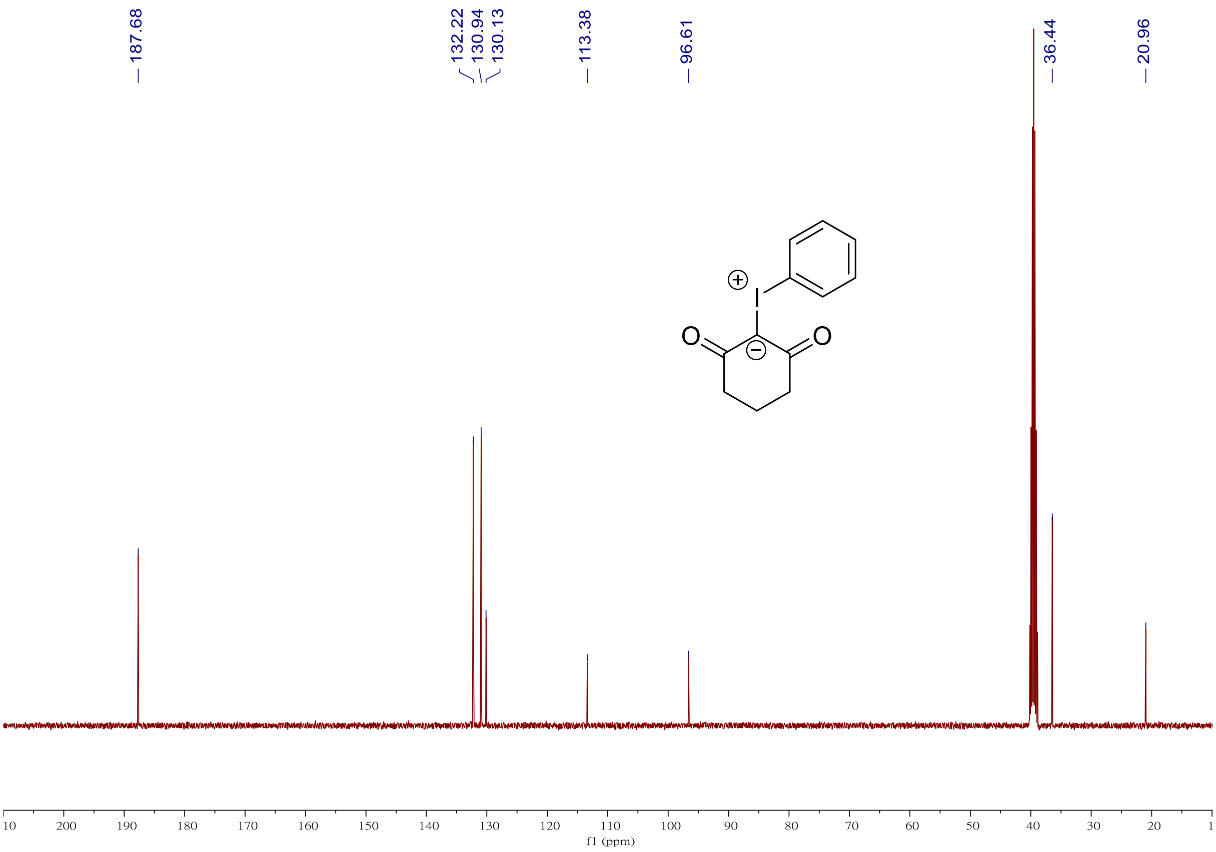


**Figure S90.** $^{13}C\{^{1}H\}$ NMR spectrum of compound **2a** (101 MHz, $(CD_3)_2SO$).

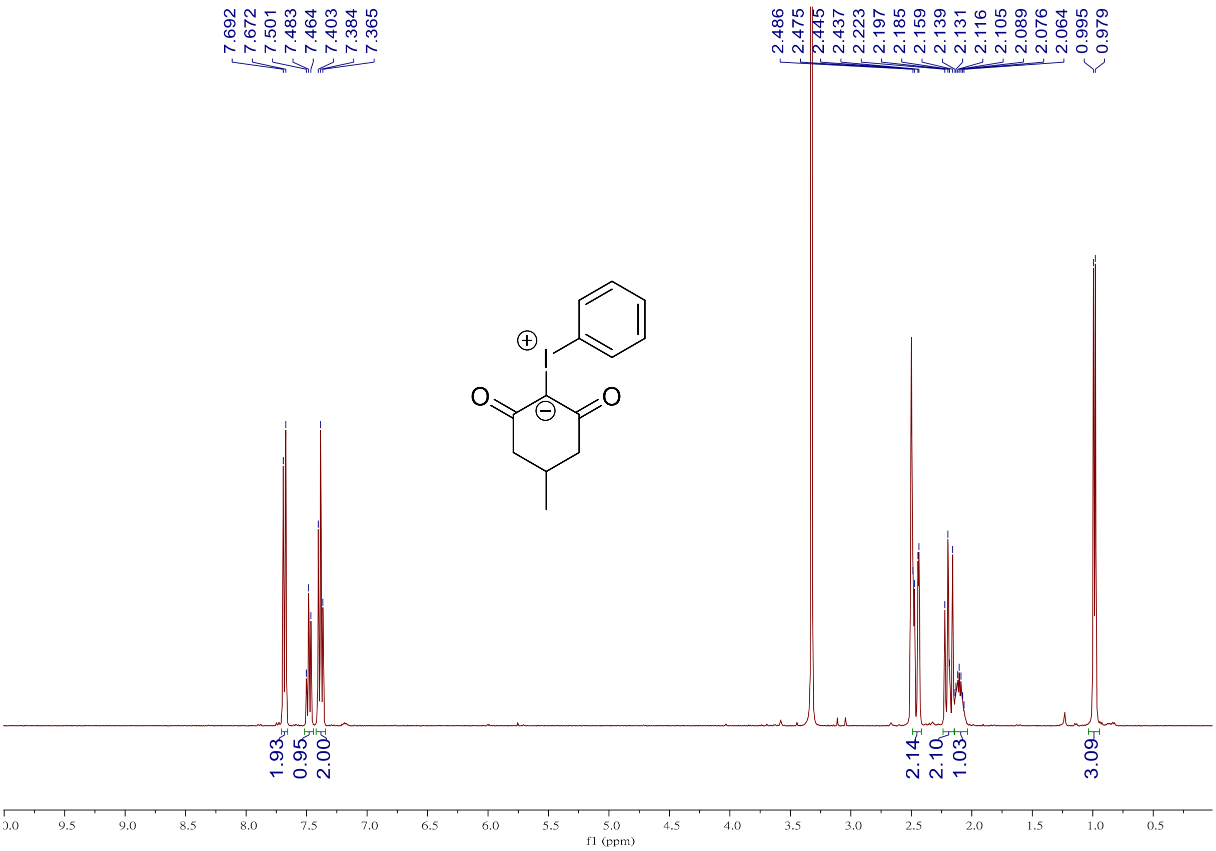


**Figure S91.** $^{1}$H NMR spectrum of compound **2b** (400 MHz, $(CD_3)_2SO$).

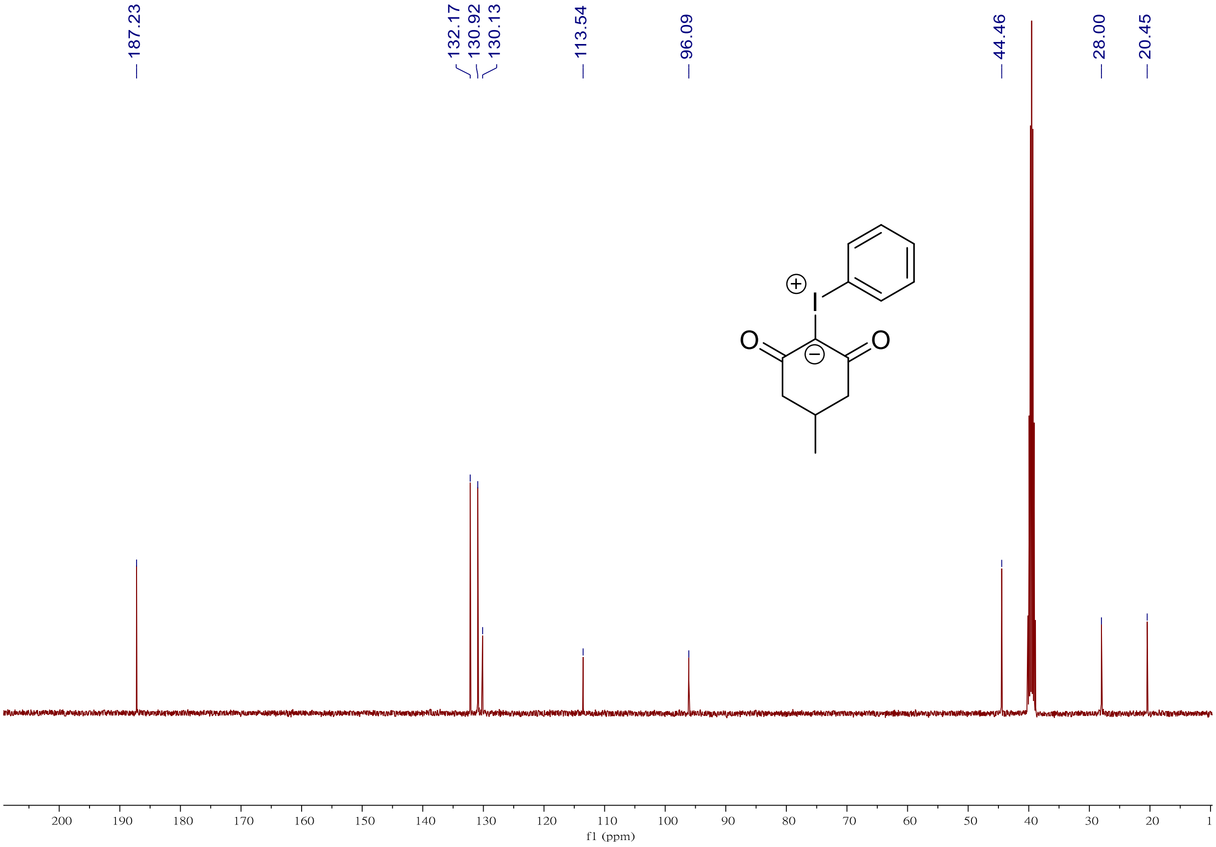


**Figure S92.** $^{13}C\{^1H\}$ NMR spectrum of compound **2b** (101 MHz, $(CD_3)_2SO$).

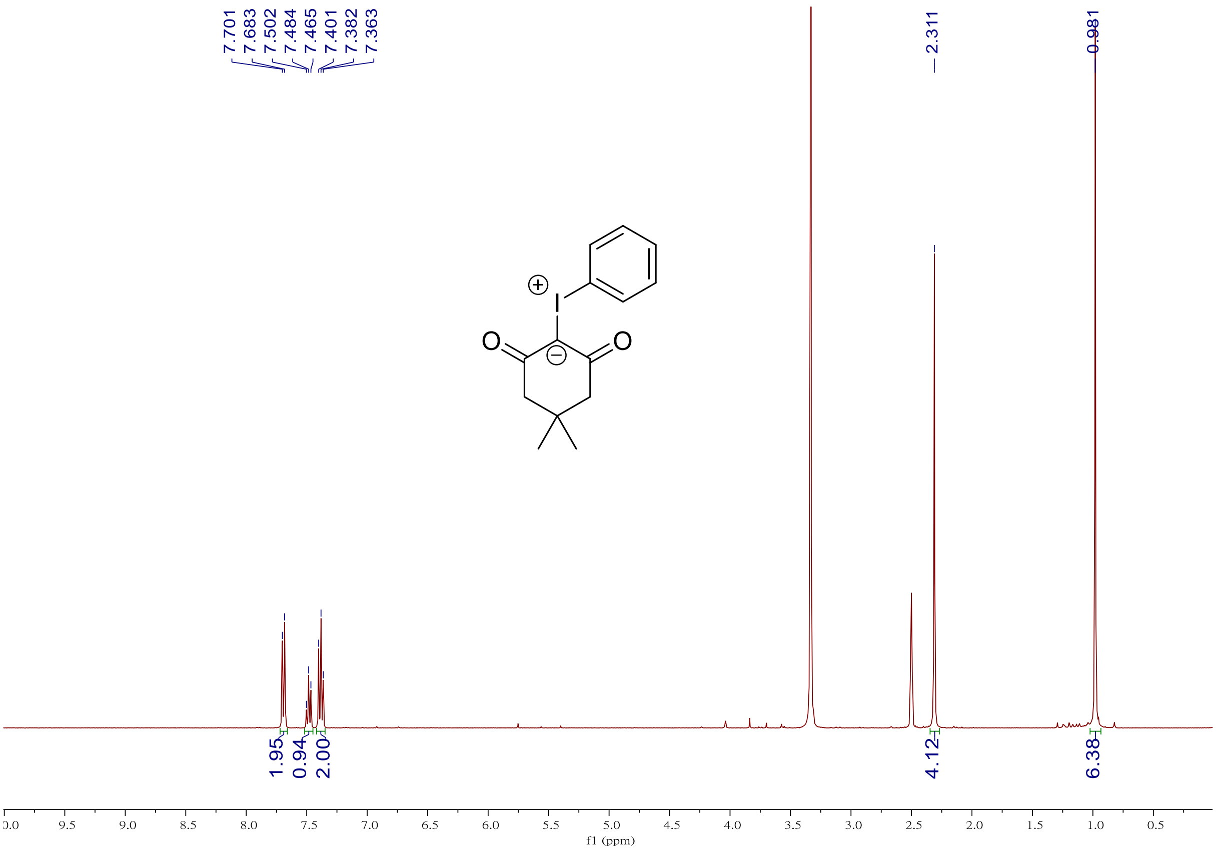


**Figure S93.** $^{1}H$ NMR spectrum of compound **2c** (400 MHz, $(CD_3)_2SO$).

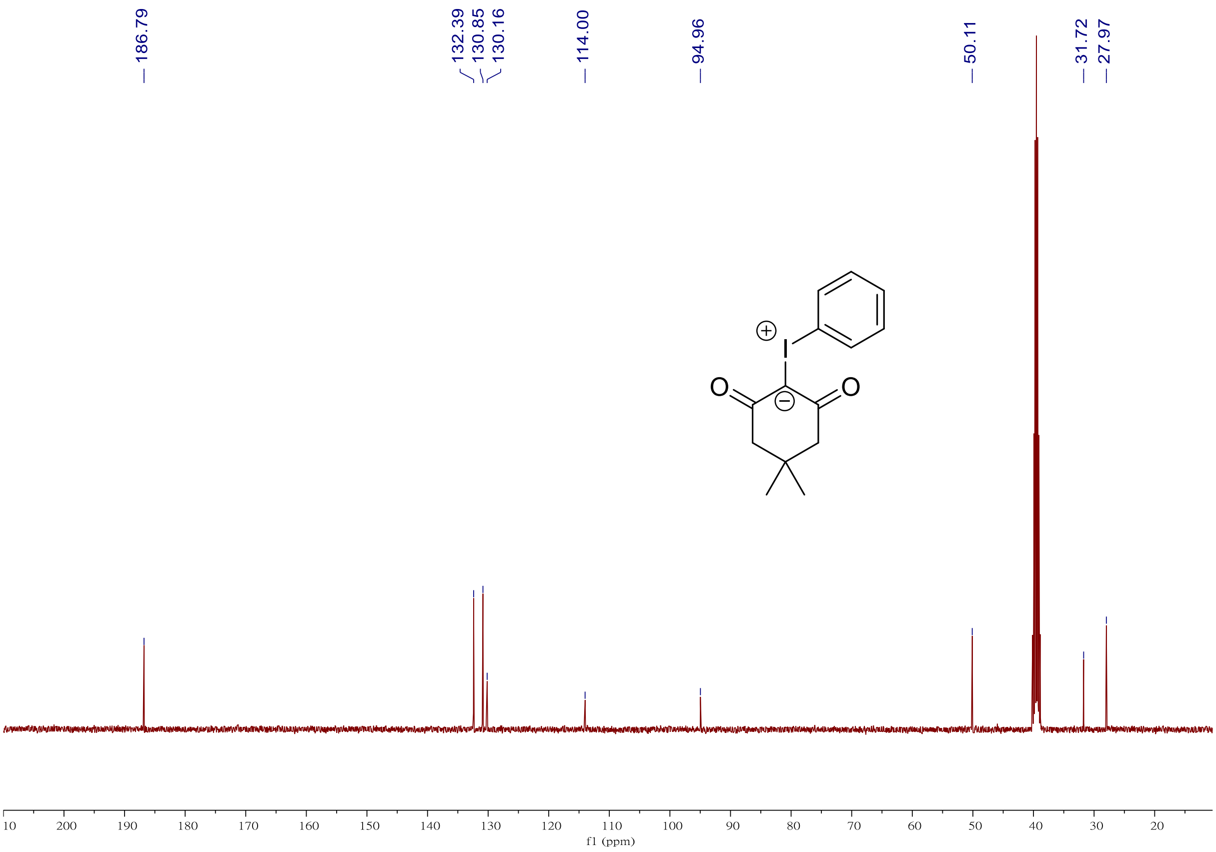


**Figure S94.** $^{13}C\{^{1}H\}$ NMR spectrum of compound **2c** (101 MHz, $(CD_3)_2SO$).

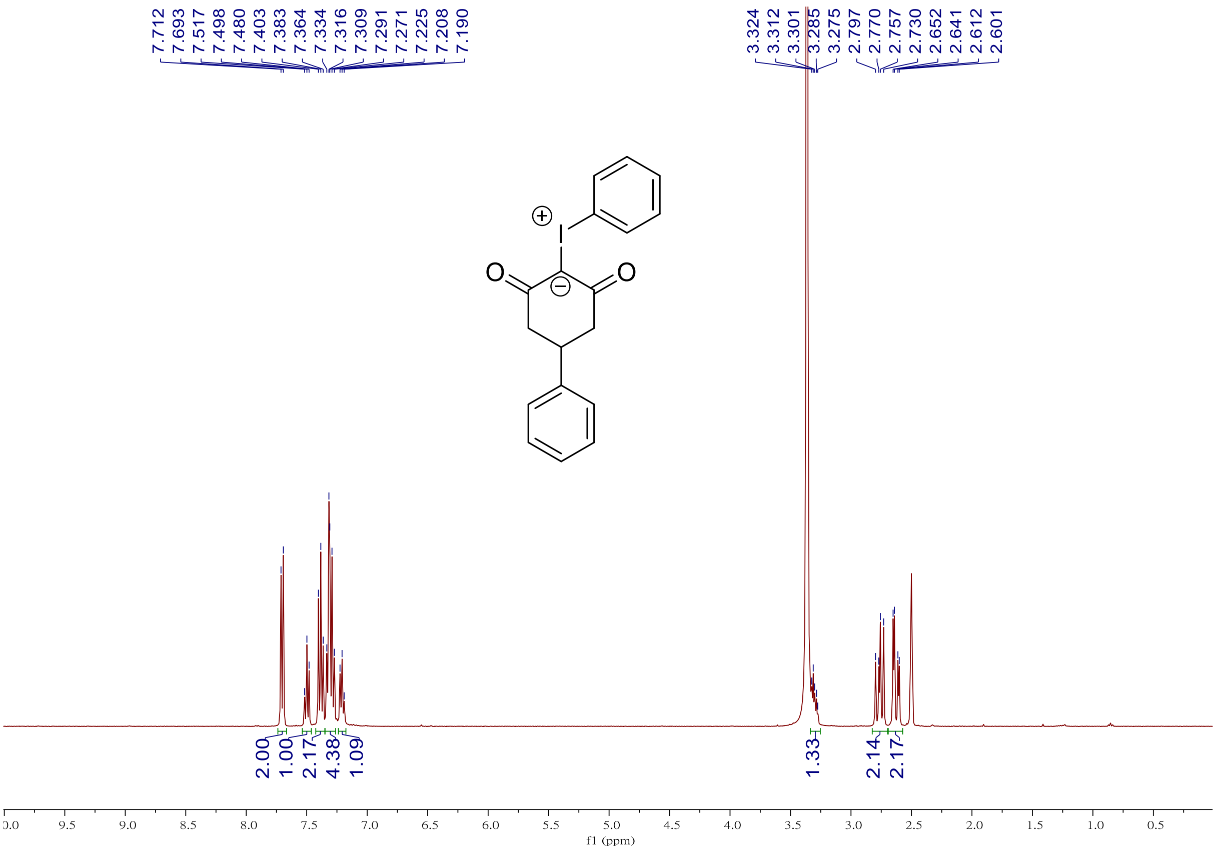


**Figure S95.** $^{1}$H NMR spectrum of compound **2d** (400 MHz, $(CD_3)_2SO$).

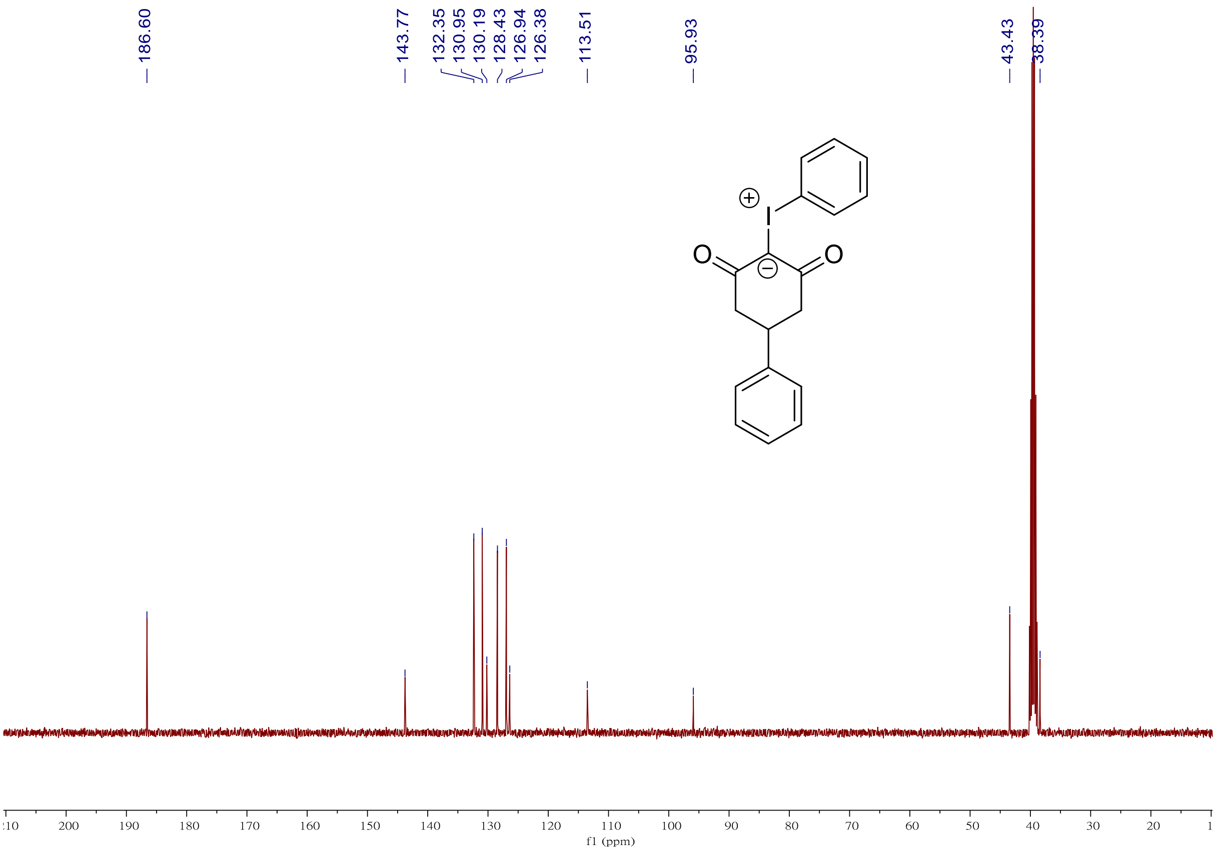


**Figure S96.** $^{13}C\{^{1}H\}$ NMR spectrum of compound **2d** (101 MHz, $(CD_3)_2SO$).

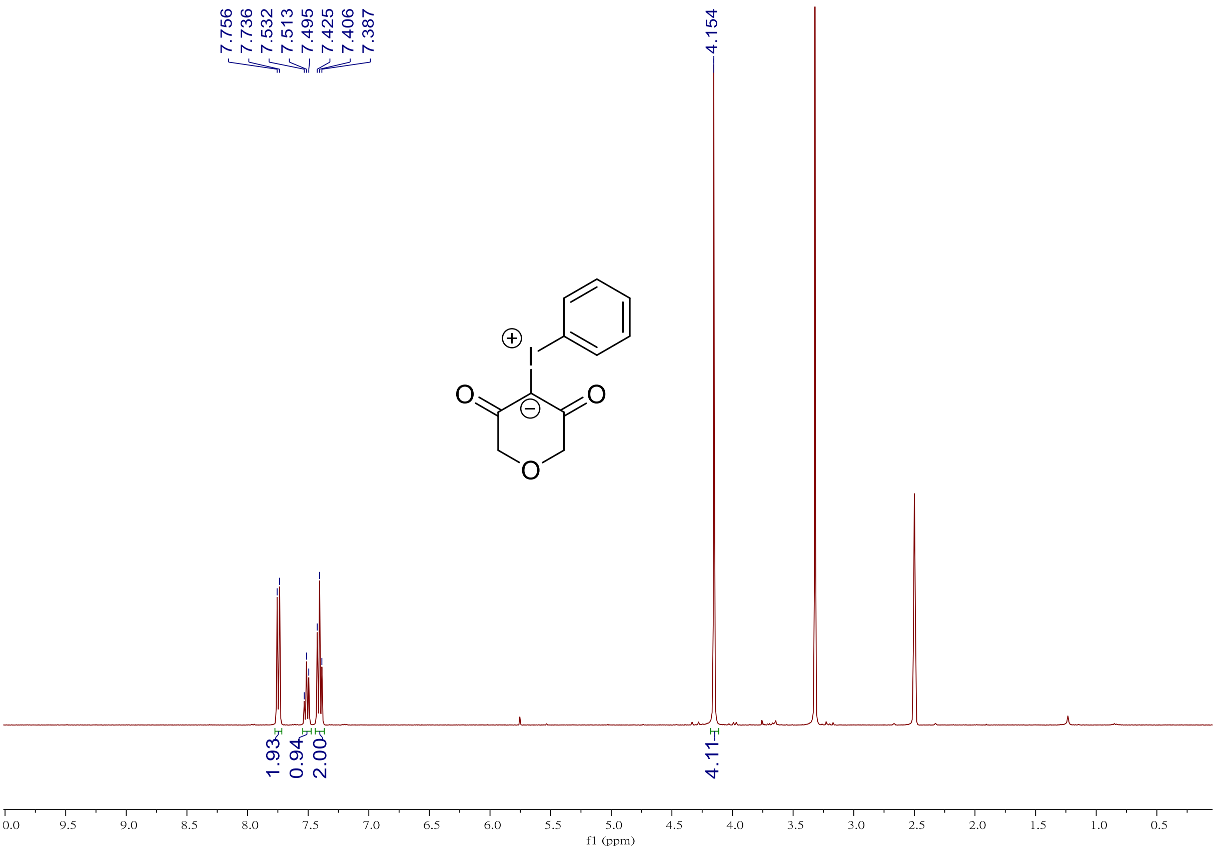


**Figure S97.** $^{1}$H NMR spectrum of compound **2e** (400 MHz, $(CD_3)_2SO$).

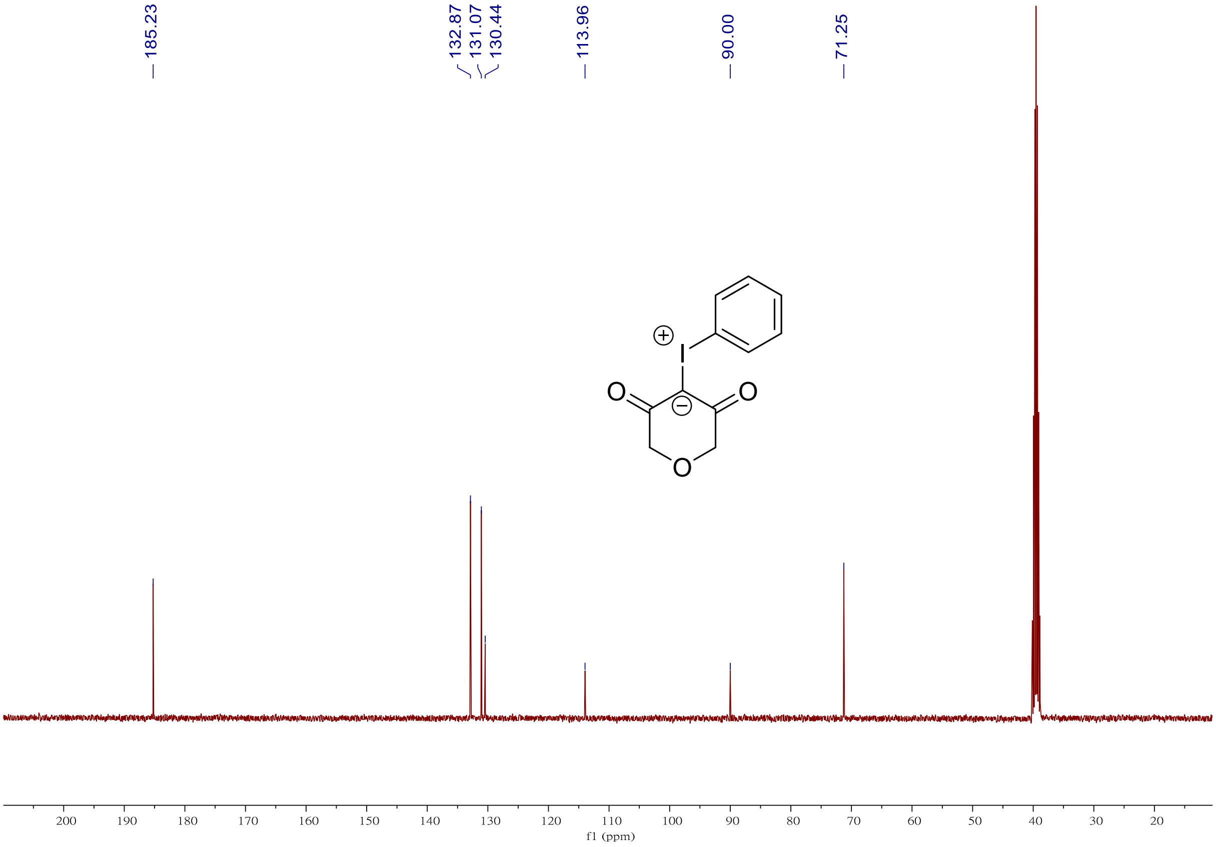


**Figure S98.** $^{13}C\{^{1}H\}$ NMR spectrum of compound **2e** (101 MHz, $(CD_3)_2SO$).

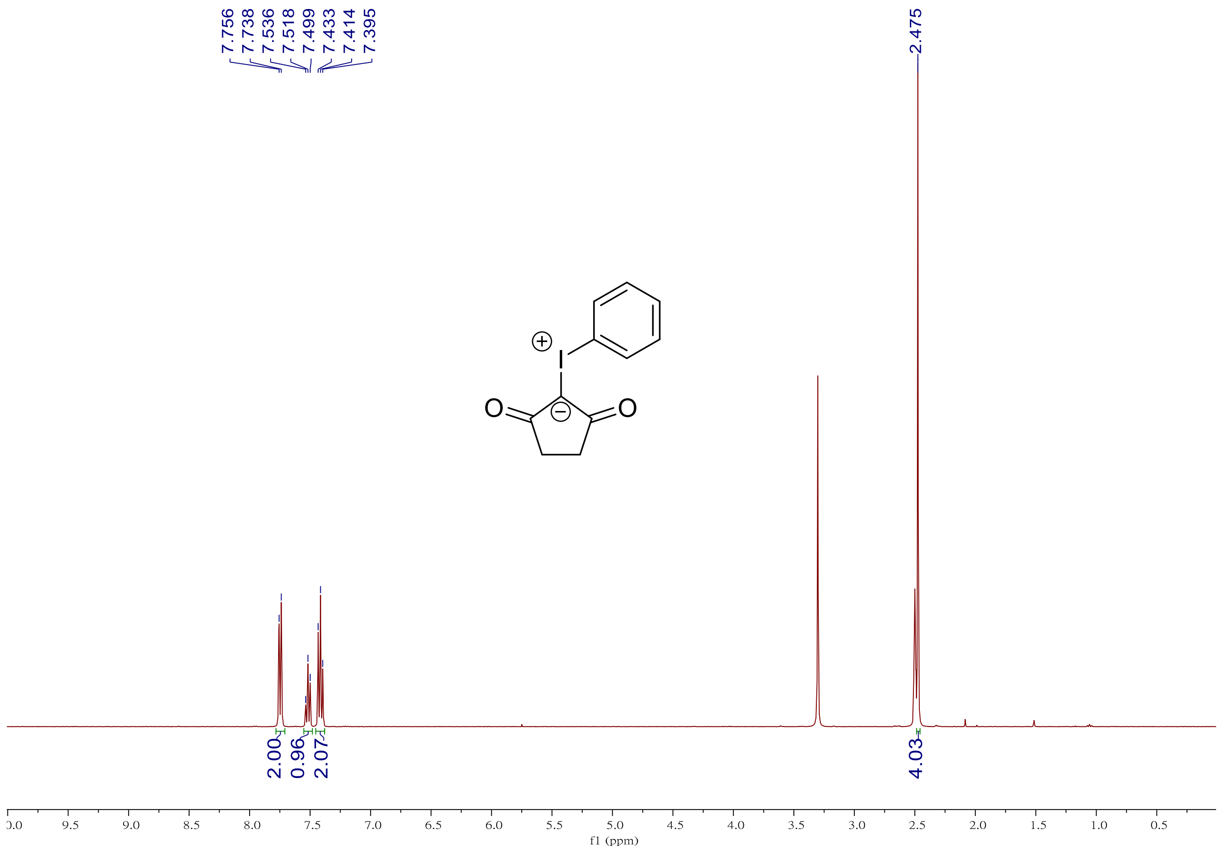


**Figure S99.** $^{1}$H NMR spectrum of compound **2f** (400 MHz, $(CD_3)_2SO$).

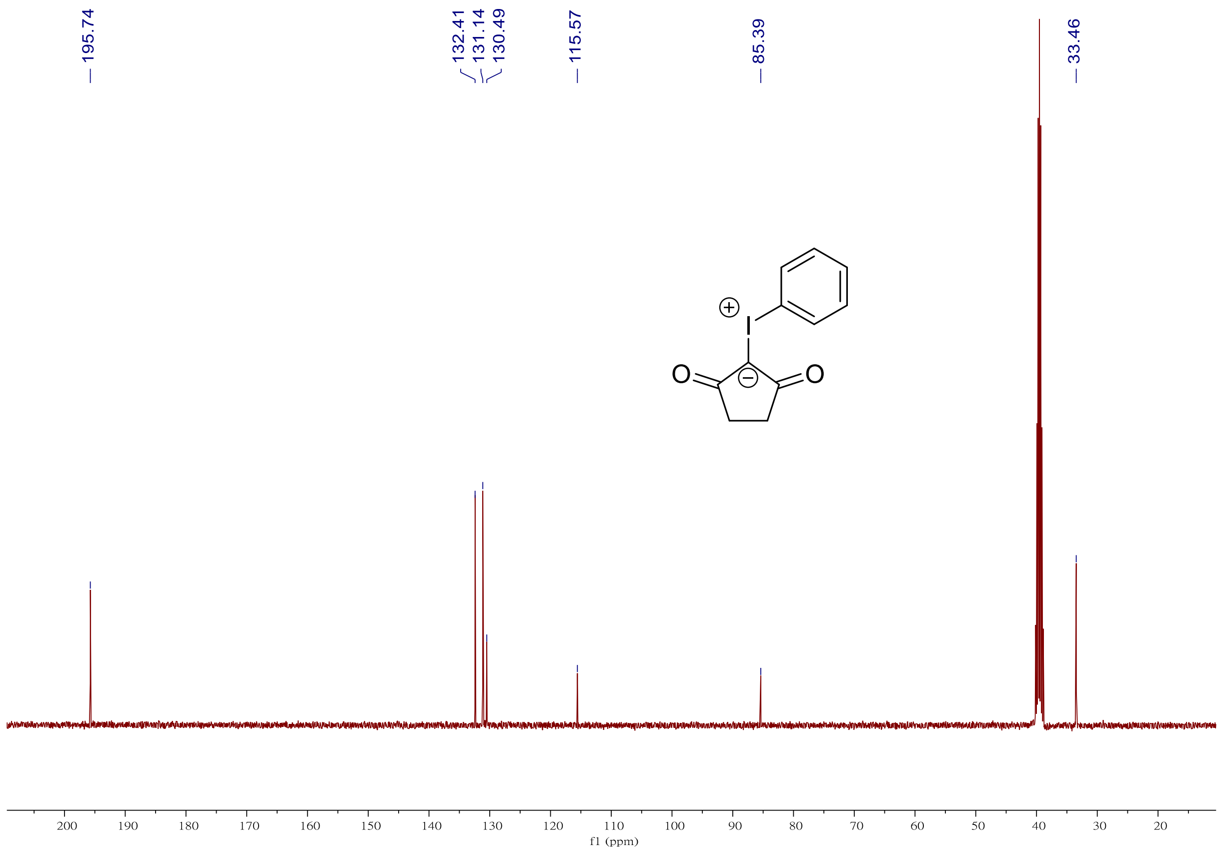


**Figure S100.** $^{13}C\{^{1}H\}$ NMR spectrum of compound **2f** (101 MHz, $(CD_3)_2SO$).

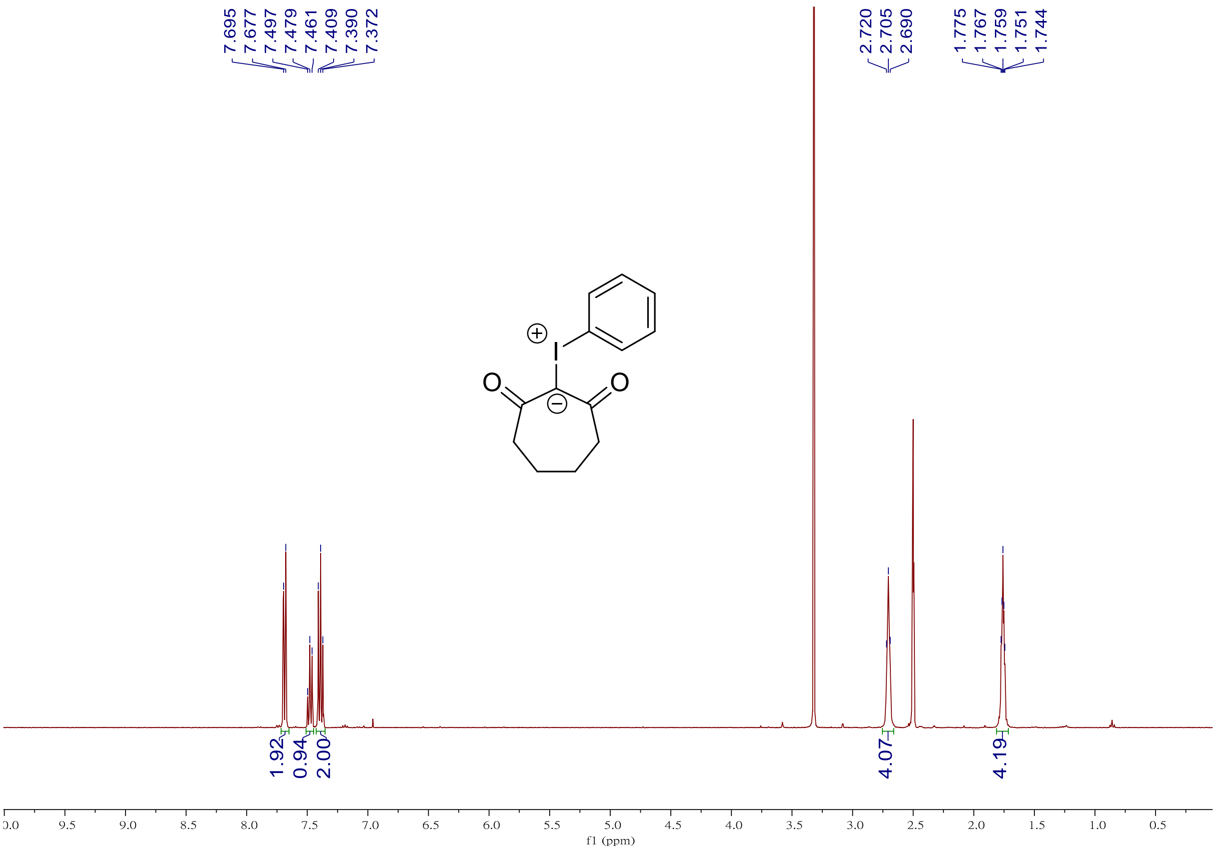


**Figure S101.** $^{1}H$ NMR spectrum of compound **2g** (400 MHz, $(CD_3)_2SO$).

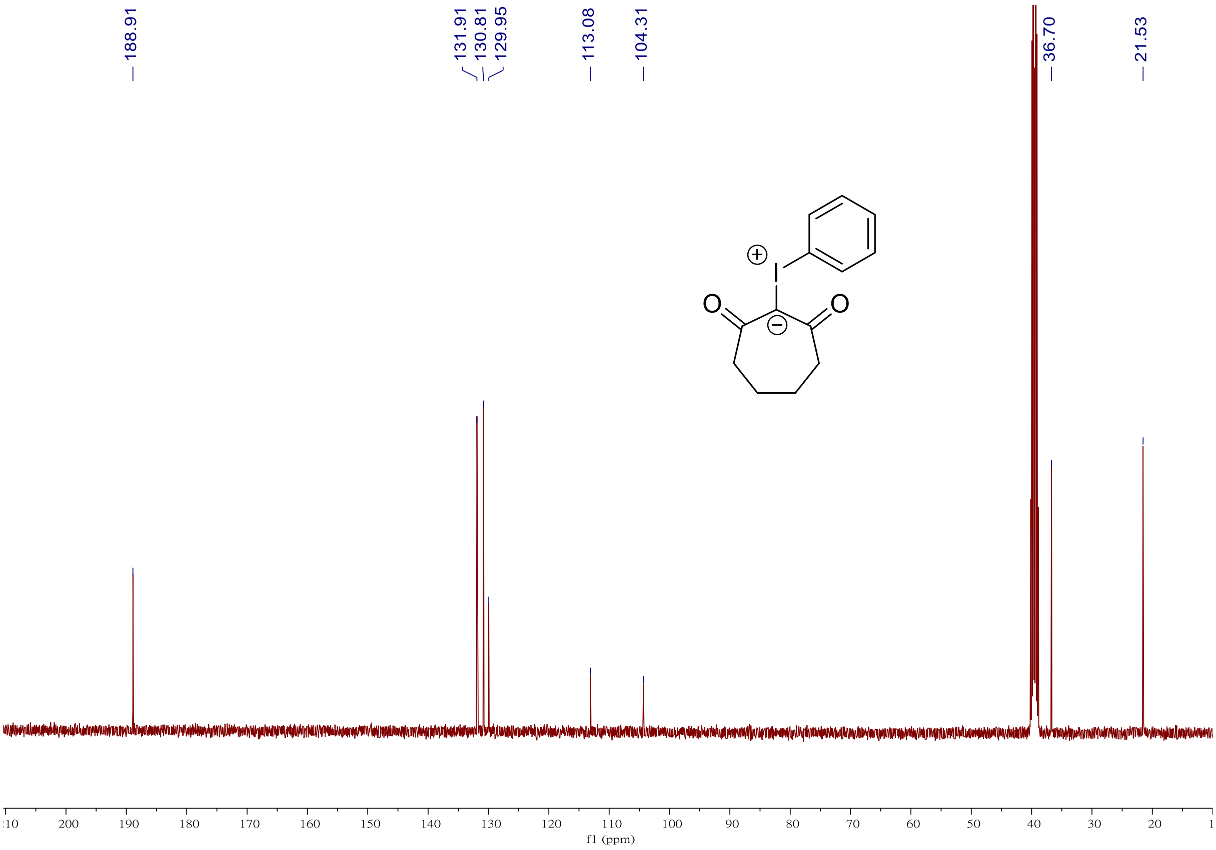


**Figure S102.** $^{13}C\{^{1}H\}$ NMR spectrum of compound **2g** (101 MHz, $(CD_3)_2SO$).

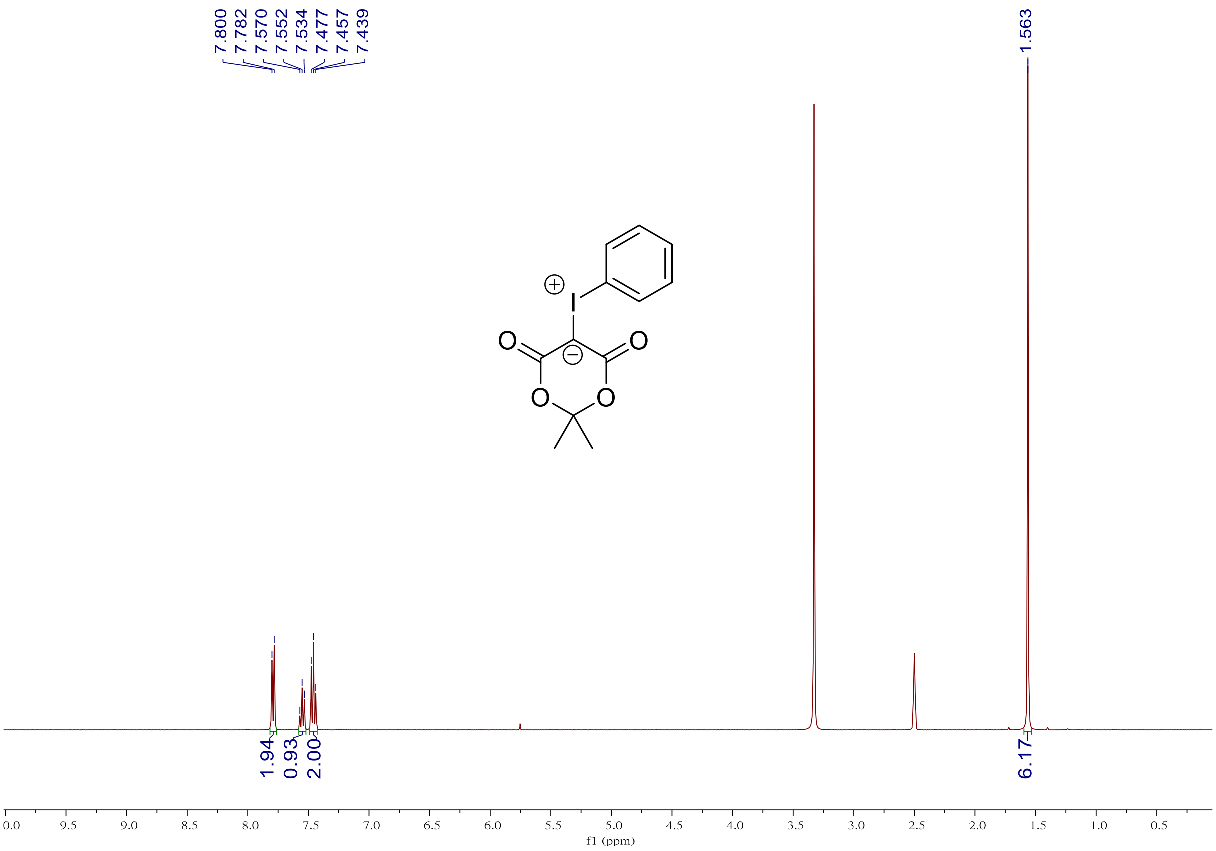


**Figure S103.** $^{1}H$ NMR spectrum of compound **2h** (400 MHz, $(CD_3)_2SO$).

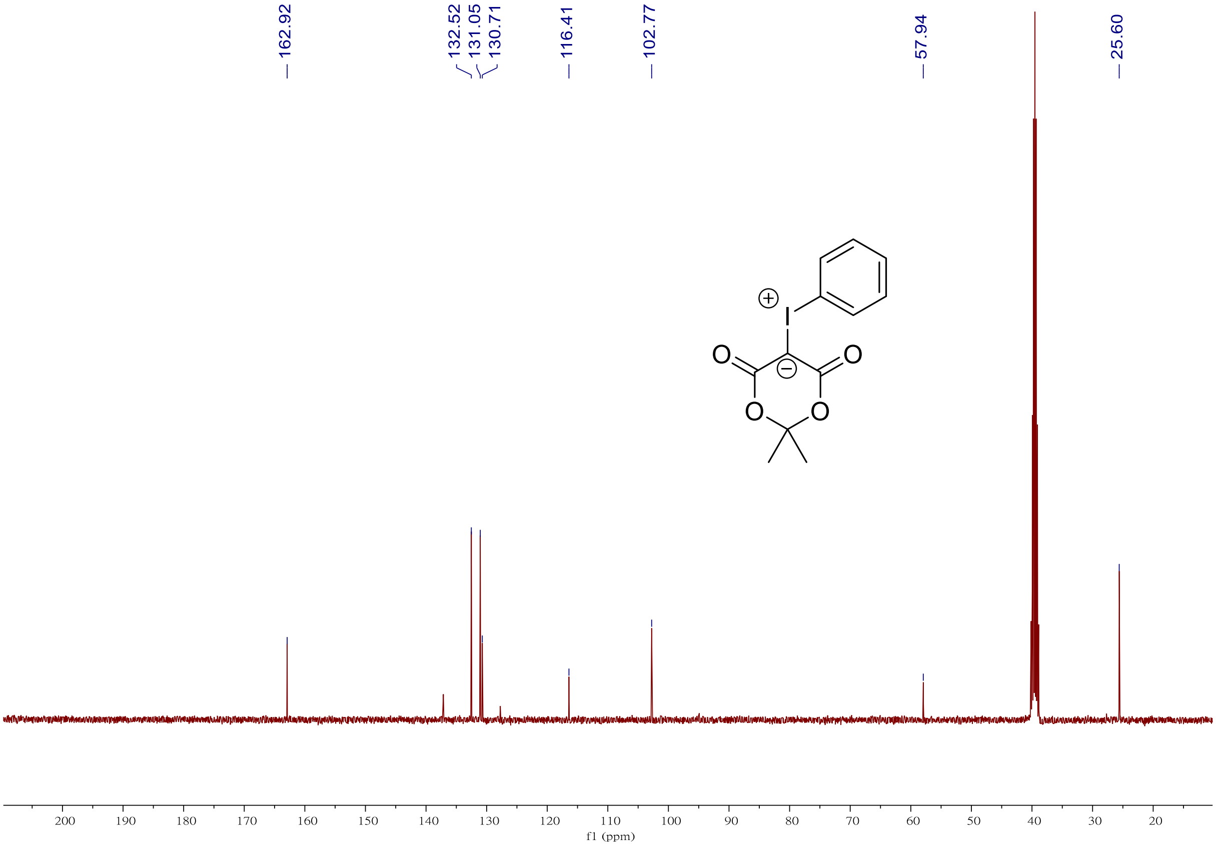


**Figure S104.** $^{13}C\{^{1}H\}$ NMR spectrum of compound **2h** (101 MHz, $(CD_3)_2SO$).

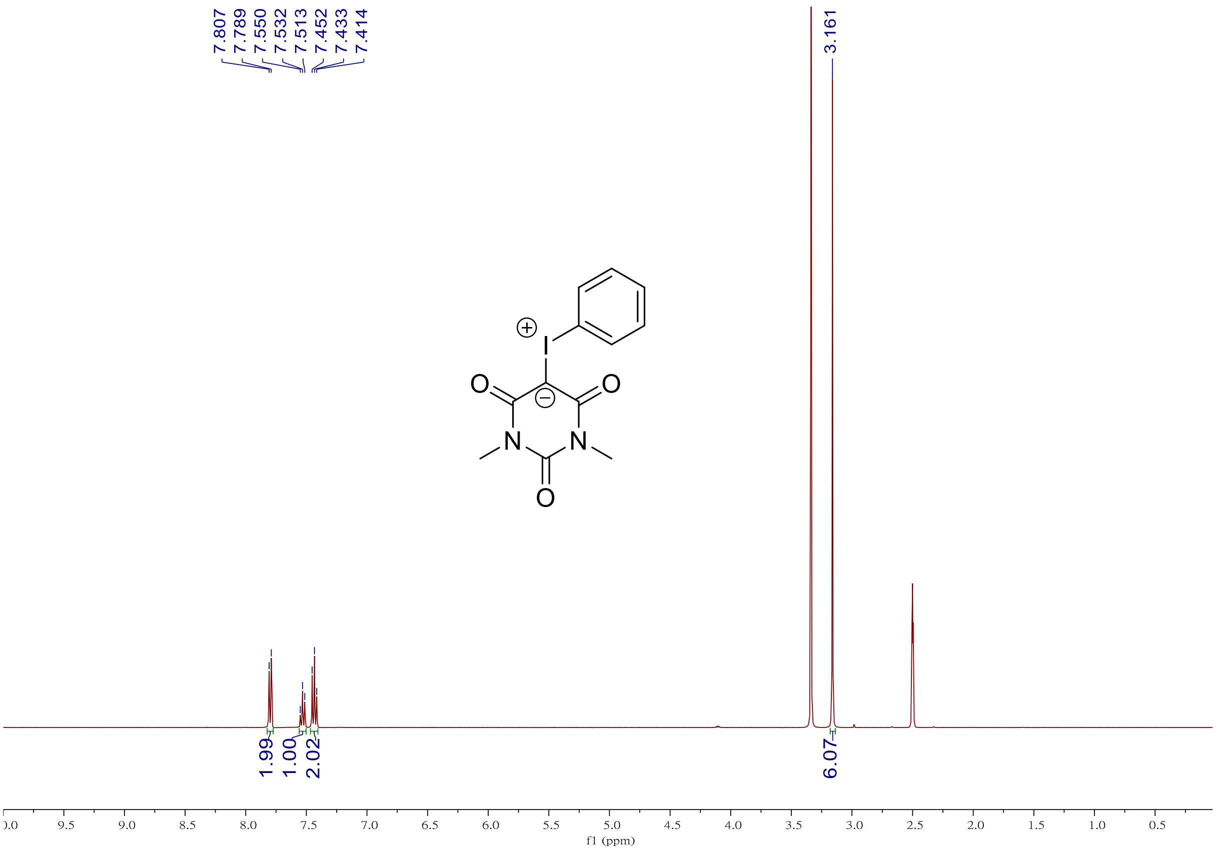


**Figure S105.** $^{1}$H NMR spectrum of compound **2i** (400 MHz, $(CD_3)_2SO$).

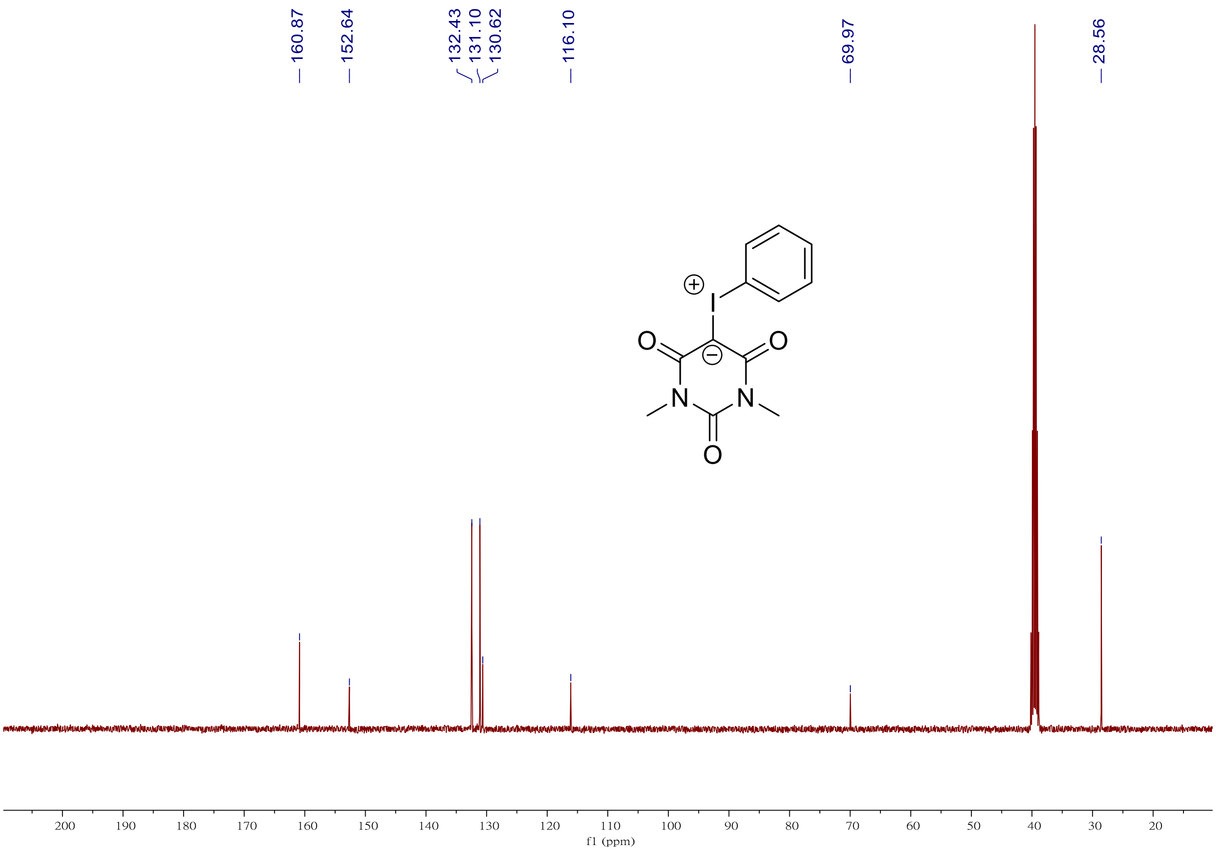


**Figure S106.** $^{13}C\{^{1}H\}$ NMR spectrum of compound **2i** (101 MHz, $(CD_3)_2SO$).

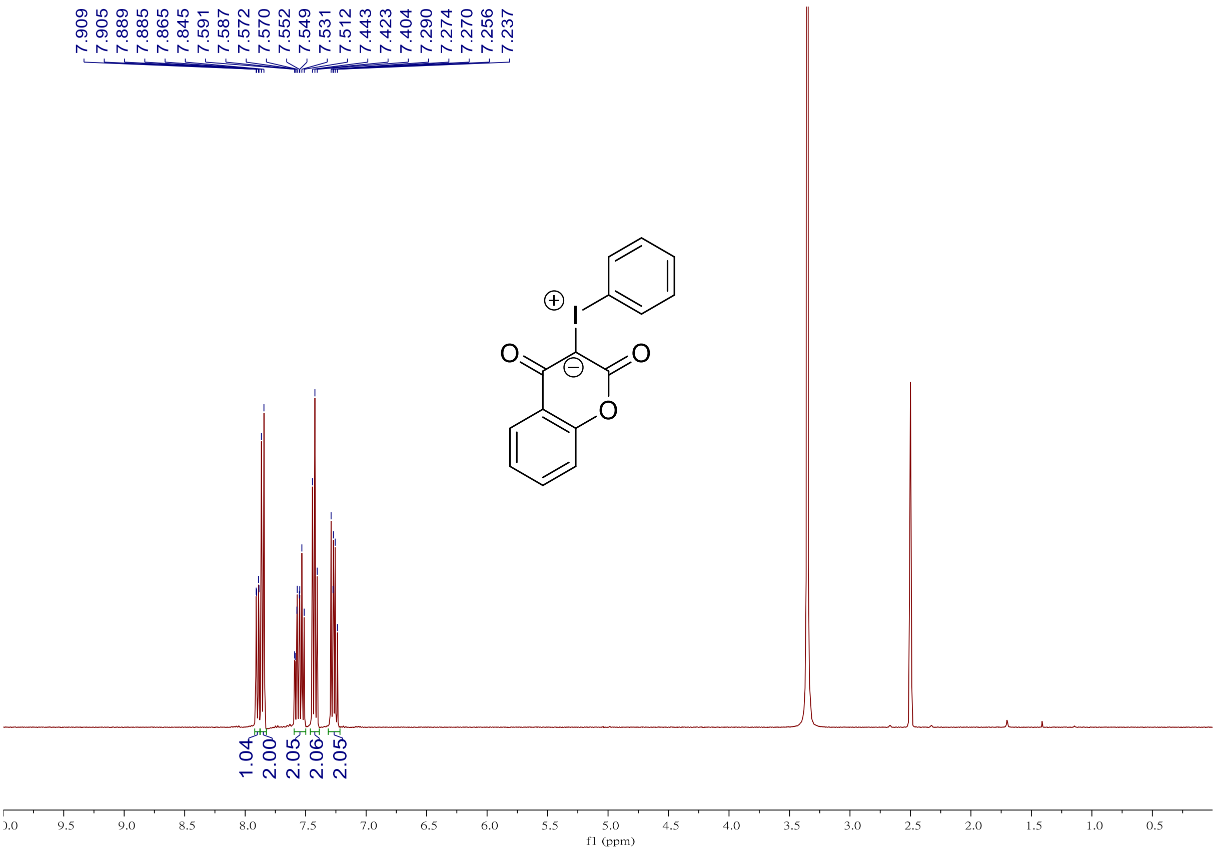


**Figure S107.** $^{1}$H NMR spectrum of compound **2j** (400 MHz, $(CD_3)_2SO$).

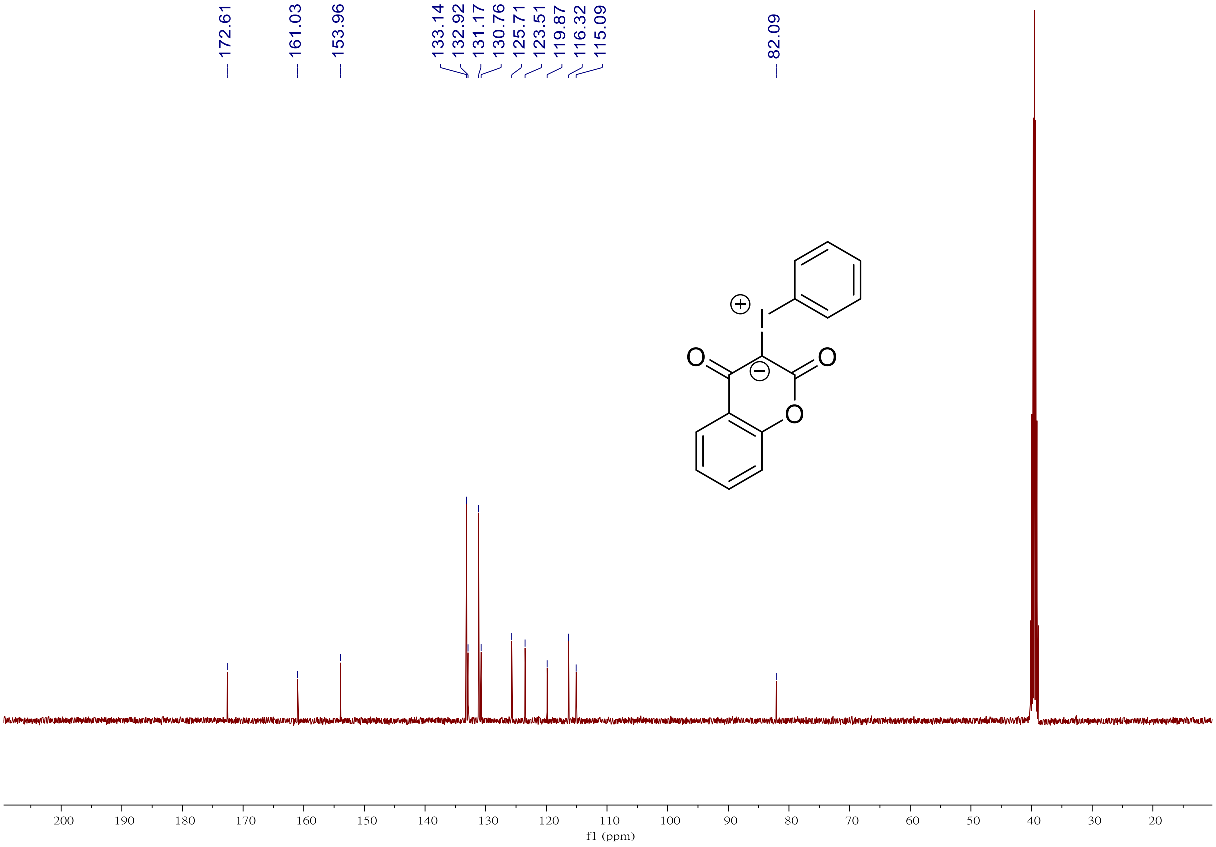


**Figure S108.** $^{13}C\{^{1}H\}$ NMR spectrum of compound **2j** (101 MHz, $(CD_3)_2SO$).

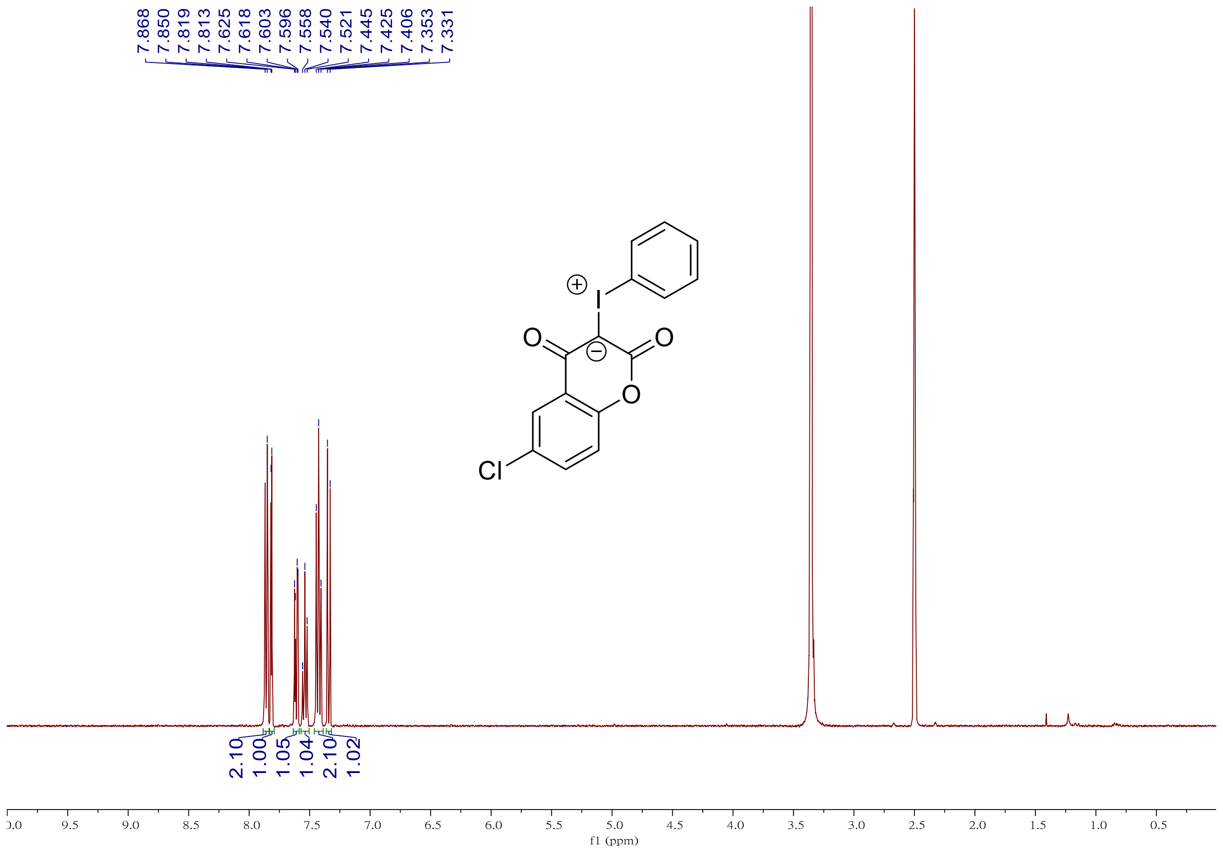


**Figure S109.** $^{1}$H NMR spectrum of compound **2k** (400 MHz, $(CD_3)_2SO$).

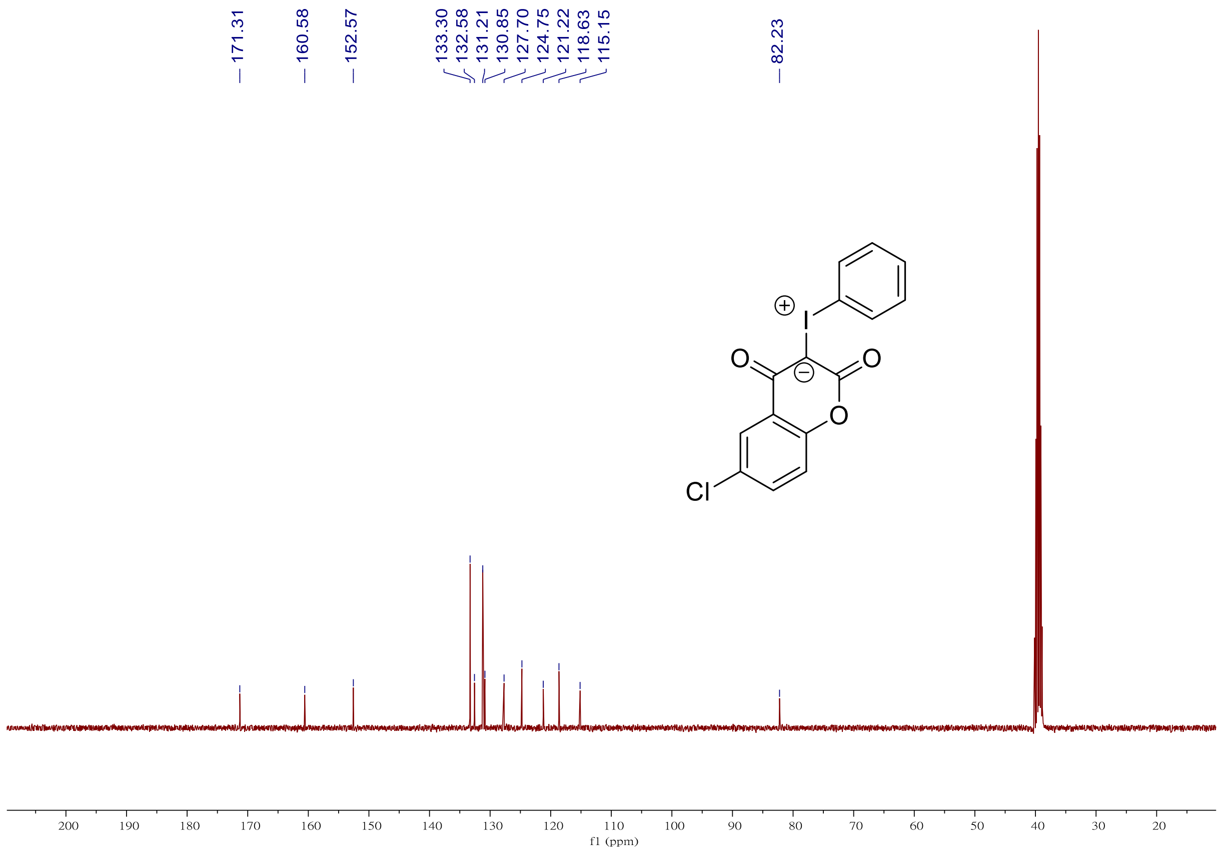


**Figure S110.** $^{13}C\{^{1}H\}$ NMR spectrum of compound **2k** (101 MHz, $(CD_3)_2SO$).

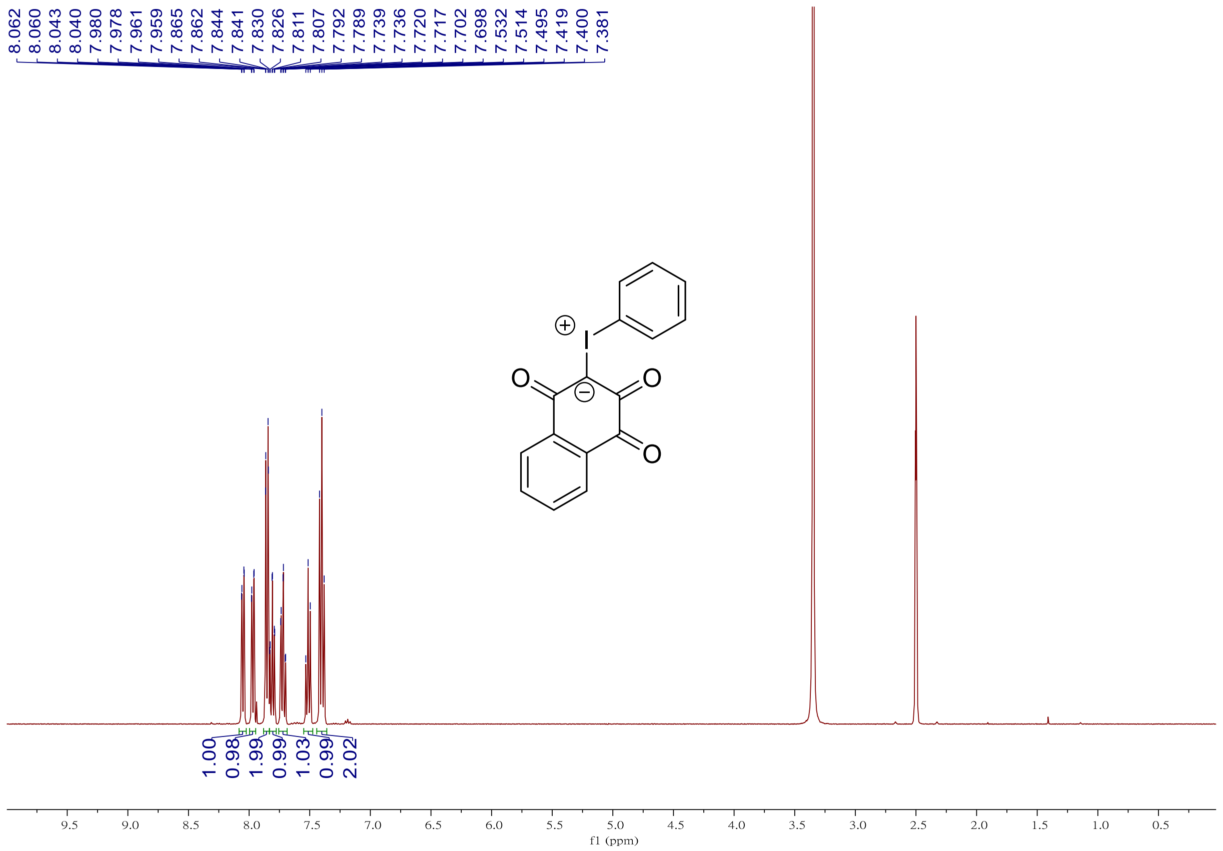


**Figure S111.** $^{1}H$ NMR spectrum of compound **2l** (400 MHz, $(CD_3)_2SO$).

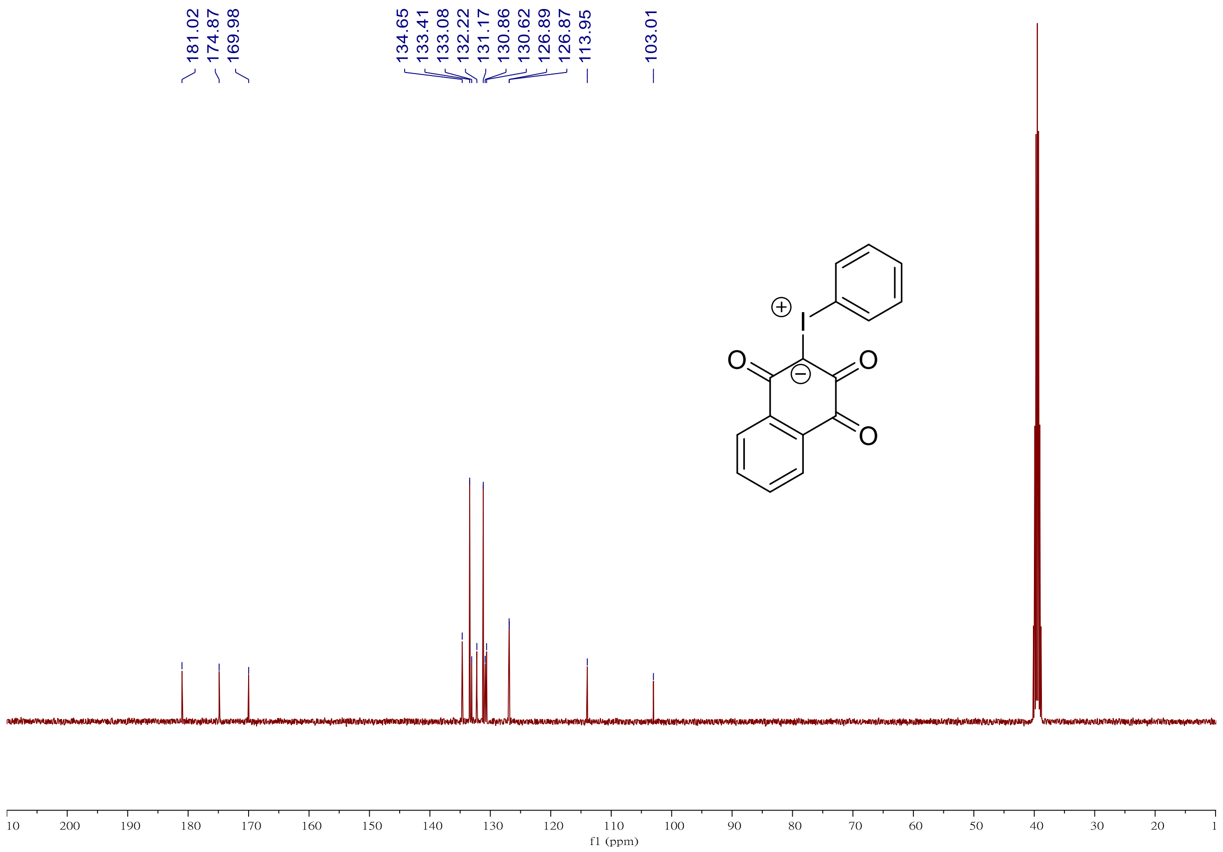


**Figure S112.** $^{13}C\{^{1}H\}$ NMR spectrum of compound **2l** (101 MHz, $(CD_3)_2SO$).

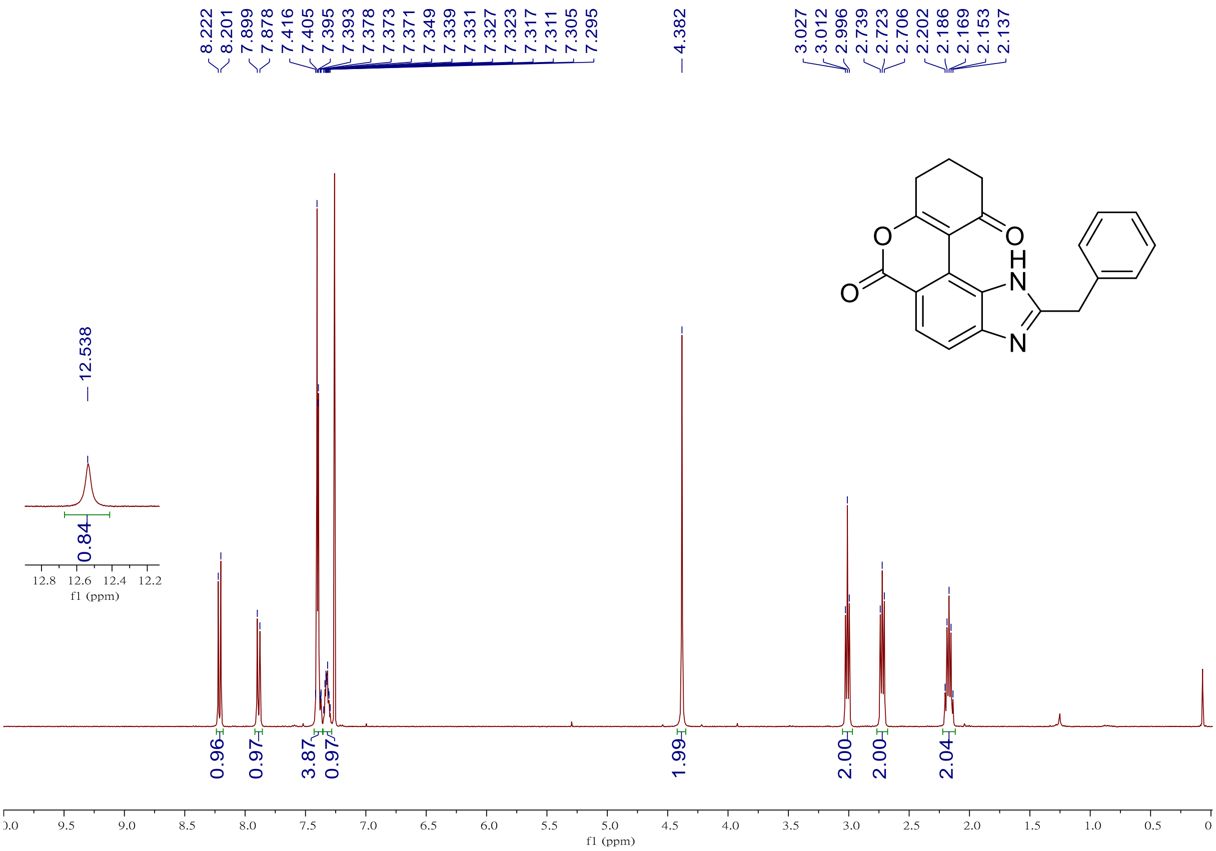

**Figure S113.** $^{1}H$ NMR spectrum of compound **3a** (400 MHz, $CDCl_3$).

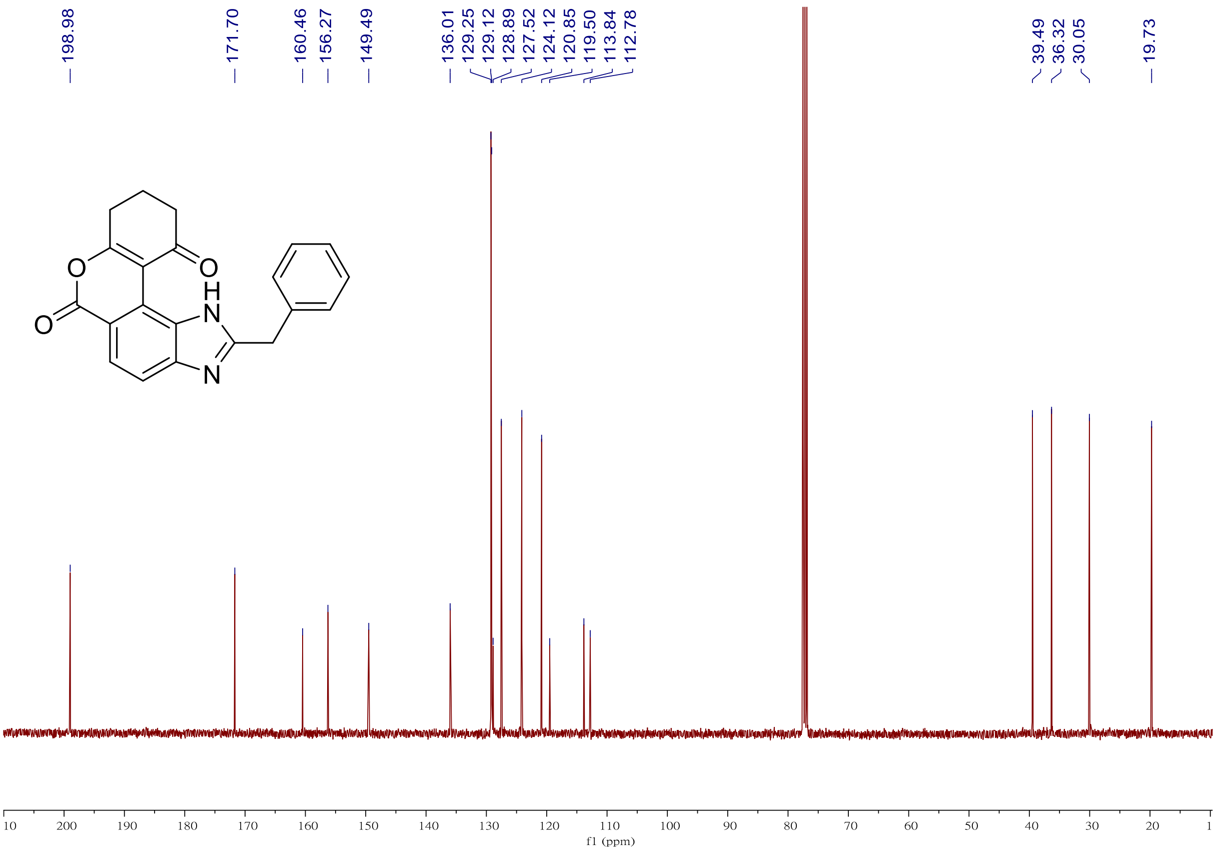

**Figure S114.** $^{13}C\{^{1}H\}$ NMR spectrum of compound **3a** (101 MHz, $CDCl_3$).

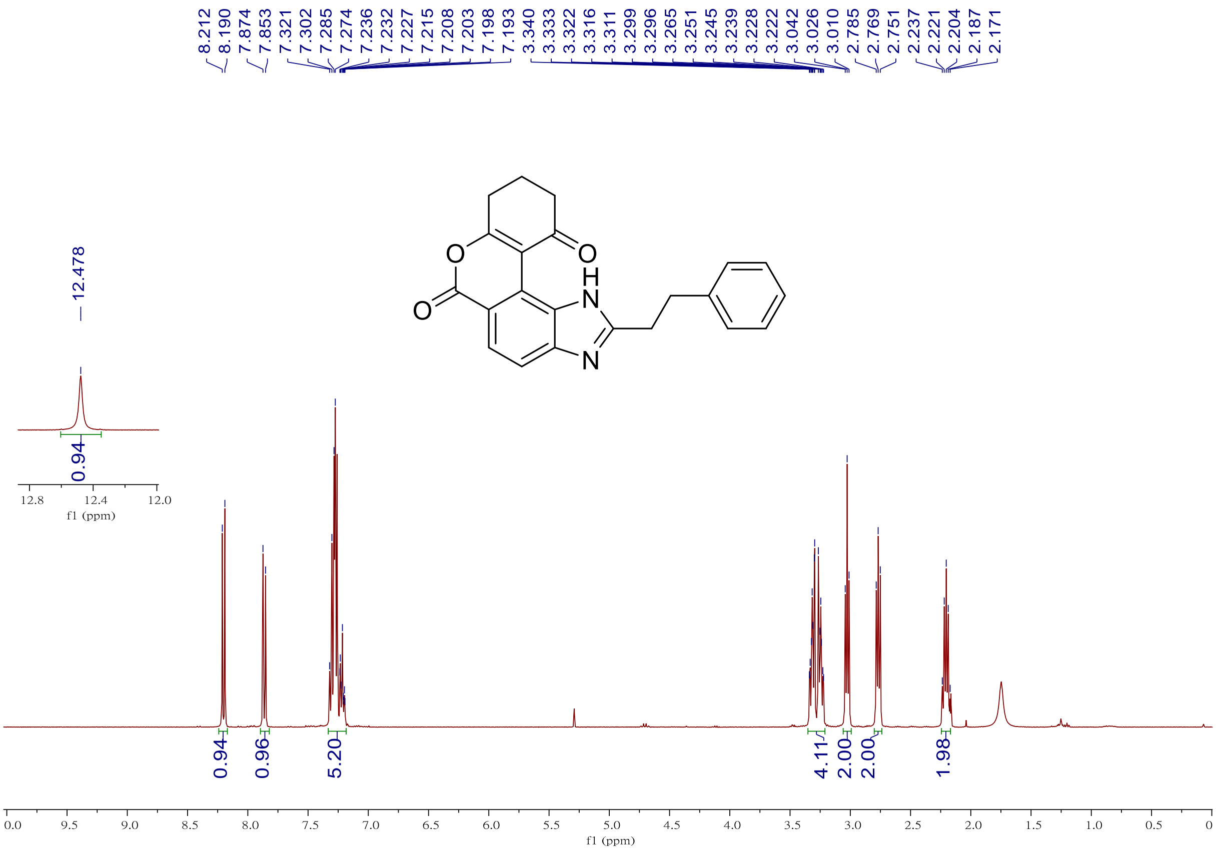

**Figure S115.** $^{1}H$ NMR spectrum of compound **3b** (400 MHz, $CDCl_3$).

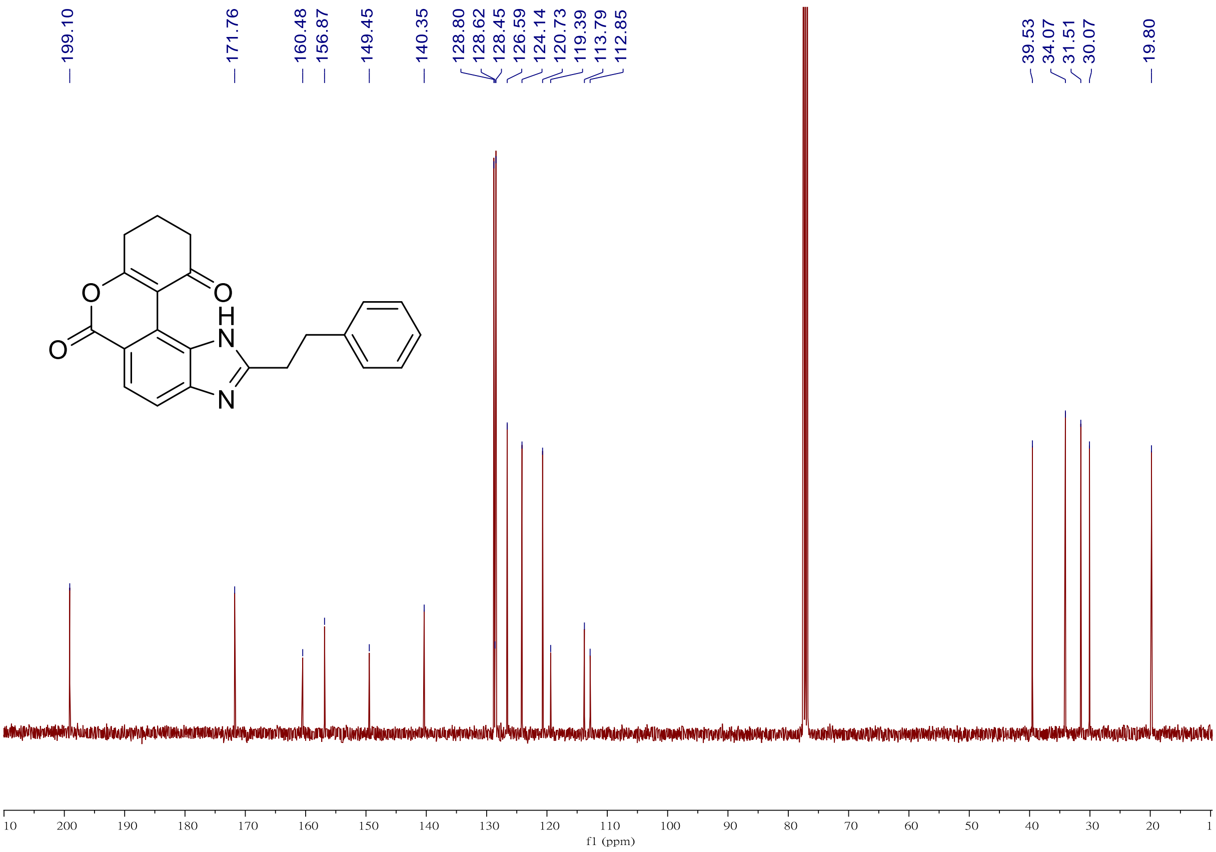

**Figure S116.** $^{13}C\{^{1}H\}$ NMR spectrum of compound **3b** (101 MHz, $CDCl_3$).

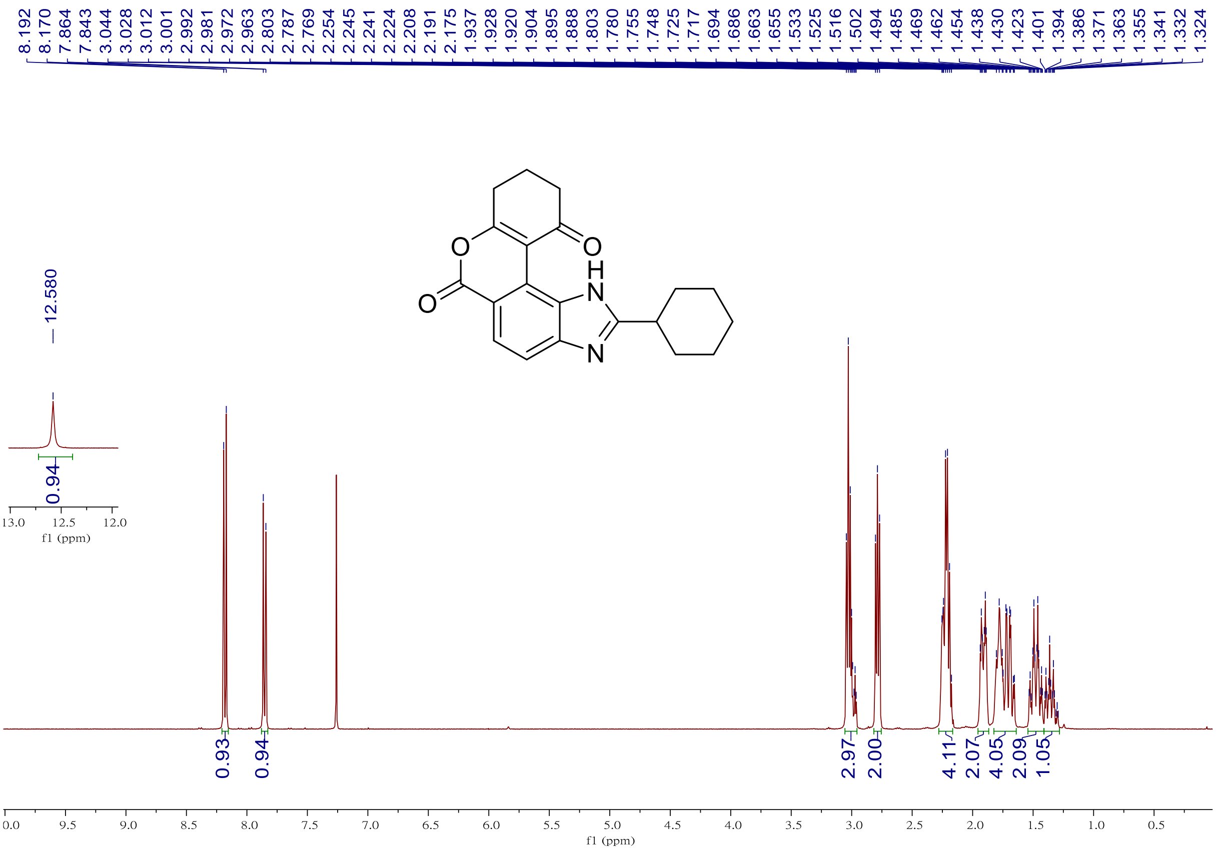


**Figure S117.** $^{1}H$ NMR spectrum of compound **3c** (400 MHz, $CDCl_3$).

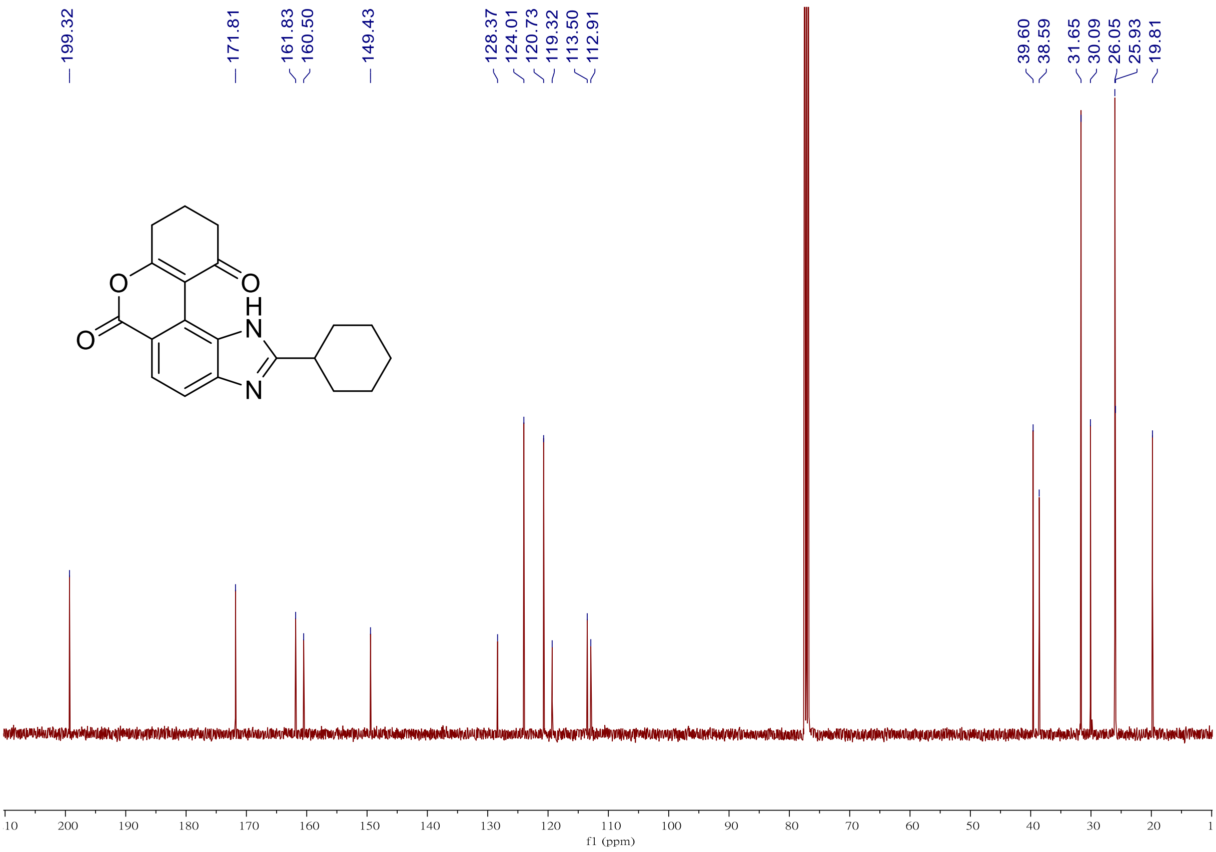


**Figure S118.** $^{13}C\{^{1}H\}$ NMR spectrum of compound **3c** (101 MHz, $CDCl_3$).

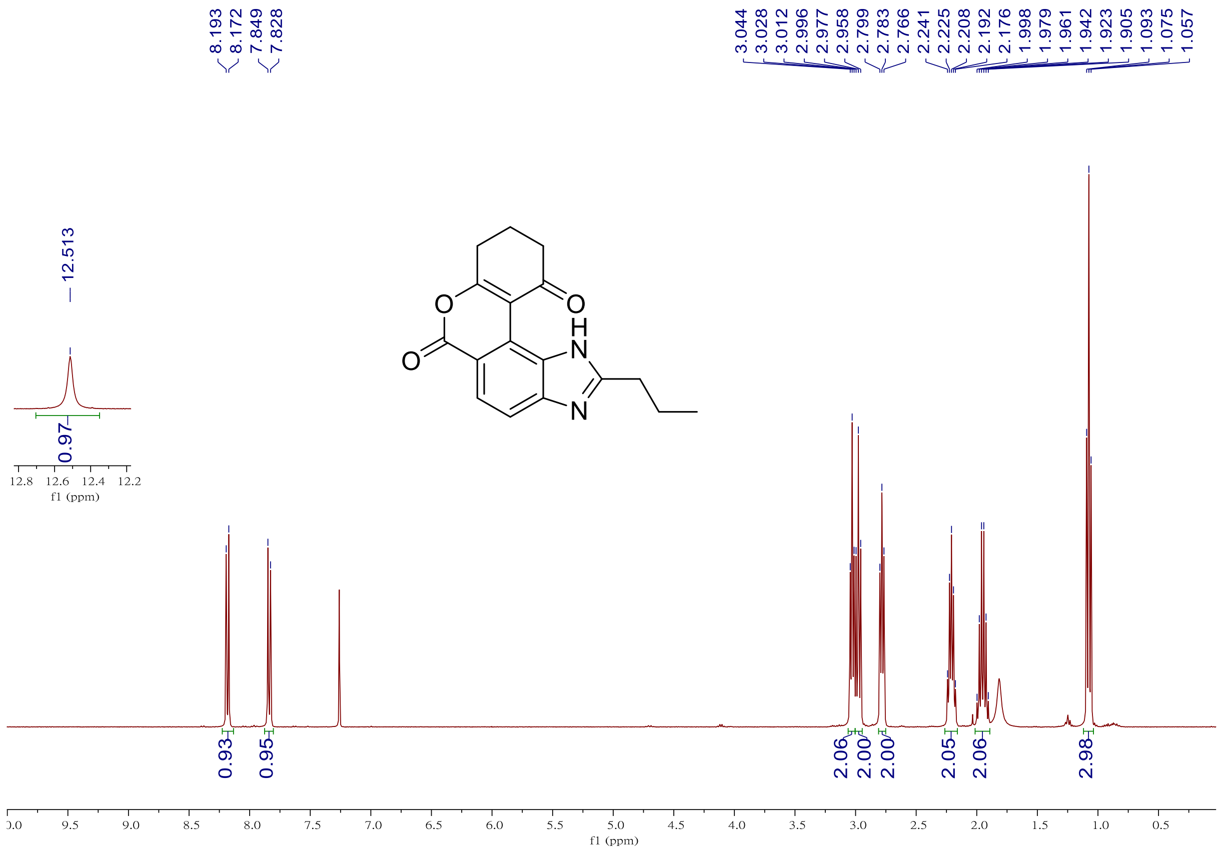


**Figure S119.** $^{1}$H NMR spectrum of compound **3d** (400 MHz, $CDCl_3$).

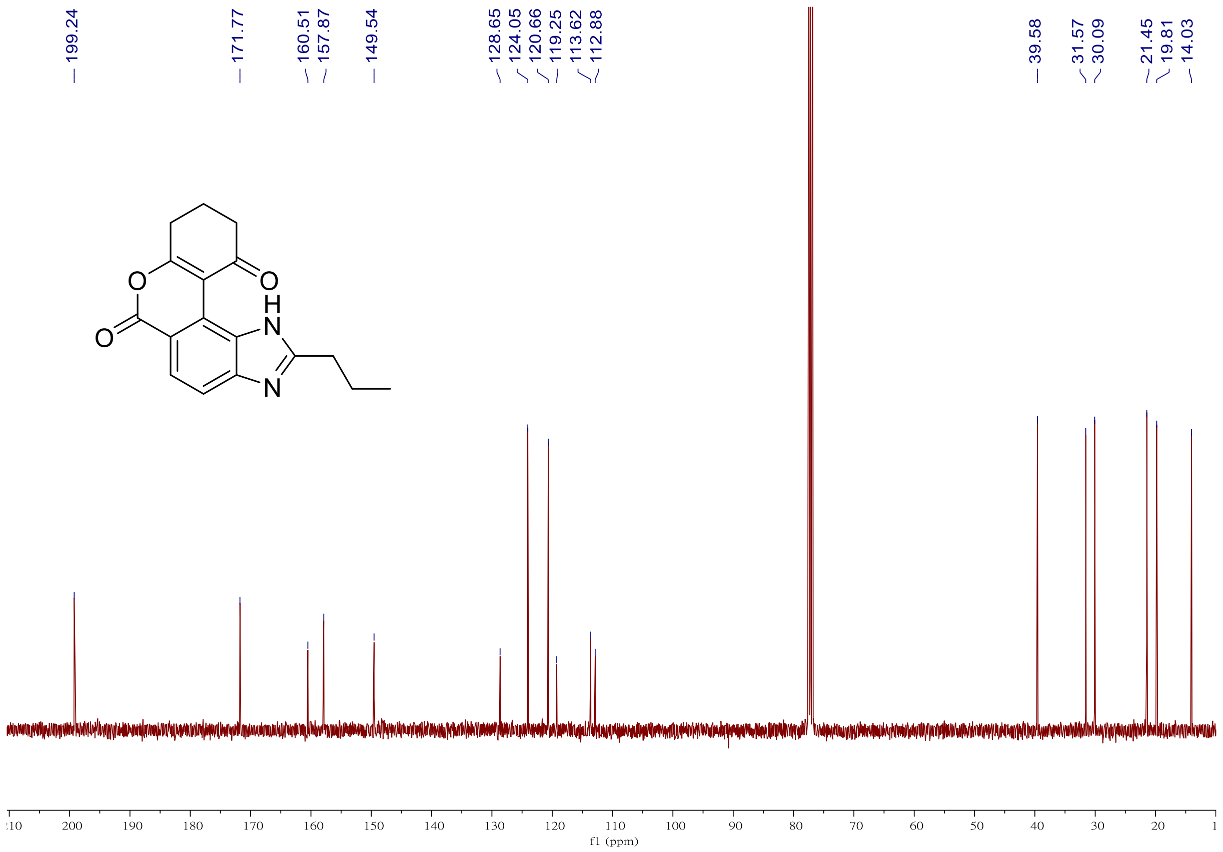


**Figure S120.** $^{13}C\{^{1}H\}$ NMR spectrum of compound **3d** (101 MHz, $CDCl_3$).

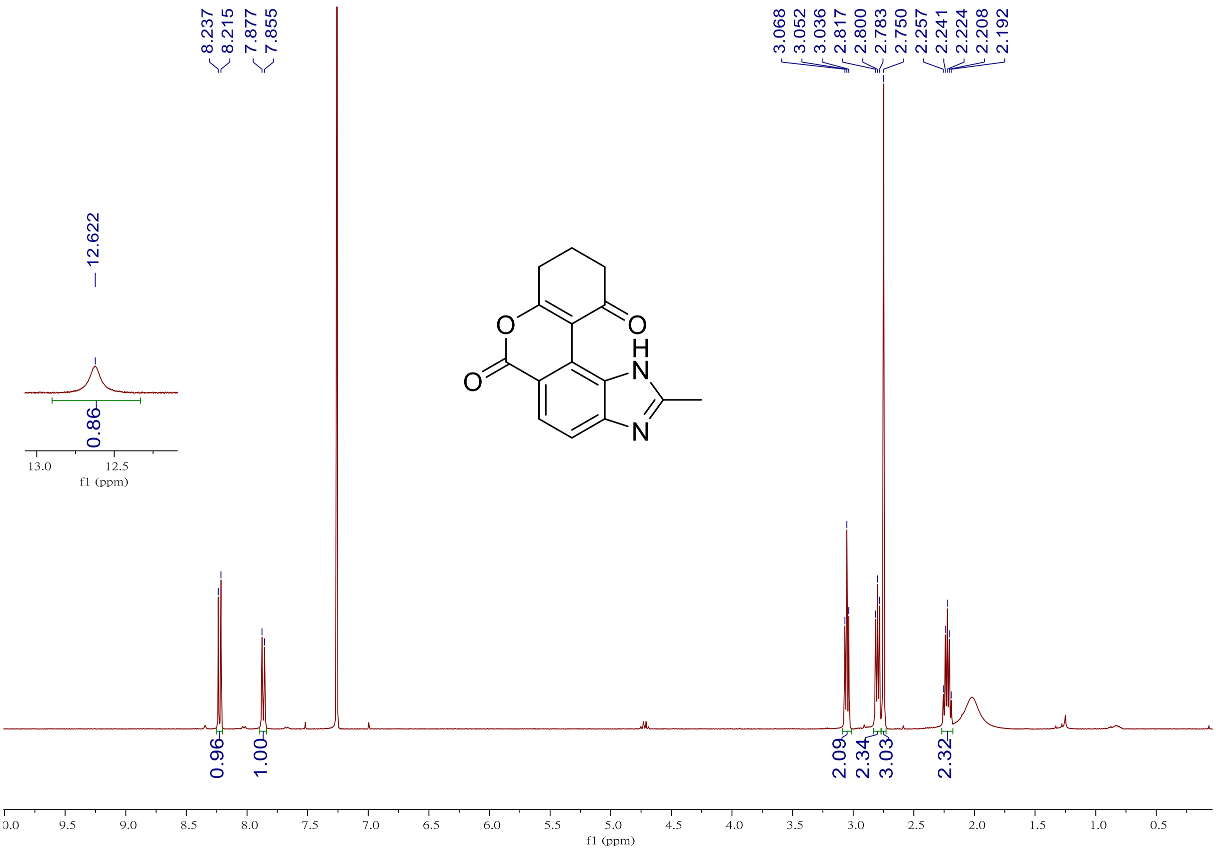


**Figure S121.** $^{1}H$ NMR spectrum of compound **3e** (400 MHz, $CDCl_3$).

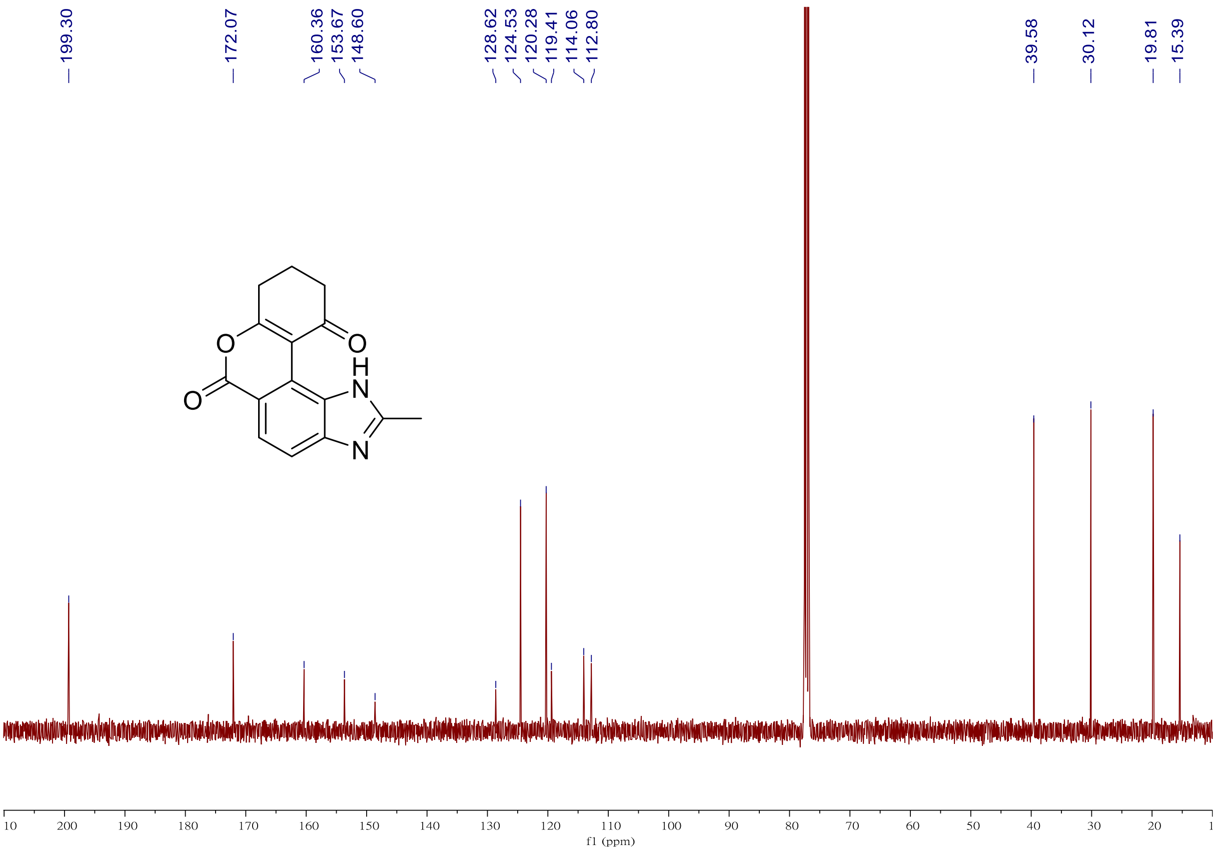


**Figure S122.** $^{13}C\{^{1}H\}$ NMR spectrum of compound **3e** (101 MHz, $CDCl_3$).

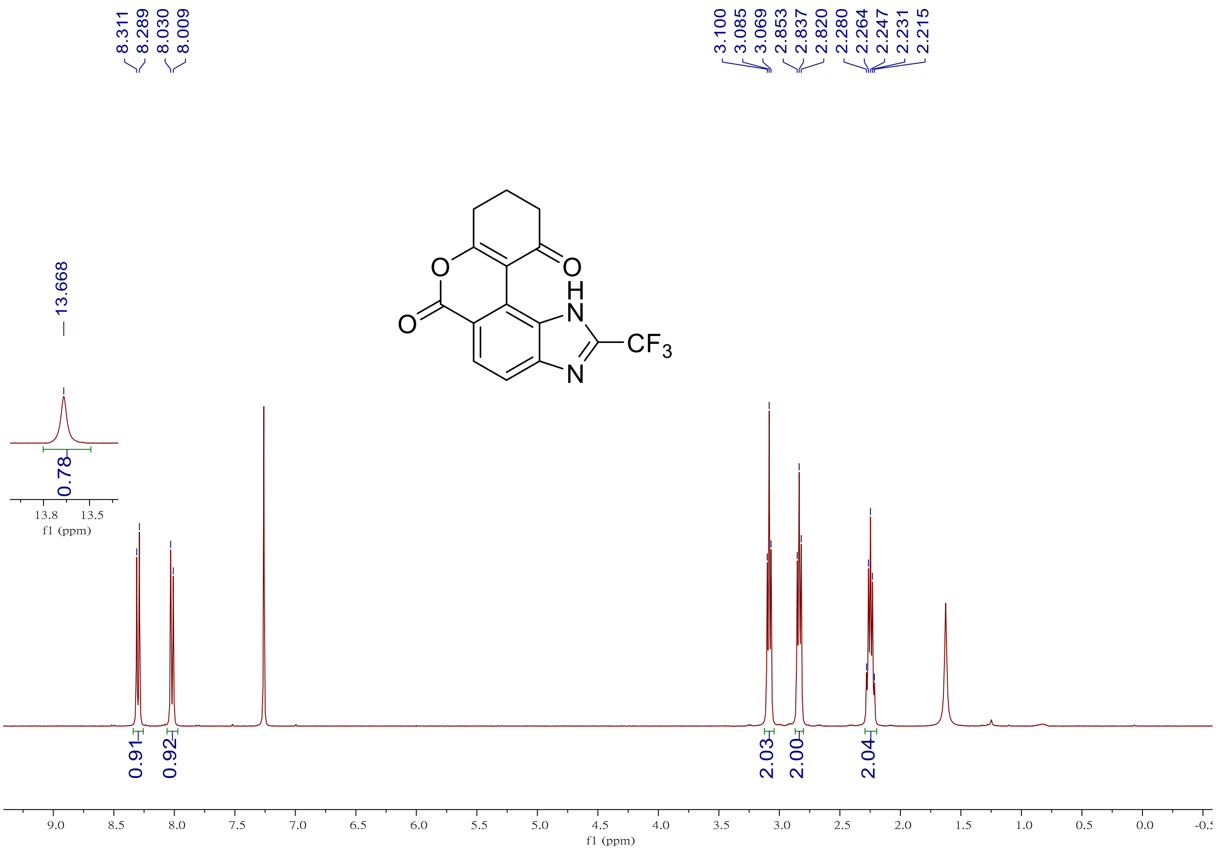


**Figure S123.** $^1$H NMR spectrum of compound **3f** (400 MHz, $CDCl_3$).

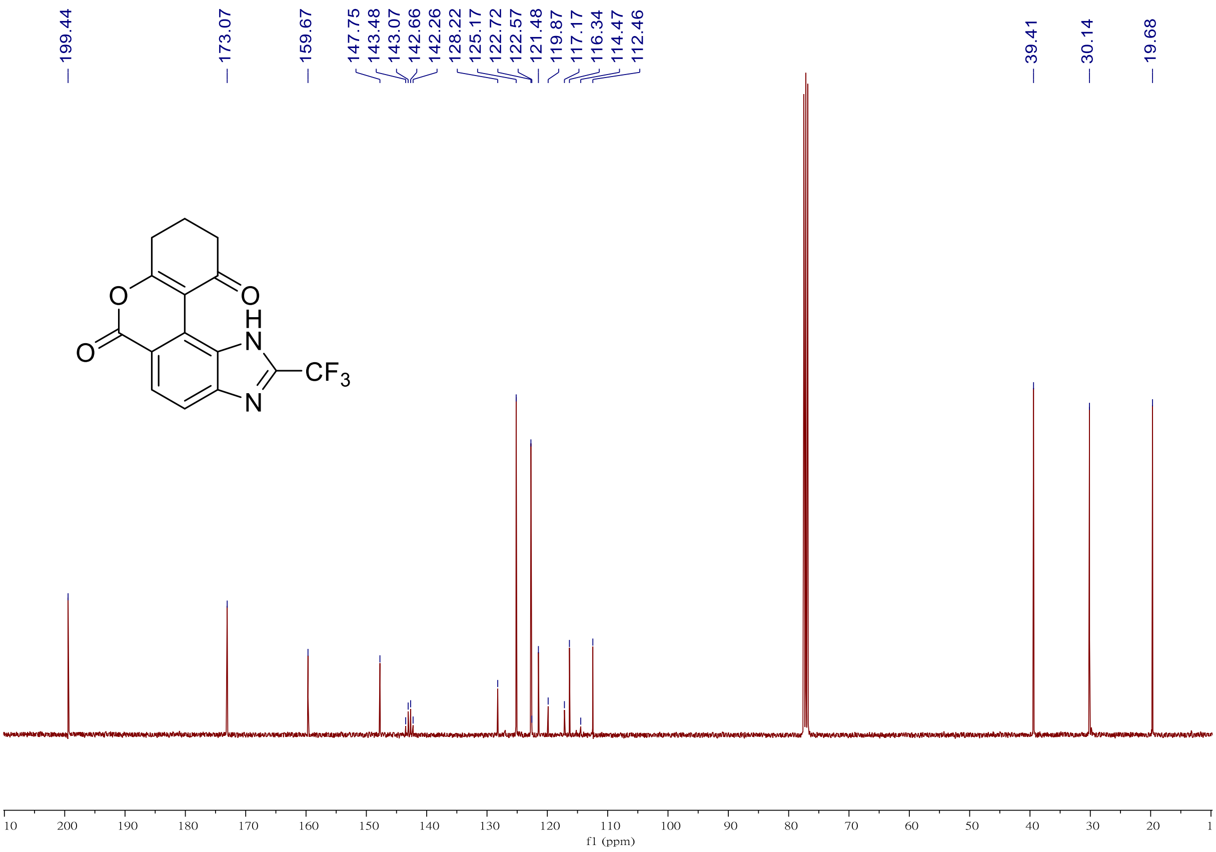


**Figure S124.** $^{13}$C{$^1$H} NMR spectrum of compound **3f** (101 MHz, $CDCl_3$).

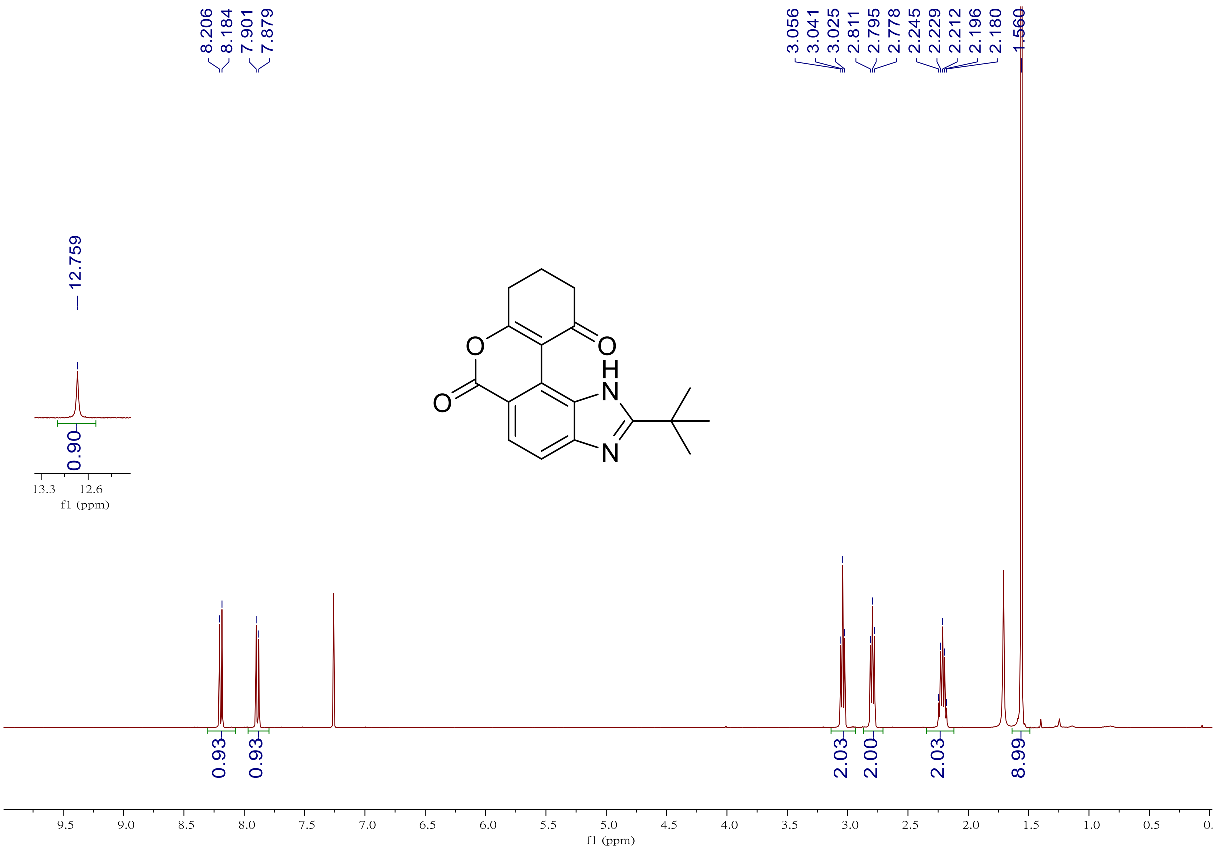


**Figure S125.** $^{1}H$ NMR spectrum of compound **3g** (400 MHz, $CDCl_3$).

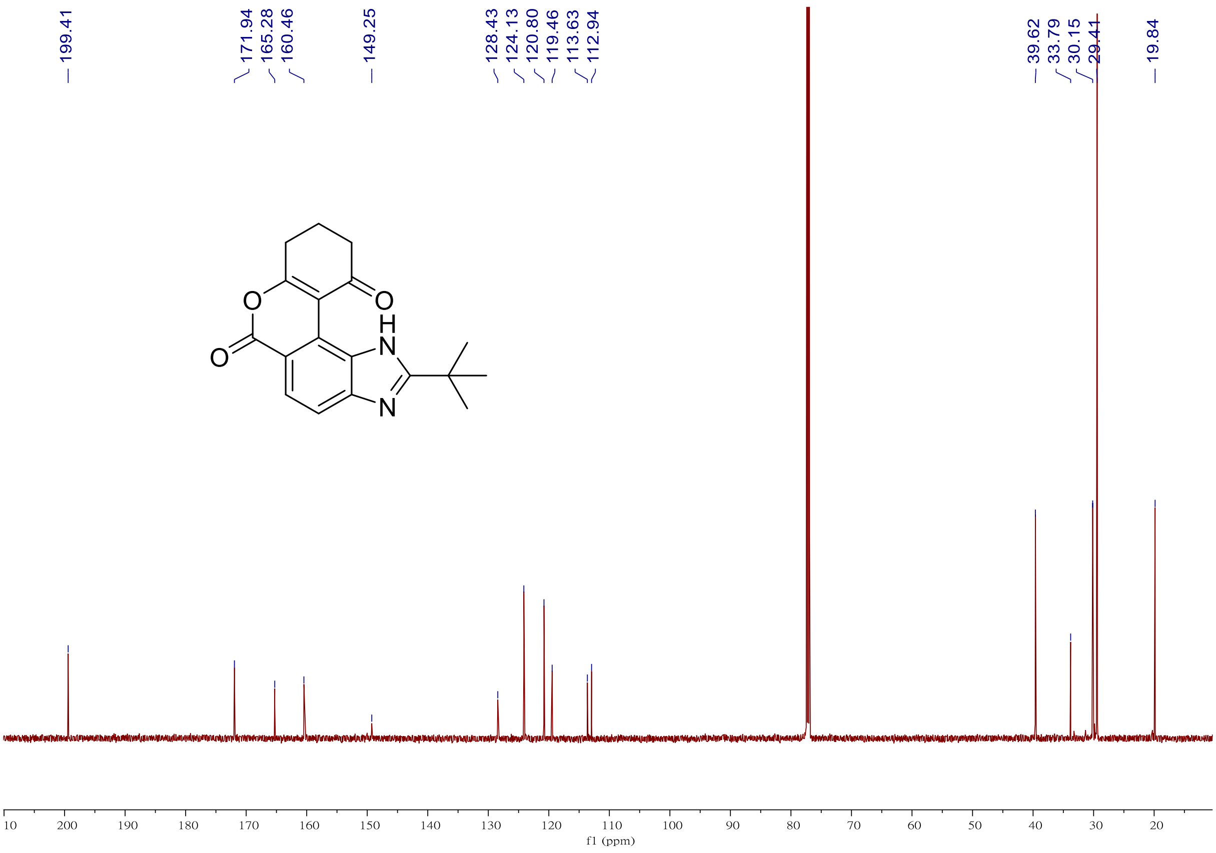


**Figure S126.** $^{13}C\{^{1}H\}$ NMR spectrum of compound **3g** (151 MHz, $CDCl_3$).

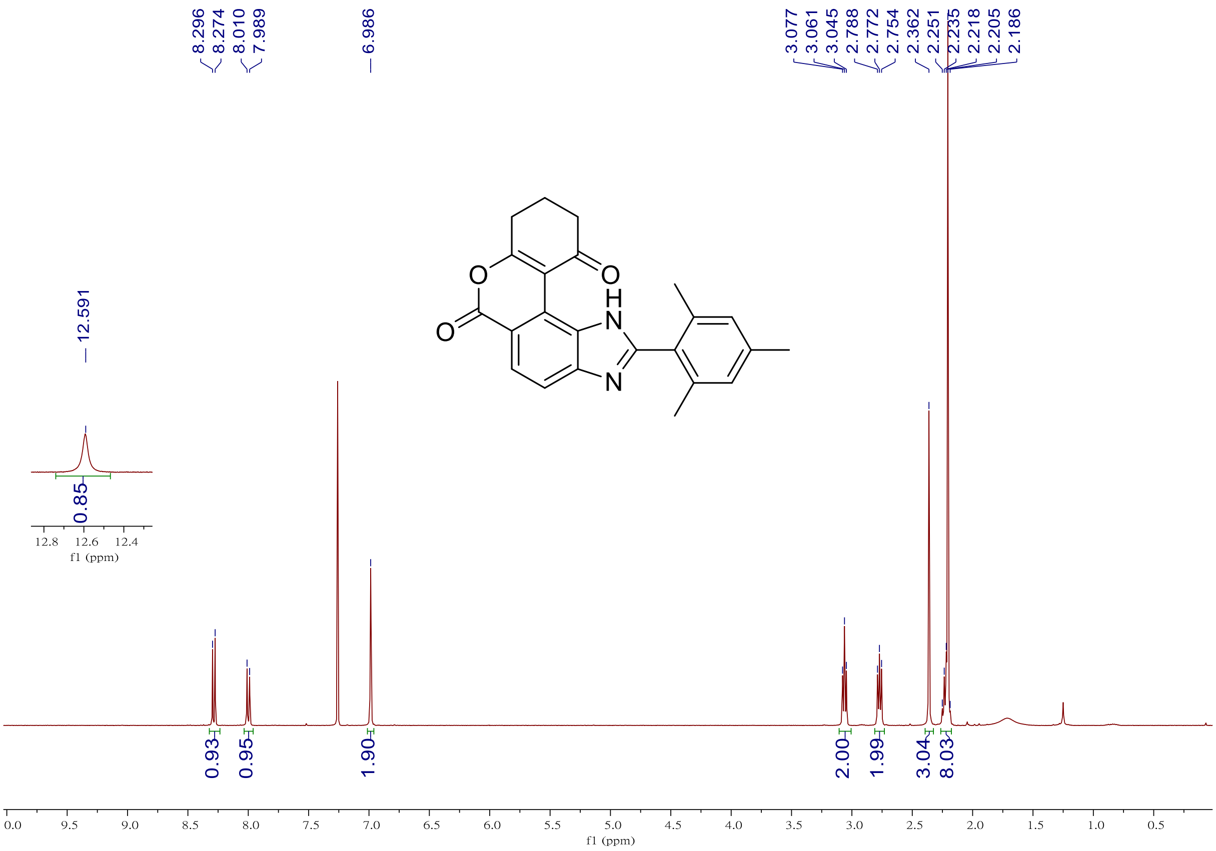


**Figure S127.** $^{1}H$ NMR spectrum of compound **3h** (400 MHz, $CDCl_3$).

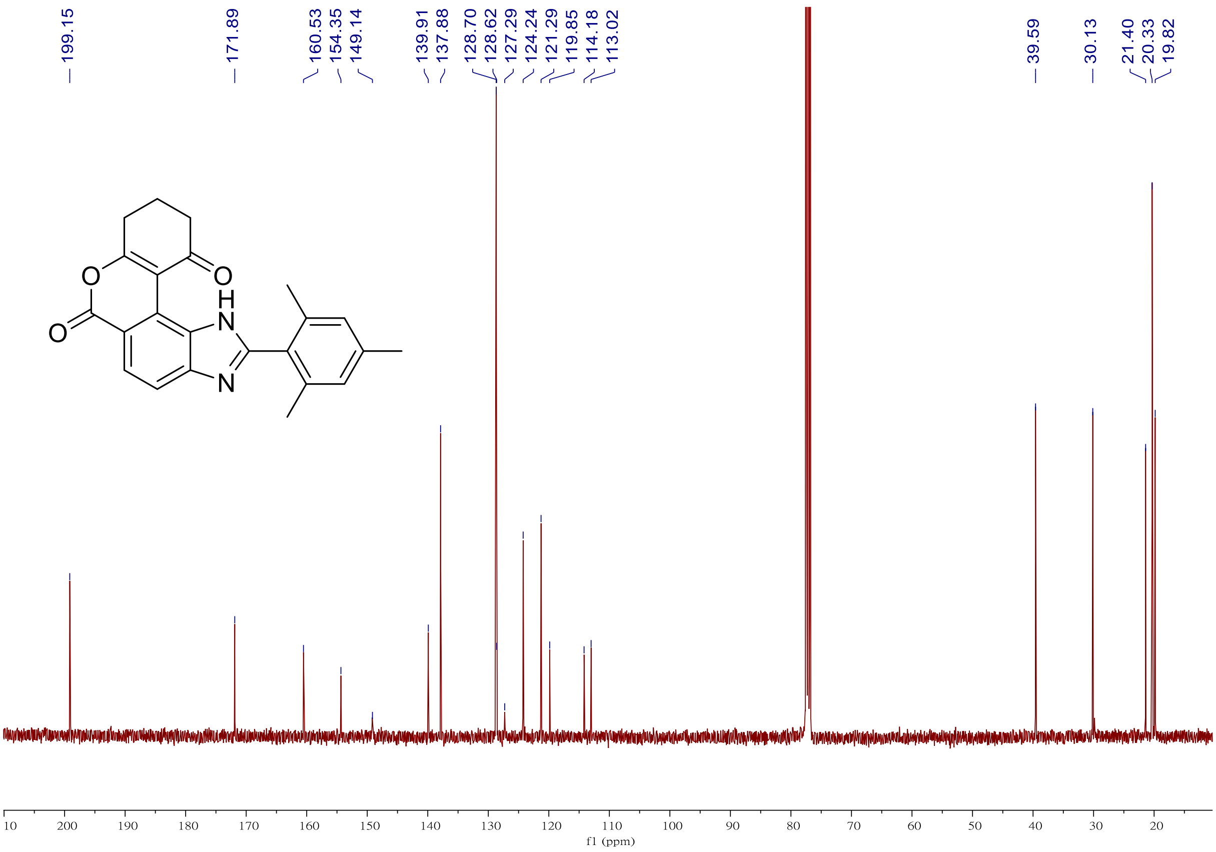


**Figure S128.** $^{13}C\{^{1}H\}$ NMR spectrum of compound **3h** (101 MHz, $CDCl_3$).

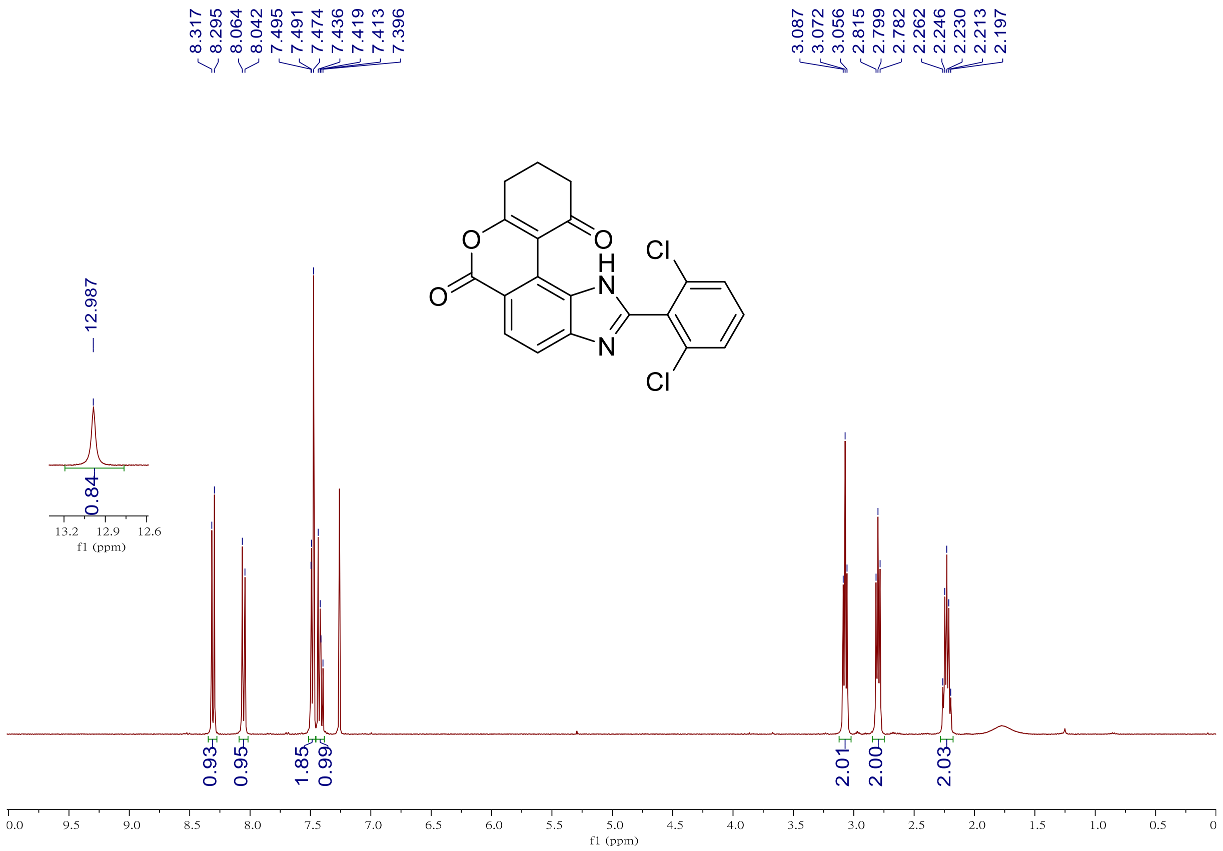

**Figure S129.** $^{1}$H NMR spectrum of compound **3i** (400 MHz, $CDCl_3$).

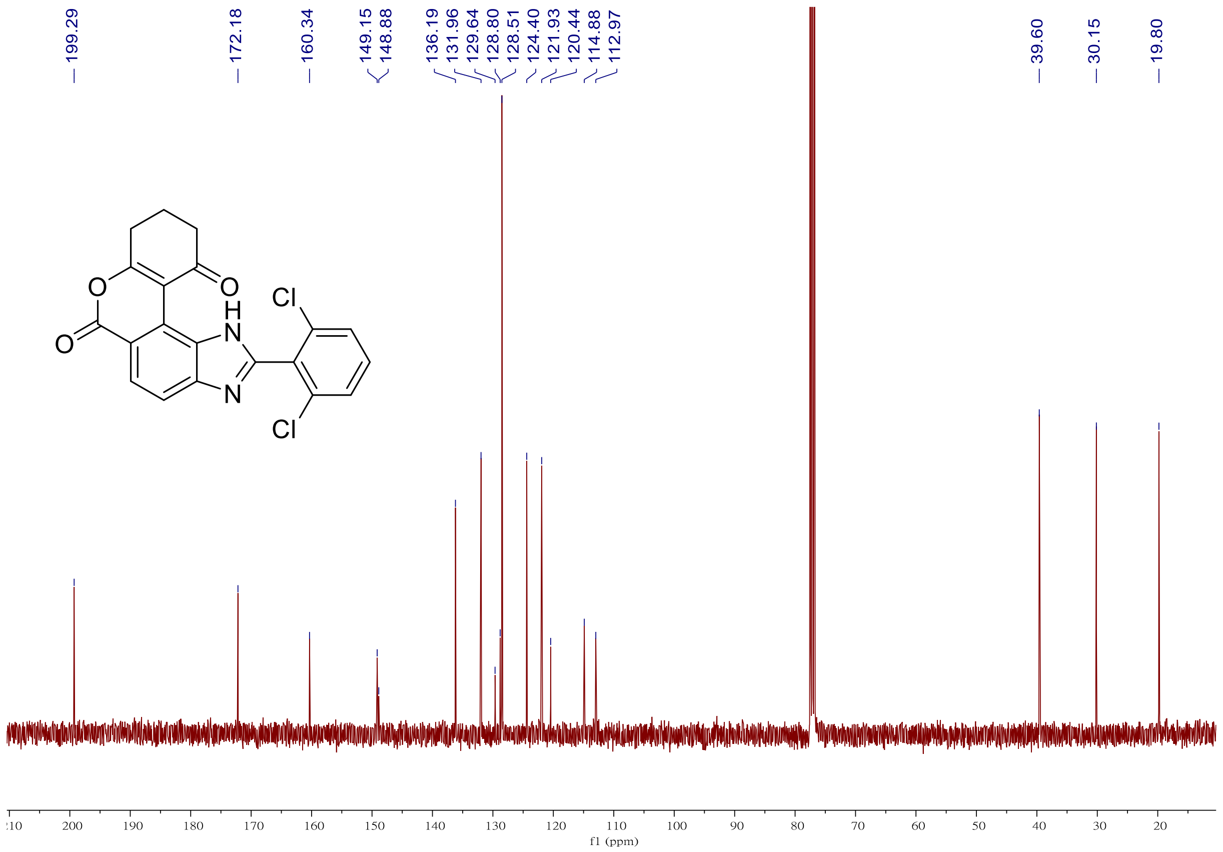

**Figure S130.** $^{13}C\{^{1}H\}$ NMR spectrum of compound **3i** (101 MHz, $CDCl_3$).

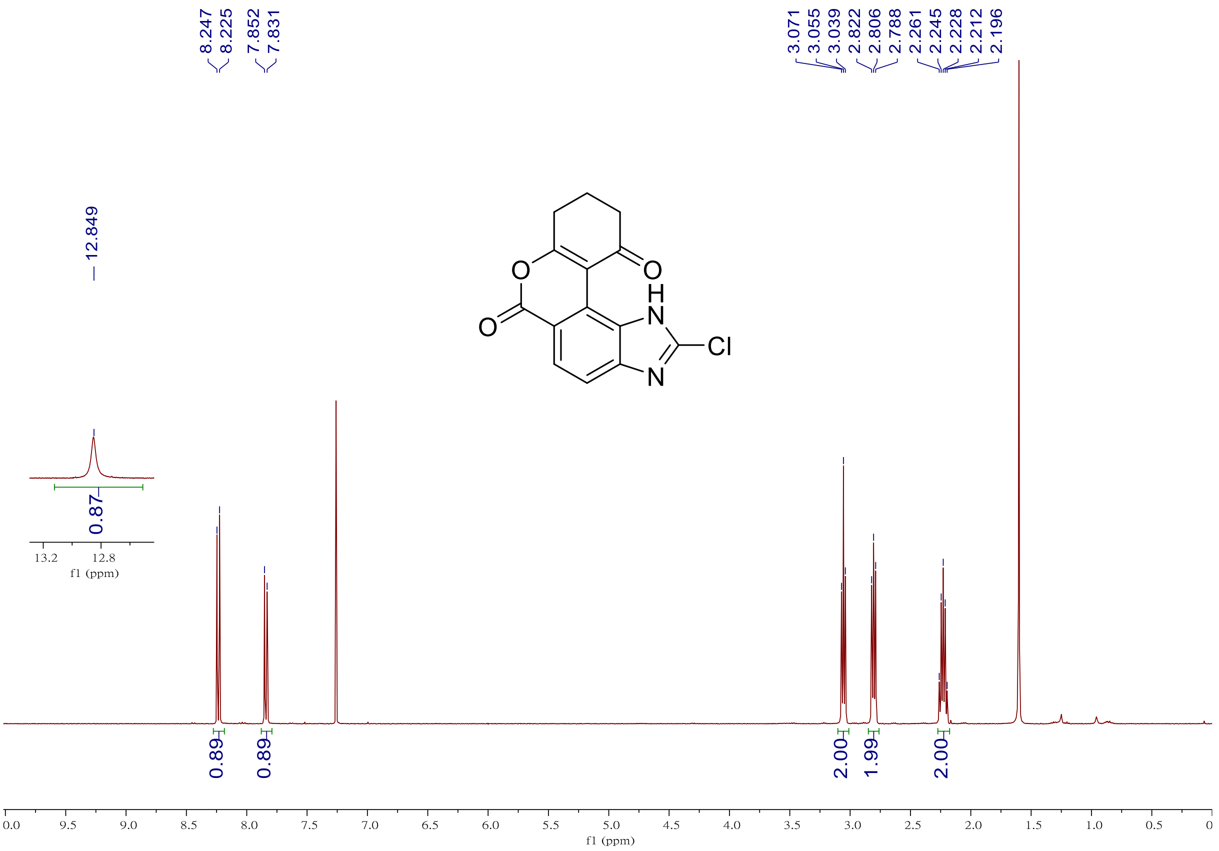


**Figure S131.** $^{1}$H NMR spectrum of compound **3j** (400 MHz, $CDCl_3$).

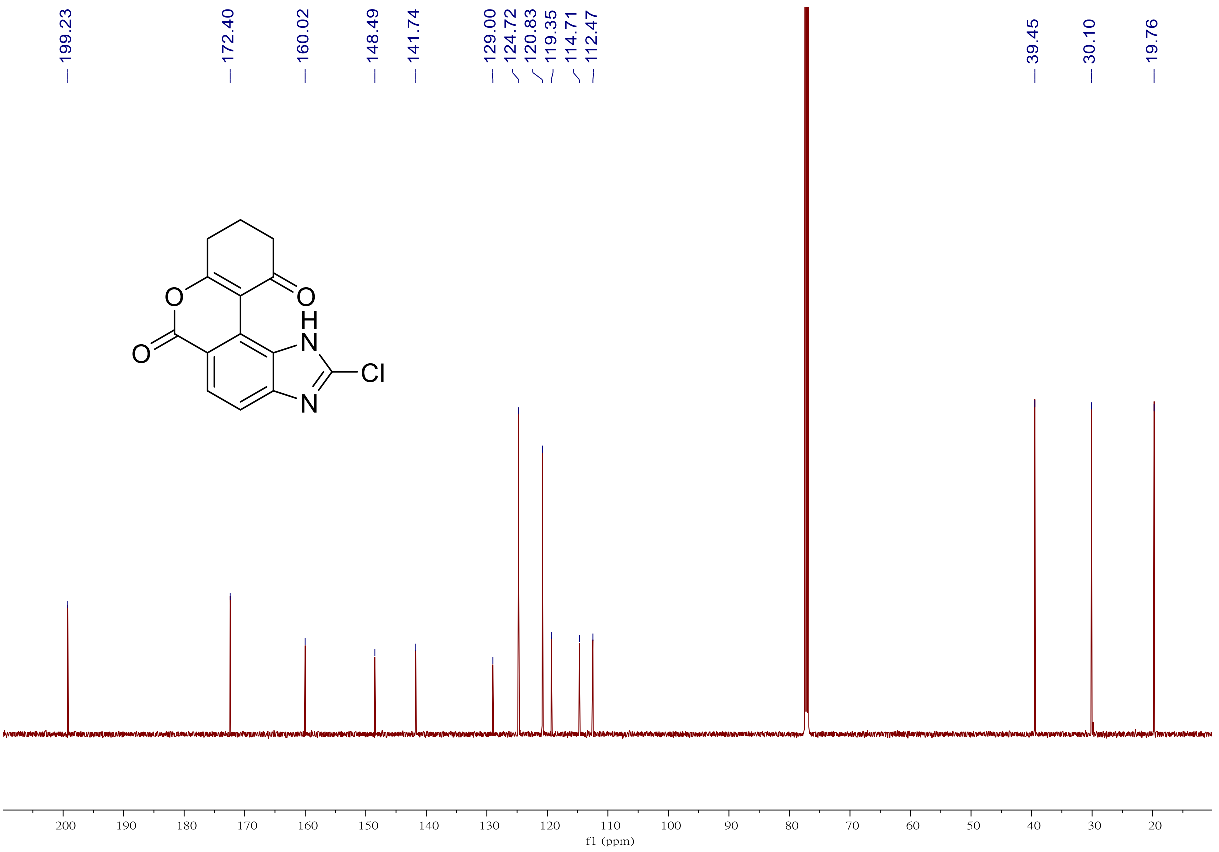


**Figure S132.** $^{13}C\{^{1}H\}$ NMR spectrum of compound **3j** (151 MHz, $CDCl_3$).

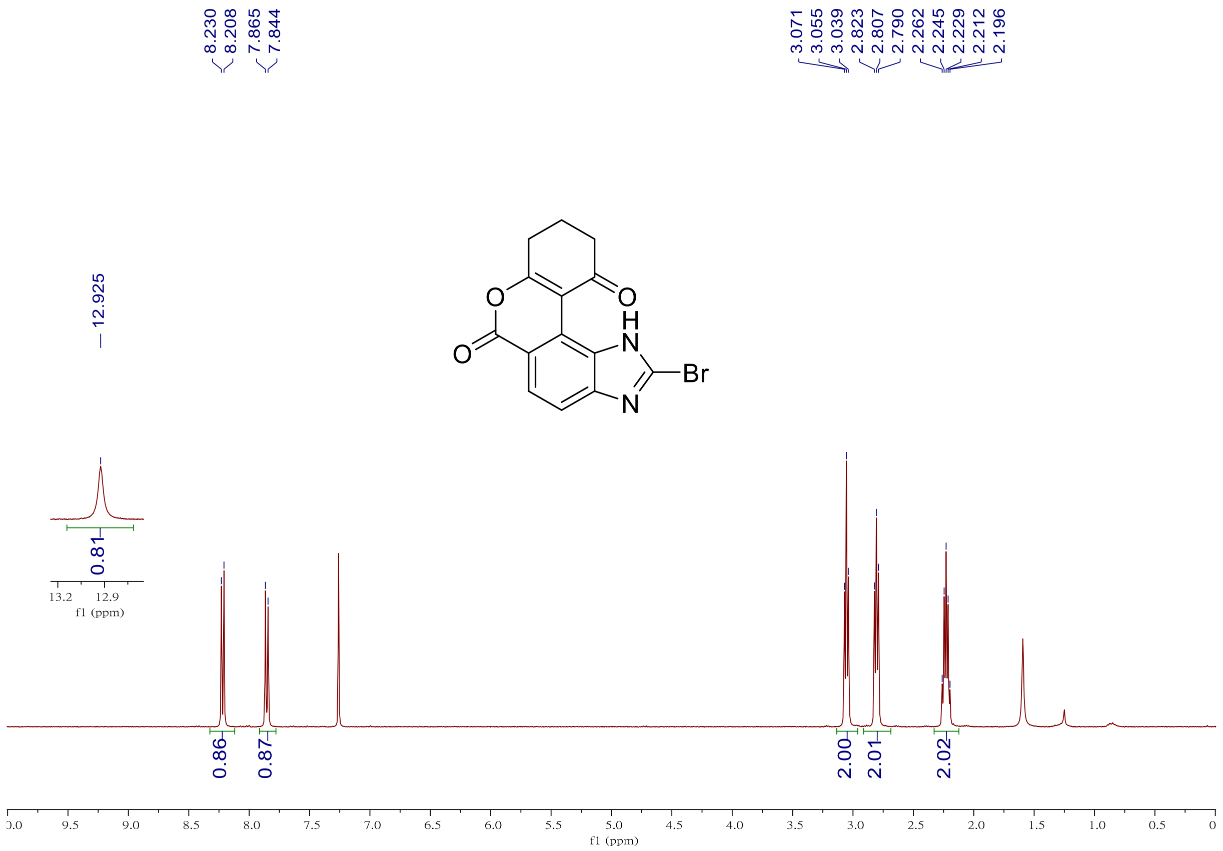


**Figure S133.** $^{1}H$ NMR spectrum of compound **3k** (400 MHz, $CDCl_3$).

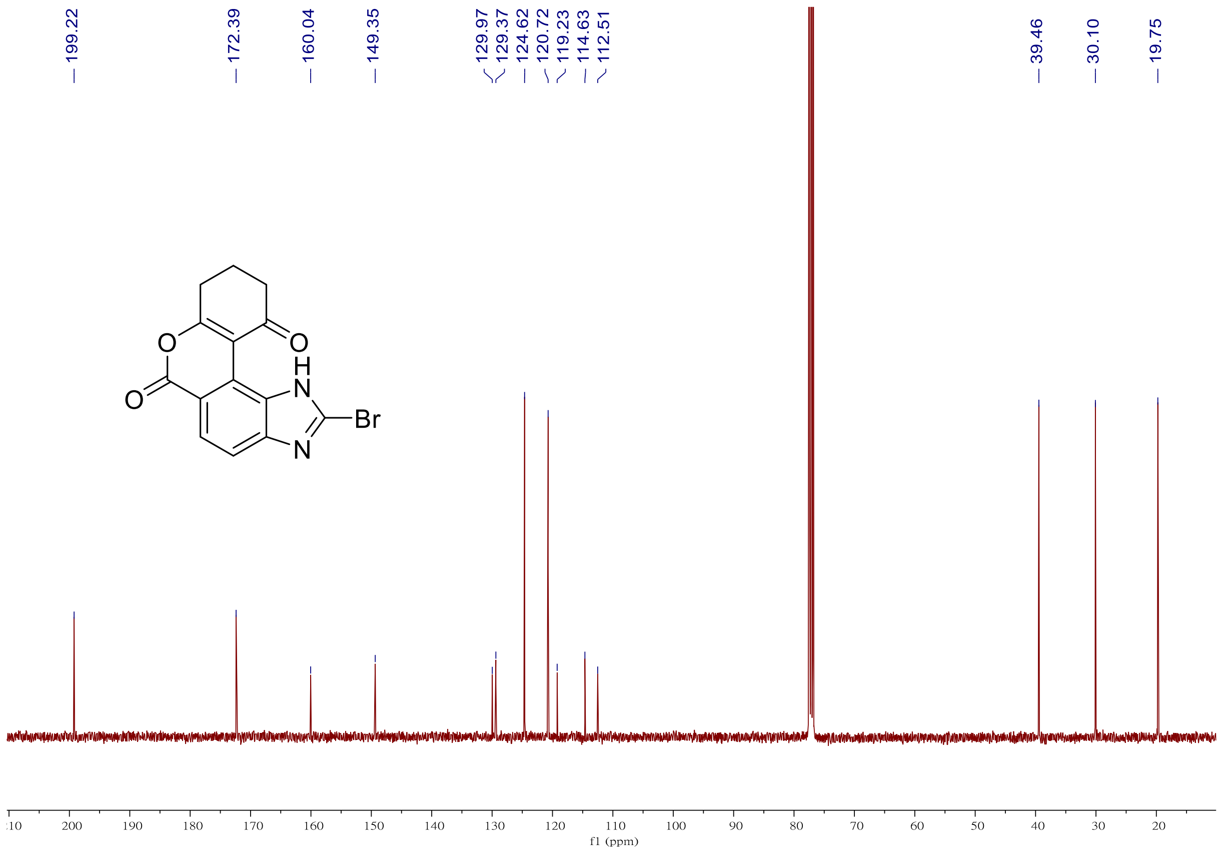


**Figure S134.** $^{13}C\{^{1}H\}$ NMR spectrum of compound **3k** (101 MHz, $CDCl_3$).

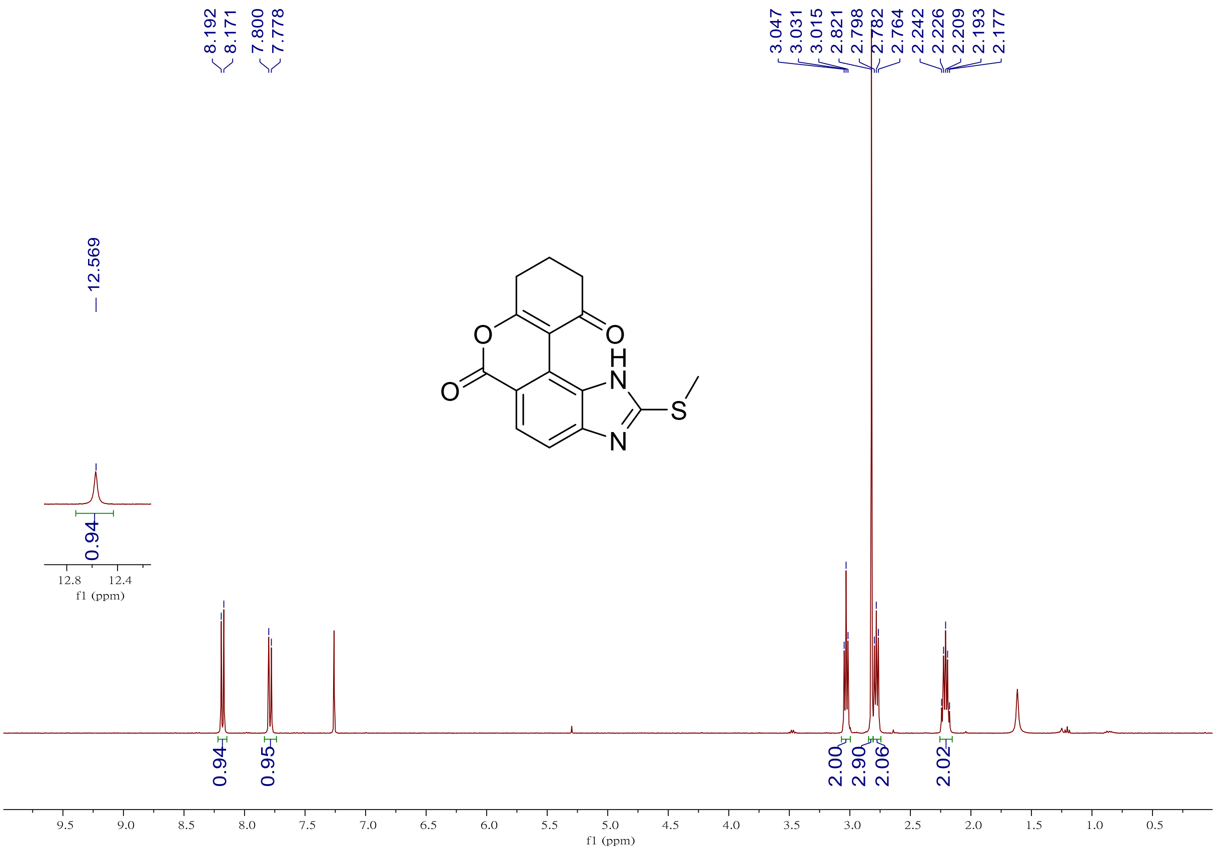

**Figure S135.** $^{1}$H NMR spectrum of compound **3l** (400 MHz, $CDCl_3$).

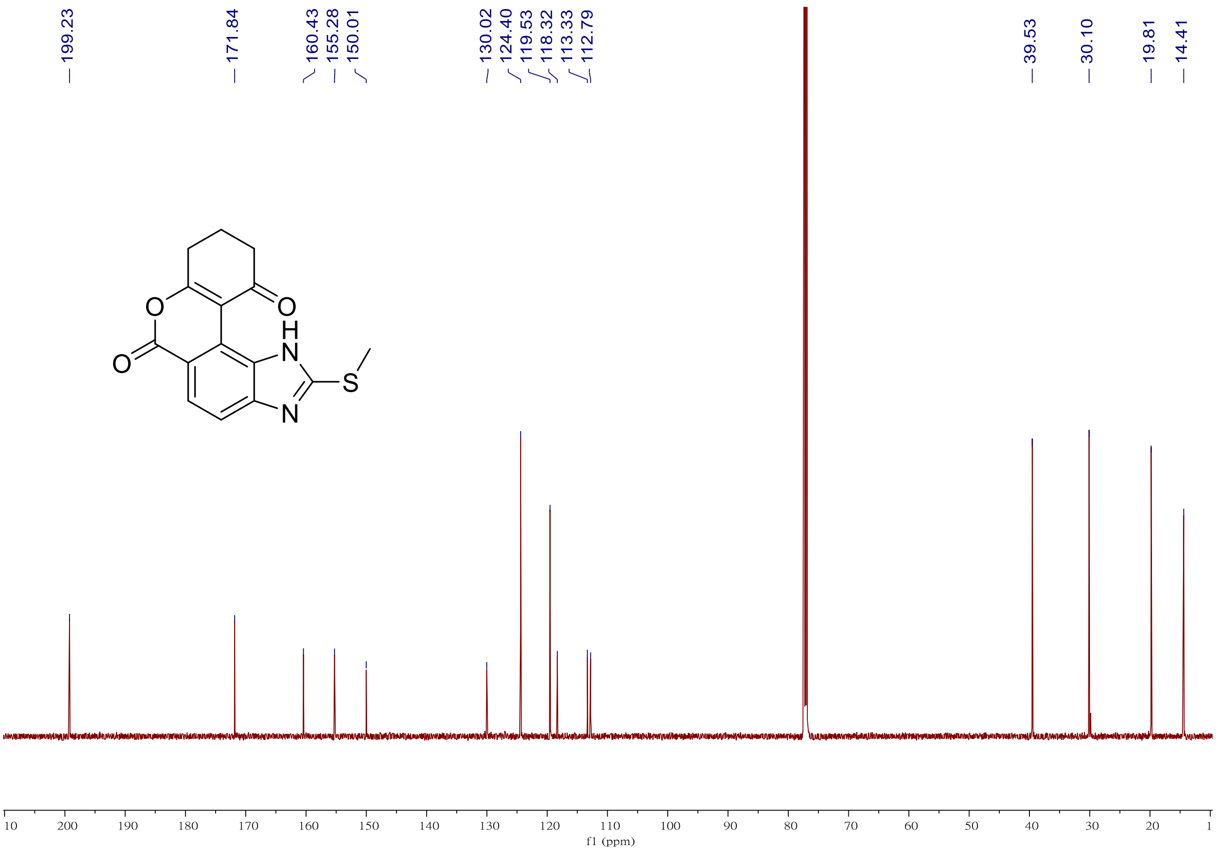

**Figure S136.** $^{13}C\{^{1}H\}$ NMR spectrum of compound **3l** (151 MHz, $CDCl_3$).

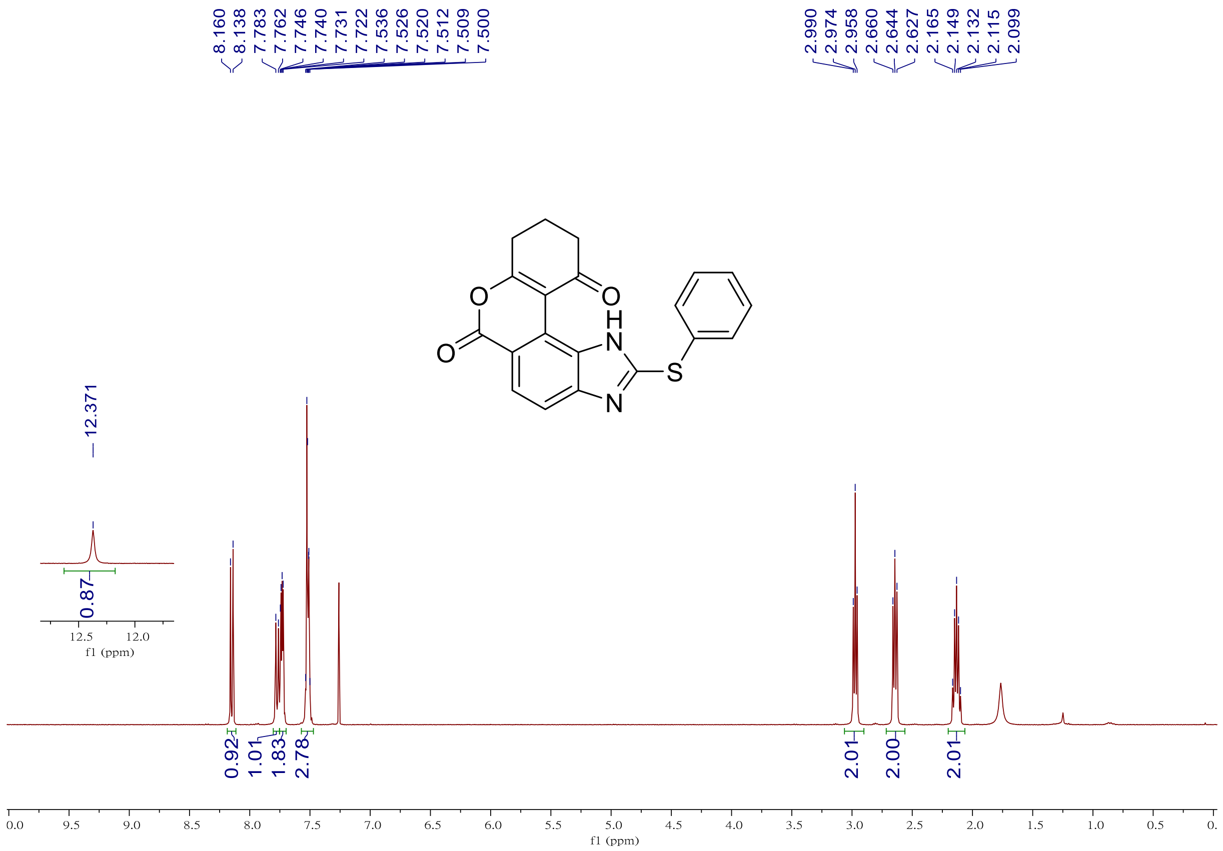

**Figure S137.** $^{1}$H NMR spectrum of compound **3m** (400 MHz, $CDCl_3$).

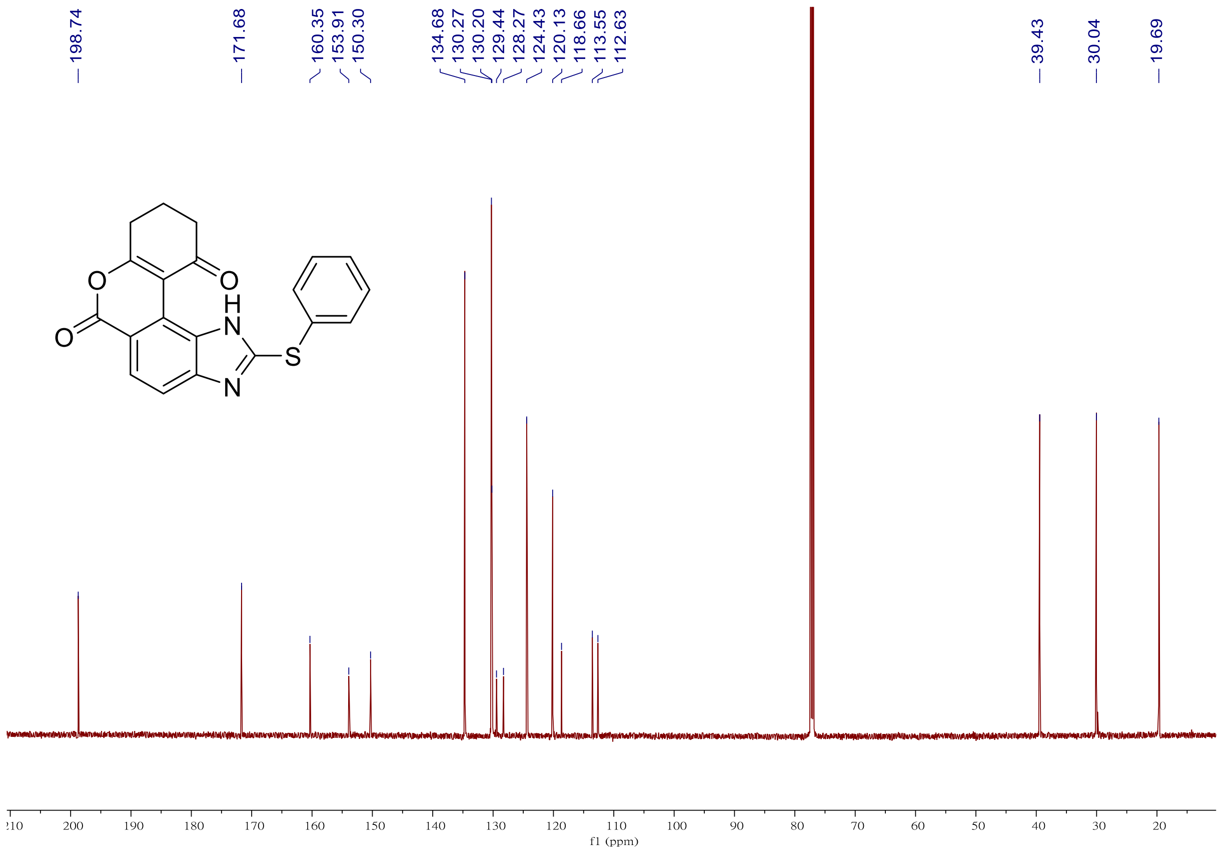

**Figure S138.** $^{13}C\{^{1}H\}$ NMR spectrum of compound **3m** (151 MHz, $CDCl_3$).

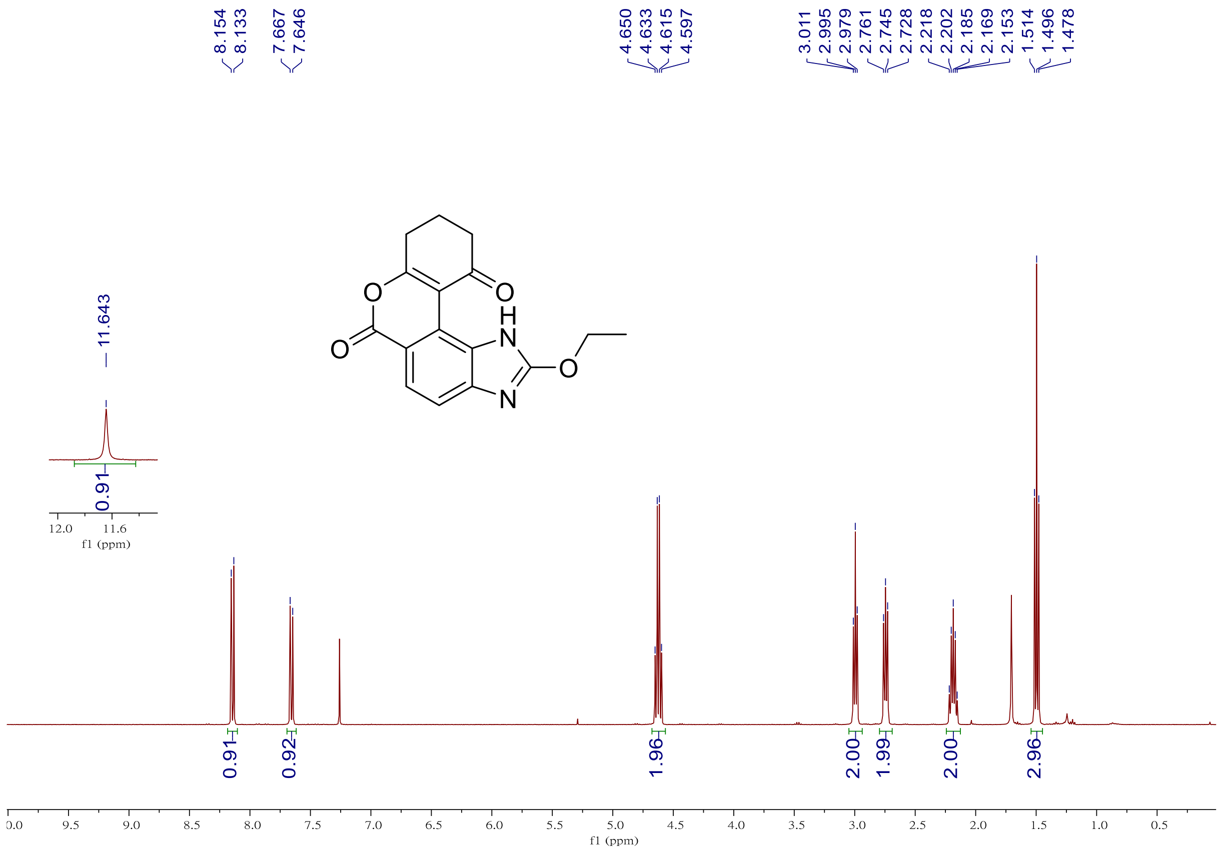

**Figure S139.** $^{1}H$ NMR spectrum of compound **3n** (400 MHz, $CDCl_3$).

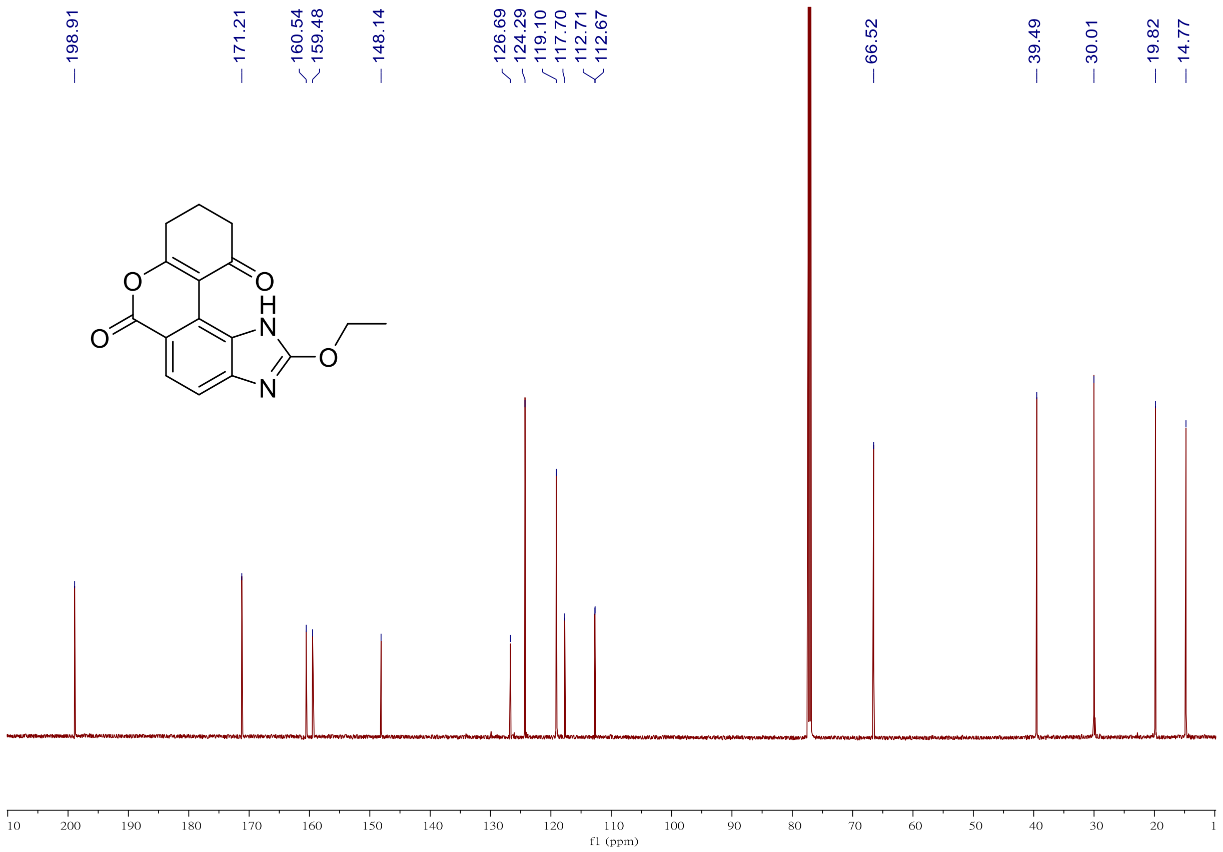

**Figure S140.** $^{13}C\{^{1}H\}$ NMR spectrum of compound **3n** (151 MHz, $CDCl_3$).

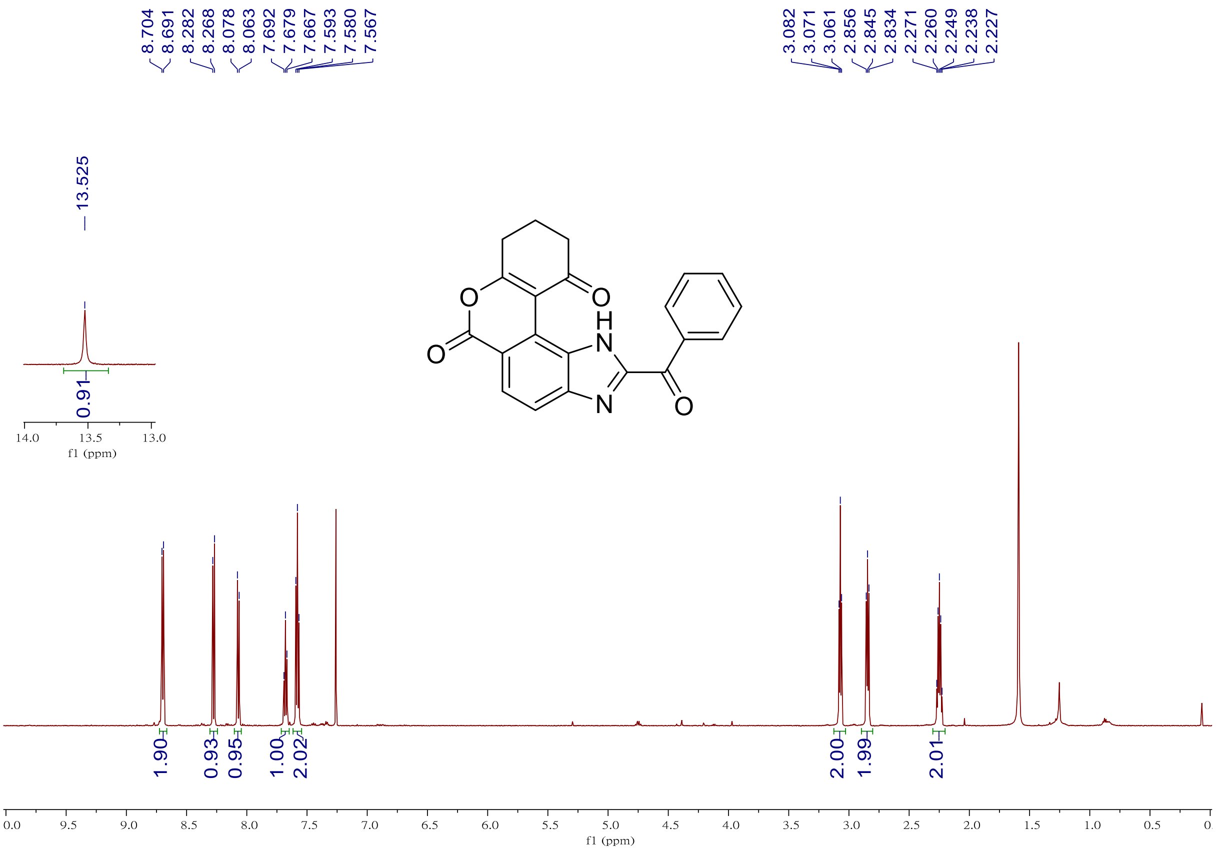


**Figure S141.** $^{1}$H NMR spectrum of compound **3o** (600 MHz, $CDCl_3$).

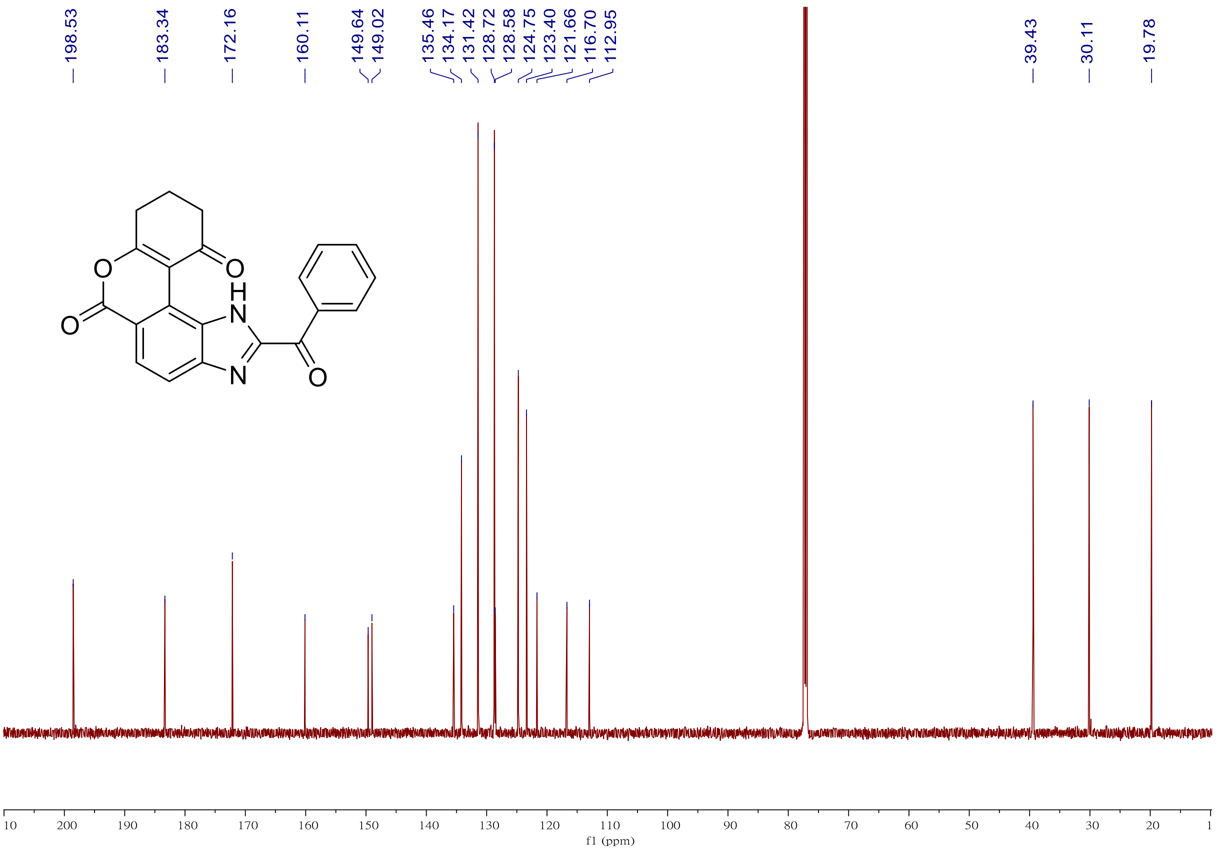


**Figure S142.** $^{13}C\{^{1}H\}$ NMR spectrum of compound **3o** (151 MHz, $CDCl_3$).

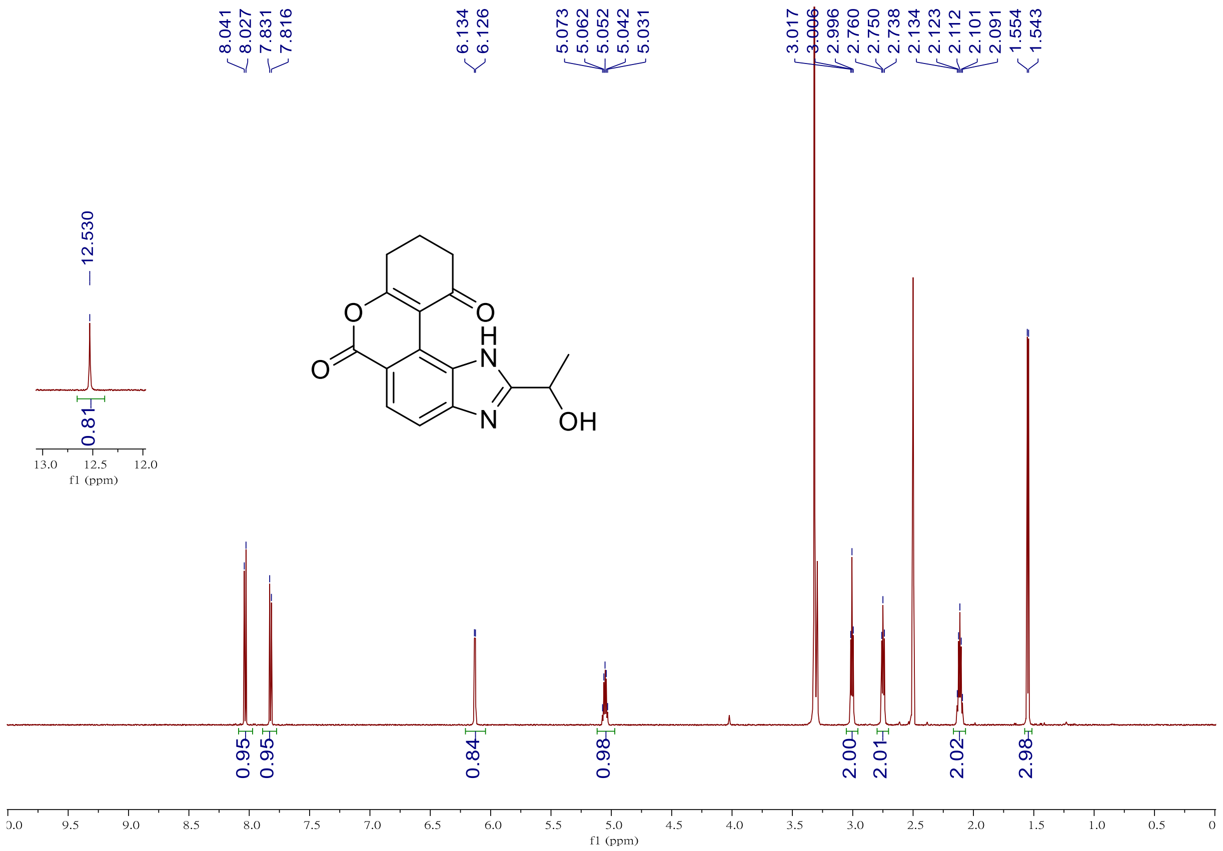

**Figure S143.** $^{1}H$ NMR spectrum of compound **3p** (600 MHz, $(CD_3)_2SO$).

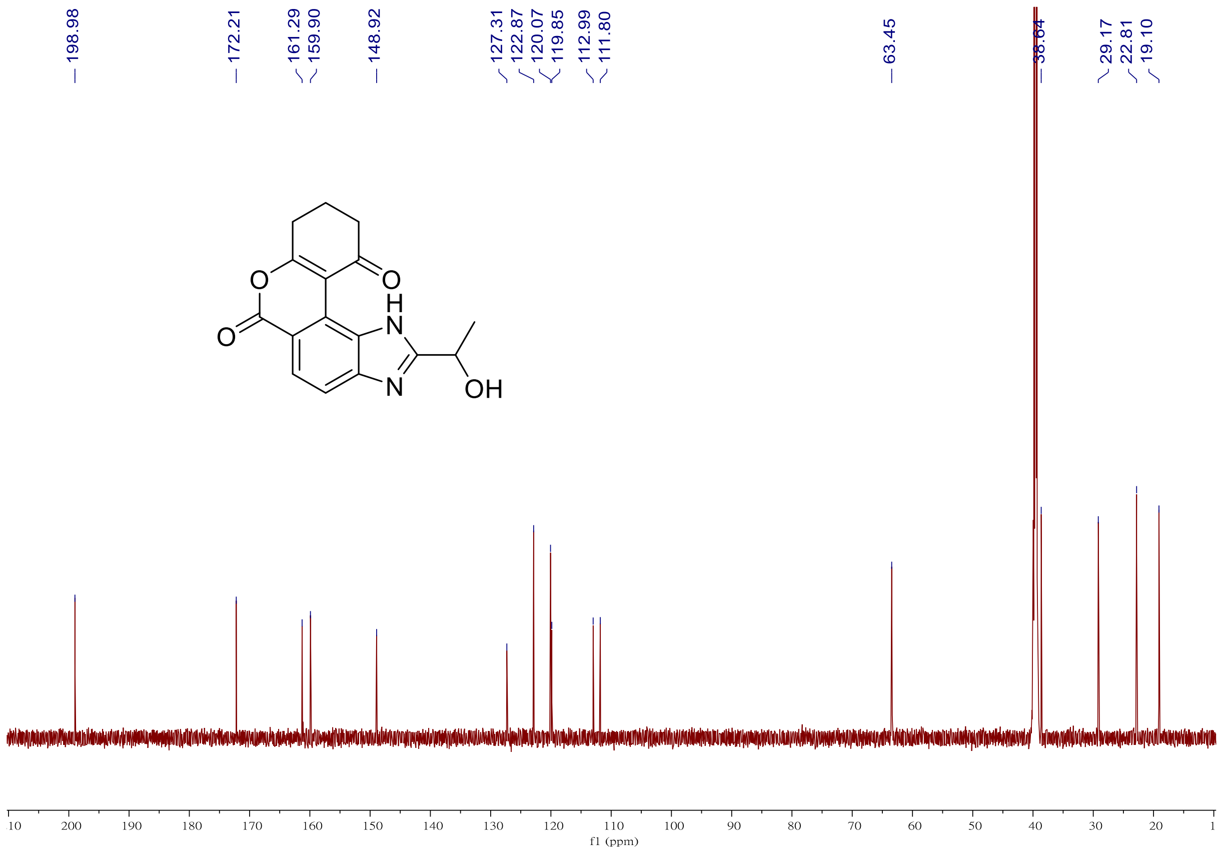

**Figure S144.** $^{13}C\{^{1}H\}$ NMR spectrum of compound **3p** (151 MHz, $(CD_3)_2SO$).

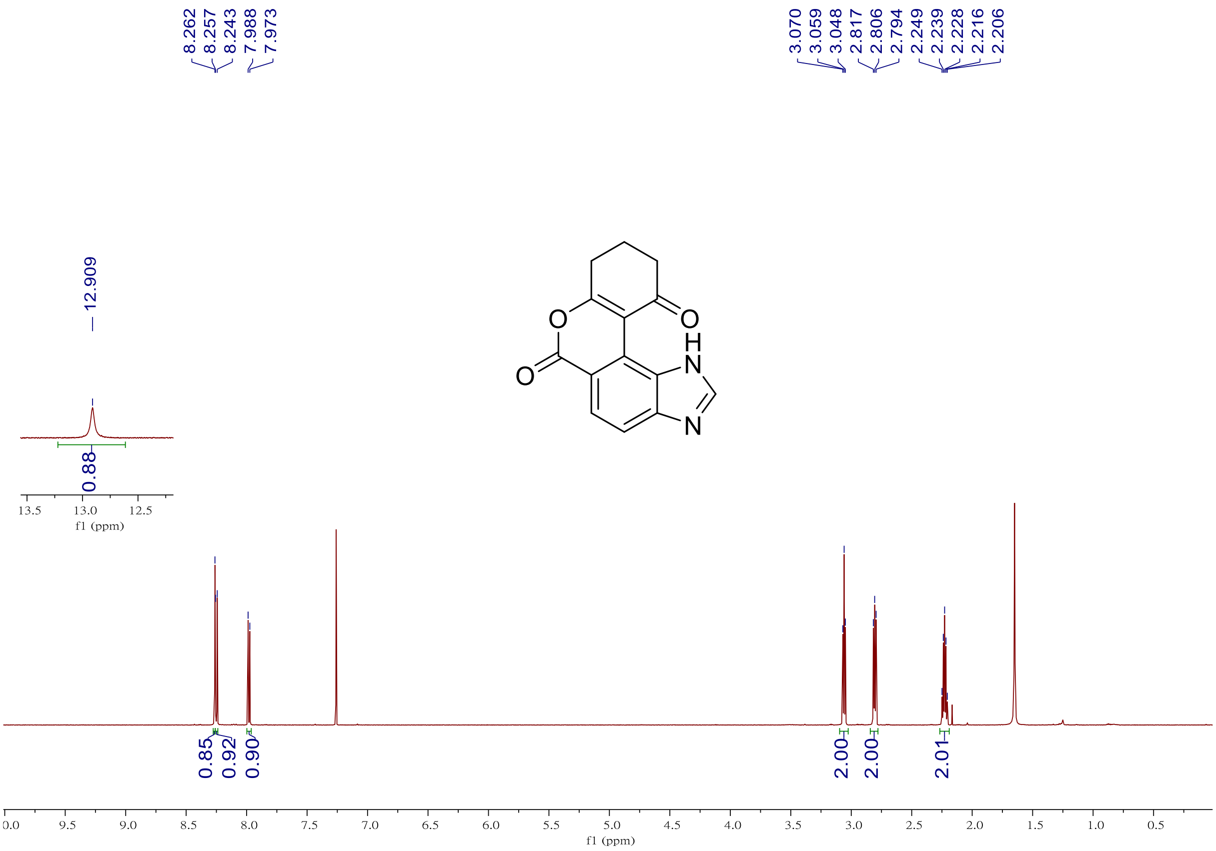


**Figure S145.** $^{1}H$ NMR spectrum of compound **3q** (600 MHz, $CDCl_3$).

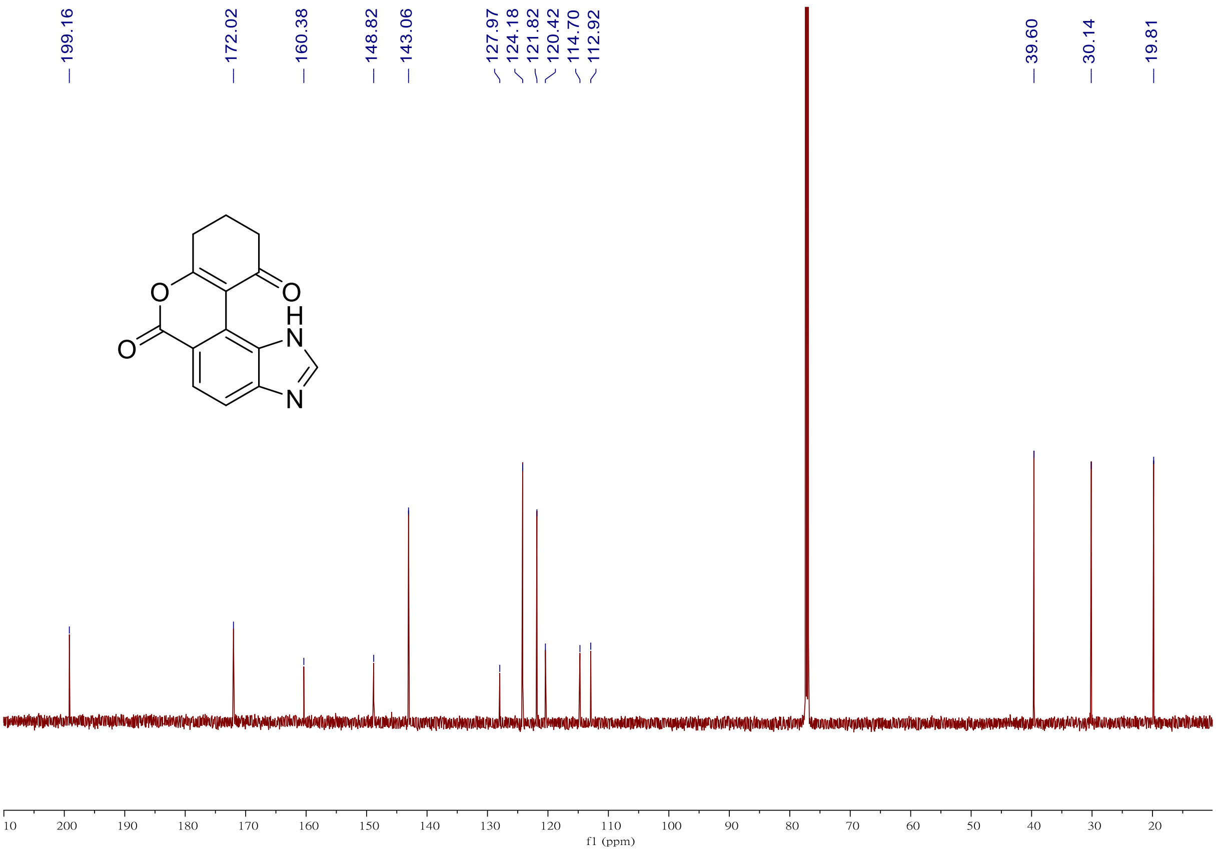


**Figure S146.** $^{13}C\{^{1}H\}$ NMR spectrum of compound **3q** (151 MHz, $CDCl_3$).

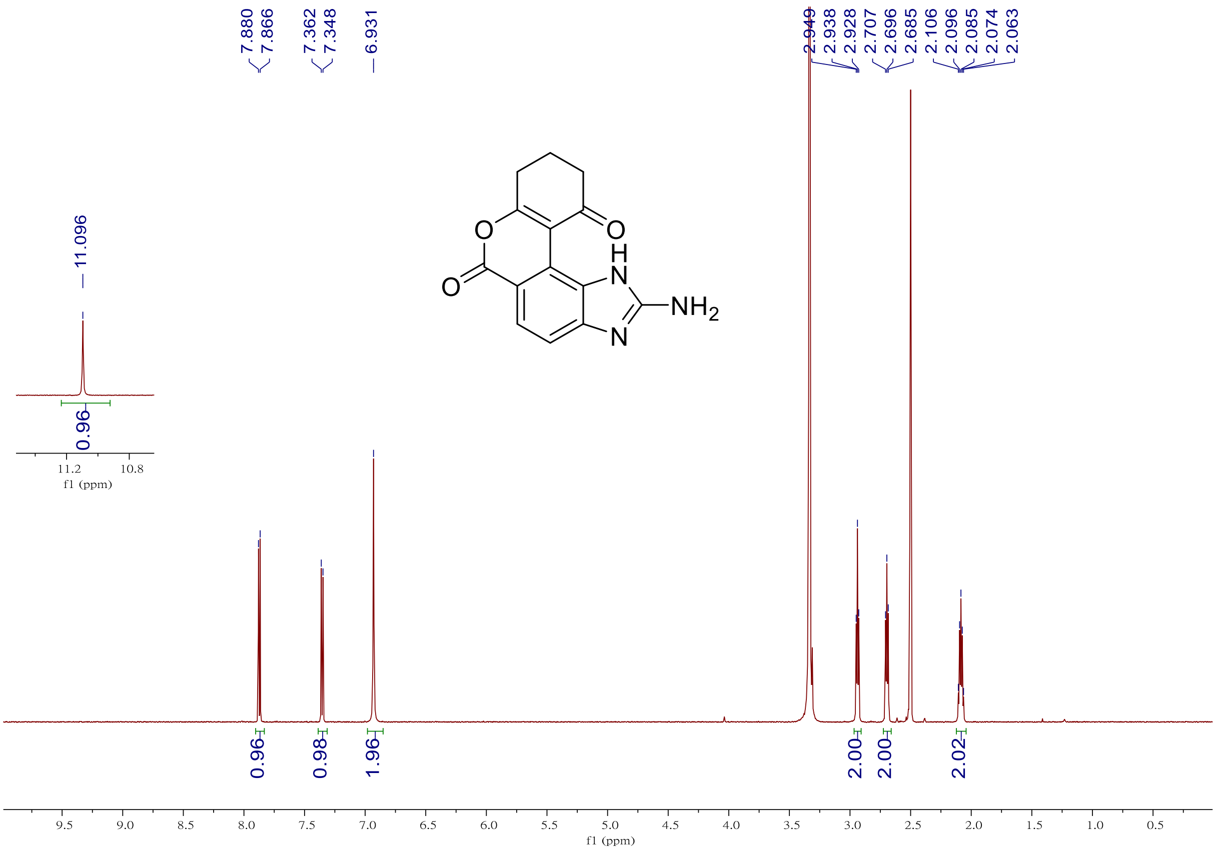


**Figure S147.** $^{1}$H NMR spectrum of compound **3r** (600 MHz, $(CD_3)_2SO$).

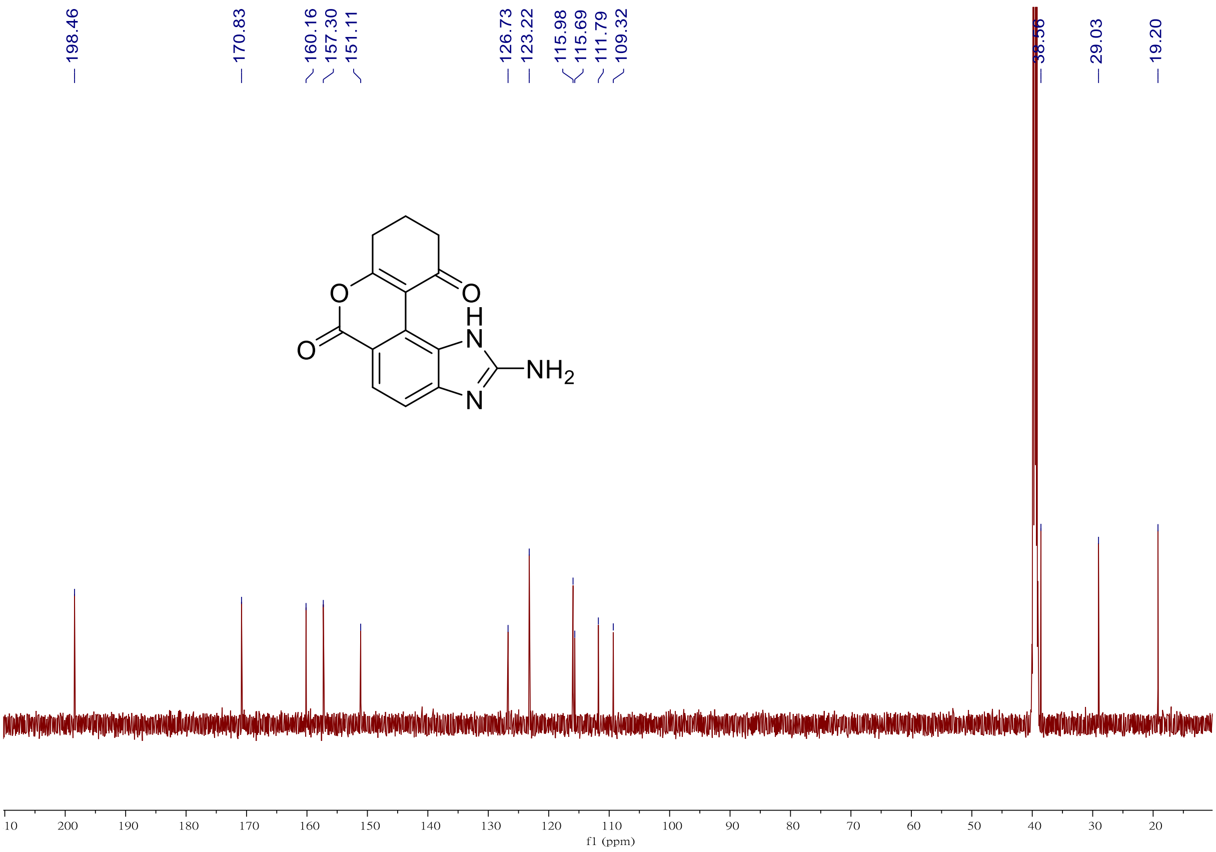


**Figure S148.** $^{13}C\{^{1}H\}$ NMR spectrum of compound **3r** (151 MHz, $(CD_3)_2SO$).

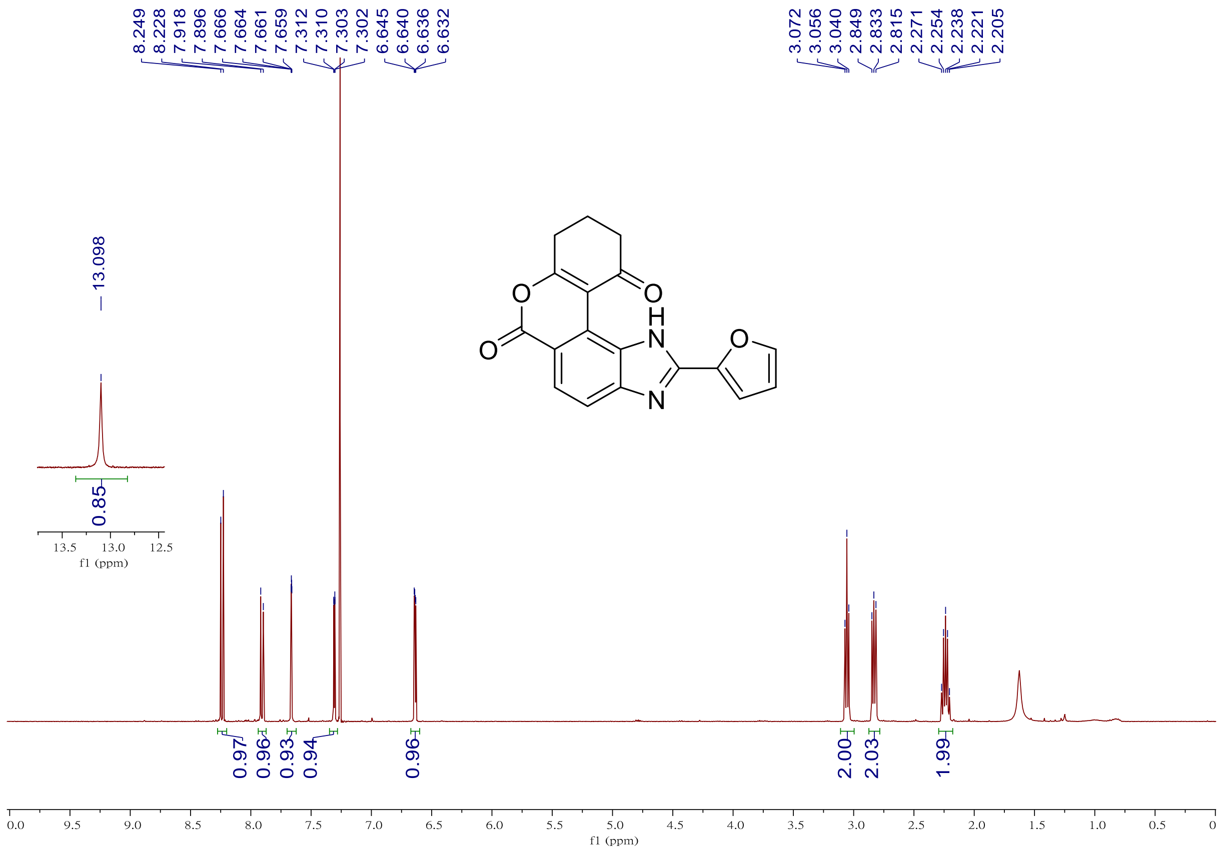


**Figure S149.** $^{1}H$ NMR spectrum of compound **3s** (400 MHz, $CDCl_3$).

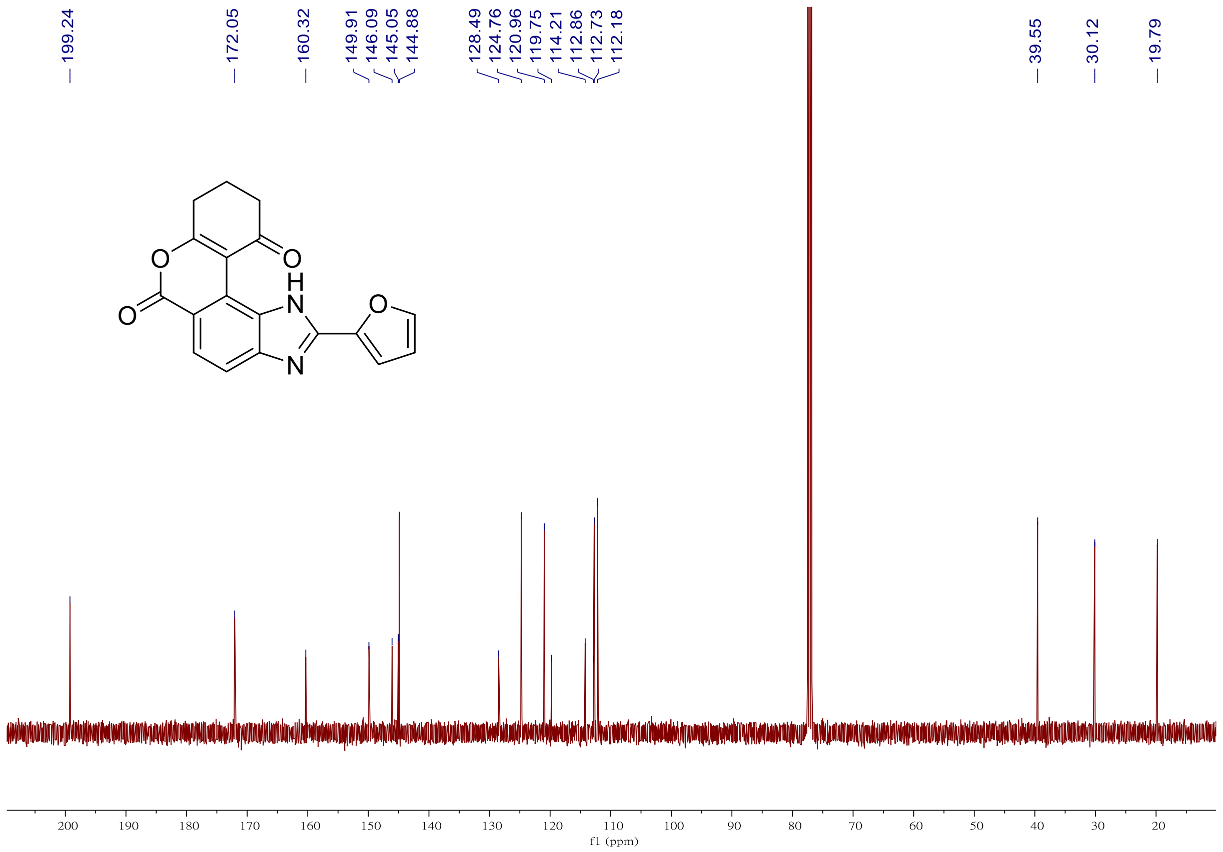


**Figure S150.** $^{13}C\{^{1}H\}$ NMR spectrum of compound **3s** (101 MHz, $CDCl_3$).

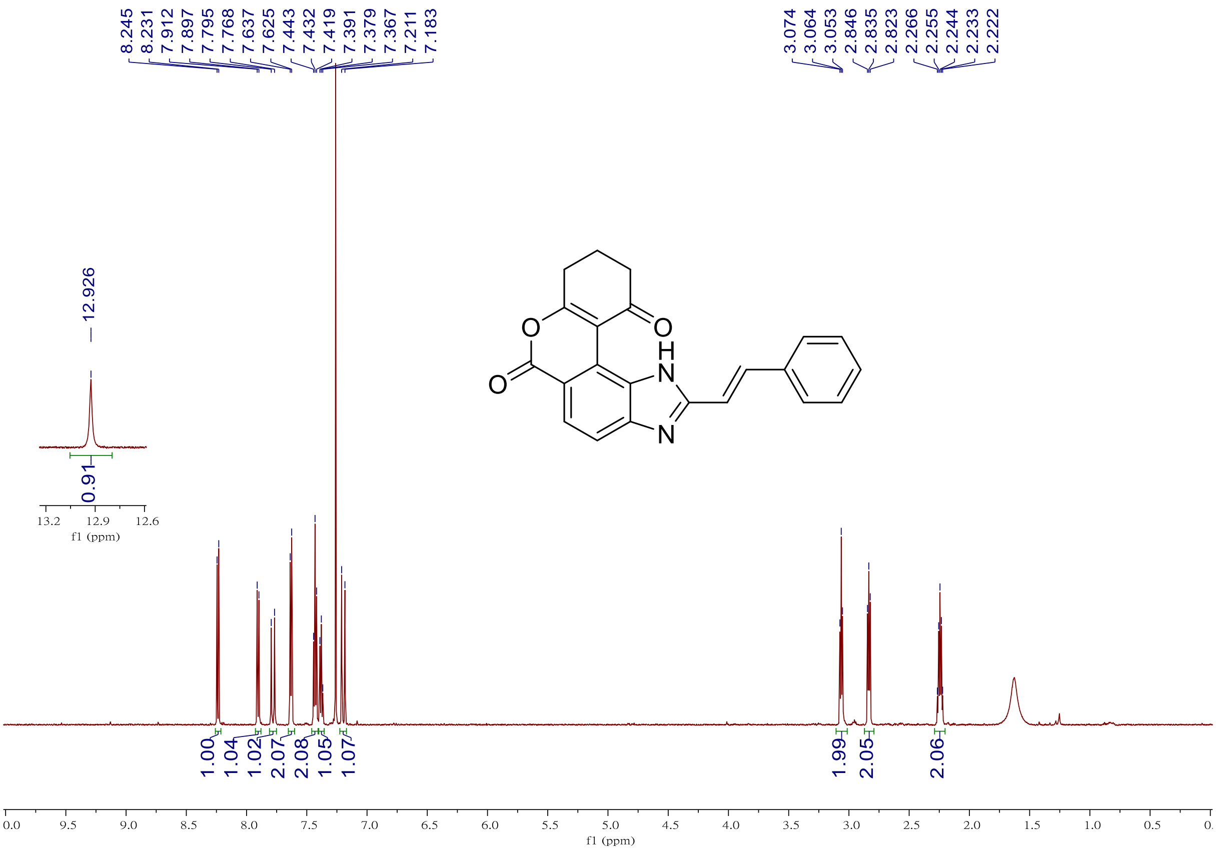


**Figure S151.** $^{1}$H NMR spectrum of compound **3t** (600 MHz, $CDCl_3$).

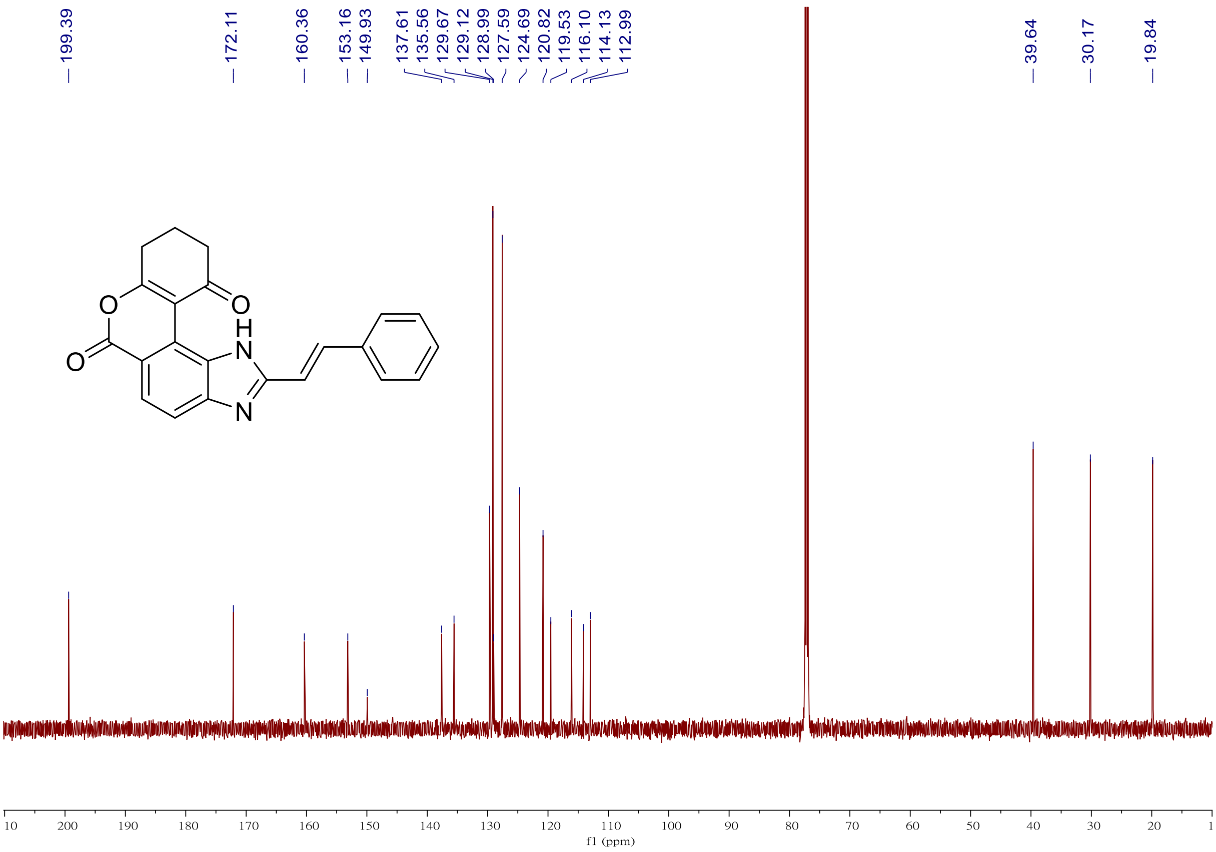


**Figure S152.** $^{13}C\{^{1}H\}$ NMR spectrum of compound **3t** (151 MHz, $CDCl_3$).

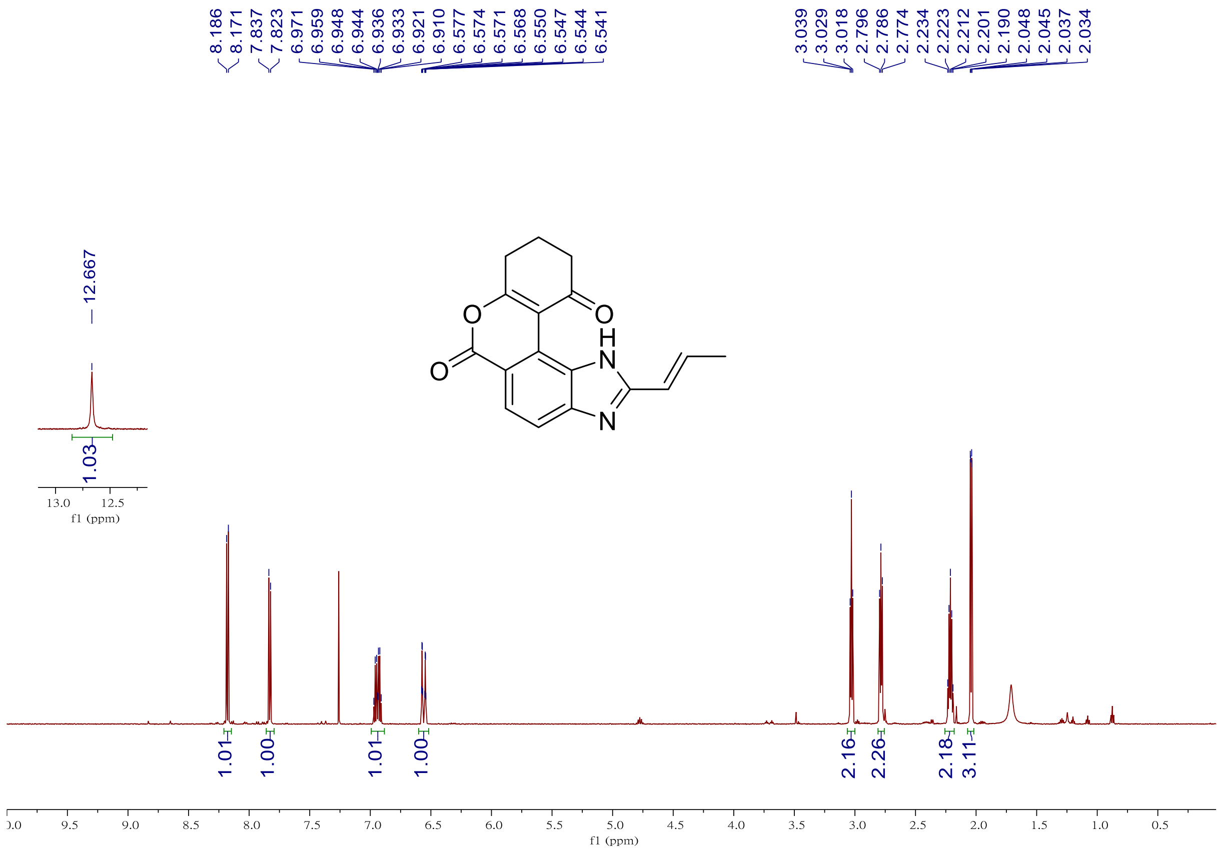


**Figure S153.** $^{1}$H NMR spectrum of compound **3u** (600 MHz, $CDCl_3$).

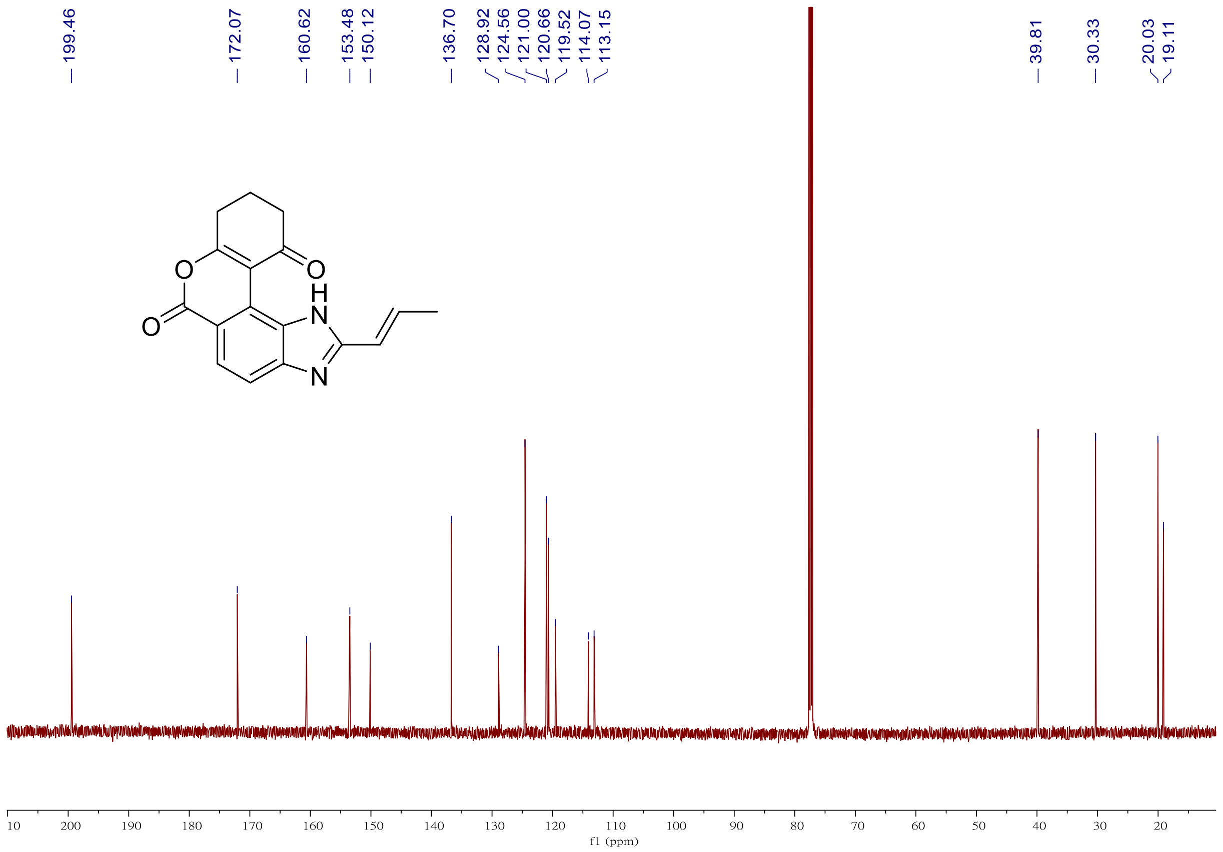


**Figure S154.** $^{13}C\{^{1}H\}$ NMR spectrum of compound **3u** (151 MHz, $CDCl_3$).

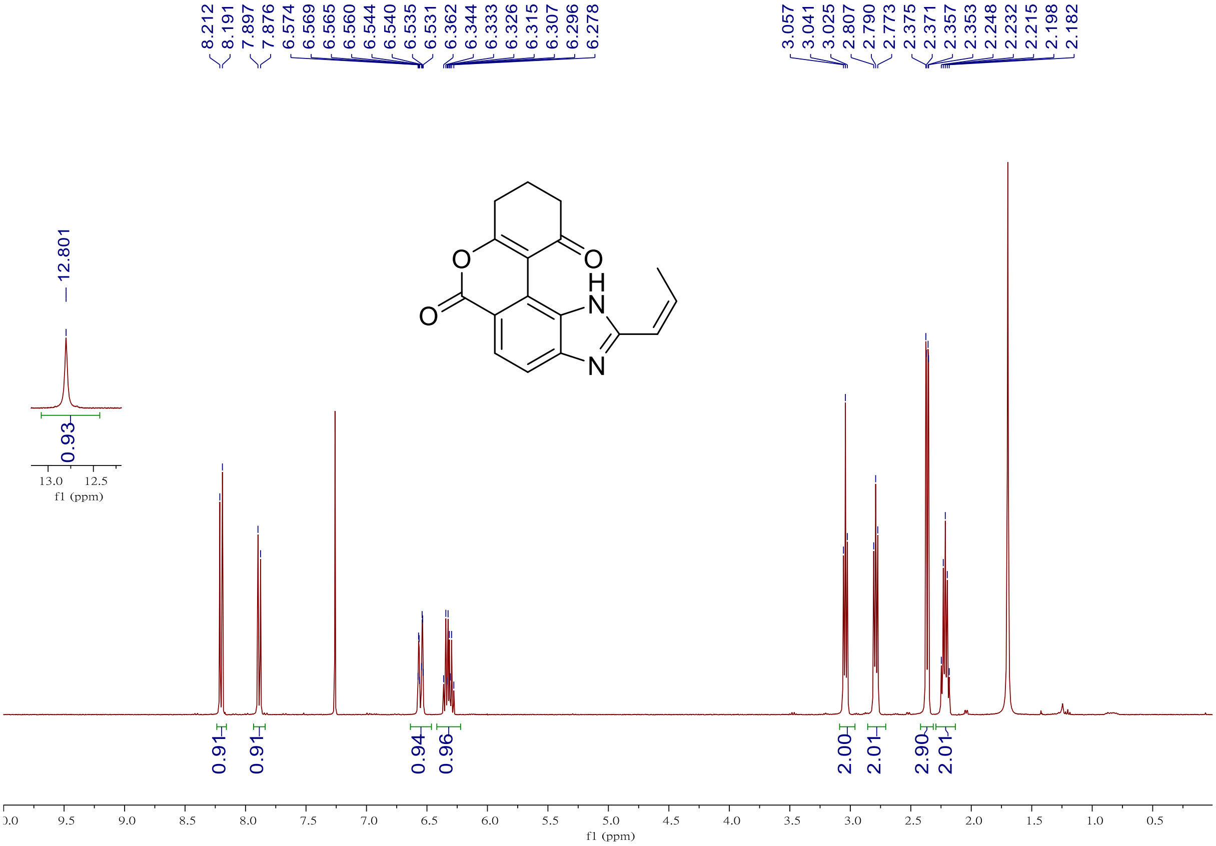


**Figure S155.** $^{1}H$ NMR spectrum of compound **3v** (400 MHz, $CDCl_3$).

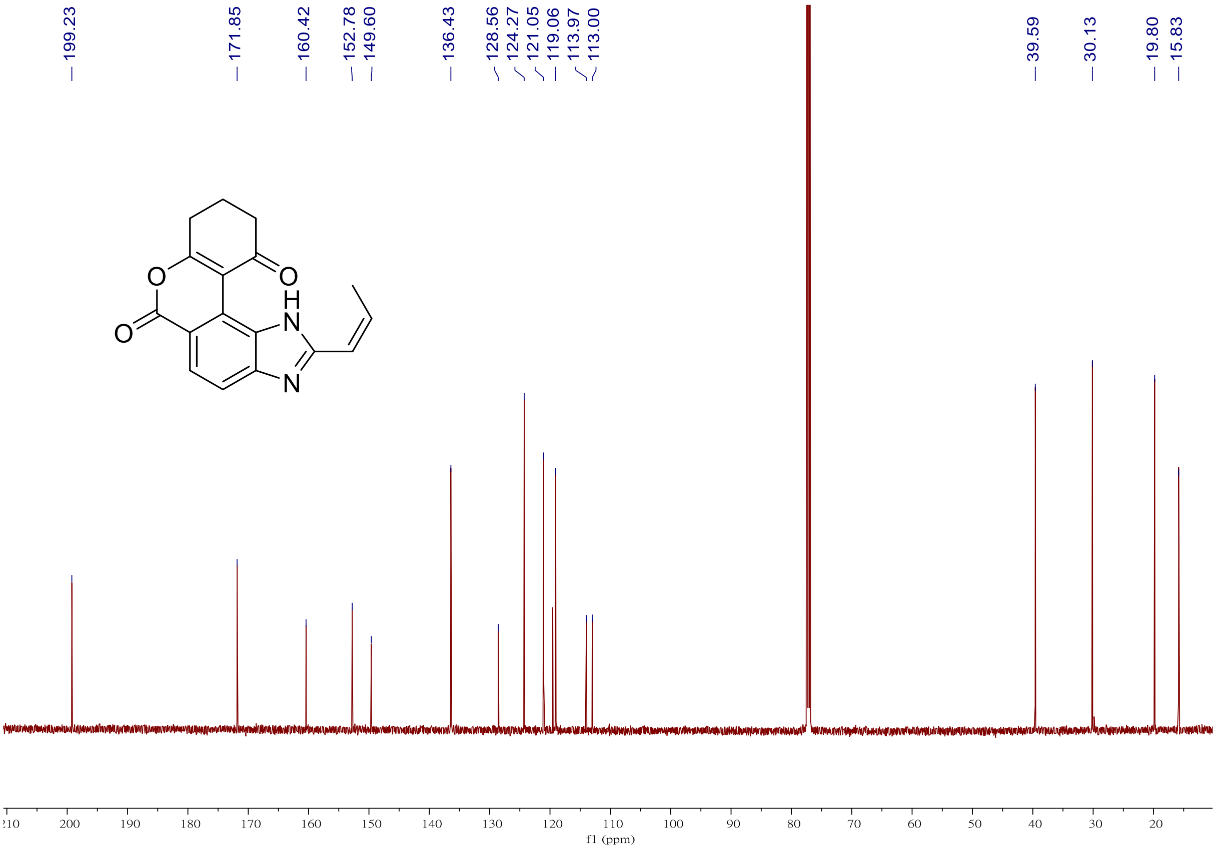


**Figure S156.** $^{13}C\{^{1}H\}$ NMR spectrum of compound **3v** (151 MHz, $CDCl_3$).

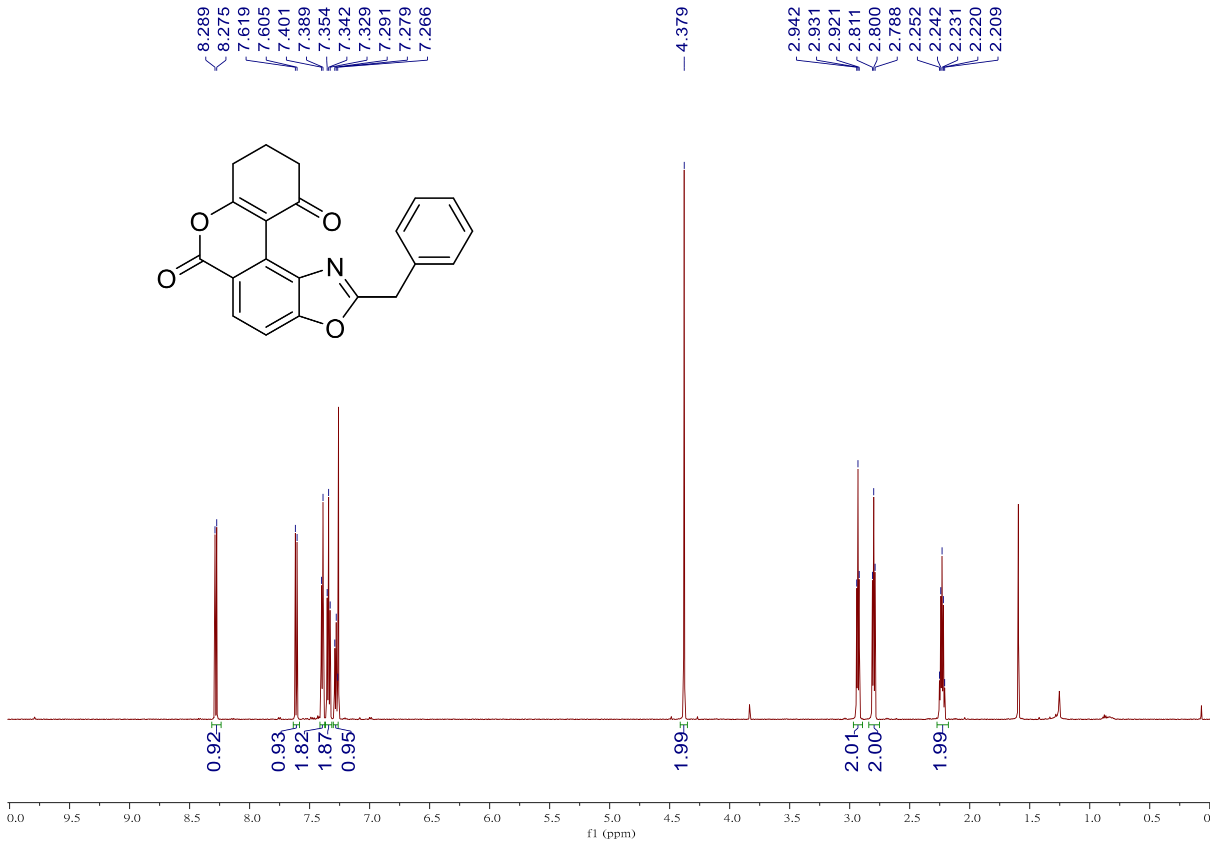

**Figure S157.** $^{1}H$ NMR spectrum of compound **3x** (600 MHz, $CDCl_3$).

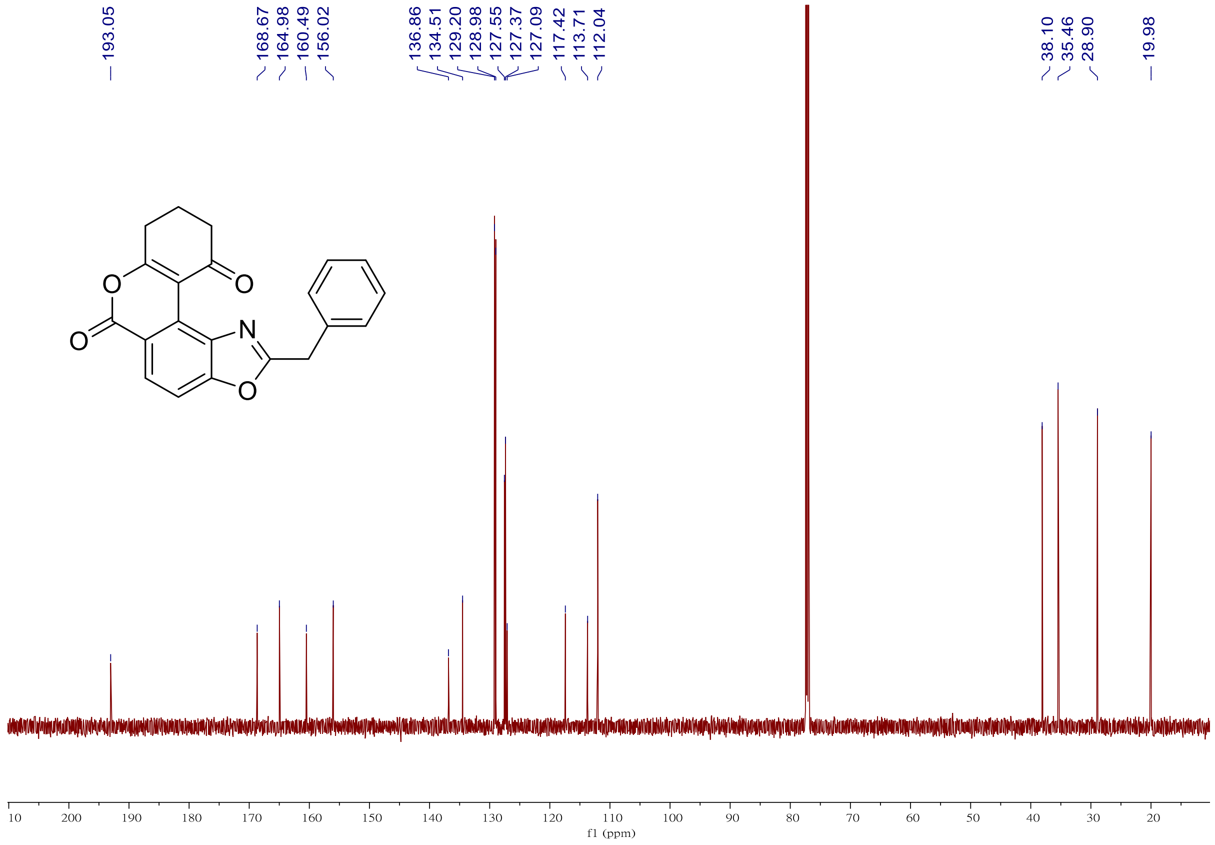

**Figure S158.** $^{13}C\{^{1}H\}$ NMR spectrum of compound **3x** (151 MHz, $CDCl_3$).

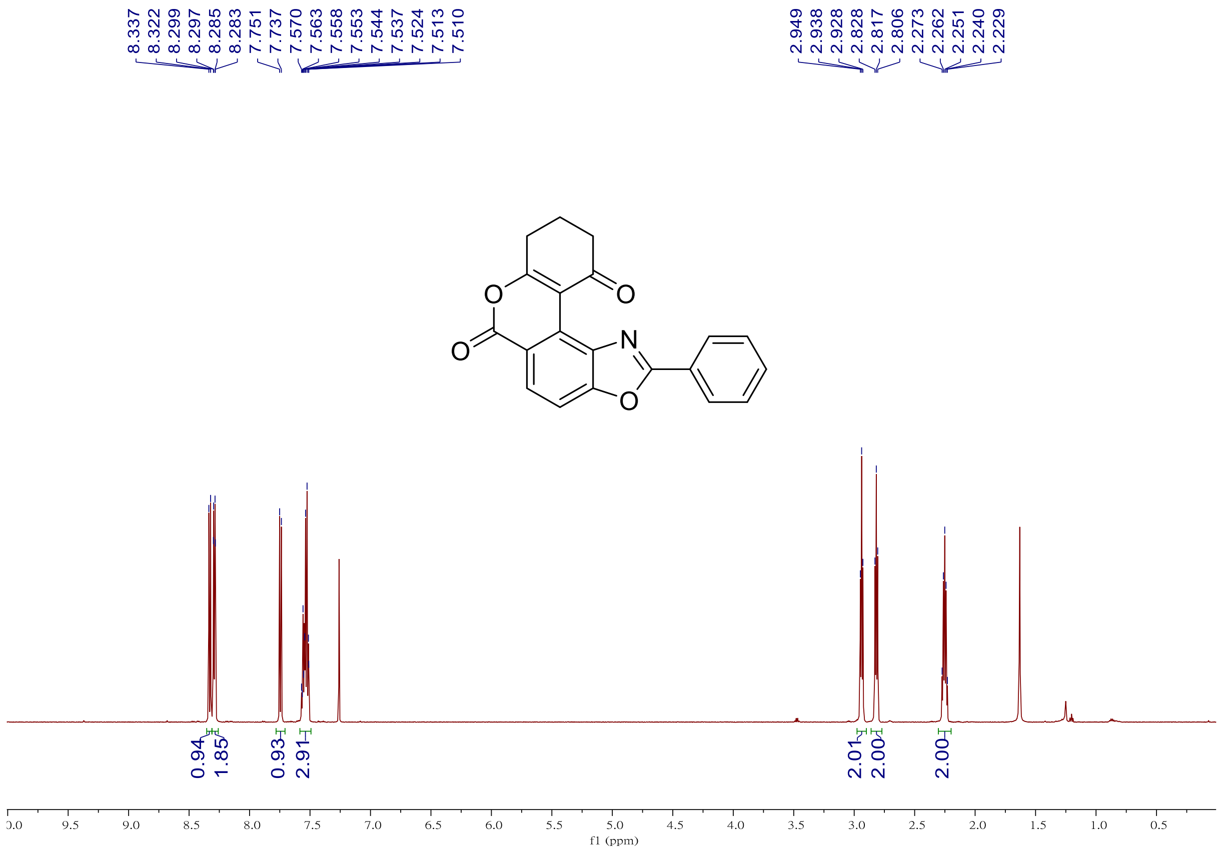


**Figure S159.** $^{1}$H NMR spectrum of compound **3y** (600 MHz, $CDCl_3$).

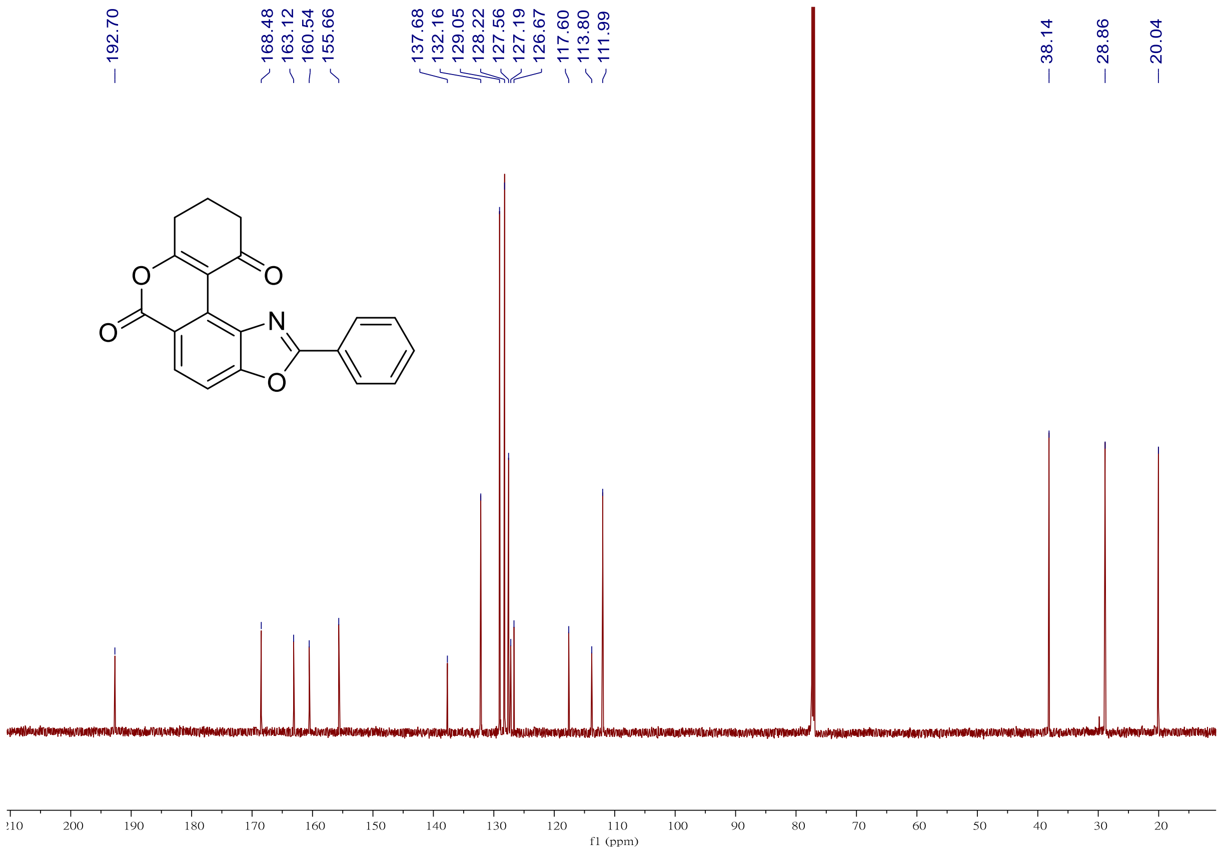


**Figure S160.** $^{13}C\{^{1}H\}$ NMR spectrum of compound **3y** (151 MHz, $CDCl_3$).

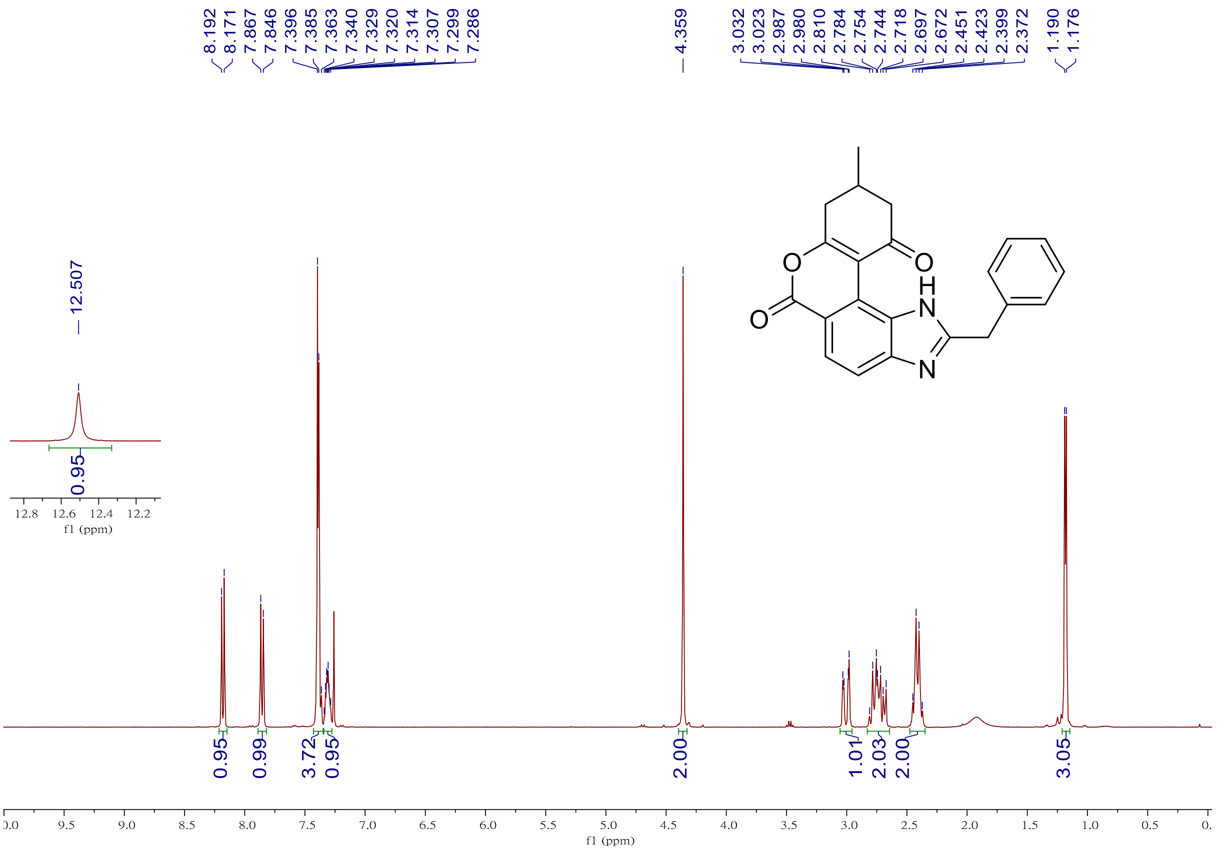


**Figure S161.** $^{1}H$ NMR spectrum of compound **3ab** (400 MHz, $CDCl_3$).

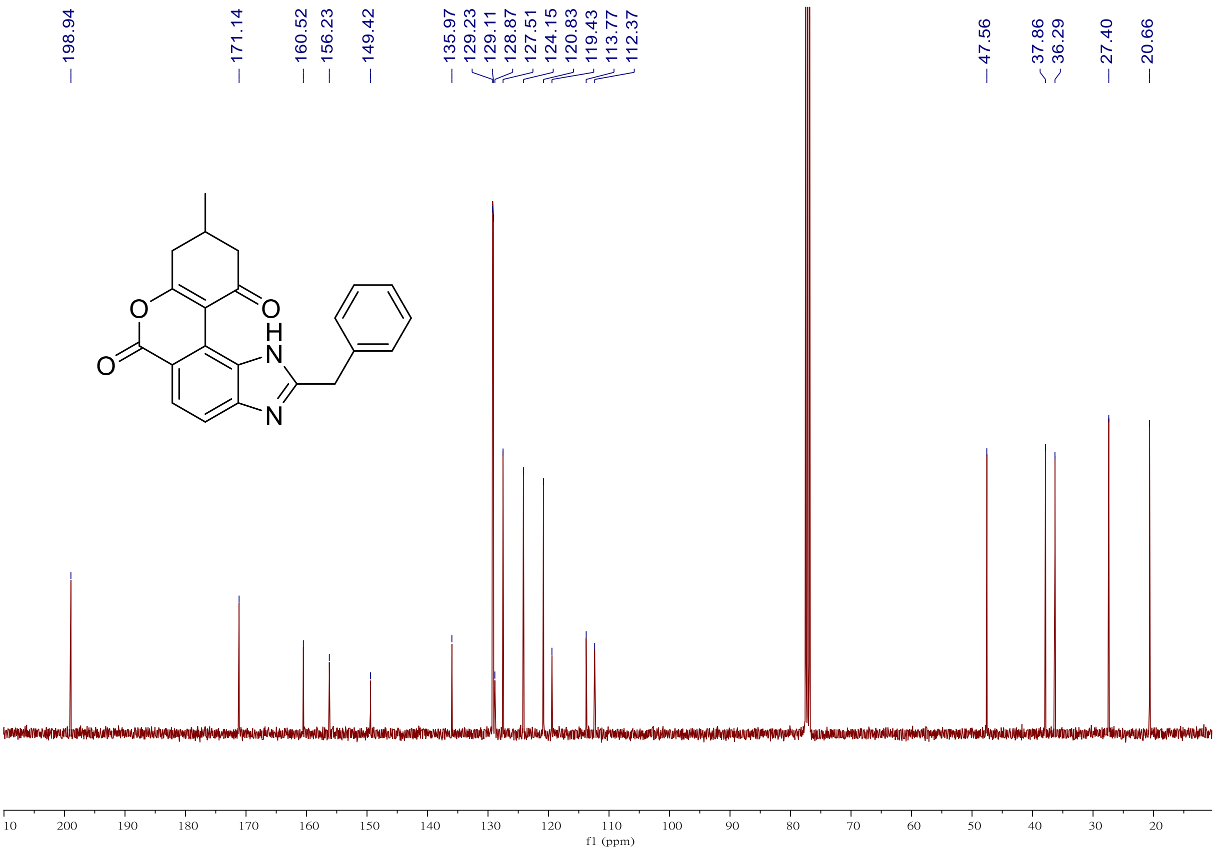


**Figure S162.** $^{13}C\{^{1}H\}$ NMR spectrum of compound **3ab** (101 MHz, $CDCl_3$).

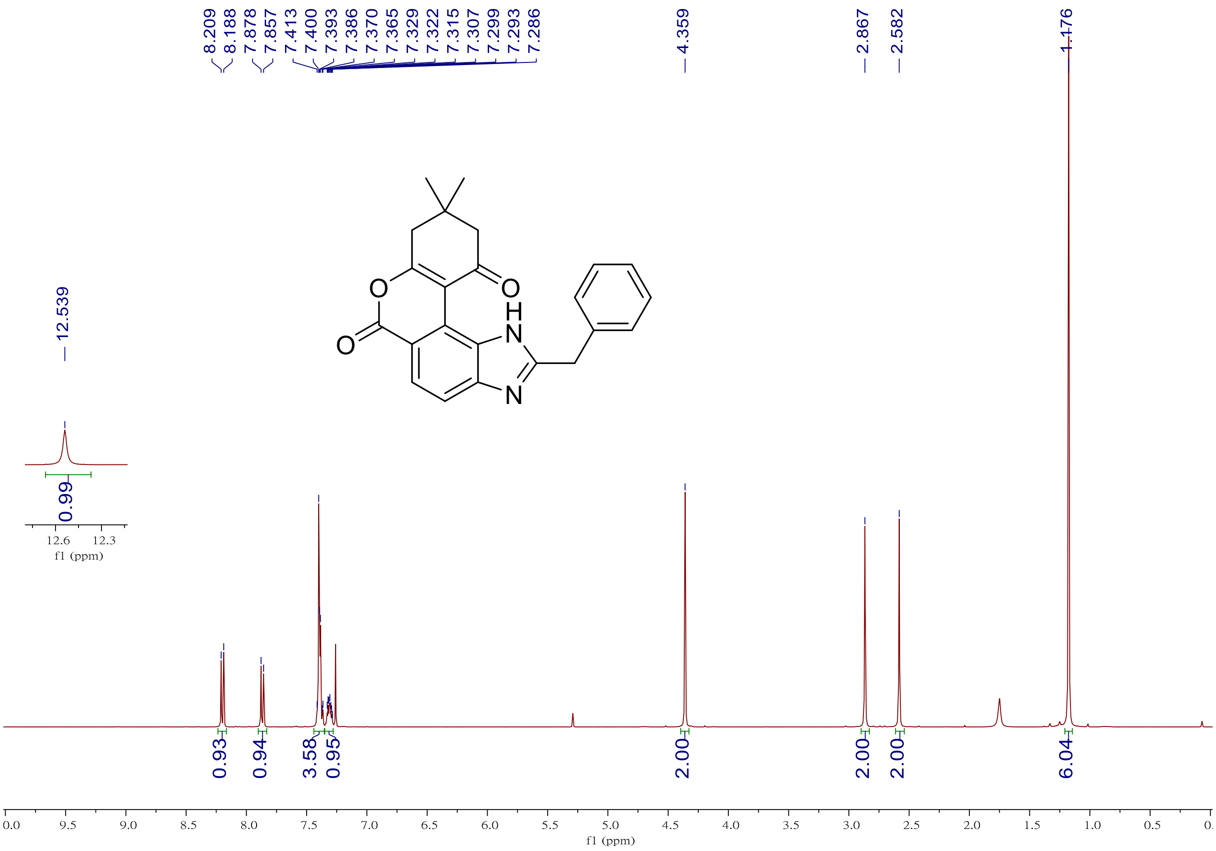


**Figure S163.** $^{1}$H NMR spectrum of compound **3ac** (400 MHz, $CDCl_3$).

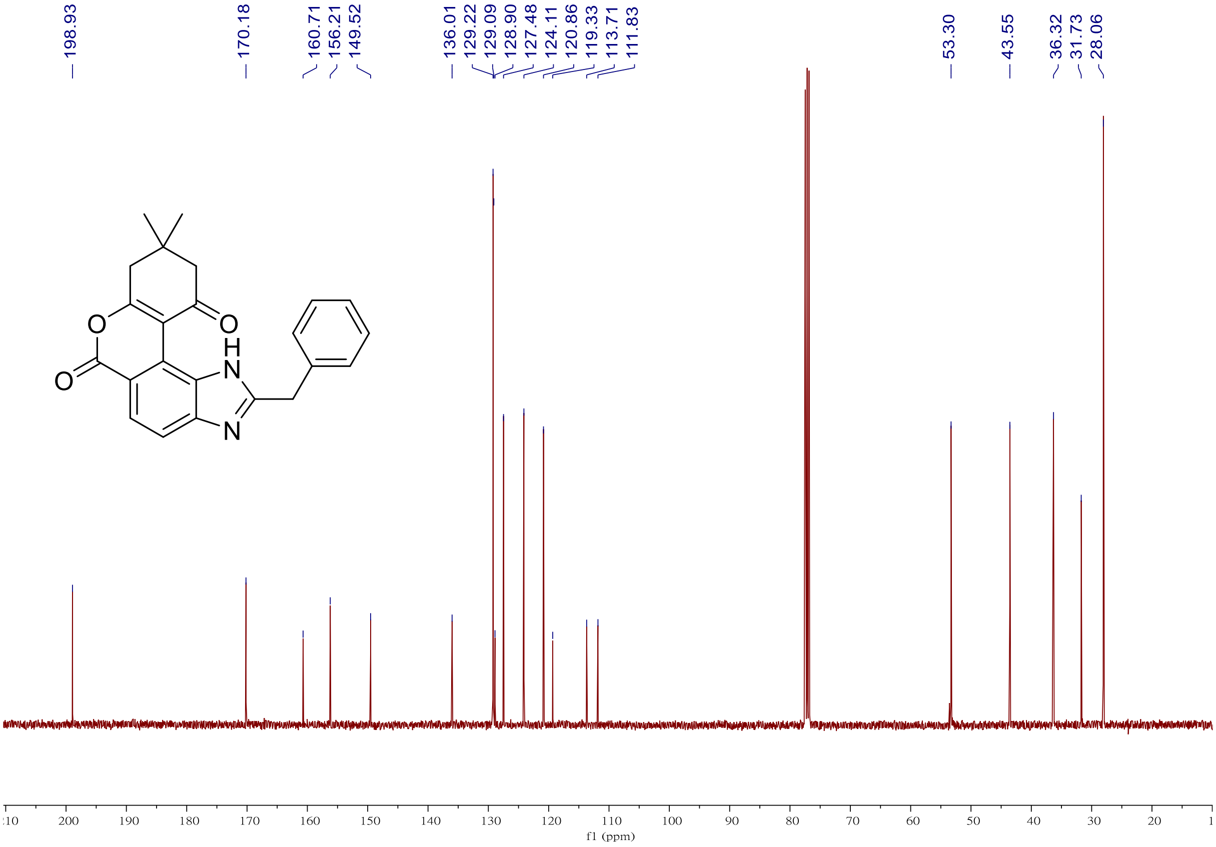


**Figure S164.** $^{13}$C{$^{1}$H} NMR spectrum of compound **3ac** (101 MHz, $CDCl_3$).

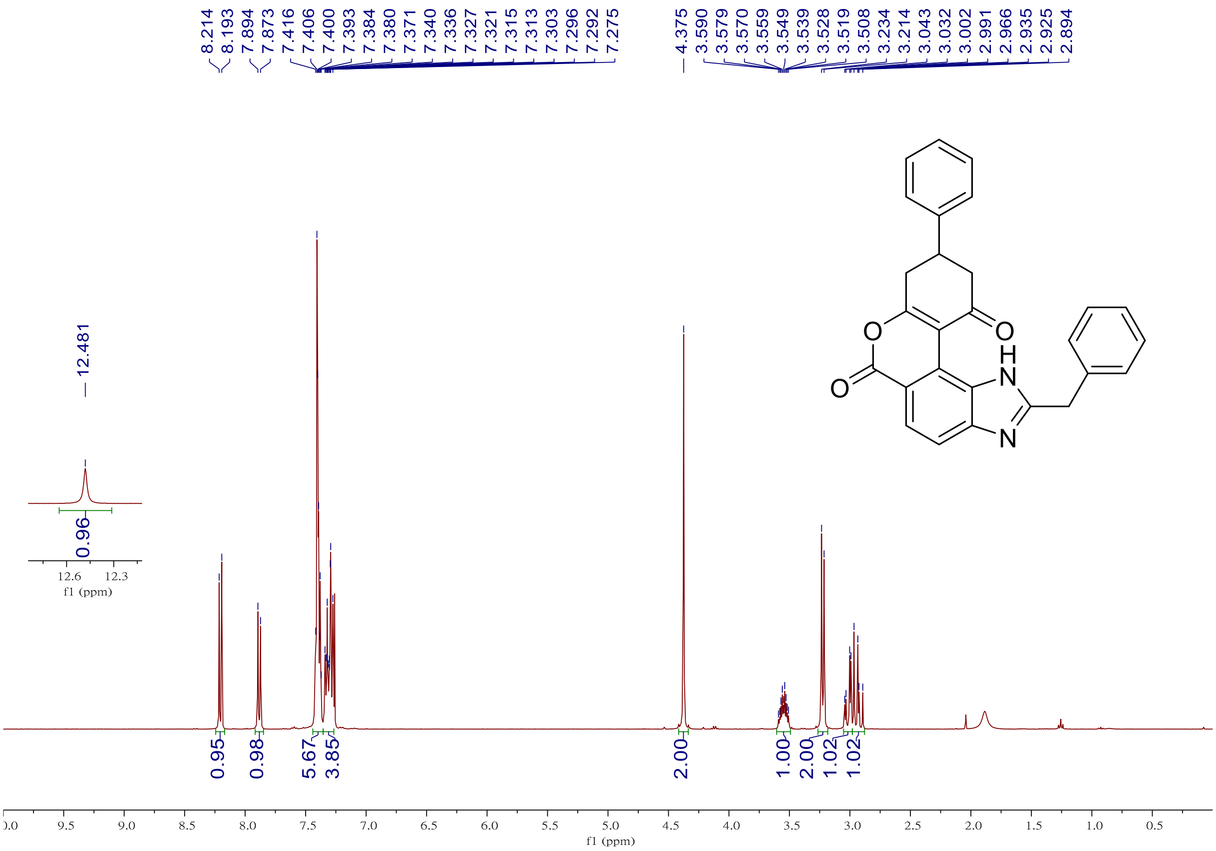


**Figure S165.** $^{1}$H NMR spectrum of compound **3ad** (400 MHz, $CDCl_3$).

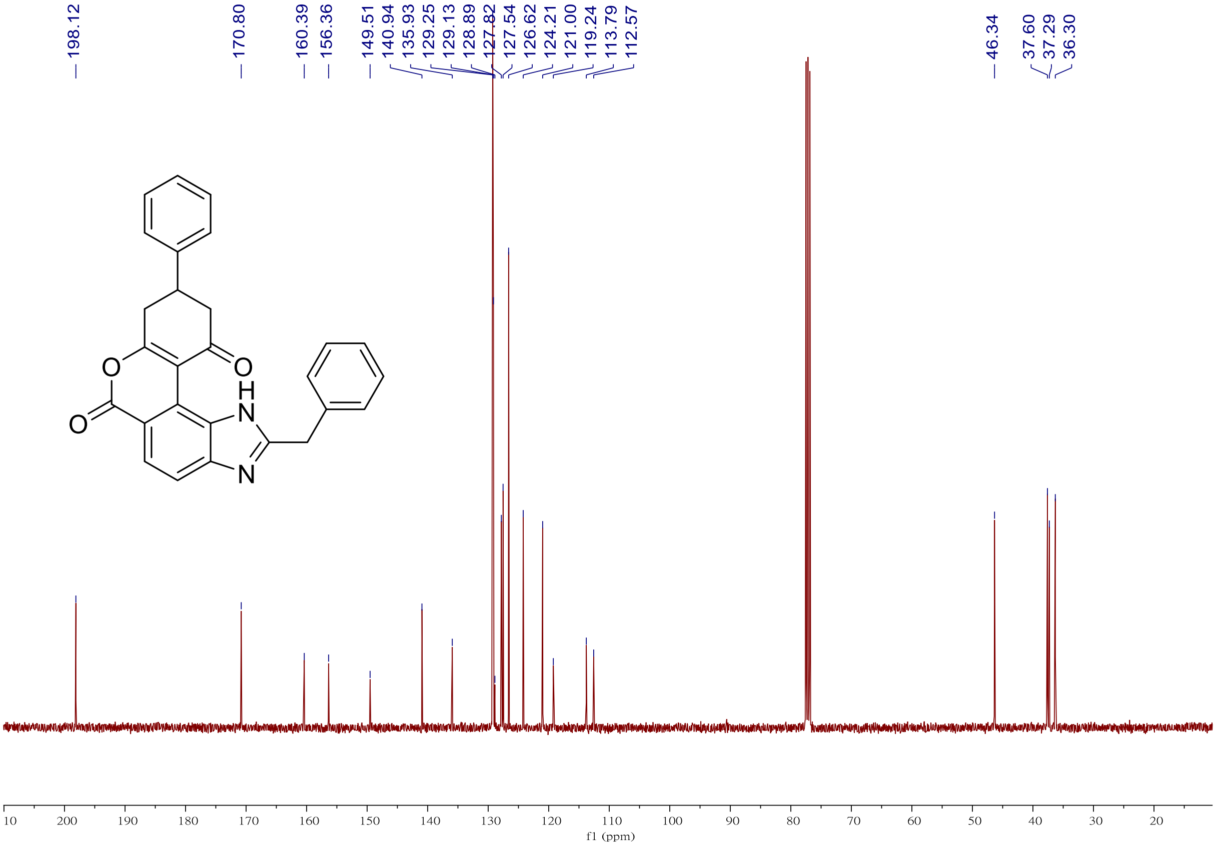


**Figure S166.** $^{13}C\{^{1}H\}$ NMR spectrum of compound **3ad** (101 MHz, $CDCl_3$).

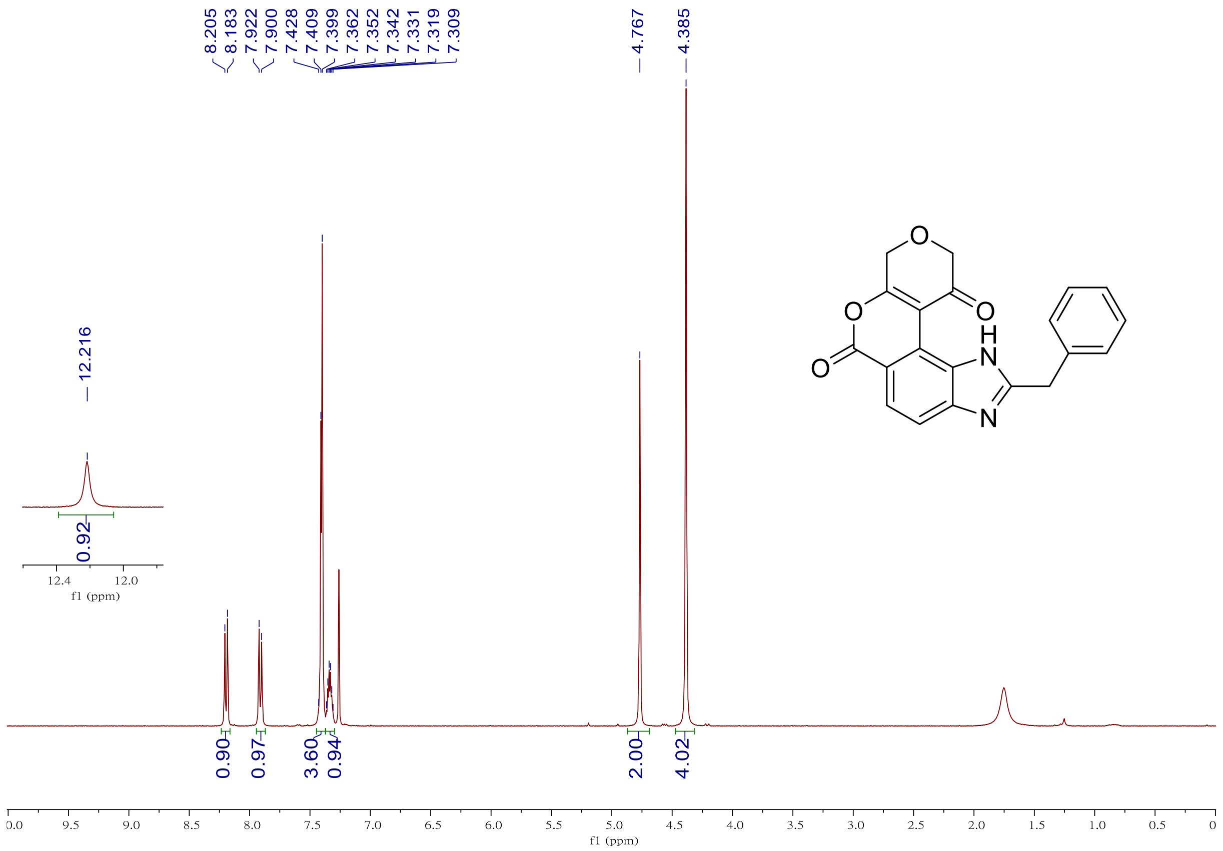

**Figure S167.** $^{1}$H NMR spectrum of compound **3ae** (400 MHz, $CDCl_3$).

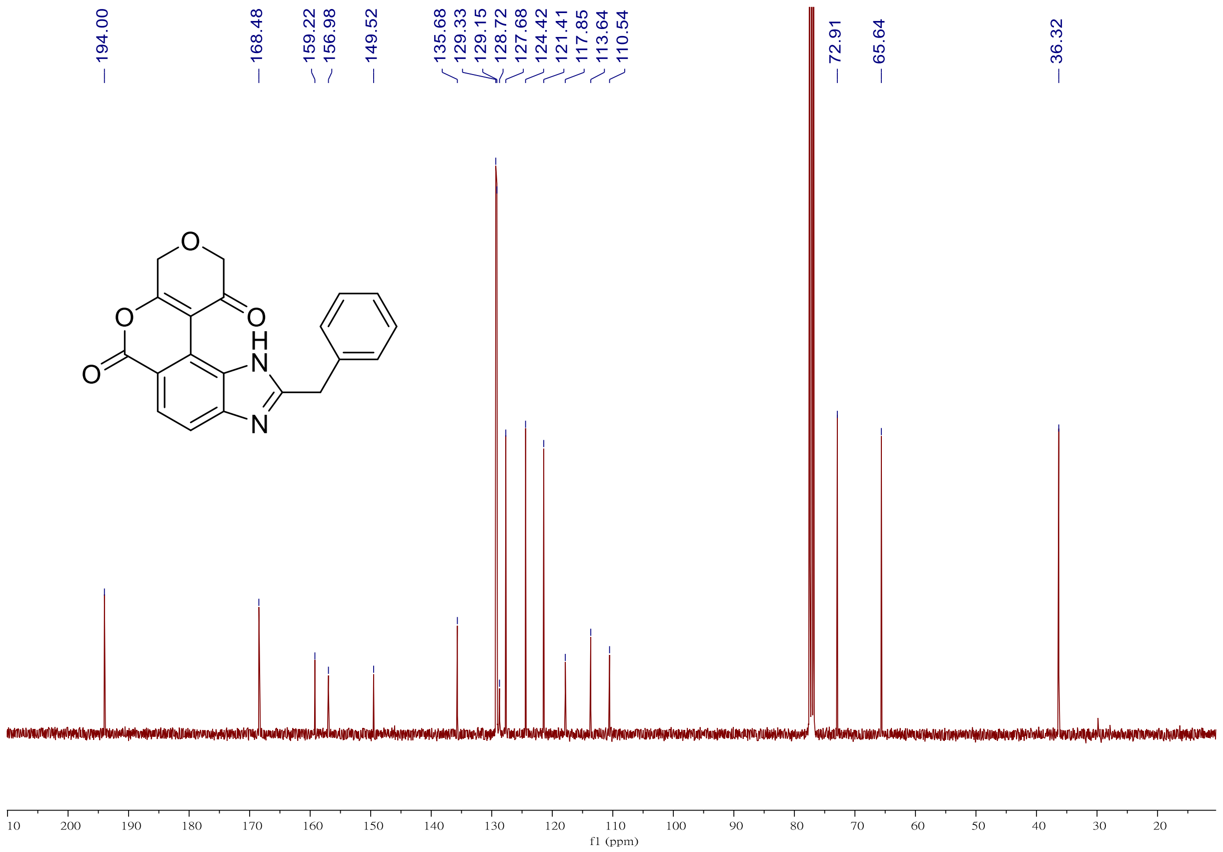

**Figure S168.** $^{13}C\{^{1}H\}$ NMR spectrum of compound **3ae** (101 MHz, $CDCl_3$).

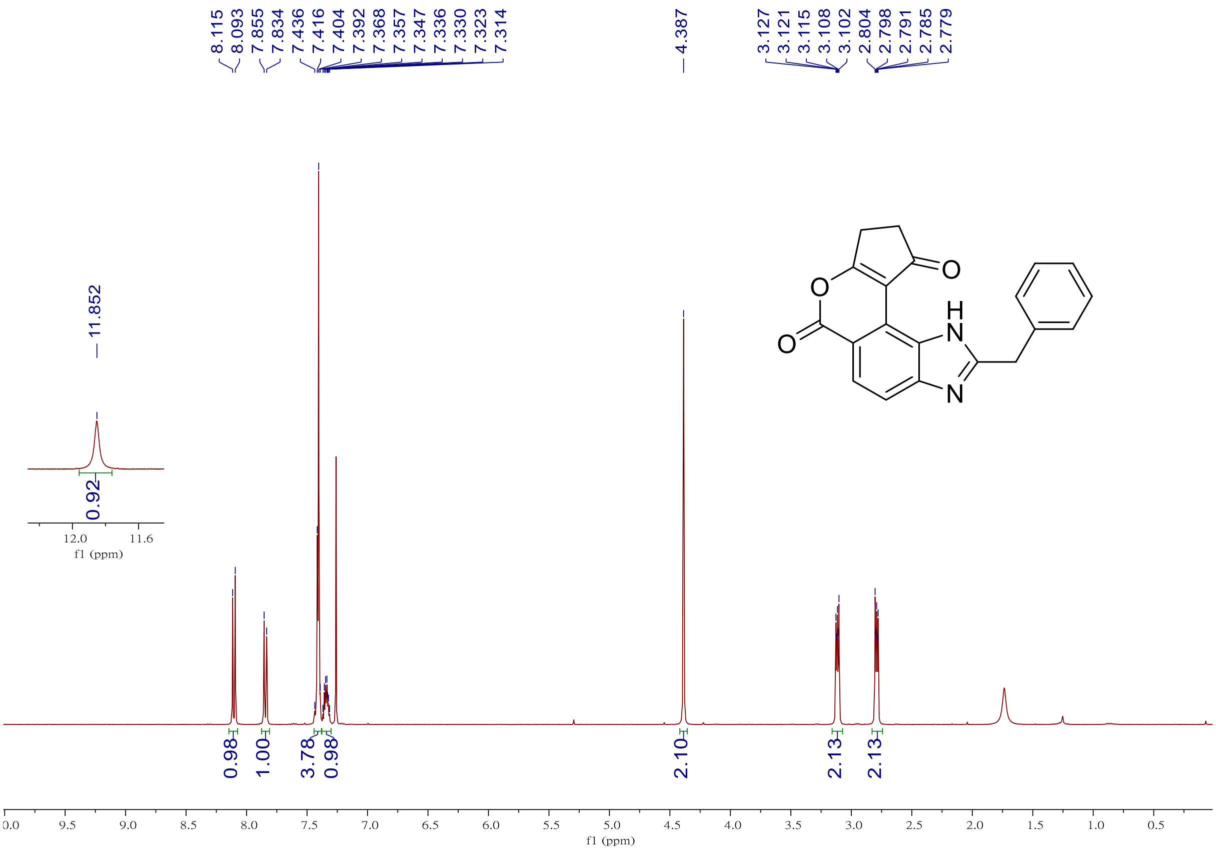


**Figure S169.** $^{1}$H NMR spectrum of compound **3af** (400 MHz, $CDCl_3$).

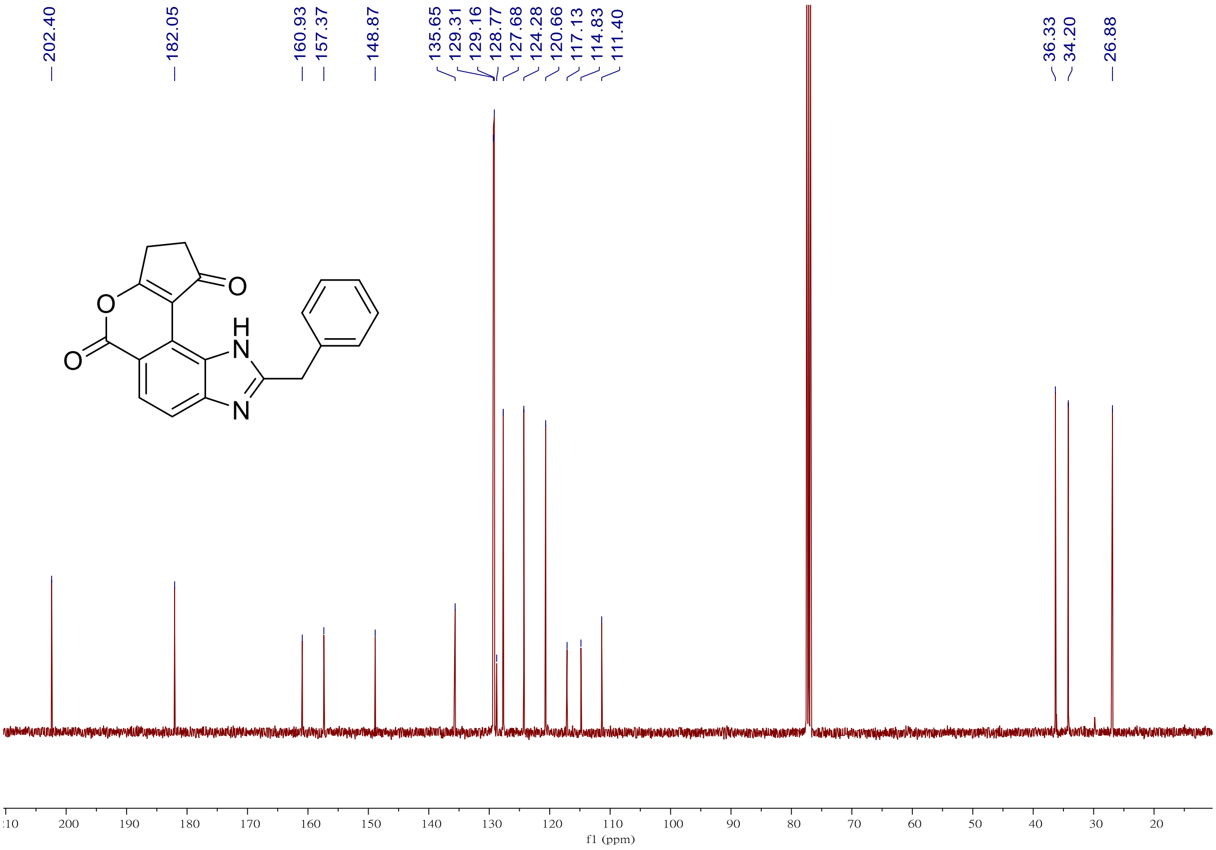


**Figure S170.** $^{13}C\{^{1}H\}$ NMR spectrum of compound **3af** (101 MHz, $CDCl_3$).

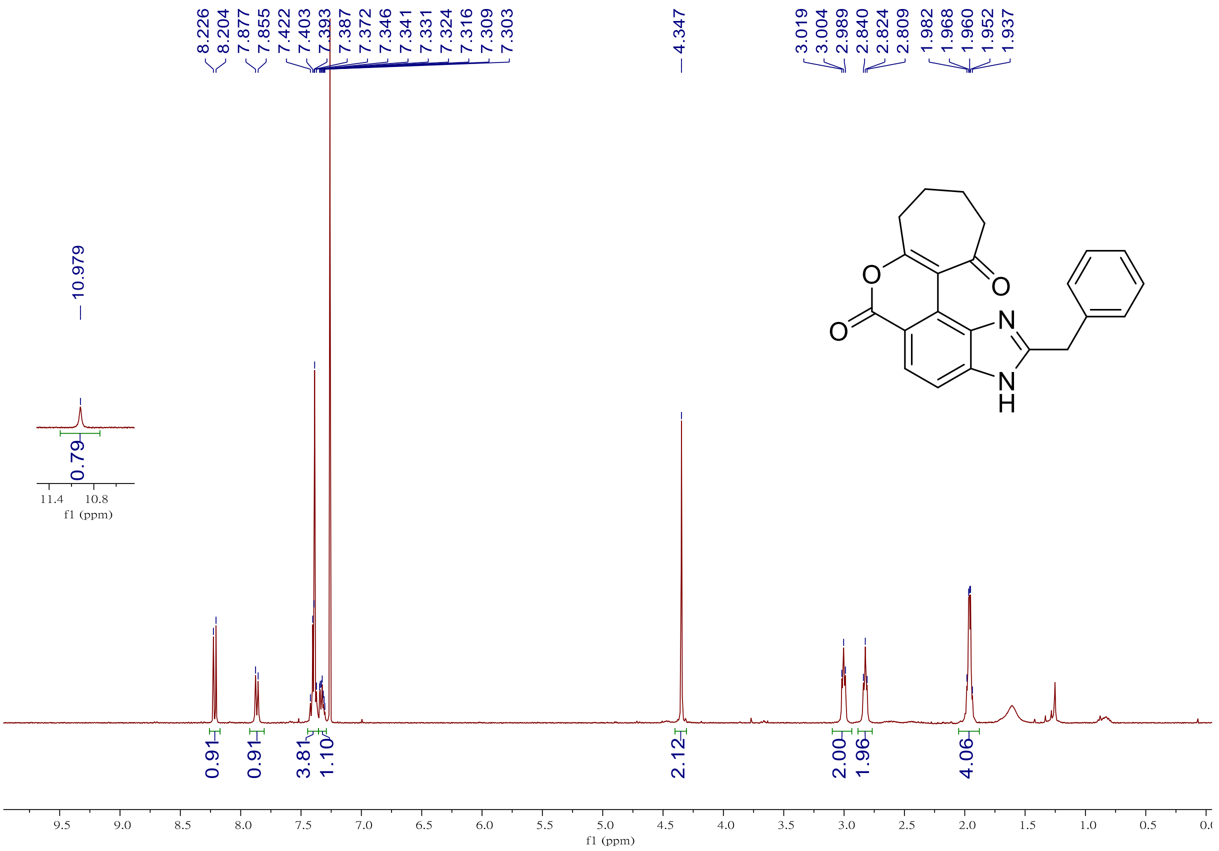


**Figure S171.** $^{1}$H NMR spectrum of compound **3ag** (400 MHz, $CDCl_3$).

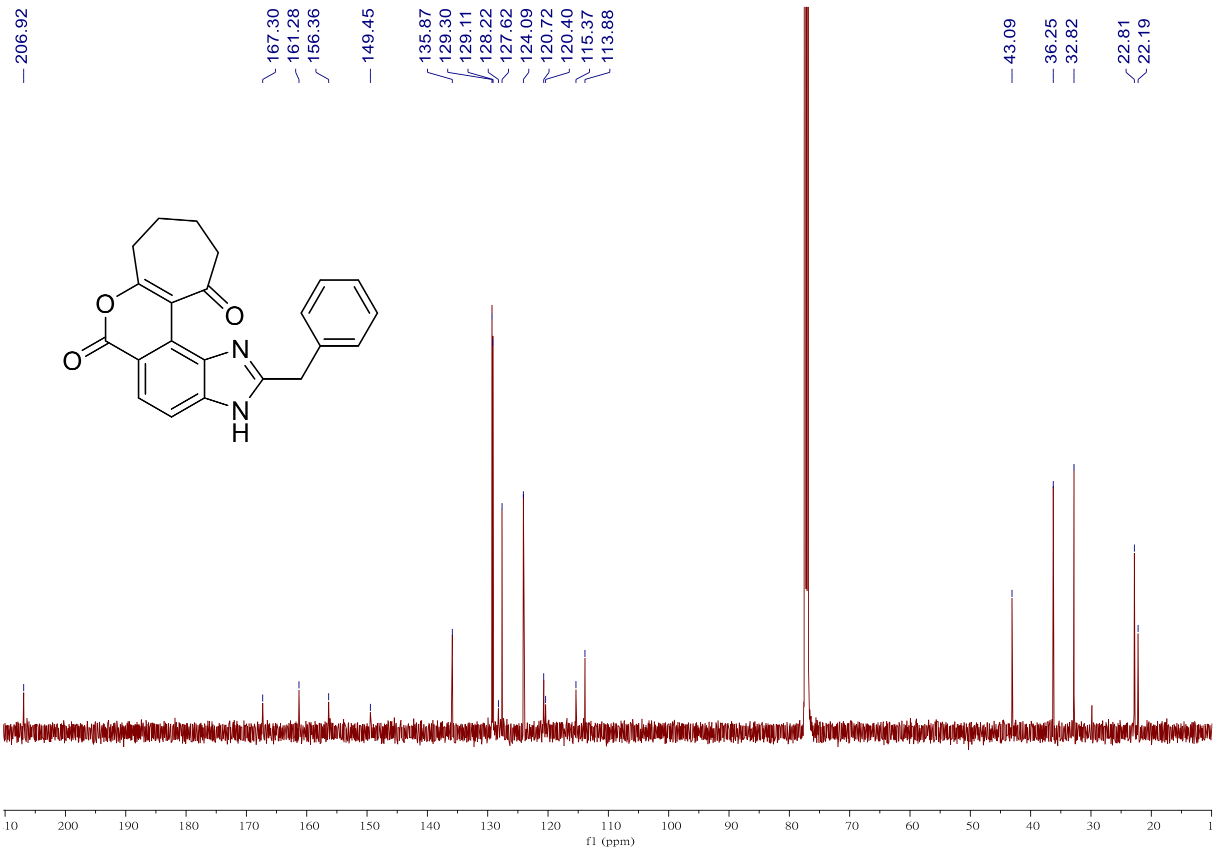


**Figure S172.** $^{13}$C{$^{1}$H}NMR spectrum of compound **3ag** (151 MHz, $CDCl_3$).

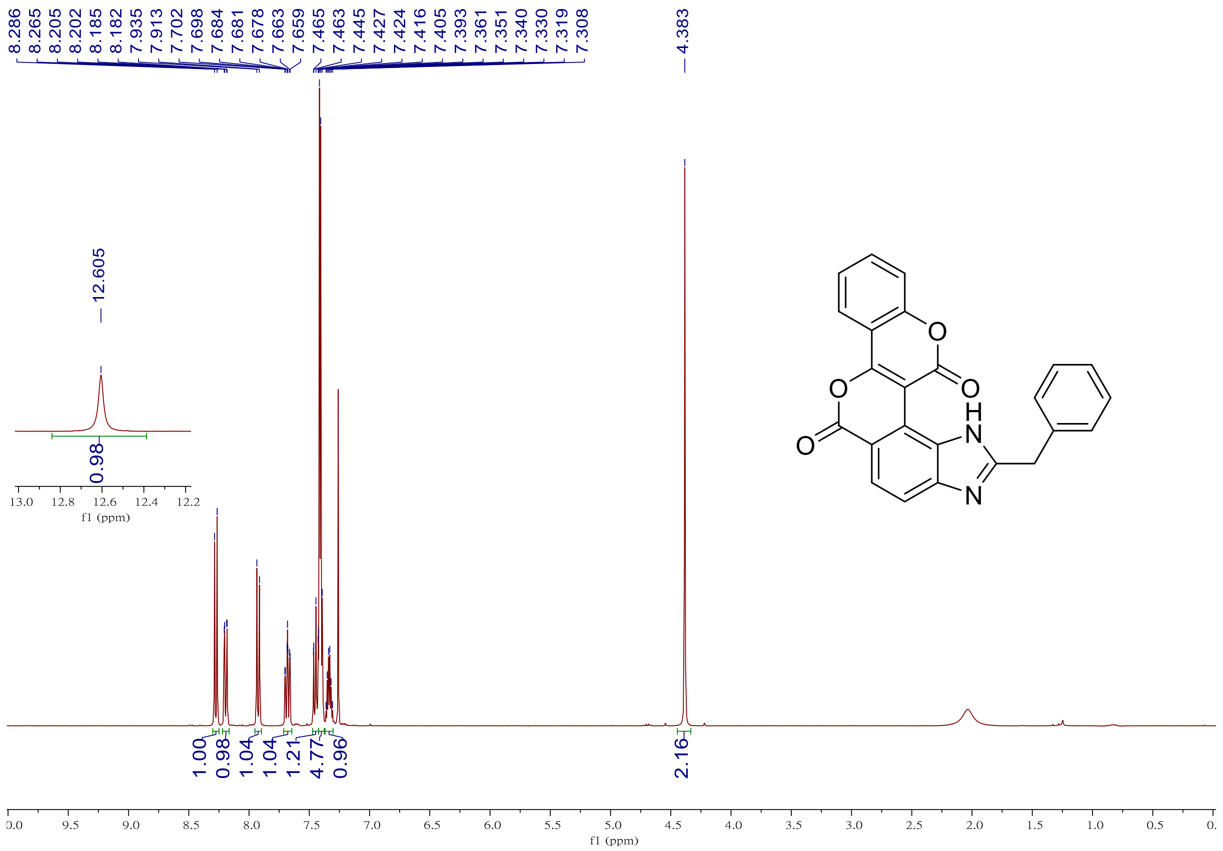

**Figure S173.** $^{1}H$ NMR spectrum of compound **3aj** (400 MHz, $CDCl_3$).

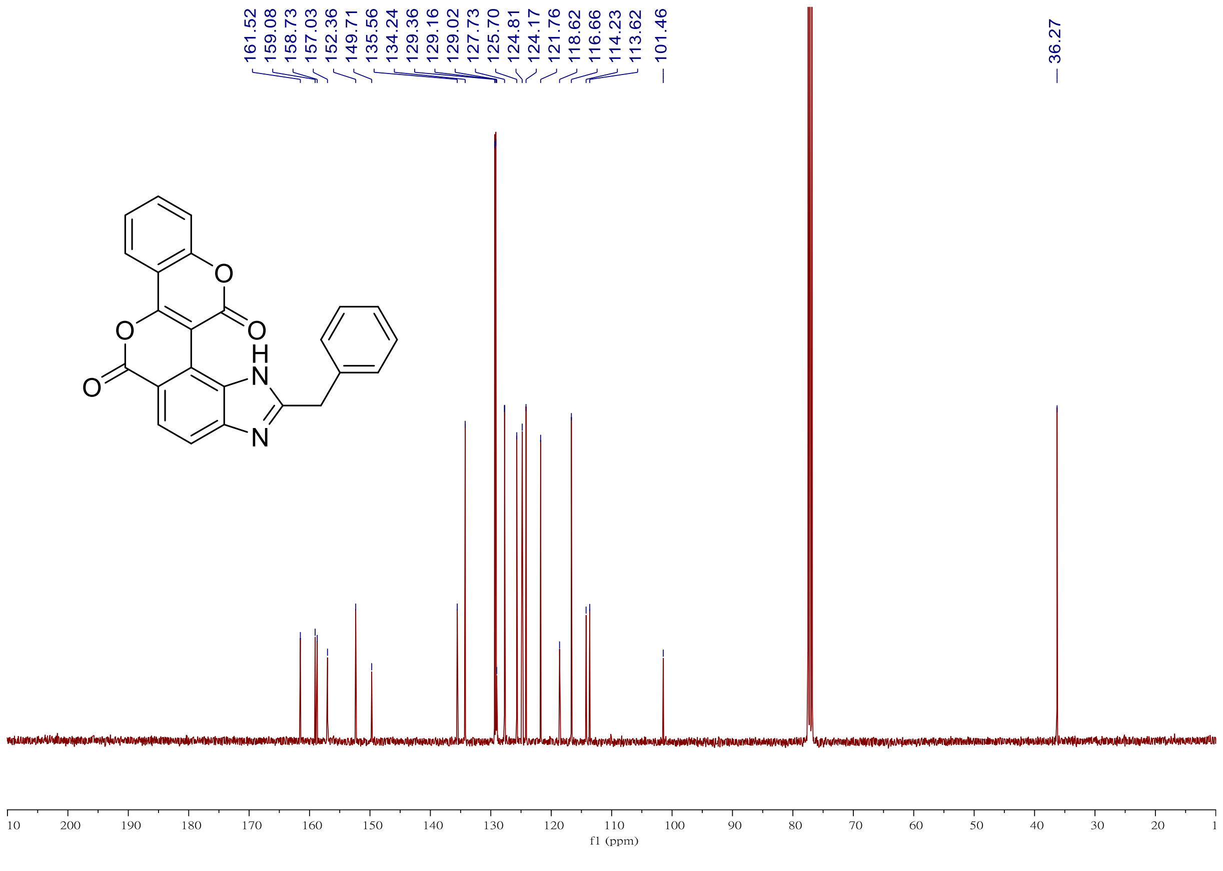

**Figure S174.** $^{13}C\{^{1}H\}$ NMR spectrum of compound **3aj** (101 MHz, $CDCl_3$).

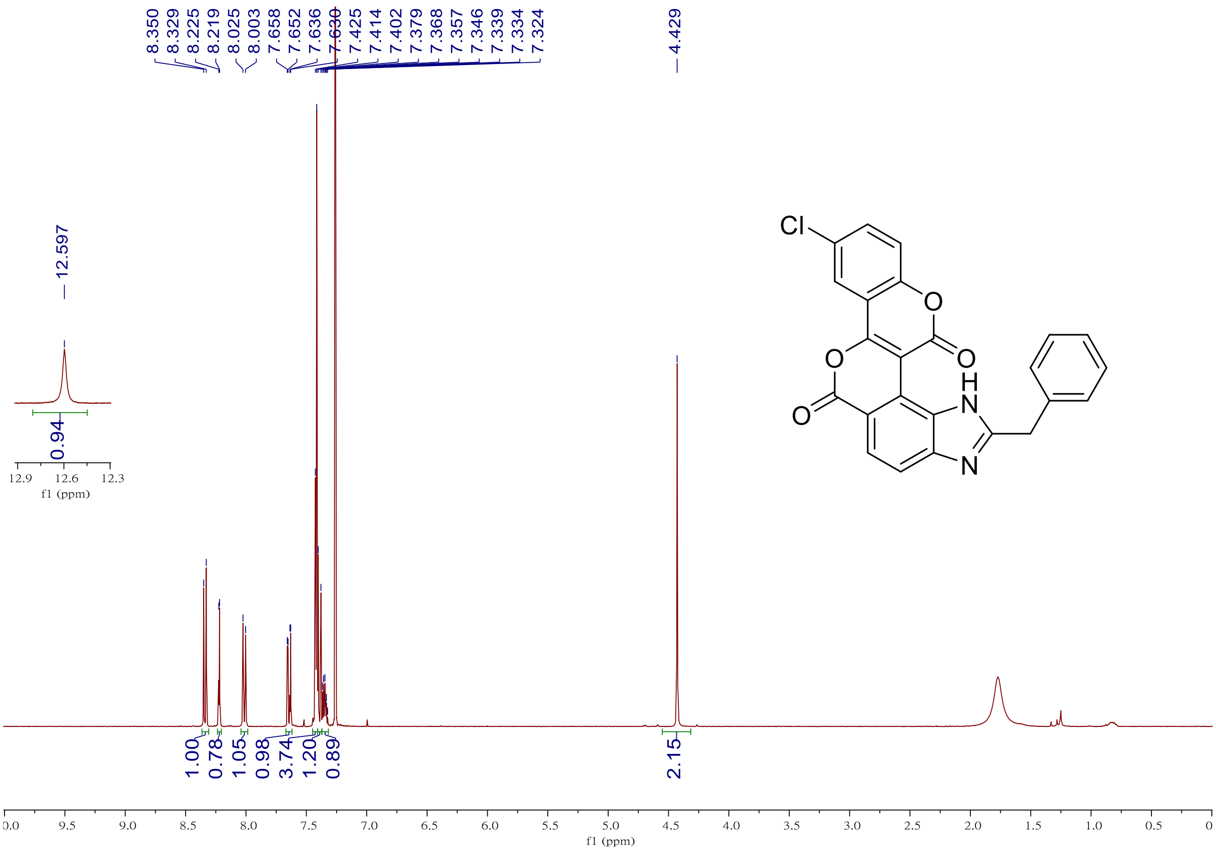

**Figure S175.** $^{1}$H NMR spectrum of compound **3ak** (400 MHz, $CDCl_3$).

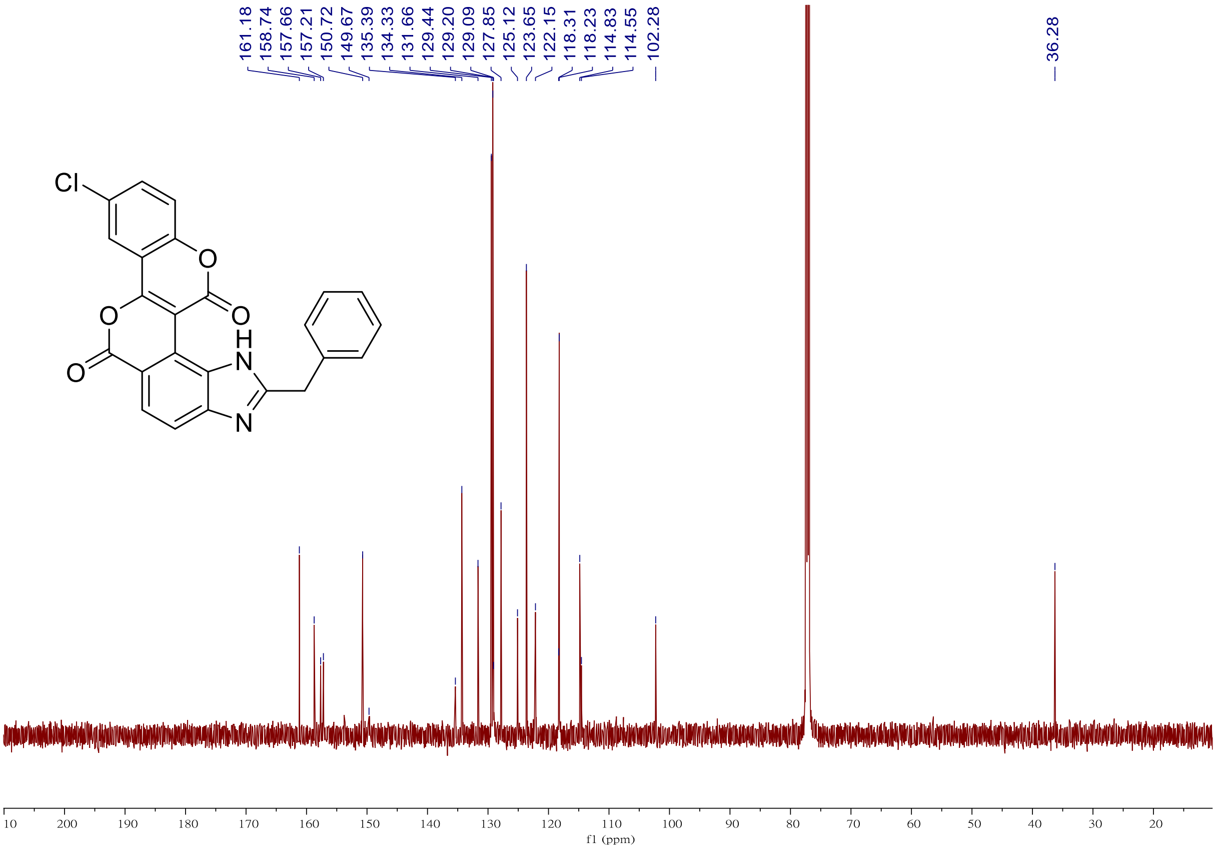

**Figure S176.** $^{13}C\{^{1}H\}$ NMR spectrum of compound **3ak** (151 MHz, $CDCl_3$).

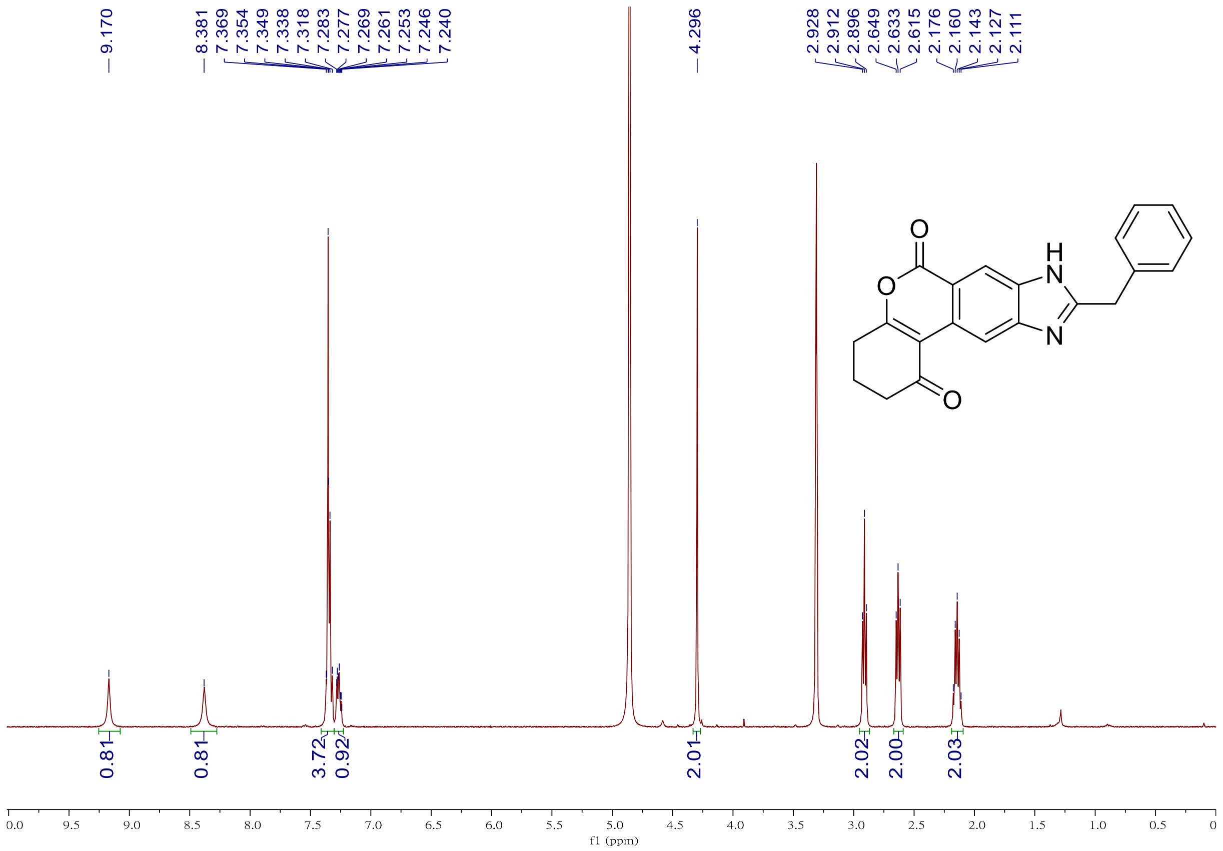


**Figure S177.** $^{1}$H NMR spectrum of compound **4a** (400 MHz, $CD_3OD$).

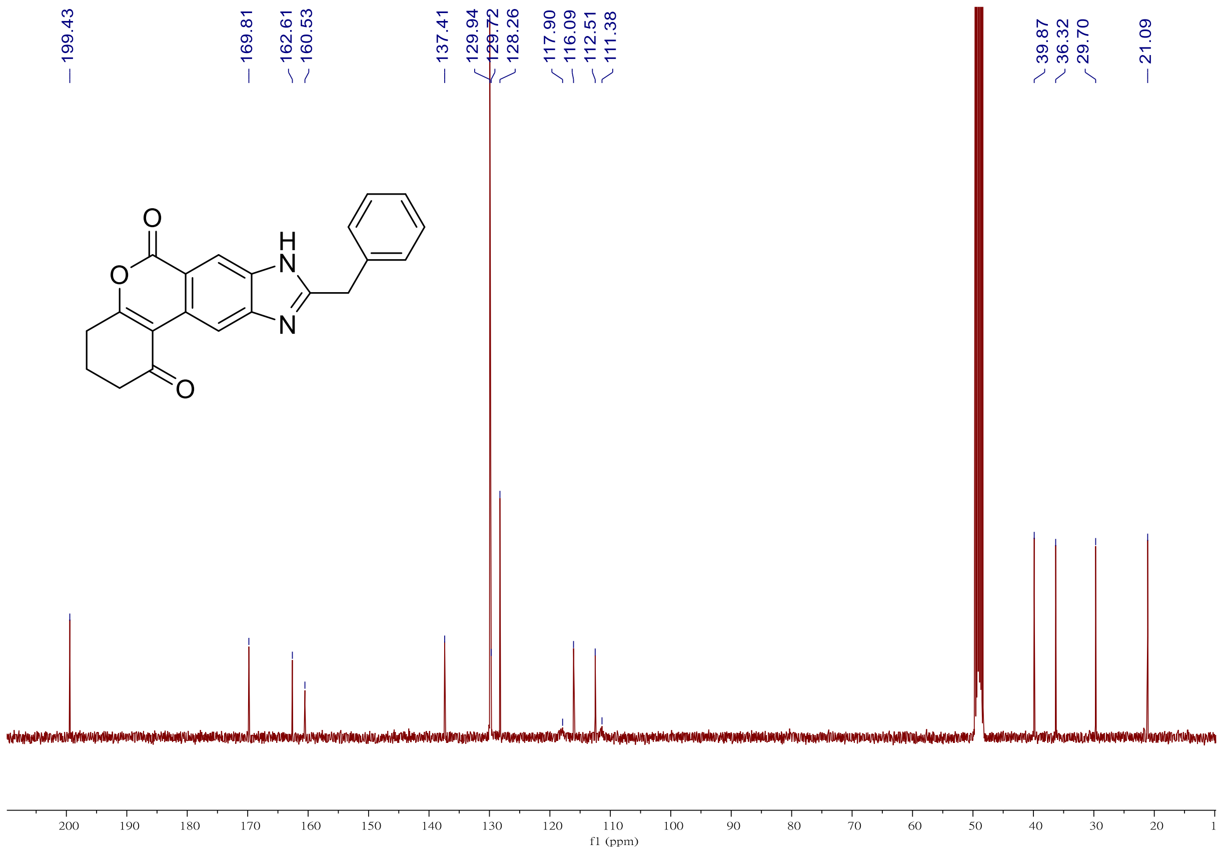


**Figure S178.** $^{13}C\{^{1}H\}$ NMR spectrum of compound **4a** (101 MHz, $CD_3OD$).

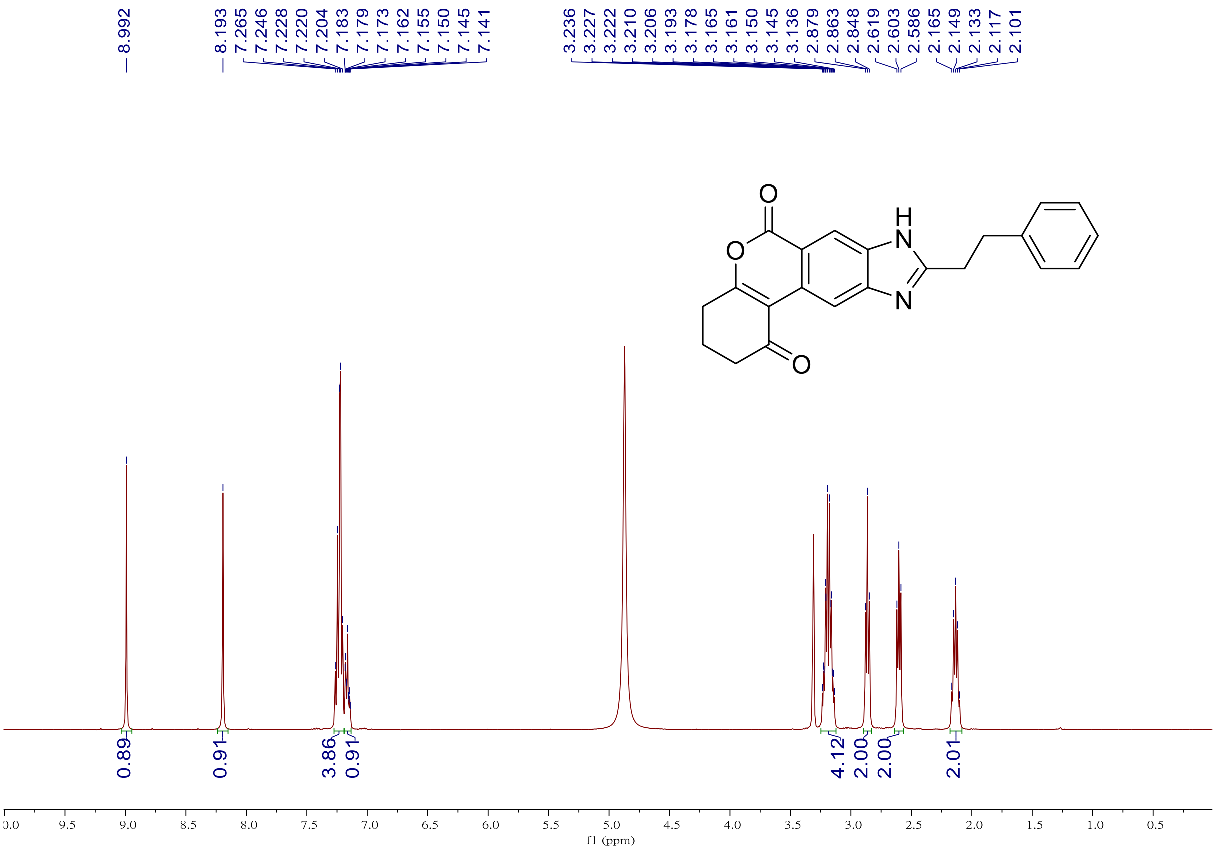


**Figure S179.** $^{1}H$ NMR spectrum of compound **4b** (400 MHz, $CD_3OD$).

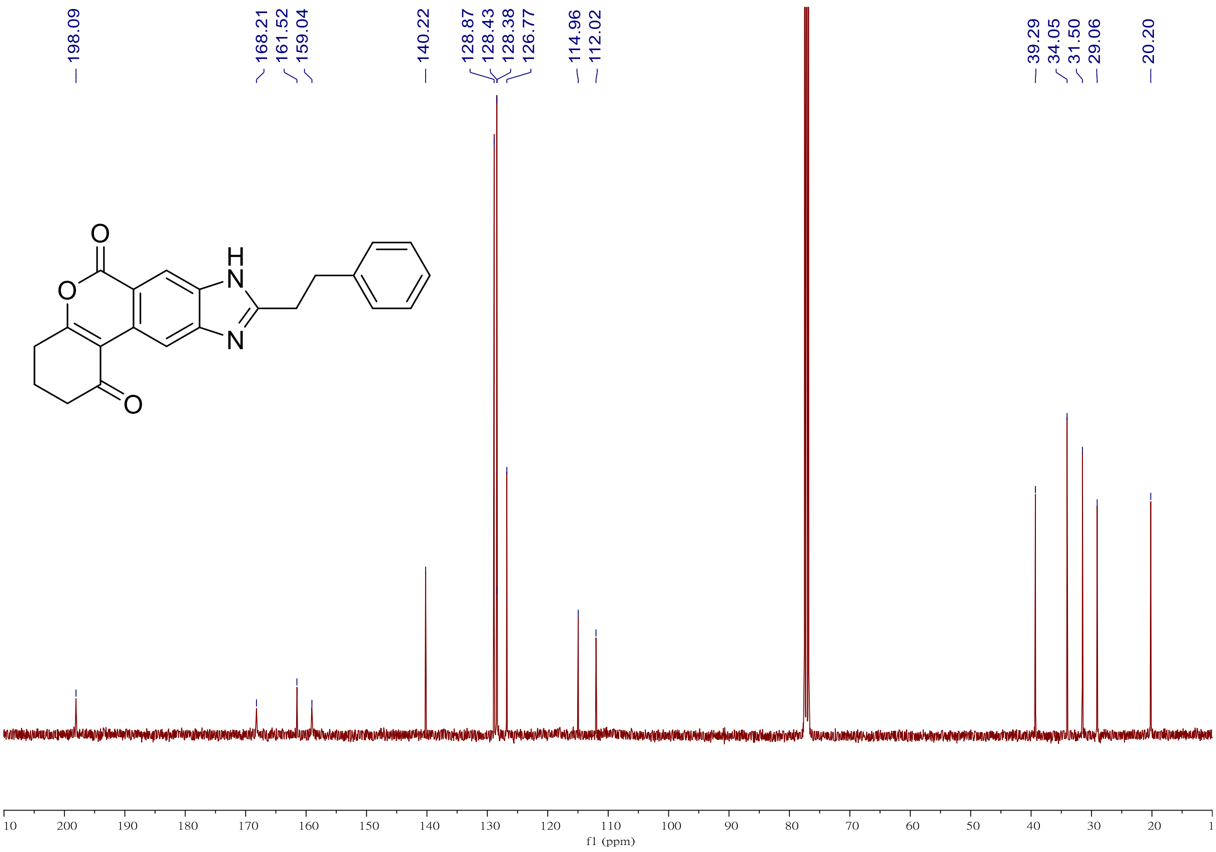


**Figure S180.** $^{13}C\{^{1}H\}$ NMR spectrum of compound **4b** (101 MHz, $CDCl_3$).

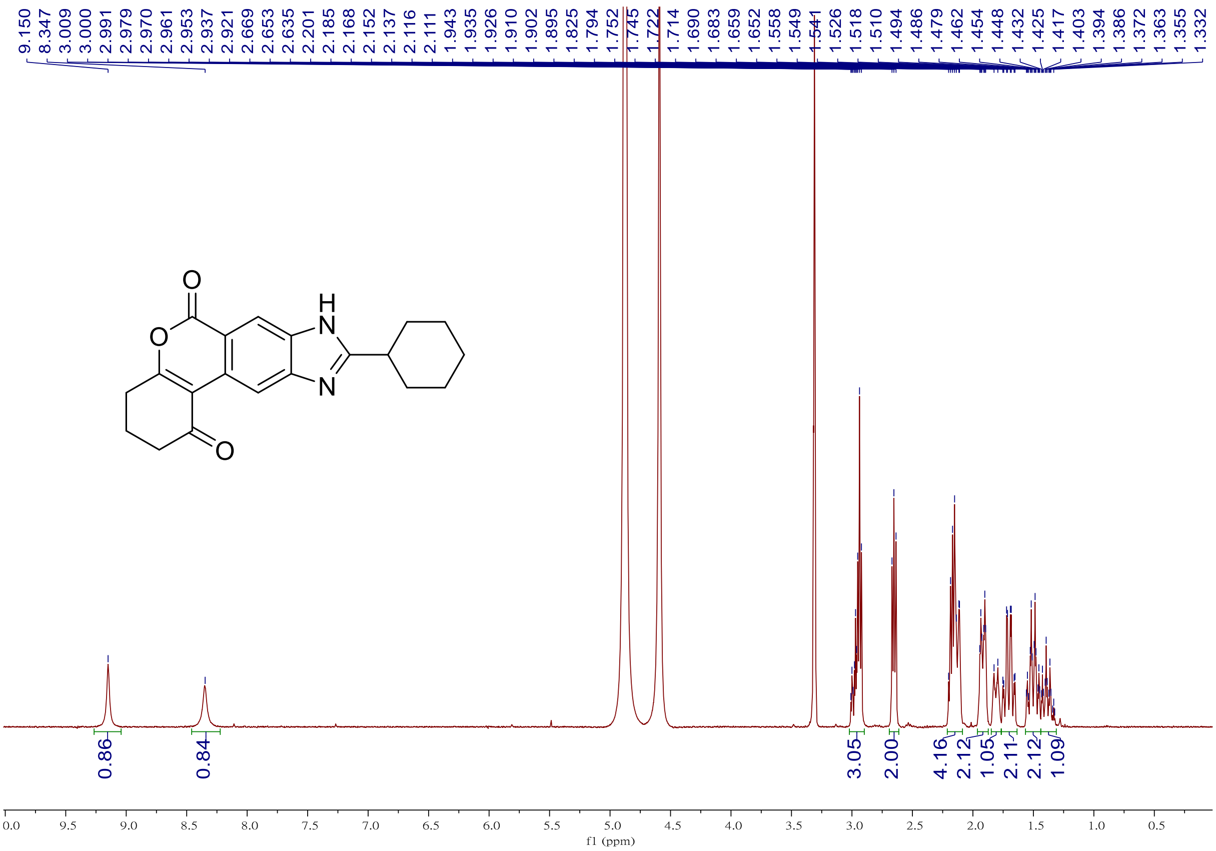


**Figure S181.** $^{1}$H NMR spectrum of compound **4c** (400 MHz, $CD_3OD$).

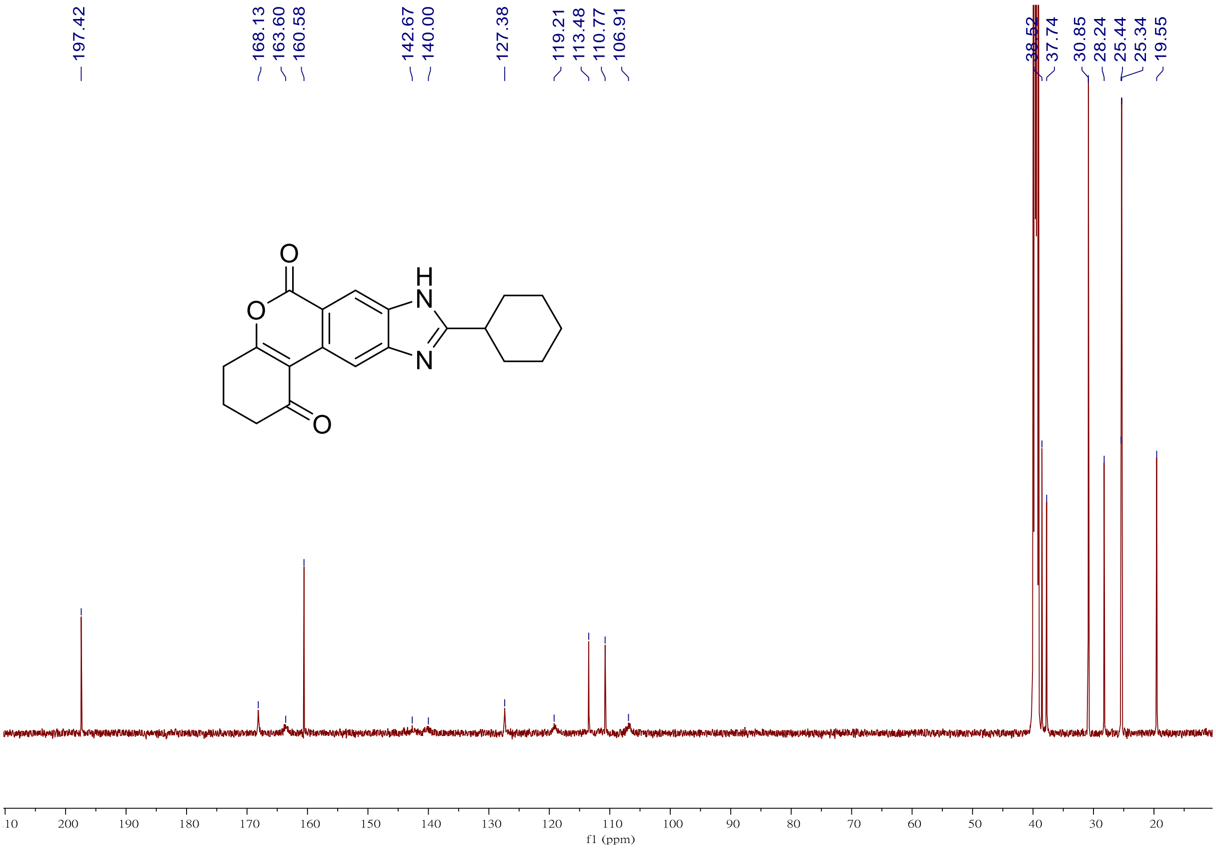


**Figure S182.** $^{13}C\{^{1}H\}$ NMR spectrum of compound **4c** (151 MHz, $(CD_3)_2SO$).

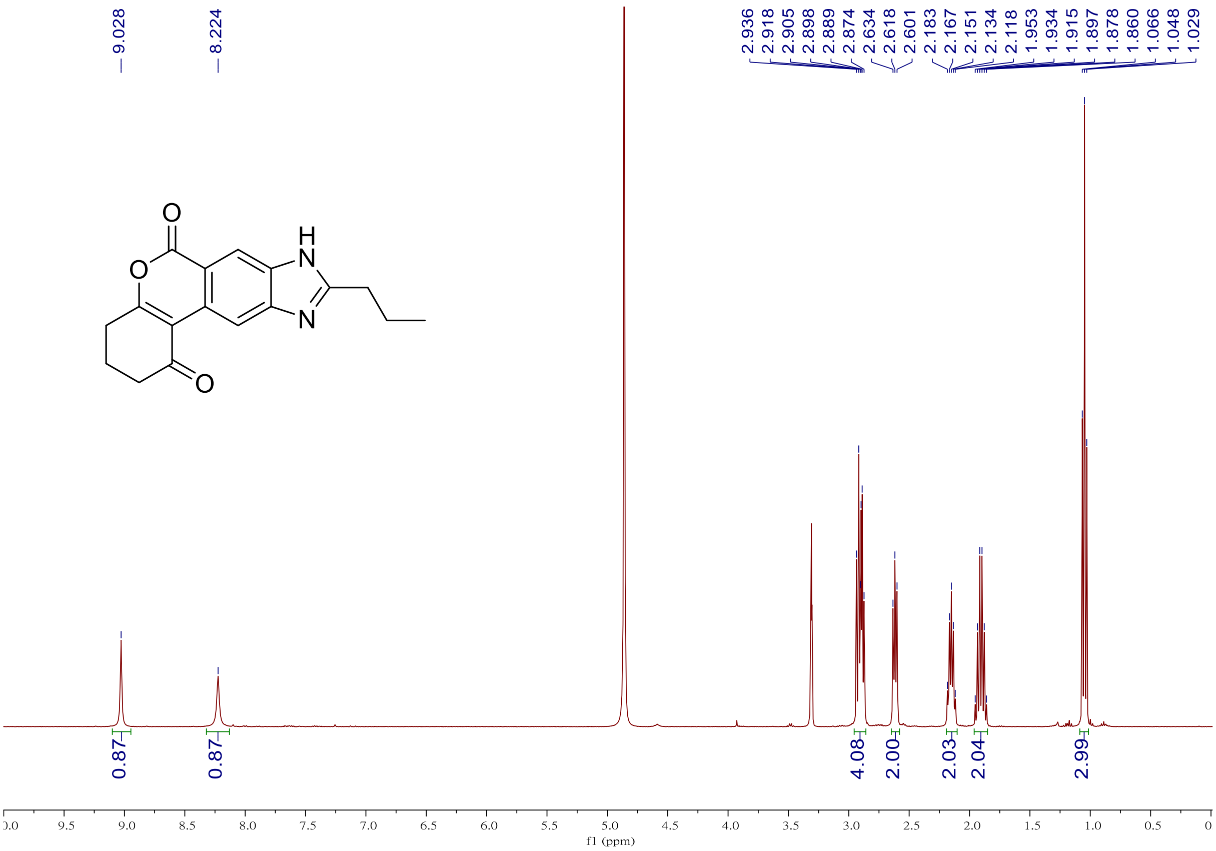


**Figure S183.** $^{1}H$ NMR spectrum of compound **4d** (400 MHz, $CD_3OD$).

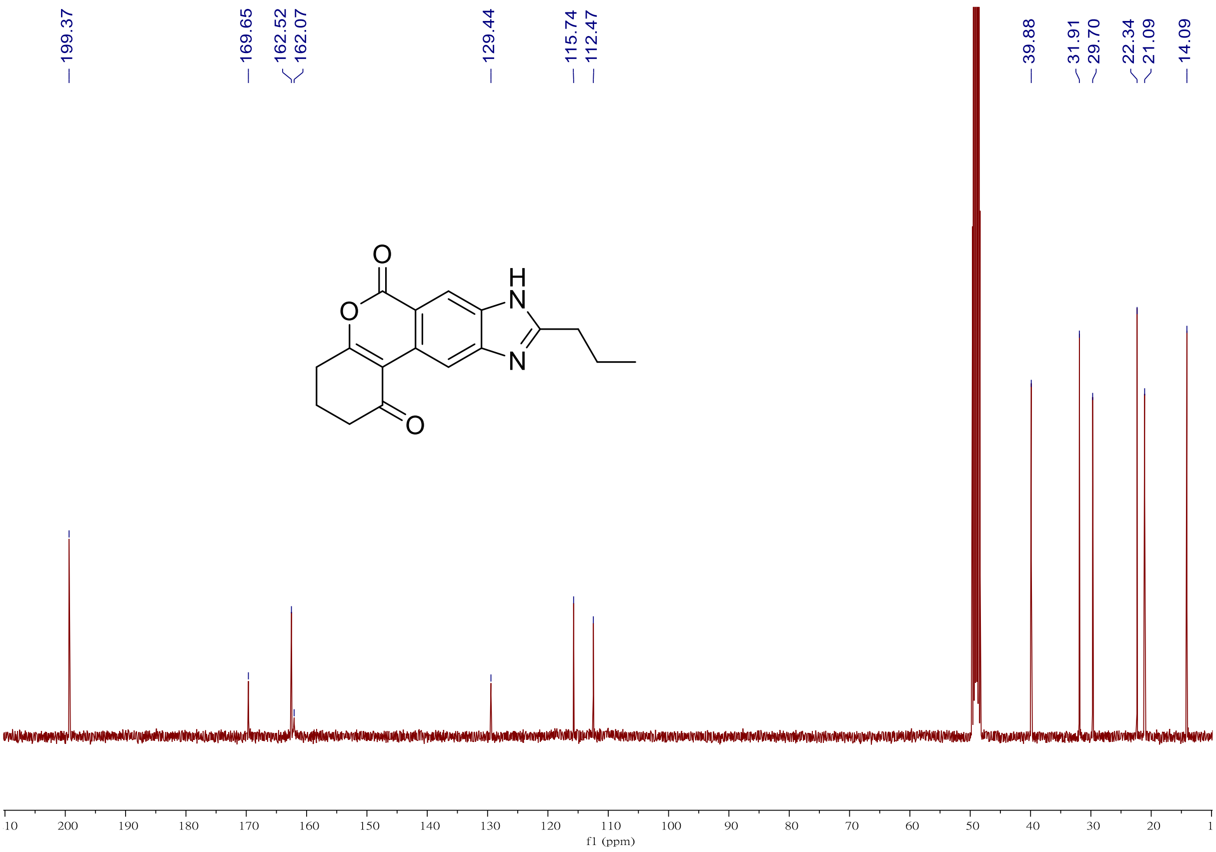


**Figure S184.** $^{13}C\{^{1}H\}$ NMR spectrum of compound **4d** (101 MHz, $CD_3OD$).

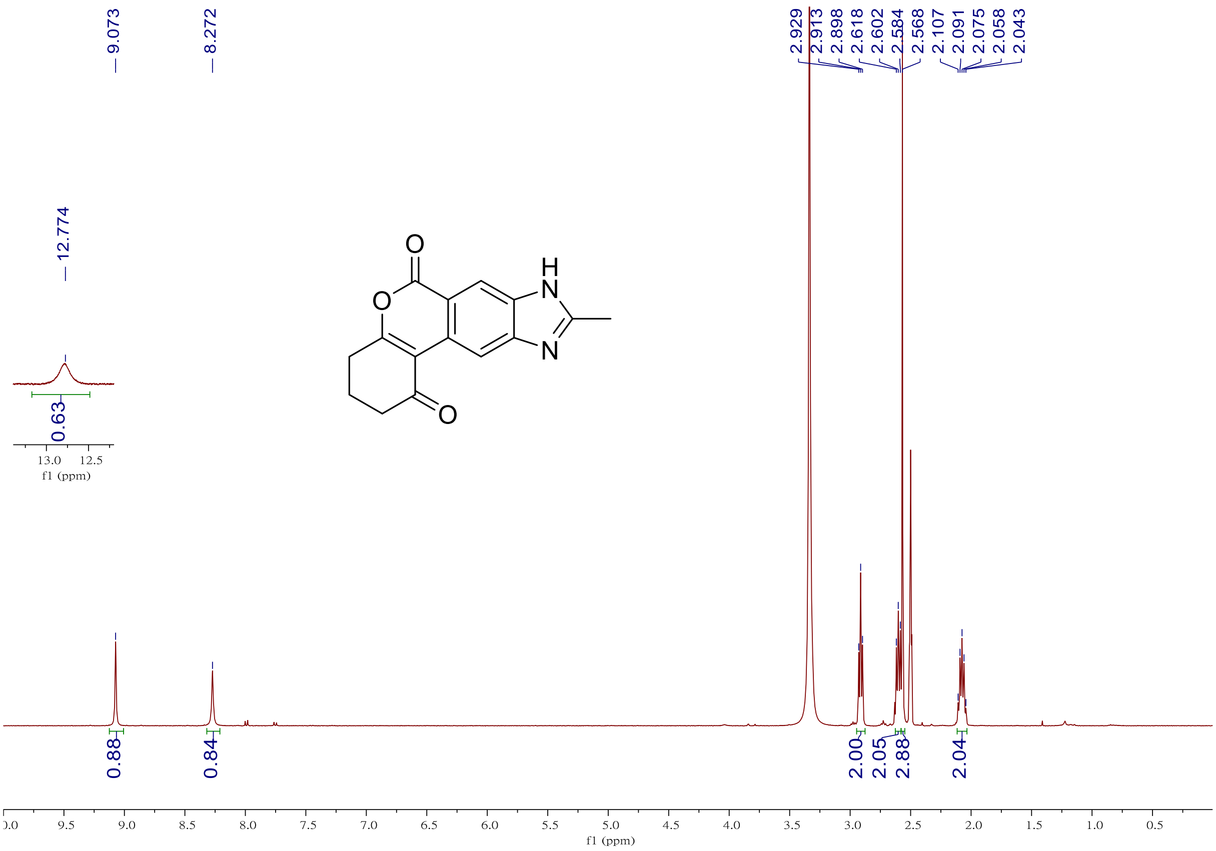


**Figure S185.** $^{1}$H NMR spectrum of compound **4e** (400 MHz, $(CD_3)_2SO$).

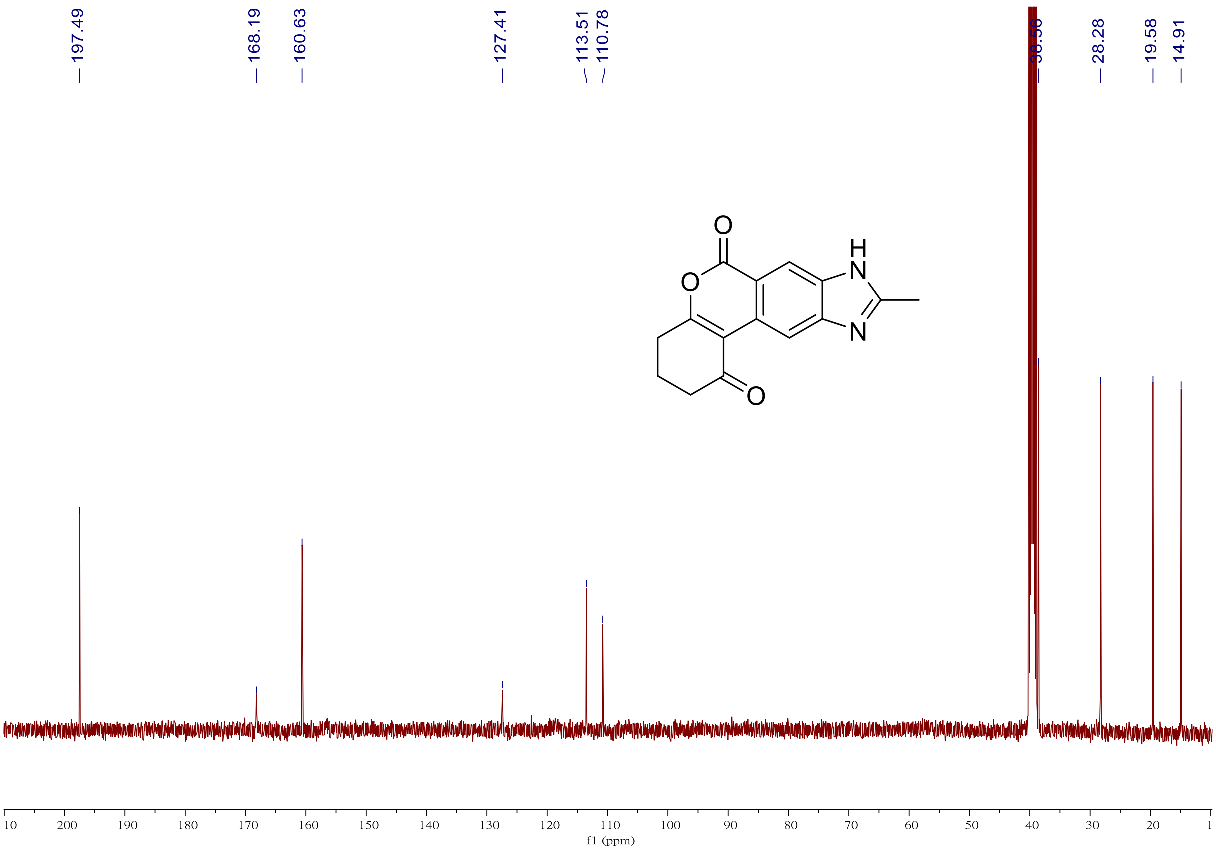


**Figure S186.** $^{13}C\{^{1}H\}$ NMR spectrum of compound **4e** (101 MHz, $(CD_3)_2SO$).

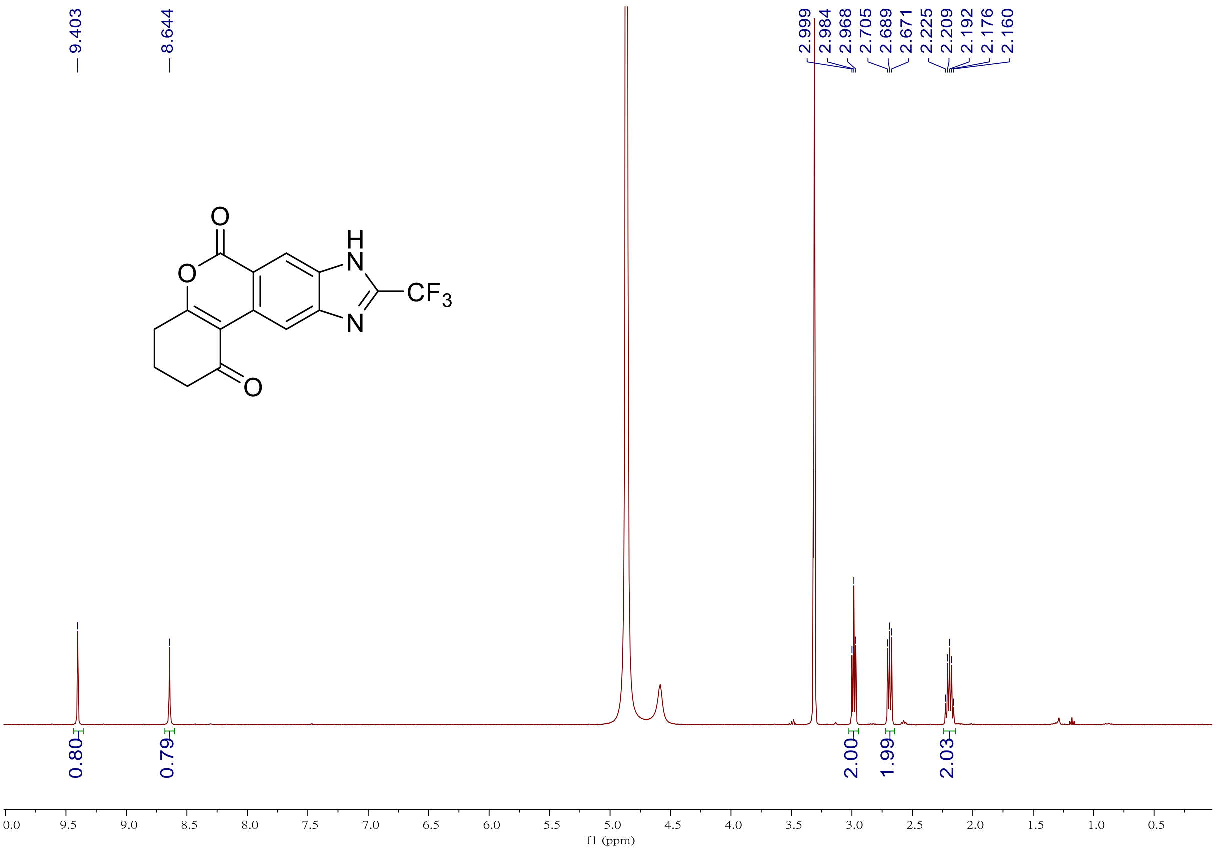


**Figure S187.** $^{1}H$ NMR spectrum of compound **4f** (400 MHz, $CD_3OD$).

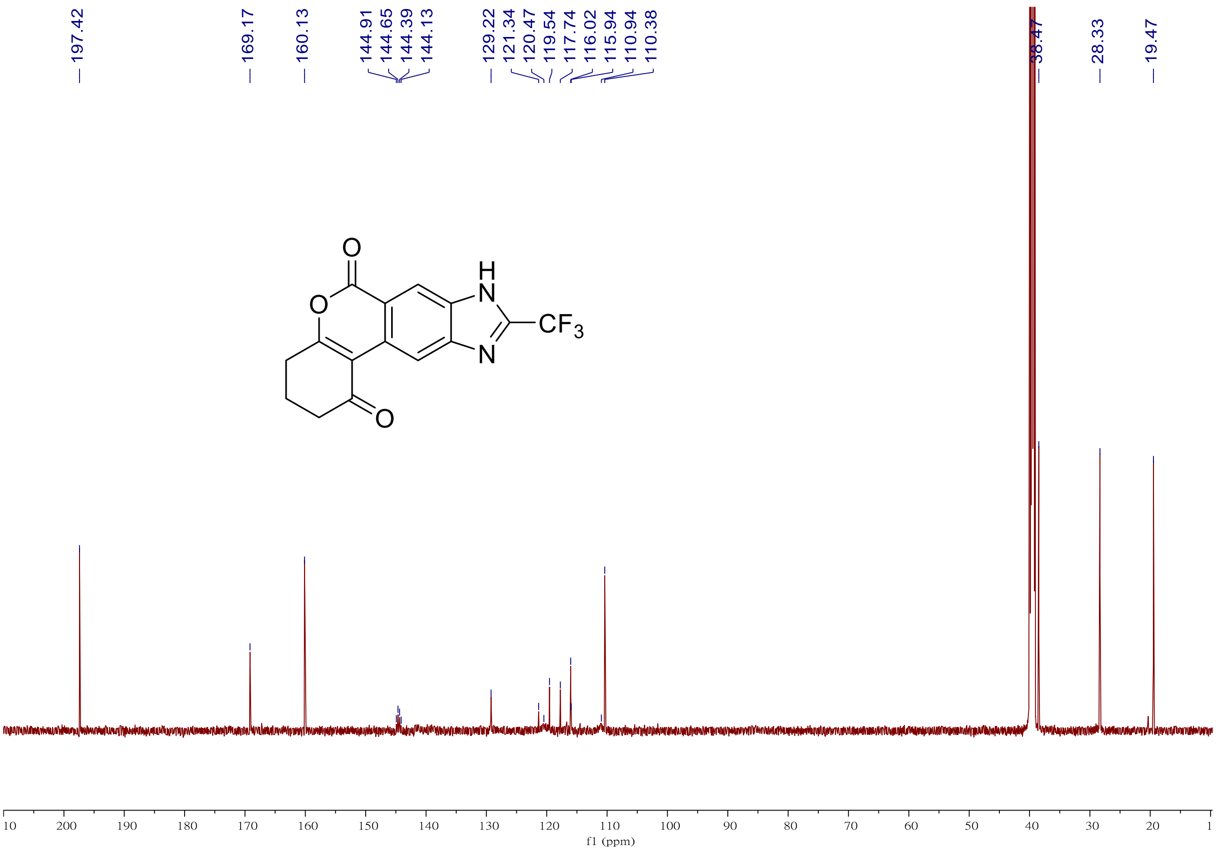


**Figure S188.** $^{13}C\{^{1}H\}$ NMR spectrum of compound **4f** (151 MHz, $(CD_3)_2SO$).

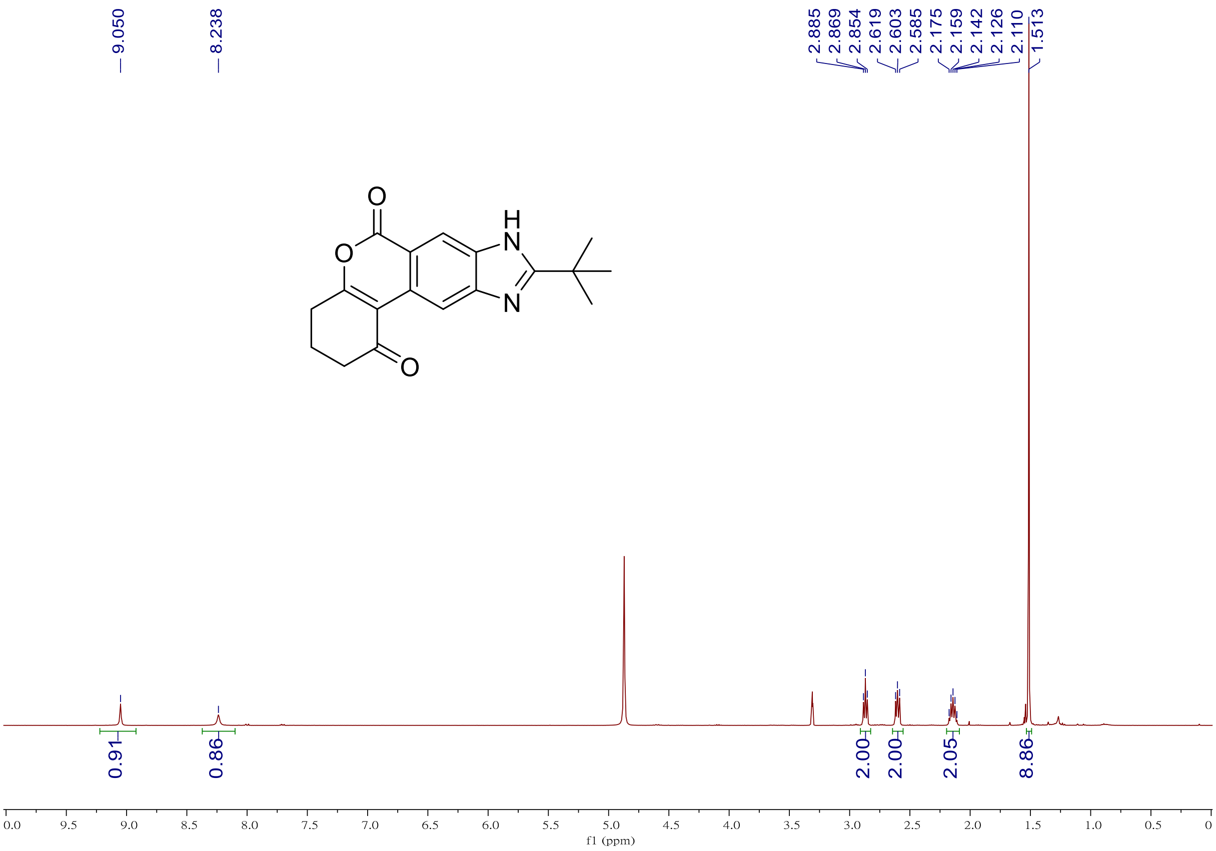


**Figure S189.** $^{1}$H NMR spectrum of compound **4g** (400 MHz, $CD_3OD$).

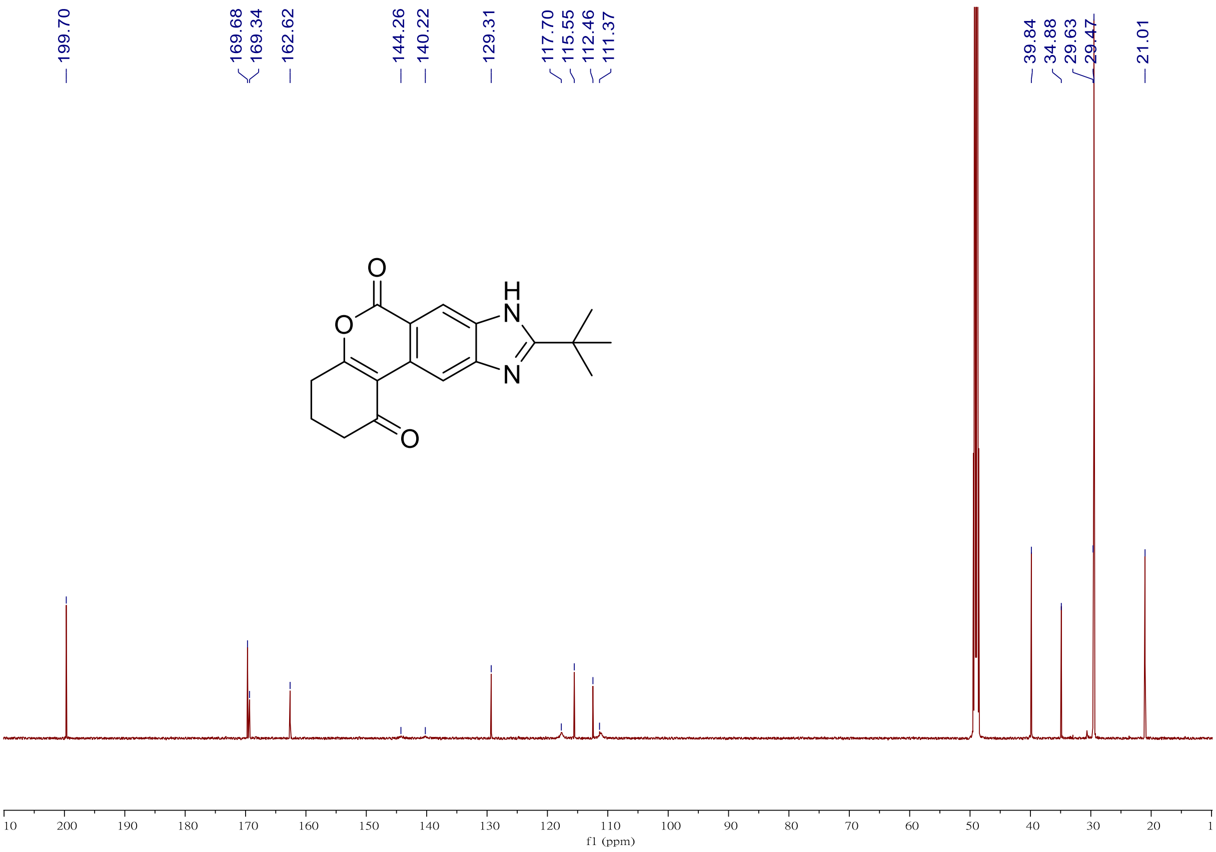


**Figure S190.** $^{13}C\{^{1}H\}$ NMR spectrum of compound **4g** (151 MHz, $CD_3OD$).

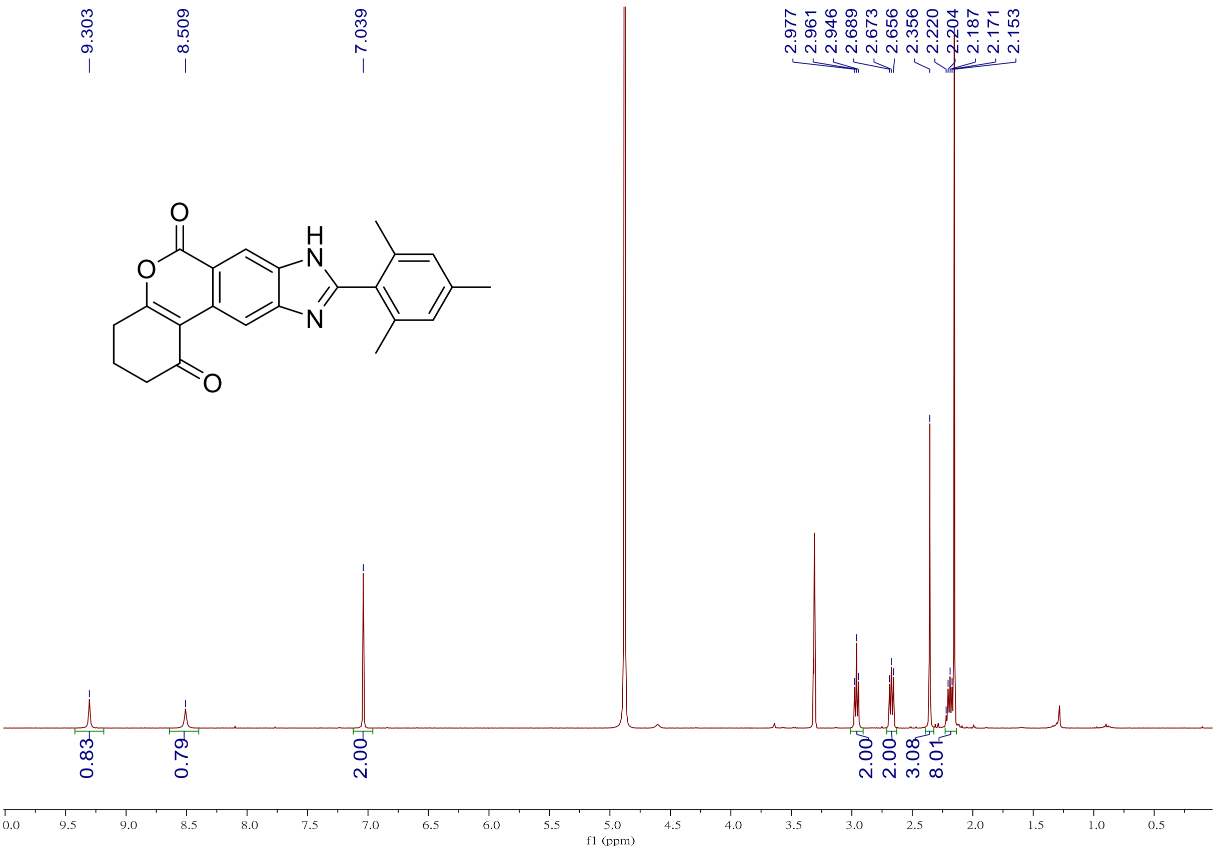


**Figure S191.** $^{1}H$ NMR spectrum of compound **4h** (400 MHz, $CD_3OD$).

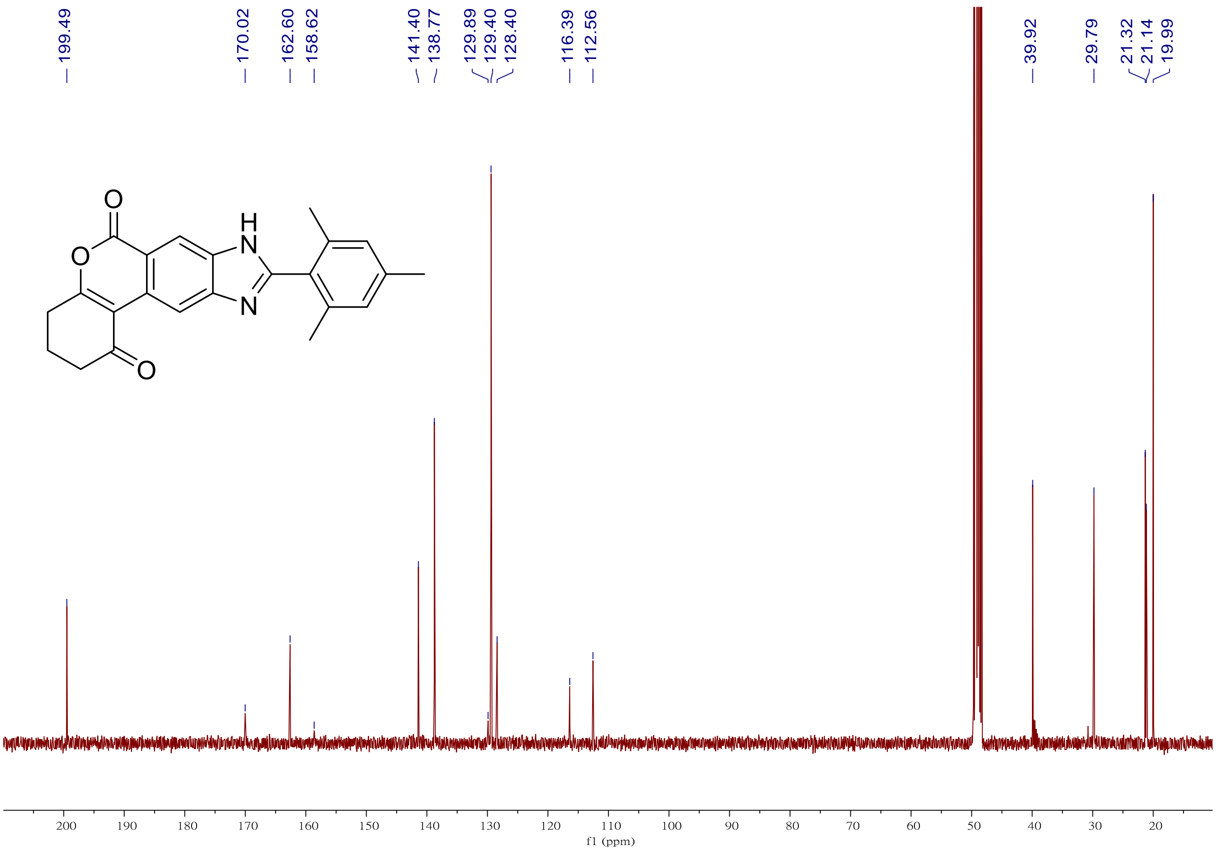


**Figure S192.** $^{13}C\{^{1}H\}$ NMR spectrum of compound **4h** (101 MHz, $CD_3OD$).

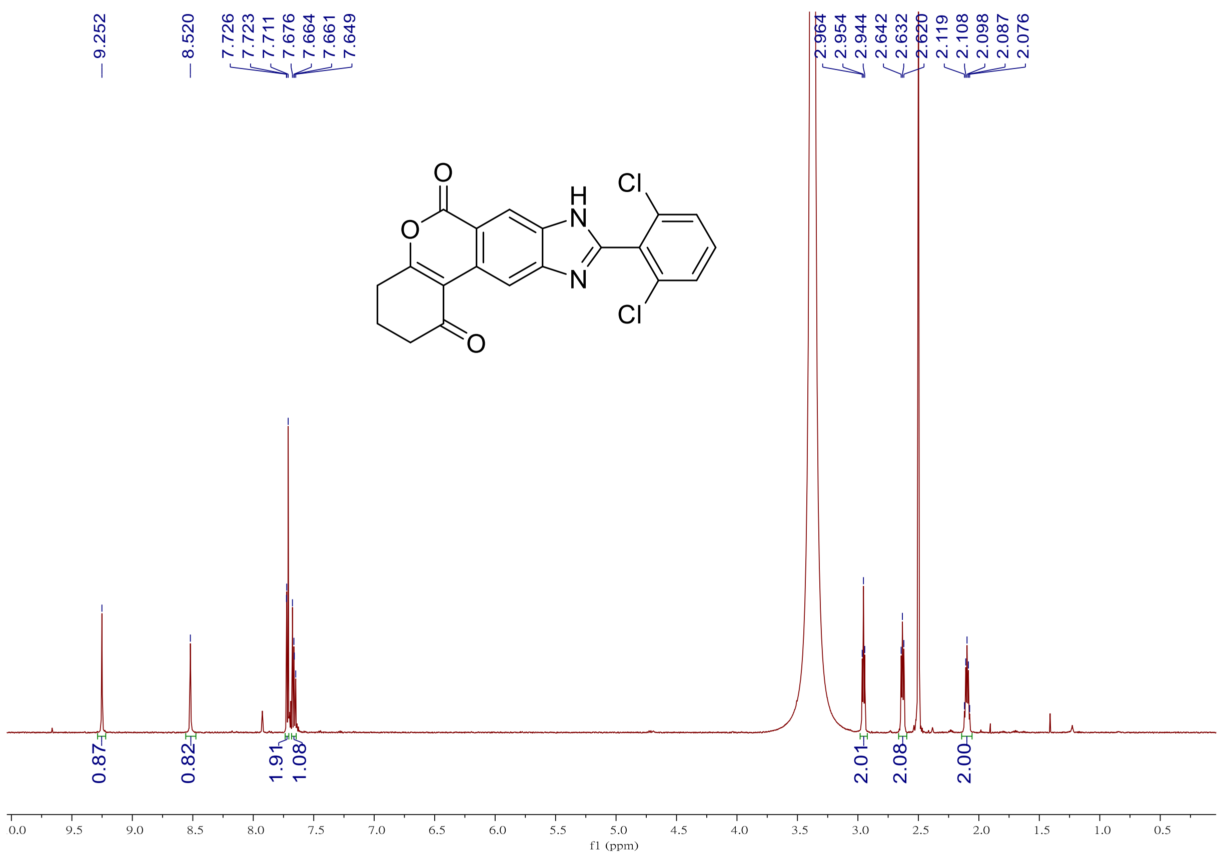

**Figure S193.** $^{1}$H NMR spectrum of compound **4i** (600 MHz, $(CD_3)_2SO$).

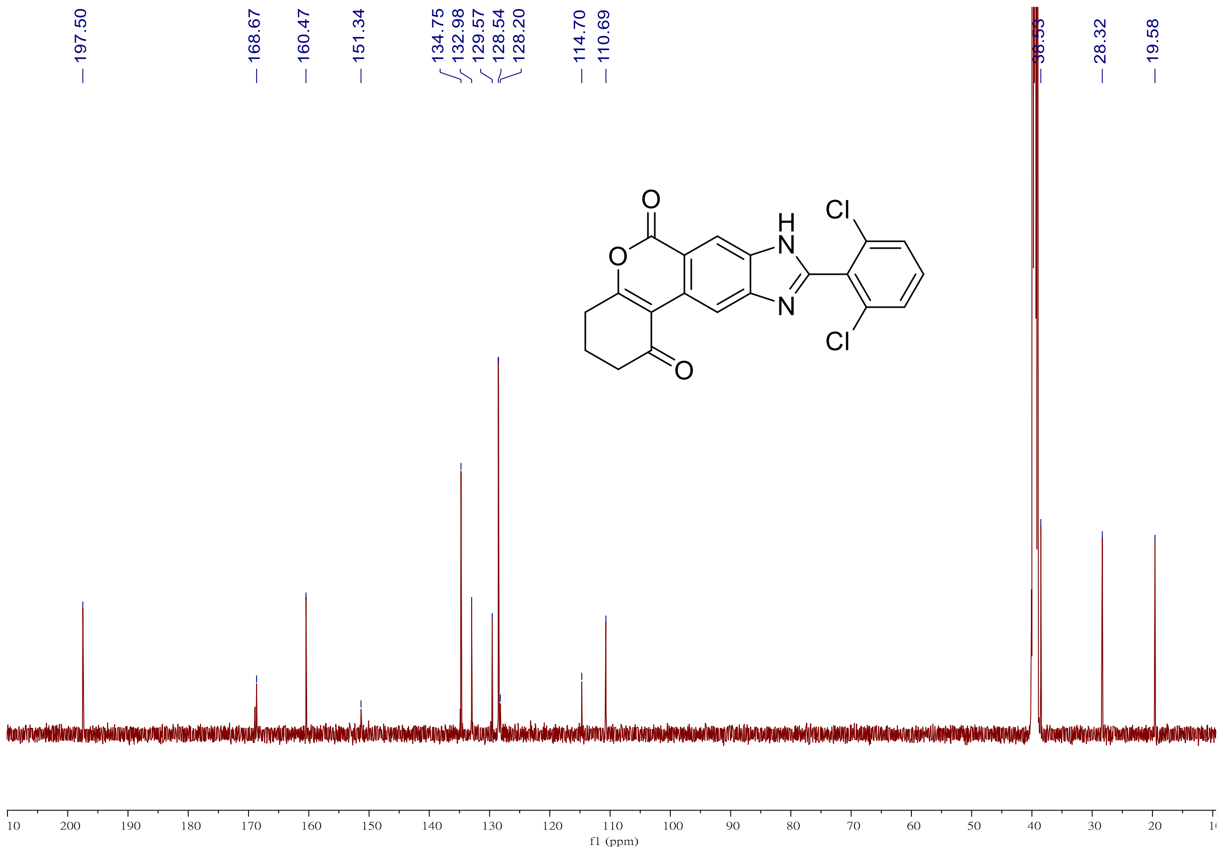

**Figure S194.** $^{13}C\{^{1}H\}$ NMR spectrum of compound **4i** (151 MHz, $(CD_3)_2SO$).

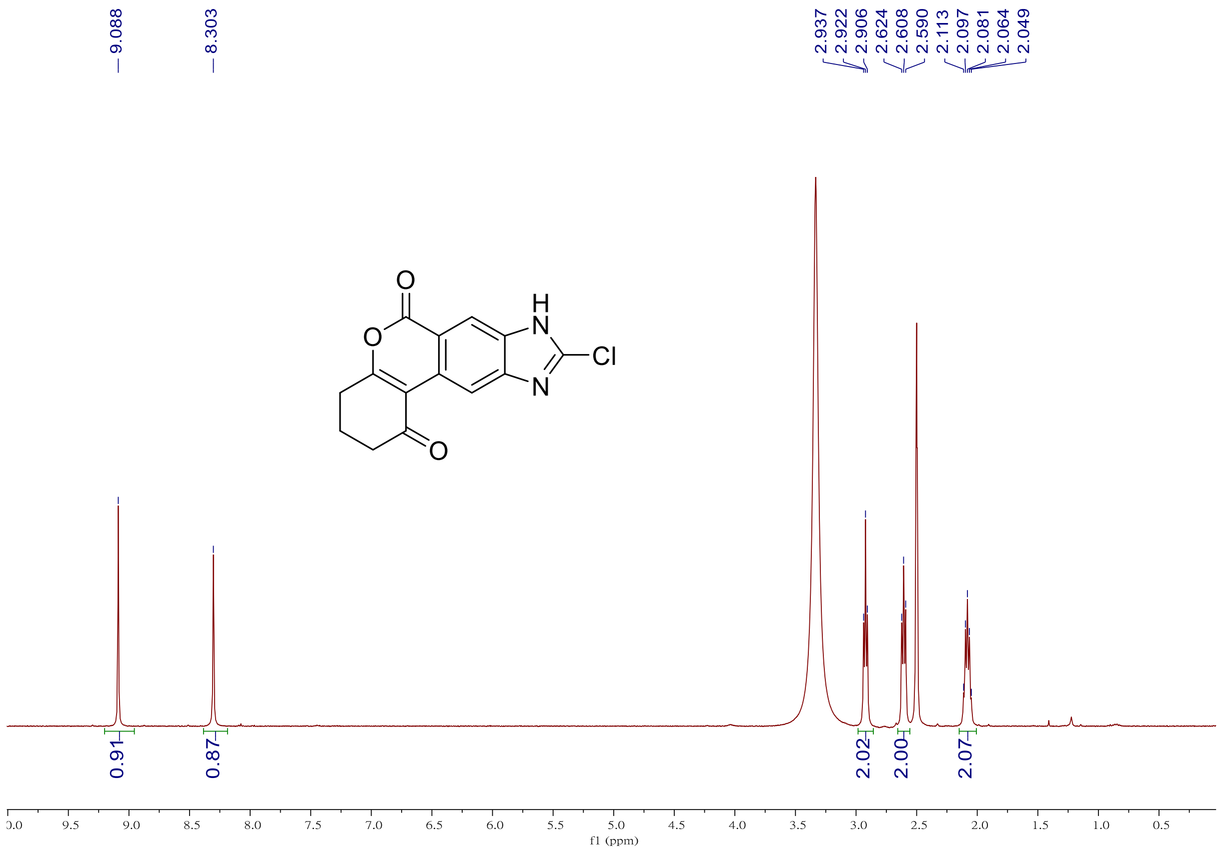


**Figure S195.** $^{1}$H NMR spectrum of compound **4j** (400 MHz, $(CD_3)_2SO$).

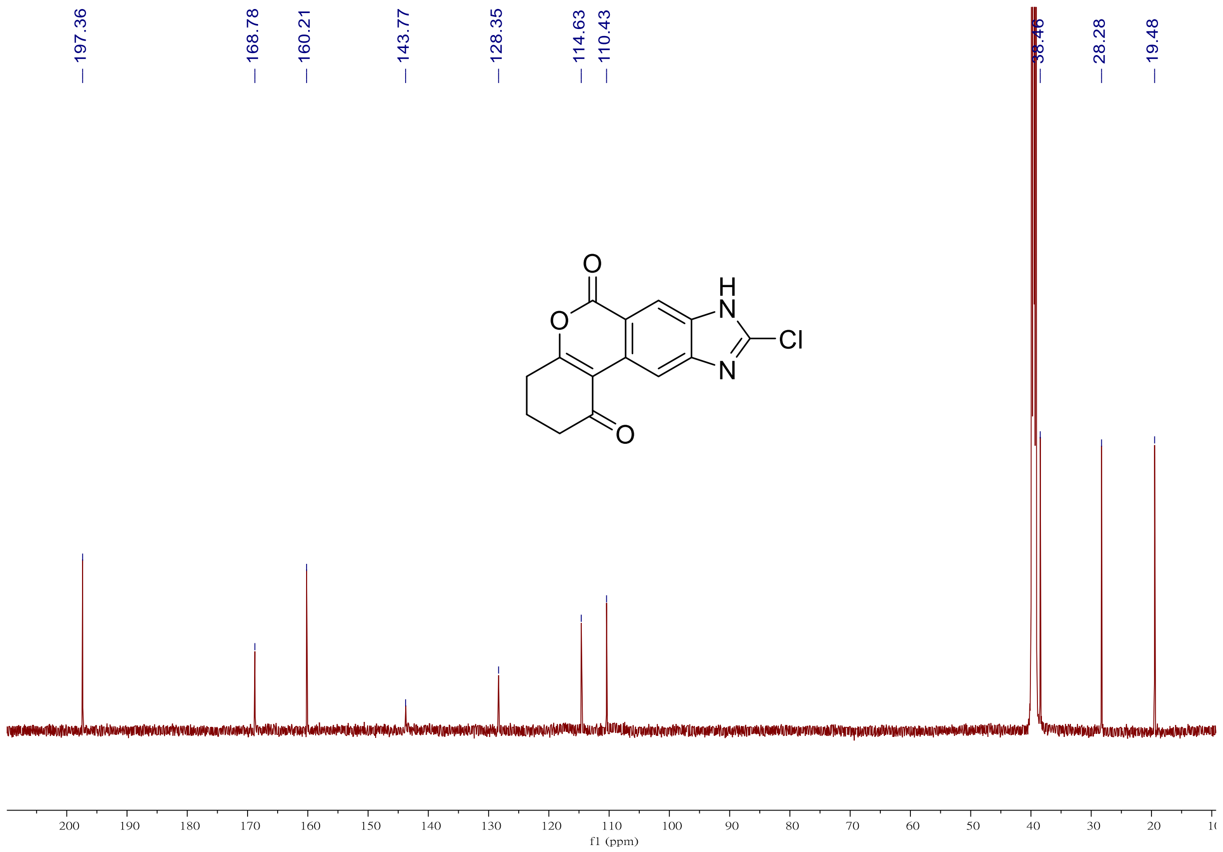


**Figure S196.** $^{13}C\{^{1}H\}$ NMR spectrum of compound **4j** (151 MHz, $(CD_3)_2SO$).

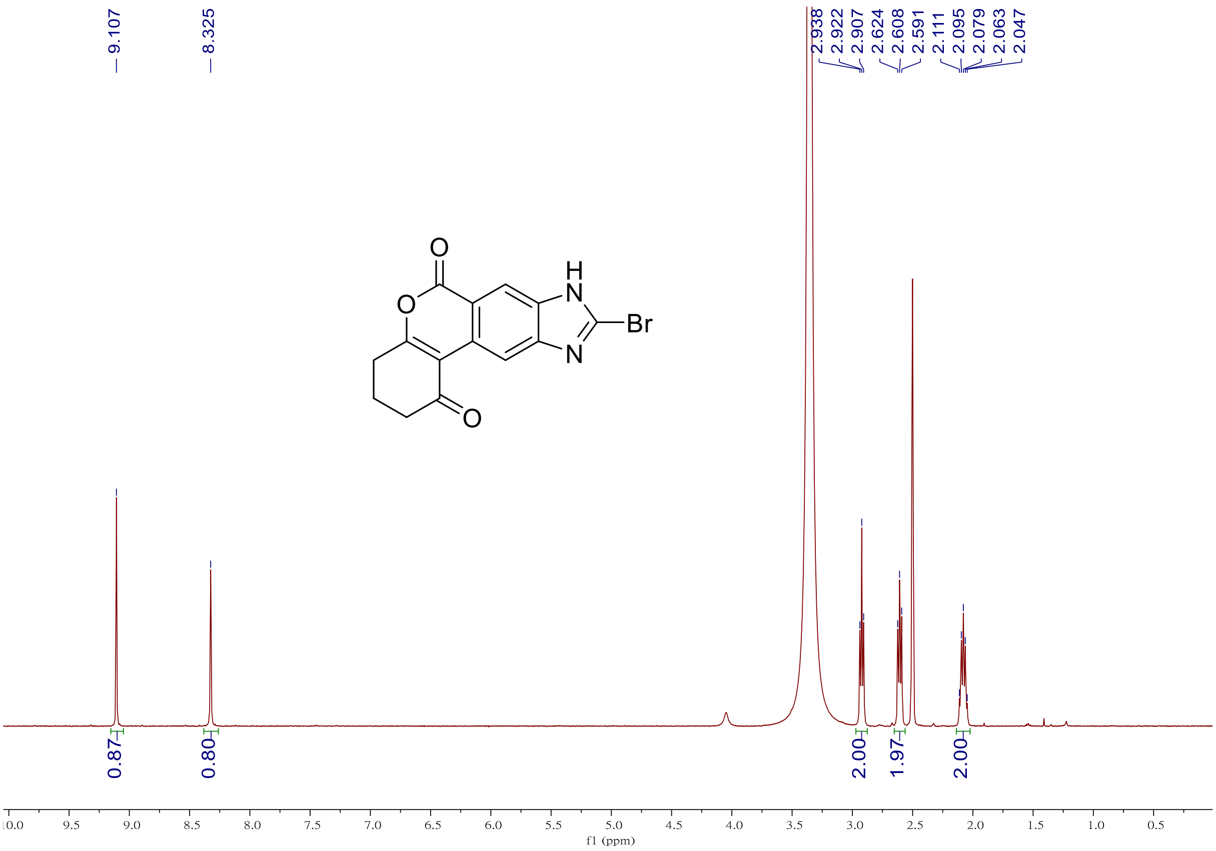


**Figure S197.** $^{1}$H NMR spectrum of compound **4k** (400 MHz, $(CD_3)_2SO$).

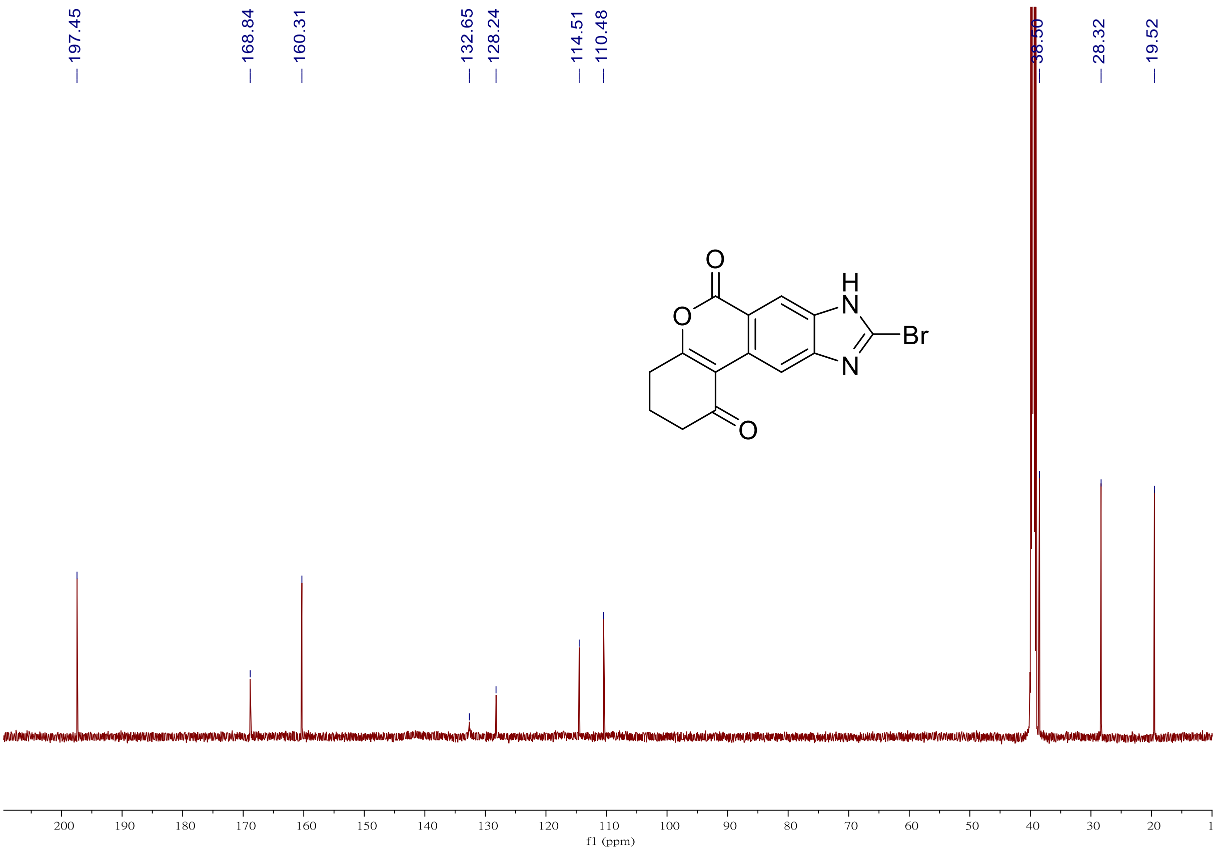


**Figure S198.** $^{13}C\{^{1}H\}$ NMR spectrum of compound **4k** (151 MHz, $(CD_3)_2SO$).

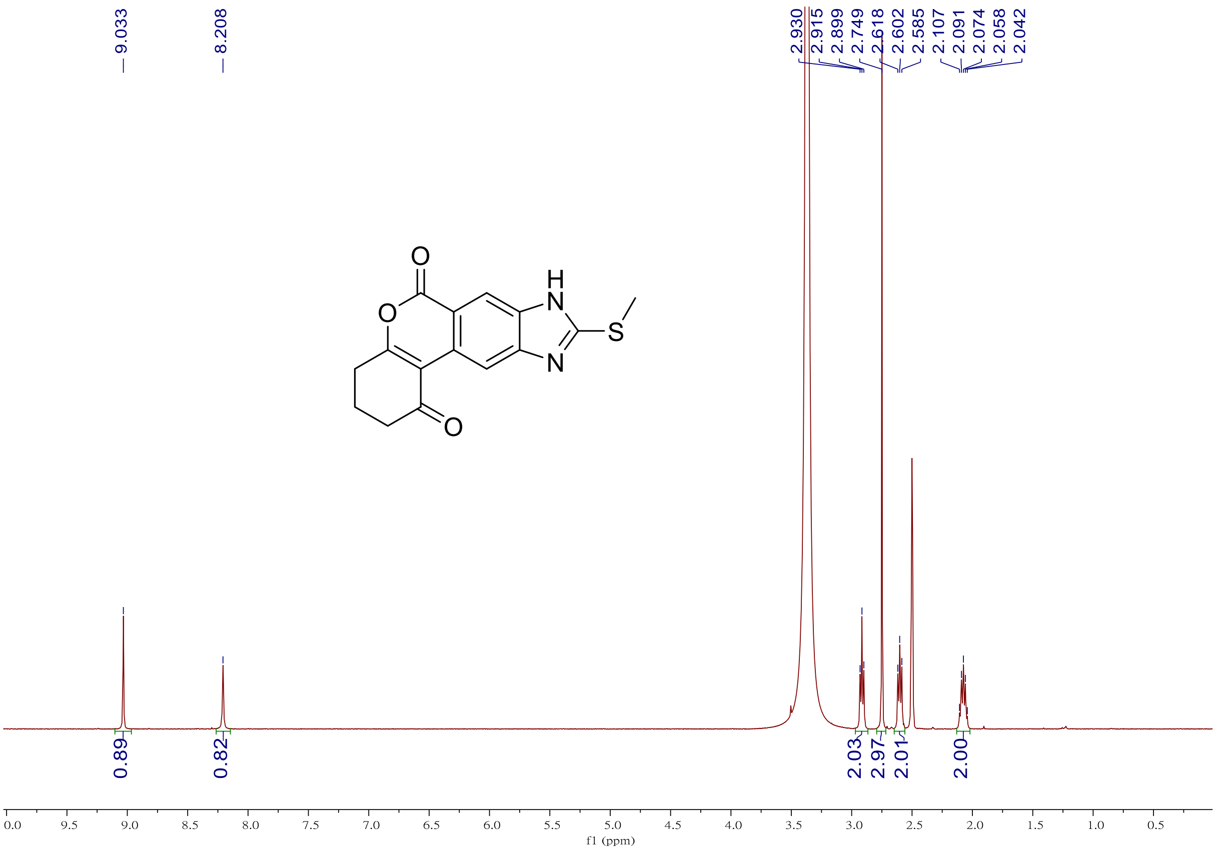


**Figure S199.** $^{1}H$ NMR spectrum of compound **4l** (400 MHz, $(CD_3)_2SO$).

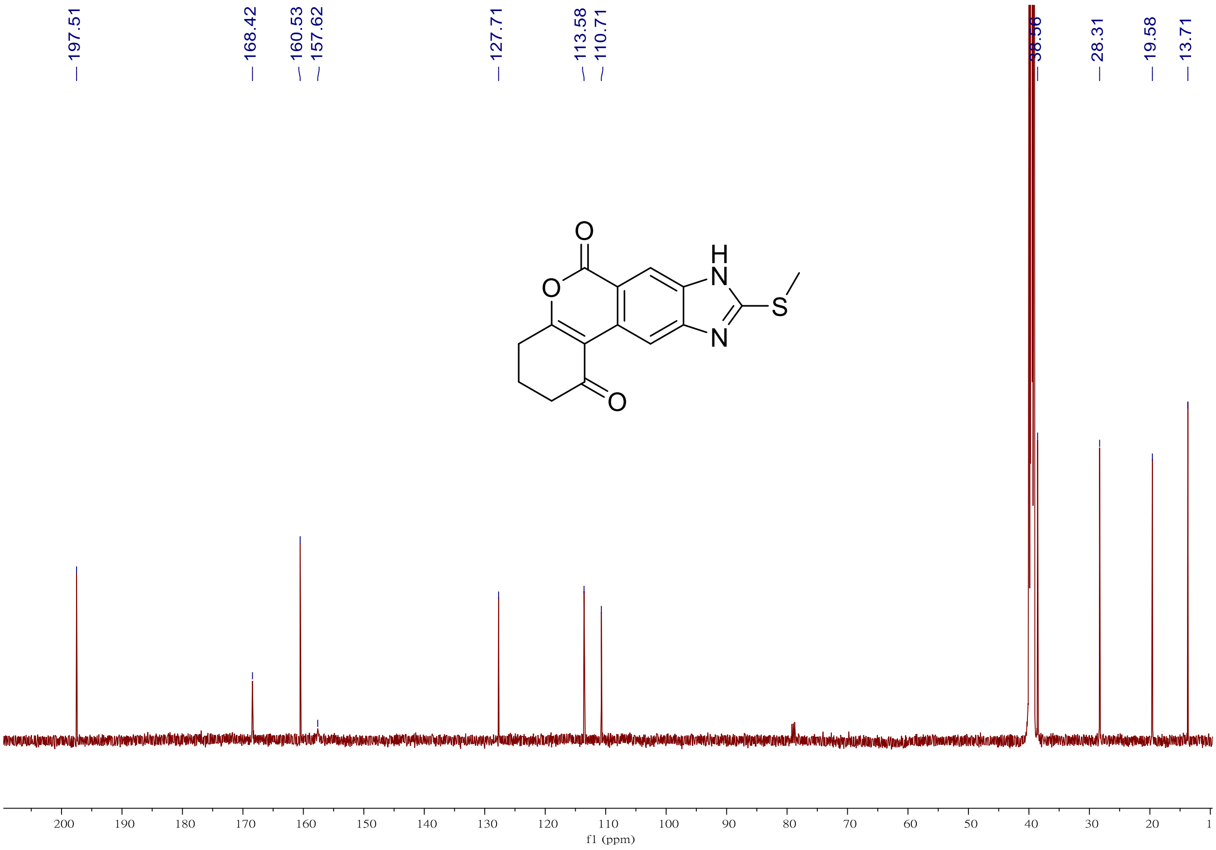


**Figure S200.** $^{13}C\{^{1}H\}$ NMR spectrum of compound **4l** (151 MHz, $(CD_3)_2SO$).

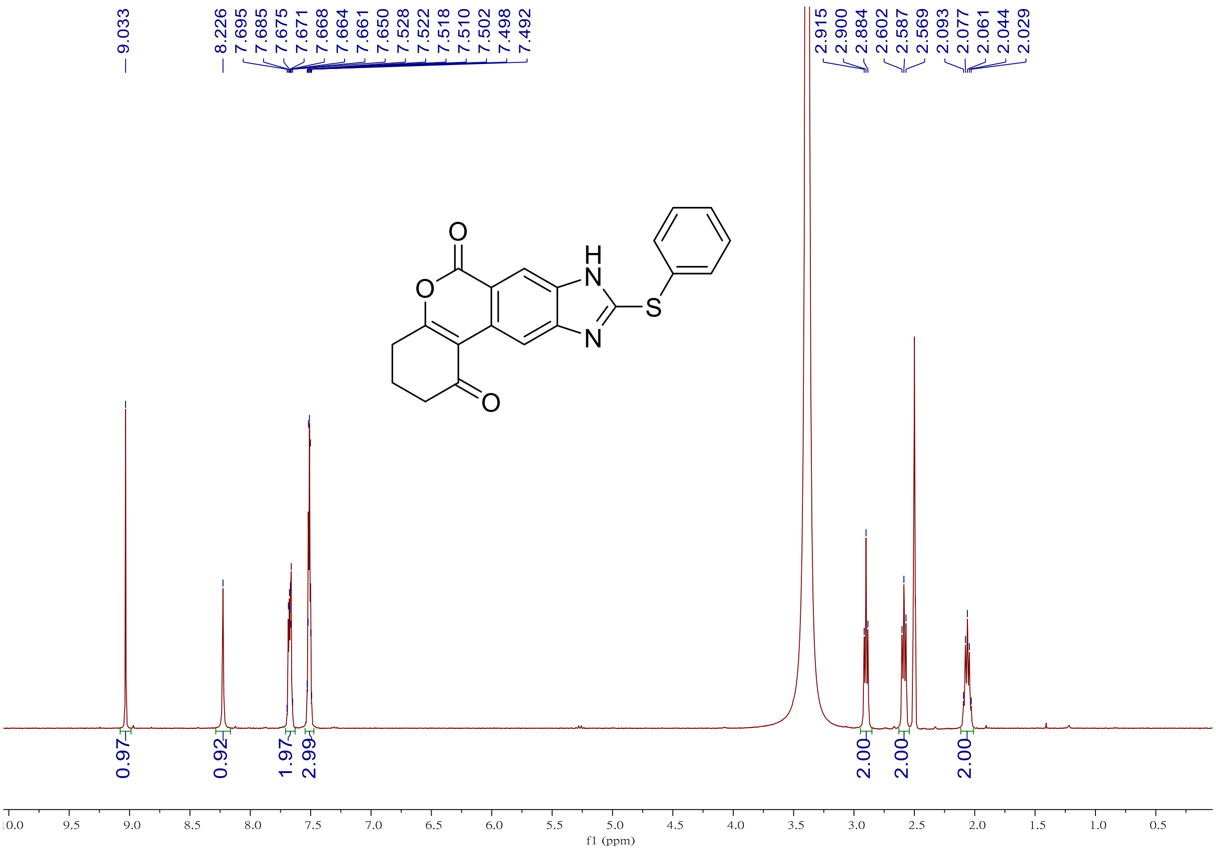


**Figure S201.** $^{1}$H NMR spectrum of compound **4m** (400 MHz, $(CD_3)_2SO$).

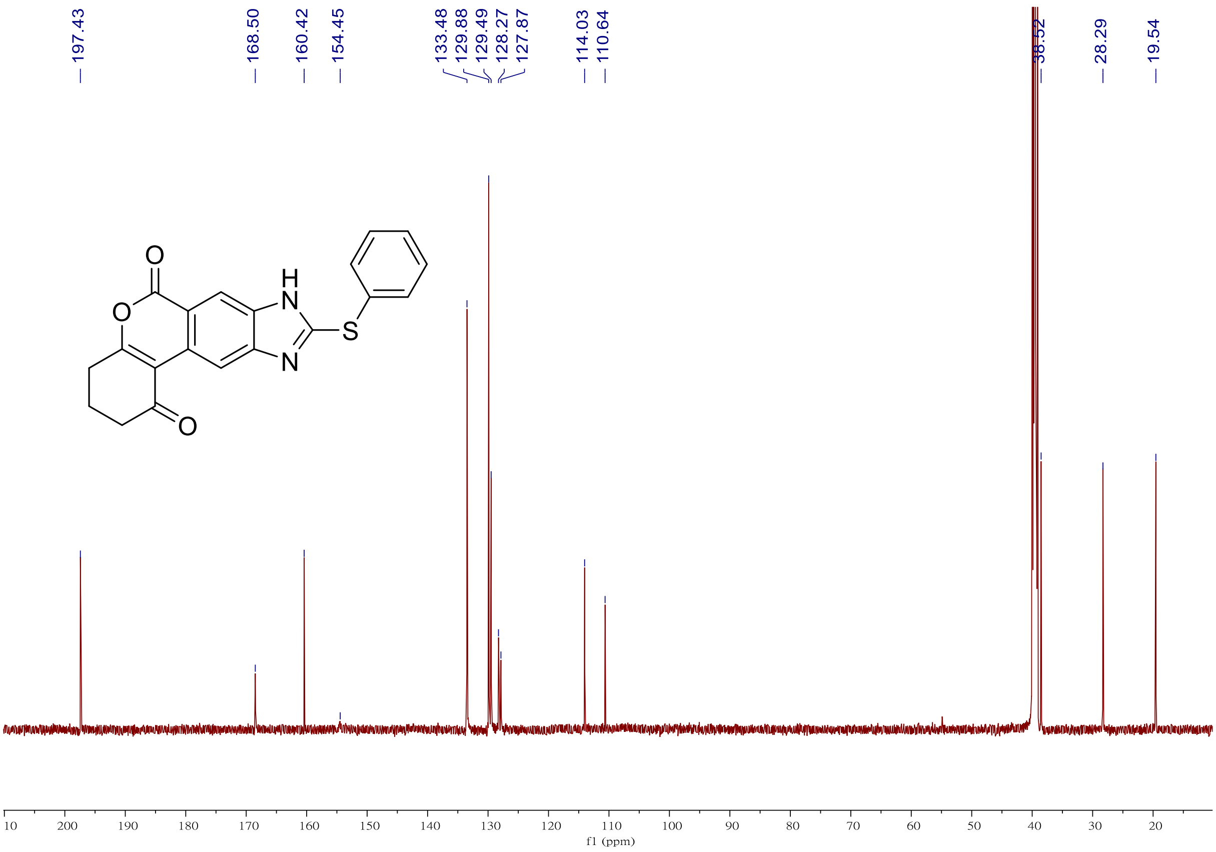


**Figure S202.** $^{13}C\{^{1}H\}$ NMR spectrum of compound **4m** (151 MHz, $(CD_3)_2SO$).

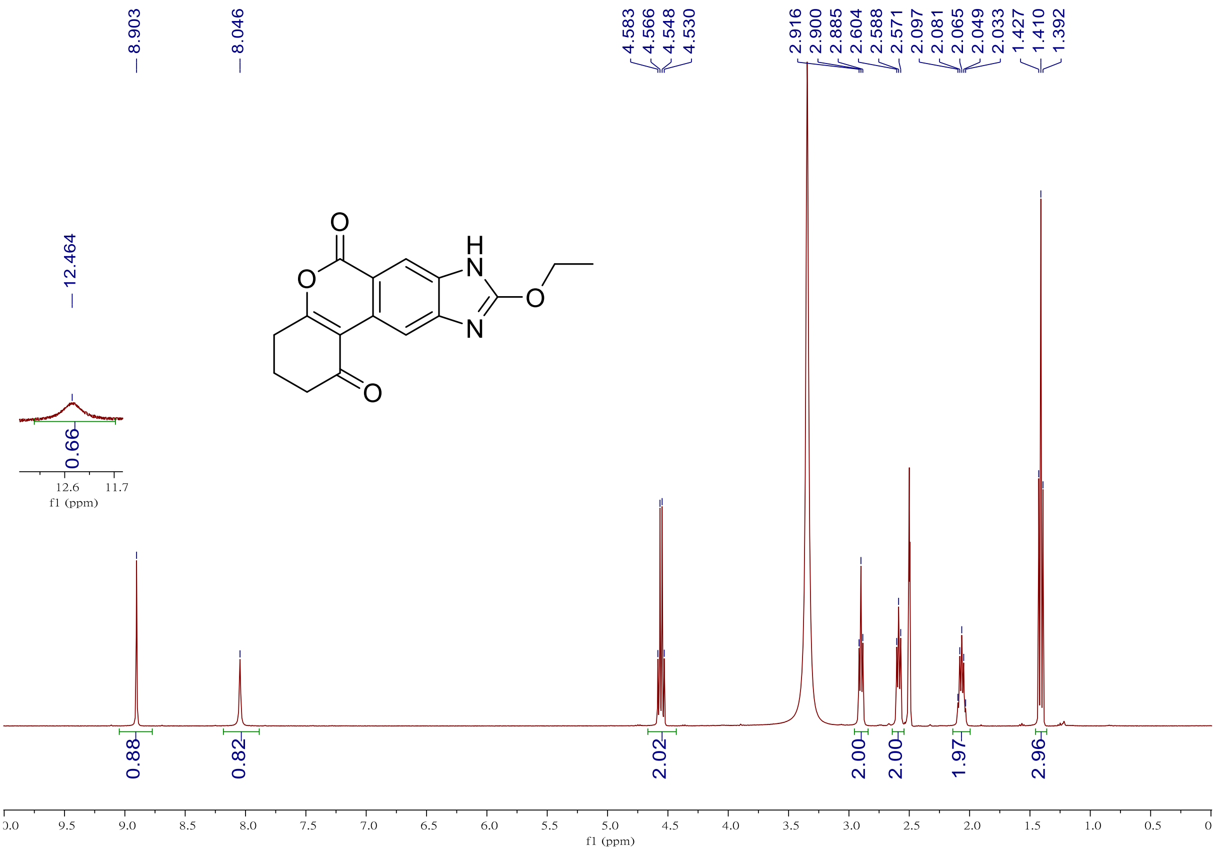


**Figure S203.** $^{1}$H NMR spectrum of compound **4n** (400 MHz, $(CD_3)_2SO$).

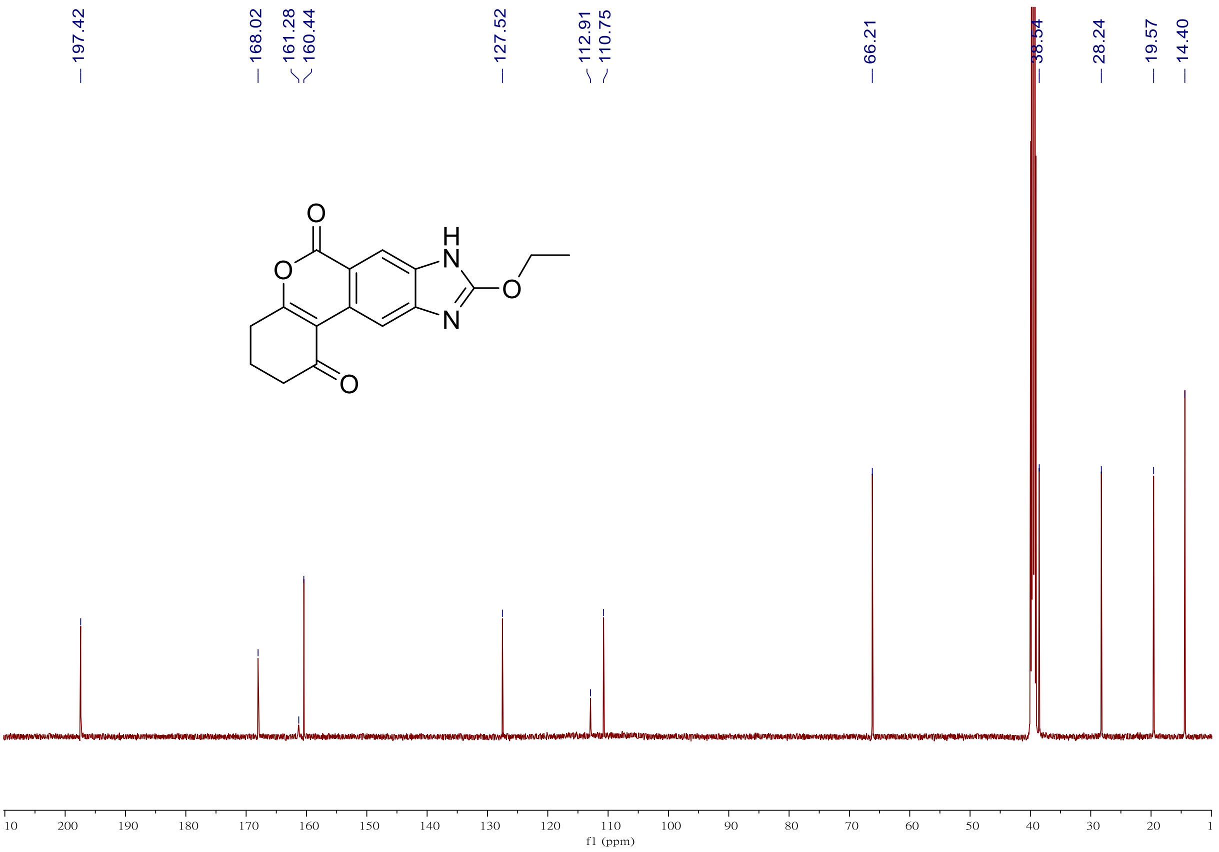


**Figure S204.** $^{13}$C{$^{1}$H} NMR spectrum of compound **4n** (151 MHz, $(CD_3)_2SO$).

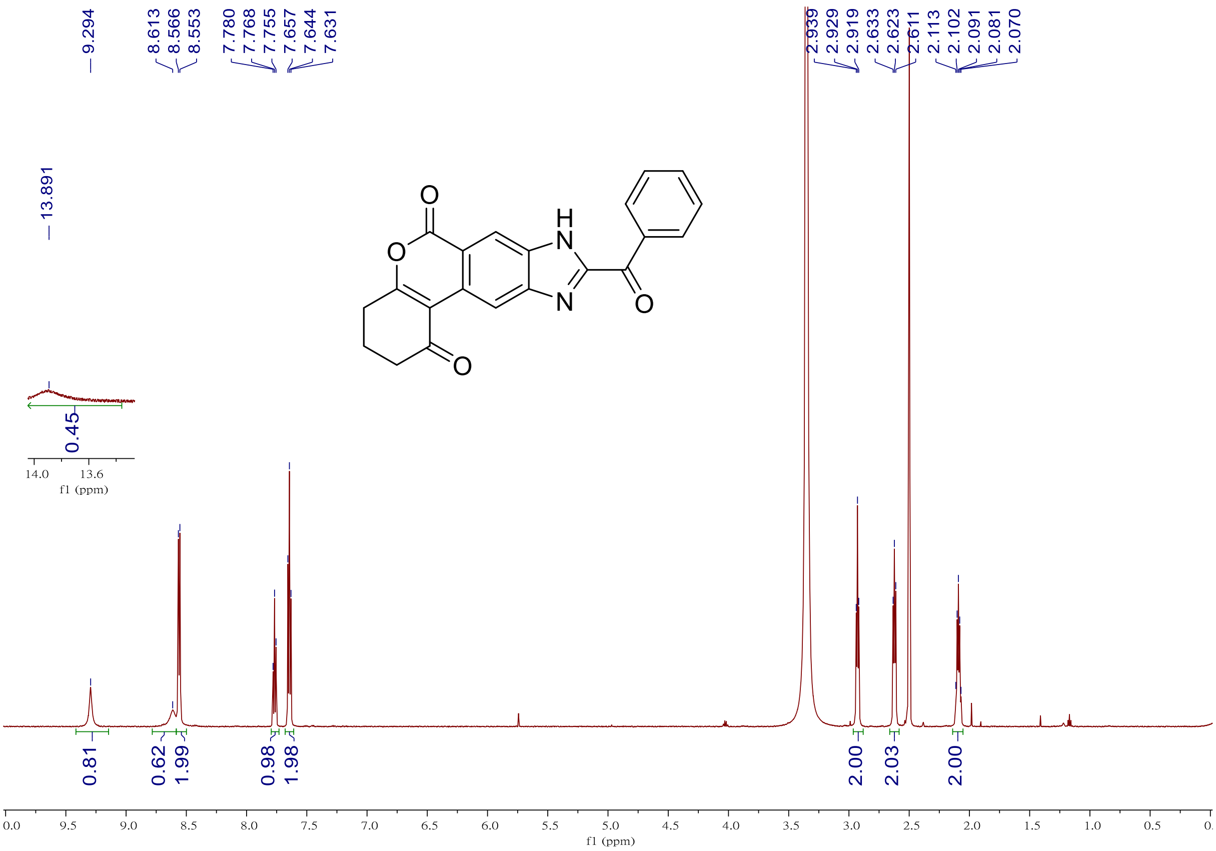


**Figure S205.** $^{1}$H NMR spectrum of compound **4o** (600 MHz, $(CD_3)_2SO$).

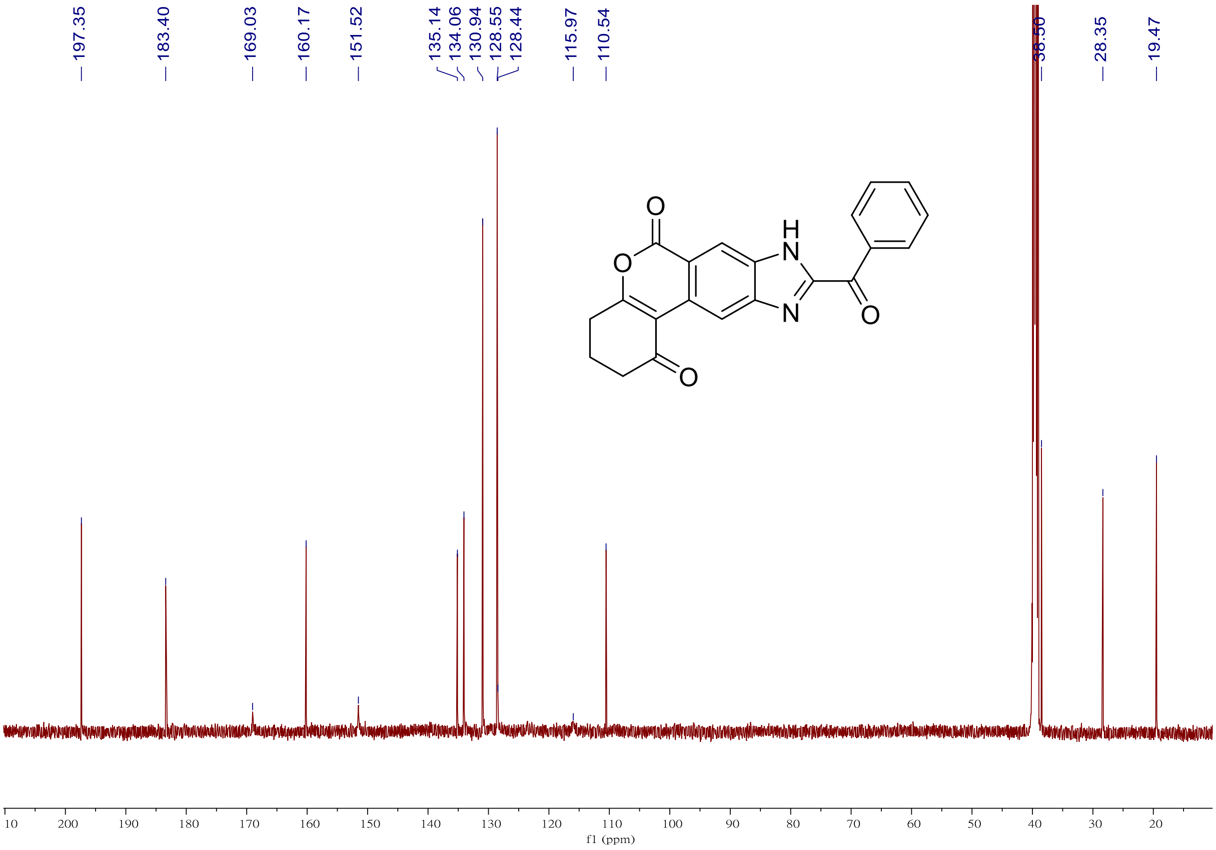


**Figure S206.** $^{13}$C{$^{1}$H} NMR spectrum of compound **4o** (151 MHz, $(CD_3)_2SO$).

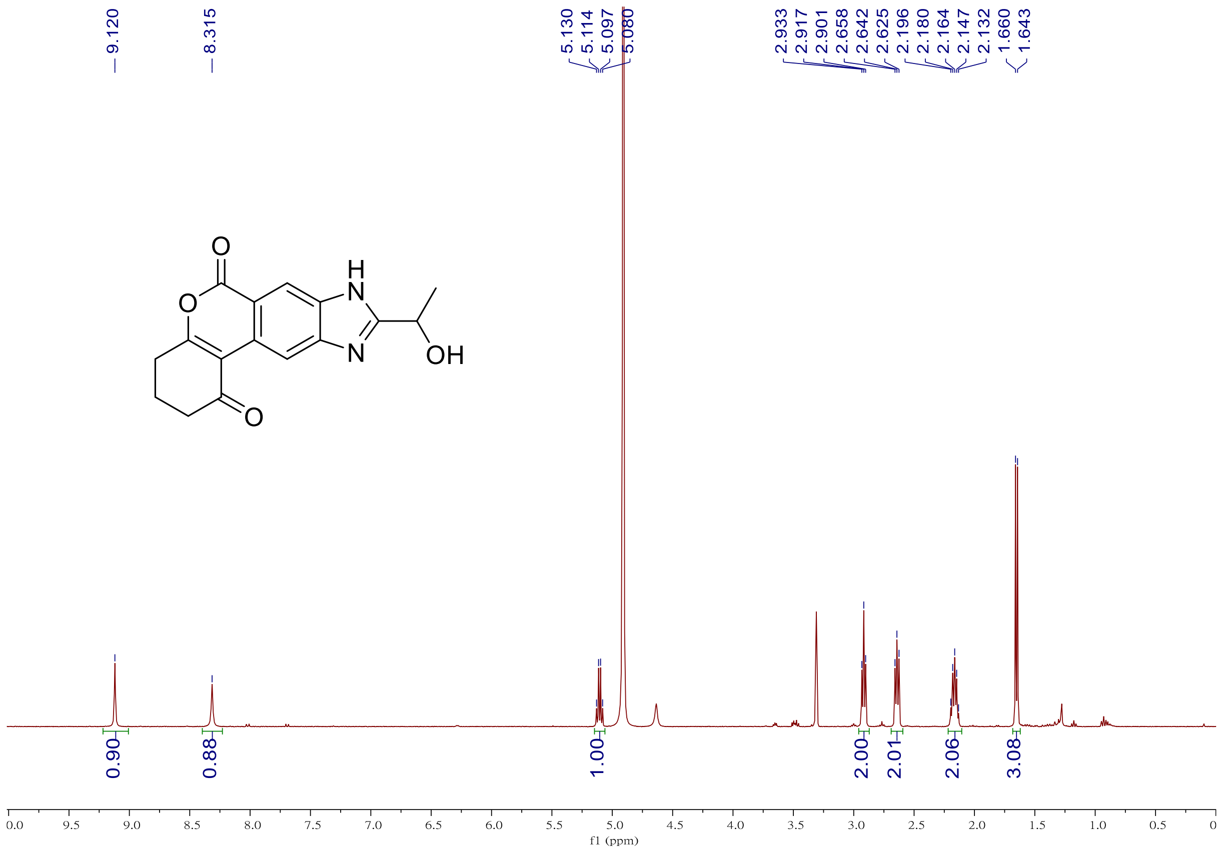


**Figure S207.** $^{1}$H NMR spectrum of compound **4p** (400 MHz, $CD_3OD$).

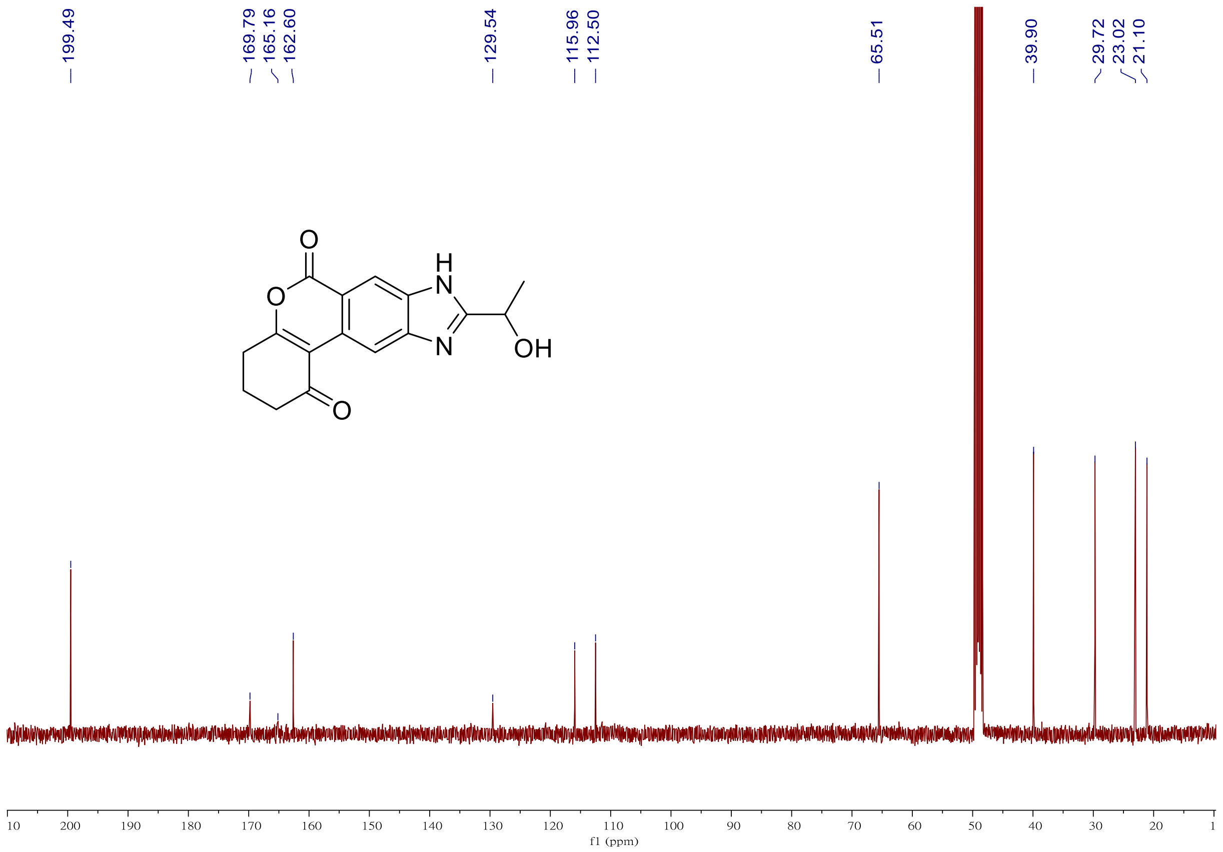


**Figure S208.** $^{13}C\{^{1}H\}$ NMR spectrum of compound **4p** (101 MHz, $CD_3OD$).

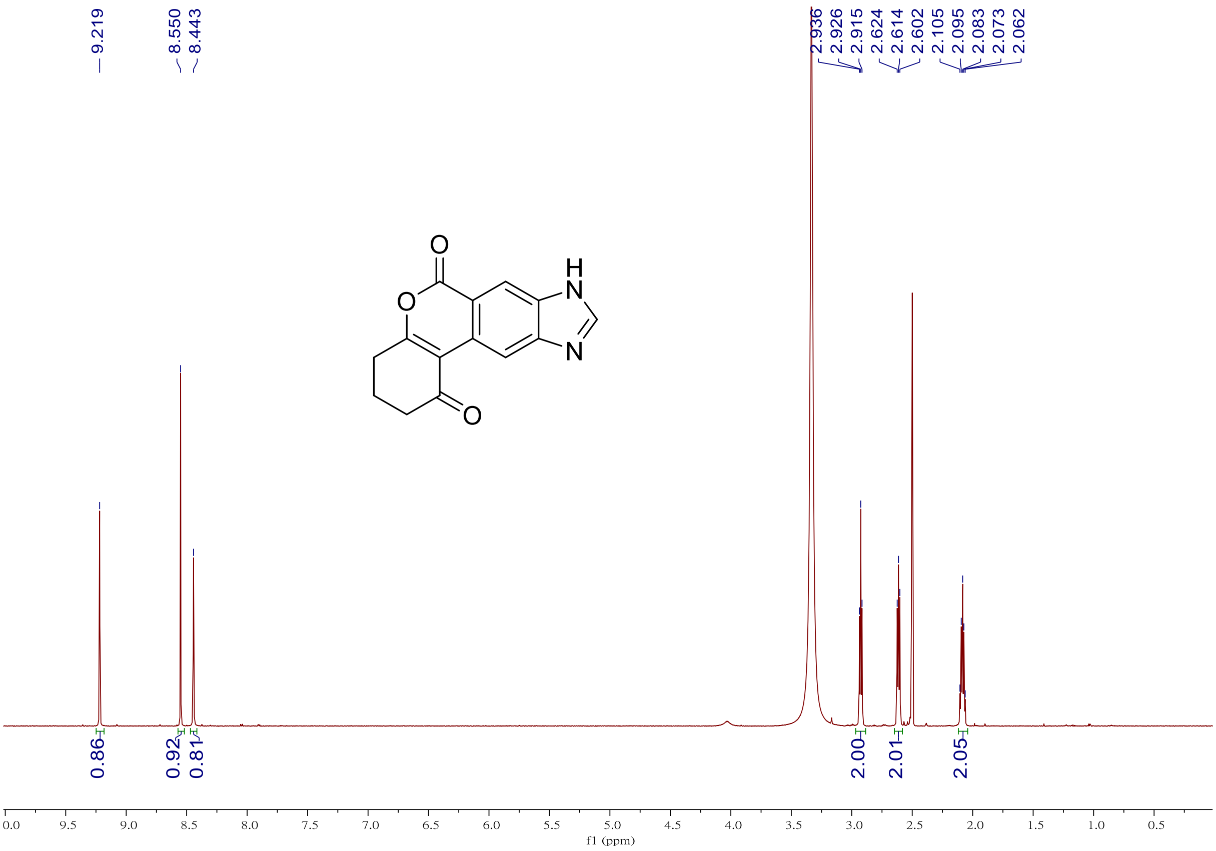


**Figure S209.** $^{1}$H NMR spectrum of compound **4q** (600 MHz, $(CD_3)_2SO$).

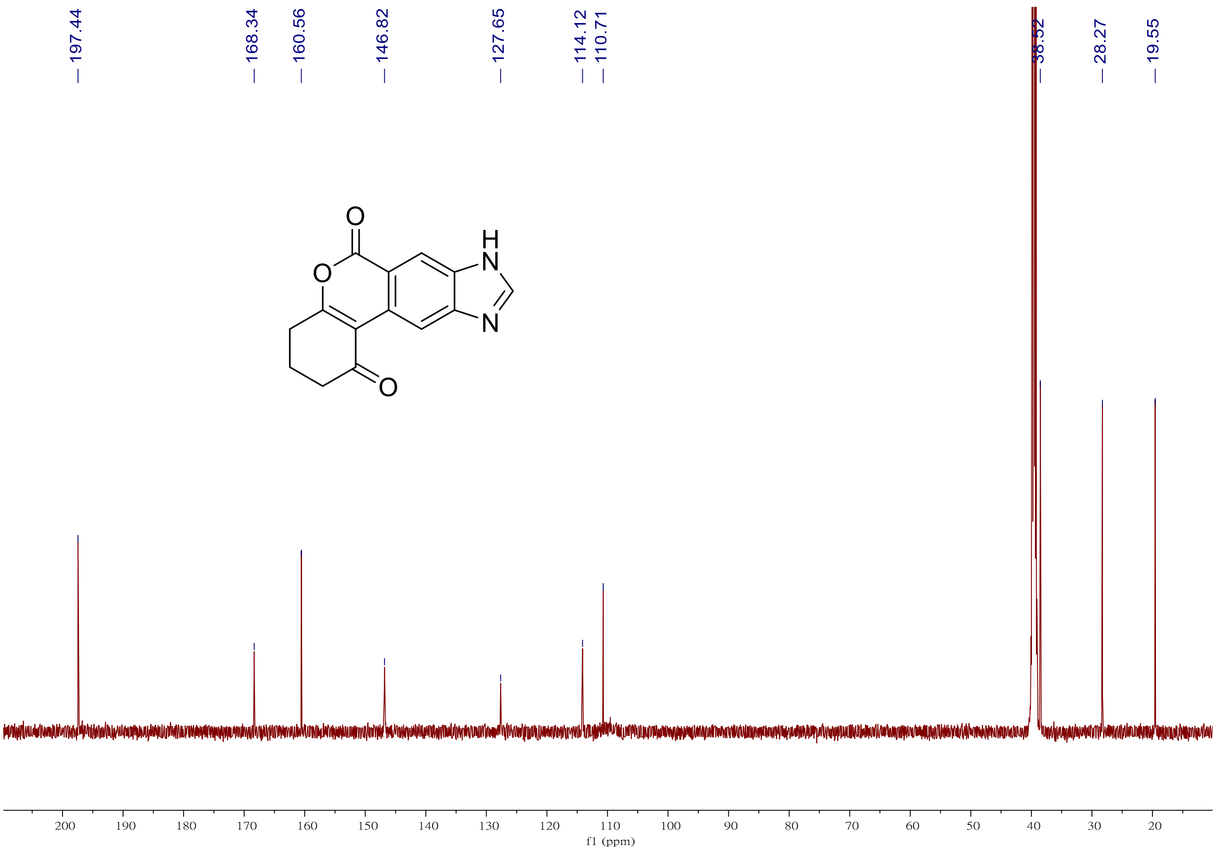


**Figure S210.** $^{13}C\{^{1}H\}$ NMR spectrum of compound **4q** (151 MHz, $(CD_3)_2SO$).

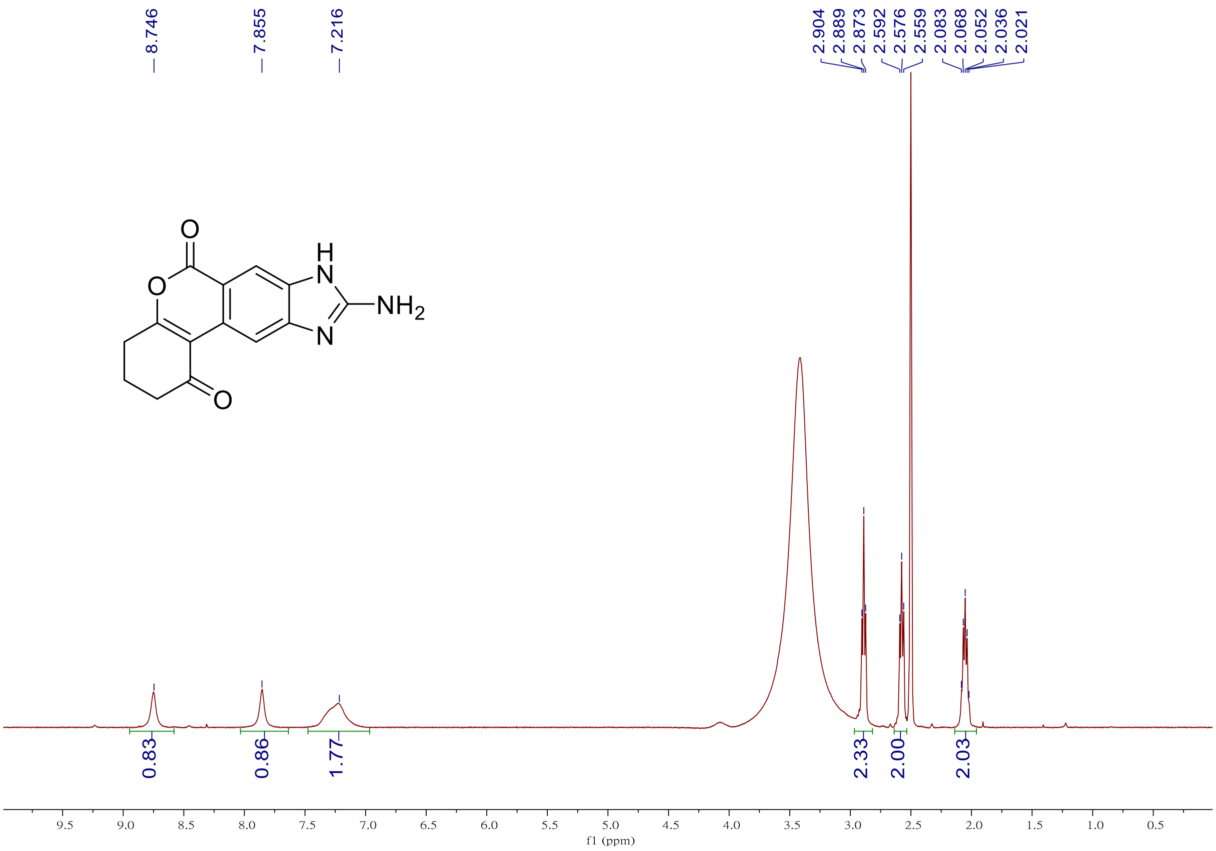


**Figure S211.** $^{1}$H NMR spectrum of compound **4r** (400 MHz, $(CD_3)_2SO$).

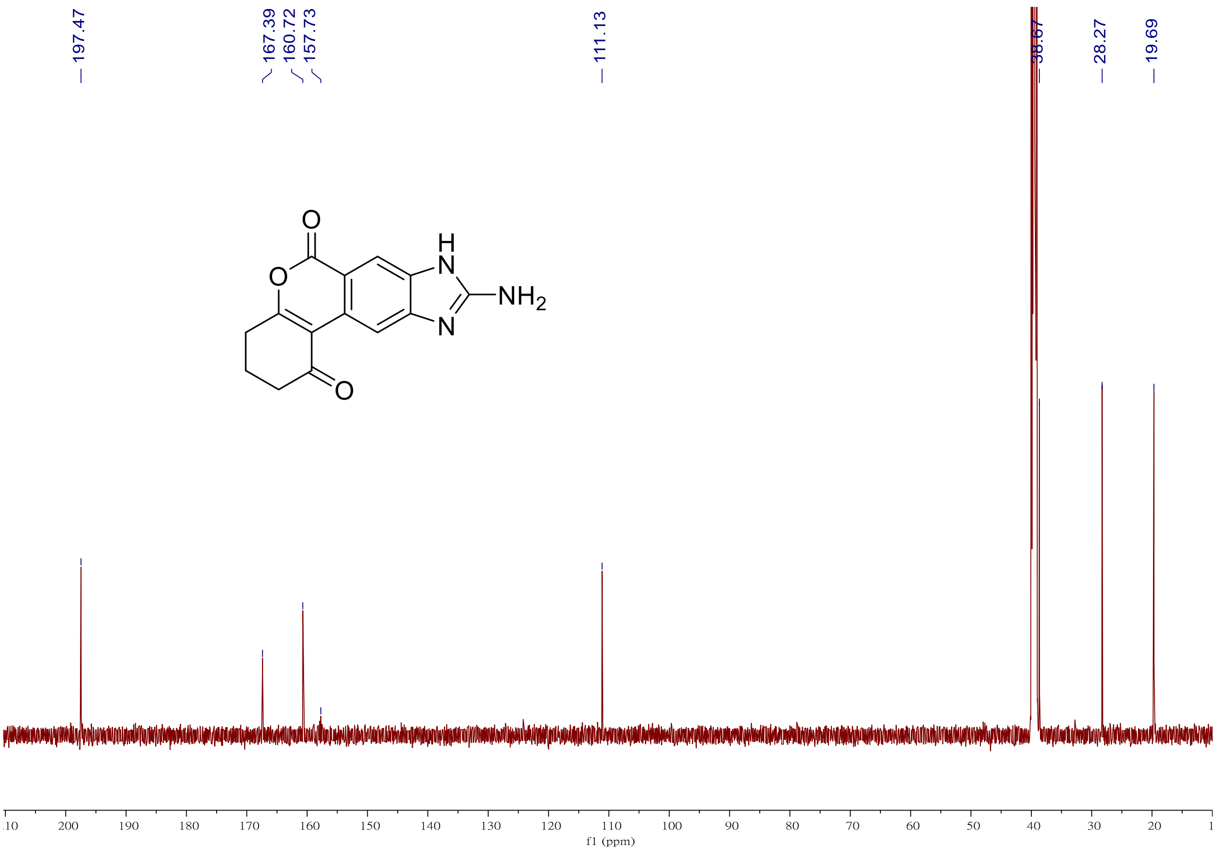


**Figure S212.** $^{13}C\{^{1}H\}$ NMR spectrum of compound **4r** (151 MHz, $(CD_3)_2SO$).

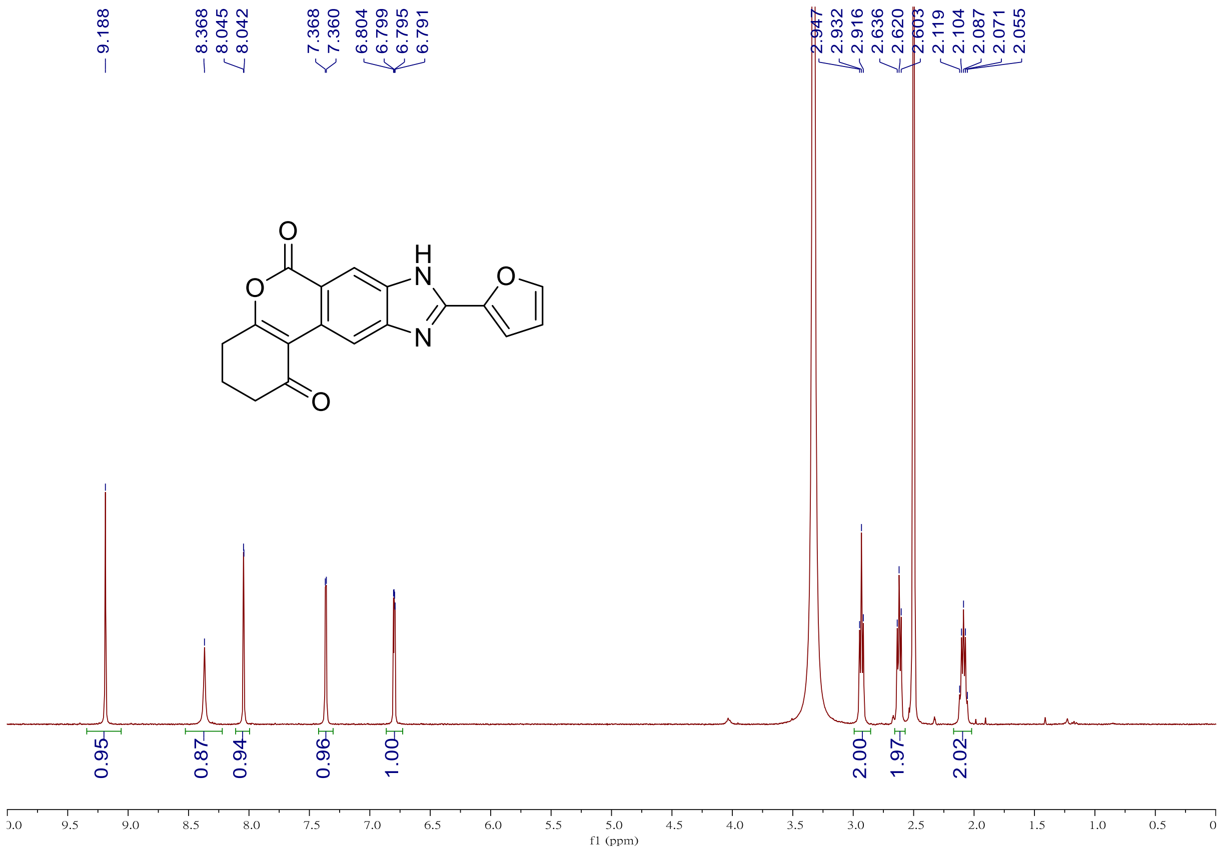


**Figure S213.** $^{1}$H NMR spectrum of compound **4s** (400 MHz, $(CD_3)_2SO$).

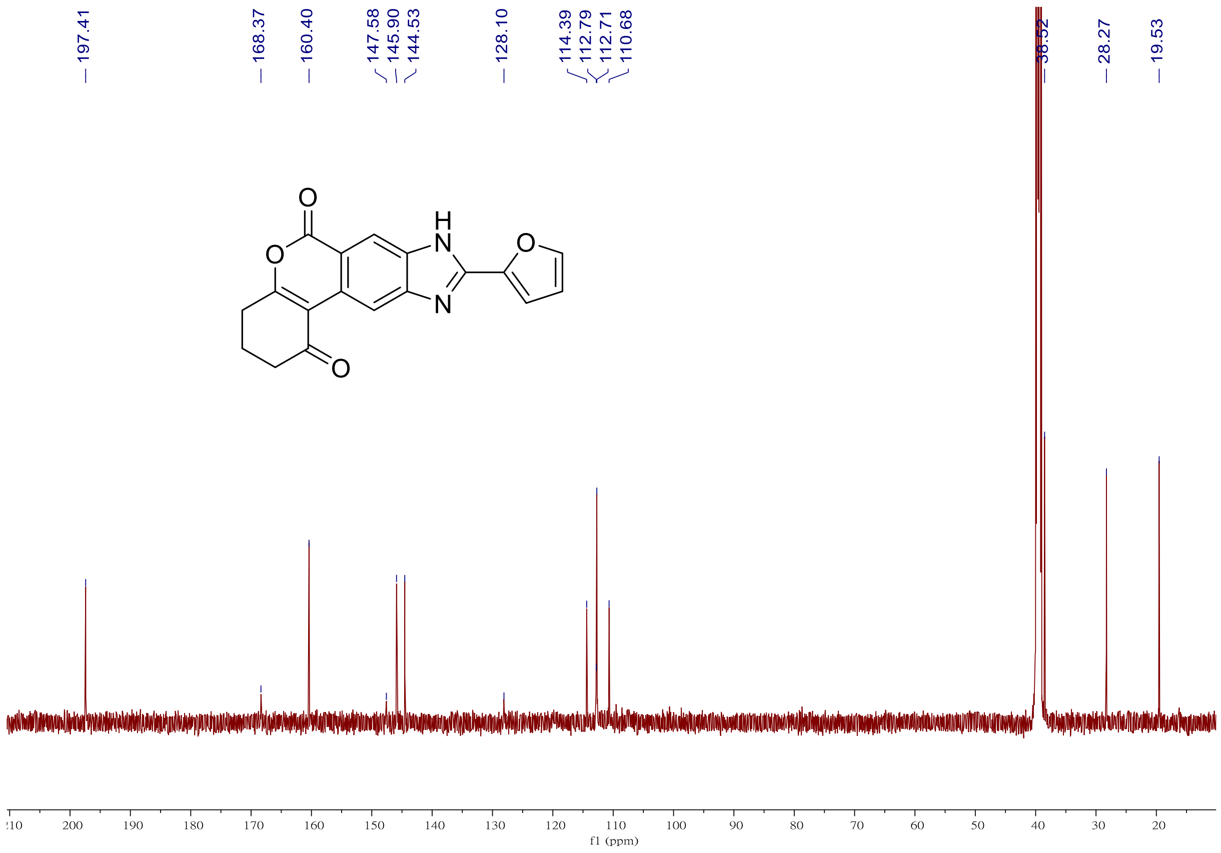


**Figure S214.** $^{13}C\{^{1}H\}$ NMR spectrum of compound **4s** (151 MHz, $(CD_3)_2SO$).

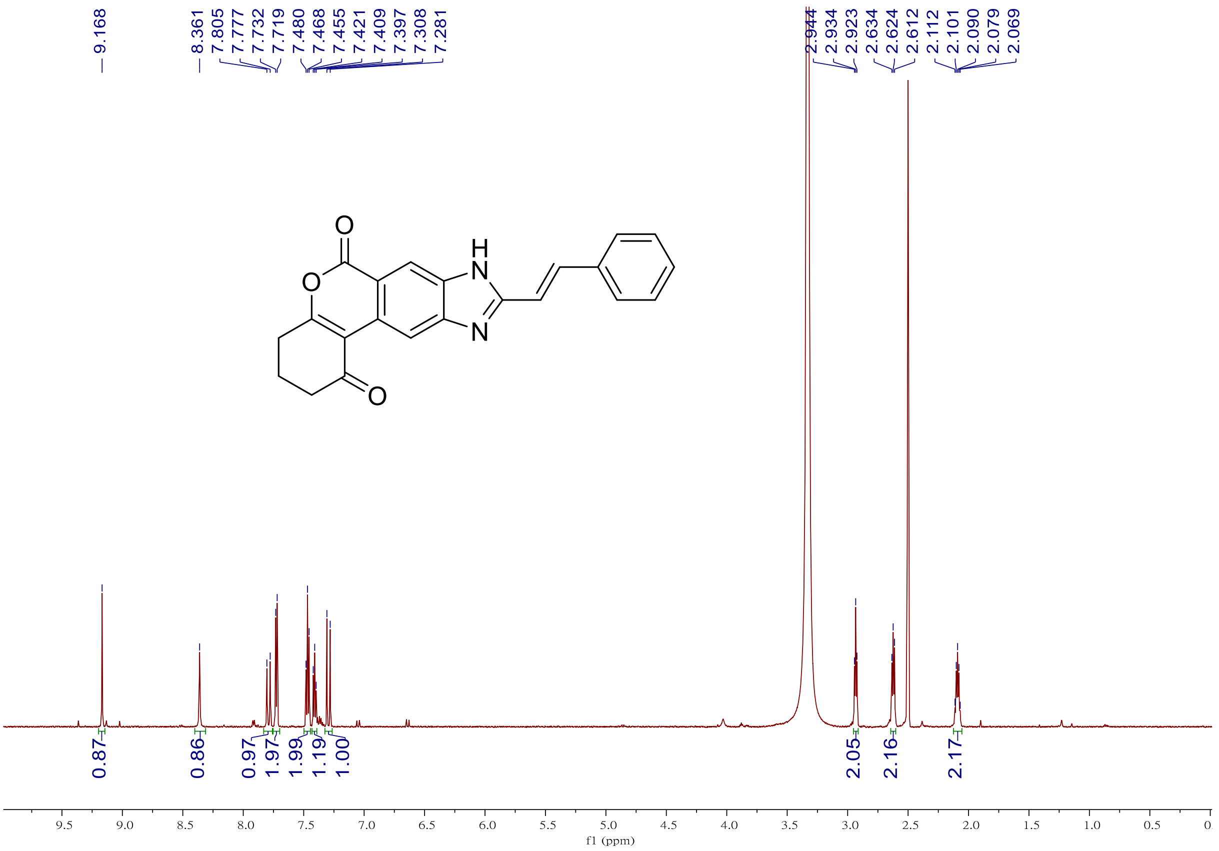


**Figure S215.** $^{1}H$ NMR spectrum of compound **4t** (600 MHz, $(CD_3)_2SO$).

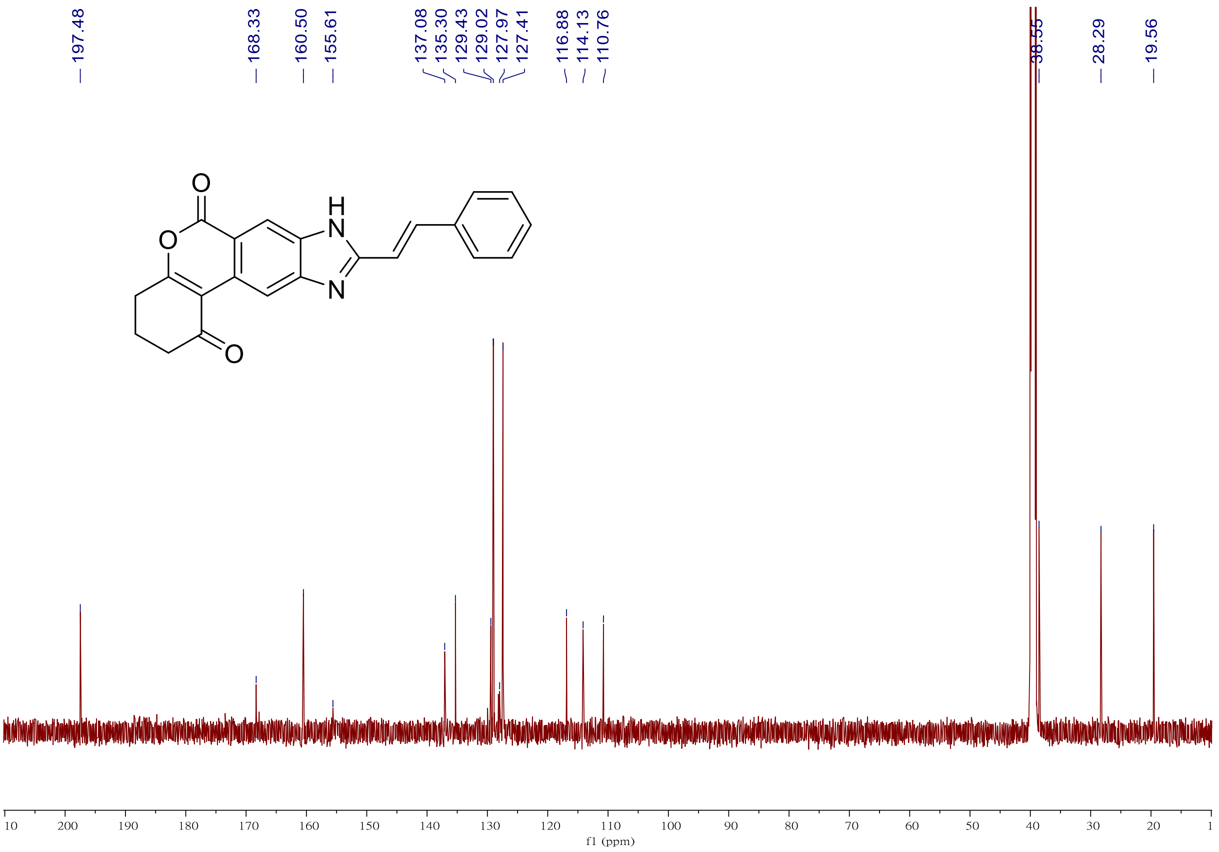


**Figure S216.** $^{13}C\{^{1}H\}$ NMR spectrum of compound **4t** (151 MHz, $(CD_3)_2SO$).

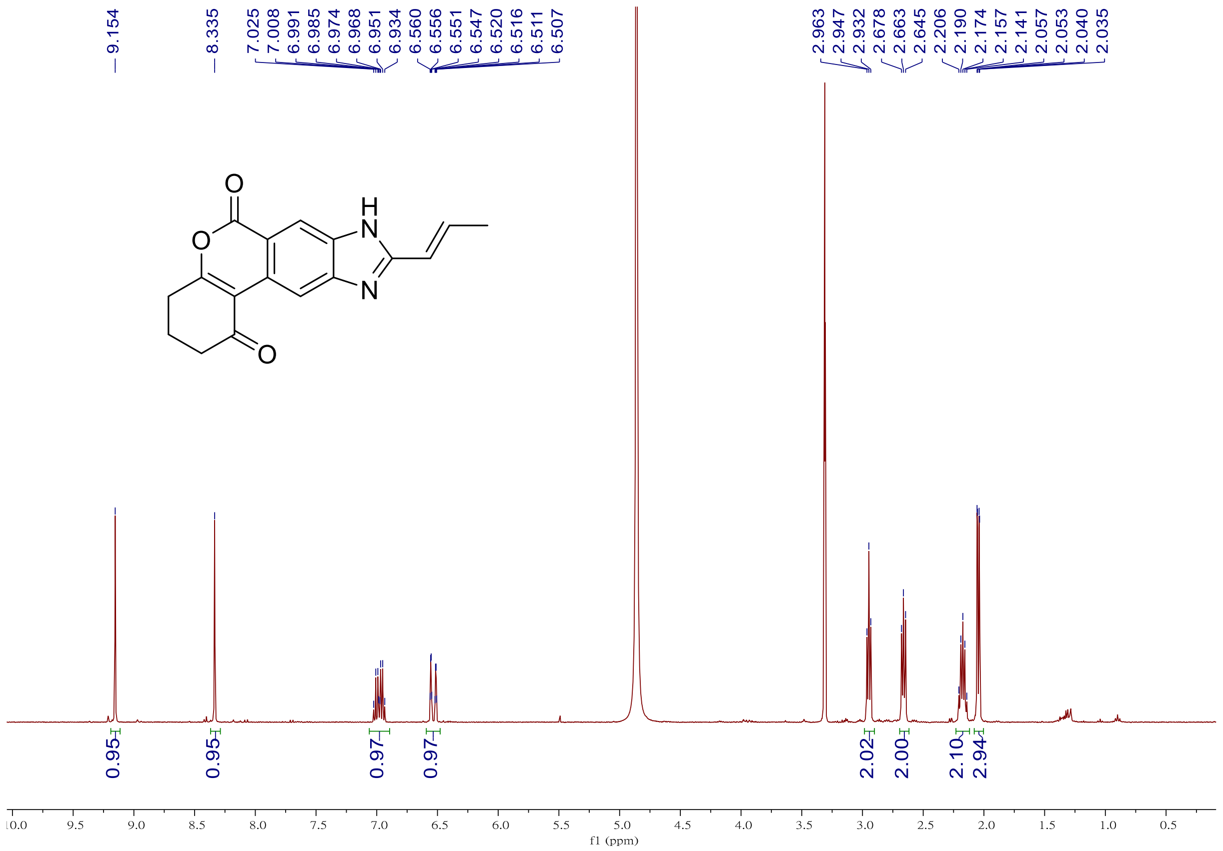


**Figure S217.** $^{1}H$ NMR spectrum of compound **4u** (400 MHz, $CD_3OD$).

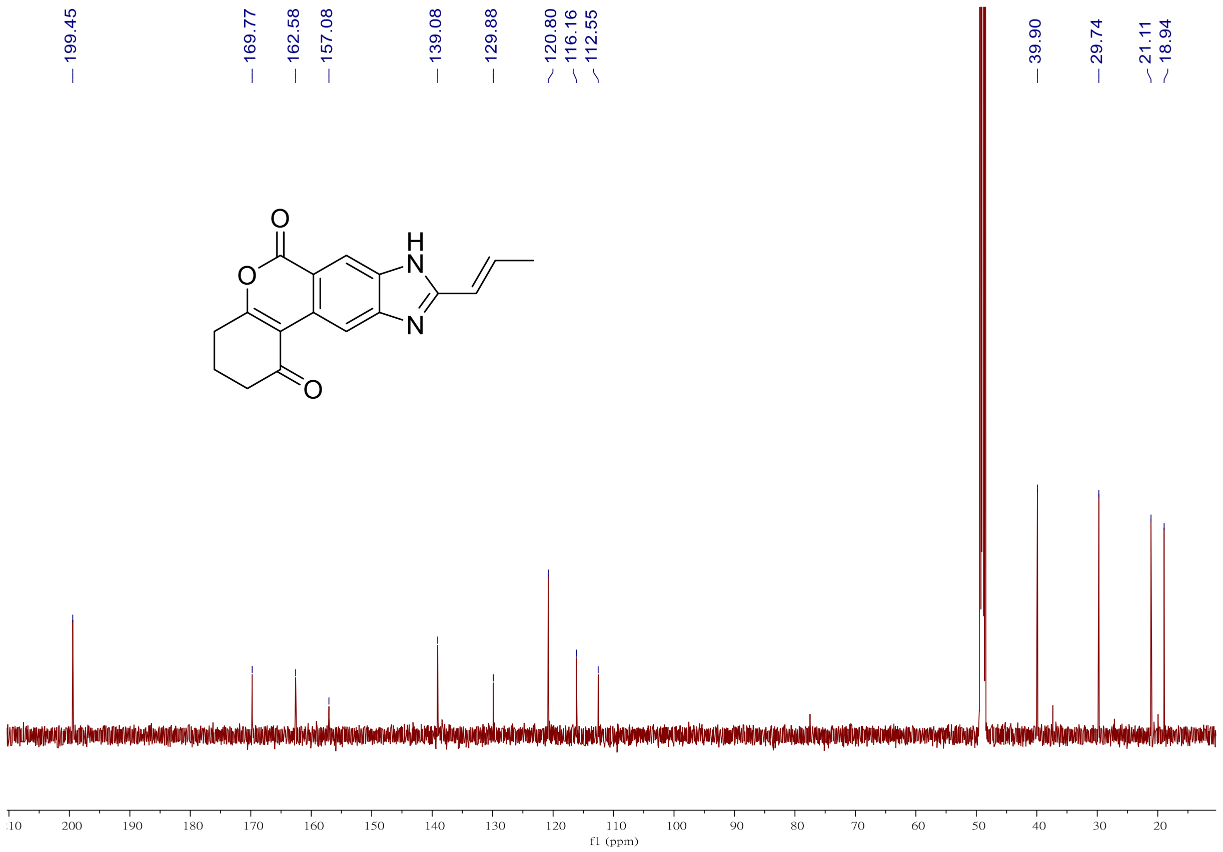


**Figure S218.** $^{13}C\{^{1}H\}$ NMR spectrum of compound **4u** (151 MHz, $CD_3OD$).

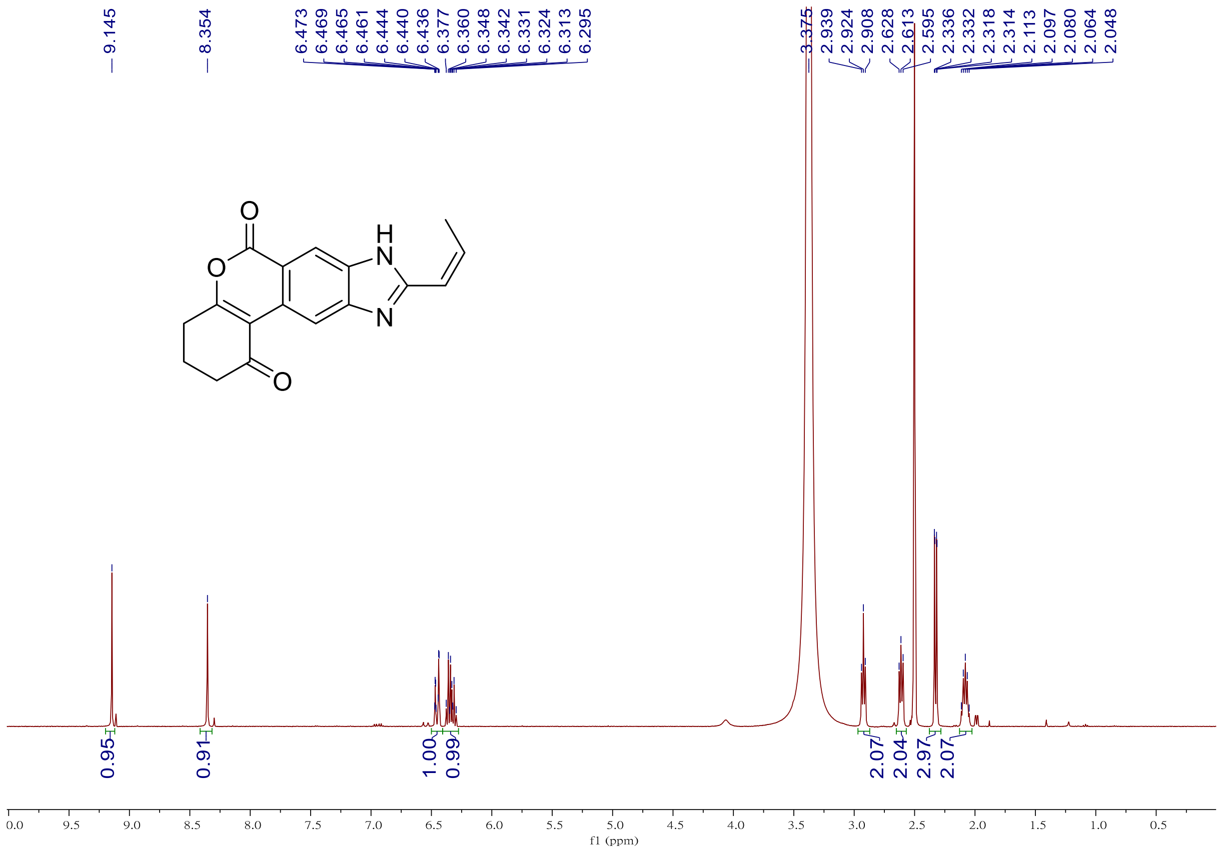


**Figure S219.** $^{1}$H NMR spectrum of compound **4v** (400 MHz, $(CD_3)_2SO$).

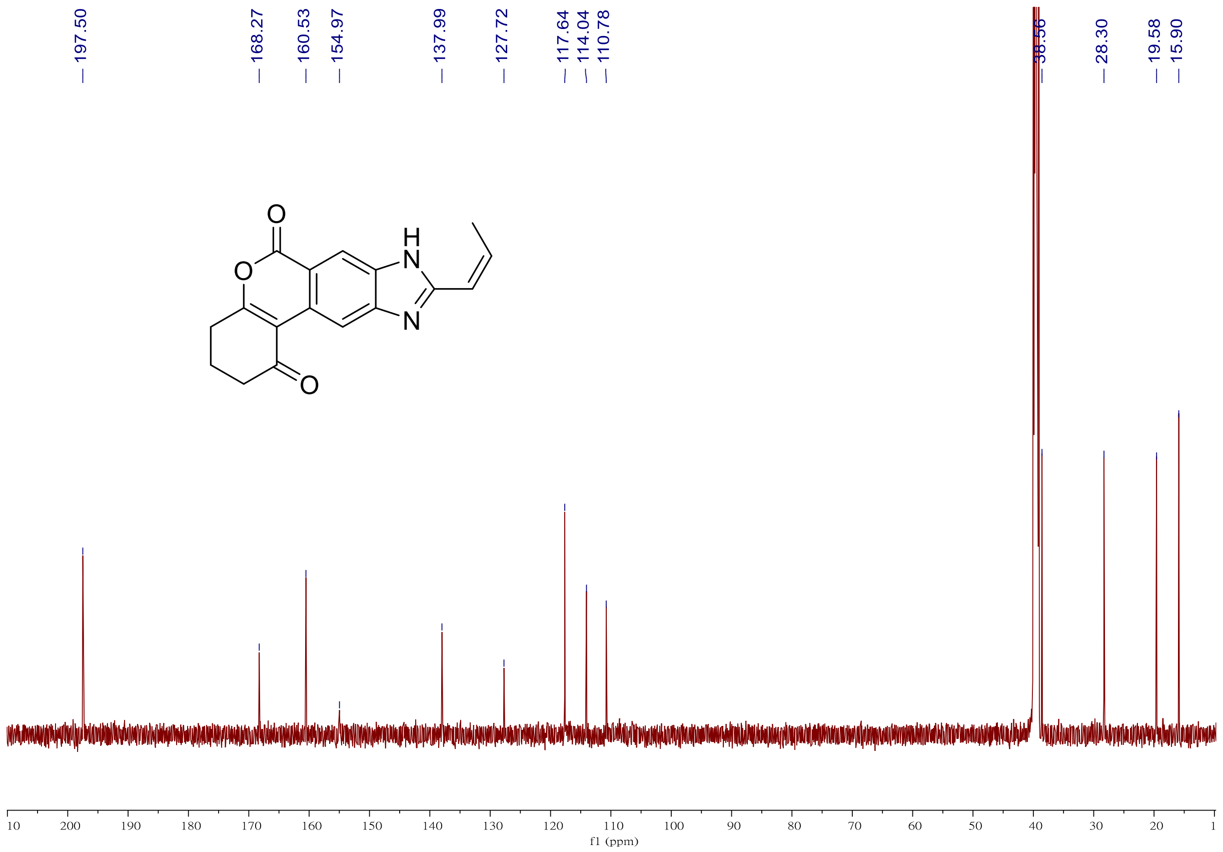


**Figure S220.** $^{13}C\{^{1}H\}$ NMR spectrum of compound **4v** (151 MHz, $(CD_3)_2SO$).

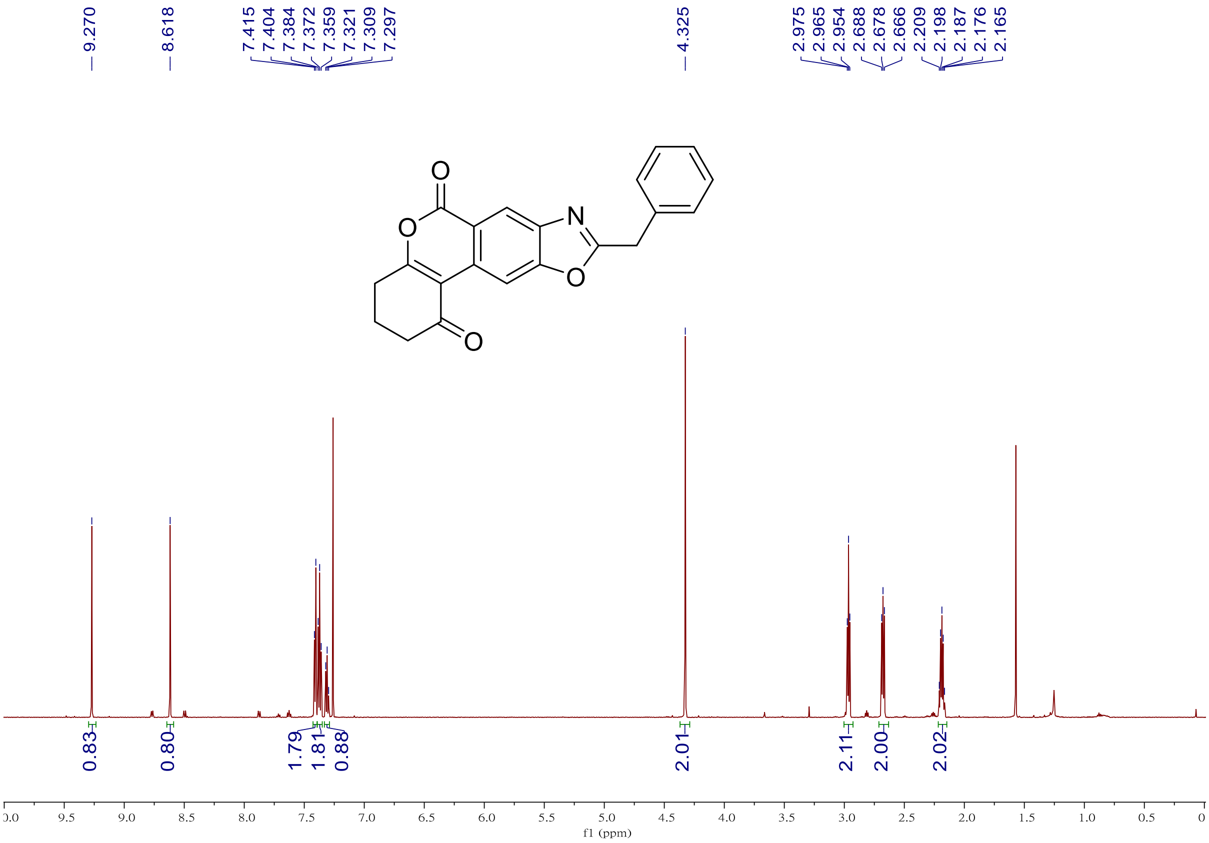


**Figure S221.** $^{1}H$ NMR spectrum of compound **4x** (600 MHz, $CDCl_3$).

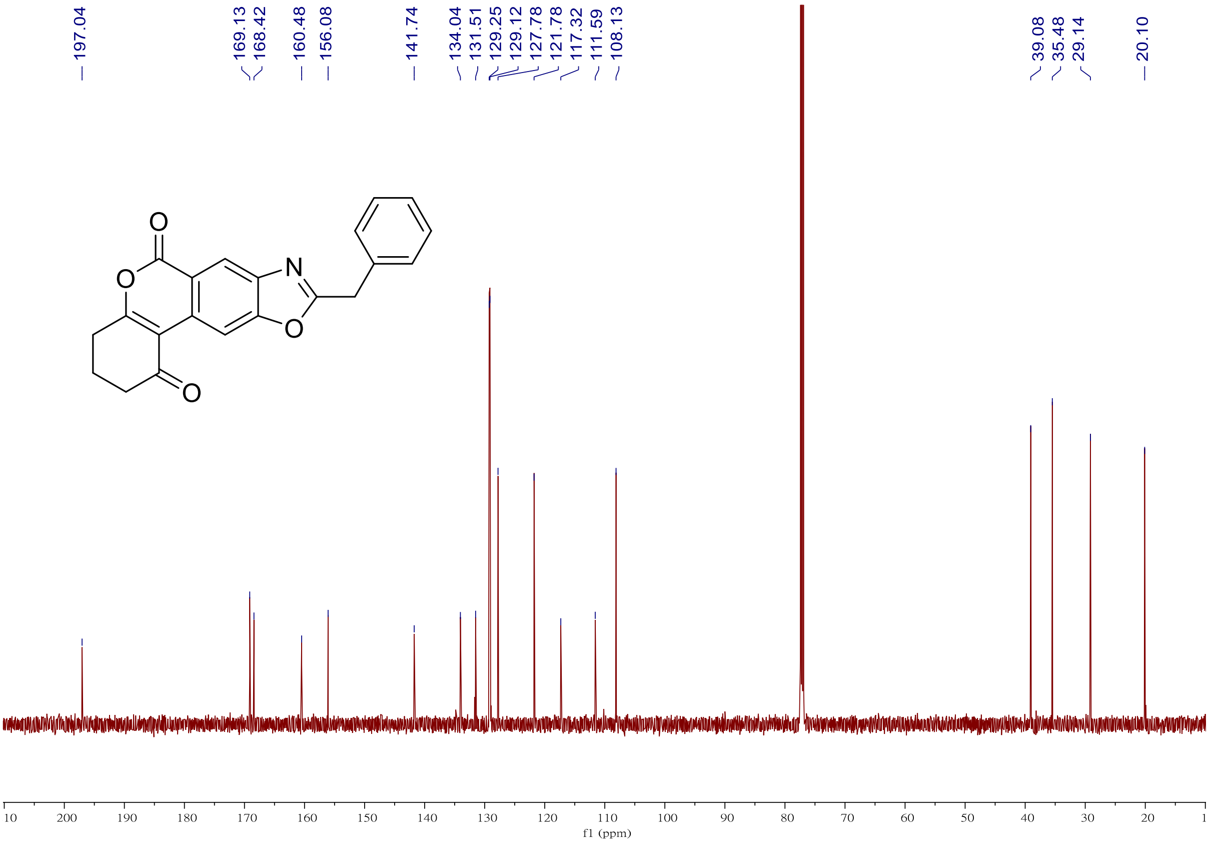


**Figure S222.** $^{13}C\{^{1}H\}$ NMR spectrum of compound **4x** (151 MHz, $CDCl_3$).

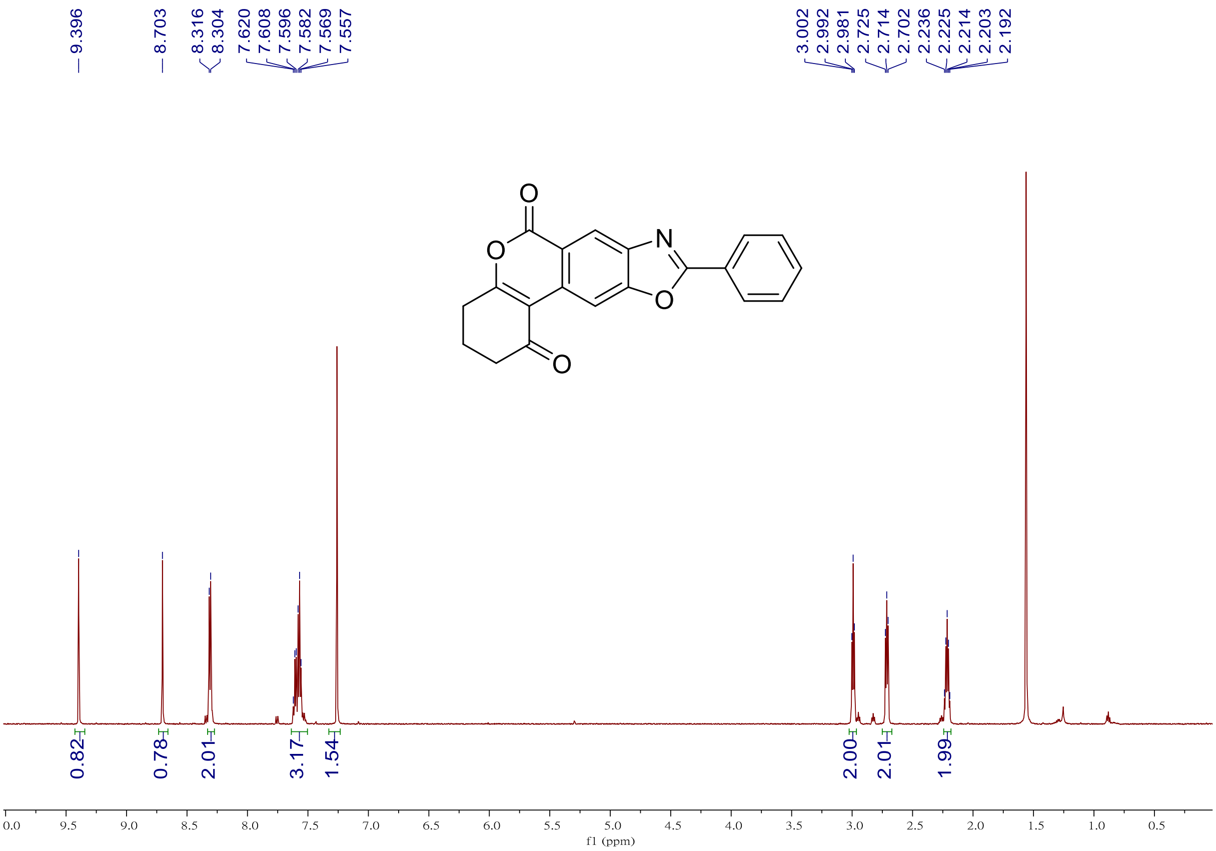


**Figure S223.** $^{1}$H NMR spectrum of compound **4y** (600 MHz, $CDCl_3$).

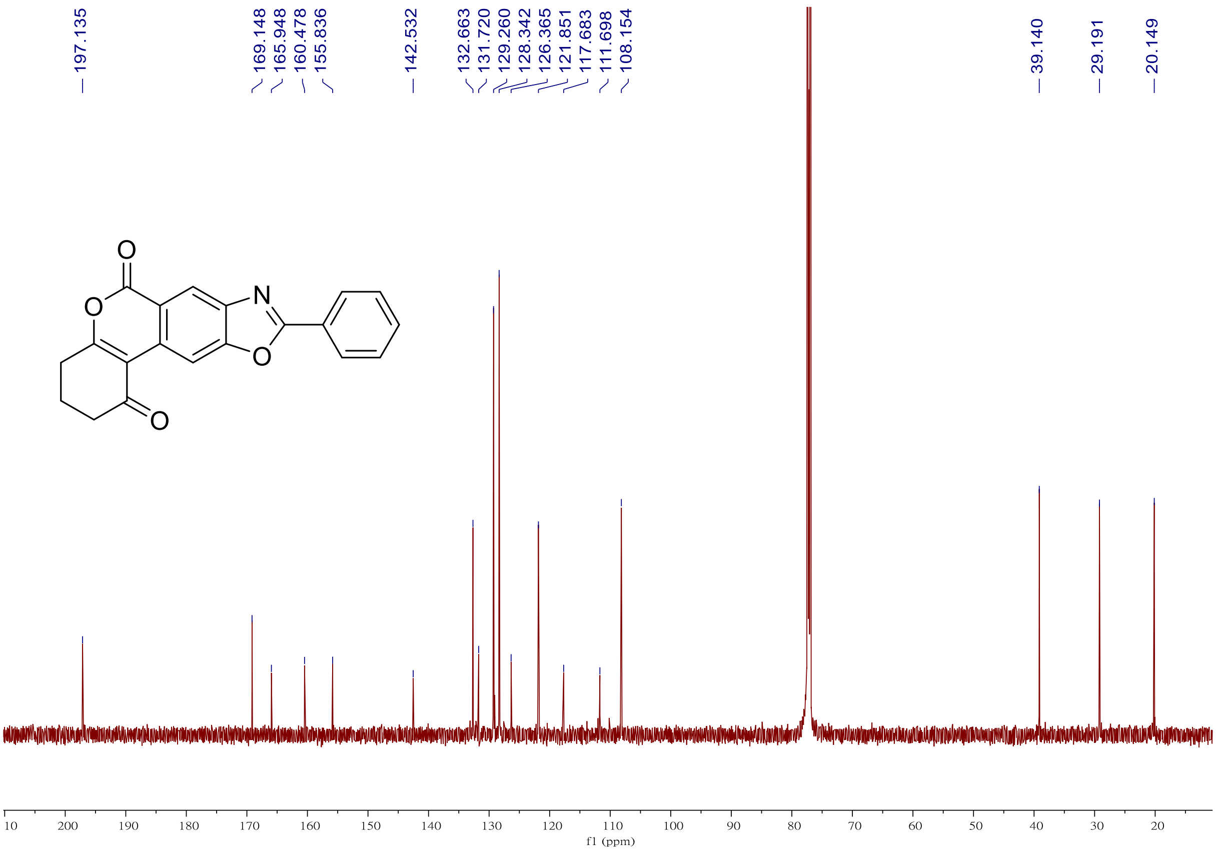


**Figure S224.** $^{13}$C{$^{1}$H} NMR spectrum of compound **4y** (151 MHz, $CDCl_3$).

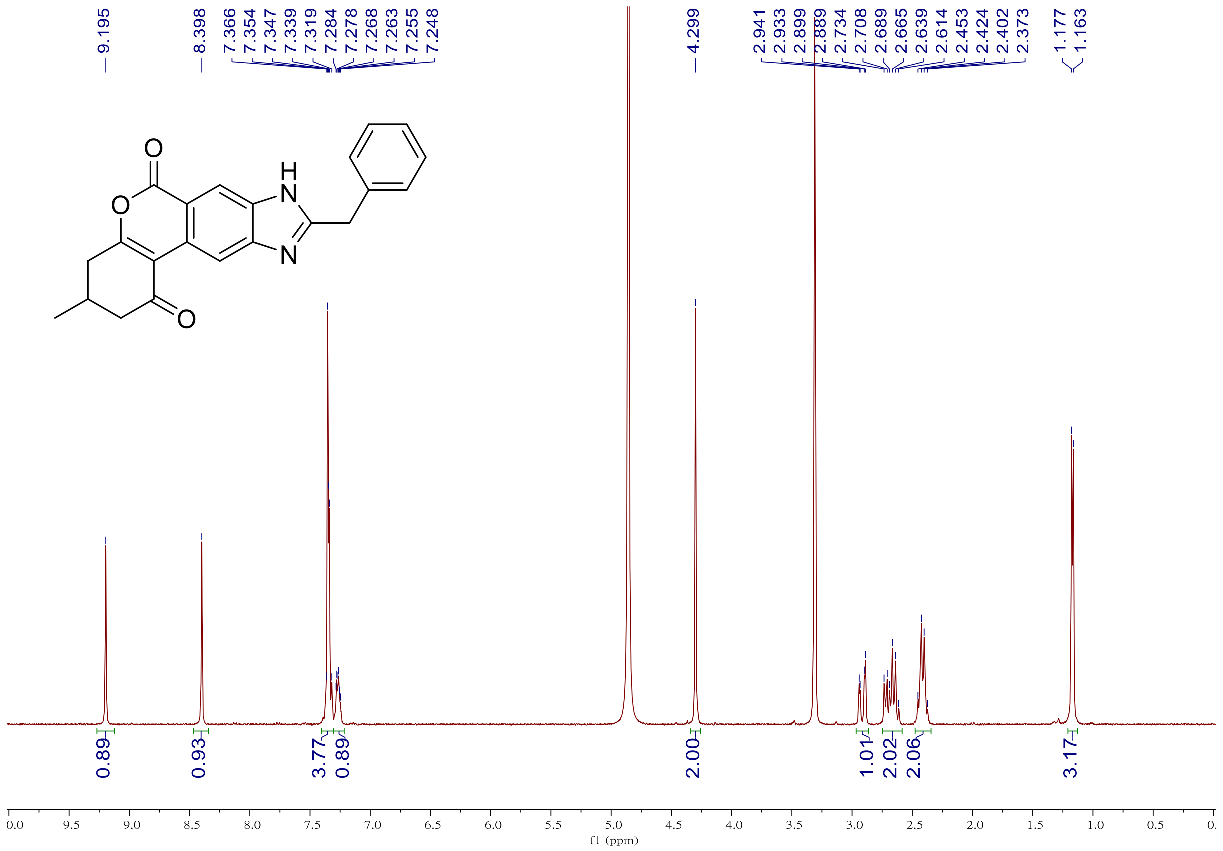


**Figure S225.** $^{1}$H NMR spectrum of compound **4ab** (400 MHz, $CD_3OD$).

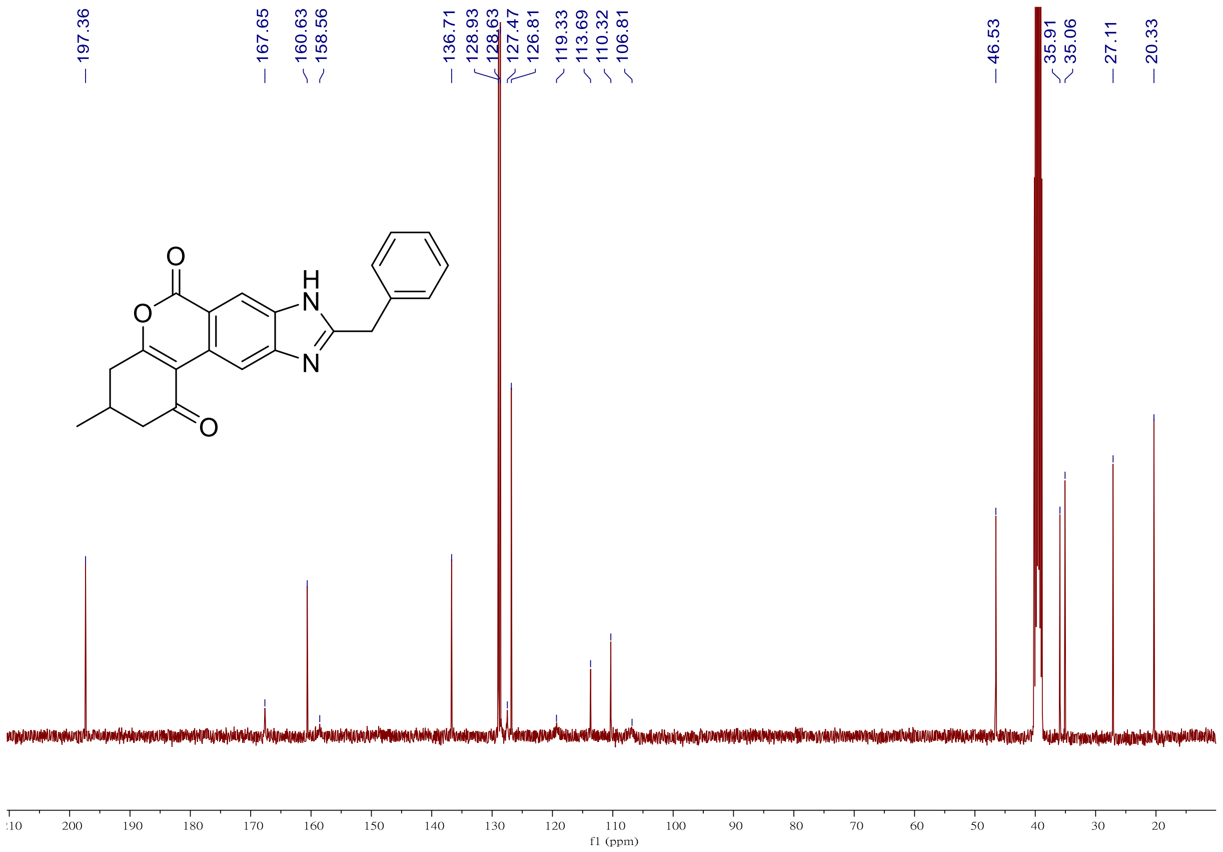


**Figure S226.** $^{13}C\{^{1}H\}$ NMR spectrum of compound **4ab** (101 MHz, $(CD_3)_2SO$).

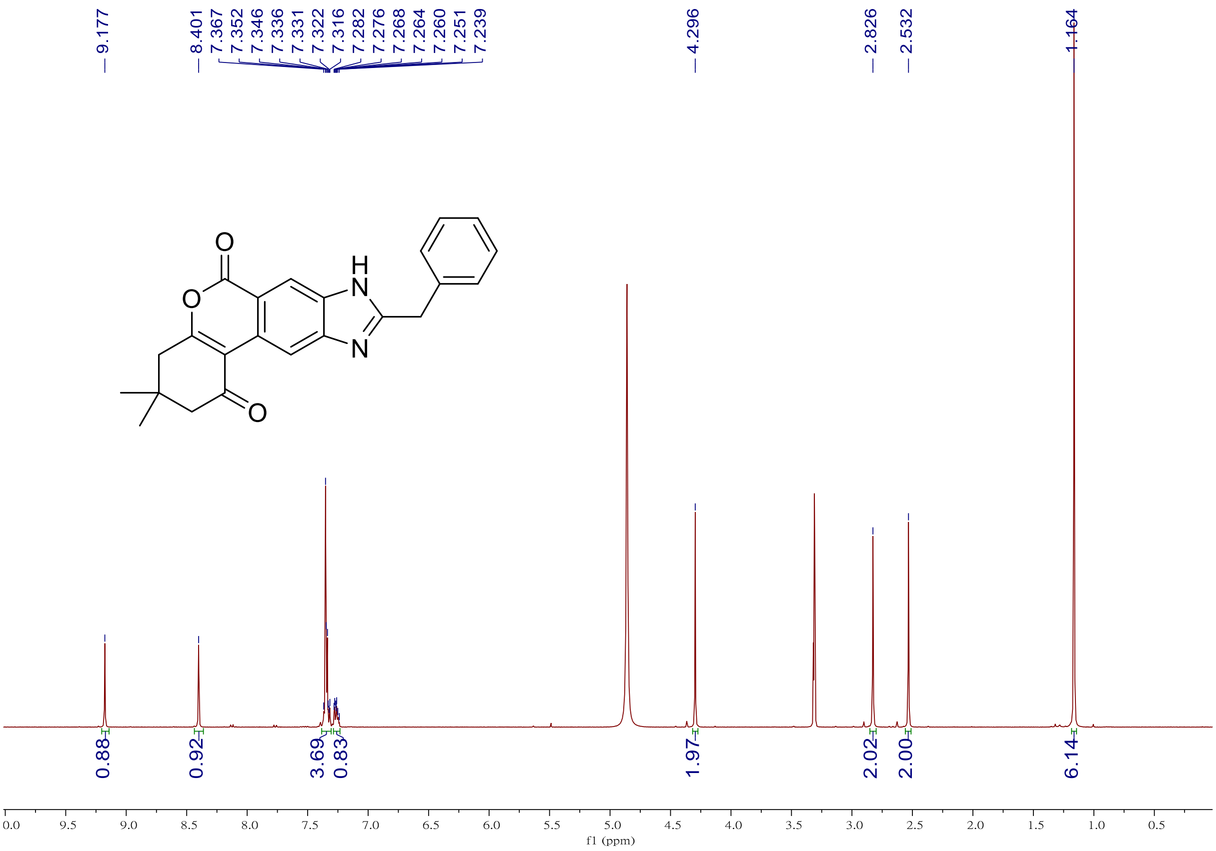

**Figure S227.** $^{1}H$ NMR spectrum of compound **4ac** (400 MHz, $CD_3OD$).

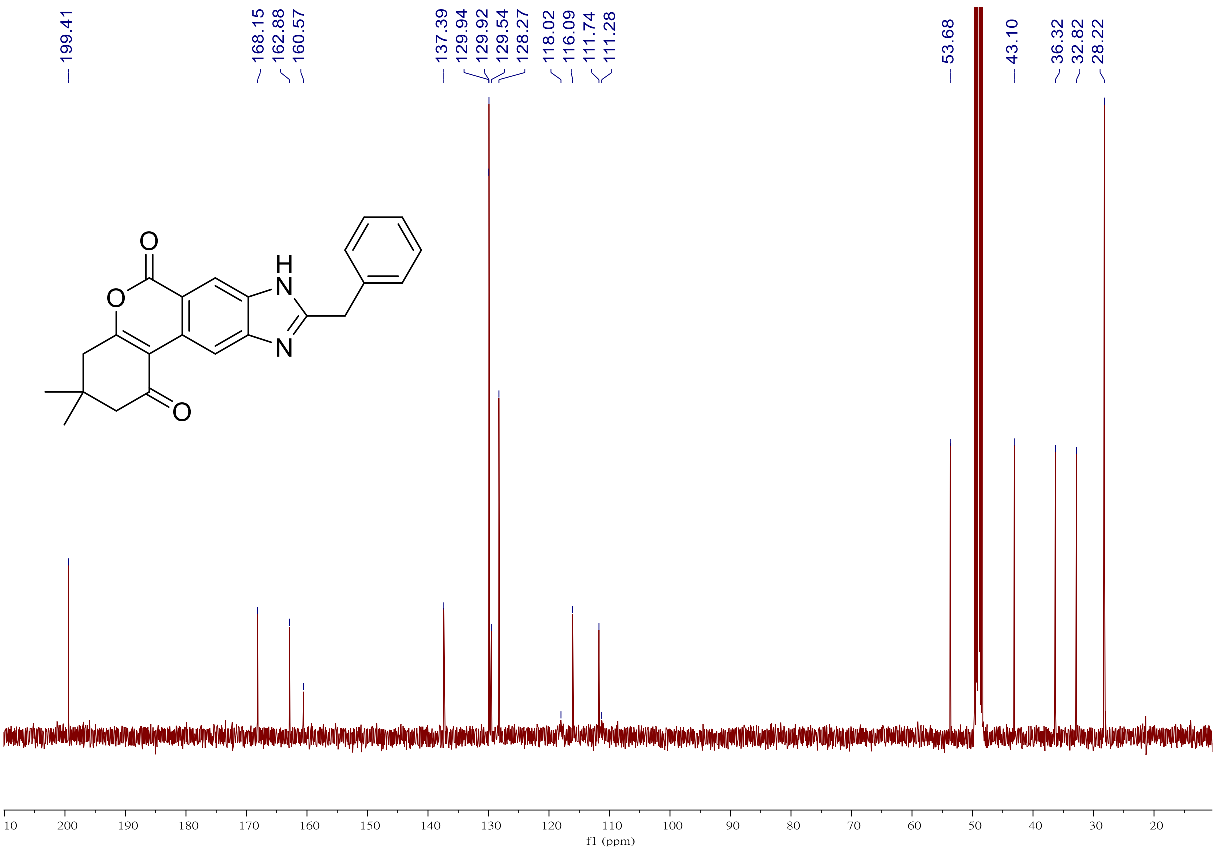

**Figure S228.** $^{13}C\{^{1}H\}$ NMR spectrum of compound **4ac** (101 MHz, $CD_3OD$).

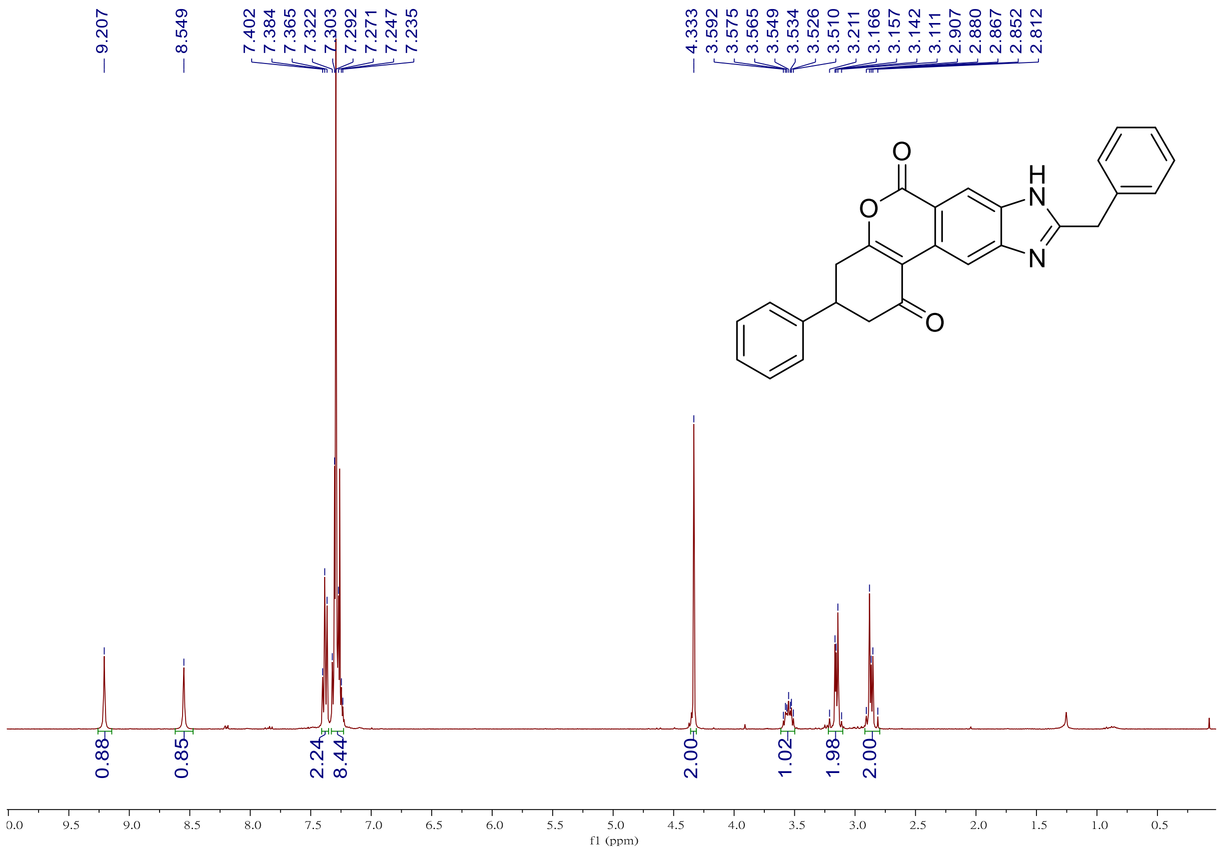


**Figure S229.** $^{1}H$ NMR spectrum of compound **4ad** (400 MHz, $CDCl_3$).

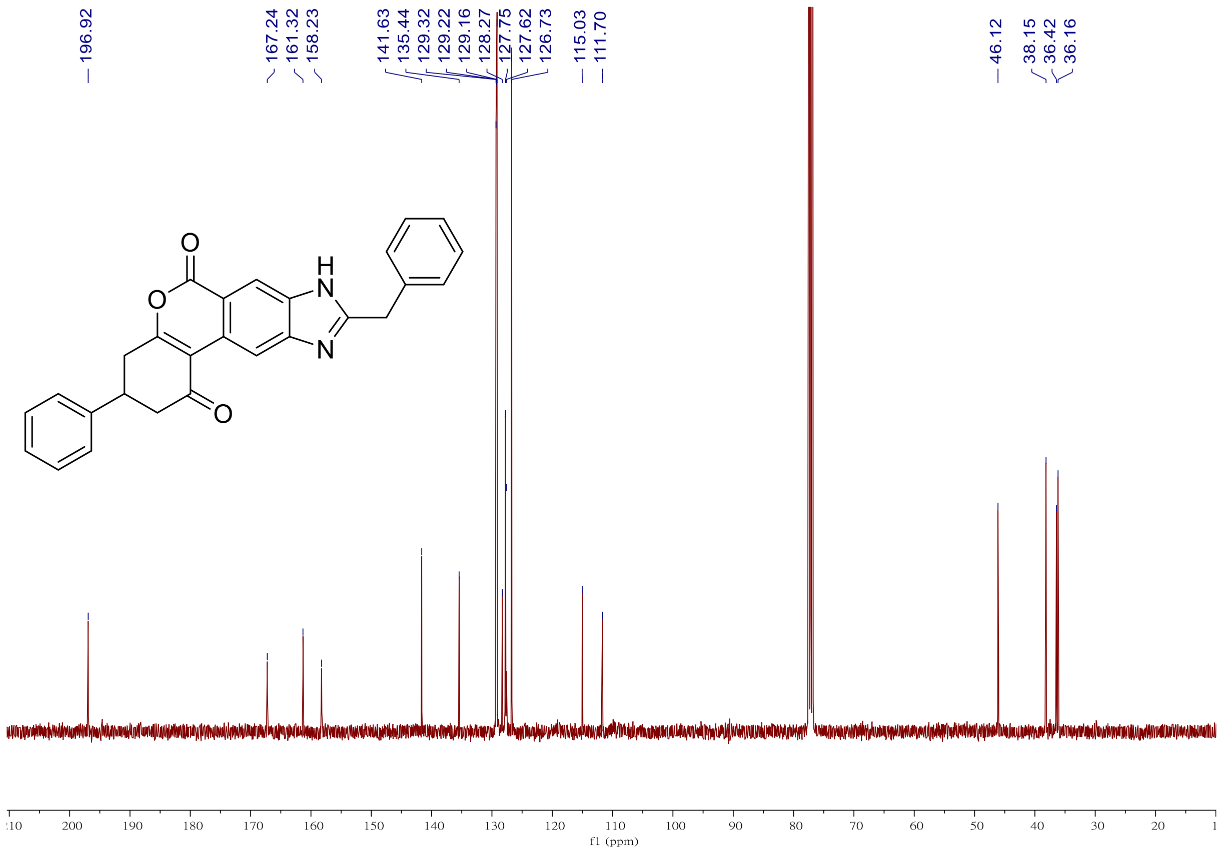


**Figure S230.** $^{13}C\{^{1}H\}$ NMR spectrum of compound **4ad** (101 MHz, $CDCl_3$).

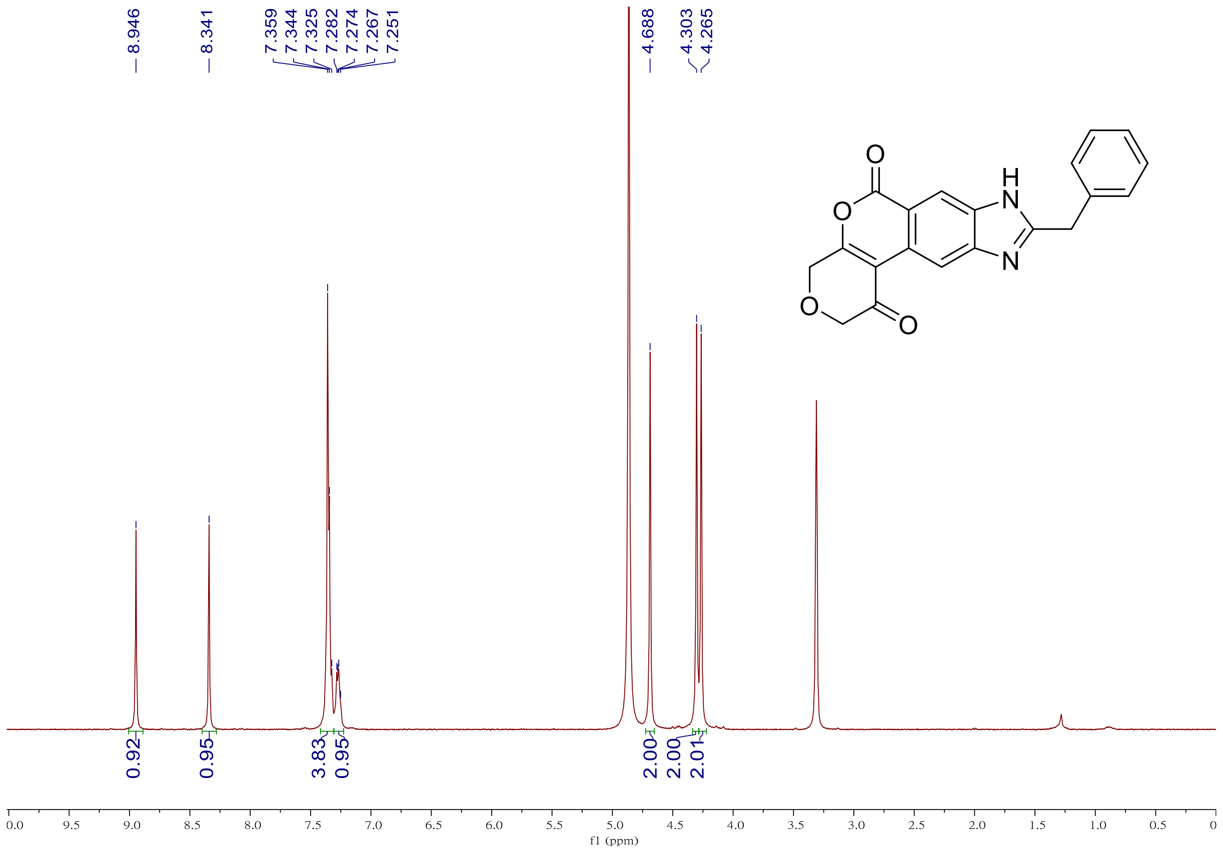


**Figure S231.** $^{1}H$ NMR spectrum of compound **4ae** (400 MHz, $CD_3OD$).

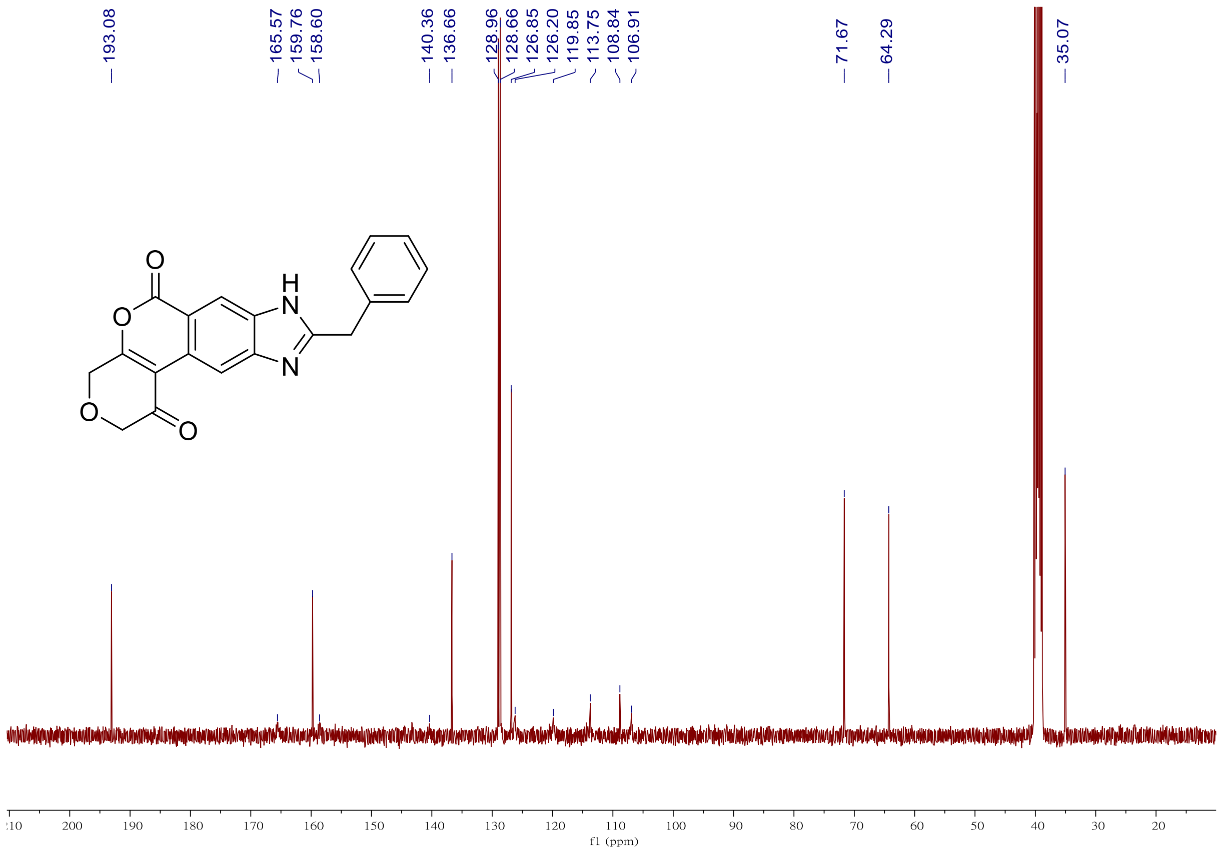


**Figure S232.** $^{13}C\{^{1}H\}$ NMR spectrum of compound **4ae** (101 MHz, $(CD_3)_2SO$).

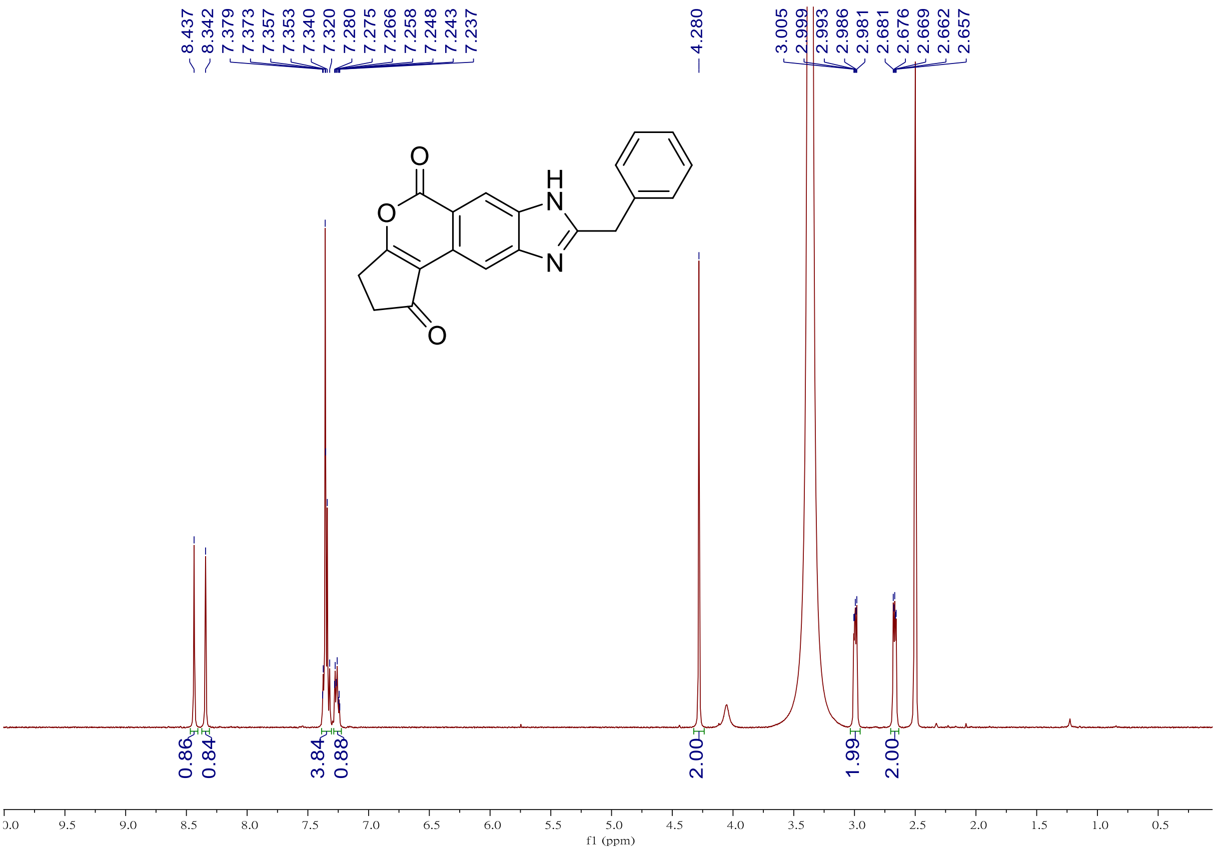


**Figure S233.** $^{1}$H NMR spectrum of compound **4af** (400 MHz, $(CD_3)_2SO$).

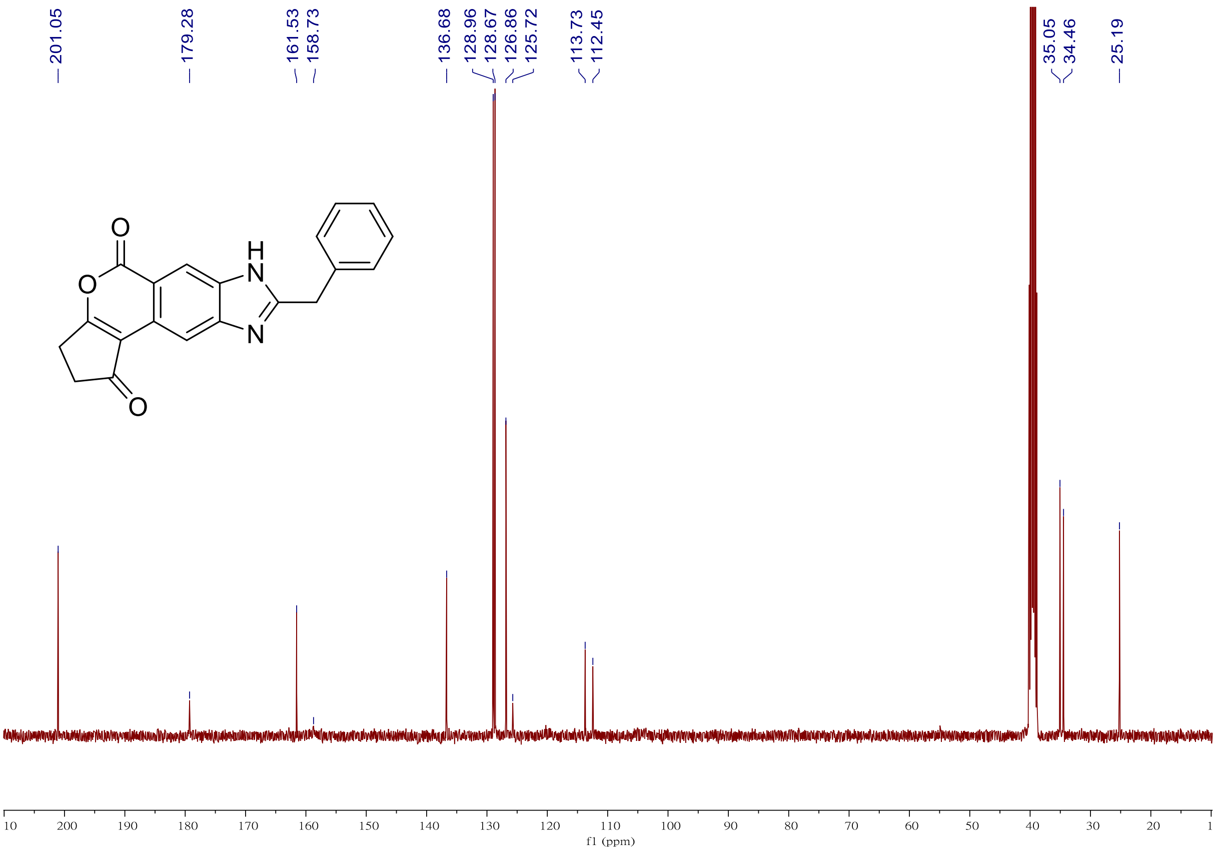


**Figure S234.** $^{13}C\{^{1}H\}$ NMR spectrum of compound **4af** (101 MHz, $(CD_3)_2SO$).

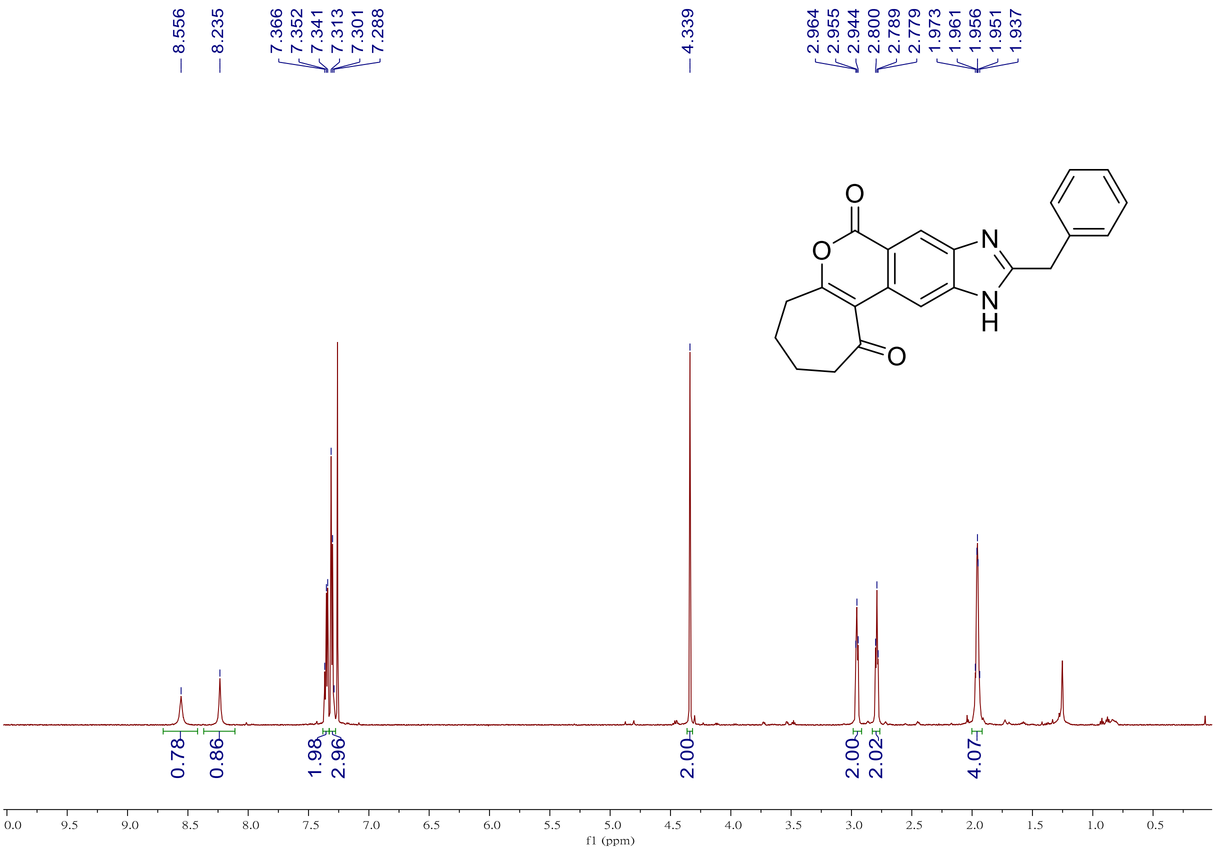


**Figure S235.** $^{1}$H NMR spectrum of compound **4ag** (600 MHz, $CDCl_3$).

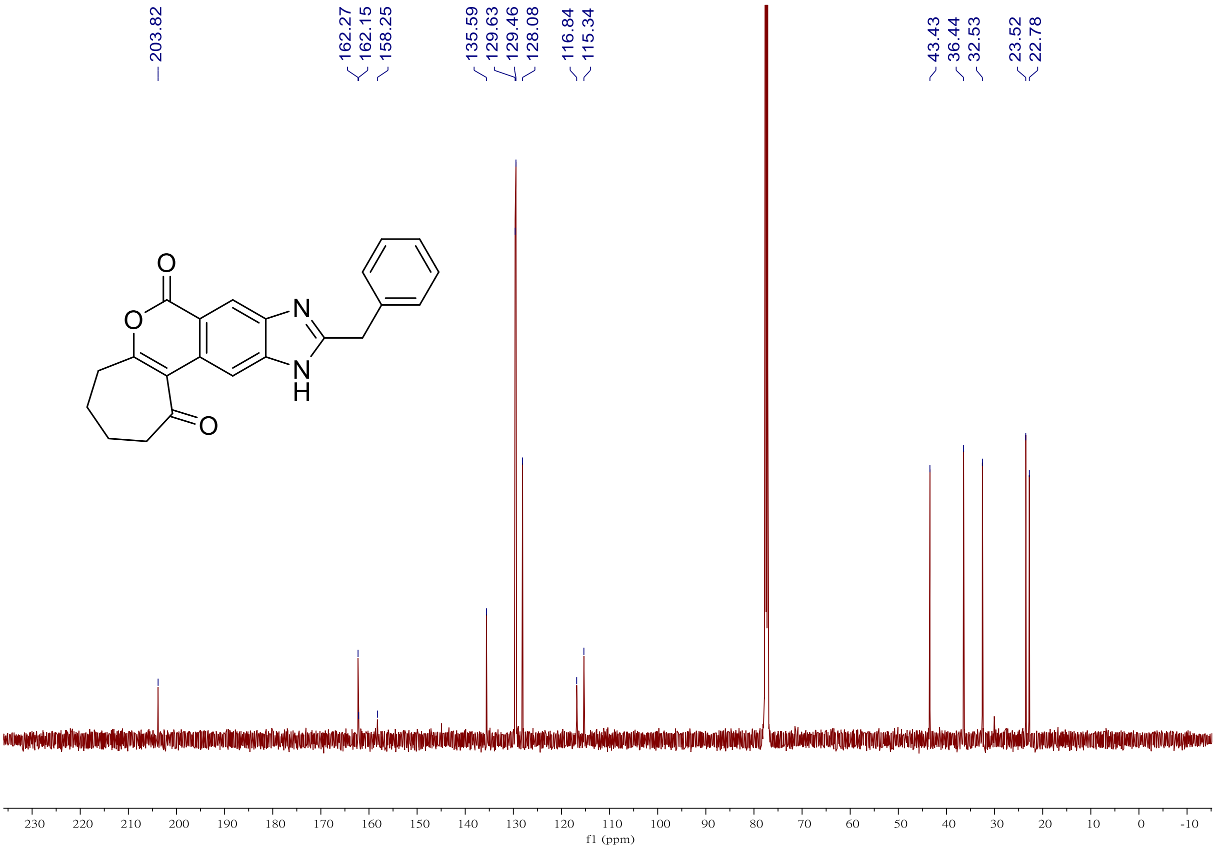


**Figure S236.** $^{13}C\{^{1}H\}$ NMR spectrum of compound **4ag** (151 MHz, $CDCl_3$).

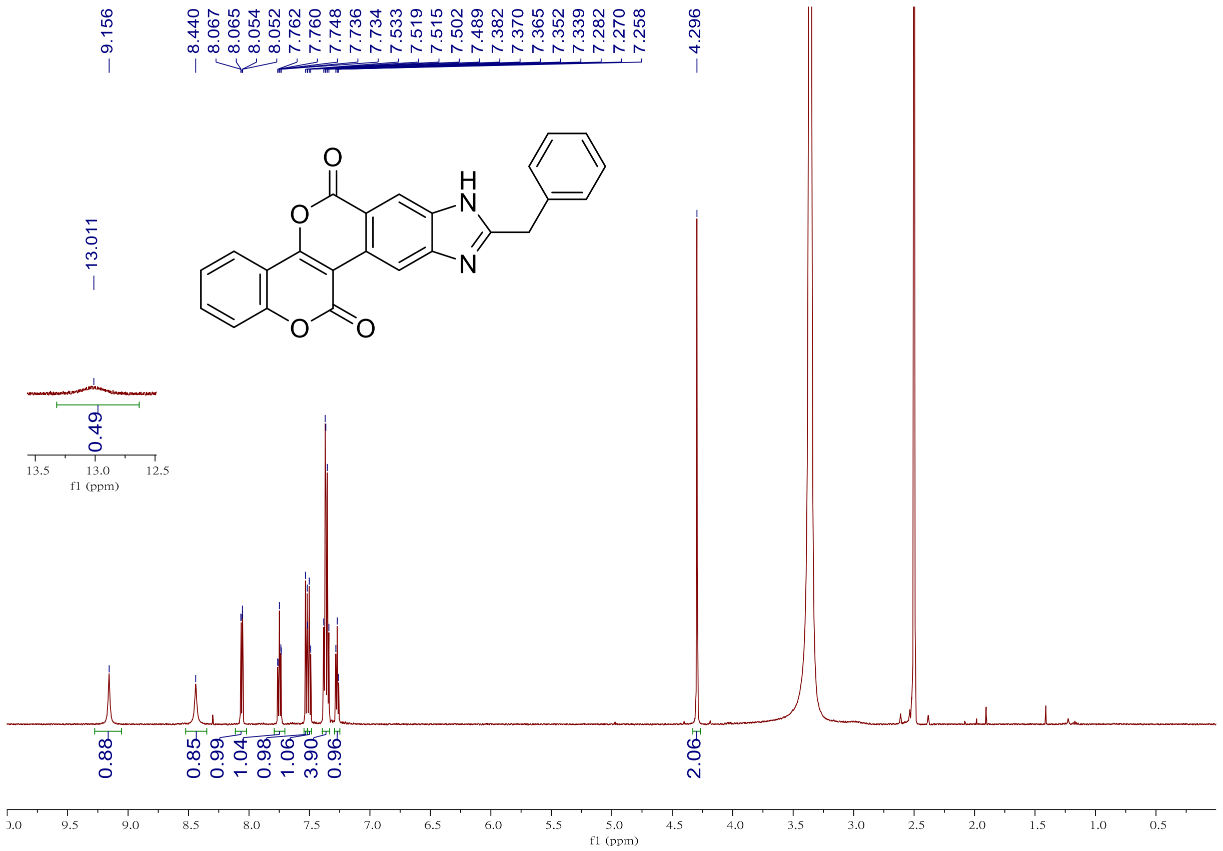


**Figure S237.** $^{1}$H NMR spectrum of compound **4aj** (600 MHz, $(CD_3)_2SO$).

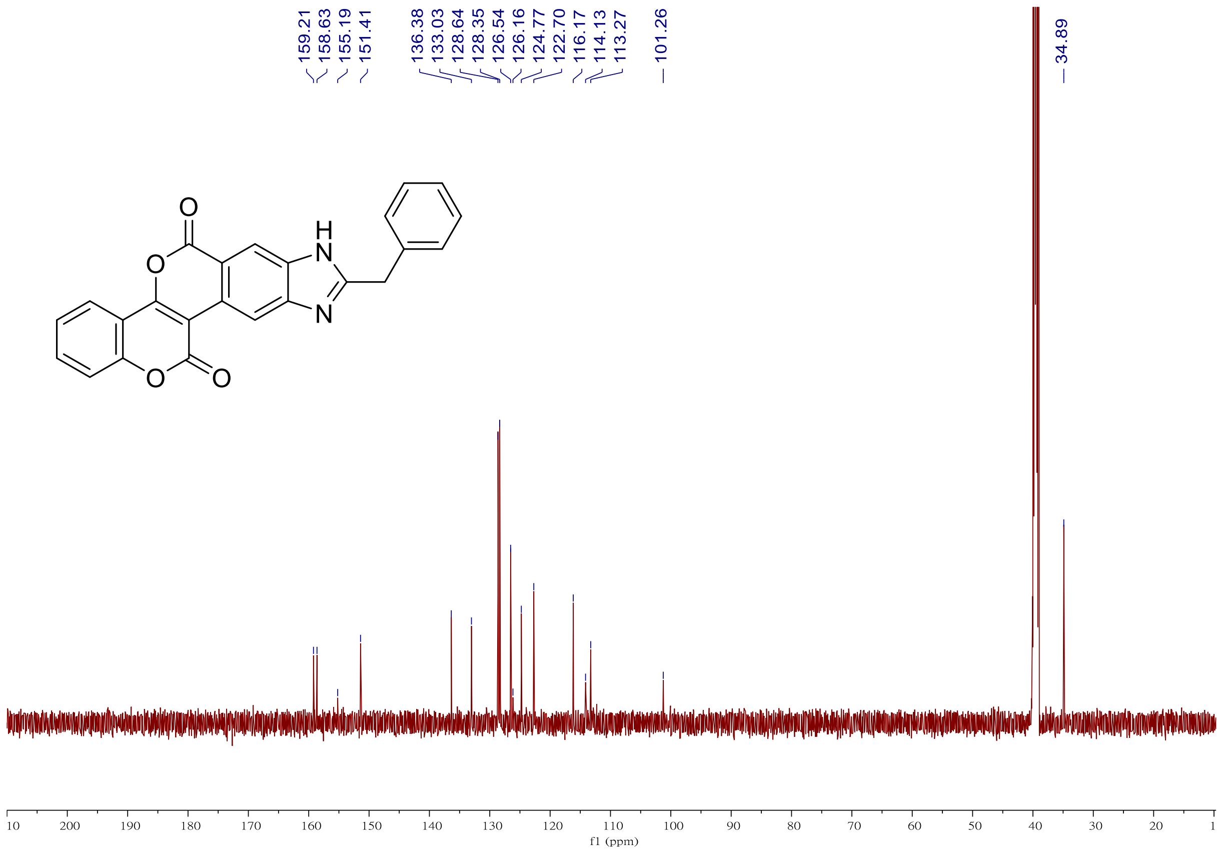


**Figure S238.** $^{13}$C{$^{1}$H} NMR spectrum of compound **4aj** (151 MHz, $(CD_3)_2SO$).

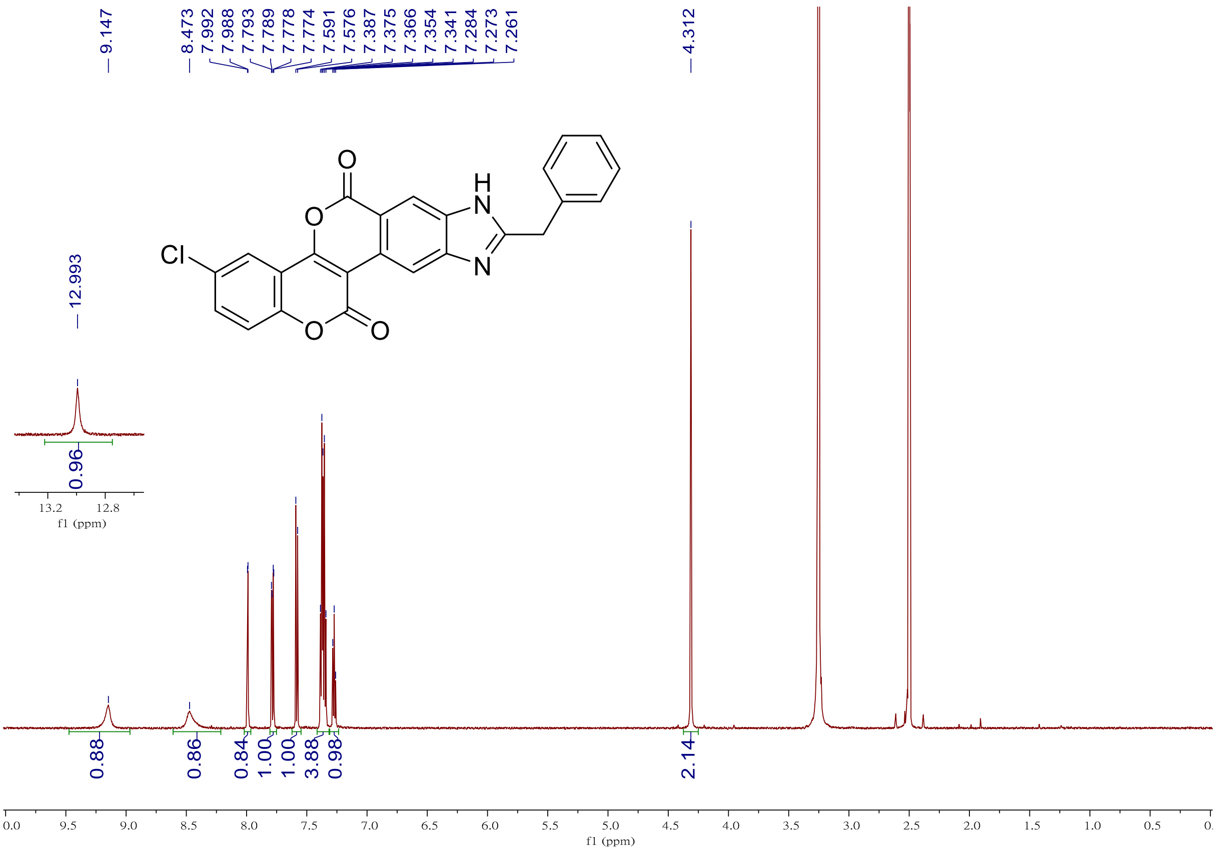


**Figure S239.** $^{1}$H NMR spectrum of compound **4ak** (600 MHz, $(CD_3)_2SO$).

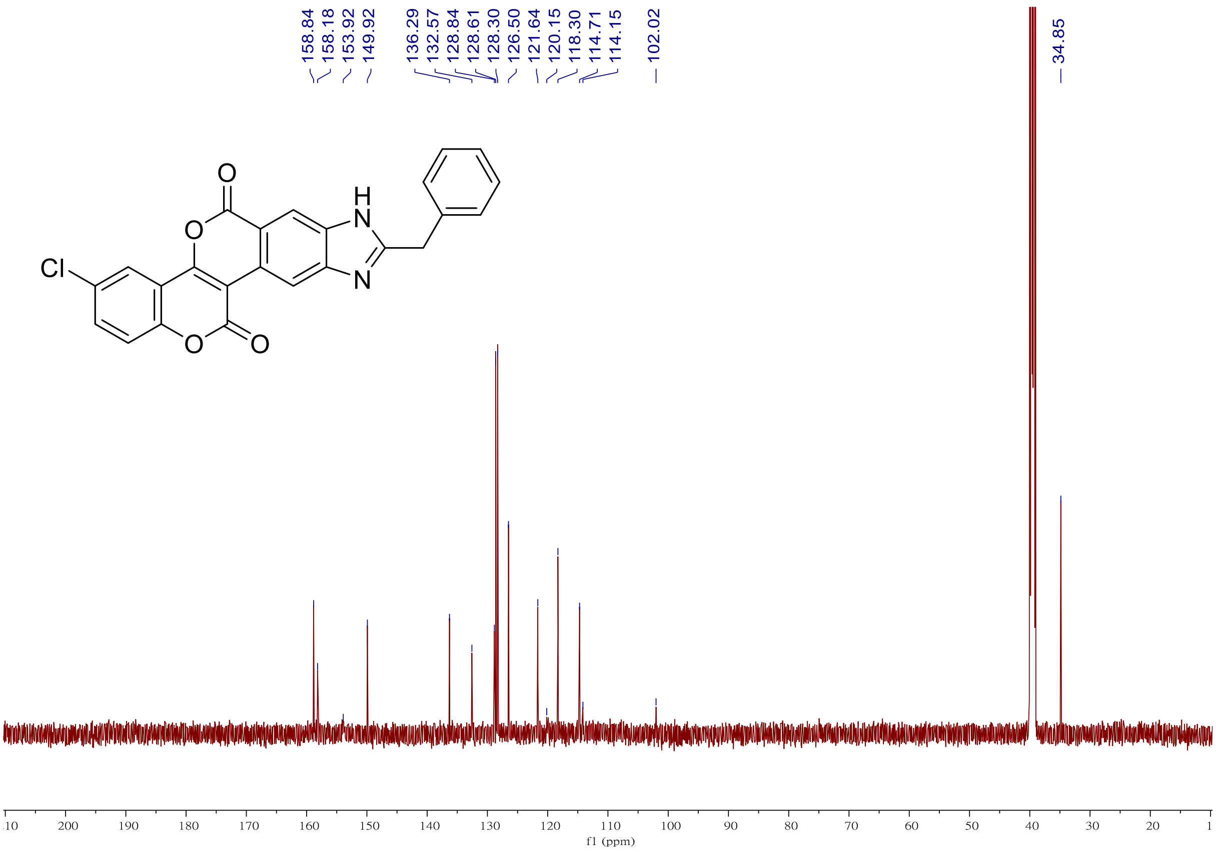


**Figure S240.** $^{13}C\{^{1}H\}$ NMR spectrum of compound **4ak** (151 MHz, $(CD_3)_2SO$).

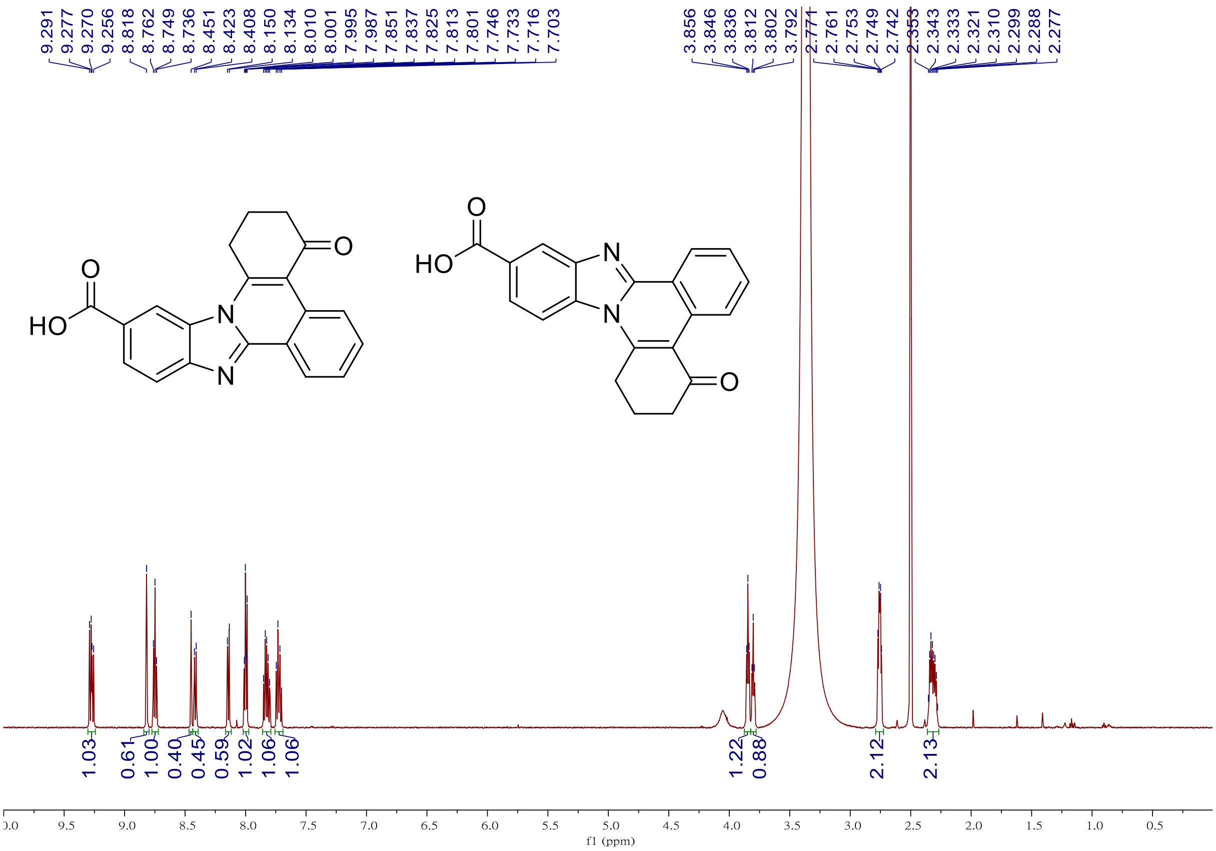

**Figure S241.** $^{1}H$ NMR spectrum of compound **5a/5a'** (600 MHz, $(CD_3)_2SO$).

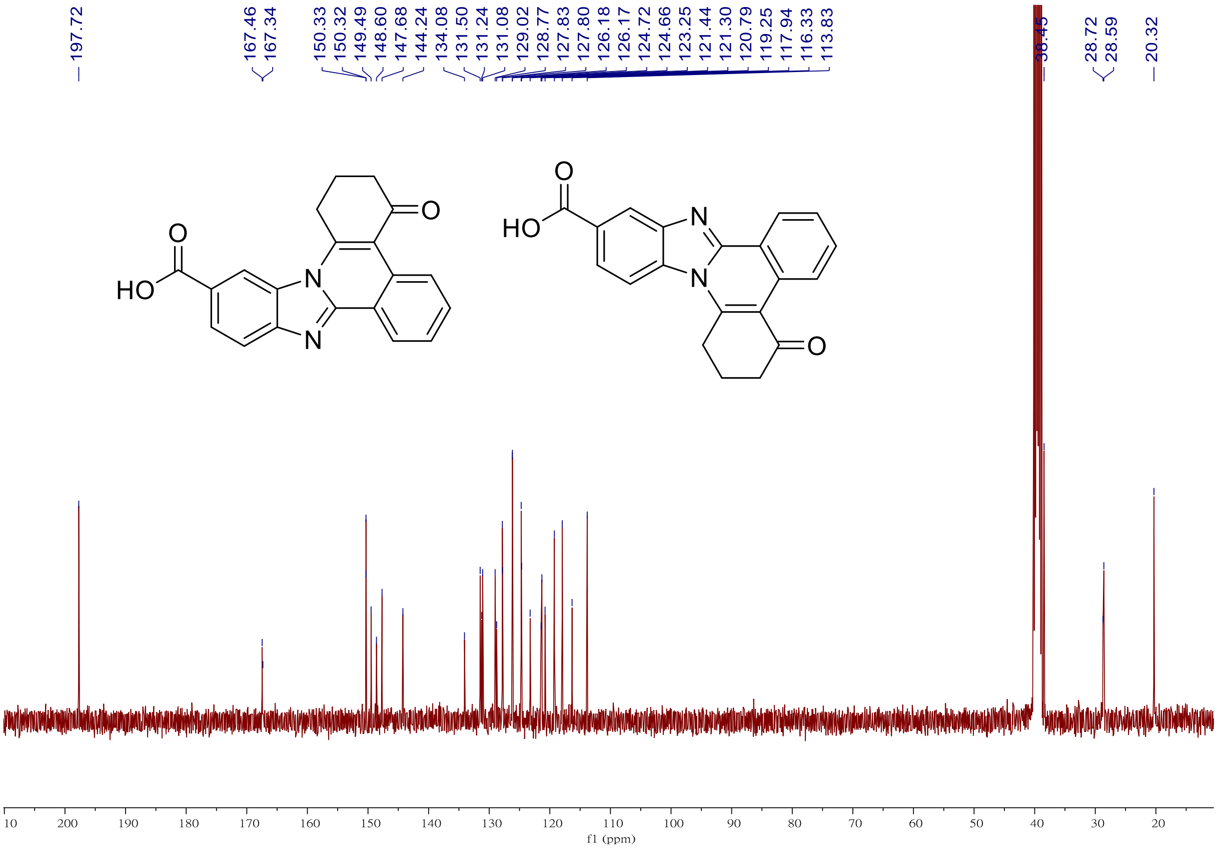

**Figure S242.** $^{13}C\{^{1}H\}$ NMR spectrum of compound **5a/5a'** (101 MHz, $(CD_3)_2SO$).

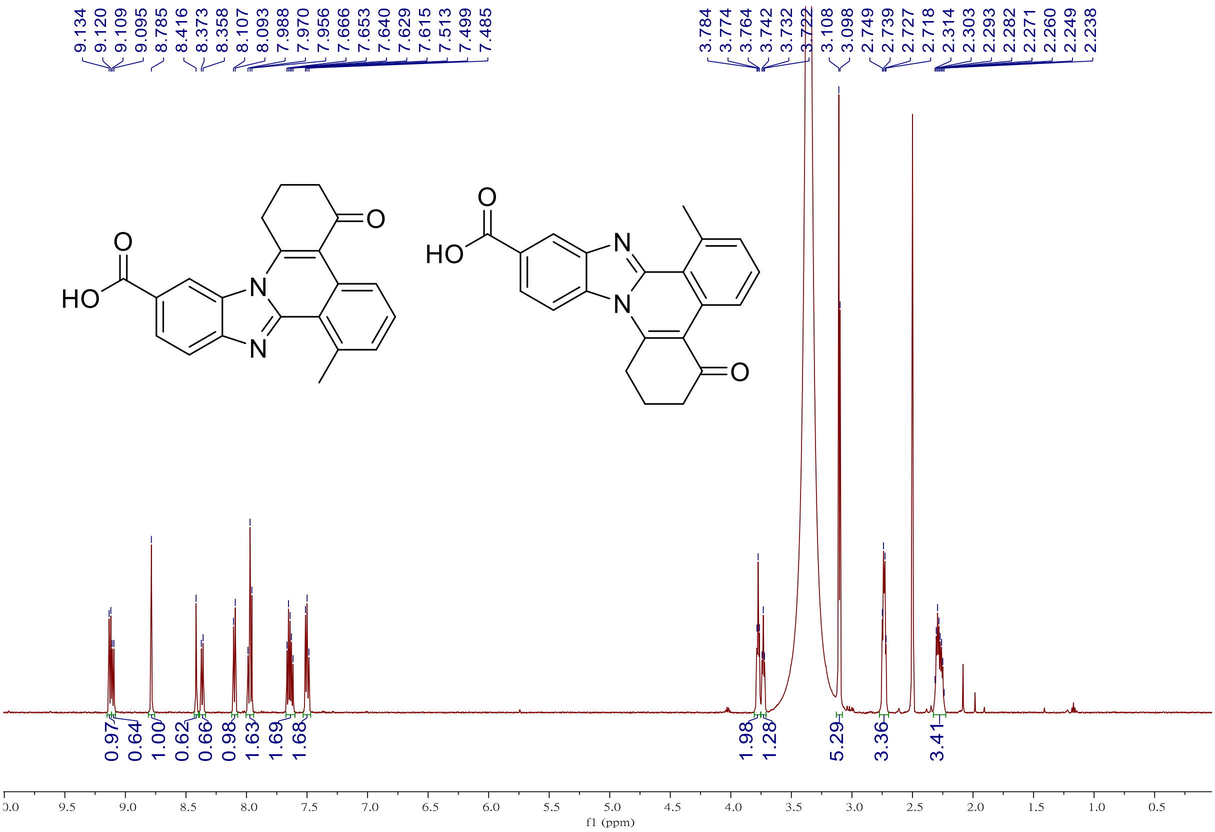


**Figure S243.** $^{1}H$ NMR spectrum of compound **5b/5b'** (600 MHz, $(CD_3)_2SO$).

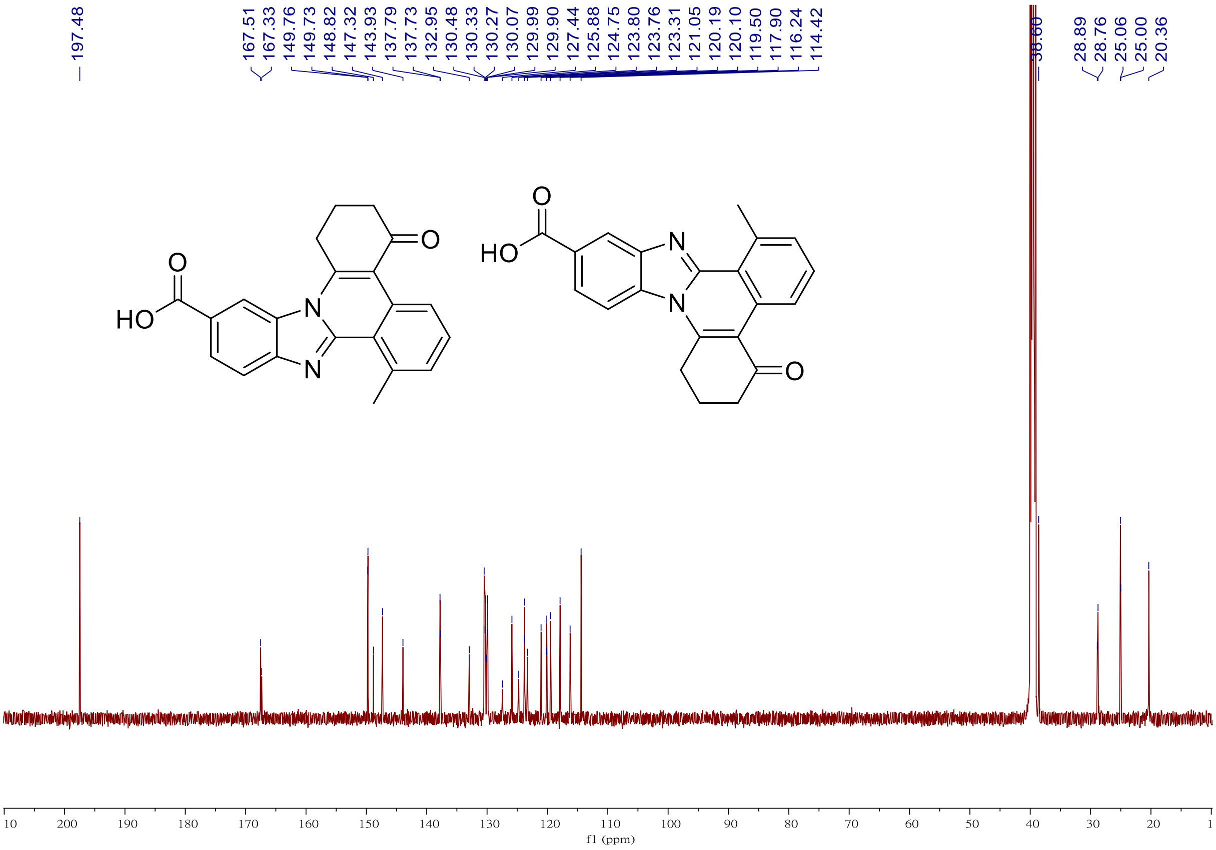


**Figure S244.** $^{13}C\{^{1}H\}$ NMR spectrum of compound **5b/5b'** (151 MHz, $(CD_3)_2SO$).

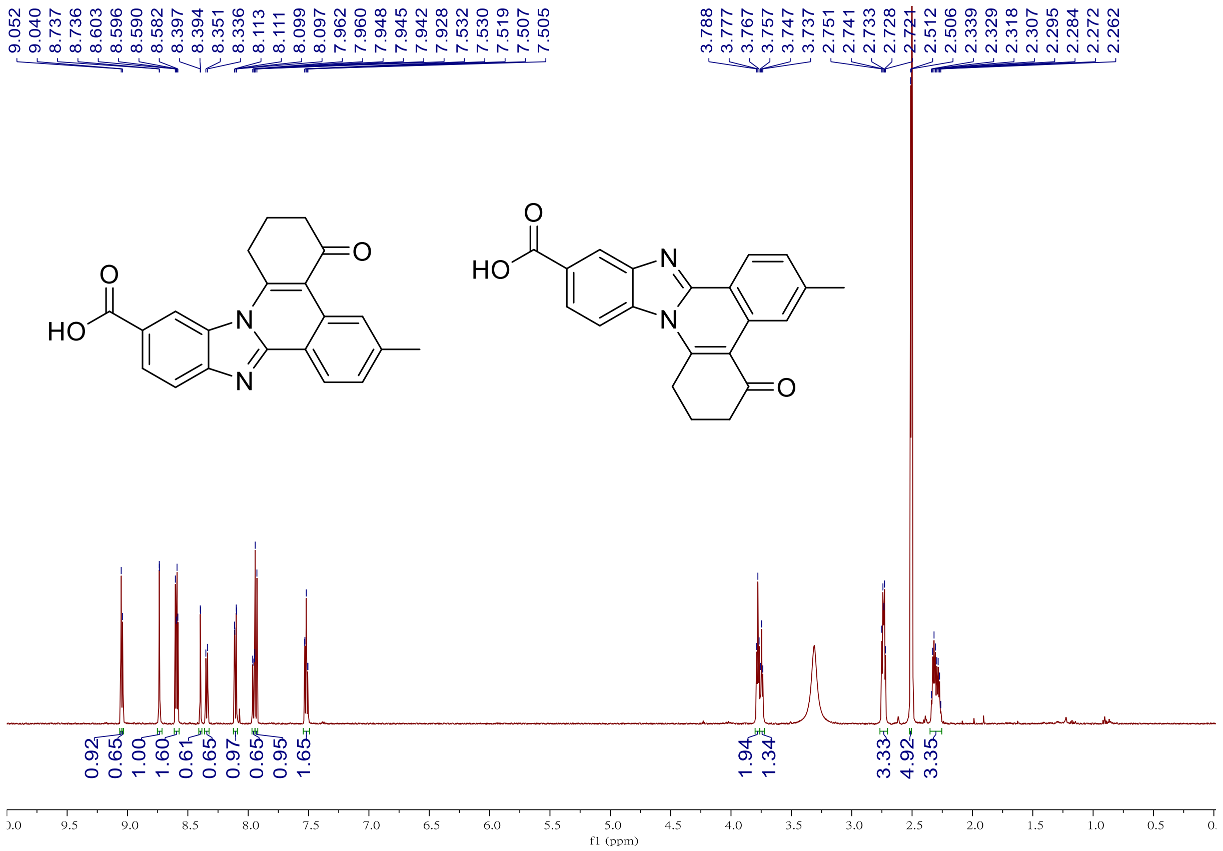

**Figure S245.** $^{1}$H NMR spectrum of compound **5c/5c'** (600 MHz, $(CD_3)_2SO$).

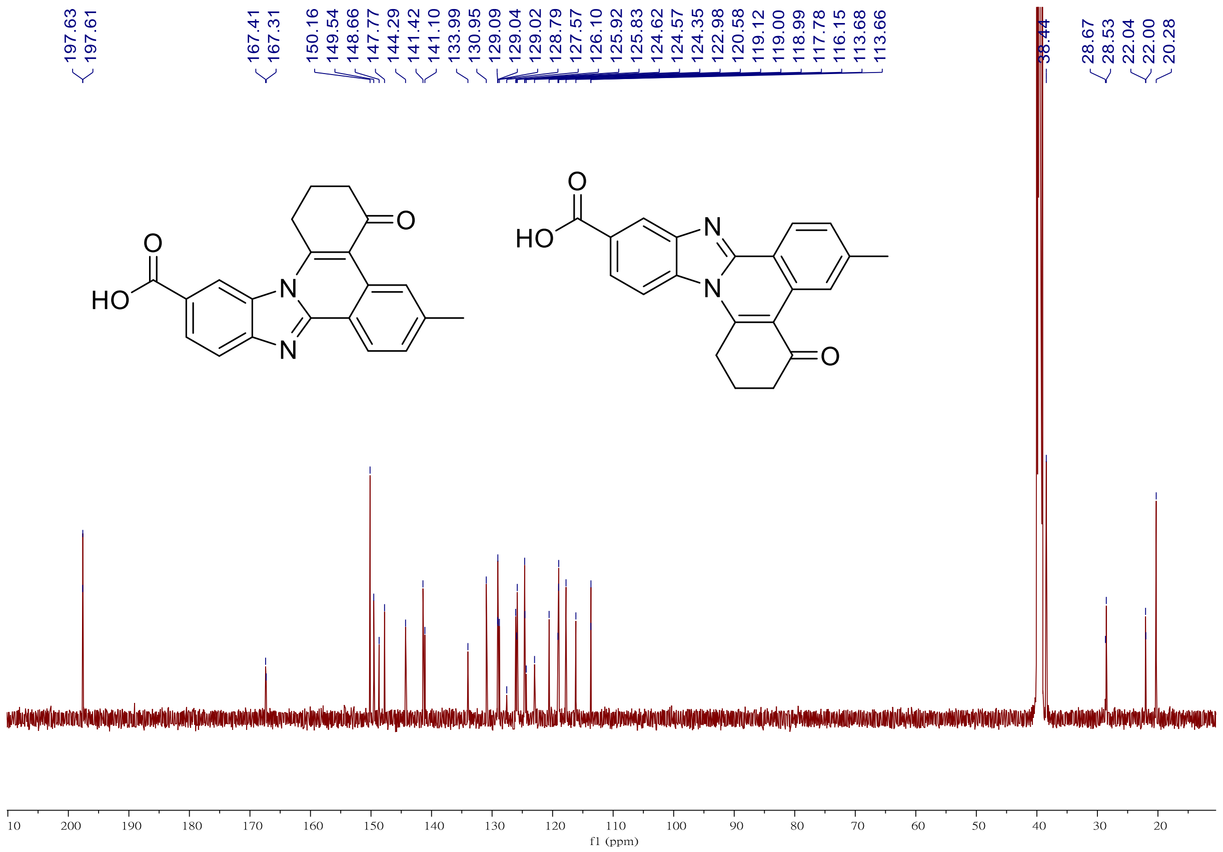

**Figure S246.** $^{13}C\{^{1}H\}$ NMR spectrum of compound **5c/5c'** (151 MHz, $(CD_3)_2SO$).

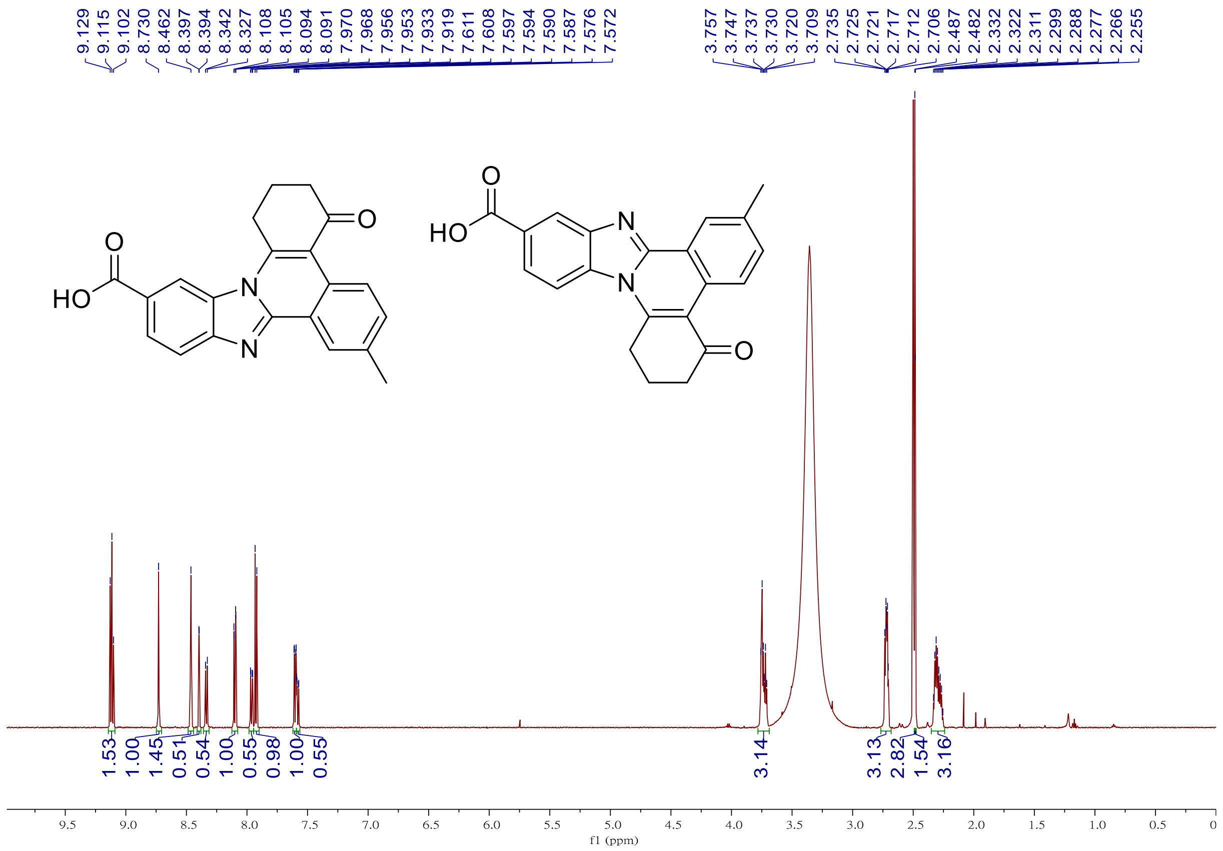


**Figure S247.** $^{1}H$ NMR spectrum of compound **5d/5d’** (600 MHz, $(CD_3)_2SO$).

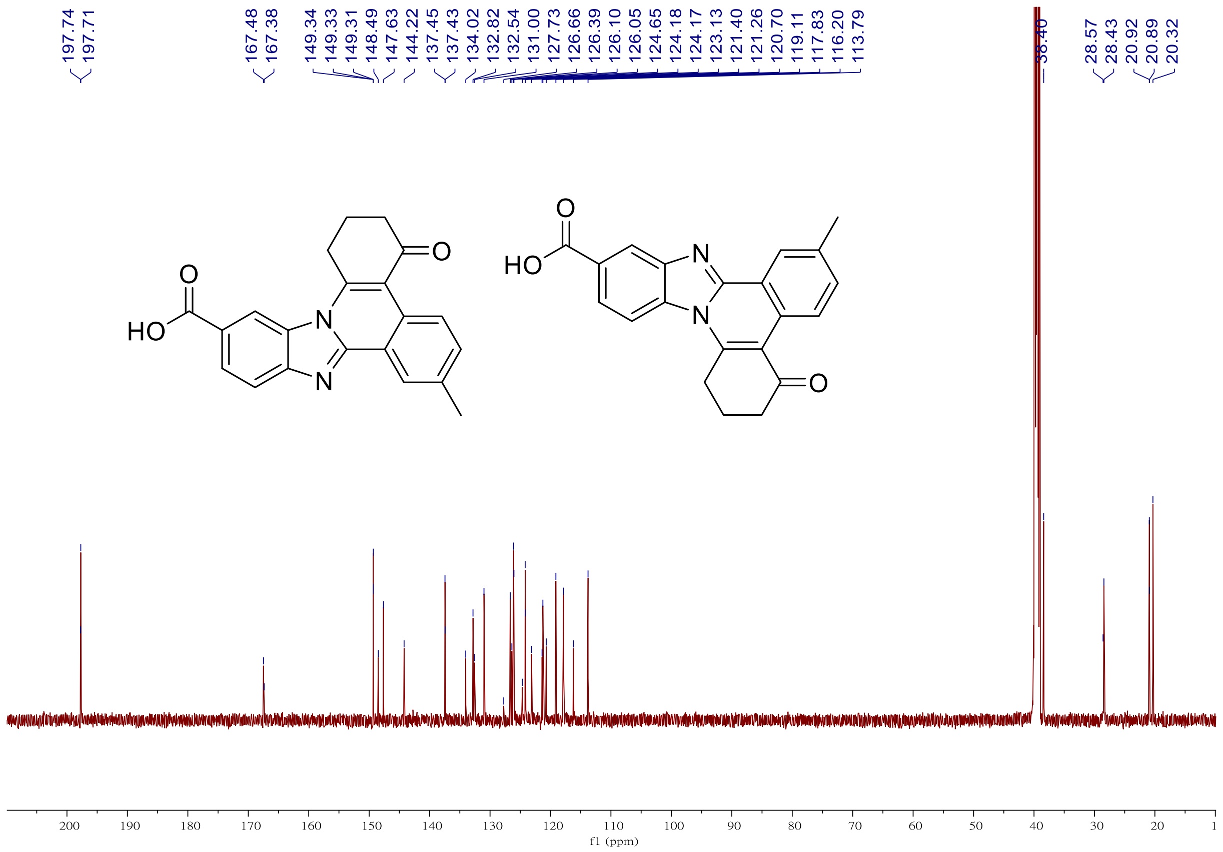


**Figure S248.** $^{13}C\{^{1}H\}$ NMR spectrum of compound **5d/5d’** (151 MHz, $(CD_3)_2SO$).

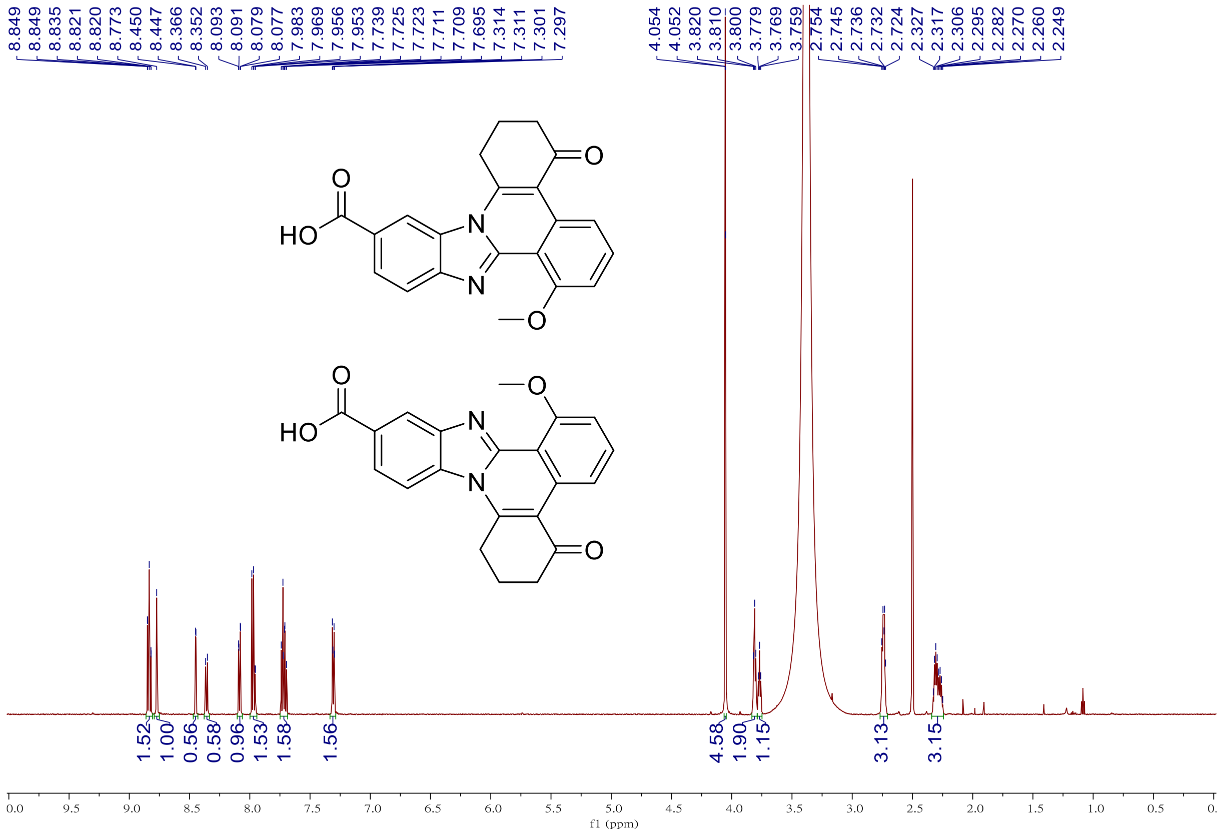

**Figure S249.** $^{1}H$ NMR spectrum of compound **5e/5e'** (600 MHz, $(CD_3)_2SO$).

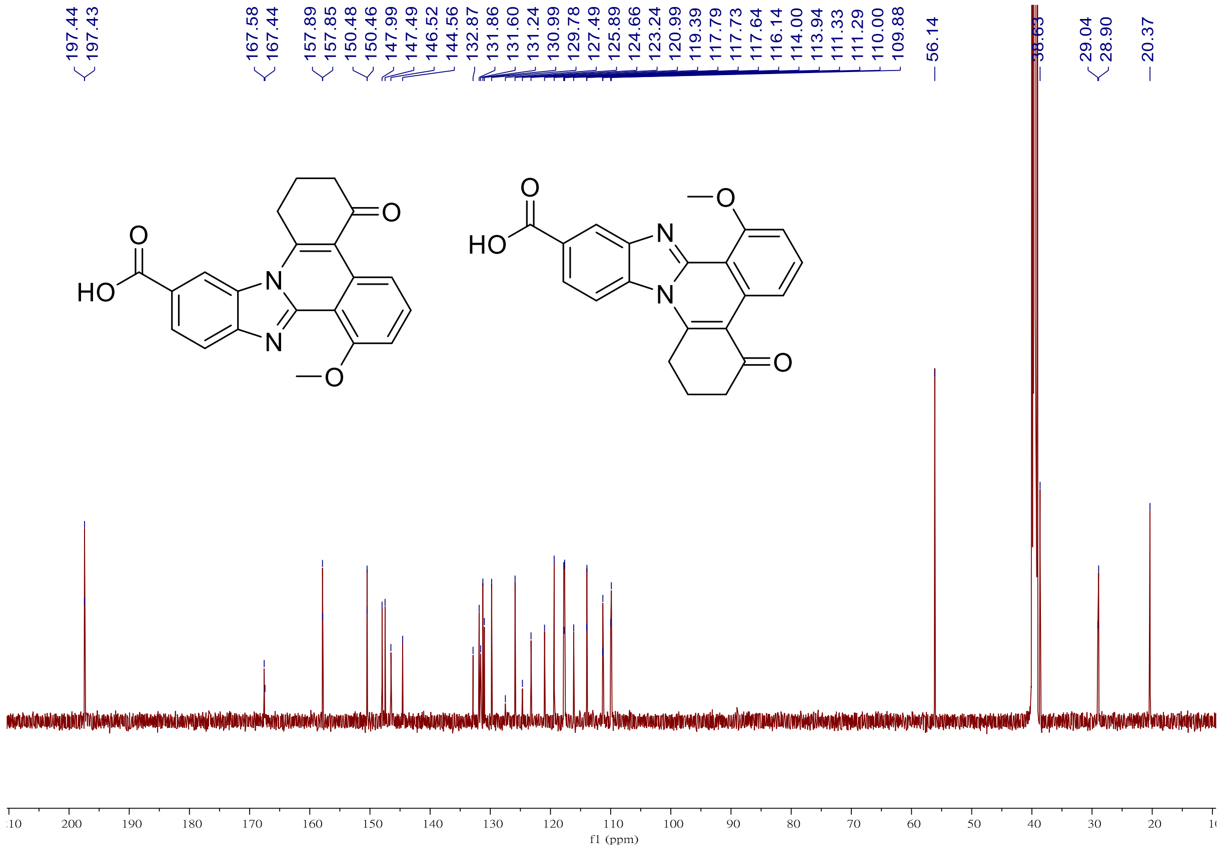

**Figure S250.** $^{13}C\{^{1}H\}$ NMR spectrum of compound **5e/5e'** (151 MHz, $(CD_3)_2SO$).

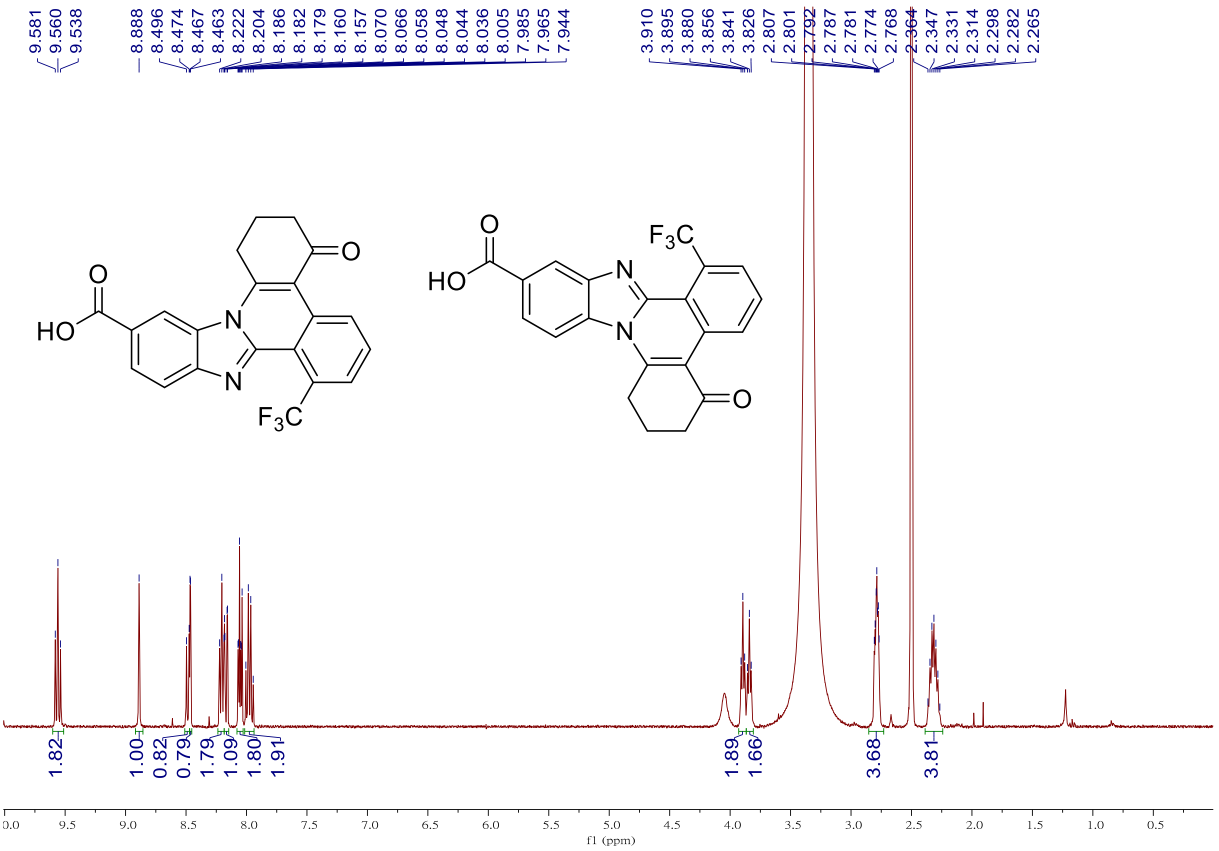


**Figure S251.** $^{1}$H NMR spectrum of compound **5h/5h'** (600 MHz, $(CD_3)_2SO$).

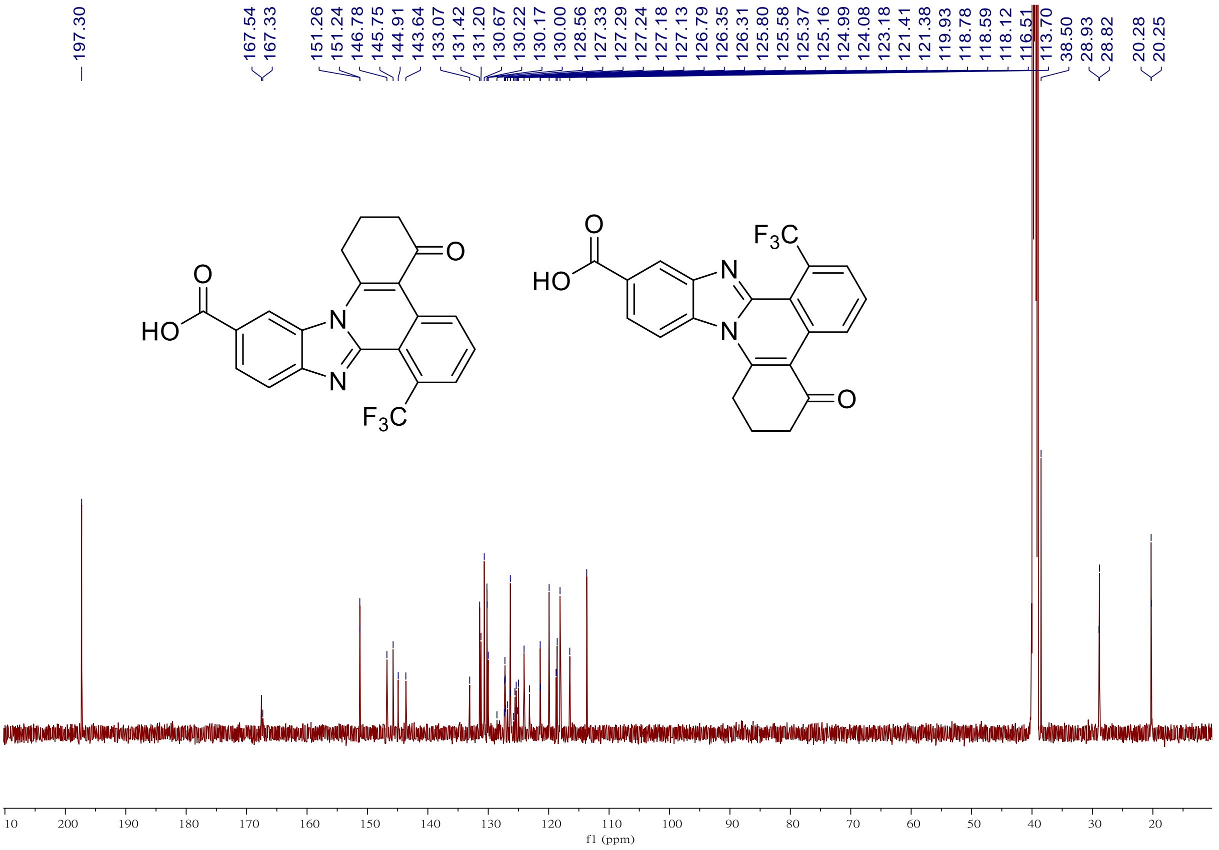


**Figure S252.** $^{13}C\{^{1}H\}$ NMR spectrum of compound **5h/5h'** (151 MHz, $(CD_3)_2SO$).

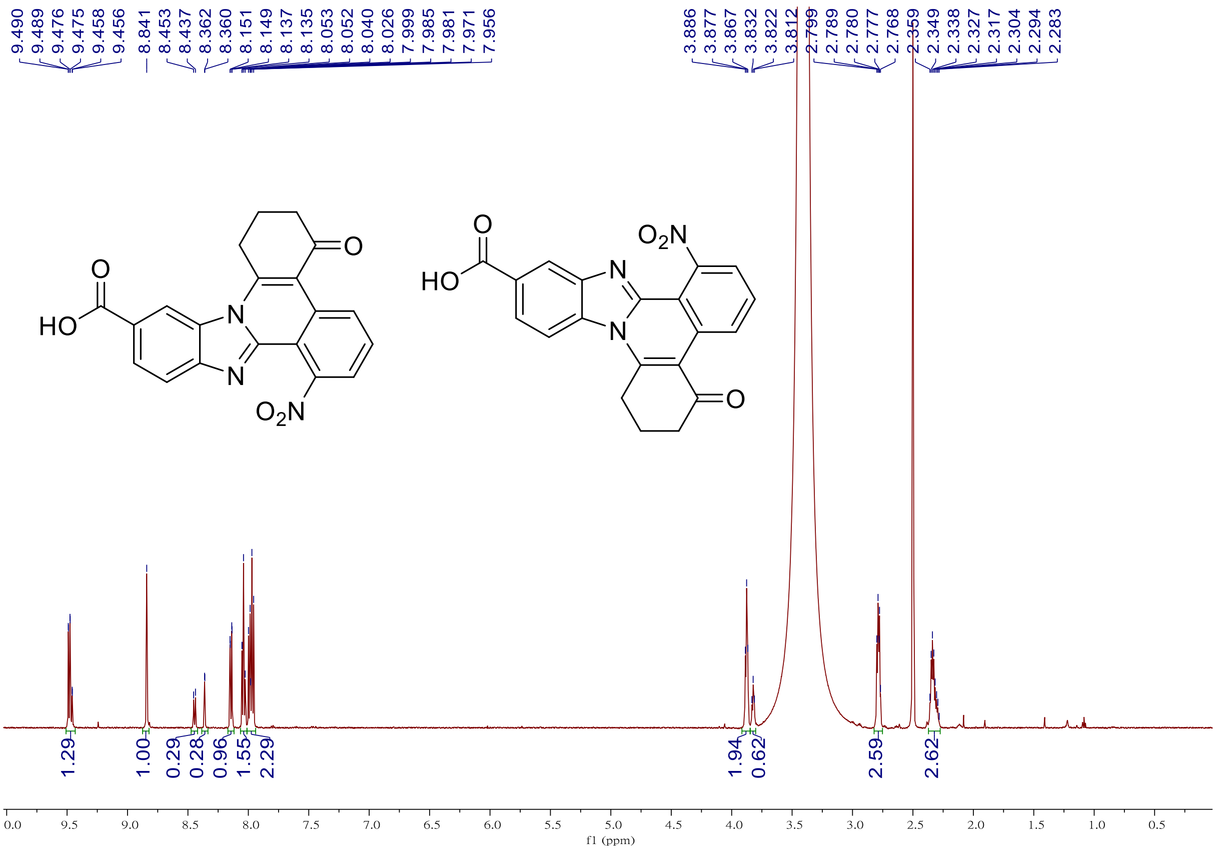


**Figure S253.** $^{1}$H NMR spectrum of compound **5i/5i’** (600 MHz, $(CD_3)_2SO$).

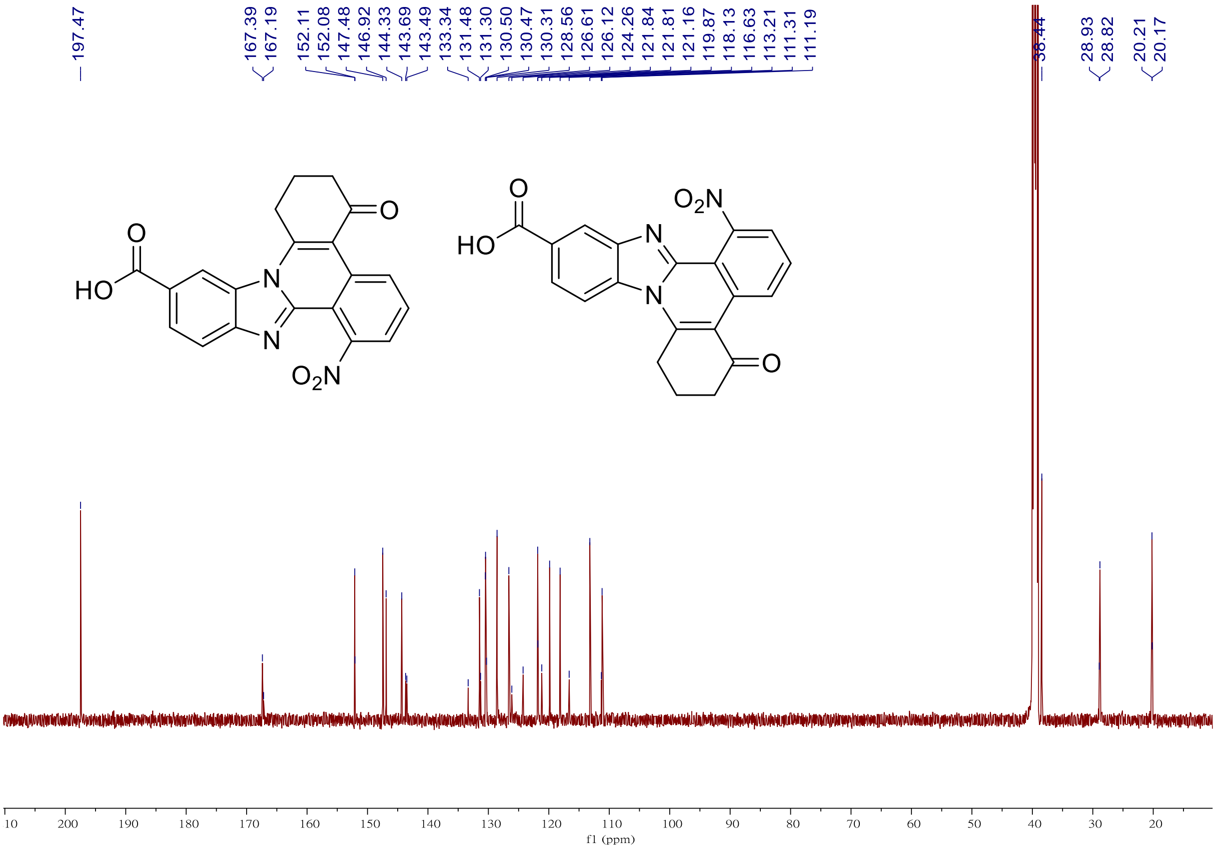


**Figure S254.** $^{13}$C{$^{1}$H} NMR spectrum of compound **5i/5i’** (151 MHz, $(CD_3)_2SO$).

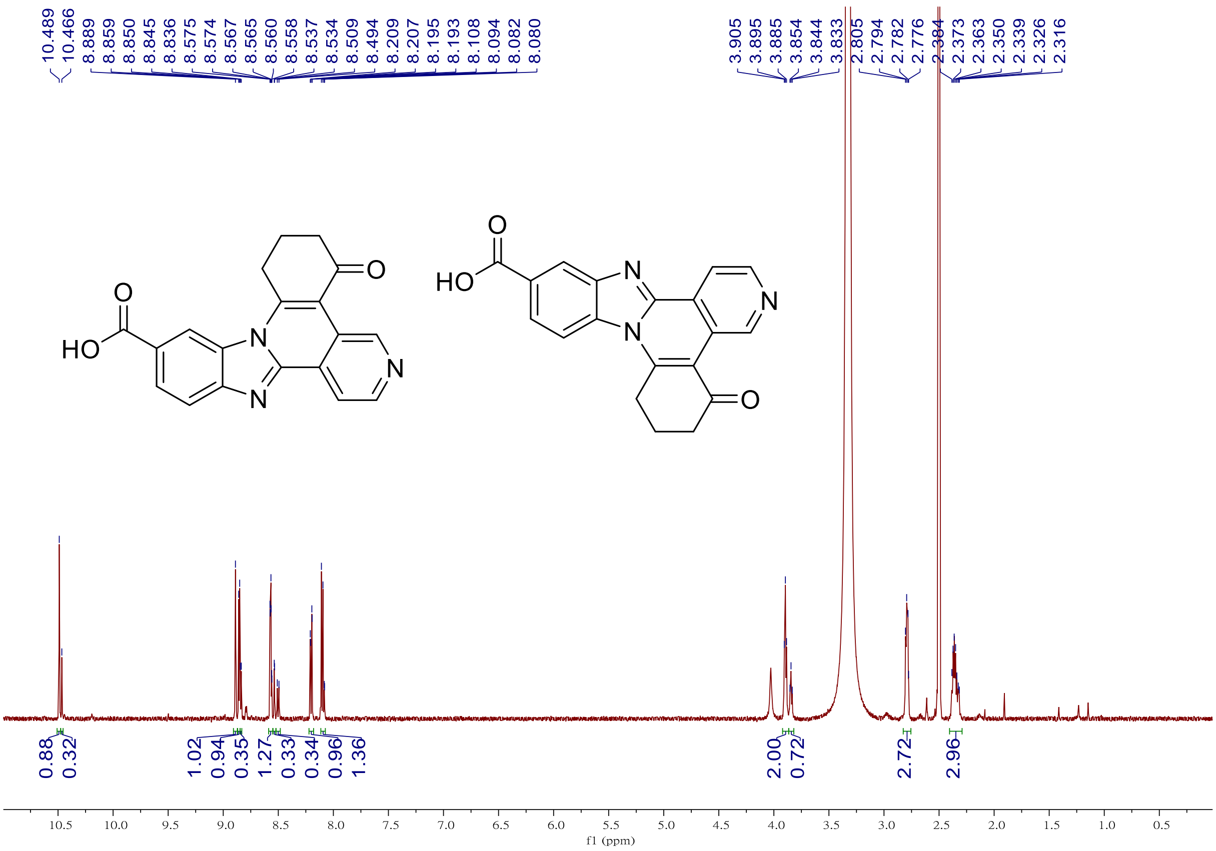

**Figure S255.** $^{1}H$ NMR spectrum of compound **5j/5j'** (600 MHz, $(CD_3)_2SO$).

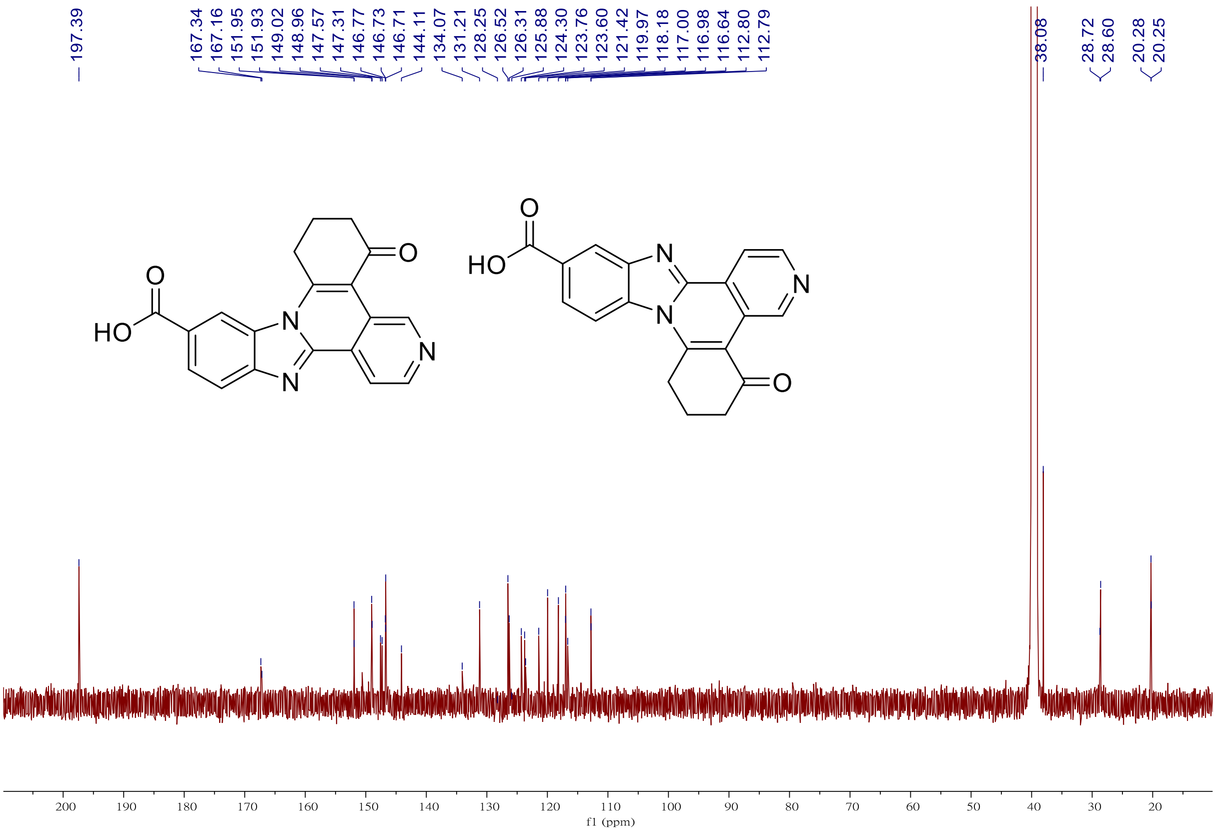

**Figure S256.** $^{13}C\{^{1}H\}$ NMR spectrum of compound **5j/5j'** (151 MHz, $(CD_3)_2SO$).

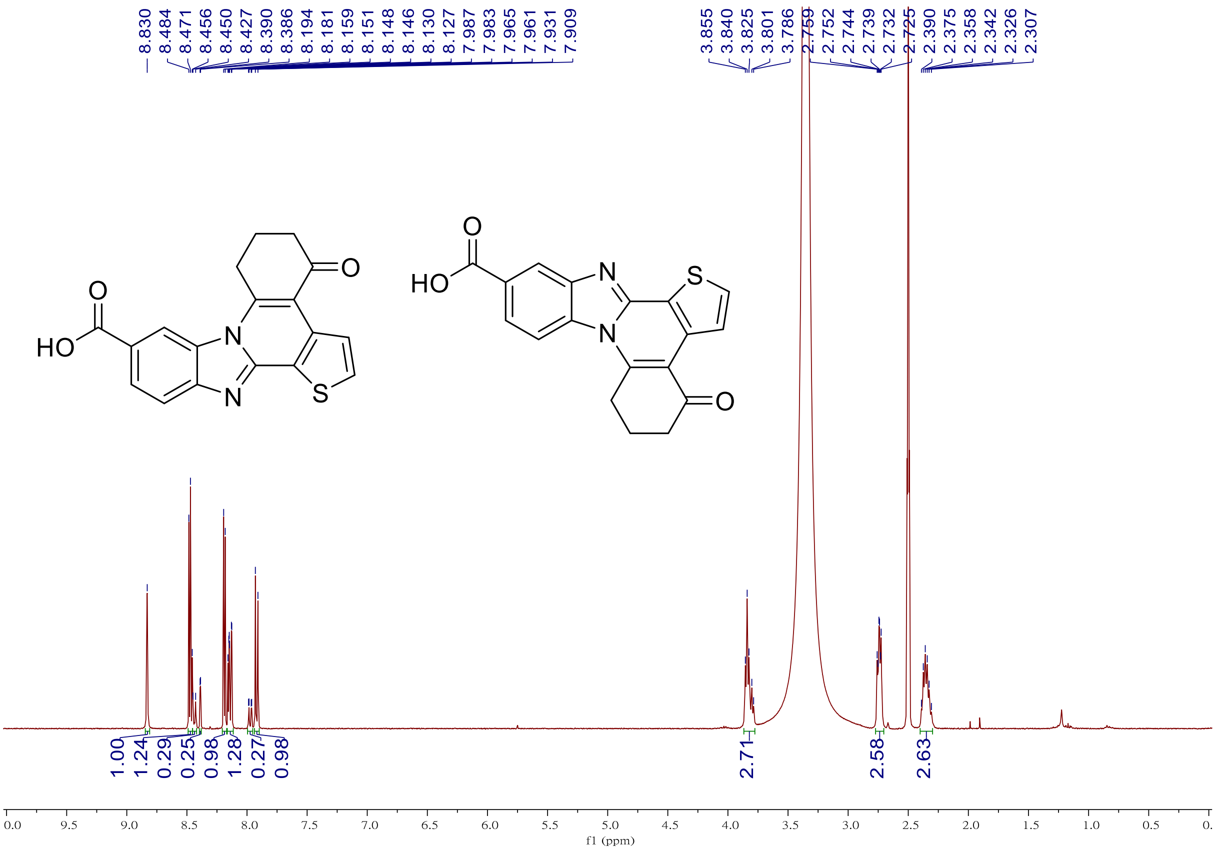

**Figure S257.** $^{1}H$ NMR spectrum of compound **5k/5k’** (400 MHz, $(CD_3)_2SO$).

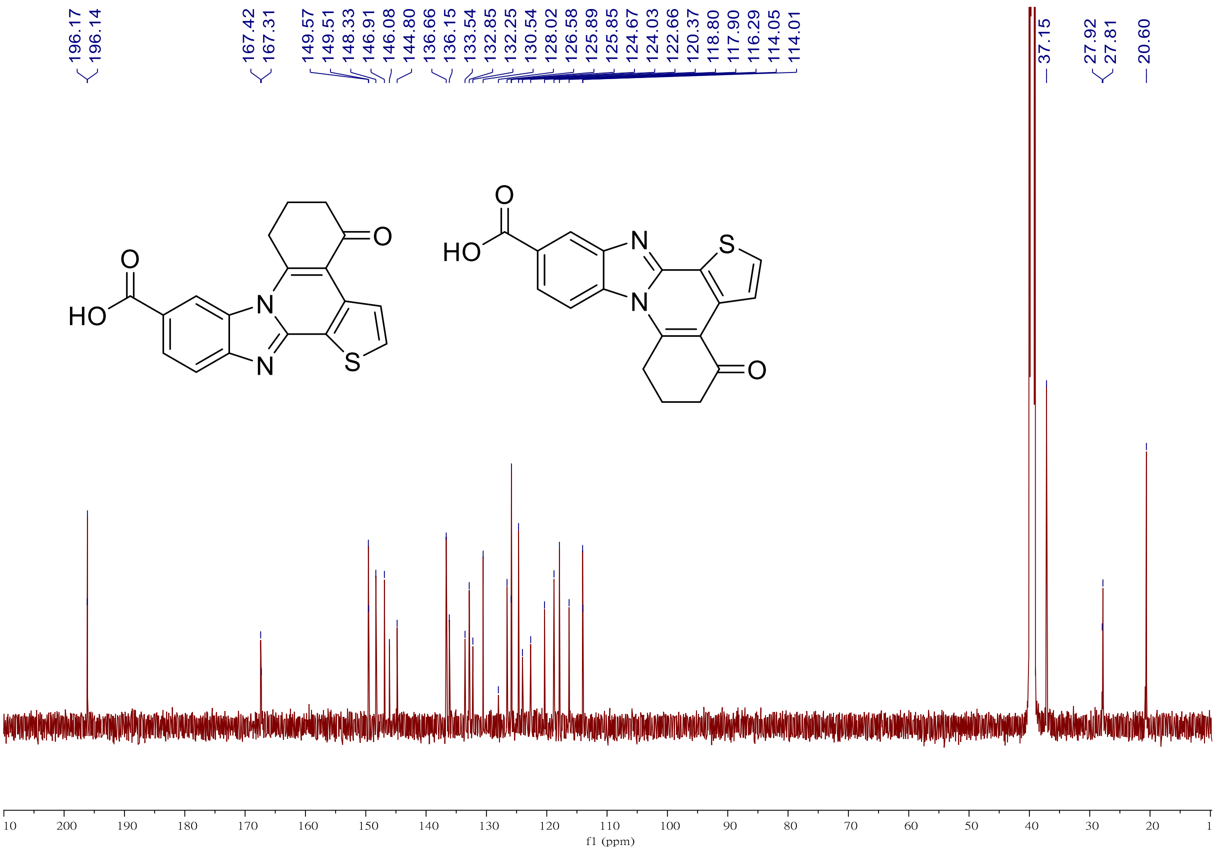

**Figure S258.** $^{13}C\{^{1}H\}$ NMR spectrum of compound **5k/5k’** (151 MHz, $(CD_3)_2SO$).

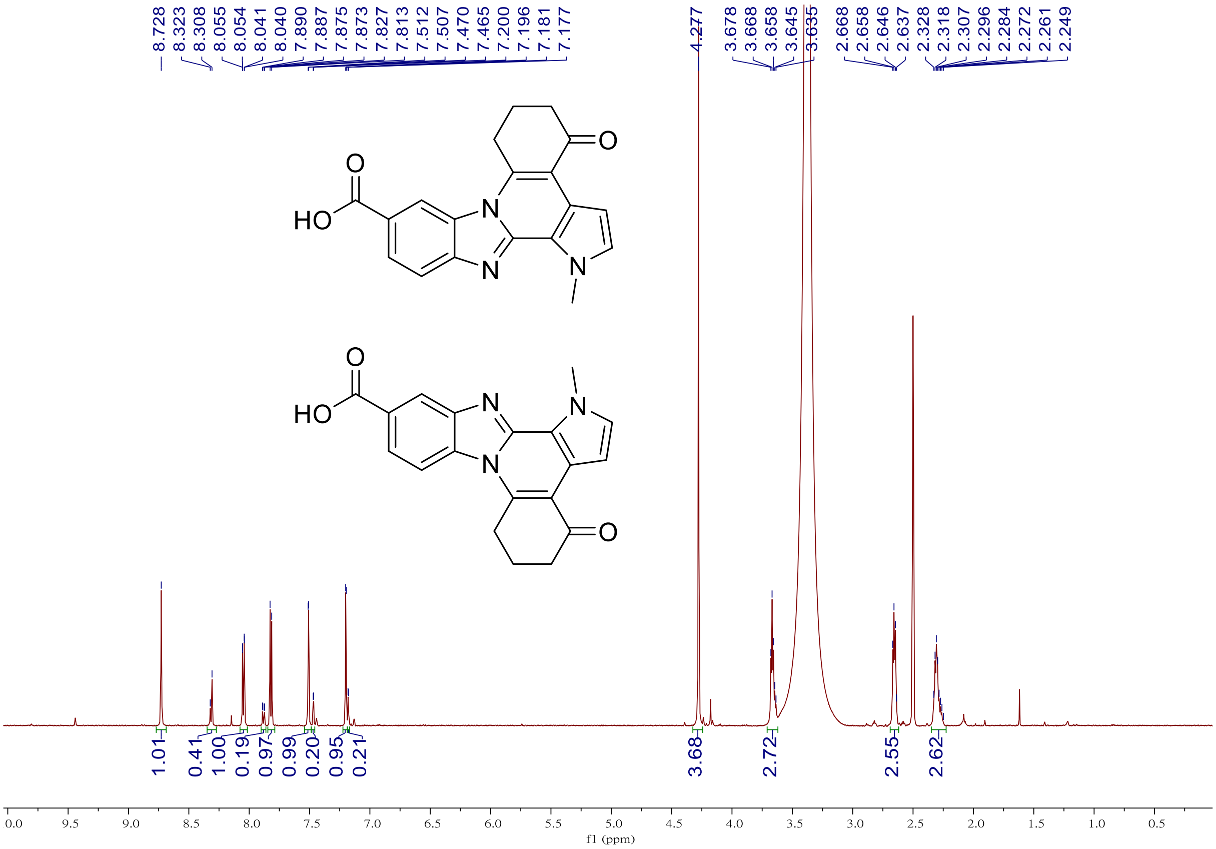


**Figure S259.** $^{1}$H NMR spectrum of compound **5l/5l'** (600 MHz, $(CD_3)_2SO$).

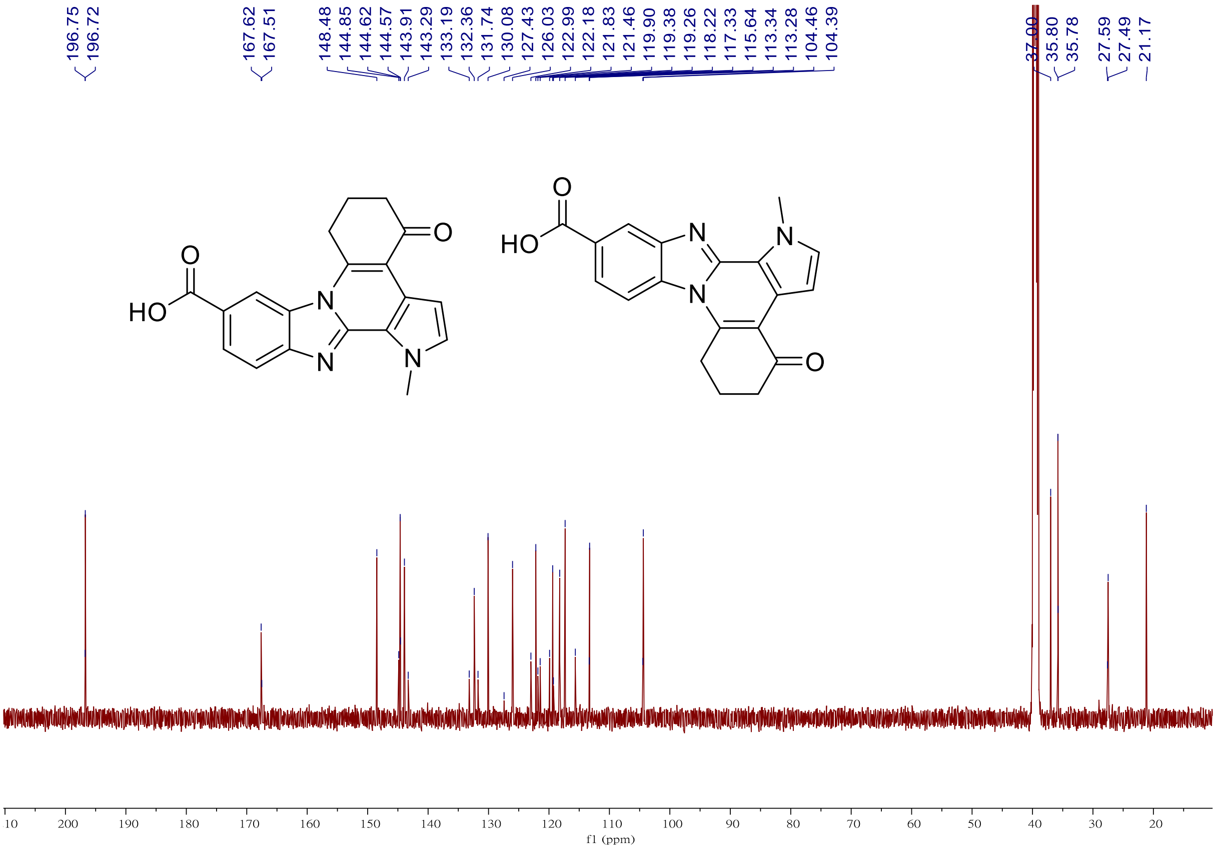


**Figure S260.** $^{13}C\{^{1}H\}$ NMR spectrum of compound **5l/5l'** (151 MHz, $(CD_3)_2SO$).

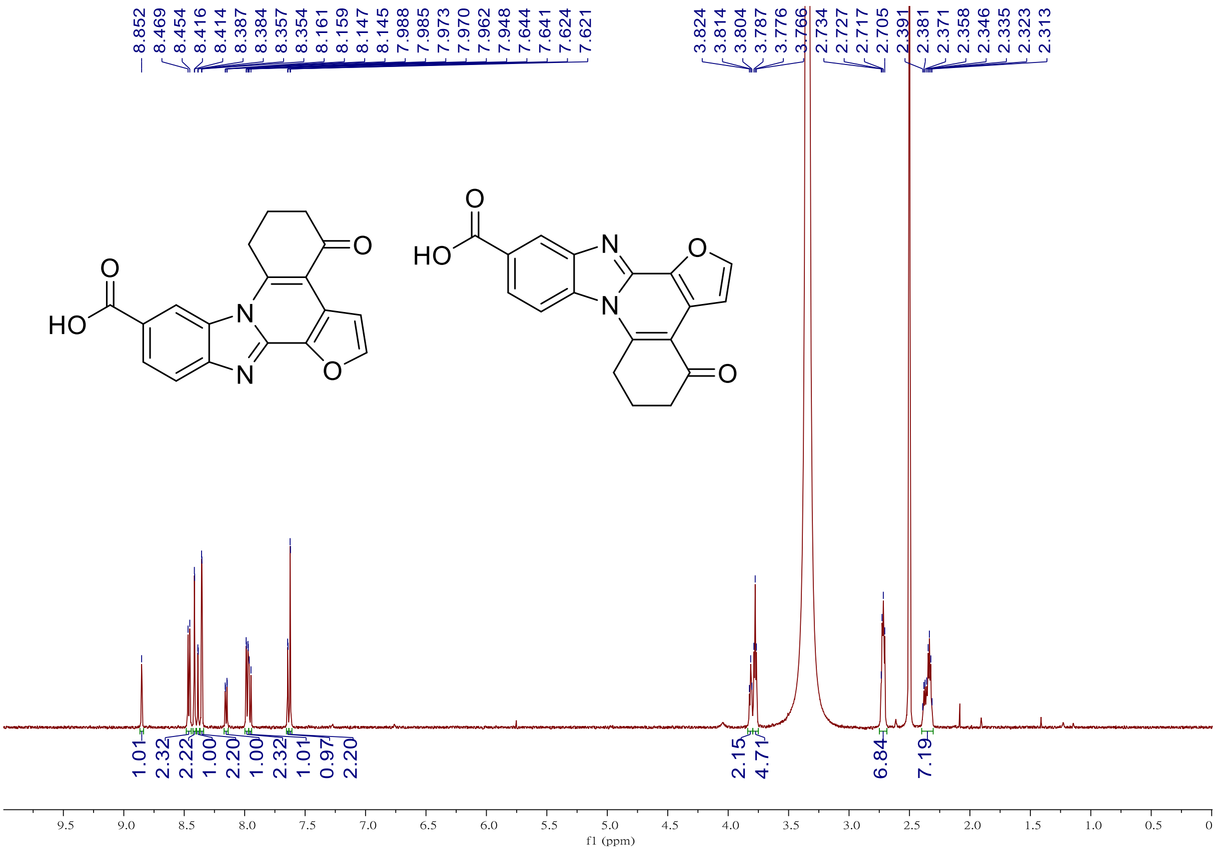


**Figure S261.** $^{1}H$ NMR spectrum of compound **5n/5n'** (600 MHz, $(CD_3)_2SO$).

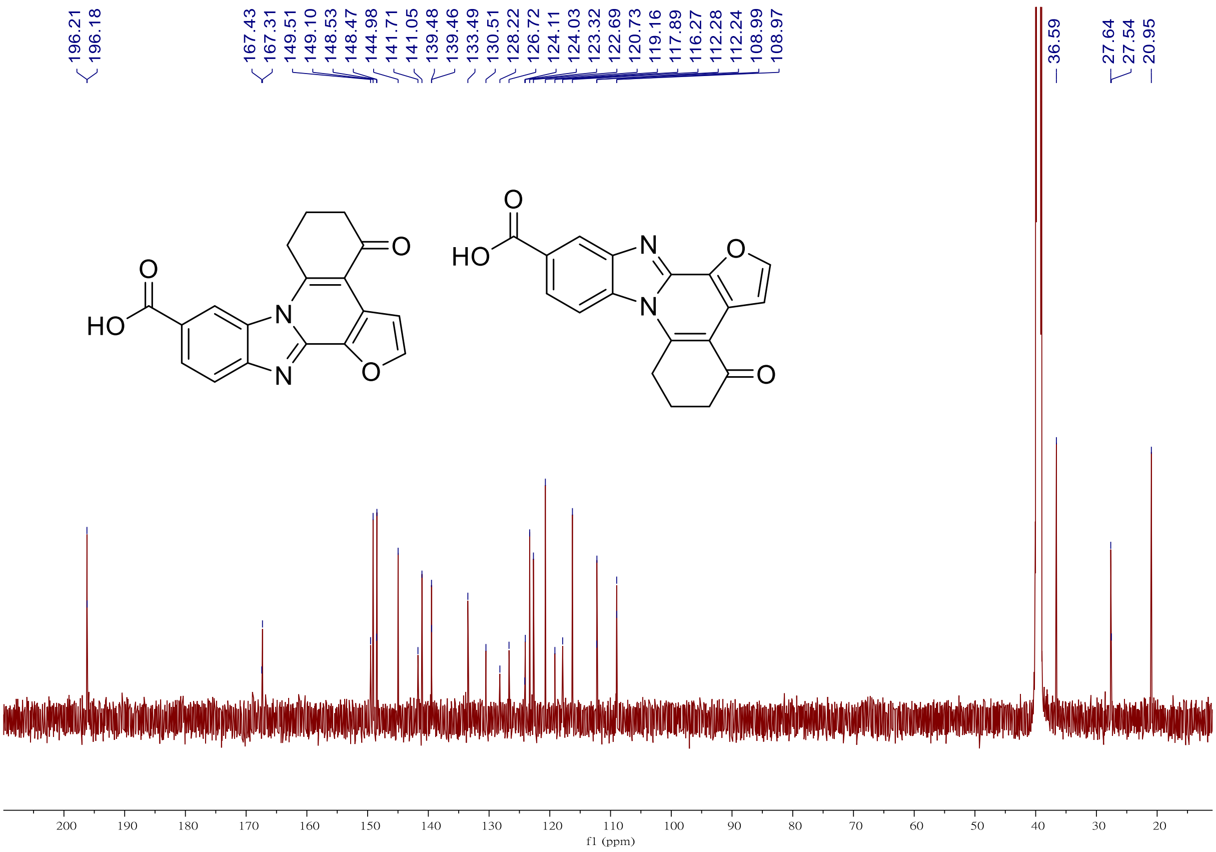


**Figure S262.** $^{13}C\{^{1}H\}$ NMR spectrum of compound **5n/5n'** (151 MHz, $(CD_3)_2SO$).

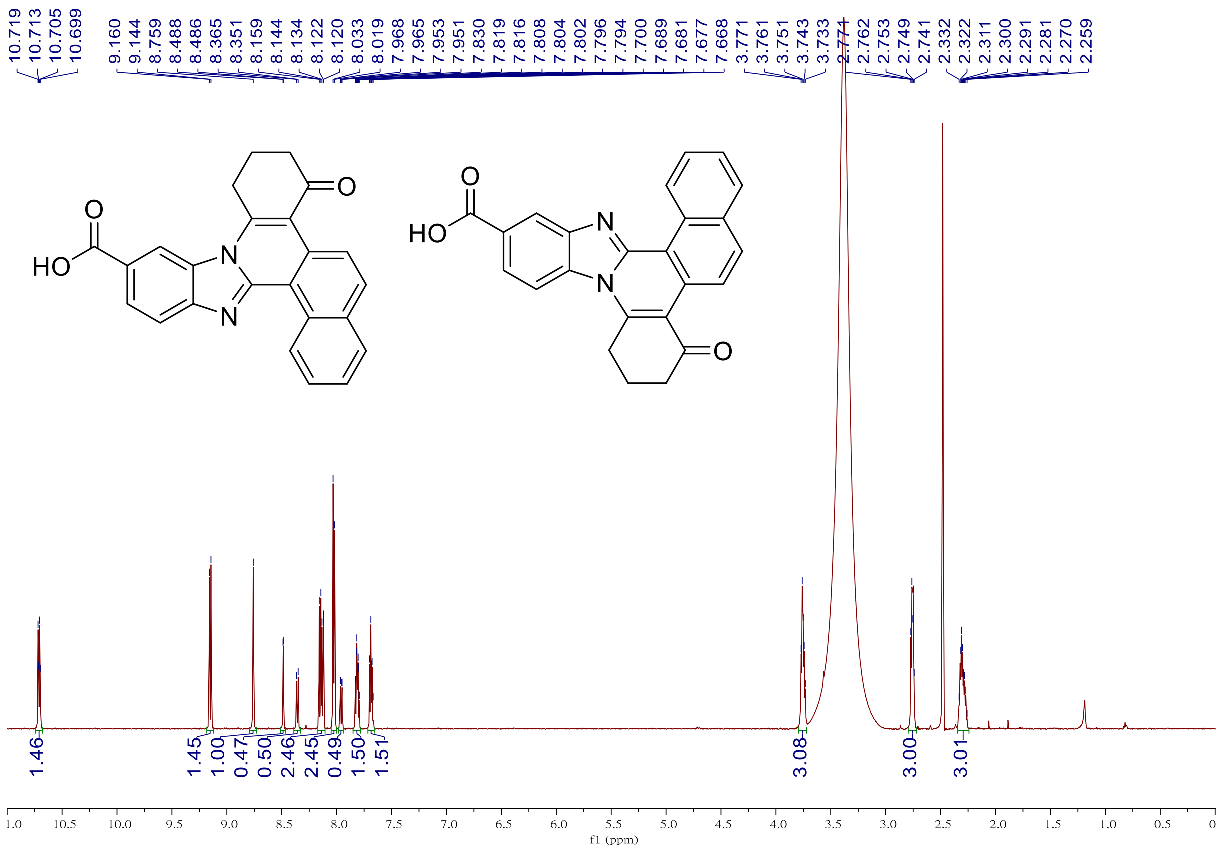

**Figure S263.** $^{1}$H NMR spectrum of compound **5o/5o'** (600 MHz, $(CD_3)_2SO$).

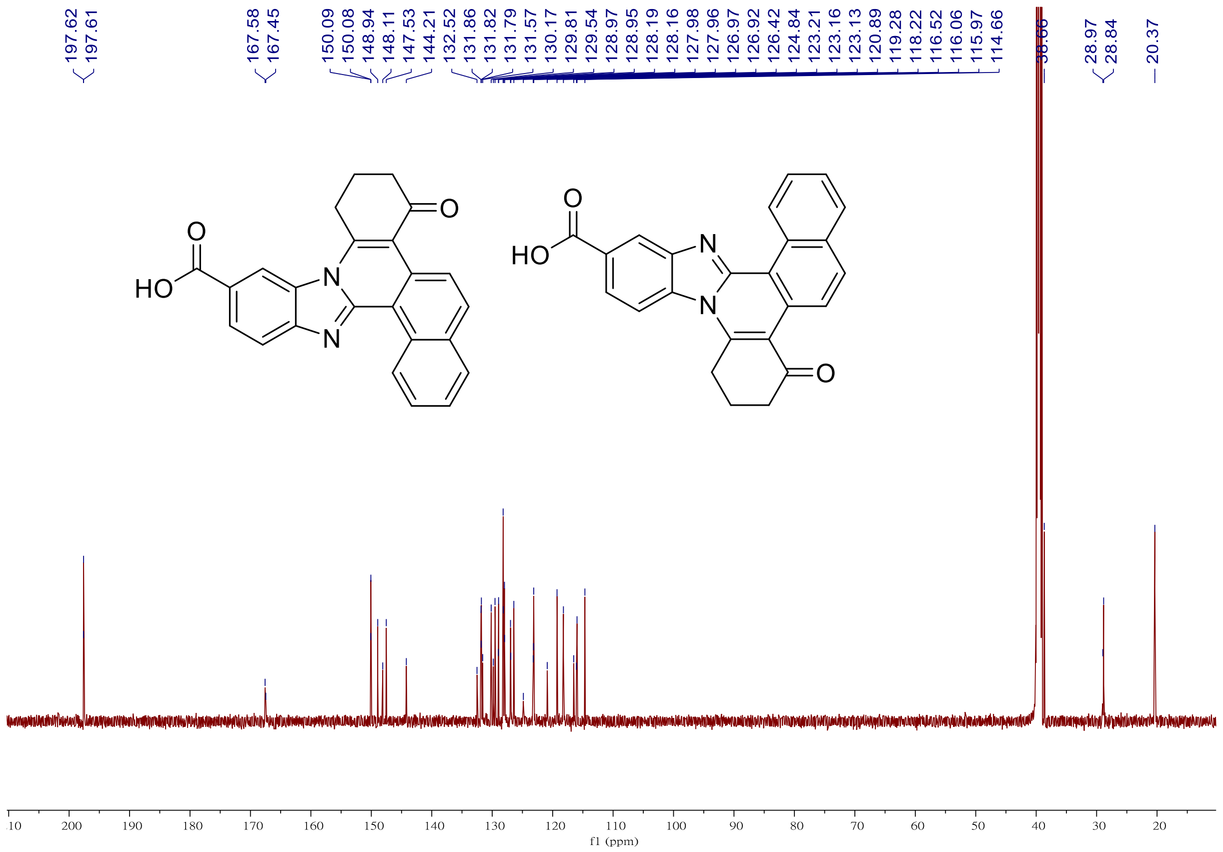

**Figure S264.** $^{13}C\{^{1}H\}$ NMR spectrum of compound **5o/5o'** (151 MHz, $(CD_3)_2SO$).

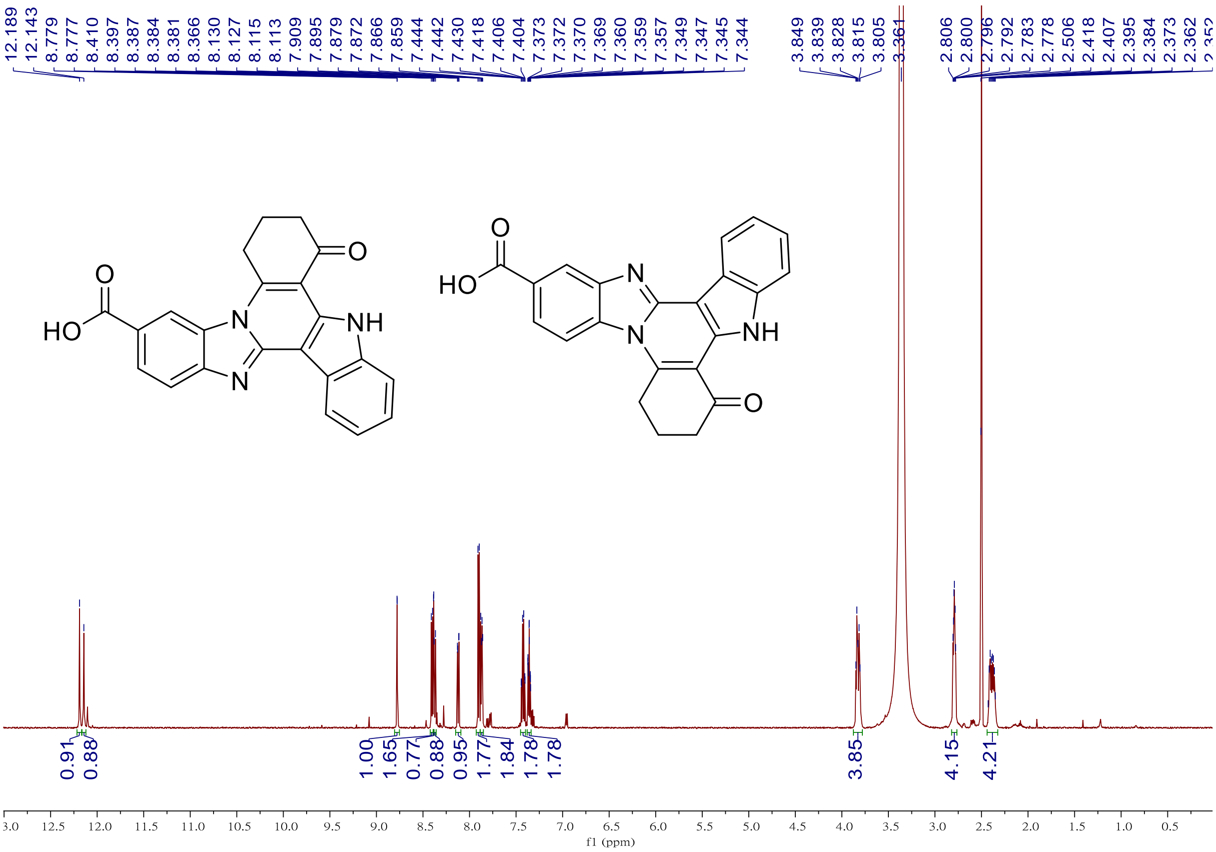


**Figure S265.** $^{1}H$ NMR spectrum of compound **5p/5p'** (600 MHz, $(CD_3)_2SO$).

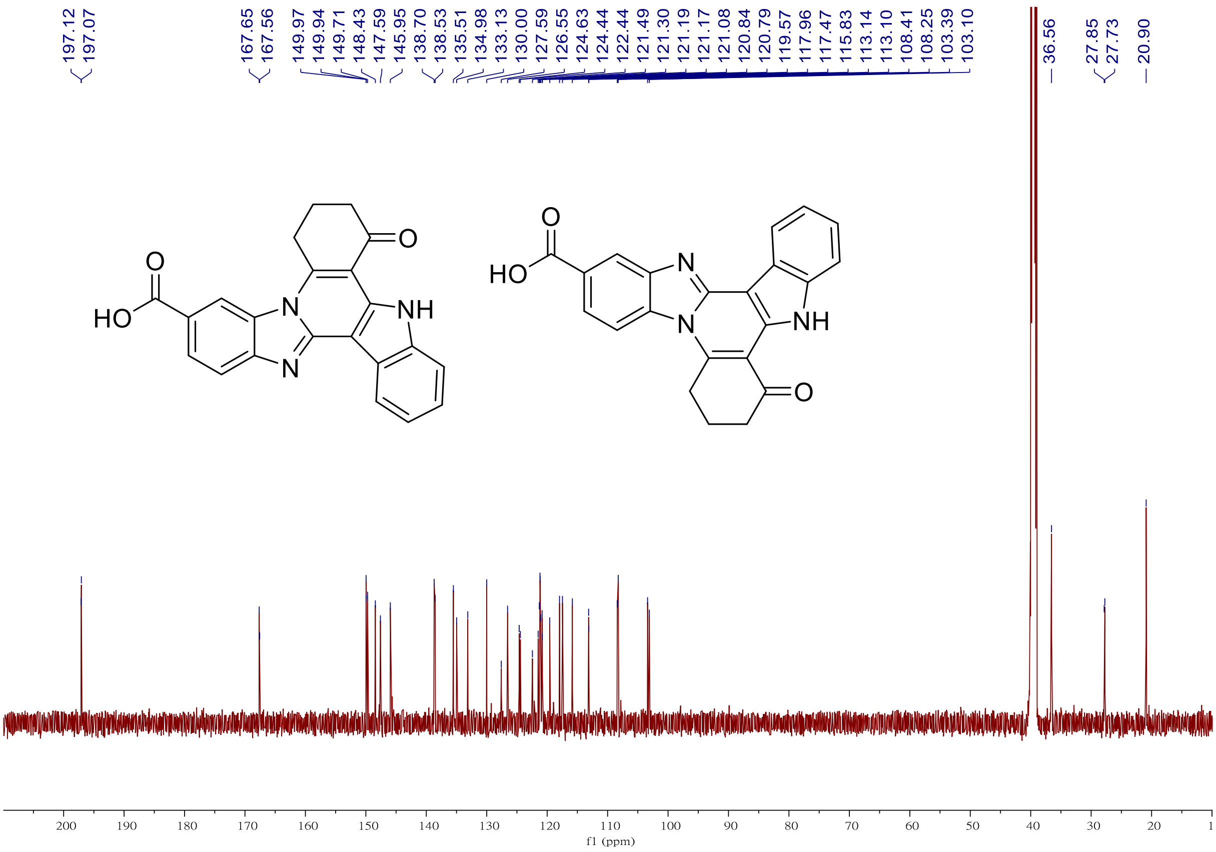


**Figure S266.** $^{13}C\{^{1}H\}$ NMR spectrum of compound **5p/5p'** (151 MHz, $(CD_3)_2SO$).

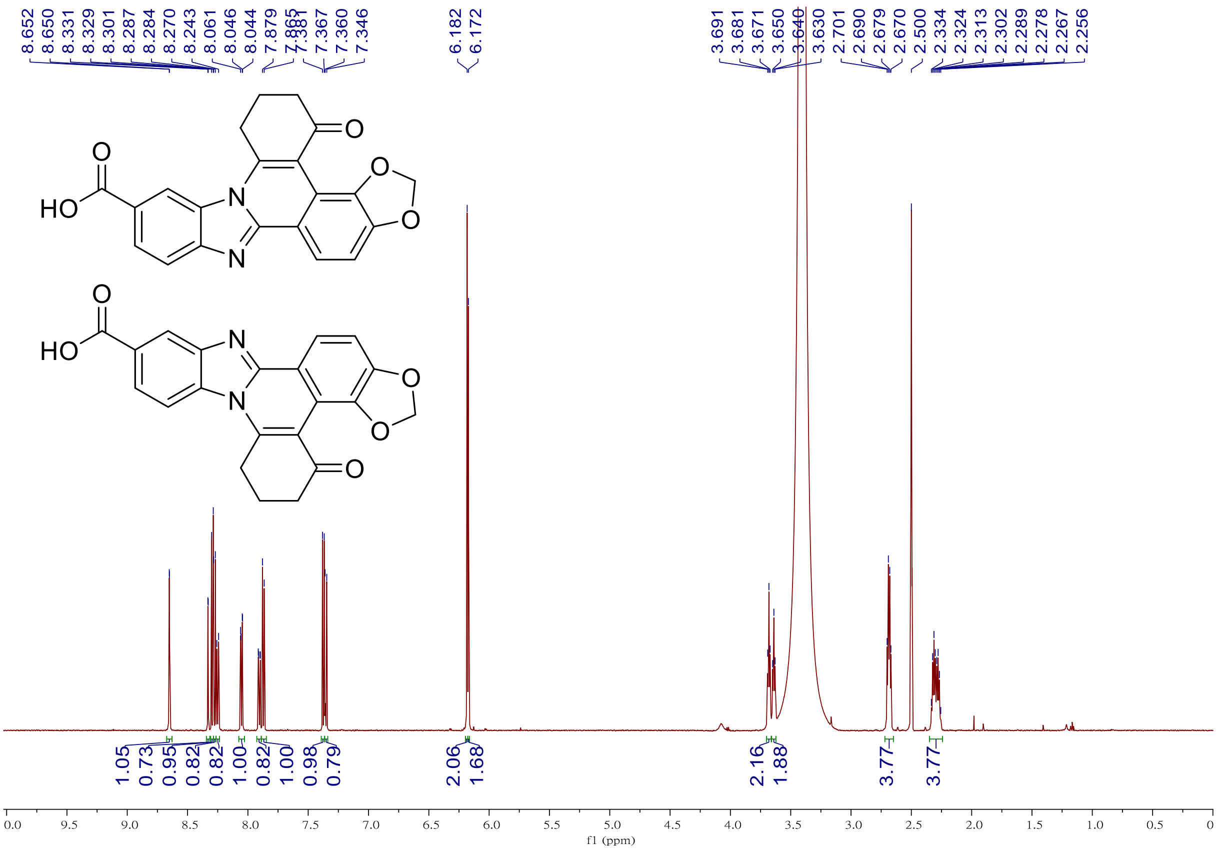


**Figure S267.** $^{1}$H NMR spectrum of compound **5q/5q'** (600 MHz, $(CD_3)_2SO$).

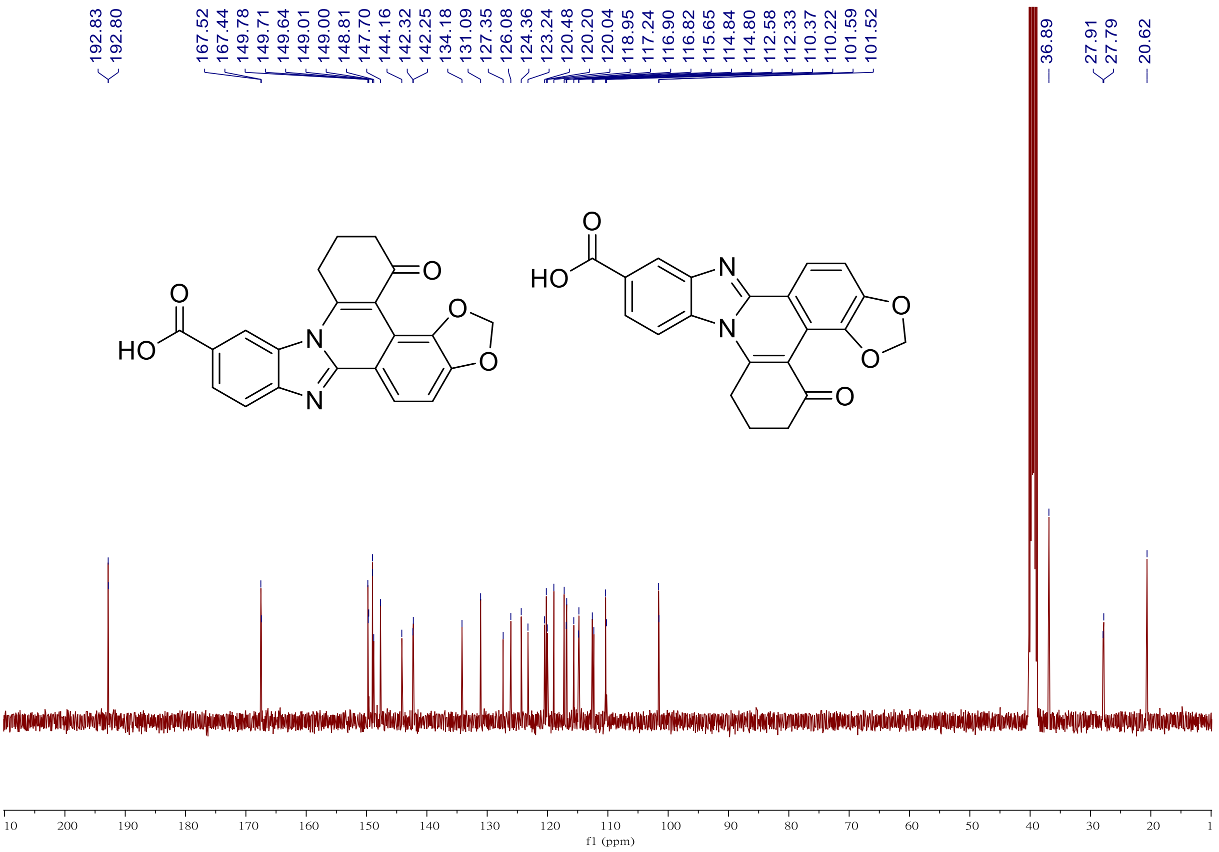


**Figure S268.** $^{13}C\{^{1}H\}$ NMR spectrum of compound **5q/5q'** (151 MHz, $(CD_3)_2SO$).

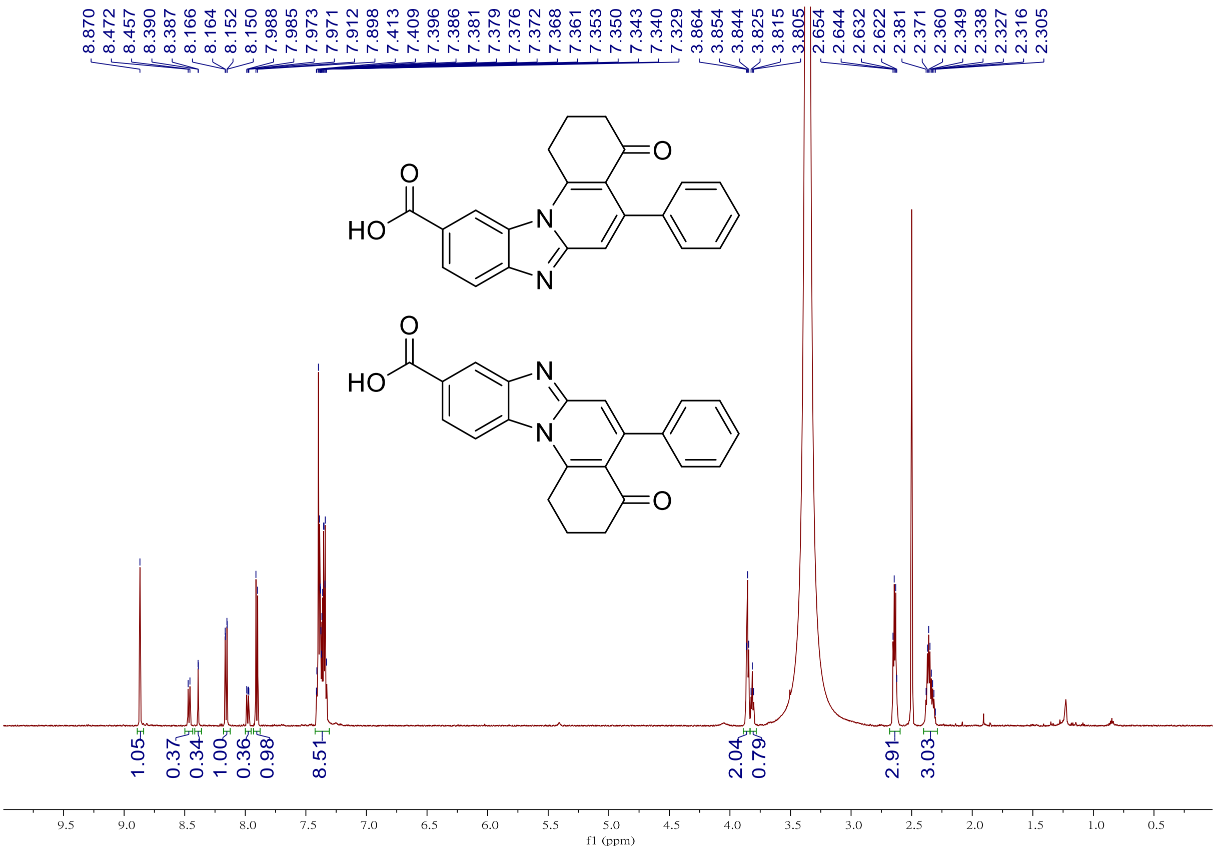


**Figure S269.** $^{1}$H NMR spectrum of compound **5r/5r'** (600 MHz, $(CD_3)_2SO$).

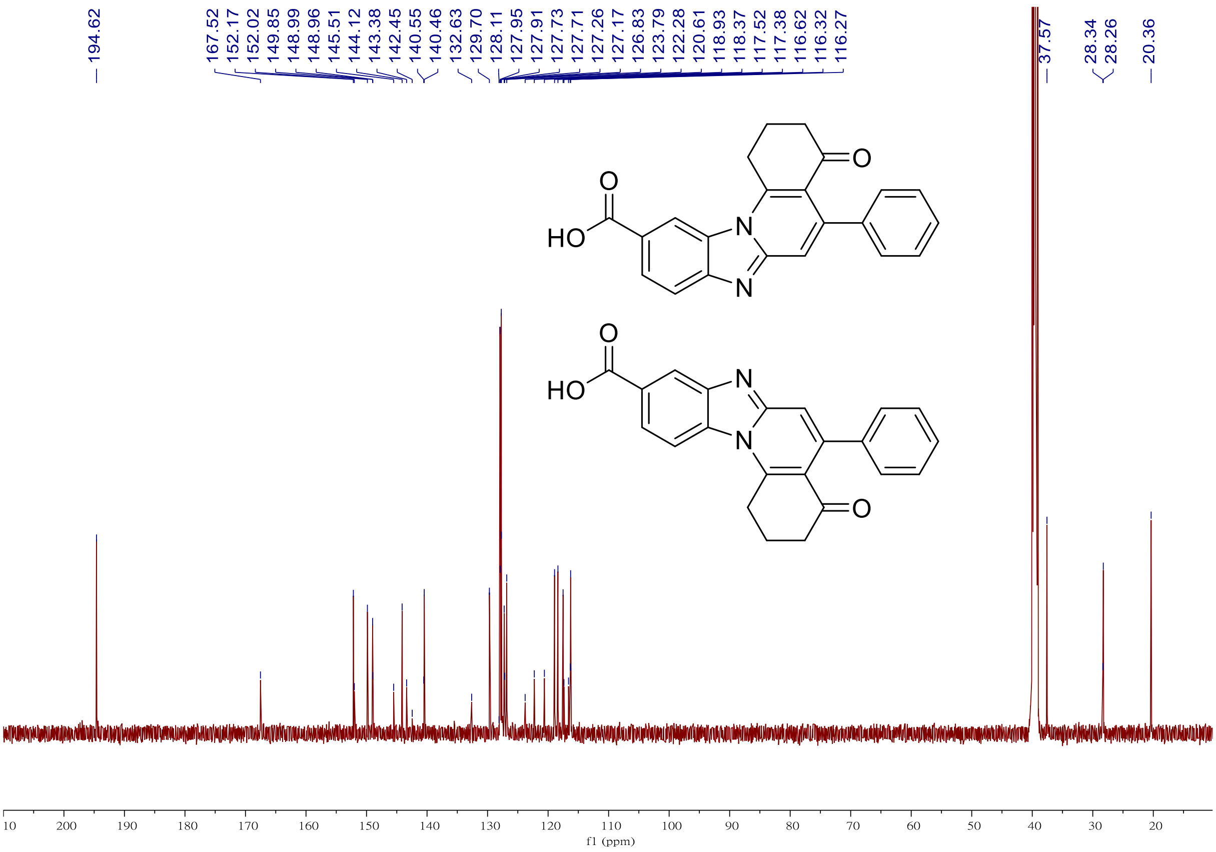


**Figure S270.** $^{13}C\{^{1}H\}$ NMR spectrum of compound **5r/5r'** (151 MHz, $(CD_3)_2SO$).

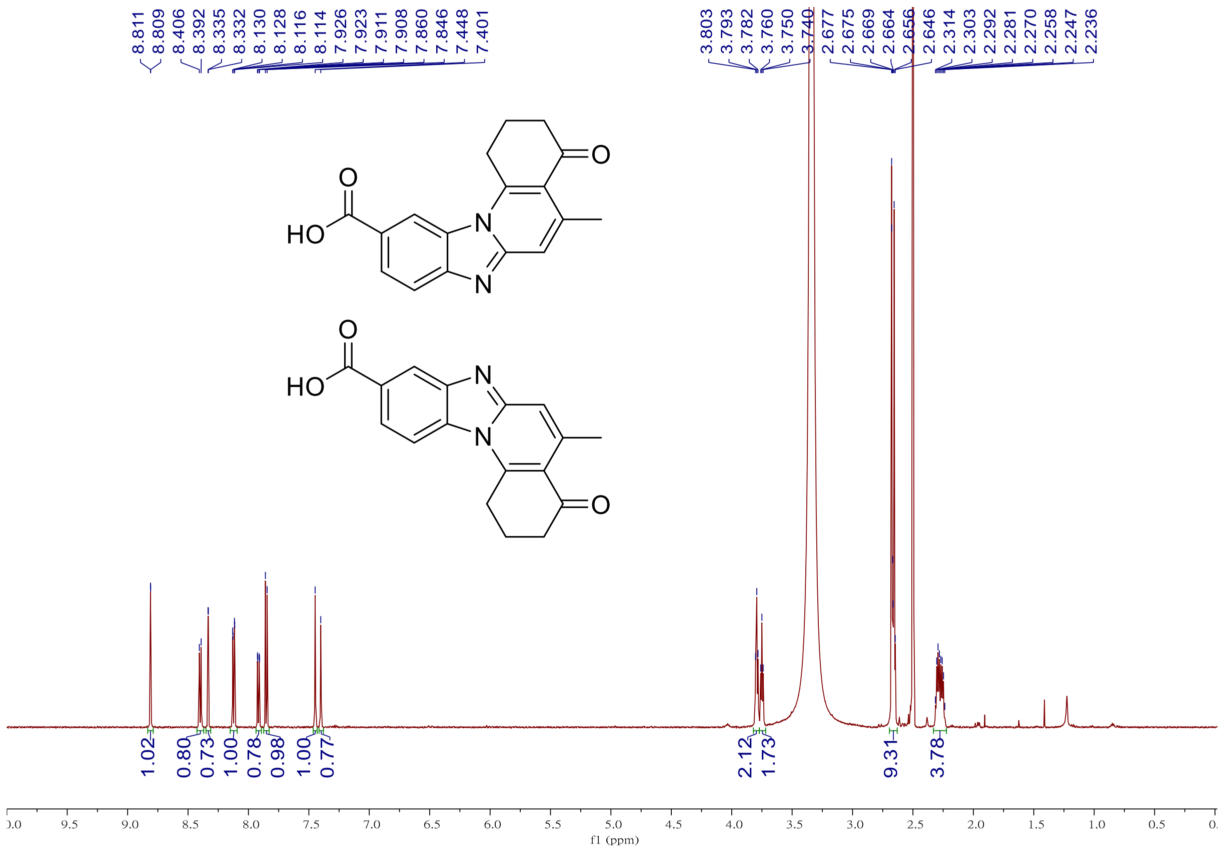

**Figure S271.** $^{1}$H NMR spectrum of compound **5s/5s'** (600 MHz, $(CD_3)_2SO$).

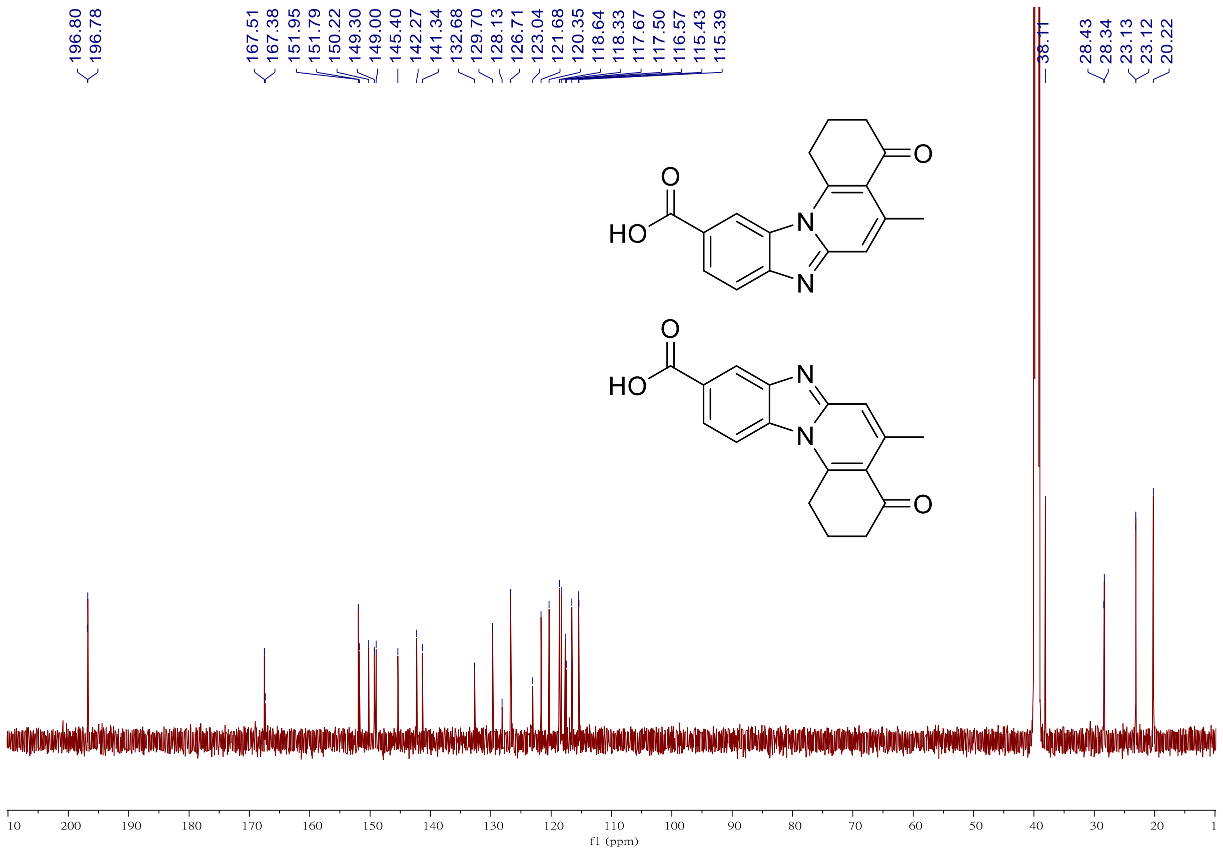

**Figure S272.** $^{13}C\{^{1}H\}$ NMR spectrum of compound **5s/5s'** (151 MHz, $(CD_3)_2SO$).

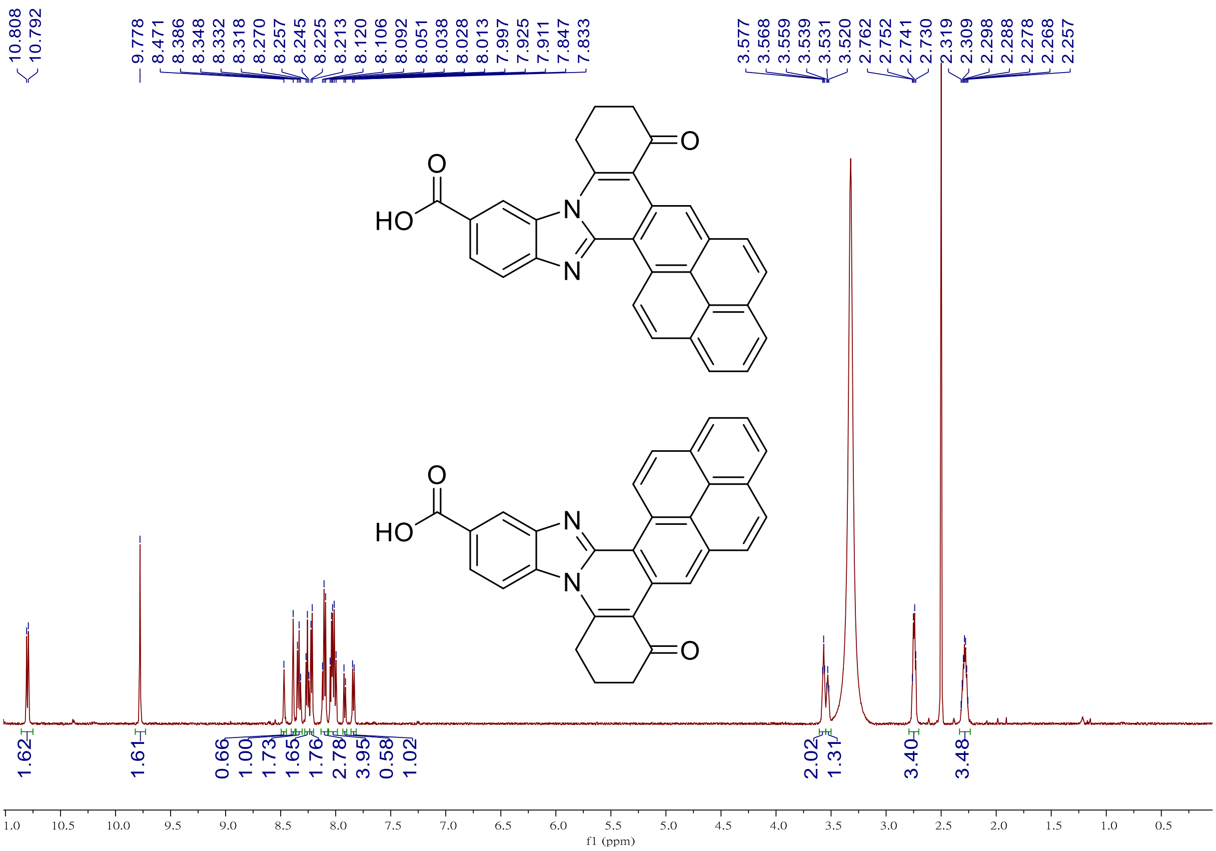


**Figure S273.** $^{1}$H NMR spectrum of compound **5t/5t’** (600 MHz, $(CD_3)_2SO$).

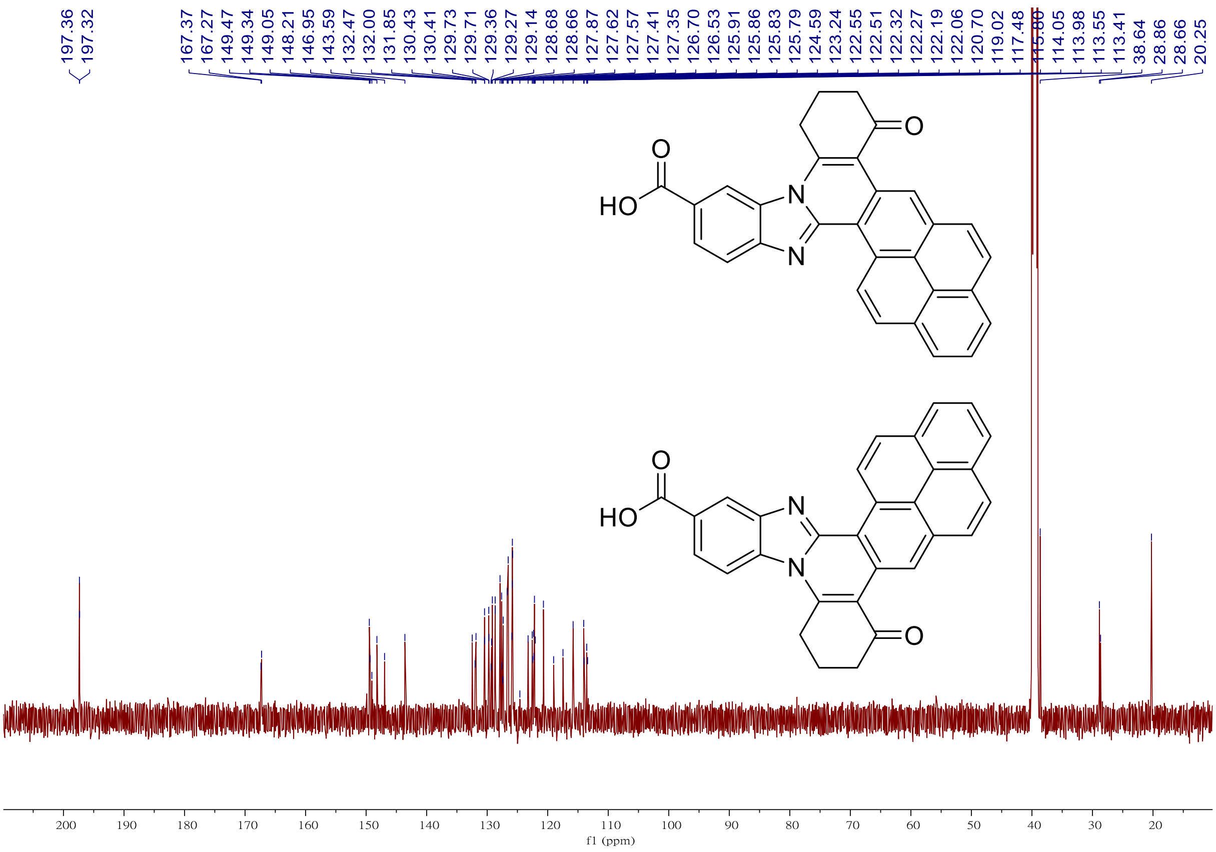


**Figure S274.** $^{13}C\{^{1}H\}$ NMR spectrum of compound **5t/5t’** (151 MHz, $(CD_3)_2SO$).

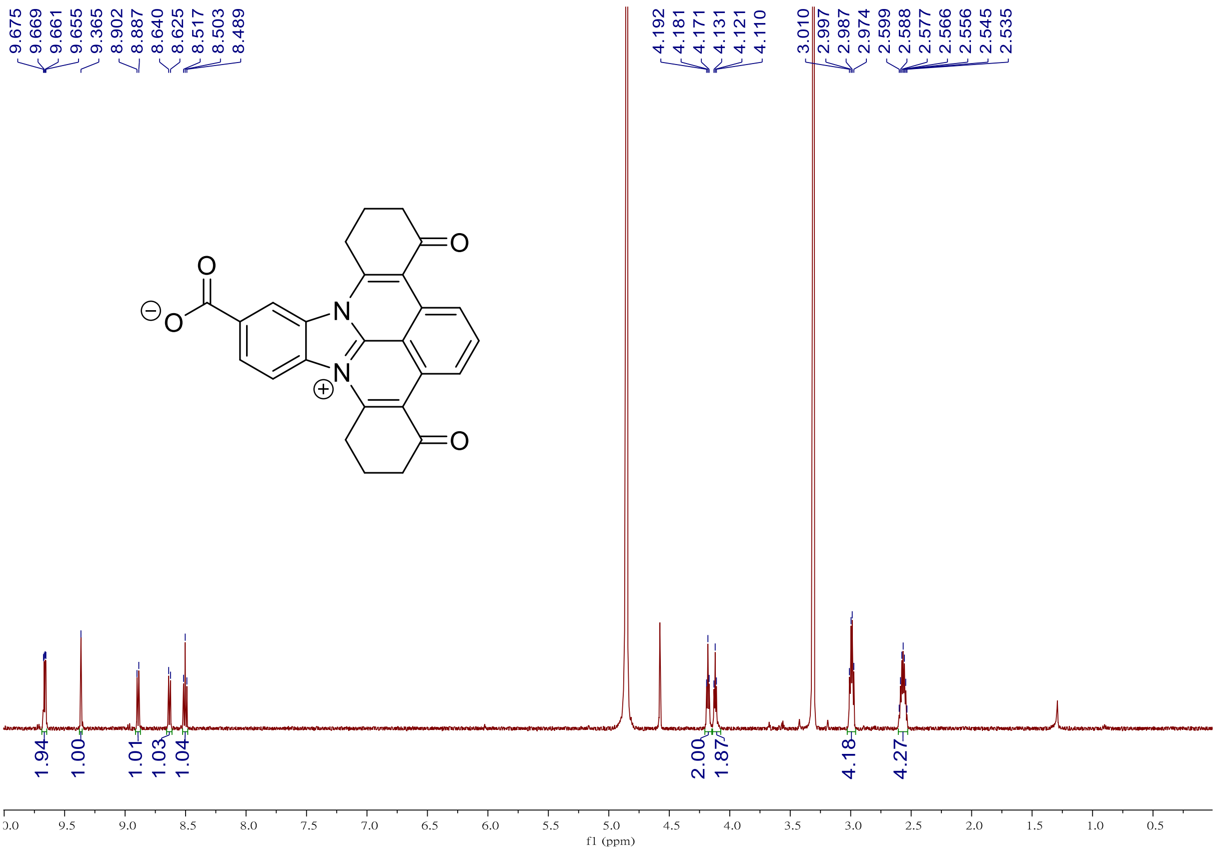


**Figure S275.** $^{1}$H NMR spectrum of compound **6a** (600 MHz, $CD_3OD$).

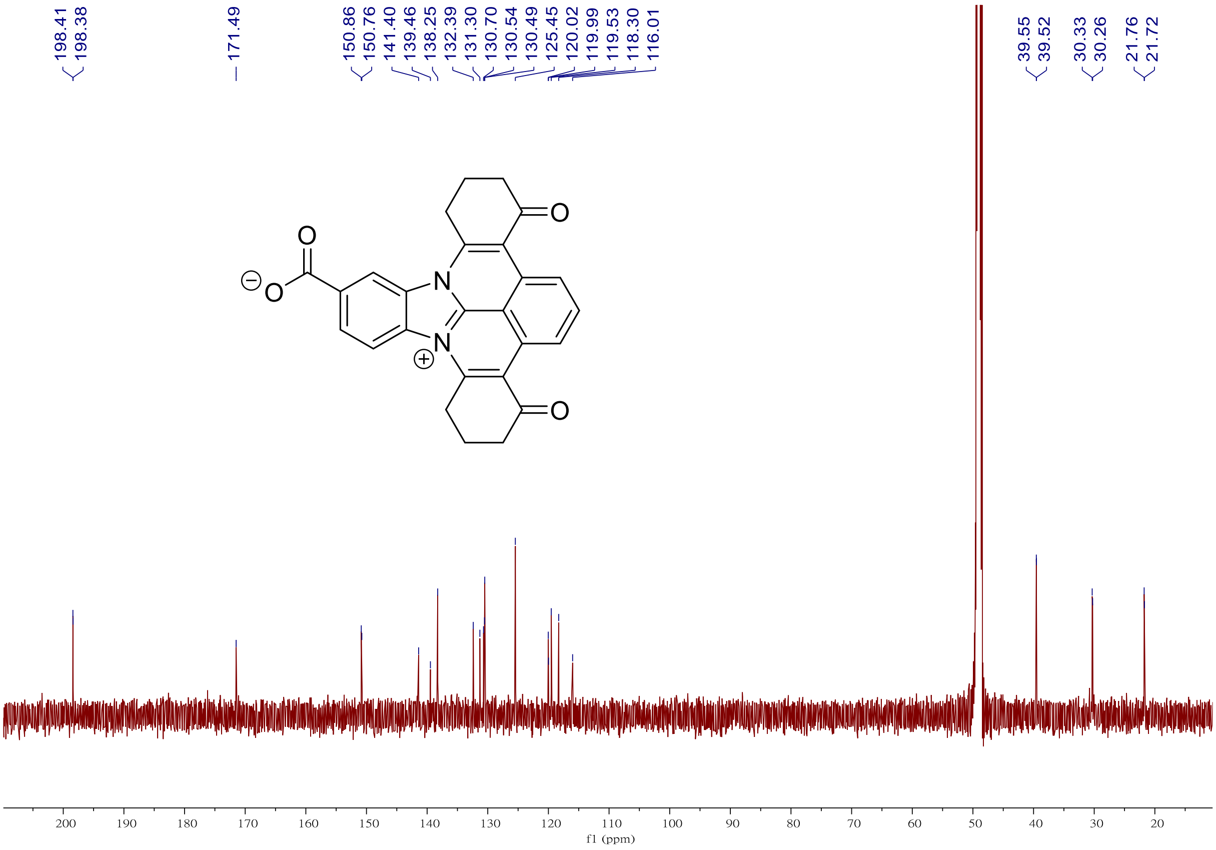


**Figure S276.** $^{13}C\{^{1}H\}$ NMR spectrum of compound **6a** (151 MHz, $CD_3OD$).

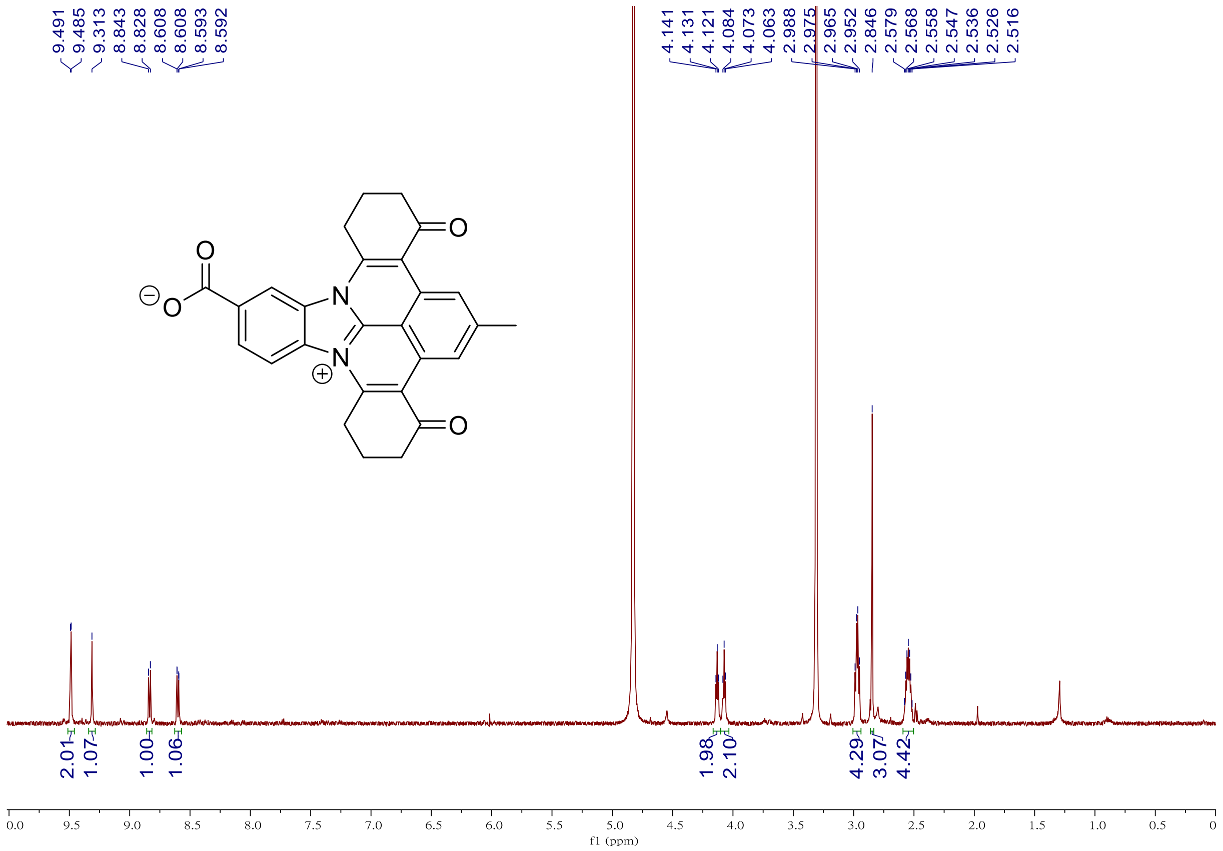


**Figure S277.** $^{1}H$ NMR spectrum of compound **6c** (600 MHz, $CD_3OD$).

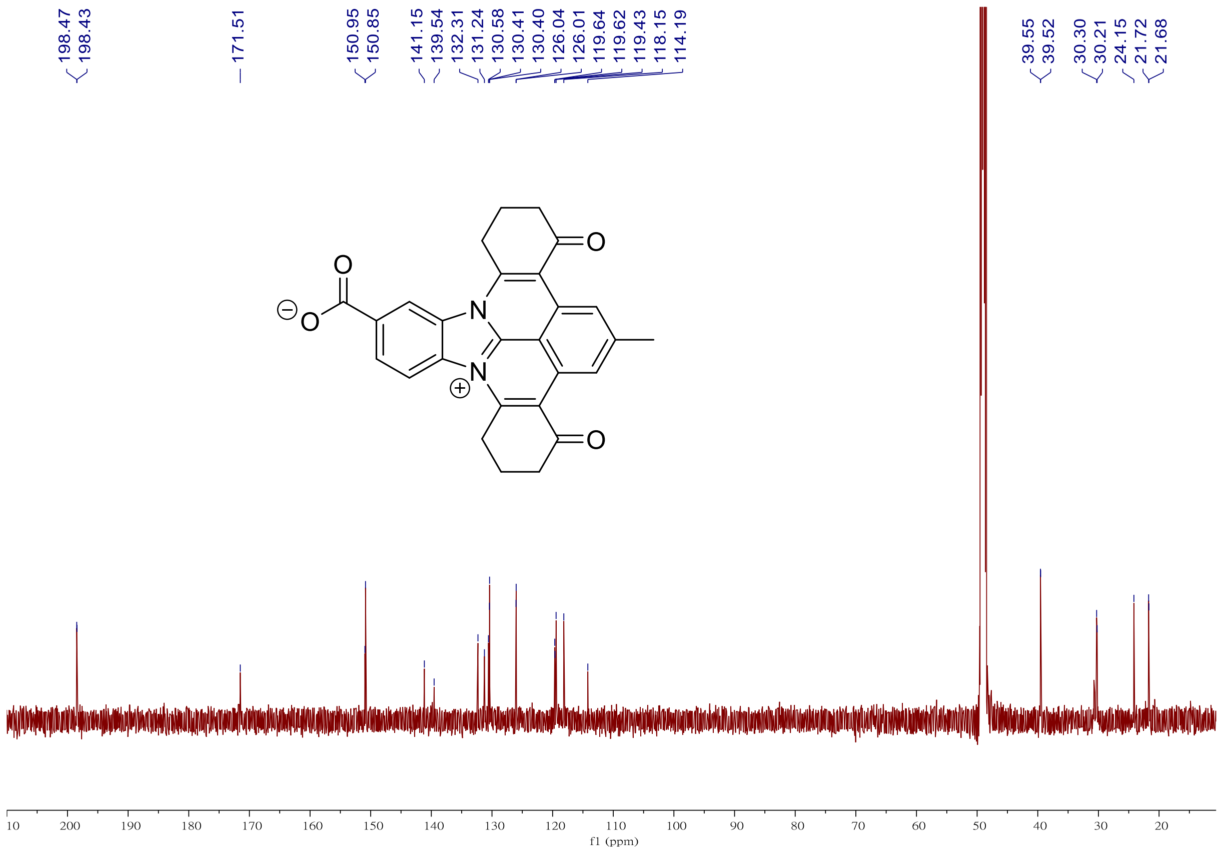


**Figure S278.** $^{13}C\{^{1}H\}$ NMR spectrum of compound **6c** (151 MHz, $CD_3OD$).

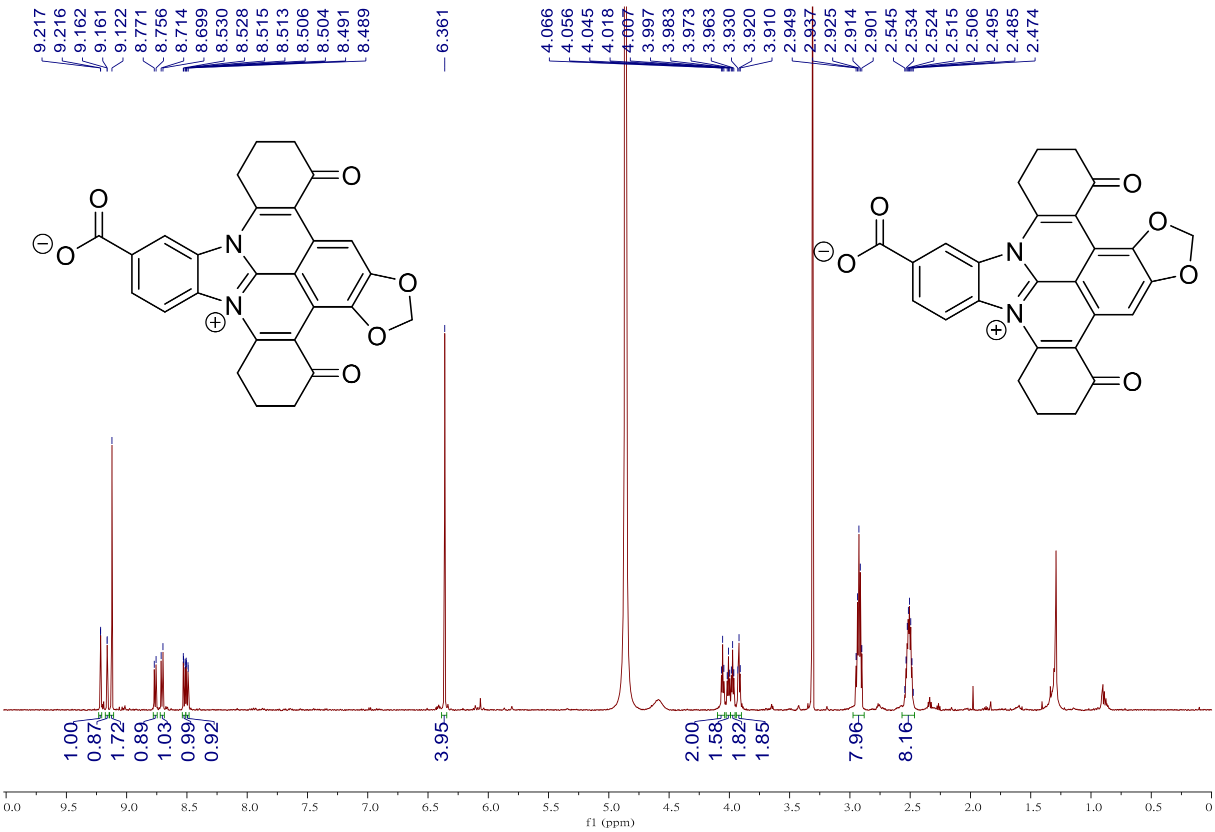

**Figure S279.** $^{1}$H NMR spectrum of compound **6q/6q'** (600 MHz, $CD_3OD$)

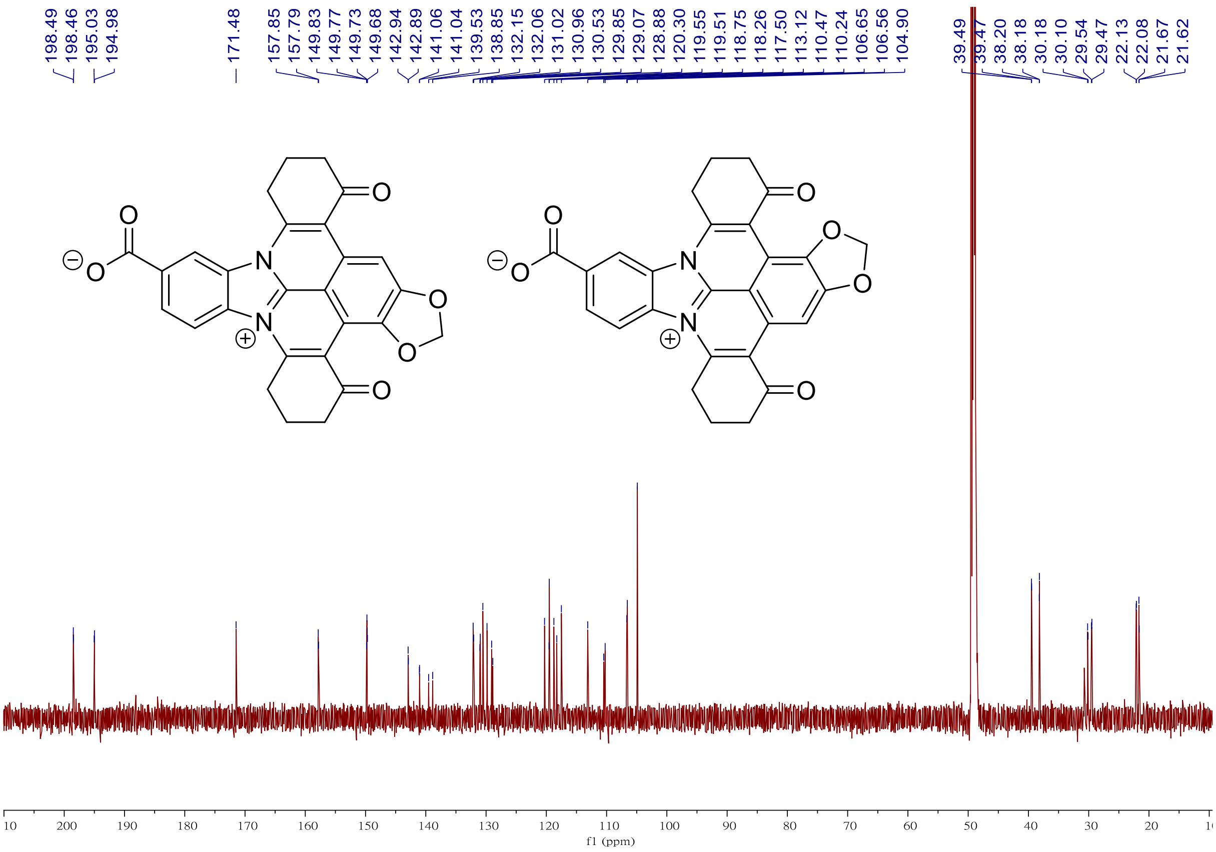

**Figure S280.** $^{13}C\{^{1}H\}$ NMR spectrum of compound **6q/6q'** (151 MHz, $CD_3OD$).

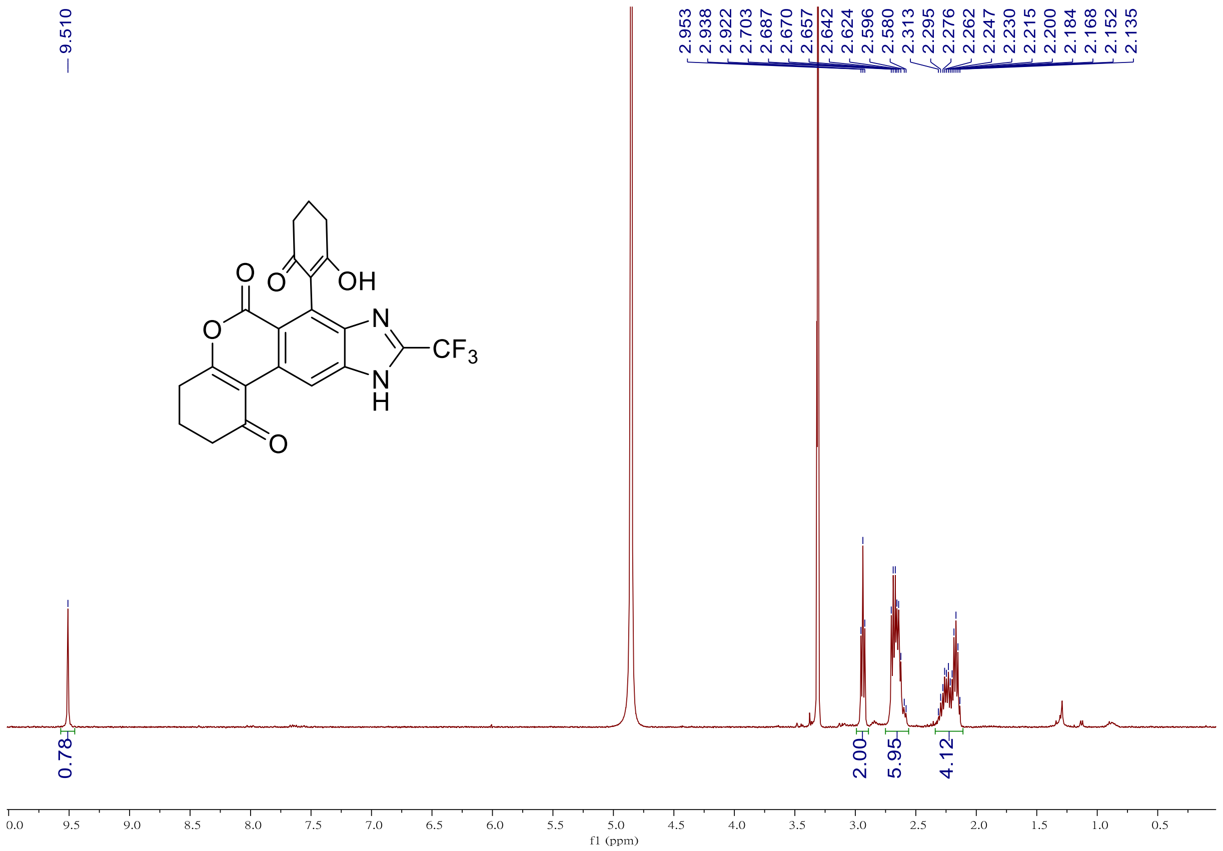


**Figure S281.** $^{1}$H NMR spectrum of compound **7f** (400 MHz, $CD_3OD$).

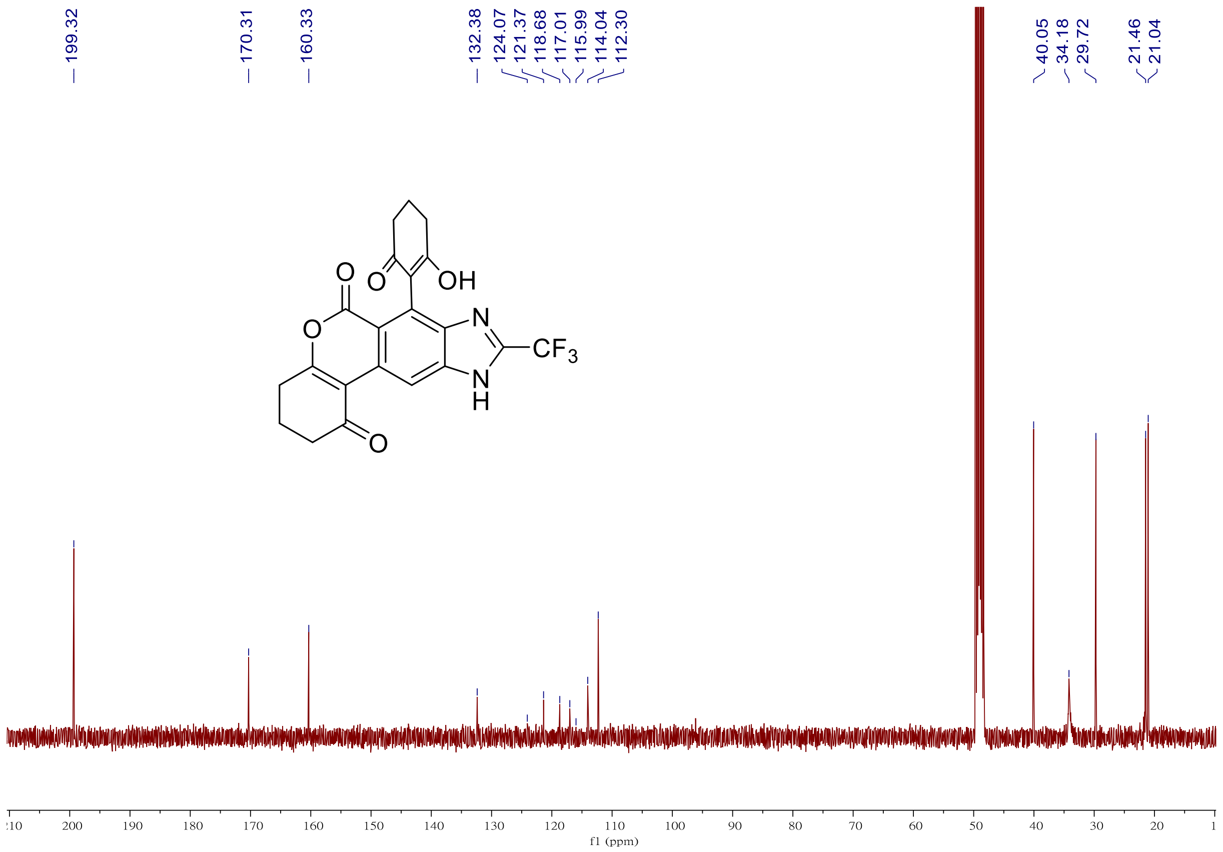


**Figure S282.** $^{13}C\{^{1}H\}$ NMR spectrum of compound **7f** (101 MHz, $CD_3OD$).

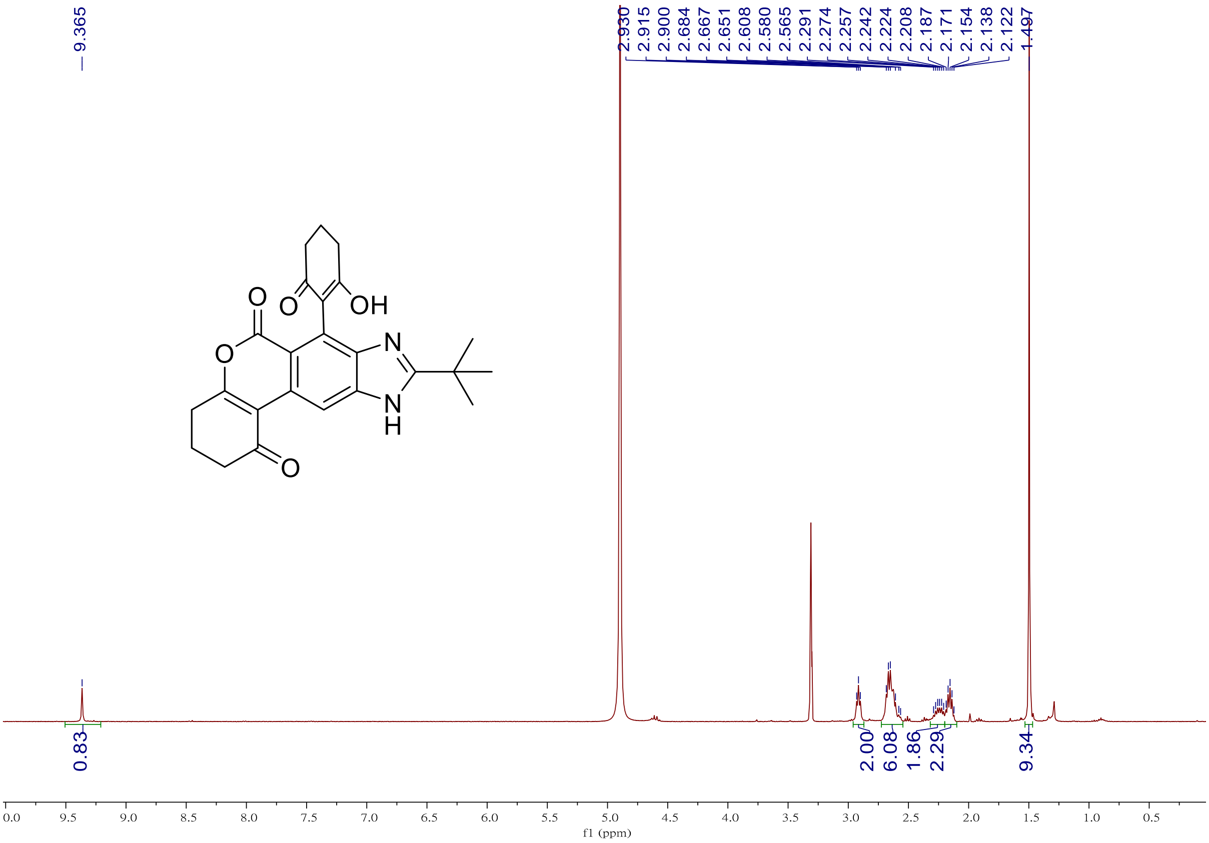

**Figure S283.** $^{1}H$ NMR spectrum of compound **7g** (400 MHz, $CD_3OD$).

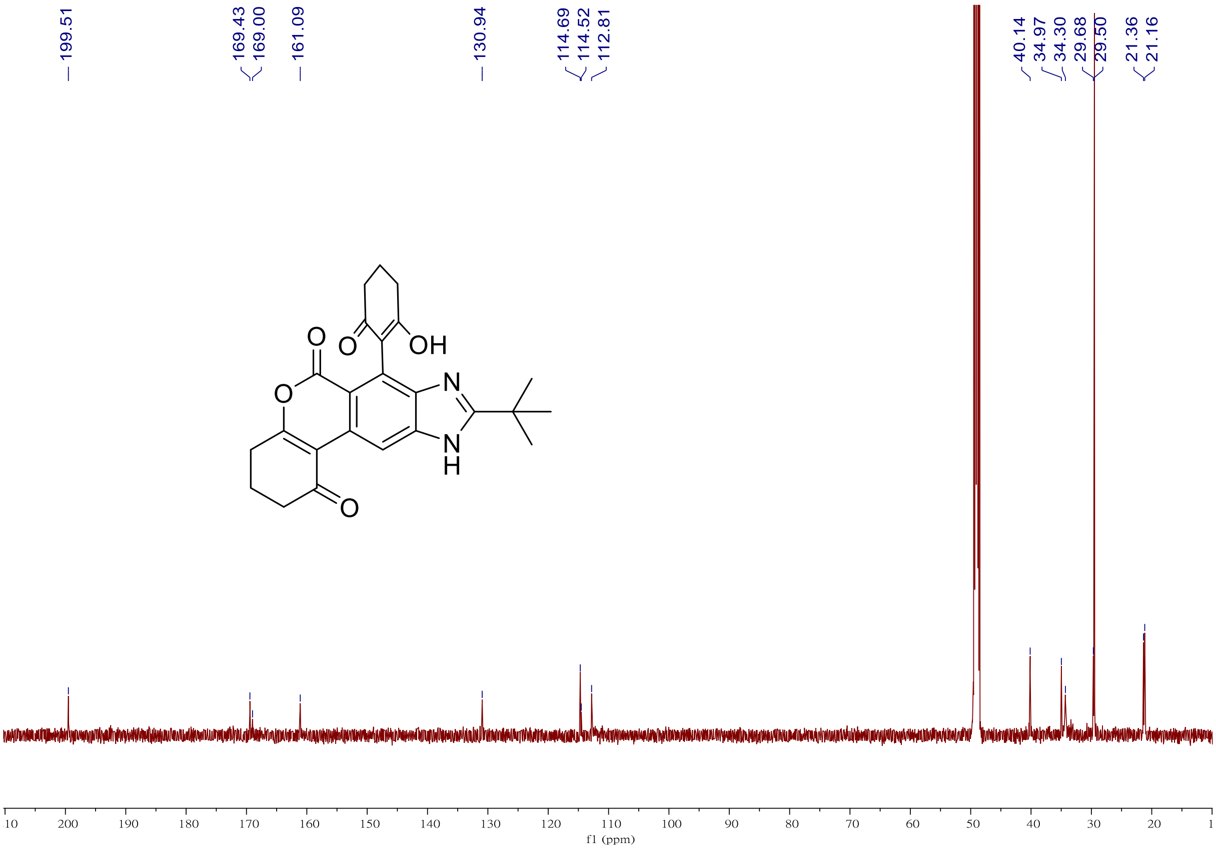

**Figure S284.** $^{13}C\{^{1}H\}$ NMR spectrum of compound **7g** (151 MHz, $CD_3OD$).

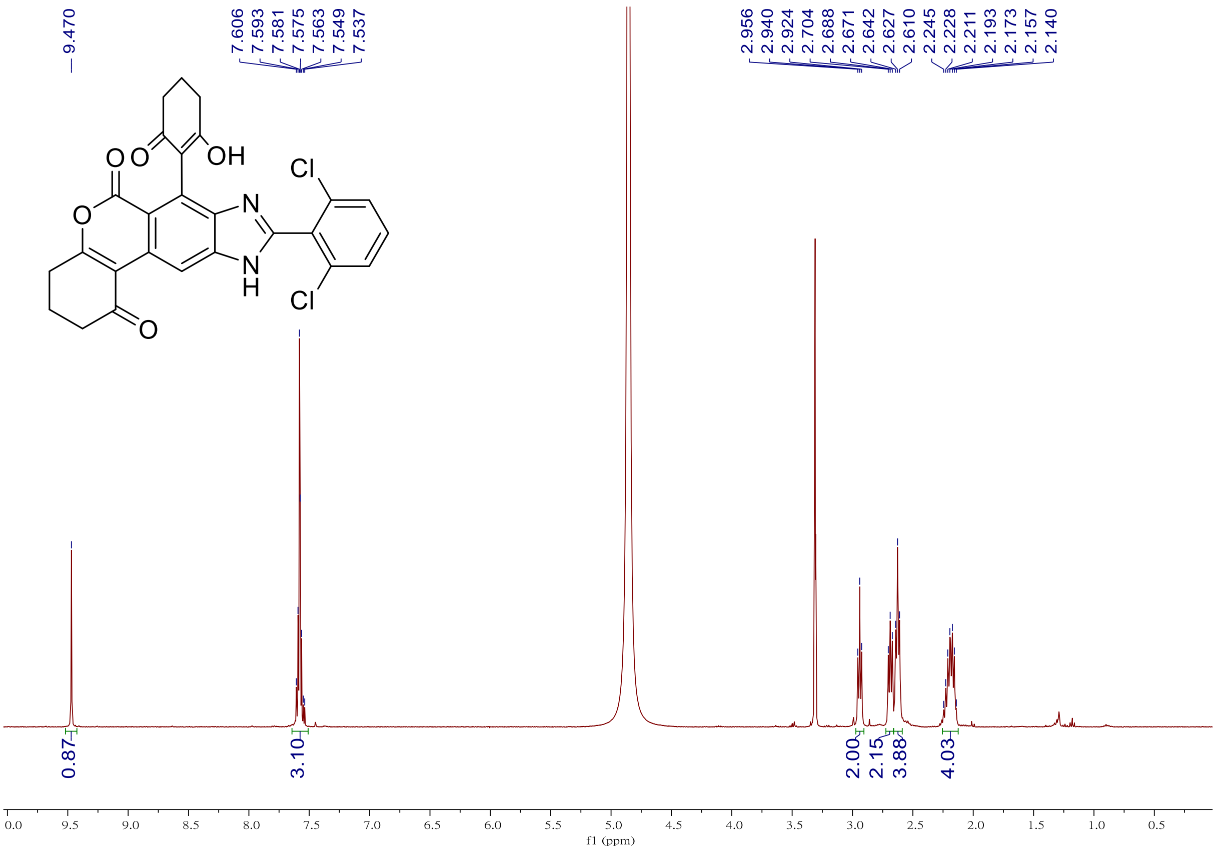


**Figure S285.** $^{1}H$ NMR spectrum of compound **7i** (400 MHz, $CD_3OD$).

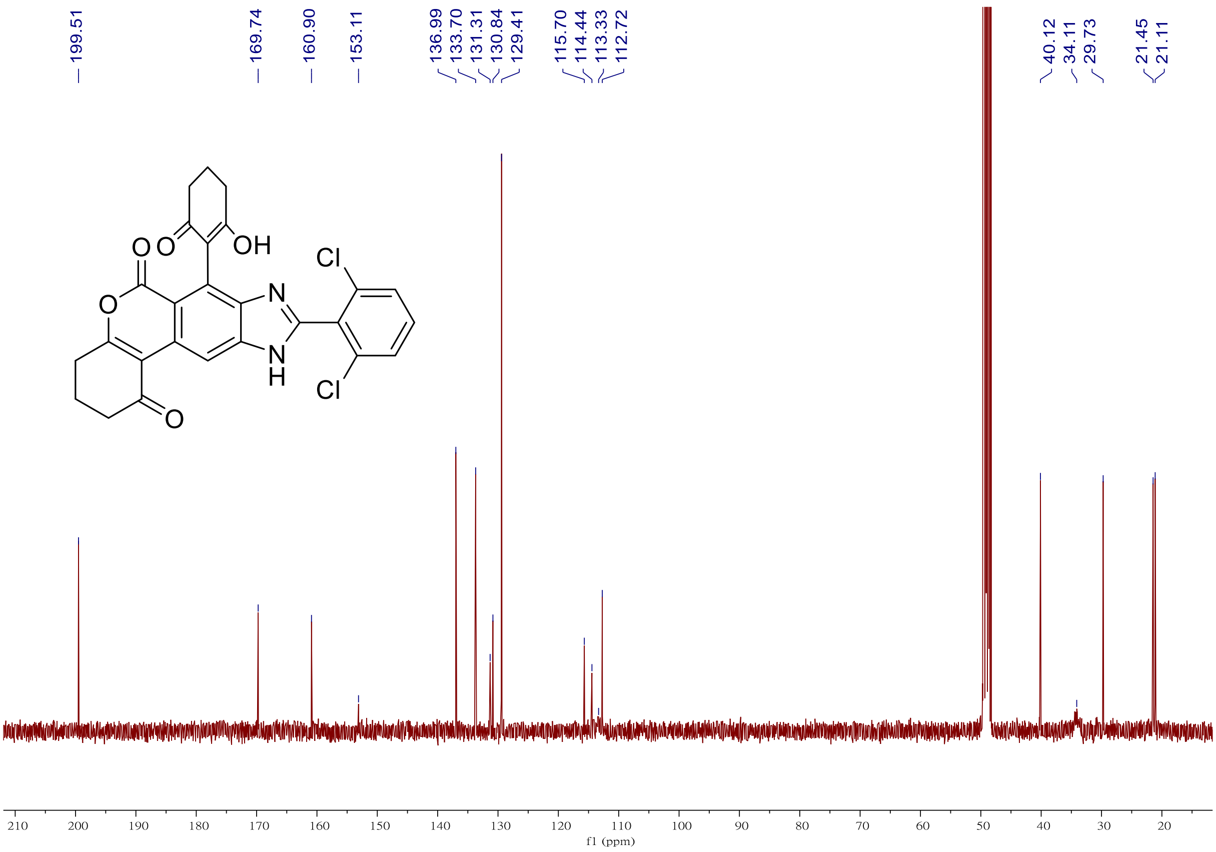


**Figure S286.** $^{13}C\{^{1}H\}$ NMR spectrum of compound **7i** (101 MHz, $CD_3OD$).

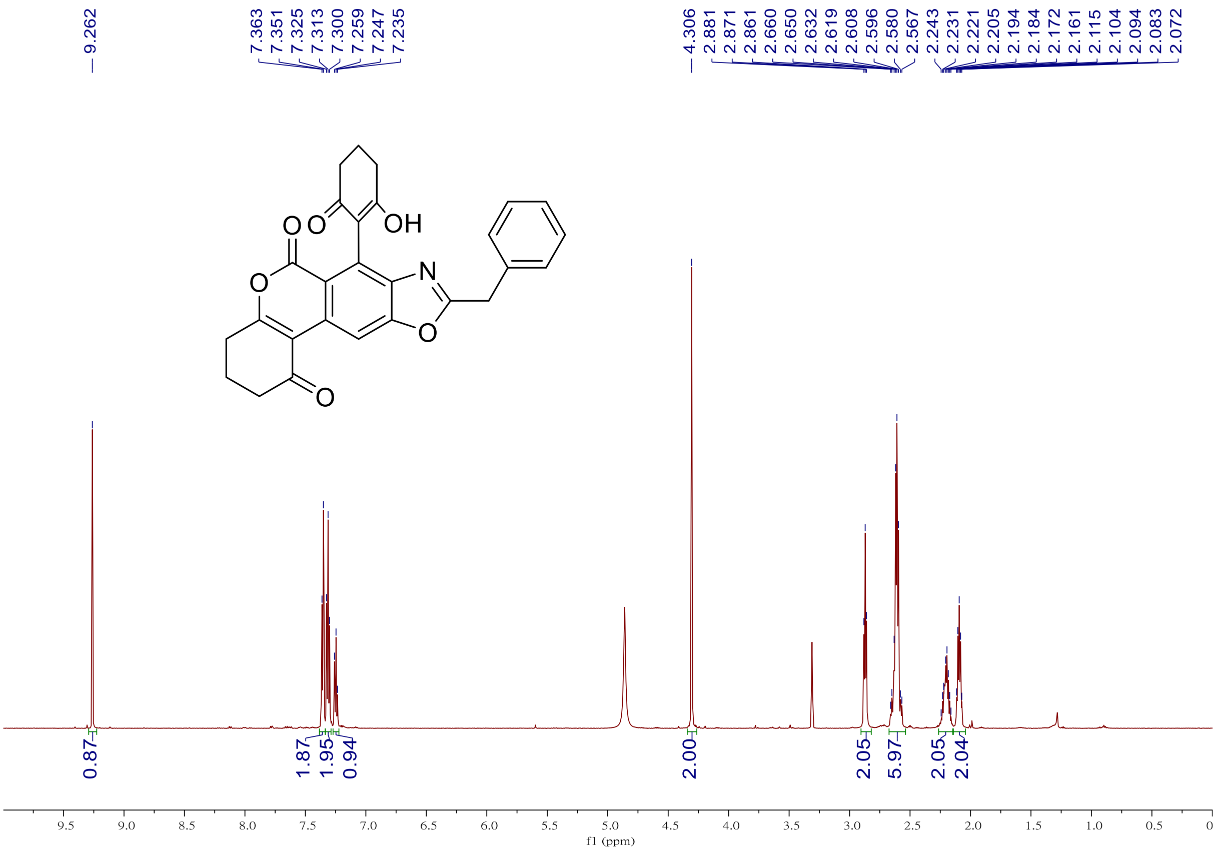


**Figure S287.** $^{1}$H NMR spectrum of compound **7x** (600 MHz, $CD_3OD$).

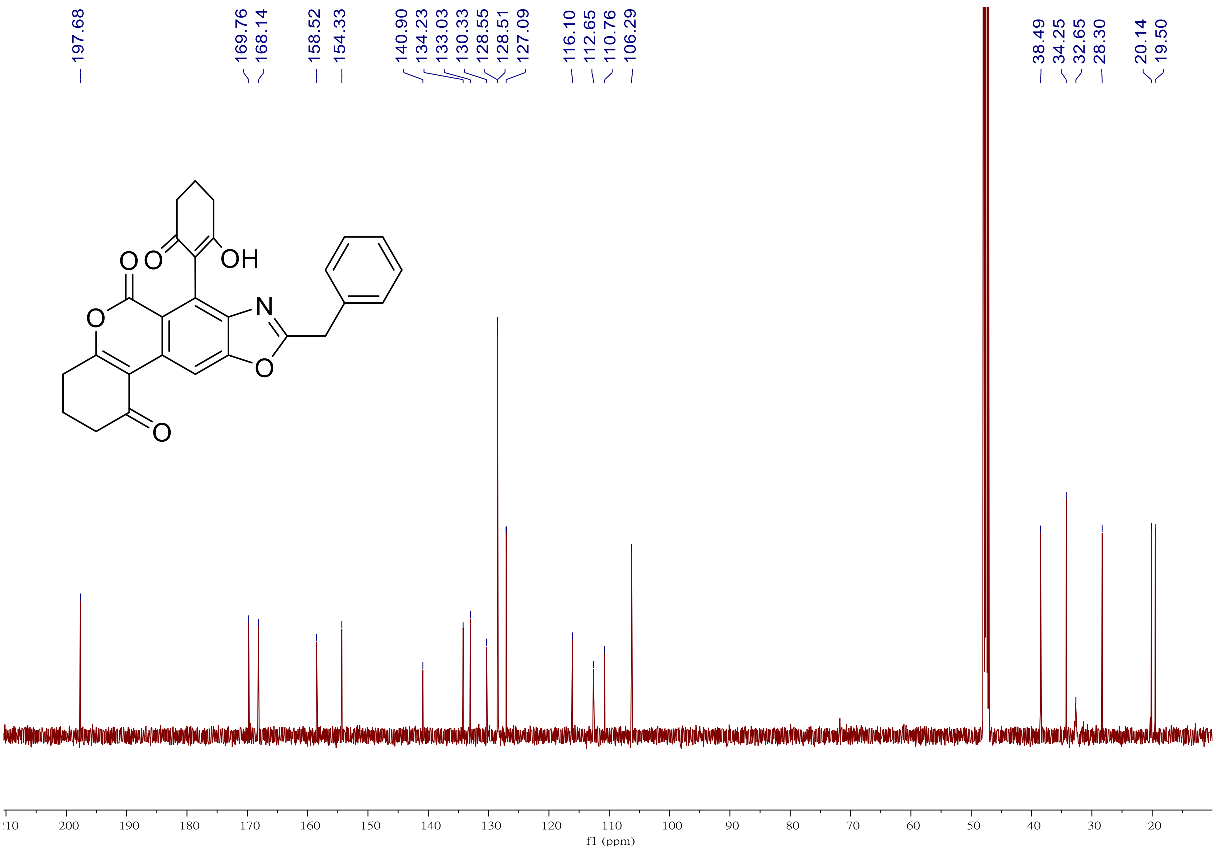


**Figure S288.** $^{13}C\{^{1}H\}$ NMR spectrum of compound **7x** (151 MHz, $CD_3OD$).

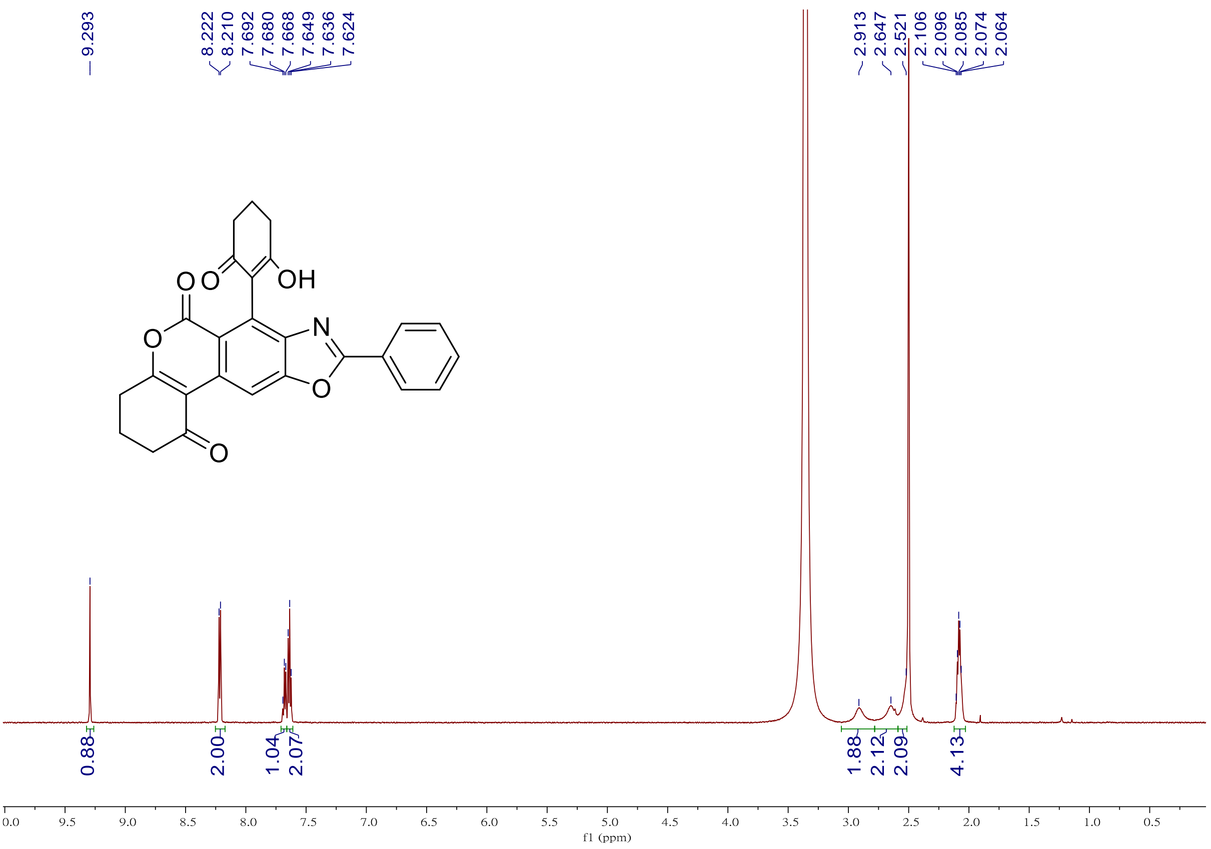

**Figure S289.** $^{1}H$ NMR spectrum of compound **7y** (600 MHz, $(CD_3)_2SO$).

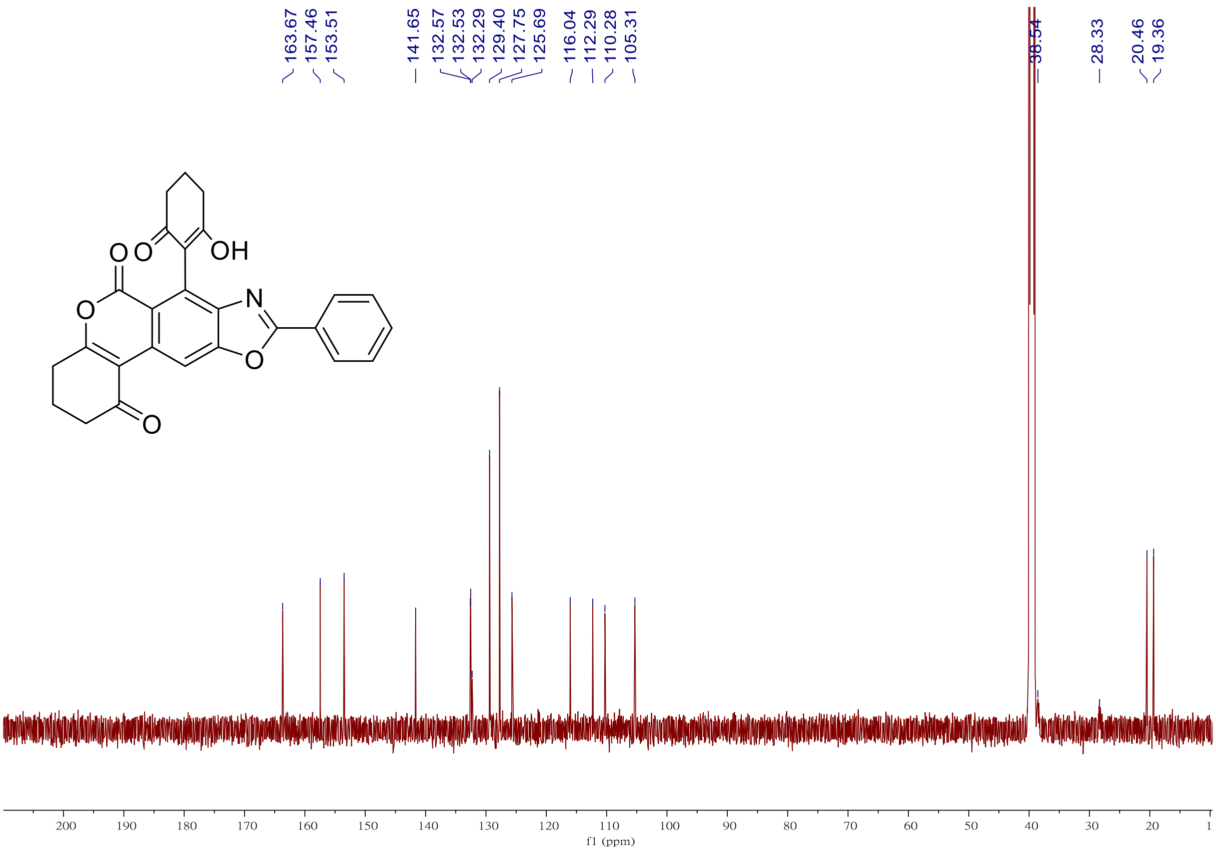

**Figure S290.** $^{13}C\{^{1}H\}$ NMR spectrum of compound **7y** (151 MHz, $(CD_3)_2SO$).

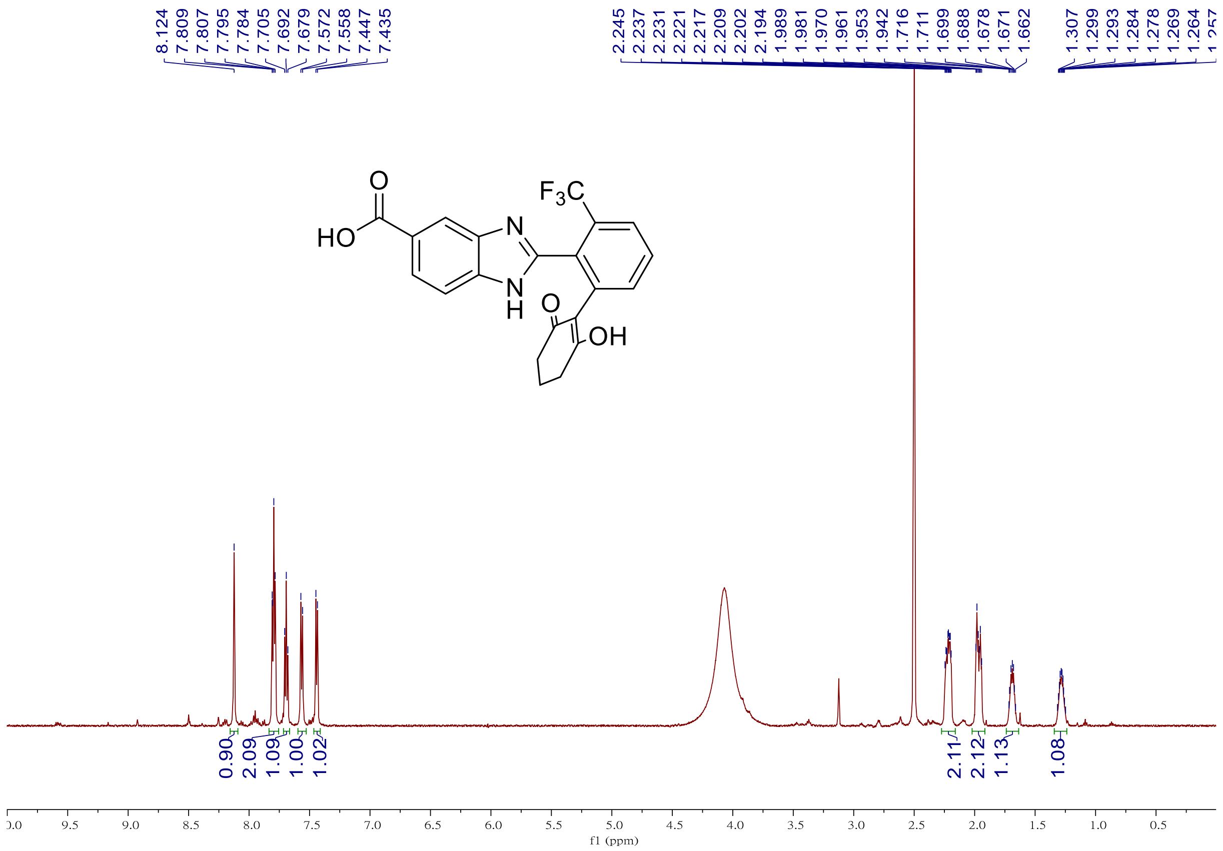

**Figure S291.** $^{1}$H NMR spectrum of compound **8h** (600 MHz, $(CD_3)_2SO$).

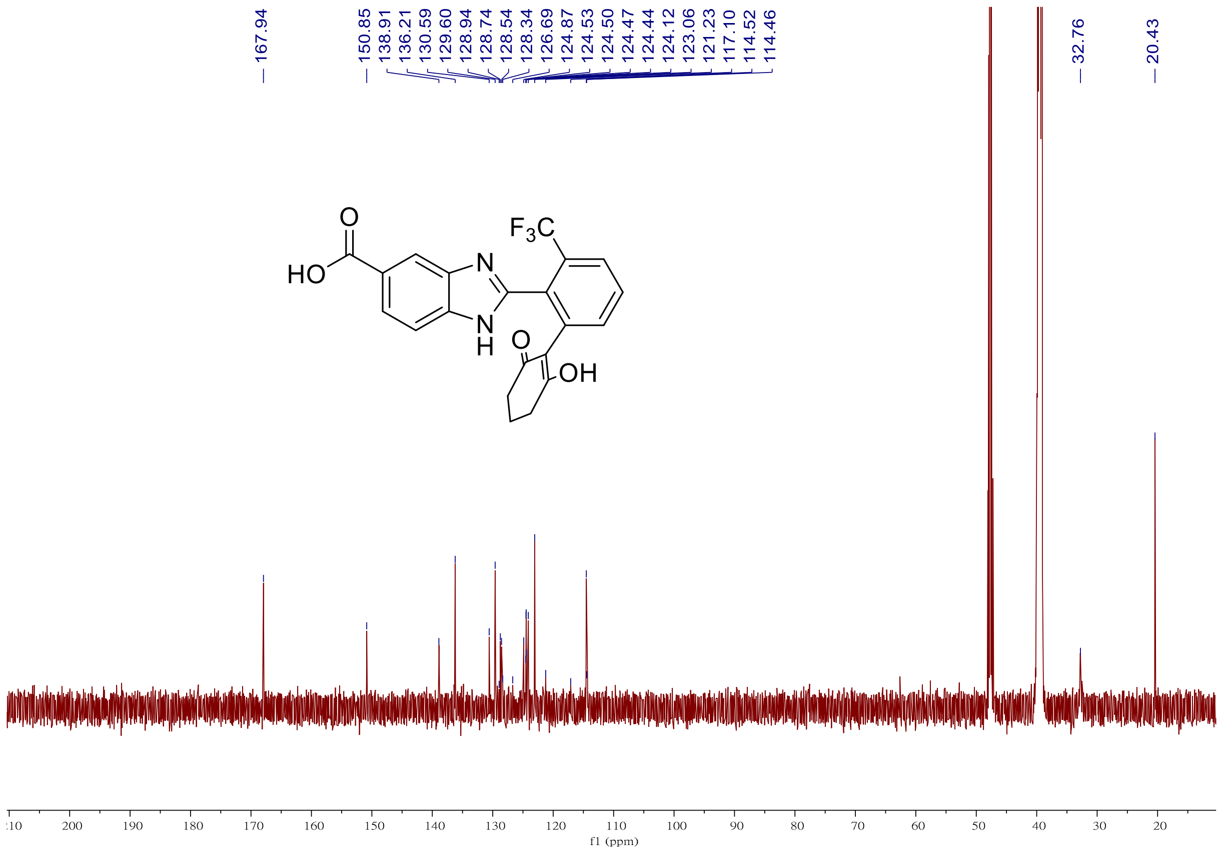

**Figure S292.** $^{13}C\{^{1}H\}$ NMR spectrum of compound **8h** (151 MHz, $(CD_3)_2SO$).

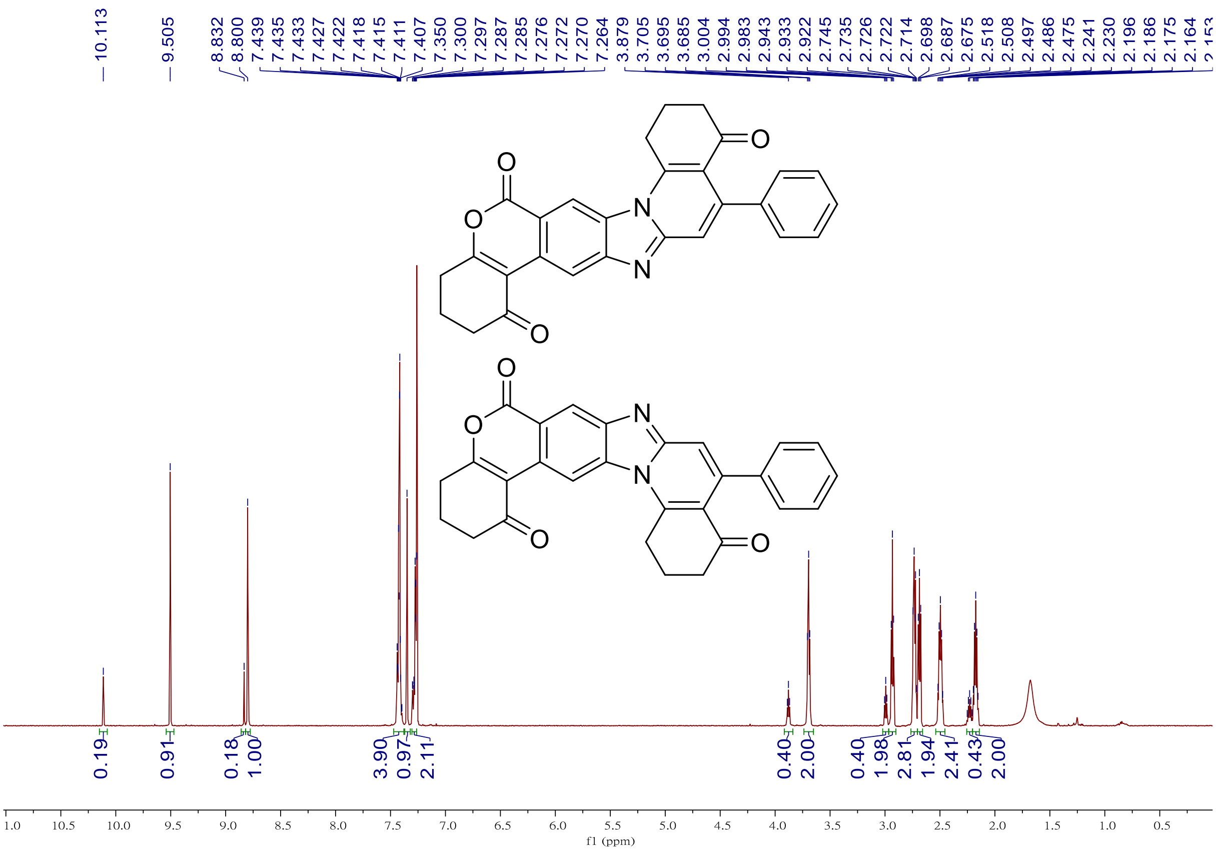


**Figure S293.** $^{1}$H NMR spectrum of compound **9r/9r'** (600 MHz, $CDCl_3$).

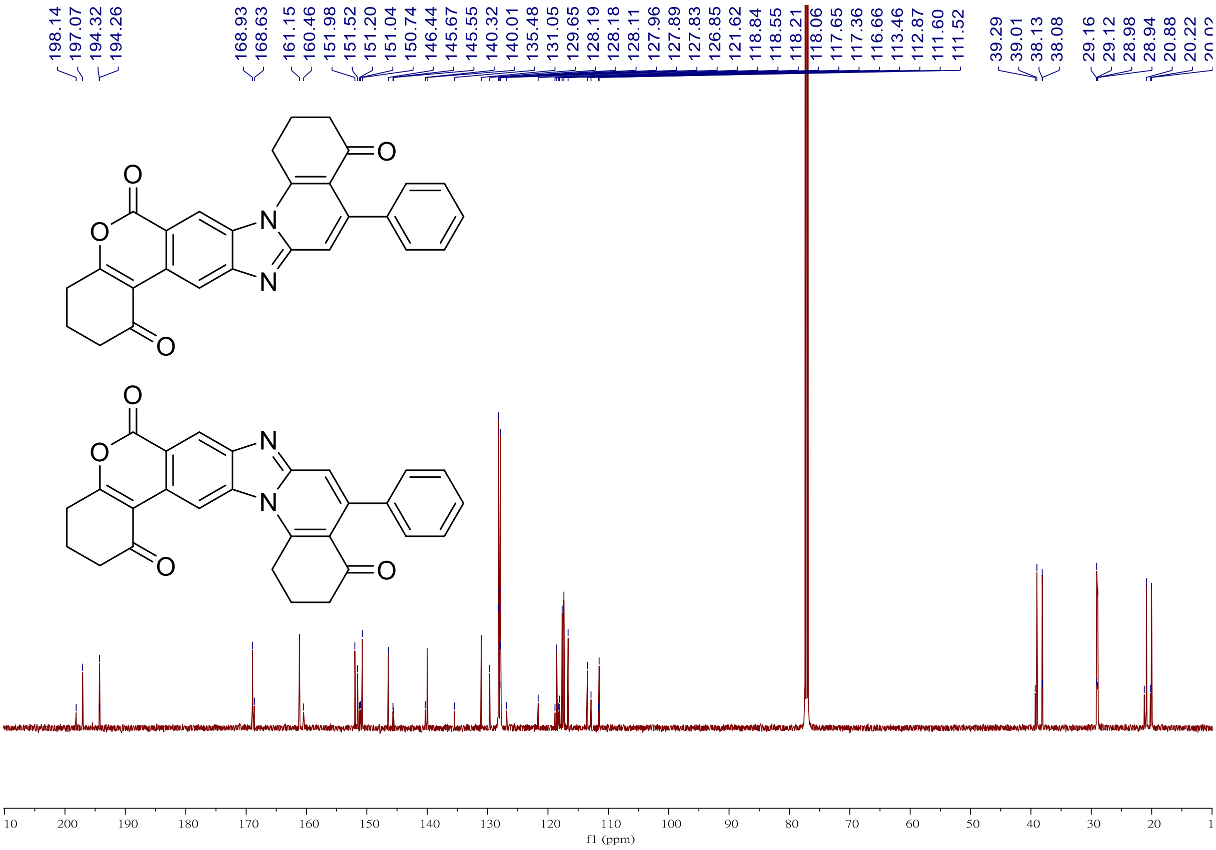


**Figure S294.** $^{13}C\{^{1}H\}$ NMR spectrum of compound **9r/9r'** (151 MHz, $CDCl_3$).

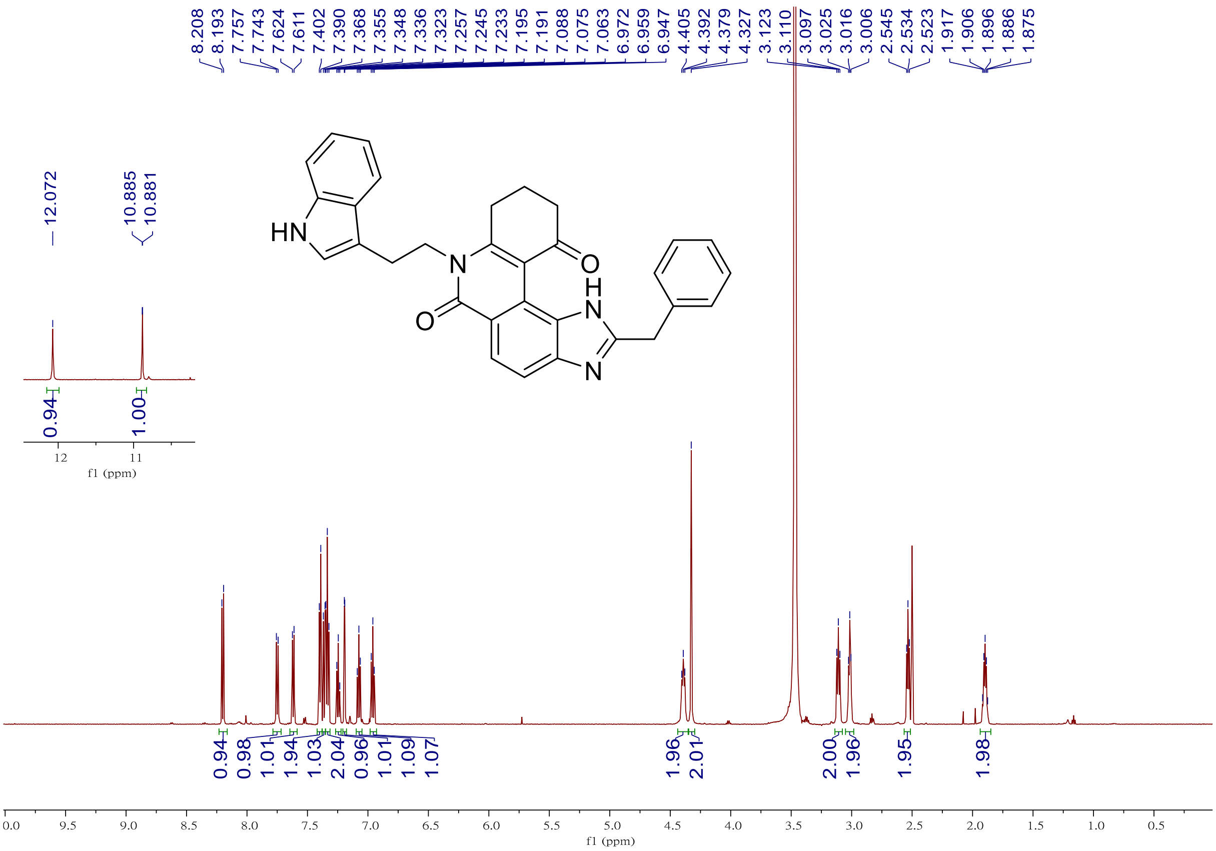

**Figure S295.** $^{1}$H NMR spectrum of compound **10** (600 MHz, $(CD_3)_2SO$).

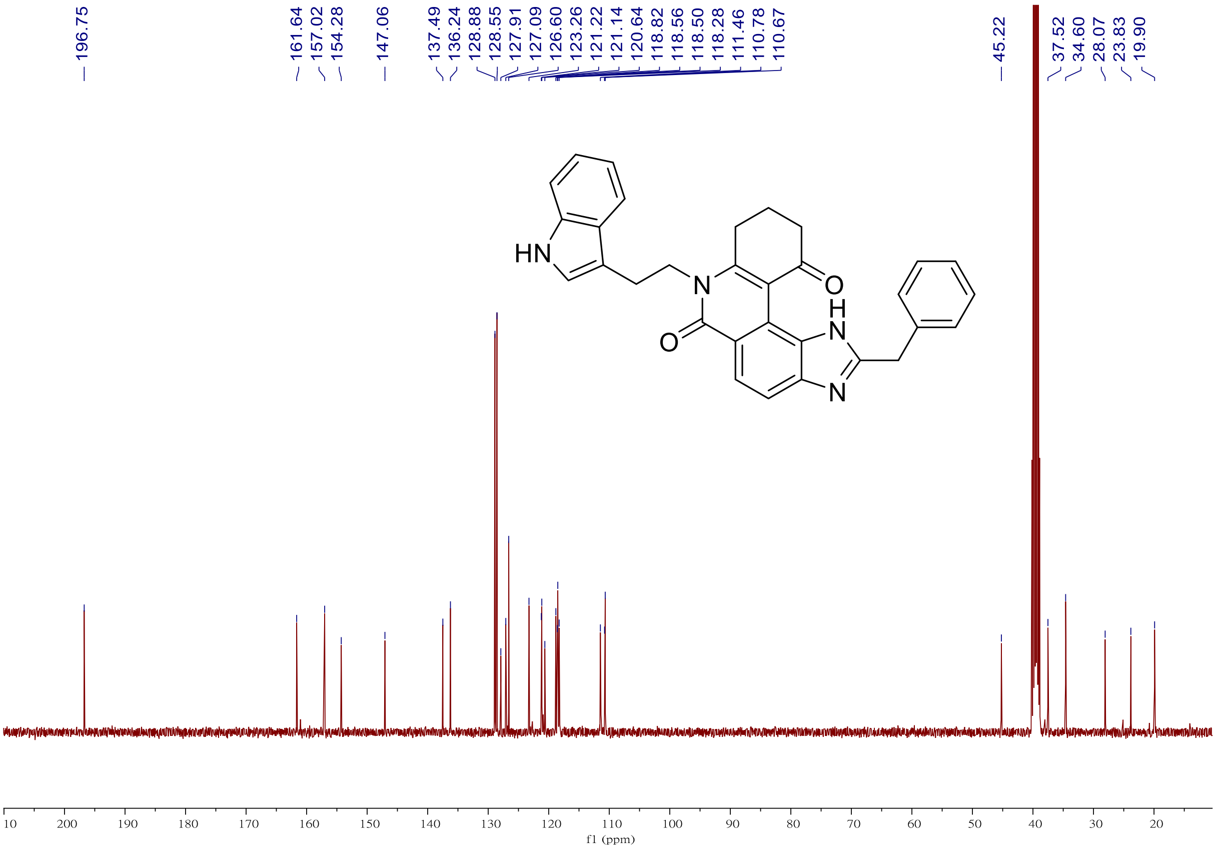

**Figure S296.** $^{13}C\{^{1}H\}$ NMR spectrum of compound **10** (151 MHz, $(CD_3)_2SO$).

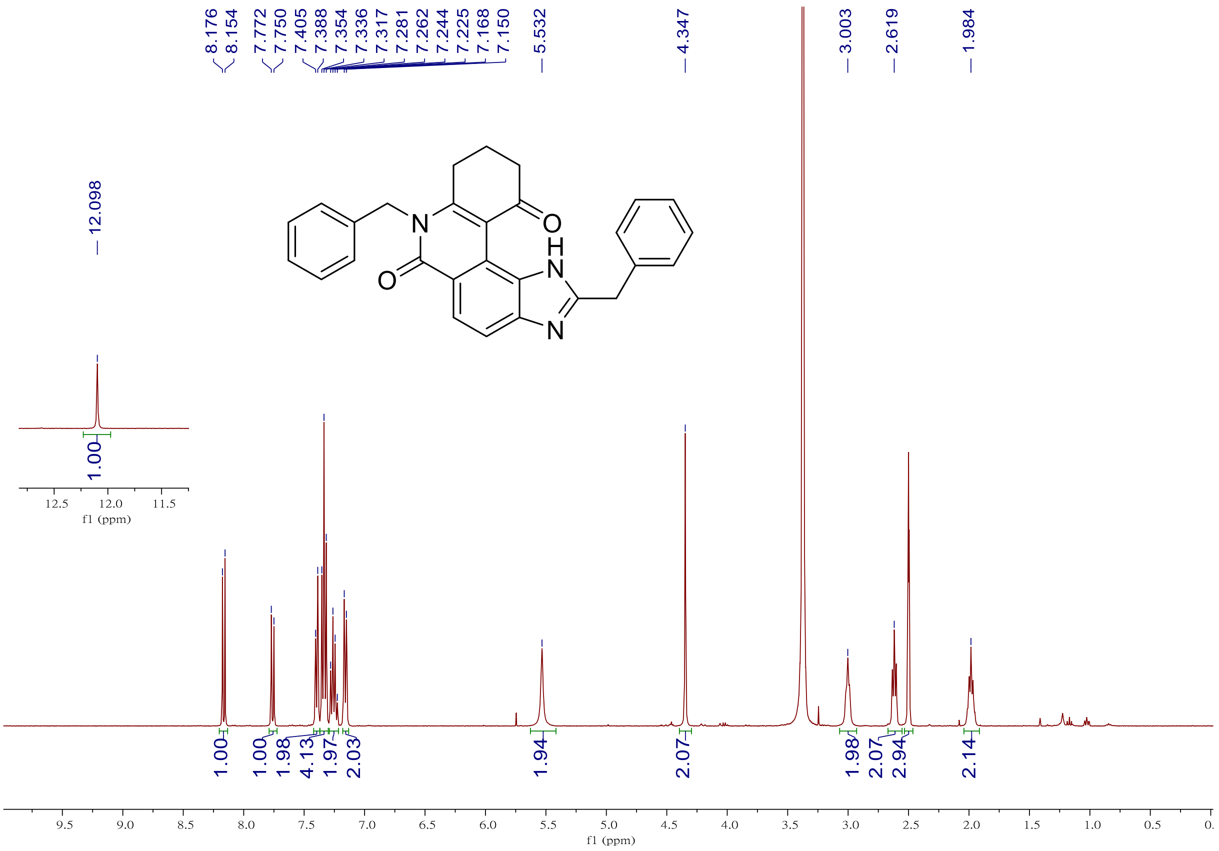


**Figure S297.** $^{1}H$ NMR spectrum of compound **11** (400 MHz, $(CD_3)_2SO$).

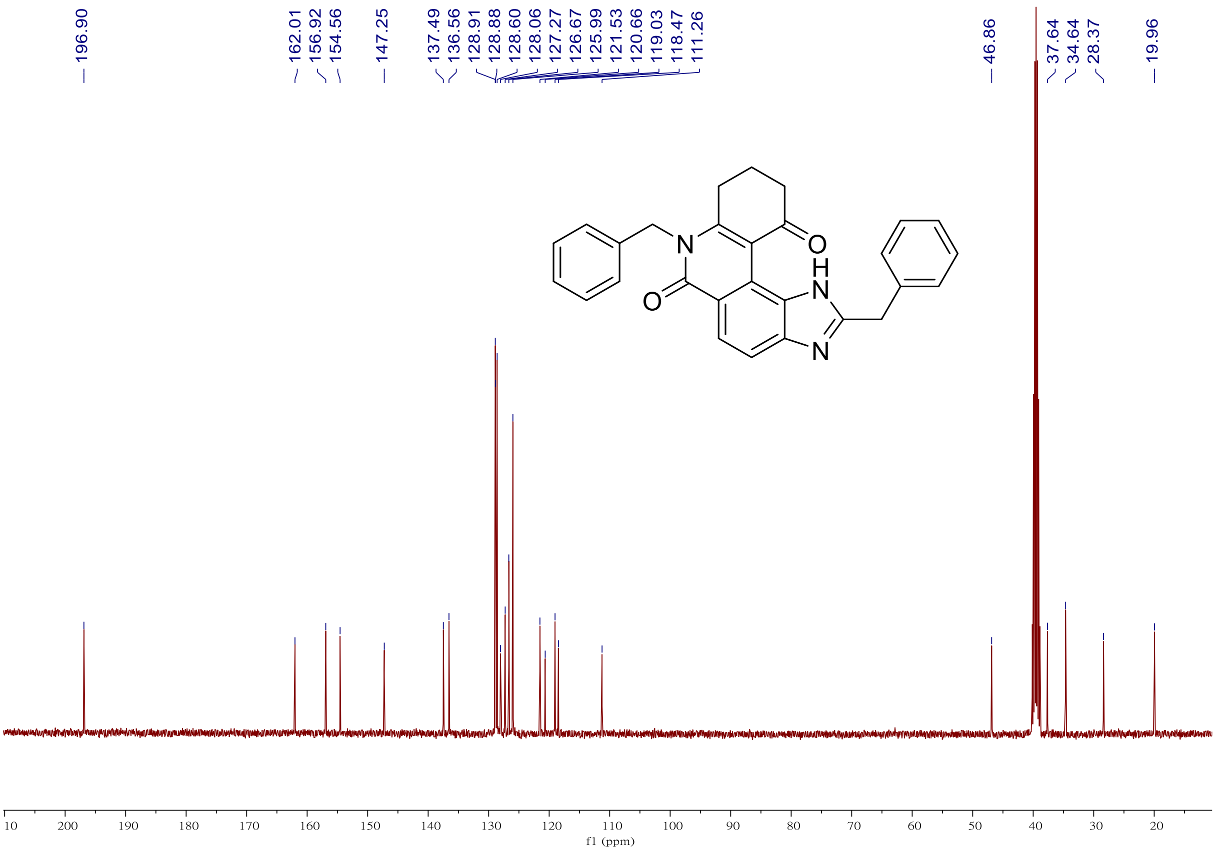


**Figure S298.** $^{13}C\{^{1}H\}$ NMR spectrum of compound **11** (101 MHz, $(CD_3)_2SO$).

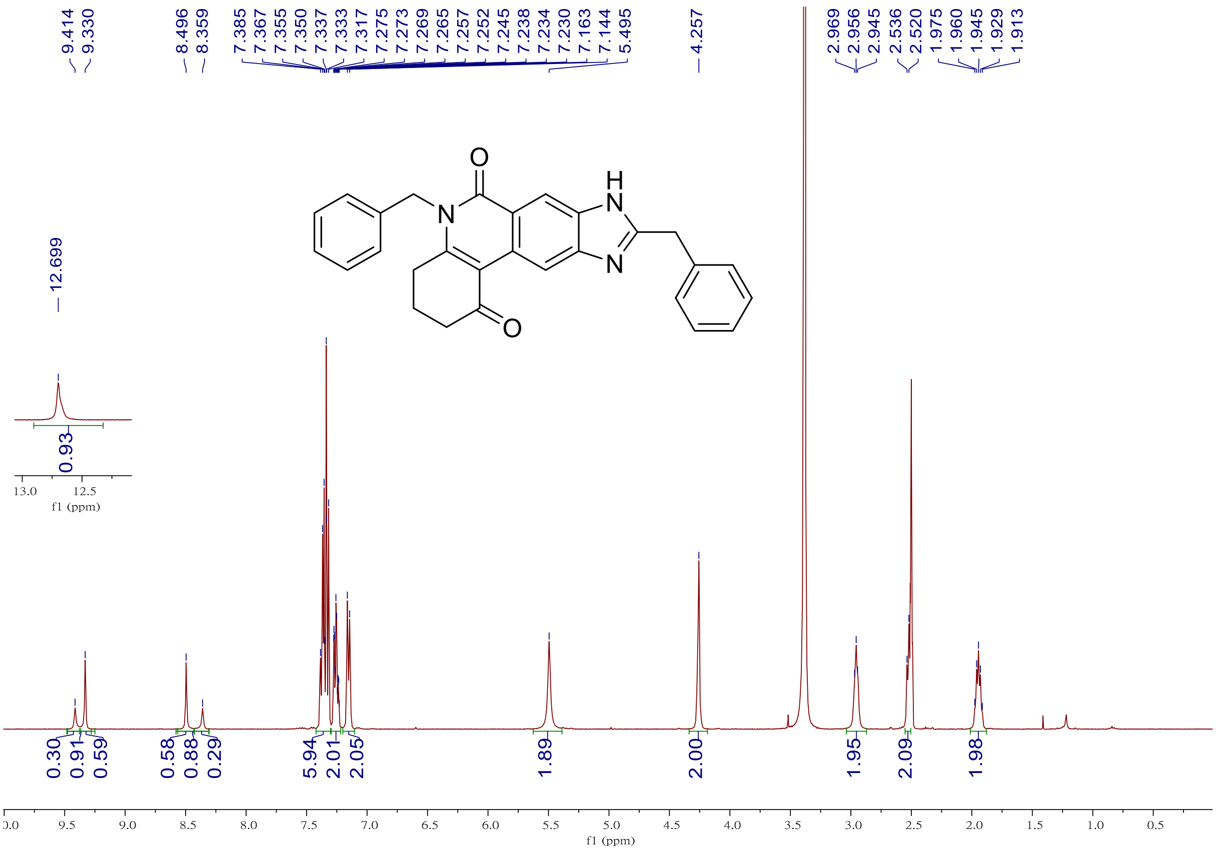


**Figure S299.** $^{1}$H NMR spectrum of compound **12** (400 MHz, $(CD_3)_2SO$).

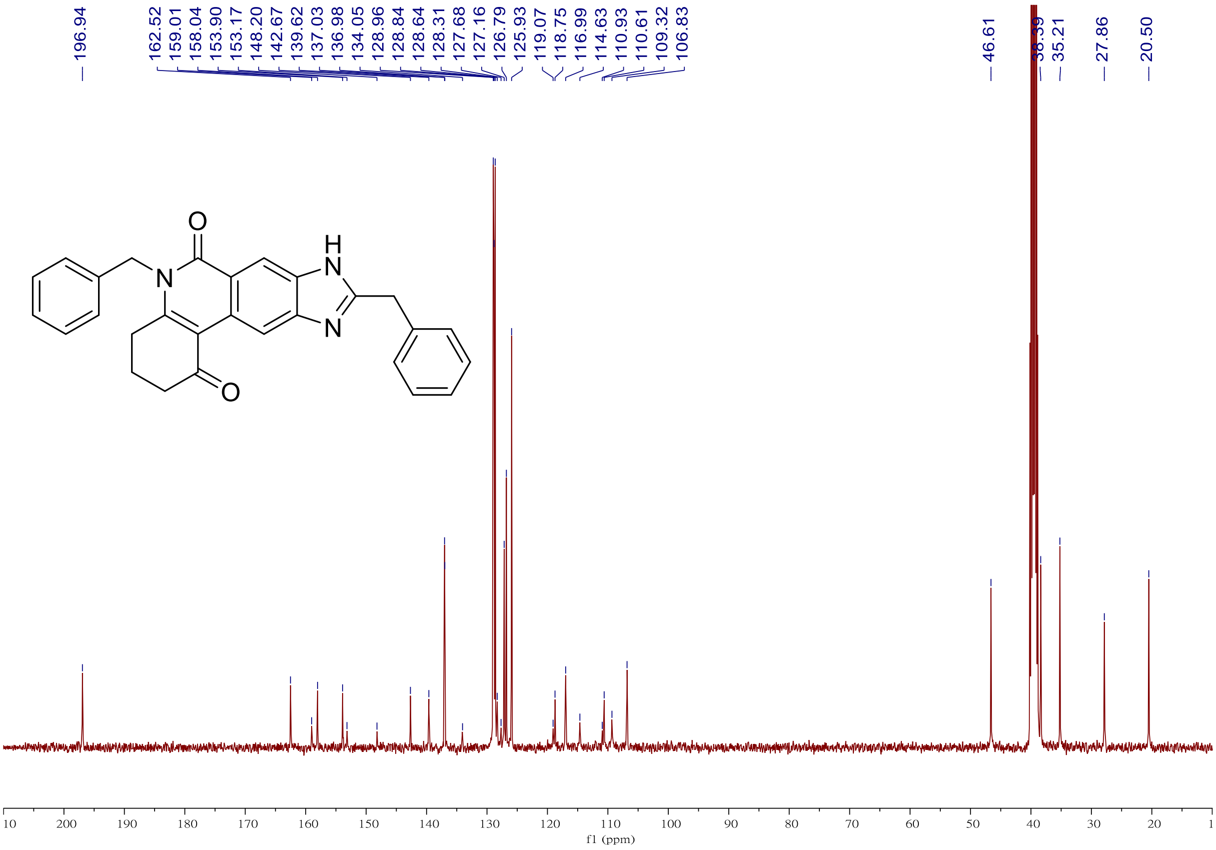


**Figure S300.** $^{13}$C{$^{1}$H} NMR spectrum of compound **12** (101 MHz, $(CD_3)_2SO$).

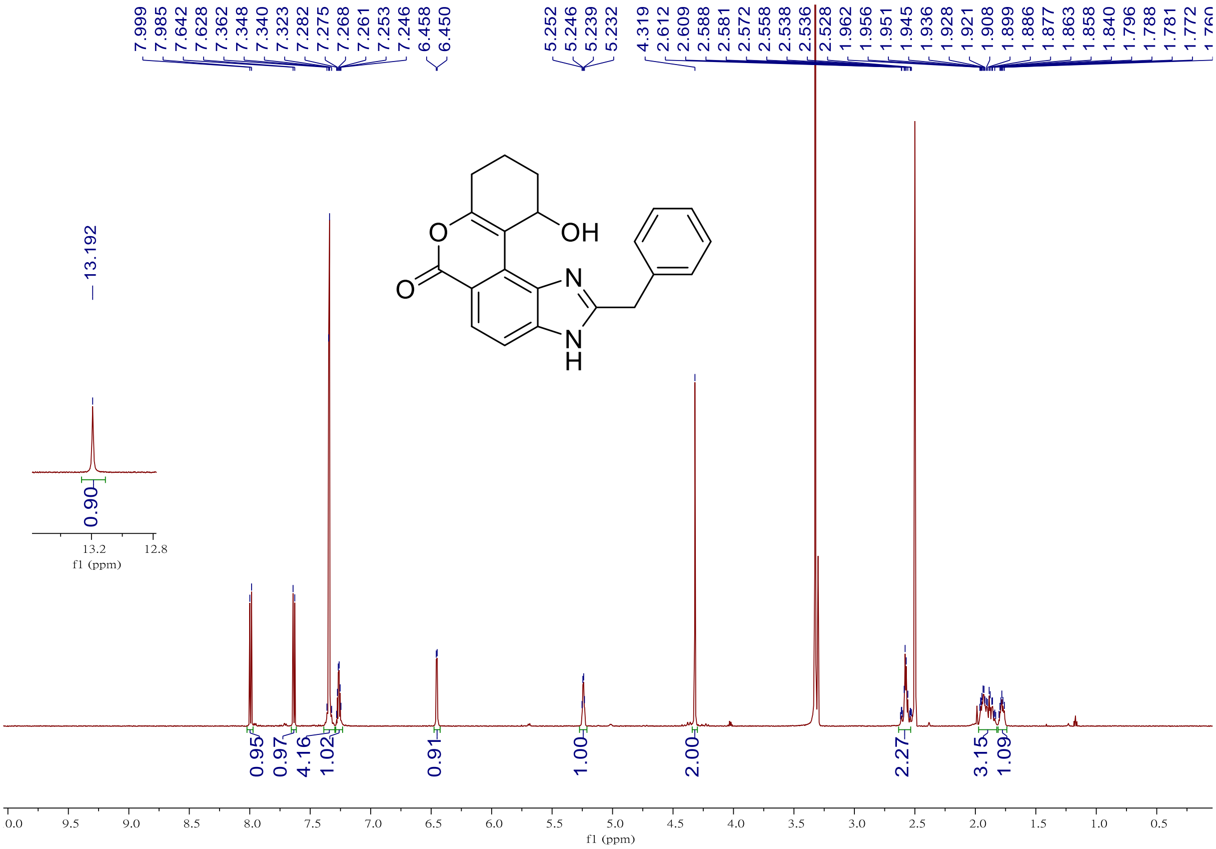


**Figure S301.** $^{1}$H NMR spectrum of compound **13** (600 MHz, $(CD_3)_2SO$).

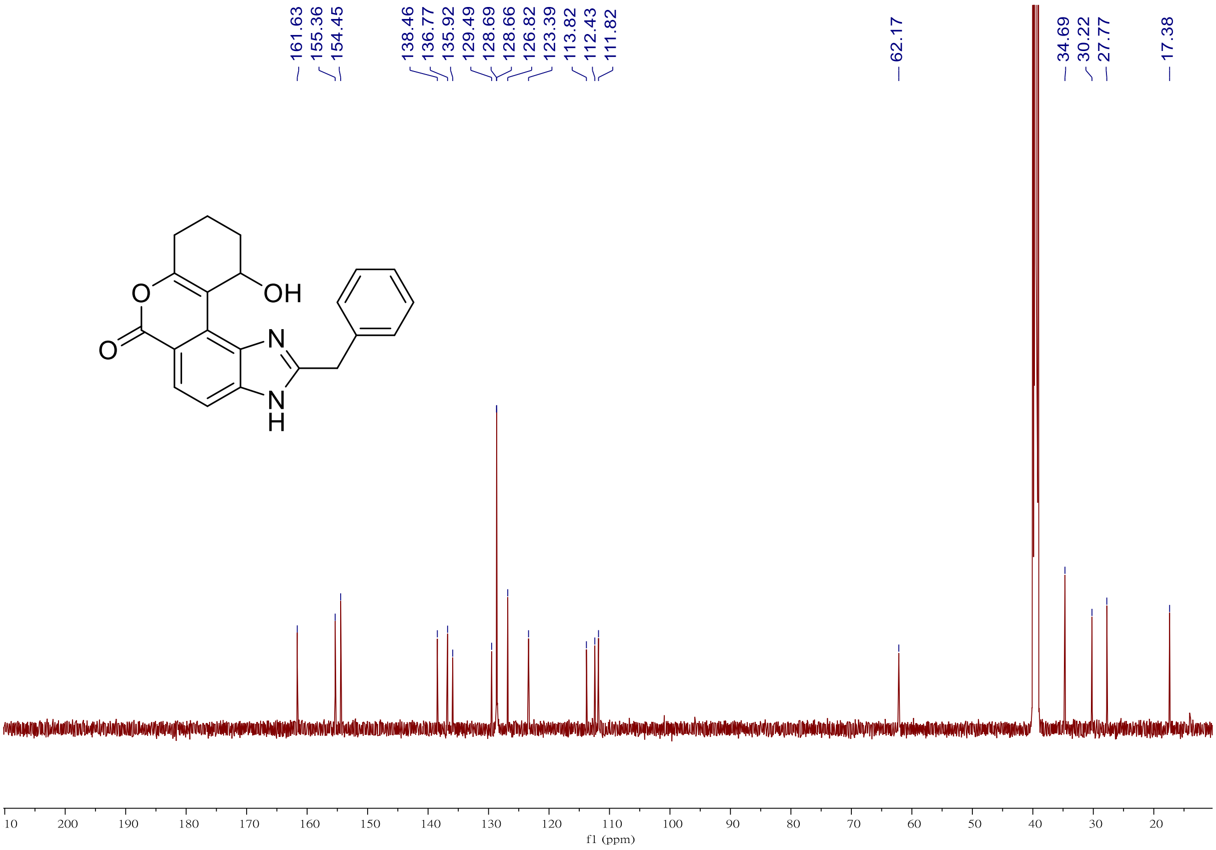


**Figure S302.** $^{13}C\{^{1}H\}$ NMR spectrum of compound **13** (151 MHz, $(CD_3)_2SO$).

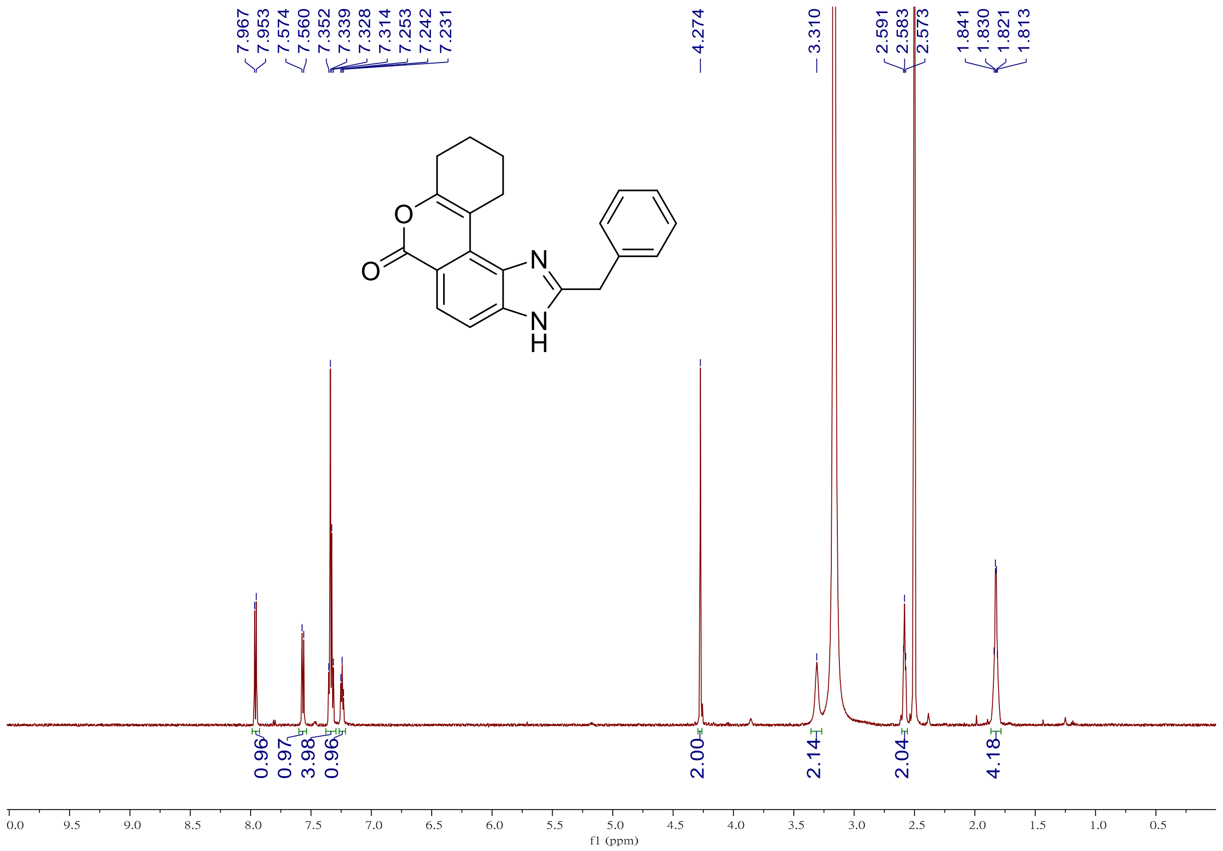


**Figure S303.** $^{1}H$ NMR spectrum of compound **14** (600 MHz, $(CD_3)_2SO$).

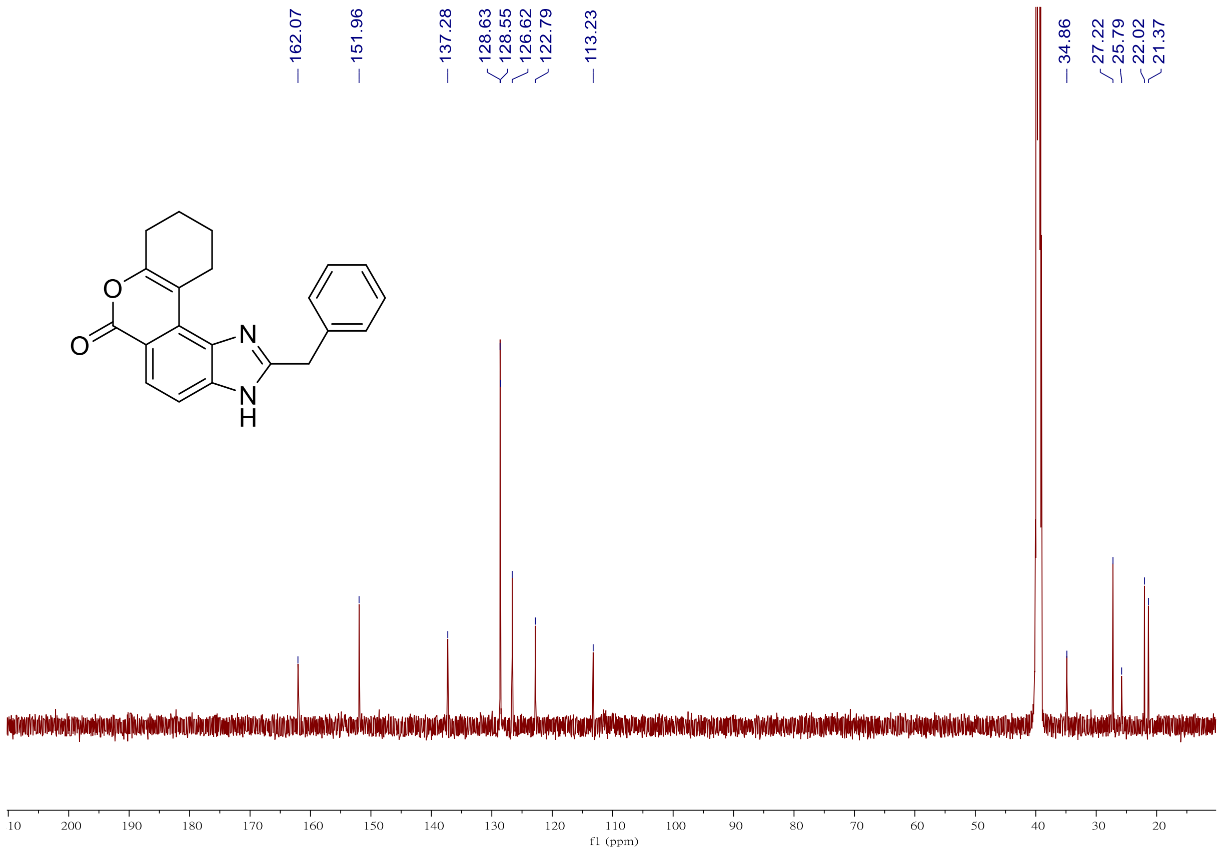


**Figure S304.** $^{13}C\{^{1}H\}$ NMR spectrum of compound **14** (151 MHz, $(CD_3)_2SO$).

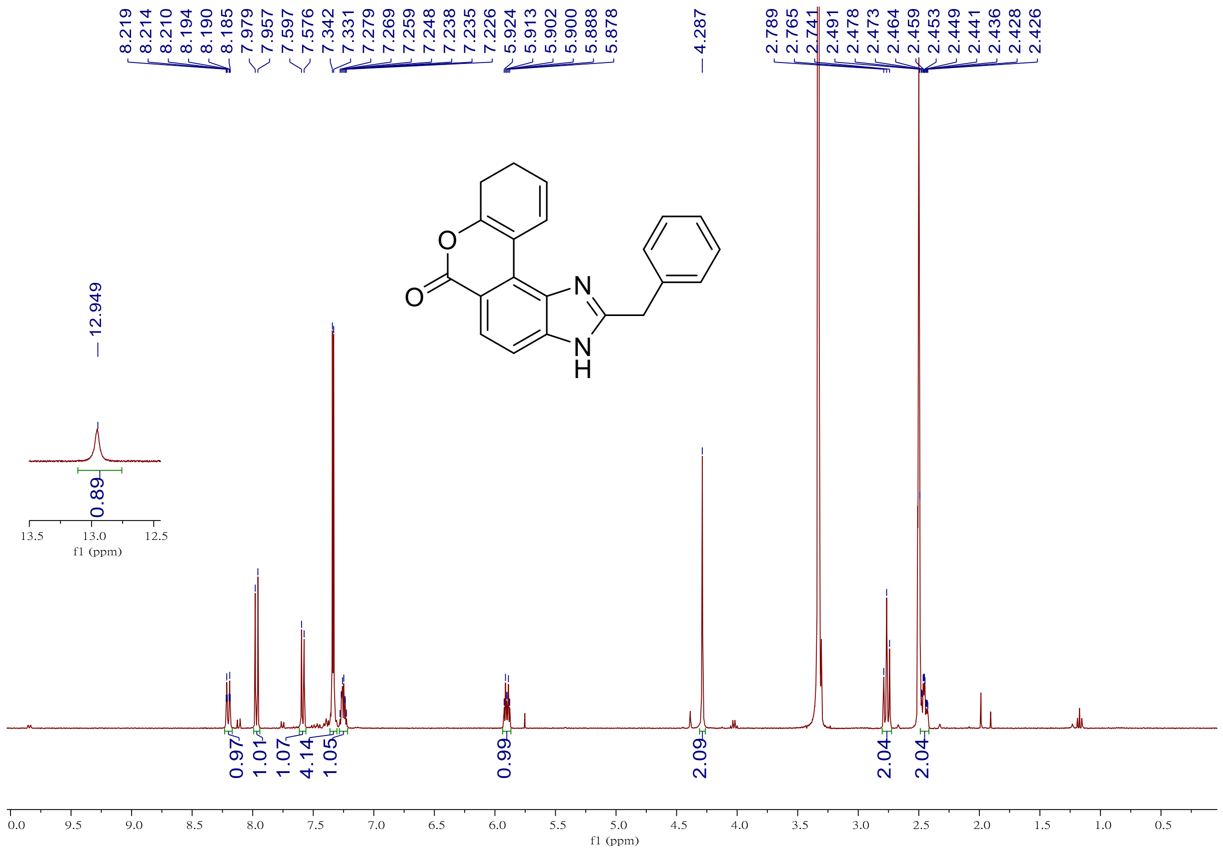

**Figure S305.** $^{1}H$ NMR spectrum of compound **15** (400 MHz, $(CD_3)_2SO$).

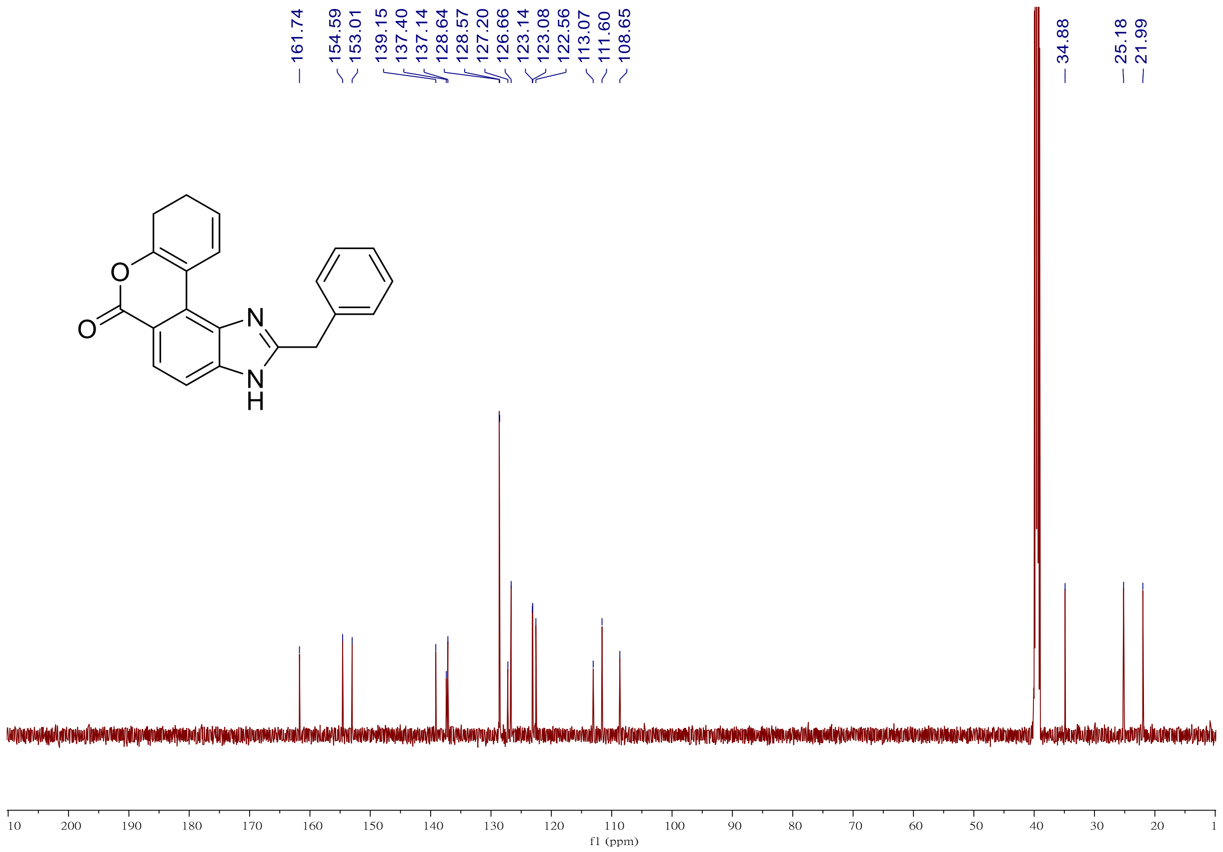

**Figure S306.** $^{13}C\{^{1}H\}$ NMR spectrum of compound **15** (151 MHz, $(CD_3)_2SO$).

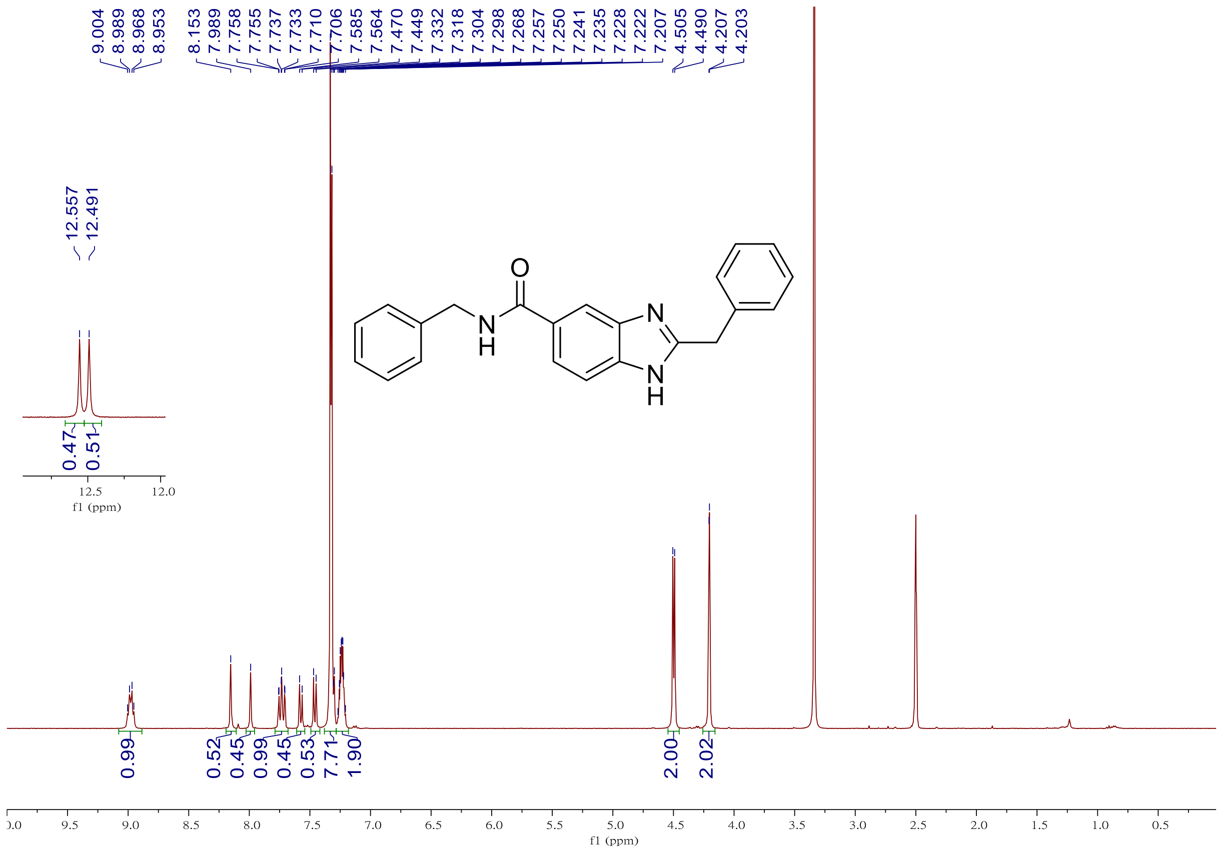


**Figure S307.** $^{1}$H NMR spectrum of compound **17** (400 MHz, $(CD_3)_2SO$).

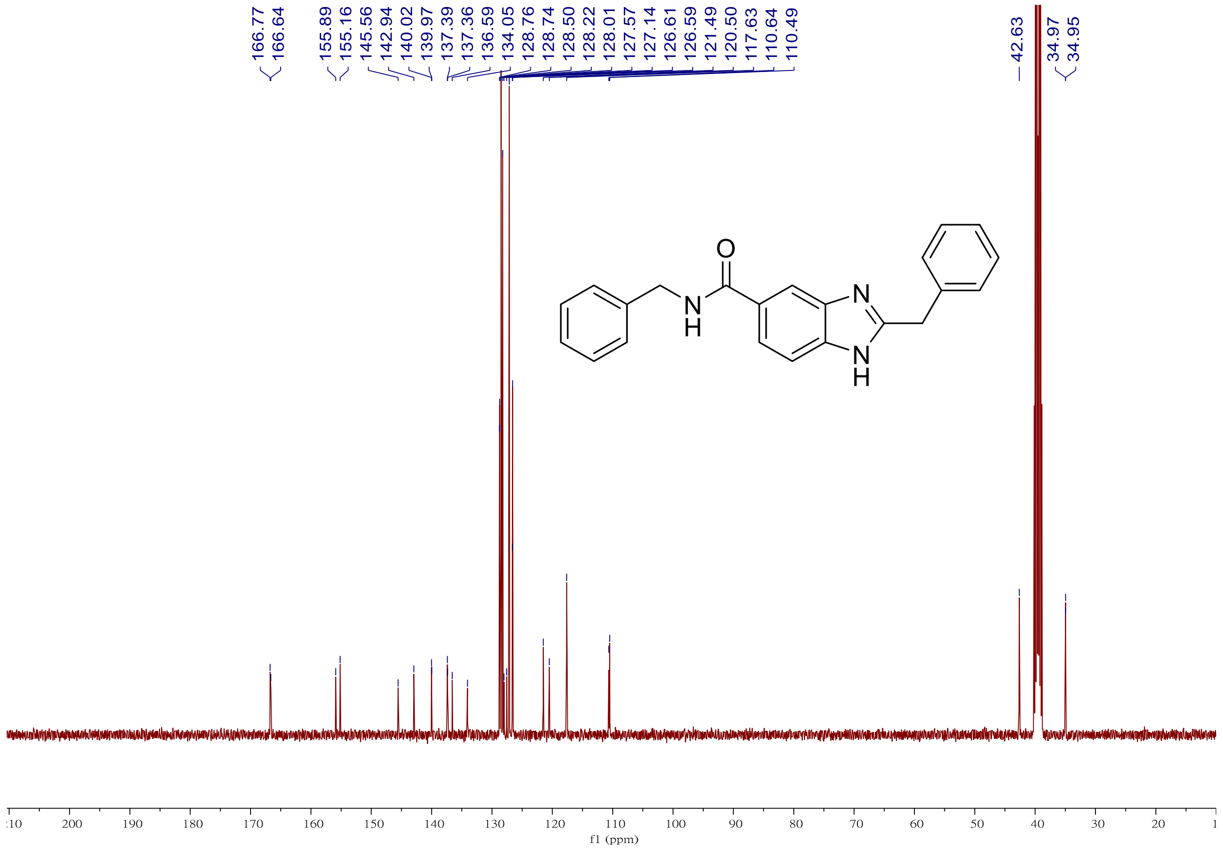


**Figure S308.** $^{13}C\{^{1}H\}$ NMR spectrum of compound **17** (101 MHz, $(CD_3)_2SO$).

## X-Ray Crystallography Data

**Table S6.** Crystal data and structure refinement for **3g.**

| | |
|---|---|
| Identification code | **3g** |
| Empirical formula | $C_{19}H_{19}Cl_3N_2O_3$ |
| Formula weight | 429.71 |
| Temperature/K | 99.98(10) |
| Crystal system | triclinic |
| Space group | P-1 |
| a/Å | 6.92620(10) |
| b/Å | 9.4138(2) |
| c/Å | 14.8179(3) |
| α/° | 88.451(2) |
| β/° | 87.494(2) |
| γ/° | 78.119(2) |
| Volume/Å$^3$ | 944.39(3) |
| Z | 2 |
| $\rho_{calc}$g/cm$^3$ | 1.511 |
| μ/mm$^{-1}$ | 4.595 |
| F(000) | 444.0 |
| Crystal size/mm$^3$ | 0.13 × 0.08 × 0.03 |
| Radiation | Cu Kα (λ = 1.54184) |
| 2Θ range for data collection/° | 9.602 to 148.248 |
| Index ranges | $-8 \leq h \leq 7$, $-11 \leq k \leq 11$, $-17 \leq l \leq 18$ |
| Reflections collected | 12026 |
| Independent reflections | 3635 [$R_{int}$ = 0.0219, $R_{sigma}$ = 0.0238] |
| Data/restraints/parameters | 3635/0/252 |
| Goodness-of-fit on F$^2$ | 1.072 |
| Final R indexes [I>=2σ (I)] | $R_1$ = 0.0281, w$R_2$ = 0.0746 |
| Final R indexes [all data] | $R_1$ = 0.0300, w$R_2$ = 0.0756 |
| Largest diff. peak/hole / e Å$^{-3}$ | 0.35/-0.28 |

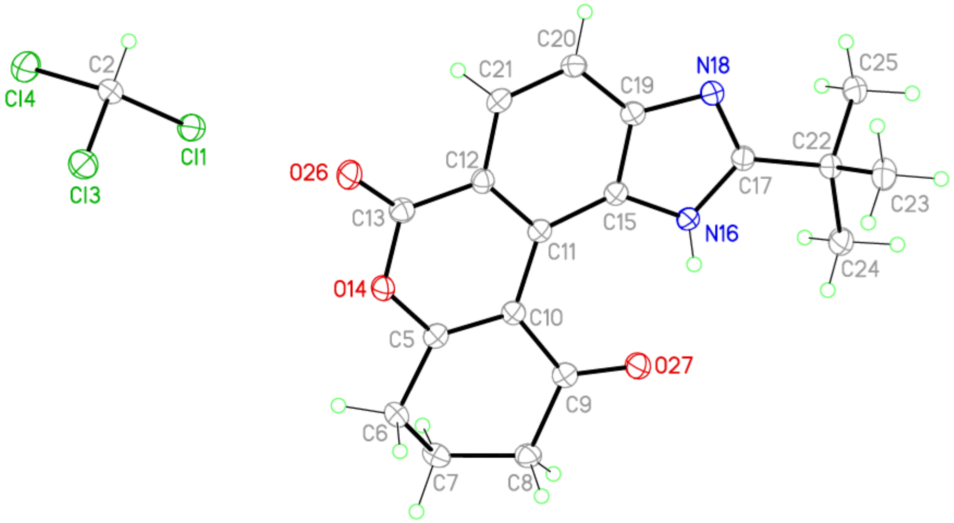


**Figure S309.** Molecular structure of **3g**, with the atomic displacement plot shown at 50% probability.

**Table S7.** Crystal data and structure refinement for **4a.**

| | |
|---|---|
| Identification code | **4a** |
| Empirical formula | $C_{21}H_{16}N_2O_3$ |
| Formula weight | 344.36 |
| Temperature/K | 100.01(10) |
| Crystal system | triclinic |
| Space group | P-1 |
| a/Å | 7.2393(3) |
| b/Å | 8.9128(2) |
| c/Å | 13.4483(5) |
| α/° | 75.201(3) |
| β/° | 76.743(3) |
| γ/° | 84.939(2) |
| Volume/Å$^3$ | 816.18(5) |
| Z | 2 |
| $\rho_{calc}$g/cm$^3$ | 1.401 |
| μ/mm$^{-1}$ | 0.773 |
| F(000) | 360.0 |
| Crystal size/mm$^3$ | 0.14 × 0.07 × 0.06 |
| Radiation | Cu Kα (λ = 1.54184) |
| 2Θ range for data collection/° | 6.96 to 146.184 |
| Index ranges | $-8 \le h \le 8, -10 \le k \le 9, -16 \le l \le 16$ |
| Reflections collected | 8993 |
| Independent reflections | 3129 [$R_{int}$ = 0.0233, $R_{sigma}$ = 0.0278] |
| Data/restraints/parameters | 3129/0/240 |
| Goodness-of-fit on $F^2$ | 1.051 |
| Final R indexes [I>=2σ (I)] | $R_1$ = 0.0378, $wR_2$ = 0.0910 |
| Final R indexes [all data] | $R_1$ = 0.0440, $wR_2$ = 0.0950 |
| Largest diff. peak/hole / e Å$^{-3}$ | 0.27/-0.20 |

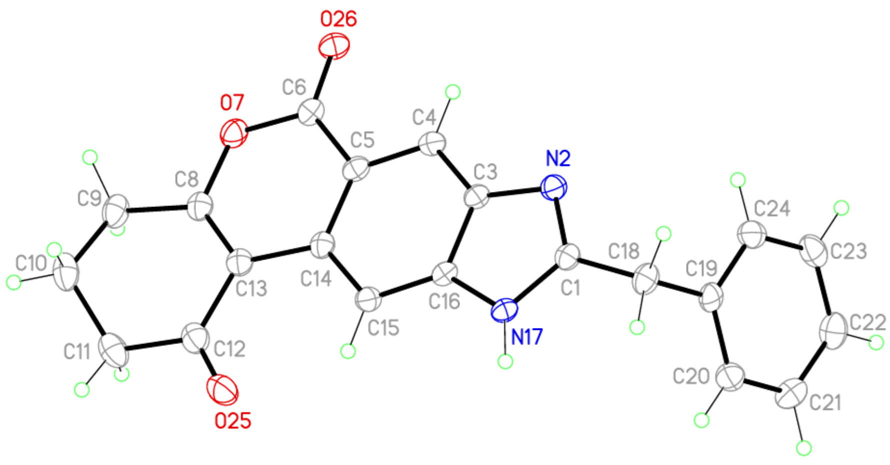


**Figure S310.** Molecular structure of **4a**, with the atomic displacement plot shown at 50% probability.

## References


1. Arya, C. G.; Munugala Chandrakanth; K. Fabitha; Neethu Mariam Thomas; Bhargava Sai Allaka; Srinivas Basavoju; Sonyanaik Banoth; Janardhan Banothu. Sodium Fluoride-Assisted, Solvent-Controlled Regioselective Synthesis of 2-Substituted and 1,2-Disubstituted Benzimidazoles with Diverse Substituents, and Unveiling Mechanistic Insights. *J. Mol. Struct.* **2024**, *1306*, 137935–137935.
2. Yamazaki, S. Chromium(VI) Oxide-Catalyzed Benzylic Oxidation with Periodic Acid. *Org. Lett.* **1999**, *1* (13), 2129–2132.
3. Giancotti, G.; Cancellieri, M.; Balboni, A.; Mariateresa Giustiniano; Novellino, E.; Delang, L.; Johan Neyts; Pieter Leyssen; Brancale, A.; Bassetto, M. Rational Modifications on a Benzylidene-Acrylohydrazide Antiviral Scaffold, Synthesis and Evaluation of Bioactivity against Chikungunya Virus. *Eur. J. Med. Chem.* **2018**, *149*, 56–68.
4. Zhou, H.-J.; Wang, J.; Yao, B.; Wong, S.; Djakovic, S.; Kumar, B.; Rice, J.; Valle, E.; Soriano, F.; Menon, M.-K.; Madriaga, A.; Kiss von Soly, S.; Kumar, A.; Parlati, F.; Yakes, F. M.; Shawver, L.; Le Moigne, R.; Anderson, D. J.; Rolfe, M.; Wustrow, D. Discovery of a First-In-Class, Potent, Selective, and Orally Bioavailable Inhibitor of the P97 AAA ATPase (CB-5083). *J. Med. Chem.* **2015**, *58* (24), 9480–9497.
5. Elham Ghobadi; Hashemi, S. M.; Hamed Fakhim; Zahra Hosseini-khah; Badali, H.; Emami, S. Design, Synthesis and Biological Activity of Hybrid Antifungals Derived from Fluconazole and Mebendazole. *Eur. J. Med. Chem.* **2023**, *249*, 115146–115146.
6. Liu, X.; Dong, Z.-B. Chemoselective Chan–Lam Coupling Reactions between Benzimidazoline-2-Thiones and Arylboronic Acids. *J. Org. Chem.* **2019**, *84* (18), 11524–11532.
7. Andrzejewska, M.; Pagano, M. A.; Meggio, F.; Brunati, A. M.; Kazimierczuk, Z. Polyhalogenobenzimidazoles: Synthesis and Their Inhibitory Activity against Casein Kinases. *Bioorg. Med. Chem.* **2003**, *11* (18), 3997–4002.
8. Ananthakrishnan, S. J.; Kumar, B. S.; Somanathan, N.; Mandal, A. B. Supramolecular Assembly in Side-Chain Conjugated Thiophene Copolymers. *RSC Adv.* **2013**, *3* (22), 8331.
9. Qi, S.-L.; Liu, Y.-P.; Li, Y.; Luan, Y.-X.; Ye, M. Ni-Catalyzed Hydroarylation of Alkynes with Unactivated β-C(Sp2)−H Bonds. *Nat. Commun.* **2022**, *13* (1), 2938–2938.
10. Jiang, Y.; Li, P.; Zhao, J.; Liu, B.; Li, X. Iodonium Ylides as Carbene Precursors in Rh(III)-Catalyzed C–H Activation. *Org. Lett.* **2020**, *22* (19), 7475–7479.
11. Nicolaou, K. C.; Montagnon, T.; Baran, P. S.; Zhong, Y.-L. Iodine(V) Reagents in Organic Synthesis. Part 4.o-Iodoxybenzoic Acid as a Chemospecific Tool for Single Electron Transfer-Based Oxidation Processes. *J. Am. Chem. Soc.* **2002**, *124* (10), 2245–2258.

12. Lau, C. K.; Dufresne, C.; Belanger, P. C.; Pietre, S.; Scheigetz, J. Reductive Deoxygenation of Aryl Aldehydes and Ketones and Benzylic, Allylic, and Tertiary Alcohols by Zinc Iodide-Sodium Cyanoborohydride. *J. Org. Chem.* **1986**, *51* (15), 3038–3043.
